# Physiologically relevant forms of Tc- and Re-pyrophosphate radioactive tracers and the basis of their transthyretin amyloid sensitivity

**DOI:** 10.1038/s41598-026-35746-5

**Published:** 2026-01-24

**Authors:** Kevin Zsolt Simon, Kende Attila Béres, Attila Farkas, Nándor Papp, Andrea Bodor, Veronika Harmat, Dávid Papp, Maria Gracheva, Máté Sulyok-Eiler, András Perczel, László Kótai, Dóra K. Menyhárd

**Affiliations:** 1 Laboratory of Structural Chemistry and Biology, Institute of Chemistry, ELTE Eötvös Loránd University, Budapest, Hungary; 2 ELTE Hevesy György PhD School of Chemistry, ELTE Eötvös Loránd University, Budapest, Hungary; 3 HUN-REN-ELTE Protein Modeling Research Group, ELTE Eötvös Loránd University, Budapest, Hungary; 4 Institute of Materials and Environmental Chemistry, HUN-REN Research Centre for Natural Sciences, Budapest, H-1117 Hungary; 5 Institute of Chemistry, ELTE Eötvös Loránd University, 1117-Budapest, Pázmány P. s. 1/A, Budapest, Hungary; 6 Department of Organic Chemistry and Technology, Faculty of Chemical Technology and Biotechnology, Budapest University of Technology and Economics, Müegyetem rkp. 3., Budapest, H-1111 Hungary; 7 ELTE, Eötvös Loránd University, Institute of Chemistry, Analytical and BioNMR Laboratory, Budapest, Hungary; 8 MTA-ELTE Lendület Ion Mobility Mass Spectrometry Research Group, Faculty of Science, Institute of Chemistry, Eötvös Loránd University, Budapest, H-1117 Hungary; 9 HUN-REN Centre for Energy Research, Konkoly-Thege Miklós út 29-33, Budapest, 1121 Hungary

**Keywords:** Chemistry, Physics

## Abstract

${^{99\text {m}}}$Technetium Pyrophosphate (${^{99\text {m}}}$Tc-PYP(Sn)) is a commonly used radioactive tracer, with a long history of use in diagnosing bone-related diseases and a newfound purpose in differentiating ATTR and AL amyloidoses. Despite its ubiquity, basic aspects like its composition and structure are as of yet undetermined, and its method of binding to ATTR amyloid fibrils is likewise hitherto unknown. This complicates the diagnostic process, as it introduces inexplicable losses of sensitivity in some ATTR and AL variants. In this paper we report the results of our comprehensive investigation into the physiologically active structure of Tc-PYP and its closely related, but experimentally more approachable counterpart, Re-PYP, built on a robust theoretical basis and backed up by multiple spectroscopic methods (focusing on the rhenium analogue). We conclude that the Re/Tc-PYP tracers possess a flexible geometry, but ultimately appear as octahedral Re(IV)/Tc(IV) diaqua dipyrophosphate complexes under physiological conditions, and predict that this structure is the reason for the high affinity of $\phantom{0}^{99m}$Tc-PYP for certain amyloids.

## Introduction

${^{99\text {m}}}$Technetium Pyrophosphate (${^{99\text {m}}}$Tc-PYP(Sn)) has long been an important radioactive tracer in bone scintigraphy, with a newfound purpose in the differentiation of cardiac amyloidosis^1,2^ originating from the aggregation and insoluble fibril formation of the light chain of immunoglobulin antibodies (AL)^3^ and from those of transthyretin (ATTR)^4^. In diagnostic practice, $^{\text {99m}}$Tc-containing tracers are synthesized in situ, via the addition of freshly generated pertechnetate to a reaction vial containing stannous chloride and sodium pyrophosphate. However, despite its long use as a tracer, the structure and even the constitutional formula of the emerging species is hitherto unclear (which is explicitly stated on the commercial Tc-PYP kit instructions provided to physicians). It is presumed that the pyrophosphate forms a mono- or multi-dentate chelate with the radioactive metal atom, thereby binding it to calcium deposits. Yet, the extent of the calcifications seen in cases AL versus ATTR amyloidoses do not always correlate to the difference in technetium uptake, prompting the question whether the tracer is (at least in part) bound directly to the amyloid fibrils^5^. To propose a robust model that describes its properties, one must first determine the exact structure of the physiologically active form of technetium pyrophosphate, which is elucidated in this paper, through its rhenium analogue.

Technetium and Rhenium have equal Pauling electronegativity values, as well as near equal radii. This causes them to be chemically similar in most cases, thus rhenium is commonly used as a safer and easier to acquire substitute for technetium^6–10^. Some isotopes of rhenium itself are often employed in similar tracer complexes^11–13^, and very recently rhenium pyrophosphate itself was evaluated for use as a technetium pyrophosphate replacement^14^, and was found to have similar tissue uptake. However we note that Tc and Re have dissimilar redox properties^12^: rhenium has a much lower standard reduction potential than technetium, which often complicates the synthesis of rhenium analogues of technetium radiopharmaceuticals, as these usually begin with the reduction of M(VII) oxides. This article focuses on the synthesis, theoretical and experimental characterization of Re-PYP; although the complexes discussed below were also calculated with technetium in all cases, and no significant deviation in structure or properties was observed from those of the corresponding species calculated with rhenium.

**Fig. 1:**
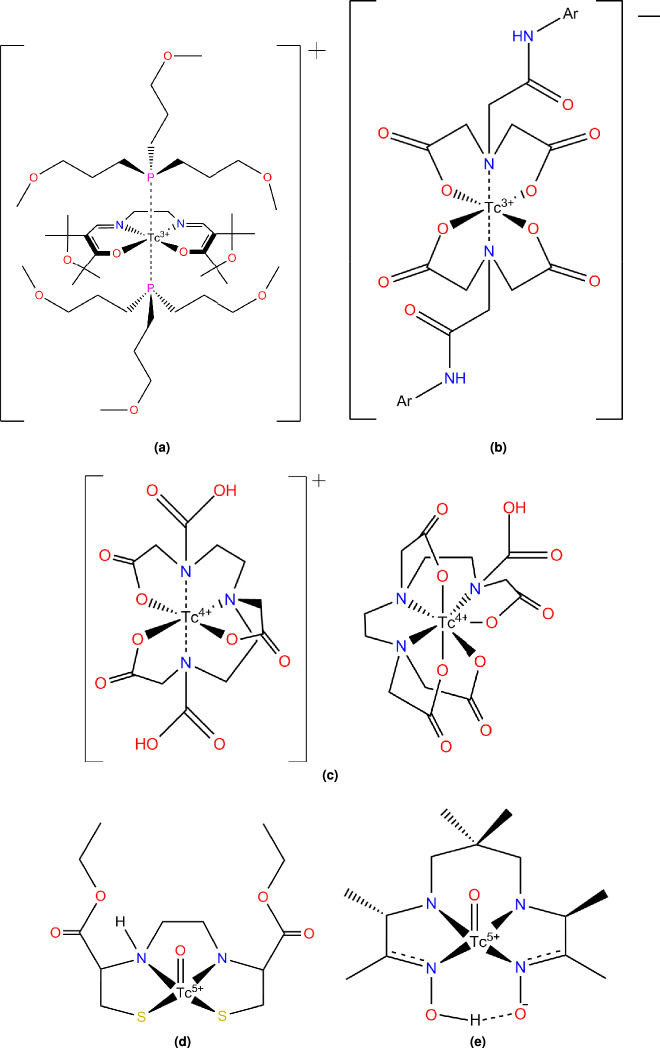
Various technetium-containing radiotracers: 1a Tc(III)-Furifosmin, 1b Tc(III)-Iminodiacetic acid (IDA), 1c Tc(IV)-Diethylenetriamine pentaacetate (DTPA), 1d Tc(V)-ethylene cysteine dimer (ECD), and 1e Tc(V)-hexamethylpropylene amine oxime (HMPAO).

Diverse organic and inorganic chelates and complexes of technetium and rhenium were previously shown to form, including the nonacoordinated nonahydridotechnetate^15^, pentacoordinated and heptacoordinated oxo-rhenium(V) species^16^, rhenium(III)-carboxylates and -chlorides containing metal-metal bonds^17^, as well as metallopolymer structures including rhenium(IV)-tetrachloride^18^. This wide array of possible derivatives and the relatively inexpensive and simple production of $^{\text {99m}}$Tc made these two elements favored in radiodiagnostics, with their most common representatives being M-O and/or M-N chelates where the metal is present as a cation of different (I-VII) oxidation states. Examples such as Tc(III)-furifosmin^9,19^, Tc(III)-IDA^19–21^, and Tc(IV)-DTPA^19,20^ feature dative bonds, while Tc(V)-ECD^19,20^ and Tc(V)-HMPAO^19,20^ counteract the electron-poor nature of the central metal cation instead by the forming a metal-oxygen double bond (Fig. 1).

Phosphates display a degree of delocalization in their electronic structure, with all phosphorous-oxygen bonds having near identical lengths, and this carries over to condensed phosphates (such as pyrophosphate), where all “terminal oxygen bonds” have equal $\pi$-character and length, while “bridging oxygen bonds” are slightly longer. Diphosphonates, such as MDP (methylene diphosphonate, or medronate), HMDP (hydroxymethylene diphosphonate), or DPD (3,3-diphosphono-1,2-propanodicarboxylate) have less of a delocalized character because of the carbon replacing the bridging oxygen. Phosphates and pyrophosphates can form ionic salts or complexes (or chelates) with highly polarized metal-oxygen bonds, even in the same system at different pHs.

Difficulties in analyzing transitional metal pyrophosphates such as these lie in the propensity of the metal cation to reoxidize while standing on air, the propensity of pyrophosphates to hydrolyze when heated or left to stand in acidic aqueous conditions, and the hygroscopic nature of pyrophosphates.

The structure of the${^{99m}}$Tc-PYP radiotracer has been studied previously^22–25^. Diagnostic Tc-PYP is formed in situ via the reduction of pertechnetate by tin(II)-chloride in a water-based solution of pyrophosphoric acid at pH 6.5–7.5.5.5. The oxidation state of the central metal atom was found to be IV by Owunwanne et al^22^, who report that tin(II)-chloride is unable to further reduce Tc, and also note that the complex is not readily oxidized on air. The oxidation state also depends on the complexing ligands and pH, according to Vanlić-Razumenić et al^23^.

The elemental composition has also been reported on, by Kroesbergen et al, in their chromatography experiments: four main complexes are formed, $\hbox {Tc}_n$, two isomers of $\hbox {TcPYP}_2$ (one at neutral and one at acidic pH), and TcPYP, at differing levels of acidity and reagent ratios. At blood pH ($\sim$7.4), the complex contained two pyrophoshate moieties for every Tc ion, and the addition of Sn(II) did not increase the amount of, or type of complex formed, acting — seemingly — only as a reducing agent^24,25^.

Of rhenium (IV), we also know that it forms from perrhenate when heated with Sn(II) and an alkaline-chlorine salt in hydrochloric acid^26^. The resulting $\hbox {ReCl}_6 ^{-2}$ undergoes ligand exchange in neutral (Pavlova et al^27^) (Equation 2) or alkaline (Maun et al^28^ and Rulfs et al^29^) (Equation 1) aquatic solution, forming a heteroleptic aqua complex which can be written as Re(OH)$\phantom{0}_4$($\hbox {H}_2$O)$\phantom{0}_2$, which quickly converts to $\hbox {ReO}_2$. Technetium and rhenium have been shown to form complexes with coordinated aqua ligands^21,30^.

1 $$\begin{aligned} & {[}\text {ReCl}_6{]}^{2-} \xrightarrow [-6\text {Cl}^-]{3\text {OH}^{-}+3\text {H}_2\text {O}} [\text {Re(OH)}_3(\text {H}_2\text {O})_3]^+ \mathop {\rightleftharpoons }\limits ^{\text {OH}^{-}}_{\text {H}^+} [\text {Re(OH)}_4(\text {H}_2\text {O})_2] \longrightarrow \underline{\text {ReO}_2} + 4 \text {H}_2\text {O} \end{aligned}$$

2 $$\begin{aligned} & {[}\text {ReCl}_6{]}^{2-} + (4-n)(\text {H}_2\text {O}) + (n)\text {OH}^{-} \rightleftharpoons \text {Re(OH)}_4 + (4-n)\text {H}^+ + 6\text {Cl}^- \longrightarrow \underline{\text {ReO}_2} + 2 \text {H}_2\text {O} \end{aligned}$$

Here we report the possible structures and properties of the form of the common Tc-PYP radiotracer that is most likely to be present in pH neutral aqueous solutions (which we call its physiologically active form), based on our theoretical (DFT and XTB) and experimental (IR, Raman, UV-Vis, NMR, ESI-MS, and Sn-Mössbauer) results, as well as a preliminary model of its possible interaction with wild-type transthyretin amyloid fibrils. It is important to stress that our goal here was not to clarify the chemistry of the Tc- or Re-pyrophosphate complexes, but to propose a plausible model of their composition, configuration and conformation under physiological settings and to test whether tracers in this form would able to interact directly with the targeted fibrils.

## Methods

*Theoretical calculations:* Density Functional Theory calculations were done using Schrödinger’s Jaguar program^31^, on the B3LYP/LACVP**$$\phantom{0}^+$$^32–36^ or B3LYP-D3/LACV3P**$$\phantom{0}^{++}$$^37–40^ levels of theory as indicated below. The isomer search was performed with ORCA’s Global Optimizer Algorithm (GOAT)^41–43^ in conjunction with the GFN2-xTB (XTB2) semi-empirical method^44,45^. No filtering was applied based on energy or rotational constant. Entropy maximization was applied. The bonds of the pyrophosphoric acid and aqua ligands were constrained. Vibrational analysis was used to confirm true minima for structures calculated via both above theoretical methods.

*Synthesis:* Synthesis of the samples was done as follows: 23.948 g of tetrasodium-pyrophoshate-decahydrate (REANAL) was dissolved in 120 ml of 1M HCl, along with 1.816 g tin(II)-chloride (Alfa Aesar) except in the case of Mixture S (see below). 60 ml of 1M NaOH was then added to bring the pH to $\sim$6, except in the case of Mixture S where no pH adjustment was needed. In the case of Mixture P, 0.736 g of sodium-perrhenate (VWR Chemicals) was dissolved in 20 ml of 0,1 HCl and added to the stirred mixture, while in the case of Mixture H and Mixture S, 1.283 g of the pre-reduced potassium hexachlororhenate (see below) was added instead.

*Deuteration:* Deuterated samples were synthesized in the following way: due to the sensitivity of the complex to dehydration, samples were prepared via dilution of concentrated HCl and solid NaOH by $\hbox {D}_2$O. Because of this, a mixture of H and D is present in the recorded spectra. The samples synthesized with $\hbox {D}_2$O were then precipitated by the dropwise addition of deuterated (D4) methanol, and left to dry in a desiccator for four days.

*UV-Vis spectroscopy:* UV-Vis spectra were taken using a Jasco V-670 spectrometer.

*IR spectroscopy:* Fourier transform infrared (FT-IR) spectra of the samples were recorded using a Bruker Tensor 27 Platinum FT-IR spectrometer (resolution: 2 $\hbox {cm}^{-1}$) in the attenuated-total-reflection (ATR) mode, between 4000 and 400 $\hbox {cm}^{-1}$.

*Mössbauer spectroscopy:* $\phantom{0}^{119}$Sn Mössbauer spectra were recorded at room temperature in transmission geometry by using a standard WissEl Mössbauer spectrometer along with a $\phantom{0}^{119m}$Sn($\hbox {CaSnO}_3$) radioactive source with an activity of $\sim$ 1.8 mCi. The source movement followed a sinusoidal velocity signal. Unfolded spectra were recorded in 2048 channels and were subsequently folded into 1024 channels for analysis. Isomer shift ($\delta$) values are quoted with respect to an $\hbox {SnO}_2$ reference (Merck) whose isomer shift can be taken to be equal to that of the $\hbox {CaSnO}_3$ source matrix. The velocity axis was calibrated by measuring the reference $\hbox {SnO}_2$ ($\delta$ =0 mm $\hbox {s}^{-1}$) powder together with $\beta$-Sn ($\delta$ = 2.56 mm $\hbox {s}^{-1}$). The obtained spectra were fitted to Lorentzian quadrupole doublets by using version 4.0i of the MossWinn program^46^.

*Mass spectrometry:* Direct injection ESI-MS and HILIC-ESI-MS analysis were performed on a Thermo Scientific Q Exactive Focus, high resolution and high mass accuracy, hybrid quadrupole-orbitrap mass spectrometer (Bremen, Germany) using on-line UHPLC coupling. HILIC separation was performed on a Dionex 3000 UHPLC system using a Waters Acquity UPLC BEH Amide column (2.1 x 150 mm, 1.7 $\upmu$m). Linear gradient elution (0 min 95% B, 1.0 min 95% B, 14.0 min 50% B, 14.1 min 40% B, 15.0 min 40% B. 15.1 min 95% B, 20 min 95% B) with eluent A (0,1% FA in water) and eluent B (0,1% FA in acetonitrile) was used at a flow rate of 0.400 mL/min at 60 $\phantom{0}^{\circ }$C column temperature. High resolution mass spectra were acquired in the 50–750 m/z range with polarity switching. The HRMS was operated in both positive and negative ionization mode with identical parameters except for the spray voltage: sheath gas flow rate: 52.5, auxiliary gas flow rate: 13.75, sweep gas flow rate: 2, capillary temperature: 300 $\phantom{0}^{\circ }$C, auxiliary gas heater: 435 $\phantom{0}^{\circ }$C, spray voltage: 3.5 kV and 2.5 kV respectively. Direct injection measurements was acquired at a flow rate of 20 μL/min for 0.5 min.

*Single crystal crystallographic studies (SCXRD):* Structure solution was used for determining chemical composition of the crystals. Single crystal X-ray diffraction data were collected on a Rigaku XTALab Synergy-R diffractometer using Cu-K$\alpha$ radiation ($\lambda$=1.54184 Å). Data collection and data processing was performed using CrysAlisPro (v.1.171.40.68a, Rigaku). Structures were solved and refined using Olex2 program^47^. The phase problem was solved using the intrinsic method with the SHELXT program^48^. The structures were refined by full-matrix least-squares techniques on $\hbox {F}^2$ (SHELXL-2018/3)^49^. Hydrogen atoms were refined in the riding positions. Figures were created using Mercury^50^. Validation was carried out using CheckCIF/PLATON^51^. Crystallographic data and the structures solved for tetrasodium pyrophosphate decahydrate as well as potassium perrhenate are presented in Supplementary Information under “Single crystal X-ray diffraction”. The structures were deposited in the Cambridge Structural Database under accession codes CCDC 2473193 and CCDC 2473194, respectively.

*MCMM docking:* Monte Carlo multiple minimum (MCMM) conformational search was carried out using the Schrödinger Suite^52^. Starting structure was retrieved from the Protein Data bank (PDB:8ADE) and missing segments (the flexible loop connecting Lys35 and Gly57) were rebuilt and minimized. The B3LYP-D3/LACV3P**$\phantom{0}^{++}$ derived structure of the Tc-Pyp complex was used along with the ESP partial charges (also from the calculation, after symmetrization, so that identical atom types carried identical charges). Tc was modeled as a generic hexavalent metal ion, with oxidation state of IV. The OPLS4 forcefield^53^ was used with continuum solvation using $\varepsilon$=80. Internal torsions of the diaqua-TcPyp complex and its relative distance and orientation with respect to the protein were randomly altered and the resultant structures minimized in each step. Minimization concerned the tracer complex and the sidechains of residues 58, 59, 61, 54, 66, 70, 72, 75, 77, 80, 81. Therefore, the experimentally determined fold (backbone structure) of the amyloid fibril did not change during the calculations.

*NMR spectroscopy:* Samples were prepared in standard 5 mm NMR tubes with a total volume of 600$\upmu$L, containing 10% $\hbox {D}_2$O. NMR measurements were performed on a Bruker Avance Neo 400 MHz spectrometer (operating at 161.99 for 31P) equipped with an automatic sample changer and a PI HR-BBO400S1-BBF/H/O-5.0-Z SP probe-head. The ^31^P NMR spectra were recorded using the standard zgpg pulse sequence at 298 K. Typical parameters were: 50 ppm spectral width (SW), transmitter offset (O1P) –5 ppm, and 32k time domain (TD) points, number of transients 64, relaxation delay (d1) 5 s. Data processing, integration and evaluation was done in TopSpin 4.3.0 software. Sample P contained 12 mM $\hbox {NaReO}_4$, 36 mM $\hbox {SnCl}_2$, and 240 mM pyrophosphoric acid; sample H contained 12 mM $\hbox {K}_2$ $\hbox {ReCl}_6$, 36 mM $\hbox {SnCl}_2$, and 240 mM pyrophosphoric acid; sample S contained 12 mM $\hbox {K}_2$ $\hbox {ReCl}_6$ and 240 mM pyrophosphoric acid.

*Raman spectroscopy:* A Horiba Jobin-Yvon LabRAM type microspectrometer was used for recording Raman spectra. A 785 nm diode laser ($\sim$80mW) and a 532 nm frequency-doubled Nd:YAG ($\sim$40mW) excited the molecules, while the samples were cooled to 123 K with a Linkam THMS600 temperature control stage using liquid nitrogen and 20$\times$ objective of an Olympus BX-40 optical microscope focused the beam onto the surface. Optical gratings with 950 and 1800 lines/mm were used to disperse the scattered light, enabling the detection of Raman peaks in the 2000–200 $\hbox {cm}^{-1}$ and 4000–100 $\hbox {cm}^{-1}$ spectral ranges, respectively, for diode and Nd:YAG lasers. Measurement times of 60–120 seconds were set to obtain Raman peaks with sufficient intensity.

*Comparative analysis of amyloid topologies:* 922 protofilaments were extracted from 477 amyloid structures from the Protein Data Bank^68^. For each 11 types of protein family separately protofilaments were clustered based on RMSD values and overlapping sequence length. Ward’s method was used for agglomerative clustering, the cut off value was 30% of the total variance for each protein family.

*Figures:* Figures were generated using Schrödinger Maestro, Gnuplot, and respective spectroscopy software packages.

## Theoretical investigations

In light of all previous studies^22–28^, the most likely structure of Tc-PYP at physiological (pH neutral, aqueous) conditions is a trans octahedral metal-dipyrophoshate with two additional oxygen-containing or halogen-ion ligands which most likely occupy the apical positions due to the bulkiness of the pyrophoshate moities.

**Fig. 2:**
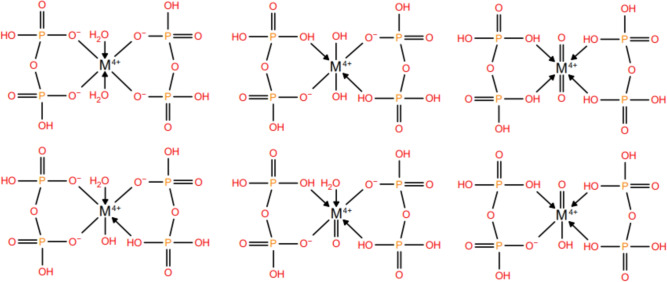
Investigated tautomeric variants of the proposed metal-dipyrophosphate structure.

Assuming a metal-oxygen octahedron and semi-protonated pyrophosphoric acid ligands at reaction pH, six tautomers can be considered (Fig. 2). Of these, geometric optimization on the B3LYP/LACVP**$\phantom{0}^+$ level of theory resulted in the protonation of the apical oxygen ligands when those ligands were hydroxides, and the formation of $\hbox {ReO}_2$ when either of them were oxo groups (which would disrupt the formation of the tracer as $\hbox {ReO}_2$ is insoluble). No rhenyl^54^ geometries were found to be stable at this oxidation state. Hydroxide ligands, in particular, result in a metastable metal complex, where if these oxygen ligands stay deprotonated, $\hbox {ReO}_2$ would again form through the formation of oxo groups.

This diaqua structure is in line with the proposed structure and stability of rhenium’s heteroleptic aquacomplex.

The resulting Re/Tc-diaqua-dipyrophosphate complexes were refined in vacuum at the B3LYP-D3/LACV3P**$\phantom{0}^{++}$ level of theory, uncharged and with doublet multiplicity. This form appears similar to the respective aquacomplexes of these metal ions, but are energetically more favorable due to the chelating effect of the multidentate ligands, the larger $\pi$-donating effect of the electron rich phosphate tetrahedra, and presence of stabilizing intramolecular hydrogen bridges. These hydrogen bridges appear between 3200 and 2500 $\hbox {cm}^{-1}$ on calculated IR spectra, when the protonation state of the pyrophosphate moieties match the protonation state at serum pH ($\sim$7.4).

Similar metal-diaqua-dipyrophosphate structures have been reported in literature^55^, including Cu(II), Zn(II), Mg(II), Ni(II), Co(II), and even Mn(II) derivatives, although no higher oxidation value transitional metal cations have yet been shown to form this kind of geometry.

**Fig. 3:**
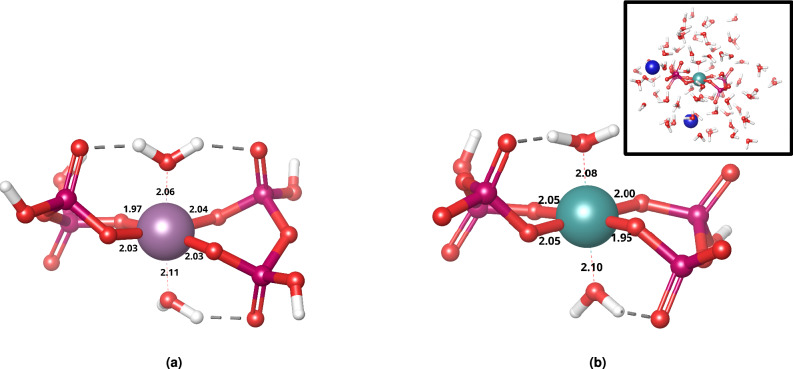
Structure of Re-PYP refined in vacuum, one of many possible conformers. Rhenium is shown in purple; P, O, and H are shown in magenta, red, and white. Structure of Tc-PYP refined via an explicit hydrate shell. Technetium is shown in teal, sodium is shown in blue. Displayed bond lengths are in Å.

It should be noted, that because of the inherent flexibility of the pyrophosphate ligands, the conformational space of this complex appears quite wide and flat, with notable variations in the number of H-bridges which result in the fluctuation of the M-O(PYP) bond lengths between 1.9–2.1 Å and the M-Aqua bond lengths between 2.0–2.3 Å (Fig. 3/a). This conformational freedom also introduces inherent deviation into calculated properties.

In order to confirm the stability of the calculated Re/Tc-diaqua-dipyrophoshate structure in solution, the complex was solvated explicitly (with full DFT treatment), with the protonation state of the pyrophosphates matching their preferred protonation state around blood pH ($\sim$7.4), that being $\hbox {HP}_2{{\hbox {O}_7}^{3-}}$. There was little change in the geometry (Fig. 3/b) (well within the range of its conformational freedom), and no deprotonation was observed on the aqua ligands.

### Octahedral monoaqua and heptacoordinated triaqua forms

**Fig. 4:**
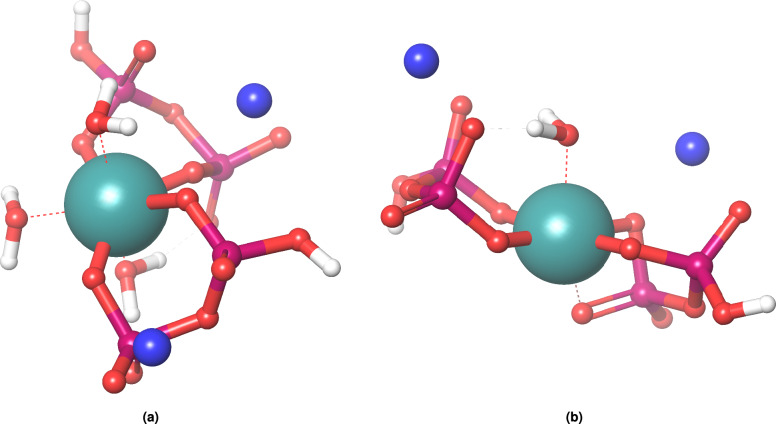
Triaqua ($\hbox {TcPYP}_2$($\hbox {H}_2$O)$\phantom{0}_3$) and monoaqua ($\hbox {TcPYP}_2$($\hbox {H}_2$O)) forms of Tc-PYP. Technetium is shown in teal, sodium is shown in blue; P, O, and H are shown in magenta, red, and white.

Two additional water containing forms are predicted to be stable by our calculations (Fig. 4).

Being electron poor, it is most likely that the central metal undergoes associative ligand exchange when exchanging its aqua ligands with the bulk solution. The resulting heptacoordianted triaqua complex was found to have a stable minimum, with it being 52 kJ/mol higher in energy. This structure is quite distorted, far from an ideal pentagonal bipyramidal geometry. The M-Aqua bonds have lengthened to 2.14–2.22 Å, and the M-O distances to 1.97–2.20 Å.

Surprisingly, we also found a monoaqua form, with one of the pyrophosphate moieties substituting the nonbonding electron pairs on the missing water, thus becoming tridentate. Due to the size of the phosphate tetrahedra compared to the M-O bond lengths, this geometry is also highly distorted, which leads to its energy being more than 250 kJ/mol higher than that of the diaqua form. Additionally, we observed in our calculations that the monoaqua form was prone to have its aqua ligand deprotonated (due to the steric constraint of the size of the bonding phosphate tetrahedron), in which case the pyrophosphates became again tridentate and a tertagonal piramidal complex was formed.

While no experimental results distinctly show such monoaqua or triaqua variants, the possible existence of these two other forms near neutral pH should be kept in mind when investigating the interaction of Tc-PYP with proteins, as they further increase the plasticity of this tracer when it comes to occupying binding sites of greatly differing sizes, polarities, and solvent accessibilities.

The possibility of a tetrahedral geometry for the complex was also investigated, but no such stable geometries were found, instead the denticity of the pyrophosphate ligands increased and the complex became octahedral (such as can be seen on Fig. 4).

It is also possible that the active form of Tc-PYP is a metallo-polymer/oligomer, as was seen in the single crystal XRD structure obtained for another member of this class of complexes, that of Tc-MDP^56^, but is it unlikely, as the higher degree of delocalization makes PYP a much better $\pi$ electron donor. Due to this, and to the relatively low concentration of pyrophosphoric acid and pyrophosphate anions versus water or hydroxide ions in physiological settings, which also disfavors polymer formation, these structures were not investigated.

*Additional theoretical results, calculated charge distribution, other possible reaction products *^19,57,58^, *and the theoretical structure of tin(IV) pyrophosphate *^59^ *are available in the Supplementary Information under “Additional theoretical results”.*

## Calculated spectroscopic properties

### Calculated excitational spectroscopic results

The UV-Vis absorption wavelengths for the identified stable species afforded by our calculations can be seen in Table 1.

Of note is the tendency of the HOMO-LUMO energy gap to change in the range of 410–450 nm in function of the number and strength of intramolecular hydrogen bridges present, and the possible geometries of the conformers dictated by them. These changes can be brought on by changing the acidity of the medium (see Section 5.1), but are also dependent on the conformer distribution of the complex, resulting in a wider band of absorption in UV-Vis spectra.

**Table 1:** HOMO-LUMO gaps of calculated structures along with their associated absorption wavelenghts. Absorption corresponding to the highlighted species was experimentally measured.

	Alpha			Beta		
	HOMO (E_h_)	LUMO (E_h_)	$\frac{hc}{\Delta E}$(nm)	HOMO (E_h_)	LUMO (E_h_)	$\frac{hc}{\Delta E}$(nm)
$\hbox {RePYP}_2$($\hbox {H}_2$O)$\phantom{0}_2$	−0.274474	−0.170461	438.0	−0.263798	−0.156369	424.1
$\hbox {RePYP}_2$($\hbox {H}_2$O)$\phantom{0}_3$	−0.252824	−0.122204	348.8	−0.240359	−0.146543	485.6
$\hbox {RePYP}_2$($\hbox {H}_2$O)	−0.224460	−0.138260	528.6	−0.213960	−0.124530	509.5
$\hbox {TcPYP}_2$($\hbox {H}_2$O)$\phantom{0}_2$	−0.271770	−0.173153	462.0	−0.269072	−0.161358	423.0
$\hbox {TcPYP}_2$($\hbox {H}_2$O)$\phantom{0}_3$	−0.251410	−0.123650	356.6	−0.239280	−0.146080	488.9
$\hbox {TcPYP}_2$($\hbox {H}_2$O)	−0.271100	−0.162270	418.7	−0.258750	−0.153900	434.6
$\hbox {RePYP}_2\hbox {Cl}_2$	−0.254927	−0.183692	639.6	−0.245543	−0.169889	602.2
$\hbox {ReOPYP}_2$	−0.251836	−0.110571	322.5	−0.228622	−0.098923	351.3
$\hbox {SnPYP}_2$($\hbox {H}_2$O)$\phantom{0}_2$	−0.329351	−0.075818	179.7			

### Calculated vibrational spectroscopic results

The calculated vibrational spectra for the rhenium- and technetium-diaqua-dipyrophosphate complexes show little difference, provided that the same conformers are investigated (geometric optimization was done on the technetium analogue, both complex structures are stable minima).

The figure below shows these spectra for the conformer seen in Fig. 3a, at its physiological protonation state, neutralized via sodium ions:

**Fig. 5:**
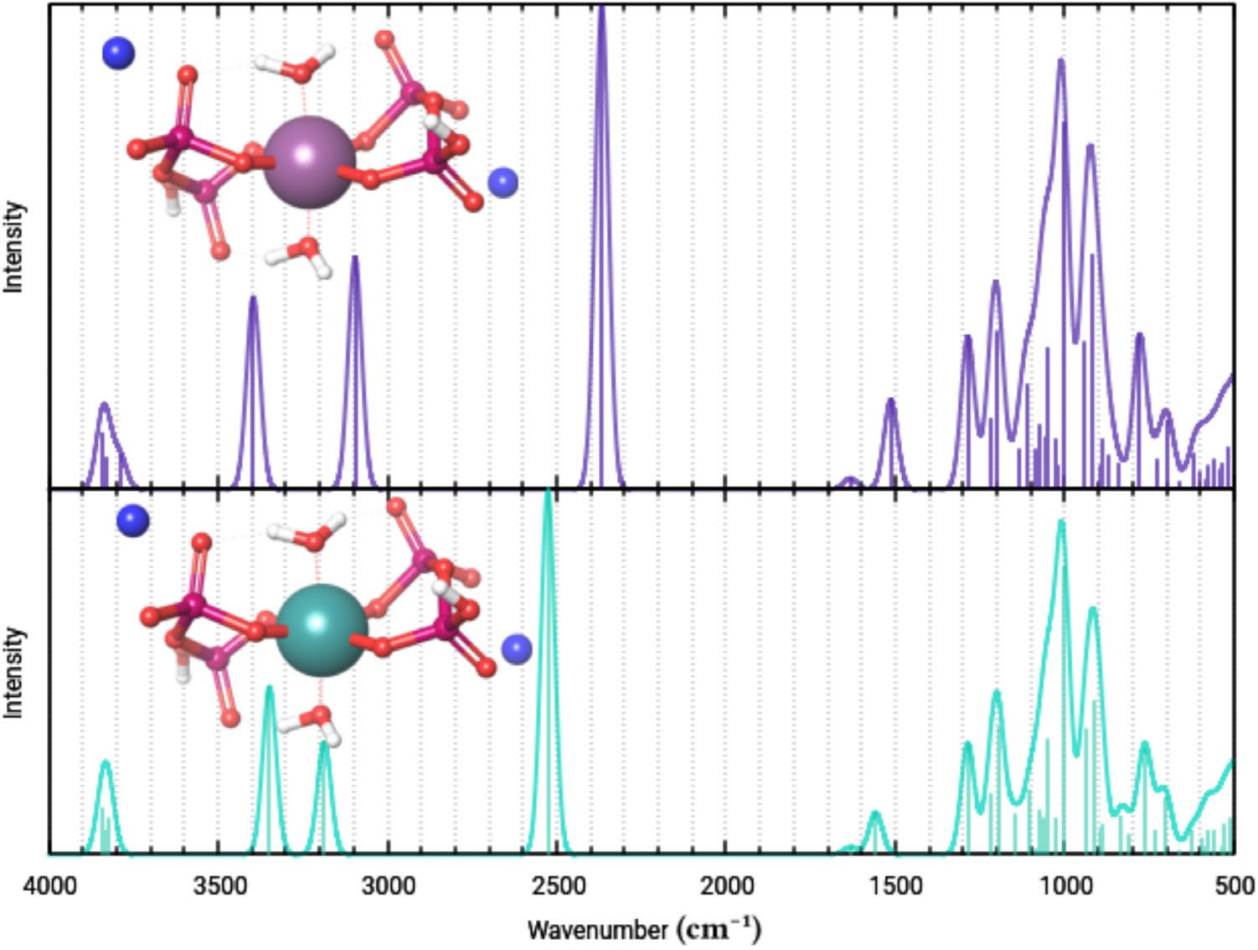
Calculated infrared-active vibrational spectra of one technetium-diaqua-dipyrophosphate conformer and its rhenium analogue. The rhenium-complex is shown in purple, the technetium-complex is shown in teal. Gaussian functions were fitted with a standard deviation of 20.

A trans-XY_4_Z_2_ unit ($\hbox {D}_{4h}$) is expected to give three XY stretching (A_1__g_+B1_g_+E_u_) and two XZ stretching ($\hbox {A}_{1g}$+$\hbox {A}_{2u}$) modes, of which $\hbox {E}_u$ and $\hbox {A}_{2u}$ are infrared-active and $\hbox {A}_{1g}$ and $\hbox {B}_{1g}$ are Raman-active. The studied isostructural Tc and Re complexes have distorted $\hbox {D}_{4h}$ geometries; thus, all of the TcO(4+2) and ReO(4+2) modes are IR and Raman active. Similarly, among the normal modes of diphosphate groups ($\hbox {C}_{2v}$) are 17 IR active in $\hbox {C}_{2v}$, but the symmetry lowering ($\hbox {C}_s$ or $\hbox {C}_1$) causes all 21 normal modes to be IR active (higher symmetry conformers may have different band intensities). Accordingly, the terminal and linking P-O-P modes and the modes of central OC-6 ions, together with the three modes of the coordinated aqua ligands, are expected (see Table 5)^60^.

Three H-bridges are spread across the two coordinated water molecules: where only one water hydrogen takes place in the bonding, its vibrational frequency denotes it as a very strong H-bond (found at 2500 $\hbox {cm}^{-1}$ on Fig. 5), going by the characterization of Hansen and Spanget-Larsen^61^; yet where both hydrogen atoms take place in H-bonding these weaker interactions coincide with nonbridging OH stretching modes (found between 3500 $\hbox {cm}^{-1}$ and 3000 $\hbox {cm}^{-1}$ on Fig. 5). As in similar diaqua-dipyrophosphate structures these apical aqua ligands take part in an intermolecular H-bridge system that stabilizes the crystal, it is unlikely that such lower wavenumber bands can be observed on the IR spectra of any crystals of the complex, but amorphous phases could potentially still exhibit them (as seen below).

The isostructural tin diaqua dipyrophosphate complexes (see the Supplementary Information under section “Additional theoretical results”) also show near identical spectra to the tracers’.

Due to the aforementioned wide and flat conformational space of the tracer’s suspected main physiologically active species, any one generated vibrational spectrum will differ from the experimental spectrum, as the experimental spectrum most likely contains absorption bands from an ensemble of these conformers. Finding those conformers that contribute most to the spectrum is challenging and time-consuming, therefore an Extended Tight Binding GOAT scan was used to automate and shorten the process.

The extended tight binding method was found to accurately approximate the structures calculated on the B3LYP-D3/LACV3P**$\phantom{0}^{++}$ level of theory for this system. Conformers of various Re-PYP species were generated using GOAT^41^, which were then re-optimized as Tc-PYP species, to provide a direct comparison. The few Tc-PYP conformers that did not converge to stable minima, or converged to outlier geometries were pruned from the ensembles. The similarity of these congener species is discussed in the Supplementary Information under subsection “Additional isomeric ensembles” (Figure S4).

Additionally, the generated ensembles provide common attributes — such as bond lengths, angles, and dihedrals — that can be used to parametrize the complex for molecular mechanical applications, such as docking or molecular dynamics. These attributes are (see Tables 2, 3 and 4): the M-OP and M-$\hbox {OH}_2$ bond lengths; the average of all O-M-O angles where both oxygen atoms are on the same pyrophosphate ($\alpha$); the average of all O-M-O angles where the oxygen atoms are on different pyrophosphates ($\beta$); the O-M-$\hbox {OH}_2$ angle between one specific bonding oxygen and aqua ligand ($\gamma$); the aqua-M-aqua angle ($\delta$); the P-O-P angle of the bridging oxygen ($\epsilon$); and finally the O-P-P-O dihedral angle between the phosphate oxygen atoms pointing away from the metal center ($\zeta$). The total number of pyrophosphates in staggered ($\zeta>30^{\circ }$) and eclipsed ($\zeta <30^{\circ }$) conformations present in the ensemble is also displayed.

The two main conformational features by which the Re/Tc diaqua dipyrophosphate complexes can be differentiated are the number of intramolecular hydrogen bridges and the shape and orientation of the M-O-P-O-P-O-M rings. In the previous DFT calculations we found that on average there are three H-bridges between the ligated water donors and the “double bonded” oxygen acceptors, which means that when the pyrophosphates are in staggered conformations, one water will form a bridge with both pyrophosphate ligands, stabilizing the structure further. Yet, there are possible geometries where the pyrophosphates are in eclipsed conformations, in which case the water ligand forms H-bridges with only one pyrophosphate.

Single-crystal X-ray diffraction structures of similar M-diaqua-dipyrophosphates have been reported with eclipsed^55^ pyrophosphate conformations, however different M-aqua-pyrophosphate complexes^62^ also show staggered pyrophosphate conformations, which — along with the small calculated energy difference between these geometries in the case of these technetium and rhenium pyrophosphates — hints at the presence of mixed staggered and eclipsed pyrophosphate geometries in solution, which should be taken into account when protein binding sites are investigated.

**Table 2:** General attributes of the physiological form’s conformational ensembles.

	Average	Min	Max	
Re-PYP (12 conformers)				
M-OP length (Å)	1.98	1.84	2.11	
M-$\hbox {OH}_2$length (Å)	2.18	2.12	2.25	
$\alpha$($\phantom{0}^{\circ }$)	89.32	85.59	96.53	
$\beta$($\phantom{0}^{\circ }$)	90.83	86.16	93.88	
$\gamma$($\phantom{0}^{\circ }$)	83.62	81.54	86.78	
$\delta$($\phantom{0}^{\circ }$)	173.87	170.19	177.21	
$\epsilon$($\phantom{0}^{\circ }$)	117.79	113.28	122.79	
$\zeta$($\phantom{0}^{\circ }$)	52.65	3.43	70.84	
Staggered pyrophosphates:	21	Eclipsed:	3	
Tc-PYP (10 conformers)				
M-OP length (Å)	1.94	1.81	2.07	
M-$\hbox {OH}_2$length (Å)	2.18	2.12	2.24	
$\alpha$($\phantom{0}^{\circ }$)	88.28	85.12	91.35	
$\beta$($\phantom{0}^{\circ }$)	91.72	87.13	95.73	
$\gamma$($\phantom{0}^{\circ }$)	85.05	82.49	88.25	
$\delta$($\phantom{0}^{\circ }$)	174.38	169.85	178.21	
$\epsilon$($\phantom{0}^{\circ }$)	117.96	113.53	122.35	
$\zeta$($\phantom{0}^{\circ }$)	48.13	4.66	58.48	
Staggered pyrophosphates:	17	Eclipsed:	3	

**Fig. 6:**
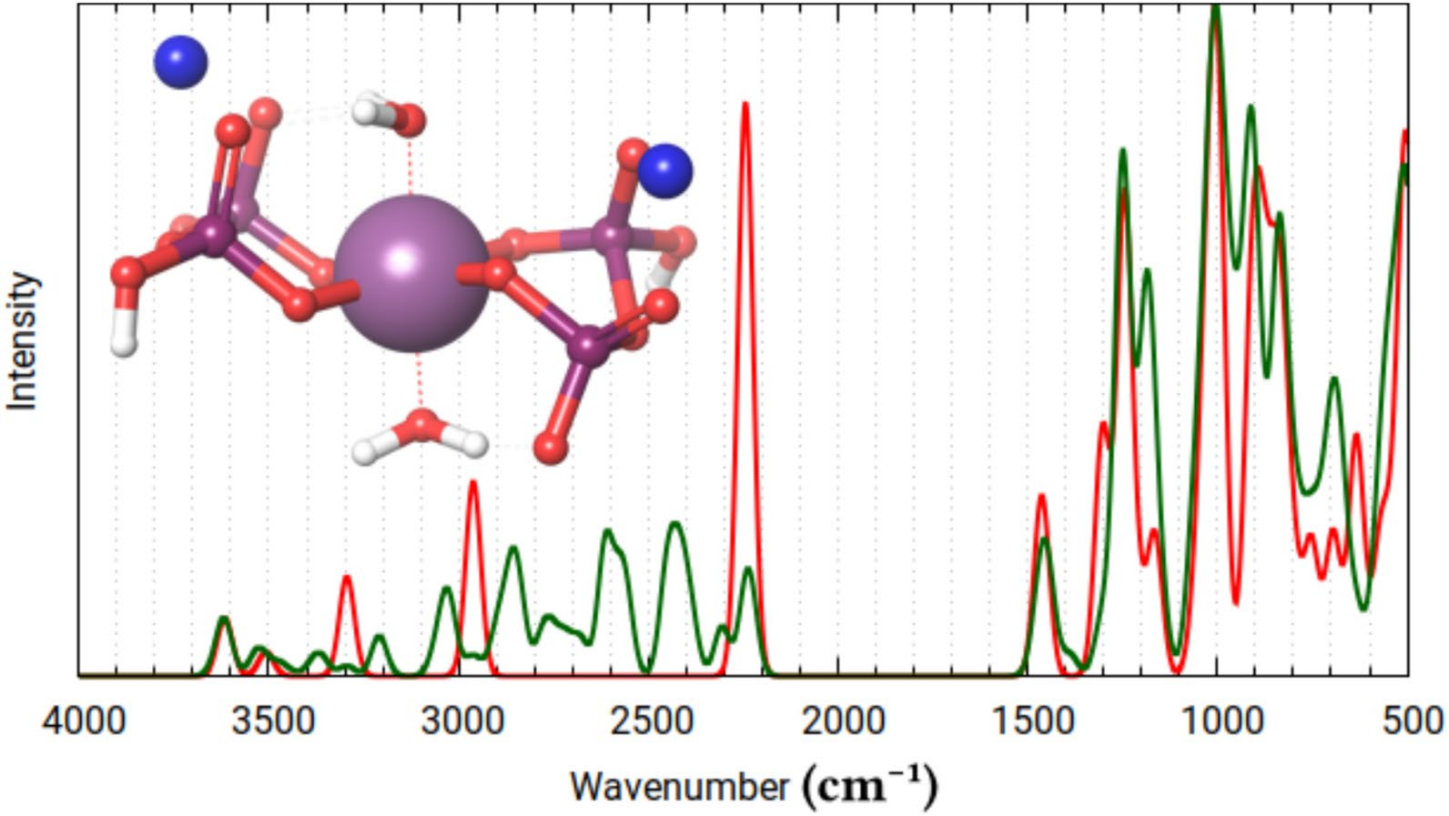
Calculated infrared spectra for the lowest energy conformer (red) and cumulatively all (12) conformers (green), of the physiological form of Re-PYP.

**Fig. 7:**
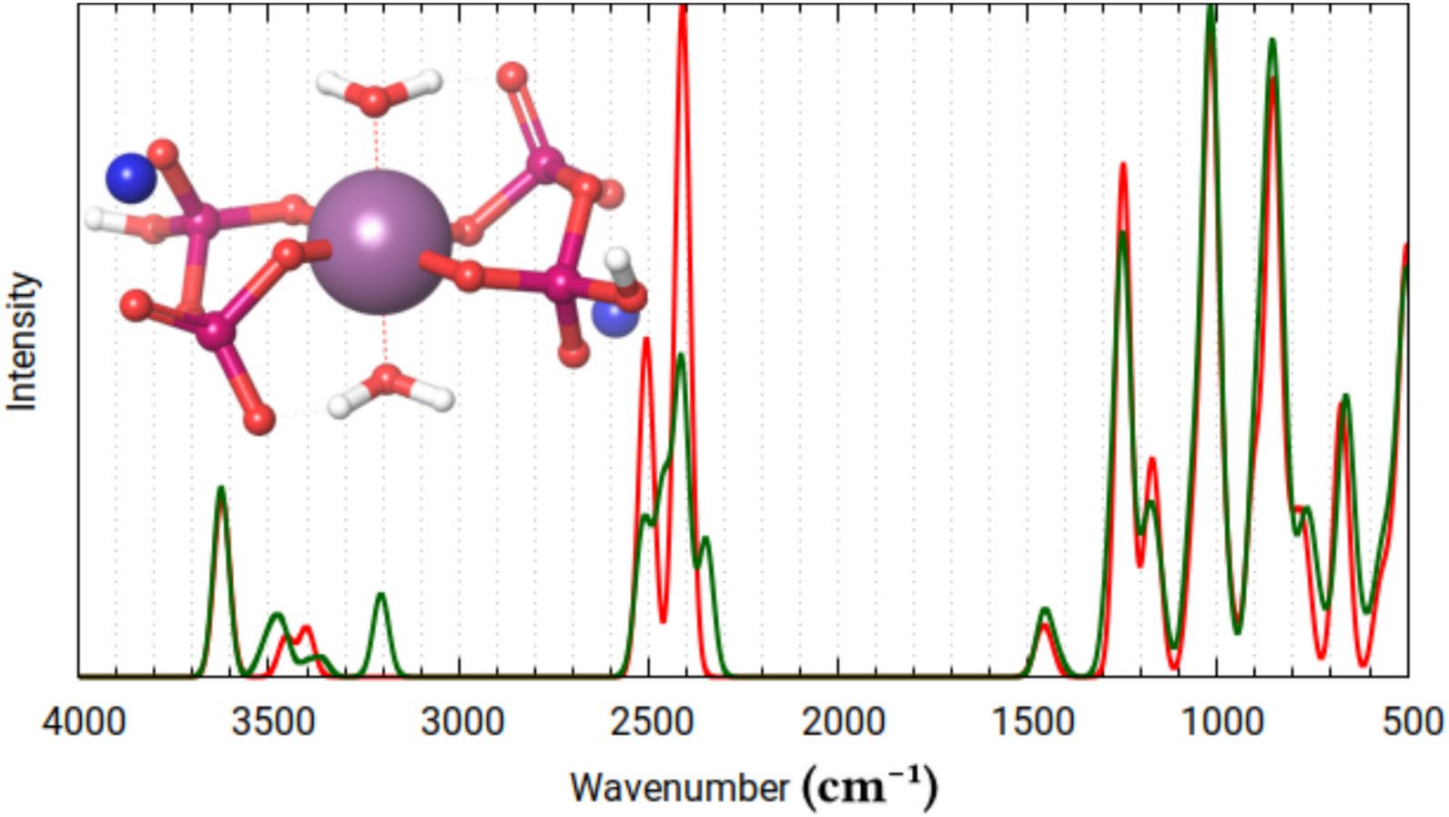
Calculated infrared spectra for the lowest energy conformer (red) and cumulatively all (4) conformers (green), of the symmetrized physiological form of Re-PYP.

The spectra generated for the “physiological form” (Fig. 6) system features split-bands at $\sim$1200 and $\sim$900 cm${^{-1}}$. This is due to the asymmetry of the electron density between the two pyrophosphate ligands, that arises from the geometry, where one of the sodium ions is in contact with two phosphate tetrahedra, while the other is in contact with only one. This splitting is no longer observable when a “symmetric salt” (Fig. 7) is chosen as an initial geometry. This further shows, that the chemical environment can intensively modify the calculated properties of Tc/Re-PYP, even with the complex staying practically the same.

**Table 3:** General attributes of the symmetrized physiological form’s conformational ensembles.

	Average	Min	Max	
Re-PYP (4 conformers)				
M-OP length (Å)	1.97	1.82	2.12	
M-$\hbox {OH}_2$length (Å)	2.17	2.14	2.18	
$\alpha$($\phantom{0}^{\circ }$)	91.44	90.38	90.96	
$\beta$($\phantom{0}^{\circ }$)	88.56	86.37	91.65	
$\gamma$($\phantom{0}^{\circ }$)	87.11	81.85	90.91	
$\delta$($\phantom{0}^{\circ }$)	175.25	174.15	177.53	
$\epsilon$($\phantom{0}^{\circ }$)	117.28	115.02	119.24	
$\zeta$($\phantom{0}^{\circ }$)	53.40	39.56	72.84	
Staggered pyrophosphates:	8	Eclipsed:	0	
Tc-PYP (4 conformers)				
M-OP length (Å)	1.95	1.79	2.10	
M-$\hbox {OH}_2$length (Å)	2.17	2.15	2.19	
$\alpha$($\phantom{0}^{\circ }$)	89.86	88.04	90.00	
$\beta$($\phantom{0}^{\circ }$)	90.14	88.02	94.30	
$\gamma$($\phantom{0}^{\circ }$)	84.77	82.17	87.49	
$\delta$($\phantom{0}^{\circ }$)	175.26	173.53	177.89	
$\epsilon$($\phantom{0}^{\circ }$)	116.98	114.85	118.74	
$\zeta$($\phantom{0}^{\circ }$)	49.27	37.93	68.94	
Staggered pyrophosphates:	8	Eclipsed:	0	

**Table 4:** General attributes of the neutralized form’s conformational ensemble.

	Average	Min	Max	
Re-PYP (12 conformers)				
M-OP length (Å)	2.01	1.82	2.22	
M-$\hbox {OH}_2$ length (Å)	2.29	2.25	2.34	
$\alpha$ ($\phantom{0}^{\circ }$)	95.46	91.67	98.16	
$\beta$ ($\phantom{0}^{\circ }$)	84.56	78.63	88.98	
$\gamma$ ($\phantom{0}^{\circ }$)	88.09	79.64	95.56	
$\delta$ ($\phantom{0}^{\circ }$)	174.83	167.18	179.93	
$\epsilon$ ($\phantom{0}^{\circ }$)	115.76	114.05	117.52	
$\zeta$ ($\phantom{0}^{\circ }$)	61.95	57.31	69.29	
Staggered pyrophosphates:	24	Eclipsed:	0	
Tc-PYP (12 conformers)				
M-OP length (Å)	1.97	1.80	2.19	
M-$\hbox {OH}_2$ length (Å)	2.26	2.20	2.30	
$\alpha$ ($\phantom{0}^{\circ }$)	92.82	90.65	95.64	
$\beta$ ($\phantom{0}^{\circ }$)	87.20	78.35	94.72	
$\gamma$ ($\phantom{0}^{\circ }$)	89.90	83.71	97.32	
$\delta$ ($\phantom{0}^{\circ }$)	176.97	171.89	179.97	
$\epsilon$ ($\phantom{0}^{\circ }$)	115.75	114.73	118.80	
$\zeta$ ($\phantom{0}^{\circ }$)	67.76	61.35	73.99	
Staggered pyrophosphates:	24	Eclipsed:	0	

**Fig. 8:**
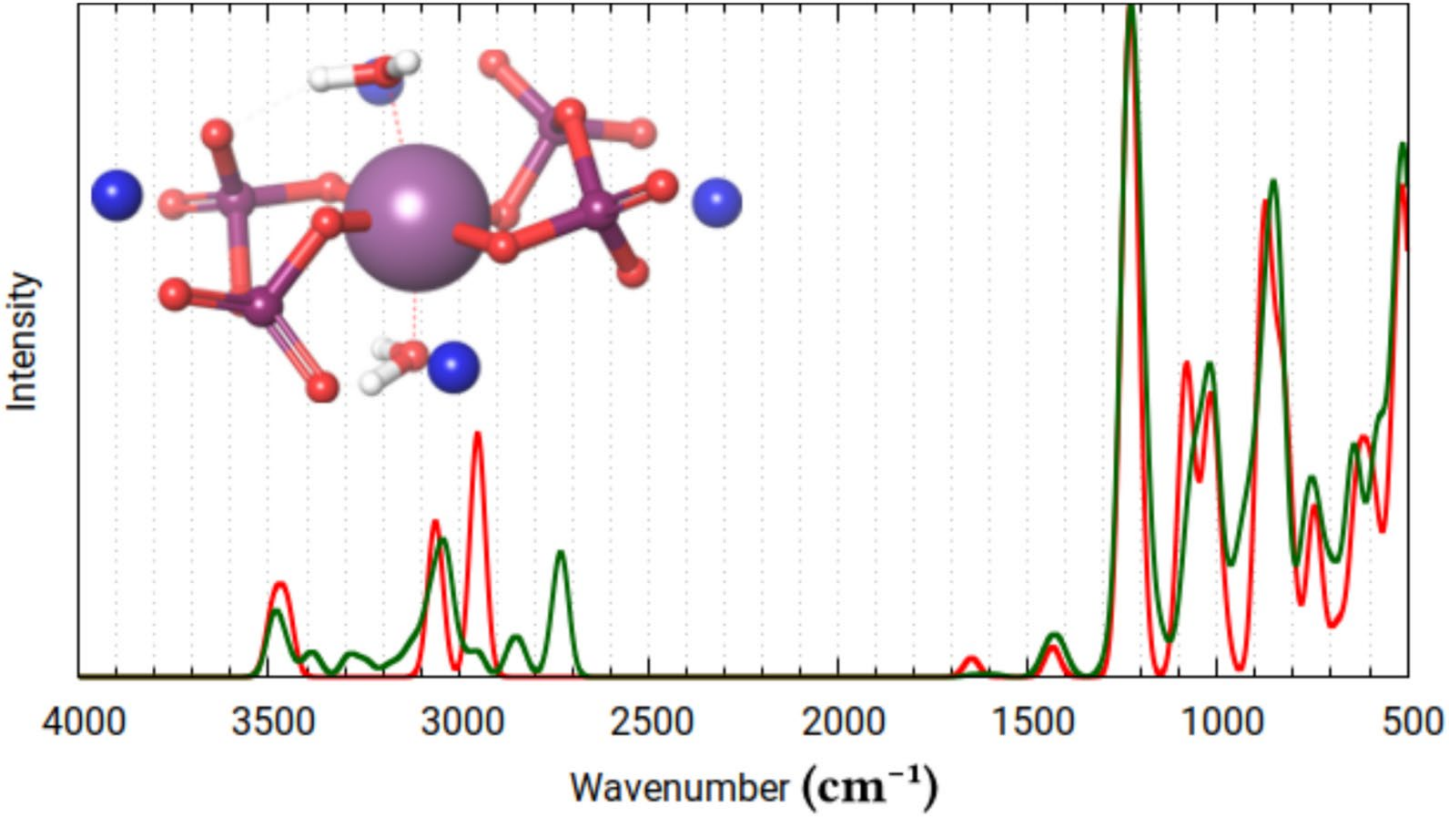
Calculated infrared spectra for the lowest energy conformer (red) and cumulatively all (12) conformers (green), of the neutralized form of Re-PYP.

In the interest of modeling the chemical environment within a buried protein binding site, a fully deprotonated species was also investigated, neutralized with four sodium ions (Fig. 8). The refined geometry and calculated vibrational spectrum correlated well (although not entirely) with those obtained using the doubly protonated complex neutralized with two sodium ions. The major differences are the shifting of the broad H-bridge ($\nu$(O-H,(M)H_2_O)) band to around 3000 cm${^{-1}}$, and the broadening of the mixed $\nu _s\mathrm{PO}_3$ and $\nu _{as}\mathrm{PO}_3$ peak near 1000 $\hbox {cm}^{-1}$.

*Additional isomeric ensembles with different protonation states are discussed in the Supplementary Information under subsection “Additional isomeric ensembles”.*

## Experimental investigations

### Synthesis

In practice, Tc-PYP is synthesized in situ, via the addition of 740 MBq (20 mCi) of pertechnetate to a solution of 12–40 mg pyrophosphoric acid and 0.4–4.9 mg tin(II)-chloride, where the pH of the reaction mixture is between 4.5–7.5 (see Additional Information).

Due to the extremely small amount of technetium present, accurate measurements cannot be taken of the product(s) of this reaction. In order to circumvent this, our synthesis used a molar ratio of 1:3:20 for Re:Sn:Pyrophosphate, which allows the full reduction of Re(VII) to Re(IV) and also provides ample pyrophosphoric acid for complexation, while producing an easily measurable amount of Re-PYP. This we called reaction Mixture P.

To confirm the oxidation state of rhenium in the products, and to investigate the contribution of the tin, potassium hexachlororhenate ($\hbox {K}_2$Re(IV)$\hbox {Cl}_{6}$) was also synthesized according to the method of Pavlova et al^26^, with which two additional samples were made where one contains the Sn(II) reducing agent (called Mixture H) and a third mixture that does not (called Mixture S). The potassium hexachlororhenate reagent was tested for purity with ATR-IR; no trace of the original perrhenate could be found.

**Fig. 9:**
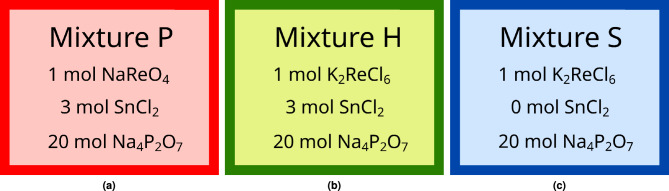
The three different reaction mixtures synthesized, with molar ratios.

**Fig. 10:**
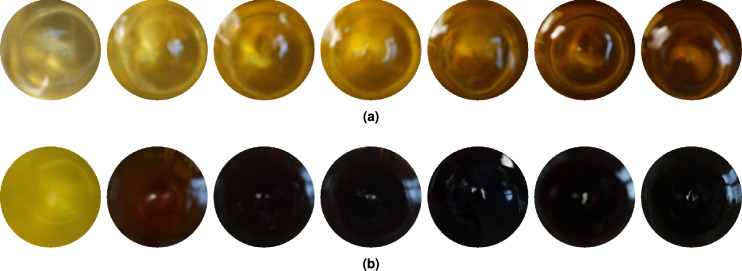
Colour evolution of Mixture P (**a**) and Mixture H (**b**), 5, 10, 15, 20, 25, 45, and 60 minutes after addition of the rhenium containing reagent.

Synthesis of the three mixtures (see Fig. 9) was conducted as outlined in the Methods section. Within seconds, after the addition of perrhenate, Mixture P and Mixture H turned a yellow-green color, which evolved into gold, brown, and finally black (see Fig. 10). These colours correspond to the increasing concentration of the product. Mixture H reacted much faster, with it reaching the colour intensity shown by Mixture P after an hour, in five minutes. Mixture S did not change for an hour, after which it suddenly reacted just as fast as Mixture H, turning brown-black. When the unreacted Mixture S was divided into two separate beakers, the sudden colour change happened ten minutes apart. In another synthesis, Mixture S reacted in under five minutes. Formation of identical products with different reaction rates in the presence and absence of Sn(II) suggests that tin has some indirect role in the formation rates of the intermediates. Because the protonation state of the complexing pyrophosphoric acid has a very large effect, through its changing denticity, small changes in pH due to the hydrolysis of potassium hexachlororhenate can also interfere with the reaction rate.

No precipitation, gas evolution, or noticeable temperature change was observed. The product mixtures remained at pH $\sim$6. Dilution of the product mixtures revealed the original yellow-green colour. Addition of acid causes the mixtures to shift to a more yellowish colour, while addition of a base shifts the colour to greenish; this is fully reversible (see the Supplementary Information, under “Pictures of the Reaction Mixtures”).

After two weeks, the mixtures become transparent, then a faded pink colour. This fading is due to the oxidative decomposition of the compound. Acidic mixtures retain their colour longer (see the Supplementary Information, under “Pictures of the Reaction Mixtures”), suggesting decreased stability in basic conditions, which is in line with our theoretical model (deprotonation of the aqua ligands destabilizes the complex, see Section 3). When frozen, the product mixtures did not turn transparent, however after three months they partially did turn dark pink.

Slow, dropwise addition of absolute ethanol to the reaction mixtures until 40 vol.% causes the precipitation of viscous black droplets forming a separate phase, which we assume to be a hydrated form of the product mixture, as it behaves similarly to the almost evaporated samples of the product mixtures when isolated. The supernatant becomes opaque and fades in colour, until the colored precipitate aggregates at the bottom of the vessel, at which point it becomes clear and colourless.

Crystallization was attempted several times under numerous conditions (see Supplementary Information section “Attempts at crystallization”), yet no crystalline phase of the product could be identified (no powder-XRD peaks were detected). Oxidative decomposition occurs in all cases where water is removed from the product mixtures, which is currently under further investigation. These decomposition products were successfully crystallized and identified with SCXRD to be alkali pyrophosphate salts and alkali perrhenates (crystal forms identified via single crystal XRD analysis isostructural with structures found in the Cambridge Structural Database^63^ ICSD 15389^64^ and CCDC 2001936^65^, respectively, see Supplementary Information section “Single crystal X-ray diffraction”). Despite this, the coloured viscous phase – which formed before the product mixtures dried completely – could be measured. ATR-IR, Raman, and Mössbauer spectra were taken of residues containing this coloured phase and are reported here, but as the product is undergoing decomposition, no quantitative analysis could be reasonably performed.

### UV-Vis spectroscopy

**Fig. 11:**
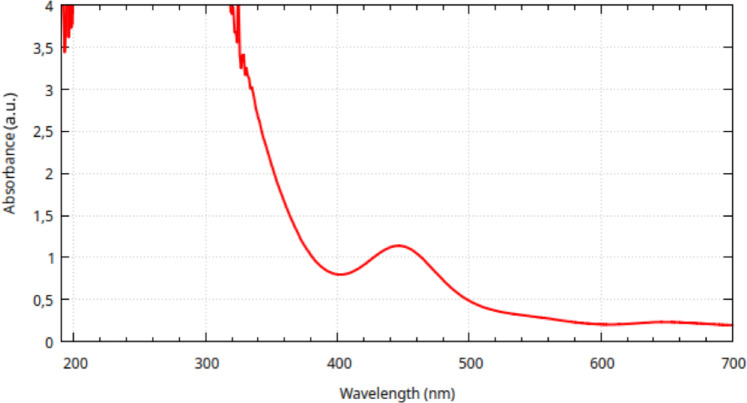
UV-Vis spectrum of Mixture P one hour from the start of the reaction. The pH was set to 7.40.

In order to investigate the physiologically active state of Tc-PYP, a freshly synthesized aqueous sample of reaction Mixture P was analysed with solution-phase UV-Vis spectroscopy after its acidity was adjusted with NaOH to match that of serum (pH=7.40) via a glass electrode pH meter. The UV-Vis spectrum (see Fig. 11) was taken one hour after the addition of the rhenium containing reagent.

Results indicate the presence of the Re(PYP)$\phantom{0}_2$($\hbox {H}_2$O)$\phantom{0}_2$ form (see Table 1). As predicted, a wider band appeared due to heterogeneous conformational ensemble and variability of the number of H-bridges. No other species appear to be present in large enough concentrations to be detected. No peaks were found in the case of the rhenium-free, tin(IV) chloride and pyrophosphoric acid containing blind.

### $\phantom{0}^{31}$P NMR spectroscopy

Changes in the Re-Sn-PYP system were studied by monitoring the chemical environments of PYP by $\phantom{0}^{31}$P NMR spectroscopy.

First, a reagent mixture containing only 240 mM pyrophosphoric acid (PYP) and 36 mM tin(II) chloride (Sn(II)$\hbox {Cl}_2$) with an initial pH of 5.93 was measured as a reference. Free PYP is a symmetric molecule with two magnetically equivalent phosphorus environments. At pH = 5.88 it is a mixture of two protonated species: $\hbox {H}_2\hbox {P}_2{{\hbox {O}_7}^{2-}}$ and $\hbox {HP}_2\hbox {O}_7^{3-}$; which due to the rapid $\hbox {H}^+$ exchange give one resonance signal at −7.87 ppm (Figure S11, S12).

**Fig. 12:**
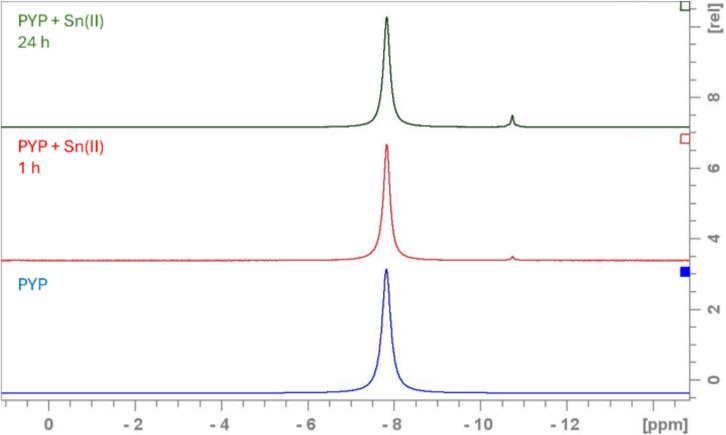
$\phantom{0}^{31}$P{$\phantom{0}^{1}$H} spectra at 400 MHz, 298 K and pH for the different systems, PYP (blue), PYP+Sn(II) 1 hour (red), PYP+Sn(II) 24 hours (green).

**Fig. 13:**
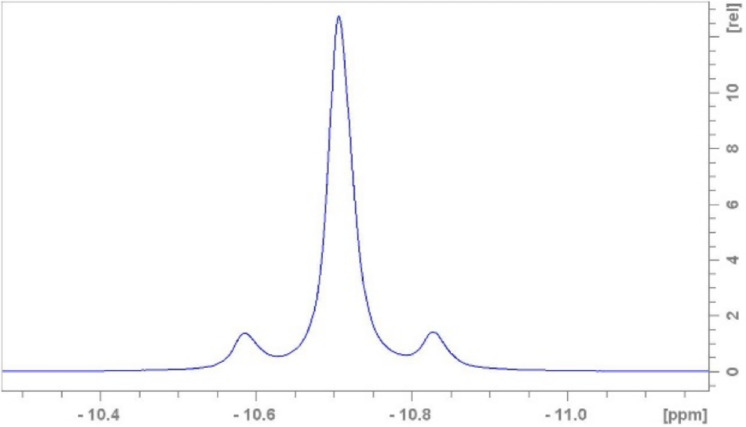
${}^{31}$P{${}^1$H} spectra at 400 MHz, 298 K and pH=5.88 zoomed for the resonances of the Sn-PYP complex.

Another signal, a multiplet at −10.75 ppm also appears in the spectrum, the intensity of which increases from 0.17% to 5.1% over 24 h (Fig. 12, top spectrum). The 1:10.5:1 ratio is due to the existence of different Sn isotopes. The NMR-active, spin 1/2 isotopes $\phantom{0}^{117}$Sn (natural abundance $\sim$7.7%) and $\phantom{0}^{119}$Sn (natural abundance $\sim$8.6%) give rise to doublets via coupling to the spin 1/2 $\phantom{0}^{31}$P, and both show similar coupling constants: $^2\hbox {J}_{\text {P}-\text {Sn}}$ = 39.2 Hz. The singlet peak in the middle originates from the $\phantom{0}^{31}$P atoms bound to the NMR-inactive Sn isotopes ($\phantom{0}^{112}$Sn, $\phantom{0}^{114}$Sn, $\phantom{0}^{116}$Sn, $\phantom{0}^{118}$Sn, $\phantom{0}^{120}$Sn, $\phantom{0}^{122}$Sn, $\phantom{0}^{124}$Sn) with a combined natural abundance of about 83.7%. (Fig. 13). This composition is highlighted in the multiplet ratio. Moreover, the existence of this multiplet is a clear indication, that a symmetric coordinative complex has been formed containing a 2-bond P-Sn motif. Note, in case of an asymmetric complex more $\phantom{0}^{31}$P resonances with corresponding coupling schemes, involving also $^2\hbox {J}_{PP}$ would be observable.

Based on the $\phantom{0}^{31}$P and $\phantom{0}^{119}$Sn NMR studies of Mathieu et al.^66,67^, who proposed a pyrophosphate-non-binding Sn(II) species and a pyrophosphate-binding symmetric Sn(IV) complex, this increase can be explained by the oxidation of Sn(II) to Sn(IV) by atmospheric oxygen, as no $\phantom{0}^{31}$P NMR resonances corresponding to a reduced Sn(II)-PYP complex were observed by us either, yet the initially low-intensity multiplet increases in conditions where oxidation to Sn(IV) is likely. This is also in line with the potentiometric studies of Duffield et al.^59^ for the tin–pyrophosphate system. Thus Sn(IV)-PYP was assigned to the multiplet at −10.75 ppm.

In our next experiment, the time evolution of the system modeling the diagnostic mixture (mixture P, see section 5.1), containing 12 mM $\hbox {NaReO}_4$, 36 mM $\hbox {SnCl}_2$, and 240 mM tetrasodium pyrophosphate at pH=5.88 was investigated.

Re(VII) is diamagnetic, however, the possibly formed Re(IV)(a $\hbox {d}^{3}$ metal species) is paramagnetic. If formed, the presence of Re(IV) will complicate spectral evaluation - especially if bound to PYP. The fast relaxation of the given coordination species – will make quantitative spectra evaluation challenging at best. Therefore, only qualitative analysis is definitive. Indeed, upon the introduction of Re(VII) to the Sn(II) and PYP containing system, the total signal intensity decreased significantly (by about $\sim$10%).

**Fig. 14:**
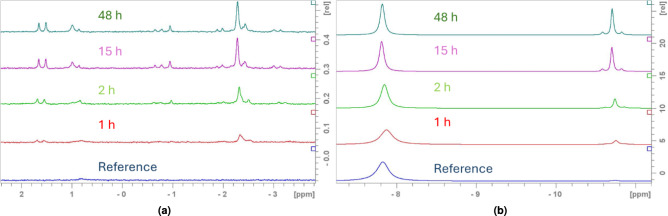
Time dependence of the two main $\phantom{0}^{31}$P{$\phantom{0}^1$H} spectral regions for sample P. Left panel shows the newly appearing peaks, while the right panel shows the characteristic PYP and Sn-PYP peaks. Change of integrals for these peaks throughout the reaction can be seen on Figure S14 in the Supplementary Information.

Besides the already detected peaks in the reference sample, no new peaks of comparable intensity were observed, however, several new low-intensity resonances appear in the 2 – (−7) ppm range (Fig. 14). Several of them appear as doublets, suggesting the presence of asymmetric PYP environments where, where the two-bond P-P couplings vary between ${^2}\hbox {J}_{PP}$ = 20.0–23.0 Hz. Based on integral values, we can assume, that the doublets at 1.58 and −7.03 belong to the same PYP fragment. Singlet resonance at −2.28 ppm belongs to a symmetric environment, or to free (ortho)phosphate. Due to low intensities, we cannot determine whether any of the new signals belonging to metal-complexed $\phantom{0}^{31}$P species are solely Re or mixed Re/Sn containing complexes.

However, it is apparent that due to the presence of the Re, the amount of Sn(IV)$\hbox {PYP}_2$ is increased significantly already after 1 h – surpassing the amount detected in the reference sample without Re(VII) after 24h. This indicates that oxidation of Sn(II) is facilitated by Re(VII), or conversely, that Re(VII) is reduced by Sn(II). It is possible that the low-intensity peaks belong to diamagnetic rhenium-containing species (such as Re(V) or Re(VII)), which can arise through the oxidative decomposition of Re(IV)$\hbox {PYP}_2$. We can not detect (due to small amounts) the Sn satellites, but we can not exclude formation of mixed Re(Sn)-PYP complexes. Following the fast increase (over $\sim$ 1 h) increase, Sn(IV)$\hbox {PYP}_2$ content continued to increase slowly until reaching saturation point around 30h. This slower oxidation phase we attribute to atmospheric oxygen in this case also.

**Fig. 15:**
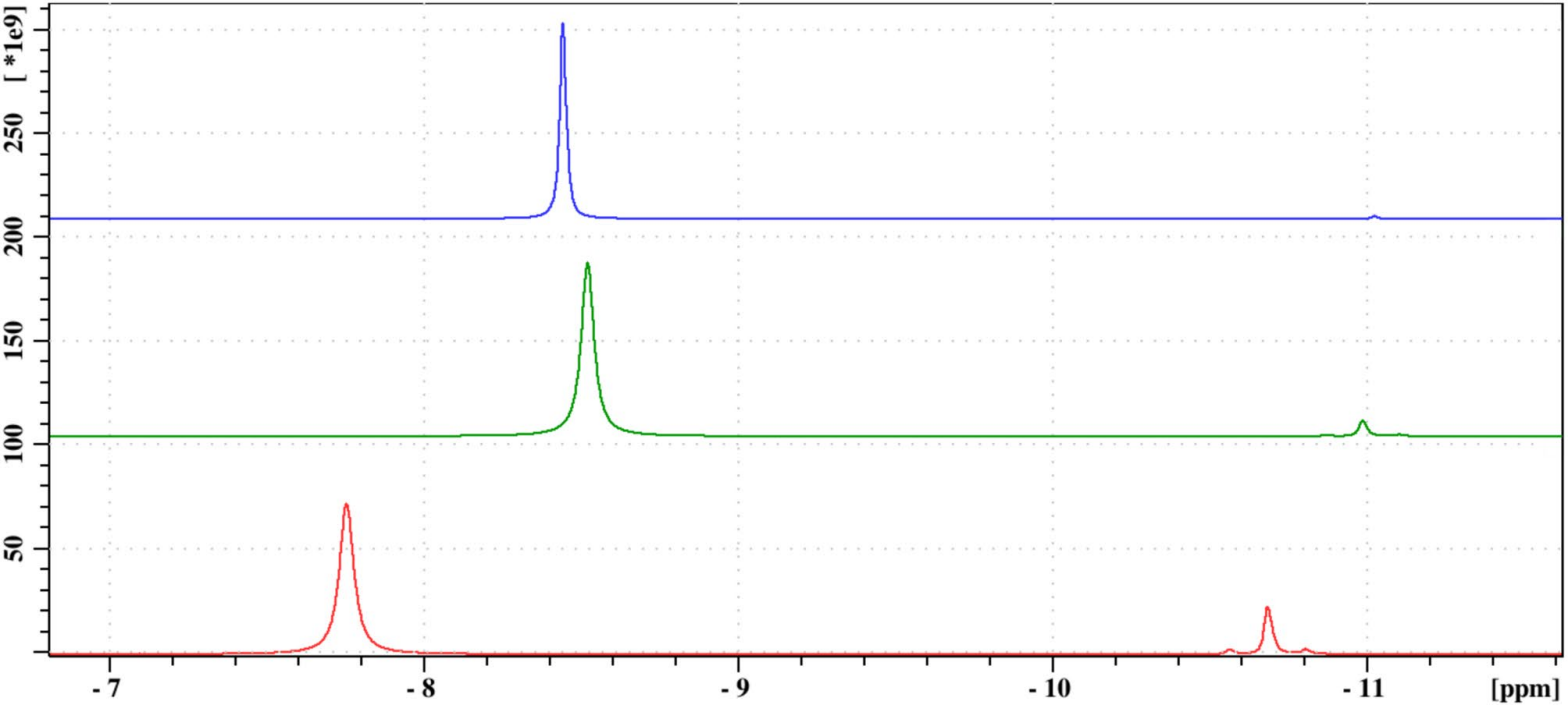
Stacked $\phantom{0}^{31}$P{$\phantom{0}^{1}$H} spectral regions for P (red), H (green), S (blue) reaction mixtures one hour after reaction start. Changes in chemical shifts are due to pH differences and Sn(IV) concentration.

Next, the effects of different oxidation conditions were monitored. In a dedicated experiment, mixtures P (pH 5.88), H (pH 6.05), and S (pH 6.25) were analyzed one hour after the reaction start (Fig. 15). In sample P the redox reaction takes place between Re(VII) and Sn(II), and a significant amount (17%) of Sn(IV)$\hbox {PYP}_2$ is detected, even in the presence of paramagnetic Re(IV). On the other hand, in mixture H this oxido-redox reaction does not take place, and the amount of $\hbox {SnPYP}_2$ is much smaller, and can be attributed to air oxidation. While in Mixture S no, or negligible Sn(IV) is present as an impurity from the synthesis of $\hbox {K}_2\hbox {ReCl}_6$. These findings also support the notion that the presence of Re(VII) enhances Sn(IV)$\hbox {PYP}_2$ formation, but also no further reduction takes place after the formation of Re(IV).

The small peaks appearing in the 2-(−7) ppm region also show different patters in the three different samples (Figure S13). The amount of small peaks is accentuated for sample P, it is less for sample H and they are also present in sample S. The increase in number of peaks of samples P and H might suggest that the formation of mixed Re-Sn diamagnetic complexes is possible as minor components. However, confirming this notion would need further investigation.

Taken together, our observation support the following redox reaction taking place in mixture P:

3 $$\begin{aligned} 2 \text {Re(VII)} + 3 \text {Sn(II)} \xrightarrow {\text {H}_2\text {O}} 2 \text {Re(IV)} + 3 \text {Sn(IV)} \end{aligned}$$

Although exact quantitative analysis of the sample containing mixture P is not possible due to the presence of the paramagnetic Re(IV) atom, it is tempting to, at least approximate, the stoichiometry of the emerging species. Supposing that the paramagnetic effect can be localized to the immediate surroundings of Re(IV) (the $\phantom{0}^{31}$P atoms of Re-binding PYP), an idealized reading of the observed spectra would allow the attribution of the approximately 10% total signal decrease solely to the presence of NMR-silent Re-PYP species. To test the applicability of this hypothesis we carried out a “calibration” measurement series and found that increasing the Re(IV) content of Re(IV)-PYP systems results in a near linear loss of total signal intensity. Three samples containing 120 nM PYP, 18 nM $\hbox {SnCl}_2$, and an increasing amount of $\hbox {K}_2\hbox {ReCl}_6$ were analyzed (Figure S15). The first mixture (pH 5.71) contained 6 mM of rhenium (1:20 Re:PYP), which caused a signal decrease of 11.3%. The second mixture (pH 5.10) contained 18 mM of rhenium (3:20 Re:PYP) and caused a signal decrease of 30.5%. The third mixture (pH 3.58) contained 30 mM of rhenium (5:20 Re:PYP) and caused a signal decrease of 51.1% – in line with their increasing Re(IV) content.

**Fig. 16:**
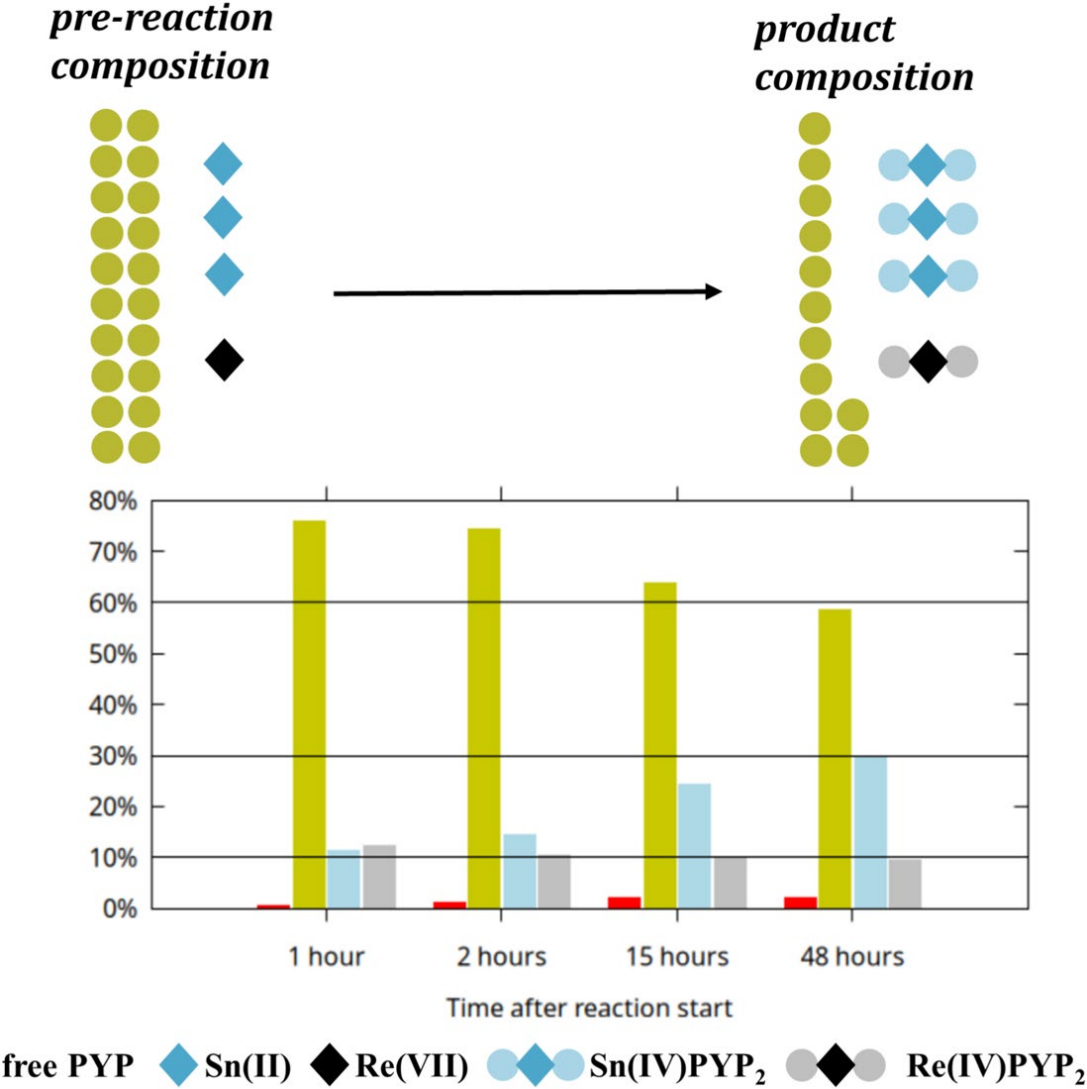
Bottom: column charts illustrating the change of component integral (concentration) ratios over time, showing also the decrease of the total signal due to the formation of paramagnetic phosphorus-containing species (see also S14). Minor components are shown in red, P of free PYP in yellow, P in Sn(IV)$\hbox {PYP}_2$ in blue, signal loss due to phosphorus taking part in a paramagnetic rhenium complex is represented in gray. Black lines show predicted final concentrations based on theoretical stoichiometric compositions. Top: schematic representation of the reaction: stoichiometry of Sn(IV)$\hbox {PYP}_2$ and Re(IV)$\hbox {PYP}_2$ formation after 48 h (12:6:2 of PYP(free):$\hbox {SnPYP}_2$:$\hbox {RePYP}_2$ corresponding to the measured 60%:30%:10% product distribution, respectively).

If we accept that the $\sim$10% intensity loss measured in case of mixture P can be attributed to the presence of approximately the same amount of Re(IV) in the system, the final integral ratios of approximately 60% PYP : 30% Sn(IV) : 10% Re (IV) (or 12: 6: 2 – see Fig. 16) would suggest the stoichiometric formation of Sn(IV)$\hbox {PYP}_2$ and Re(IV)$\hbox {PYP}_2$ species from the pre-reaction mixture of 20 PYP : 3 Sn(II) : 1 Re(VII).

The equation for the complexation reaction is as follows:

4 $$\begin{aligned} \text {Re(IV) intermediate species} + 2 [\text {H}_2\text {P}_2\text {O}_7]^{2-} \xrightarrow {\text {H}_2\text {O}} \text {Re}(\text {H}_2\text {P}_2\text {O}_7)_2(\text {H}_2\text {O})_2 \end{aligned}$$

This stoichiometry agrees with the results reported by Kroesbergen et al.^24,25^ for the technetium pyrophosphate complex using gel chromatography, as well as with the NMR studies by Mathieu et al.^66,67^. Since these molar ratios account for the entirety of the Sn-content, these considerations would also suggest that no (or very little amount of) mixed Tc/Sn-pyrophosphate species are created in the synthesis of the diagnostic agent. While this is in agreement with the theoretical model, the paramagnetic relaxation still makes quantitative analysis unreliable, and so these results should be taken with caution.

*MS-ESI-HILIC analysis of the solution-phase product mixture is available in the Supplementary Information under section “Mass spectrometry and HILIC”.*

### ATR-IR and Raman spectroscopy of the amorphous hydrate

Due to the inherent flexibility of the complex, all measured spectra should be seen as the spectra of conformational ensembles, further complicated by the continuous decomposition of the rhenium-complex and its inability to crystallize. This decomposition results in the intensity growth of the $\nu _{as}$(P-O-P) band in the spectra (due to the appearance of perrhenates), as well as the decrease of Re-PYP bands.

#### Vibrational spectra of the reaction mixtures and deuterated variants

**Fig. 17:**
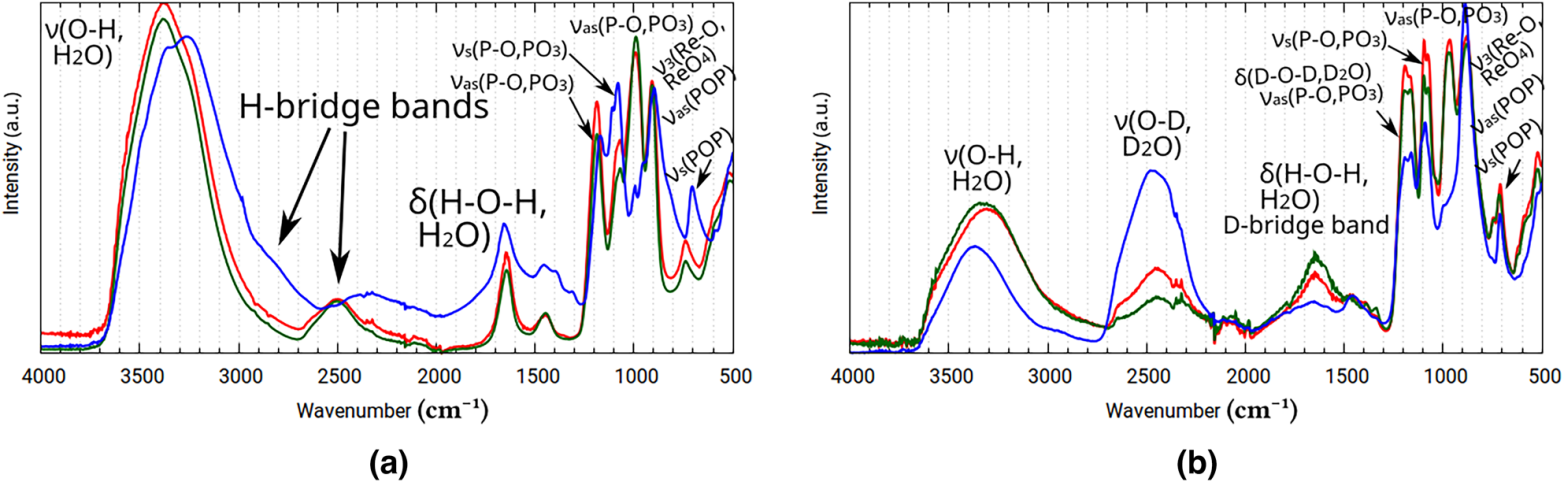
Experimental ATR-IR spectra: **(a)** Mixtures P (red), H (green), and S (blue), **(b)** Deuterated variants of Mixtures P (red), H (green), and S (blue).

**Fig. 18:**
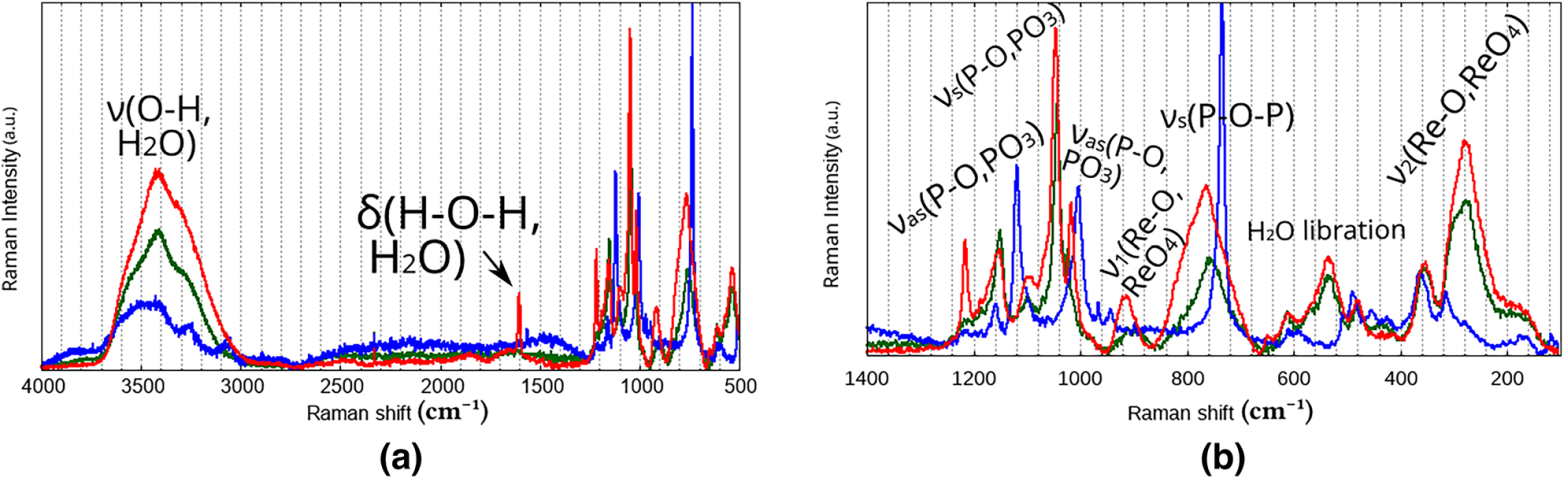
Experimental Raman spectra of Mixtures P (red), H (green), and S (blue). **(a)** 4000-500 $\hbox {cm}^{-1}$ **(b)** Same spectra, 1400-100$\hbox {cm}^{-1}$.

The experimentally determined spectra contain bands belonging to Re(IV) pyrophosphate itself, but also Sn(IV) pyrophosphate (except in the case of Mixture S), and a large excess of pyrophosphate alkaline metal salts (each having at least 21 normal modes^60^), as well as two distinct chemical environments for water molecules (water of crystallization and aqua ligands coordinated to the metals). Perrhenates also contribute 3 vibrational bands^60^. as decomposition products, although the amount of perrhenate can vary. This diversity of chemical species complicates the identification of vibrational bands greatly. Nevertheless the recorded spectra do contain information about the chemical environments of water in these systems.

Both in IR and Raman (see Fig. 18) spectra, the strong intramolecular H-bridges predicted by the theoretical investigations were visible in a broad band around 2500 $\hbox {cm}^{-1}$. The location of this characteristic band lies within the range predicted via the XTB isomeric search, with some variation due to the water content of the measured hydrate samples, and to the pH, affecting the isomer-ensemble. The spectrum of Mixture S (see Fig. 17a) features a noticeable upfield shift, displaying a shoulder at 3000 $\hbox {cm}^{-1}$, hinting at stronger rhenium-aqua interaction in the absence of tin, likely due to the formation of an alternative conformational composition.

These tight hydrogen-bridge systems were investigated via deuteration: the spectra then collected display a shift of the H-bridge band upfield to around 1700 $\hbox {cm}^{-1}$ (see Fig. 17b), which is in line with the calculated value of 1750 $\hbox {cm}^{-1}$, although finding the exact center of the peak is difficult, as it coincides with the bending vibrations of the deuterated, semi-deuterated, undeuterated, and uncoordinated water molecules. Furthermore, the undeuterated and deuterated spectra also differ in their degree of decomposition, and vary in conformational makeup as discussed above in Section 4.3. Because of these complications, no exact H/D ratio could be calculated for the aqua ligands of the complex. The water of hydration however has a H/D ratio of 1.36±0.007 for the three Mixtures, showing no change in chemical environment.

#### Vibrational spectra of the stoichiometric reaction mixtures

Stoichiometric reaction mixtures were prepared with molar ratios of 1 $\hbox {NaReO}_4$ : 1.5 $\hbox {SnCl}_2$ : 5 $\hbox {Na}_4\hbox {P}_2\hbox {O}_7$ for Stoichiometric Mixture P, 1 $\hbox {K}_2\hbox {ReCl}_6$ : 1.5 $\hbox {SnCl}_2$ : 5 $\hbox {Na}_4\hbox {P}_2\hbox {O}_7$ for Stoichiometric Mixture H, and 1 $\hbox {NaReO}_4$ : 0 $\hbox {SnCl}_2$ : 2 $\hbox {Na}_4\hbox {P}_2\hbox {O}_7$ for Stoichiometric Mixture S. These mixtures were synthesized the same way the as before, except for the changed molar ratios. A fourth, reference mixture was also synthesized, with a molar ratio of 0 $\hbox {NaReO}_4$ : 1 $\hbox {SnCl}_2$ : 2 $\hbox {Na}_4\hbox {P}_2\hbox {O}_7$.

**Fig. 19:**
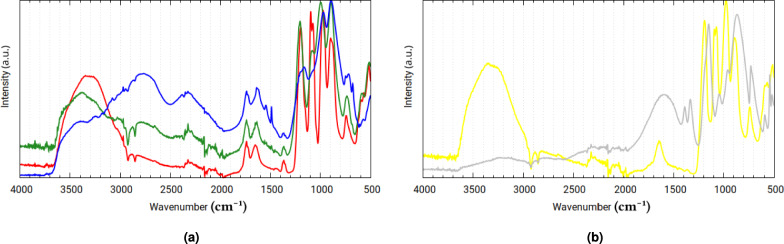
Experimental ATR-IR spectra of **(a)** Stoichiometric Mixtures P (red), H (green), and S (blue), **(b)** the reference Sn(PYP)$\phantom{0}_2$ (yellow), and $\hbox {Na}_2\hbox {H}_2\hbox {P}_2\hbox {O}_7$ (gray).

**Fig. 20:**
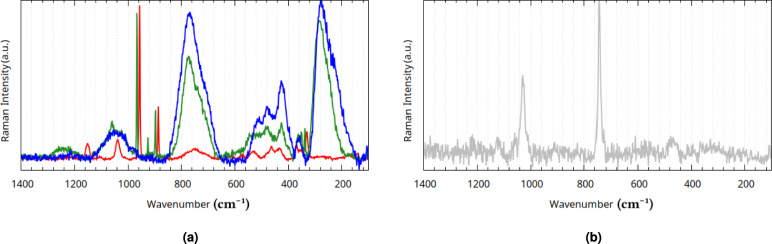
Experimental Raman spectra of **(a)** Stoichiometric Mixtures P (red), H (green), and S (blue), and **(b)** $\hbox {Na}_2\hbox {H}_2\hbox {P}_2\hbox {O}_7$ (gray).

After two days of standing on air, 1.5 ml of each were placed in a desiccator for two more days. The resultant dark-green (for Stoichiometric Mixtures P, H, S) and white (for the reference mixture) residues were measured via Raman (see Fig. 20) and ATR-IR (see Fig. 19) spectroscopy no more than four hours apart. Raman spectra of the reference mixture could no be taken due to fluorescence.

**Table 5:** Band assignments of Stoichiometric Mixtures P, H, S. s = strong, m = medium, w = weak, v = very (weak/strong), b = broad.

Infrared bands (1/cm)			Raman bands (1/cm)			Assigned vibration
P	H	S	P	H	S	
3597wb	3597wb	3540wb				
3304mb	3384mb	3384wb				
	3253mb	3253wb				$\nu{H}_2{O}$
3026wb	3026wb	3076mb				$$\nu({M}){H}_2{O}$$$$\nu({P}){OH}$$
2891wb	2878wb	2878mb				$\nu ({P}){OH}$
2808wb	2808wb	2766mb				
2326vwb	2326wb	2326mb				
1741wb	1741wb	1741wb				$\nu _{as}$(P-O-P) combination band
1719wb	1719wb	1719wb				$\nu _3\hbox {ReO}_4$ combination band
1654wb	1642wb	1632wb				$\delta \hbox {H}_2{O}$
		1543wb				$\delta ({M}){H}_2{O}$
		1487w				
1374vwb	1374vwb	1374vwb				$\delta (\mathrm{P-O-H})$
1200s	1208s	1232m		1243wb		
		1207m				$\nu _{as} ({PO}_3)$
		1199m				
1151mb		1165m	1152m			
1096s	1090m	1085mb				
1074s			1040m	1058mb	1045mb	$\nu _{as} ({PO}_3)$
976s	1012s	971s	956s	967s		$\nu _{as}{PO}_3$ and $\nu _{1}{ReO}_4$
						$\hbox {H}_2$O libration (rocking)
			923w	926m		
896sb	896sb	896sb	887m	897m		$\nu _{3}{ReO}_4$
874sb	874sb	874sb				$\nu _{as}$(P-O-P)
745wb	739wb	749wb	748wb	772sb	771sb	
		720wb				$\nu _s$(P-O-P)
	683vwb	679w				
625vwb	627vwb	630vwb	614wb			
		608vwb				
596wb	586wb		573wb			
566wb		572wb				
518mb	511mb	471mb	530mb	534mb	512mb	$\hbox {H}_2$O libration (wagging)
			463mb	479mb	479mb	
			433mb	424mb	424mb	Translational bands
			370mb	361mb	361mb	
			346mb	350m		$\nu _{2} {ReO}_4$
			330s	337m		
				285sb	276sb	
			173wb			
			141wb			

The vibrational spectra of the mixtures feature distinct bands when compared to the spectrum of disodium dihydrogen pyrophosphate: the $\nu _{as}\mathrm{PO}_3$ bands shifted downfield from 1153 $\hbox {cm}^{-1}$ and 964 $\hbox {cm}^{-1}$ to 1232-1200 $\hbox {cm}^{-1}$ and 1012-971 $\hbox {cm}^{-1}$, with the corresponding symmetric band $\nu _{s}\mathrm{PO}_3$ shifting from 1048 $\hbox {cm}^{-1}$ to 1096-1074 $\hbox {cm}^{-1}$. The $\nu _{as}$(P-O-P) band shifted downfield from 868 $\hbox {cm}^{-1}$ to 896-874 $\hbox {cm}^{-1}$, and the $\nu _{s}$(P-O-P) shifted from 731 $\hbox {cm}^{-1}$ to 749-679 $\hbox {cm}^{-1}$.

Although the decomposition and isomeric diversity causes uncertainty in the location and intensity of the measured bands, as indicated by the isomer search, three characteristic absorptions with relatively sharp peaks are always observed near the $\nu _{as}\hbox {PO}_3$and $\nu _{as}$(P-O-P) asymmetric vibration bands (at around 1200, 1000, and 900 $\hbox {cm}^{-1}$).

Mixture P and H gave practically identical spectra, as expected, but surprisingly so did the Reference Mixture. On the other hand, Mixture S gave dissimilar spectra featuring broad bands around 2766 $\hbox {cm}^{-1}$ corresponding to a strong hydrogen-bridge system as seen before. The presence of Sn-PYP modifies the conformational ensemble of Re-PYP greatly, even though no mixed rhenium-tin major species could be identified in solution (see Section 5.3).

As previously discussed, the theoretical structures of tin(IV)- and rhenium/technetium(IV) diaqua dipyrophoshate are near identical; this isostructurality can allow the formation of co-crystals: microcrystals of Sn-PYP may be able to act as nucleation sites for Re-PYP, leading to the formation of Re-PYP microcrystals with similar complex geometry.

The Raman spectrum of Mixture S resembles that of $$\hbox {K}_{2}$$Mn(II)($$\hbox {H}_{2}\hbox {P}_{2}\hbox {O}_{7}$$)$$\cdot$$2$$\cdot$$$$\hbox {H}_{2}$$O^55^, lending credence to the notion that a structurally similar structure forms despite the difference of the central cation’s oxidation state. The largest divergences from the spectrum of $$\hbox {K}_{2}$$Mn(II)($$\hbox {H}_{2}\hbox {P}_{2}\hbox {O}_{7}$$)$$\cdot$$2$$\hbox {H}_{2}$$O measured by Essehli et al. are the $$\nu _2\hbox{ReO}_4$$ and $$\nu _3\hbox {ReO}_4$$ bands due to the presence of the decomposition product. Assignations of distinguishable vibration bands are available in Table 5.

**Fig. 21:**
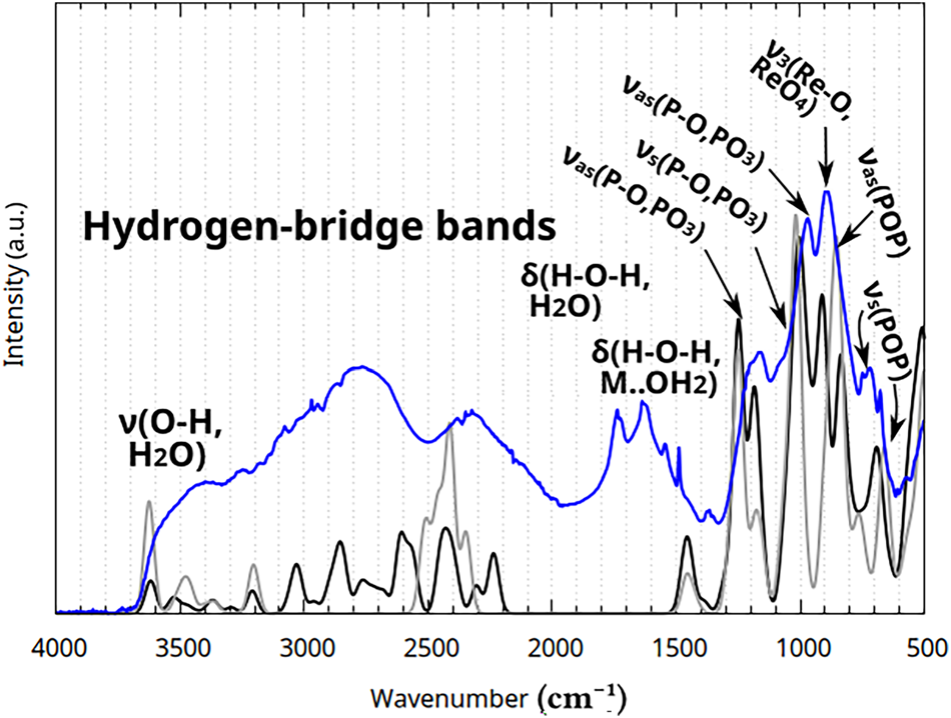
Comparison of Mixture S’ (blue) and the asymmetric (black) and symmetrized (gray) physiological ensembles’ spectra from the XTB conformer search.

The theoretical spectra calculated for the physiological forms’ conformer ensembles (the semi-protonated asymmetric and symmetric Re/Tc diaqua dipyrophoshate structures shown in Figs. 6 and 7) align well with the experimental spectrum of Mixture S (Fig. 21). The only visible bands not described by this molecular model are those of uncoordinated water, perrhenates (at near 900 $\hbox {cm}^{-1}$), pyrophosphoric acid salts, the latter of which causes noticeable splitting to appear at 1153 and 712 $\hbox {cm}^{-1}$.

The effects of broadening of certain bands due to the highly flexible nature of the system are evident when we compare the theoretical spectra for the asymmetric (more conformers) and symmetrized (less conformers) forms: Fig. 21 reveals the discrepancy concerning the broadening of the hydrogen-bridge band that was not visible with a one-conformer DFT analysis (Fig. 5); because the coordinated waters’ bending band is quite narrow in comparison, the stretching and bending vibrations of the aqua ligands produce bands of the same intensity maxima. The relative intensities of the three characteristic vibrational bands are not only highly variable because of the rhenium diaqua dipyrophosphate’s sensitivity to slight changes in its chemical environment, but also because any breakage in symmetry during crystallization can lead to different amounts of splitting and broadening in them.

*Sn-Mössbauer analysis of the solid-phase product mixture is available in the Supplementary Information under section “Mössbauer spectroscopy”.*

## Investigating the possibility of an ATTR binding site

**Fig. 22:**
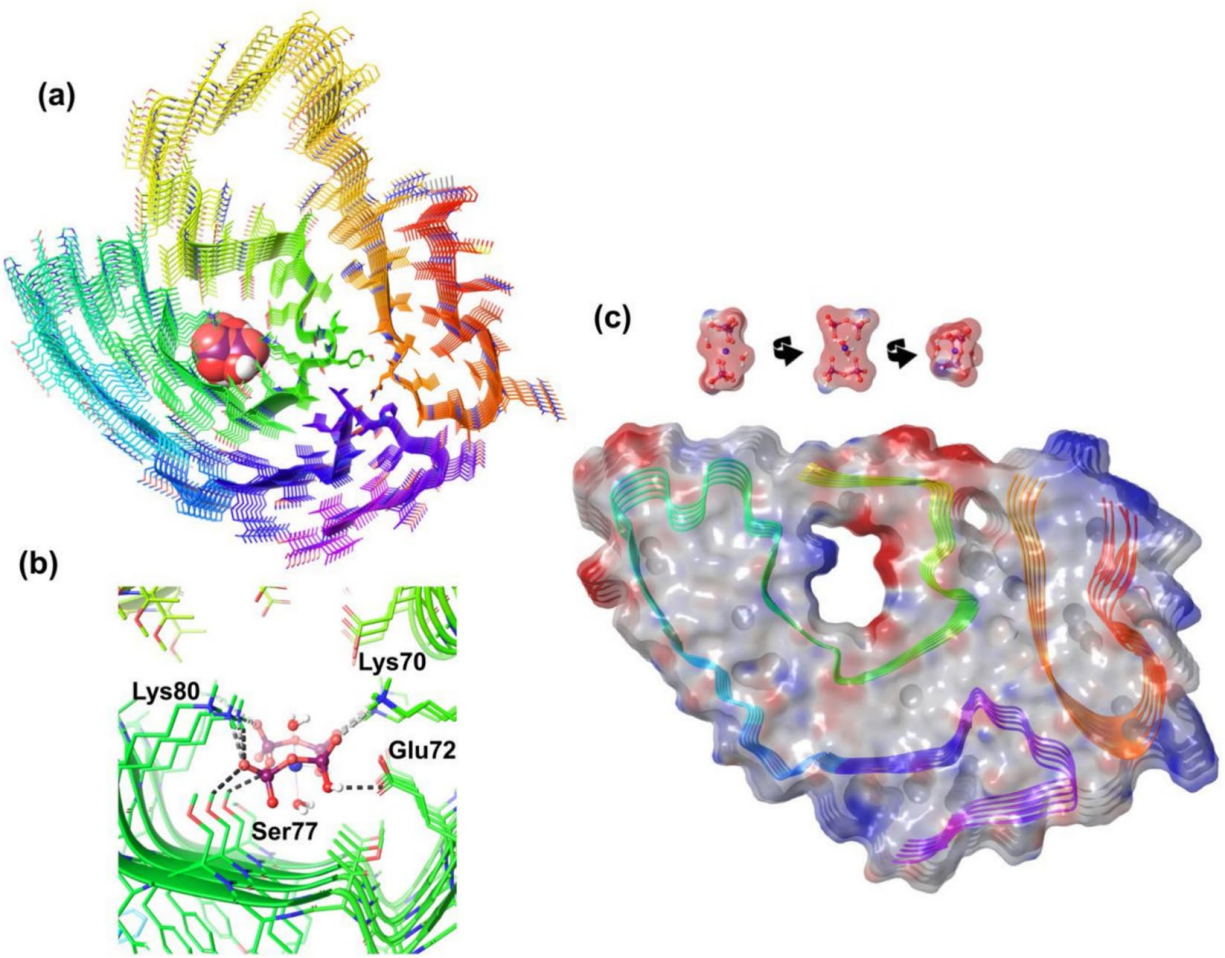
**(a)** Model of the Tc-PYP – amyloid fibril complex derived using the MCMM search method. The fibril is shown from the top (left) and the side (right), its backbone colored by the progression of the sequence from red to deep blue (N-terminus to C-terminus). Atoms of the diaqua-Tc-PYP species is shown as balls, while the protein atoms as sticks (non-polar hydrogens were omitted for clarity). **(b)** H-bond (dark gray) and salt-bridge (light gray) formed between the tracer and the protein matrix. **(c)** Molecular surface (shown on the same scale) and electrostatic pattern of the interacting partners. Spacious inner channels are not present in all amyloid fibrils, typically closely packed sidechains that even exclude water molecules (dry zippers) form the core of fibril structures. For a tableau of topologies see Figures S24-S25.

Monte Carlo multiple minimum (MCMM) conformational search was carried out to establish whether the size and charge distribution of the proposed Tc-PYP species is compatible with those of the interior of amyloid fibrils. The cryo-electronmicroscopic (cryo-EM) structure of a transthyretin amyloid fibril (wild-type) originating from a senile ATTR patient (retrieved from the Protein Data Bank: 8ade) was used as the target, expanded with its disordered loop segment not detectable with cryo-EM. The results demonstrate that diaqua-, doubly protonated Tc-PYP fits into the spacious central channel of the amyloid, where it typically forms 4 H-bonds and 6 salt bridges with the polar and charged sidechains of Lys70, Glu72, Ser77 and Lys80 (see Fig. 22). More detailed studies will be required to determine its precise binding conformation; the present results should be viewed as a simple confirmation that such a complex can indeed form.

## Conclusions

The isomeric diversity of Re-PYP and Tc-PYP makes the determination of a single exact geometry difficult (resulting in the partial determinations of previous authors) and less useful in practice. Based on our results we propose that the physiologically active form is mostly a mixture of differently protonated diaqua dipyrophosphate complexes (Re/Tc($\hbox {P}_2\hbox {O}_7$H)$\phantom{0}_2$($\hbox {H}_2$O)$\phantom{0}_2$ $\phantom{0}^{2-}$), with a possible minority of monoaqua and triaqua forms. The coordination space of the Re/Tc metal atom is similar to its heteroleptic aqua complex. This isomeric flexibility could be the basis of its ability to directly and selectively coordinate to certain disease causing amyloid fibrils in physiological settings. We further confirmed, using a molecular modeling method, that its size and polarity are both compatible with the inner channel of wild type transthyretin amyloid fibrils.

No crystalline phase of the tracer’s rhenium analogue could be analyzed, due to the oxidative decomposition observed in low water content environments. The amorphous hydrate of this complex is stable when dry but highly hygroscopic. The complex is resistant to decomposition in acidic solutions, alkaline solutions cause increased decomposition most probably due to the deprotonation of the aqua ligands.

As for oxidation state of Re (and by analogy, Tc) and the role of tin, we confirmed the IV oxidation state indirectly via solution NMR and UV-Vis spectroscopy, and showed some evidence that tin does not form a common species with rhenium (and by extension technetium) in solution, meaning that it has no effect on the binding mechanism of the tracer. Possible intermediers of the reaction were found, but the exact mechanism was not elucidated; still, using hexachlororhenate instead of perrhenate was found to greatly hasten the production of Re-PYP.

We conclude that the topology of Re- and Tc-PYP radiotracers is compatible with direct association with the protein component of disease causing deposits - a possiblity that has not been, as of yet, explored. We believe that the molecular models proposed here for these radiotracers can be used in further modeling studies aimed at determining the structural and electrostatic requirements of this association to certain physiological protein partners. This would greatly aid the interpretation of diagnostic results in the field of amyloidosis illnesses that have become an ever-increasing threat due to the increasing life expectancies of our societies, and pave the way for the development of more effective and selective diagnostic and therapeutic agents.

### A list of common $\phantom{0}^{99m}$Tc-PYP diagnostic kits

**FDA (Mallinckrodt):** https://www.accessdata.fda.gov/drugsatfda_docs/label/2017/017538s019lbl.pdf**IAEA:** https://www-pub.iaea.org/mtcd/publications/pdf/trs466_web.pdf** CANM (Calgary Radiopharmaceutical Centre):** https://canm-acmn.ca/resources/Documents/Email%20Blast%20Documents/PYP%20Package%20Insert%202018.pdf

## Supplementary Information

Supplementary Information.

## Data Availability

The data used to support the findings of this study are available in this article or the attached supplementary.

## Code availability

CCDC 2473193 and CCDC 2473194 contain the supplementary crystallographic data for this paper. These data can be obtained free of charge via www.ccdc.cam.ac.uk/data_request/cif, or by emailing data_request@ccdc.cam.ac.uk, or by contacting The Cambridge Crystallographic Data Centre, 12 Union Road, Cambridge CB2 1EZ, UK; fax: +44 1223 336033.
