# Supplementary material for: Physiologically relevant forms of Tc- and Re-pyrophosphate radioactive tracers and the basis of their transthyretin amyloid sensitivity
Source: Sci Rep. 2026 Jan 24;16:6111. doi: 10.1038/s41598-026-35746-5 (PMC12901035; doi:10.1038/s41598-026-35746-5)
Supplement: Supplementary file 1 — Supplementary Information. [file 41598_2026_35746_MOESM1_ESM.pdf]

Supplementary Information for "Physiologically relevant forms of Tc- and Re-pyrophosphate radioactive tracers and the basis of their transthyretin amyloid sensitivity"

Kevin Zsolt Simon[1,2], Kende Attila Béres[4,5], Attila Farkas[6], Nándor Papp[7,2], Andrea Bodor[7], Veronika Harmat[1], Dávid Papp[8,2], Maria Gracheva[9], Máté Sulyok-Eiler[3], András Perczel[1,3], László Kótai[4,*], and Dóra Karancsiné Menyhárd[1,3,*]

[1] *Laboratory of Structural Chemistry and Biology, Institute of Chemistry, ELTE Eötvös Loránd University, Budapest, Hungary*
[2] *ELTE Hevesy György PhD School of Chemistry, ELTE Eötvös Loránd University, Budapest, Hungary*
[3] *HUN-REN-ELTE Protein Modeling Research Group, ELTE Eötvös Loránd University, Budapest, Hungary*
[4] *Institute of Materials and Environmental Chemistry, HUN-REN Research Centre for Natural Sciences, H-1117 Budapest, Hungary*
[5] *Institute of Chemistry, ELTE Eötvös Loránd University, 1117-Budapest, Pázmány P. s. 1/A, Hungary*
[6] *Department of Organic Chemistry and Technology, Faculty of Chemical Technology and Biotechnology, Budapest University of Technology and Economics, Műegyetem rkp. 3., H-1111 Budapest, Hungary*
[7] *ELTE, Eötvös Loránd University, Budapest, Hungary, Institute of Chemistry, Analytical and BioNMR Laboratory*
[8] *MTA-ELTE Lendület Ion Mobility Mass Spectrometry Research Group, Faculty of Science, Institute of Chemistry, Eötvös Loránd University, Budapest, H-1117, Hungary*
[9] *HUN-REN Centre for Energy Research, Konkoly-Thege Miklós út 29-33, 1121 Budapest, Hungary*
[*] *dora.k.menyhard@ttk.elte.hu, kotai.laszlo@ttk.hu*

December 18, 2025

Contents

1 Additional theoretical results **2**
1.1 Charge distribution . . . 2
1.2 Other possible Tc-PYP reaction products and intermediates . . . 3
1.3 The isostructural nature of Tin(IV)-PYP . . . 3

2 Additional isomeric ensembles **5**

3 Attempts at crystallization **14**

4 NMR **15**

5 Mass spectrometry and HILIC **17**

6 Mössbauer spectroscopy **19**

7 Pictures of the Reaction Mixtures **20**

8 Single crystal crystallographic studies (SCXRD) **21**

9 A tableu of amyloid structures **25**

10 Theoretical structures **29**
10.1 Structures from DFT calculations . . . 29
10.1.1 Figure 3, Article 3 . . . 29
10.1.2 Figure 4, Article 3.1 . . . 29
10.1.3 Figure 1, SI 1.2 . . . 32
10.1.4 Figure 5, Article 3.2 . . . 33
10.1.5 Figure 2, SI 1.3 . . . 34
10.1.6 Figure 3, SI 1.3 . . . 34

10.2 Structure ensembles from conformational search . . . 35
10.2.1 Table 2, Article 4.3 . . . 35
10.2.2 Table 3, Article 4.3 . . . 47
10.2.3 Table 4, Article 4.3 . . . 51
10.2.4 Table 2, SI 2 . . . 64
10.2.5 Table 3, SI 2 . . . 73
10.2.6 Table 4, SI 2 . . . 79
10.2.7 Table 5, SI 2 . . . 100
10.2.8 Table 6, SI 2 . . . 139
10.2.9 Table 7, SI 2 . . . 190
10.2.10 Table 8, SI 2 . . . 196

1 Additional theoretical results

1.1 Charge distribution

Charge distribution can change with the conformational variety, but in all investigated Re/Tc-diaqua-dipyrophoshate geometries the partial charge of the cental metal atom (Tc/Re) is below +2, far from the formal charge of +4, which indicates a large degree of electron donation from the pyrophosphate ligands. The Tc/Re(IV) d^3 metal ion itself is very electron poor, in order to reach an ideal 18 e^- state as an octahedral complex, π-donation as well as σ-donation is necessary. The partially delocalized electronic structure of phosphate tetrahedra make them ideal π-donors.

Tc1	+1.67	P11	+1.53	O21	-0.85
O2	-0.75	O12	-0.68	H24	+0.50
O3	-0.82	O13	-0.81	H27	+0.50
O4	-0.64	O14	-0.86	H30	+0.48
O5	-0.81	O15	-0.81	H31	+0.50
O6	-0.70	O16	-0.85	H28	+0.51
O7	-0.66	O17	-0.81	H29	+0.49
P8	+1.31	O18	-0.65	Na28	+0.94
P9	+1.49	O19	-0.82	Na30	+0.94
P10	+1.45	O20	-0.79		

(a)

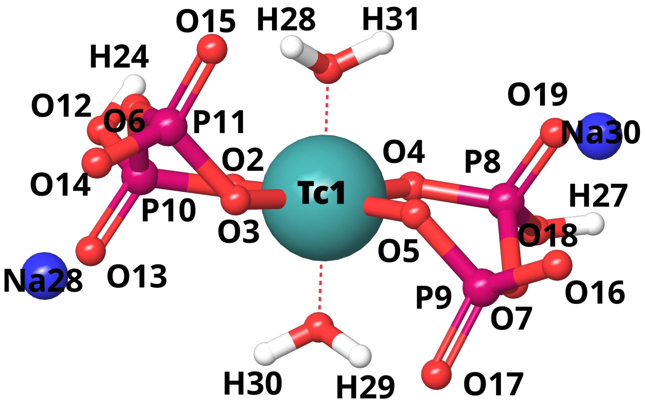

(b)

Table S1: ESP charge distribution calculated for one Tc-PYP conformer.

1.2 Other possible Tc-PYP reaction products and intermediates

The tautomeric mapping hinted at the possibility of a tetragonal pyramidal structure containing a metal-oxygen double bond.

Tetragonal pyramidal geometries are thought to exist for certain Tc(V) tracers [1] (see Figure 1 in Subsection 1 of the main text), but the one known Re(V)-pyrophosphate structure does not contain an oxo group [2], and no oxo group containing Tc/Re(IV) analogues are known.

We found a single plausible trigonal bipyramidal Re(IV) complex structure, with an apical oxo group with our calculations; where the M-O bondlength is 1.74 Å (characteristic of a rhenium-oxygen double bond), the Re-O(P) bond is 2.00 Å and the two Re-O(H;P) bonds were found to be 2.15 Å long (characteristic of rhenium-aqua dative bonds [3]). An isostructural stable geometry was also found when the oxidation state of the metal was increased to +V.

Another type of complex that might be considered in the case of the present reactants is a variant that contains chloride ions on these apical positions, which could be an intermediary state between pertechnetate/perrhenate and the technetium/rhenium-diaqua-dipyrophosphate complex. This complex too is predicted to be stable by calculations.

Neither form was found to have stable geometries where more than four negatively charged atoms are around the center metal, thus they can only exist in acidic media, and could well be the other "$TcPYP_2$" species discovered by Kroesbergen. Nevertheless, no experimental evidence was found for either's existence at the examined pH and reaction mixture composition.

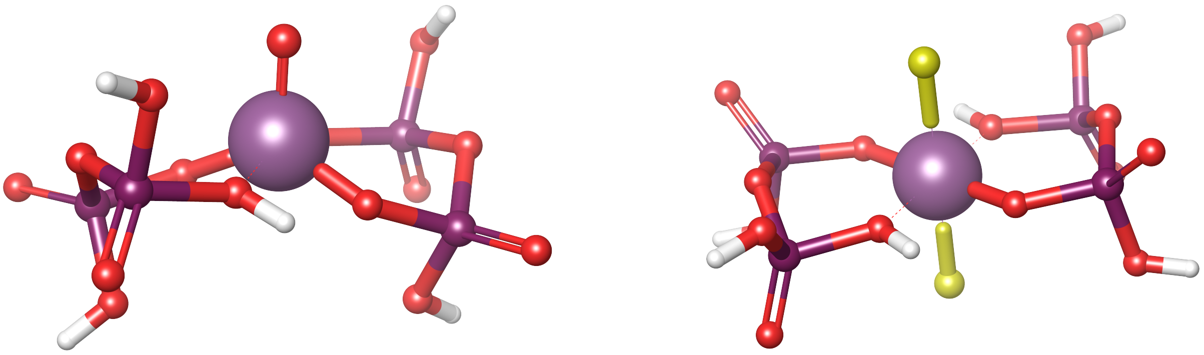

(a) (b)

Figure S1: $ReOPYP_2$ and $RePYP_2Cl_2$, possible intermediers of the complex formation.

1.3 The isostructural nature of Tin(IV)-PYP

The speciation of Duffield et al. [4], as well as our own ^{31}P NMR investigations (see main text) indicated a constitutional formula of $SnPYP_2$ (at near neutral pH). The equivalency in oxidation state and chemical environment, as well as the similarity in electron-negativity and radius to the Tc/Re atoms, has opened the possiblity of these two reaction products being isostructural in solution.

We performed quantum-mechanical calculations on the same level of theory (B3LYP-D3/LACV3P**++), with singlet multiplicity. The resultant Sn(IV)-PYP complex (Figure S2) is more symmetric then its Tc/Re analogues, featuring uniform M-O(PYP) and M-Aqua bond lengths of 2.0 and 2.1 Å respectively.

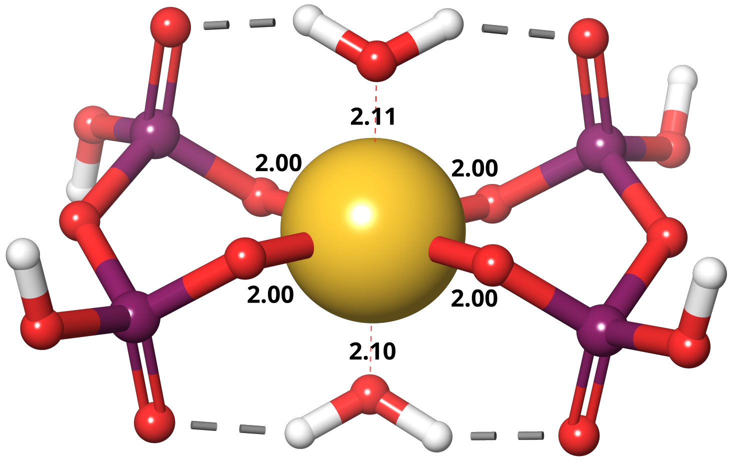

Figure S2: Structure of Sn-PYP refined in vacuum, one of many possible conformers. Tin is shown in yellow; P, O, and H are shown in magenta, red, and white.

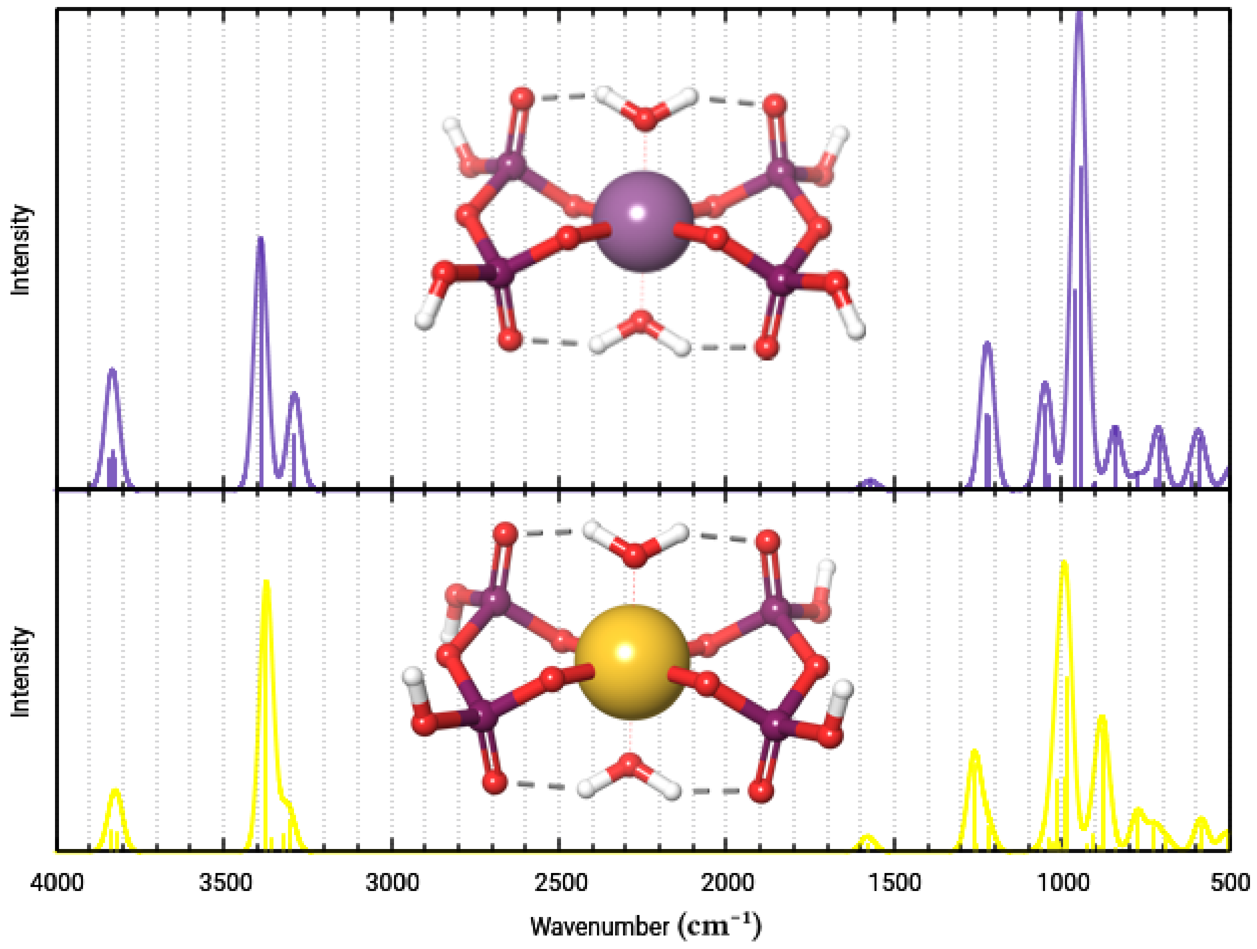

Figure S3: Calculated vibrational spectra of one tin-diaqua-dipyrophosphate conformer and its rhenium analogue. The rhenium-complex is shown in purple, the tin-complex is shown in yellow. Gaussian functions were fitted with a standard deviation of 20.

2 Additional isomeric ensembles

Section 4.3 of the main text showed structural similarities between rhenium and technetium pyrophosphates. Beyond their geometric characteristics, the rhenium analogues are also close in energy to their technetium counterparts, and their structural ensembles show a very similar Boltzmann-distribution at room temperature, reinforcing the congener nature of these two species.

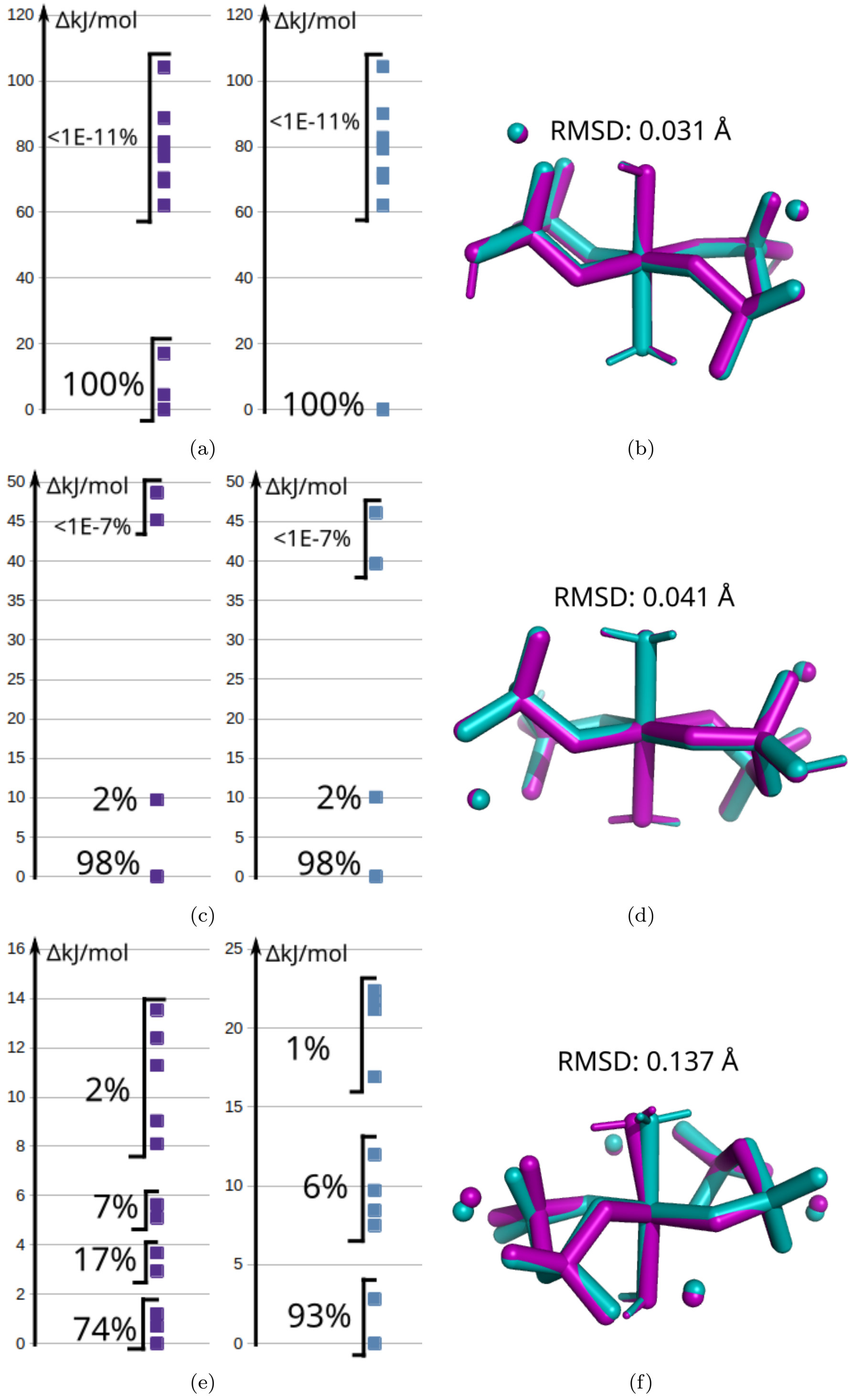

Figure S4: **(a,c,e)** Energy distribution of conformers in the "physiological", "symmetrized", and "neutralized" ensembles (see subsection "Conformational search for isomers and differently protonated species" in the main text), along with Boltzmann-distributions. **(b,d,f)** Similarity of the lowest energy conformers of the respective ensembles, technetium-pyrophosphate is shown in teal, rhenium-pyrophosphate is shown in purple, bond orders are not shown.

Fully protonated, semi-protonated and non-neutraized fully deprotonated forms were also investigated alongside the physiological (semi-protonated) form. As the number of protons decreases, all of the M-O bond lengths increase, by approximately 0.05 Å. The semi-deprotonated and fully deprotonated forms gave more conformers, featuring hexacoordinated geometries where no water-metal bonds are present and both pyrophosphates are tridental, a few monohydroxides, and many monoaqua and diaqua geometries. The fully protonated form yielded less diaqua conformers, and no other isomers found in the fully deprotonated ensemble.

The strength of the H-bridge connecting the pyrophosphate oxygens and the water molecules and the electron density of the pyrophosphate ligands seem to be correlated, where with increasing the electron density the water O-H stretching vibrations shift to lower vawenumbers and become more dispersed — resulting in a wider band.

	Average	Min	Max
Re-PYP (9 conformers)			
M-OP length (Å)	1.98	1.86	2.09
M-OH_2 length (Å)	2.11	2.08	2.17
α (°)	88.38	86.05	91.20
β (°)	91.61	86.32	96.36
γ (°)	92.25	86.32	96.64
δ (°)	176.96	173.79	179.29
ϵ (°)	119.51	115.69	127.24
ζ (°)	47.47	0.36	70.19
Staggered pyrophosphates:	14	Eclipsed:	4
Tc-PYP (9 conformers)			
M-OP length (Å)	1.95	1.83	2.07
M-OH_2 length (Å)	2.12	2.08	2.19
α (°)	86.88	84.83	88.99
β (°)	93.12	87.45	98.71
γ (°)	91.27	86.81	95.46
δ (°)	177.22	174.08	178.75
ϵ (°)	119.93	115.81	127.87
ζ (°)	44.97	0.33	66.27
Staggered pyrophosphates:	14	Eclipsed:	4

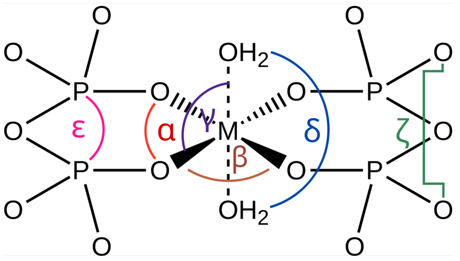

Table S2: General attributes of the diaqua fully protonated form's conformational ensembles.

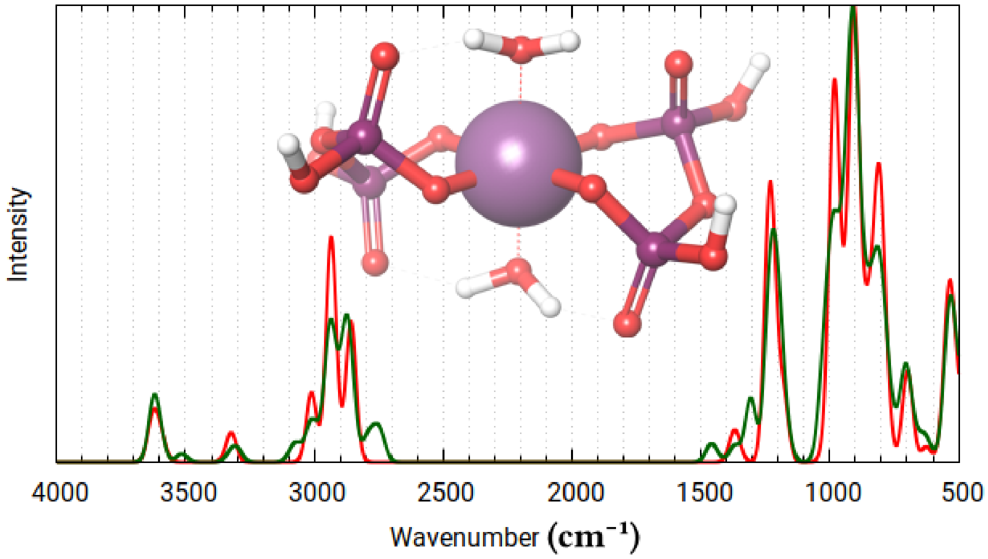

Figure S5: Calculated infrared spectra for the lowest energy conformer (red) and cumulatively all (9) conformers (green), of the fully portonated form of Re-PYP.

	Average	Min	Max
Re-PYP (7 conformers)			
M-OP length (Å)	2.05	1.90	2.16
M-OH_2 length (Å)	2.22	2.11	2.33
α (°)	88.51	86.09	91.24
β (°)	91.40	86.87	88.73
γ (°)	92.34	85.67	95.05
δ (°)	173.49	165.73	179.94
ϵ (°)	129.20	123.93	134.02
ζ (°)	55.63	0.53	95.58
Staggered pyrophosphates:	12	Eclipsed:	2
Tc-PYP (7 conformers)			
M-OP length (Å)	2.00	1.85	2.12
M-OH_2 length (Å)	2.20	2.08	2.29
α (°)	87.69	86.03	89.86
β (°)	92.35	86.87	90.14
γ (°)	91.63	85.63	93.93
δ (°)	176.27	166.59	180.00
ϵ (°)	128.99	123.62	133.82
ζ (°)	56.46	0.18	123.55

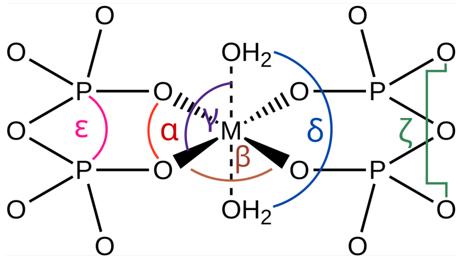

Table S3: General attributes of the diaqua deprotonated form's conformational ensembles.

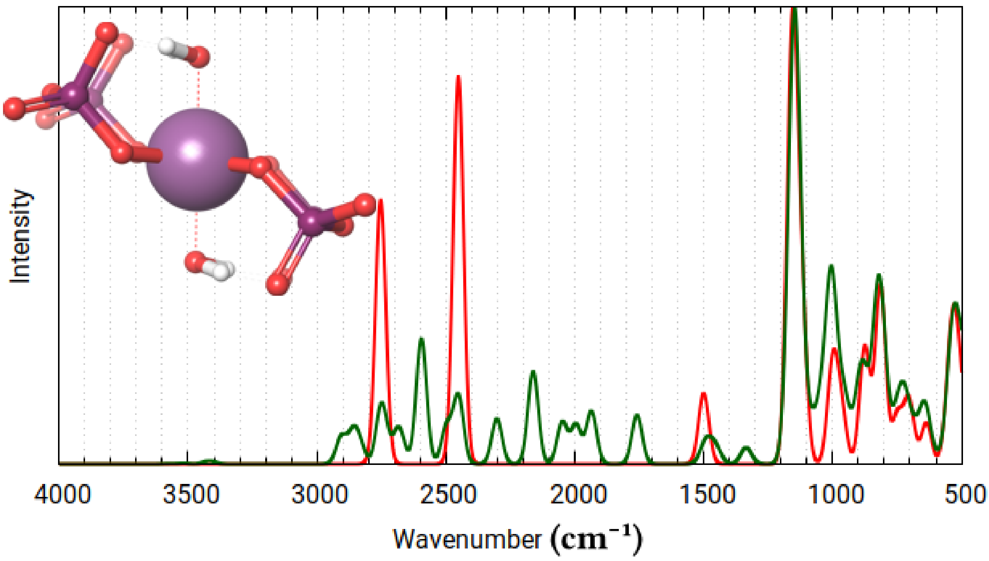

Figure S6: Calculated infrared spectra for the lowest energy conformer (red) and cumulatively all (7) conformers (green), of the diaqua deprotonated form of Re-PYP.

	Average	Min	Max
Re-PYP (24 conformers)			
M-OP length (Å)	2.08	1.89	2.24
M-OH_2 length (Å)	2.32	2.28	2.34
α (°)	86.41	84.37	90.27
β (°)	97.02	87.82	111.03
γ (°)	96.16	81.70	106.38
ϵ (°)	127.36	121.42	133.98
ζ (°)	43.23	4.90	81.59
Staggered pyrophosphates:	33	Eclipsed:	15
Tc-PYP (24 conformers)			
M-OP length (Å)	2.04	1.84	2.28
M-OH_2 length (Å)	2.31	2.26	2.41
α (°)	86.20	84.61	89.51
β (°)	96.92	87.35	109.44
γ (°)	94.79	89.21	105.43
ϵ (°)	126.79	120.50	133.49
ζ (°)	43.48	4.59	85.18
Staggered pyrophosphates:	33	Eclipsed:	15

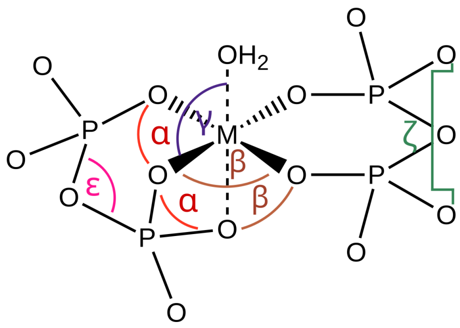

Table S4: General attributes of the monoaqua deprotonated form's conformational ensembles.

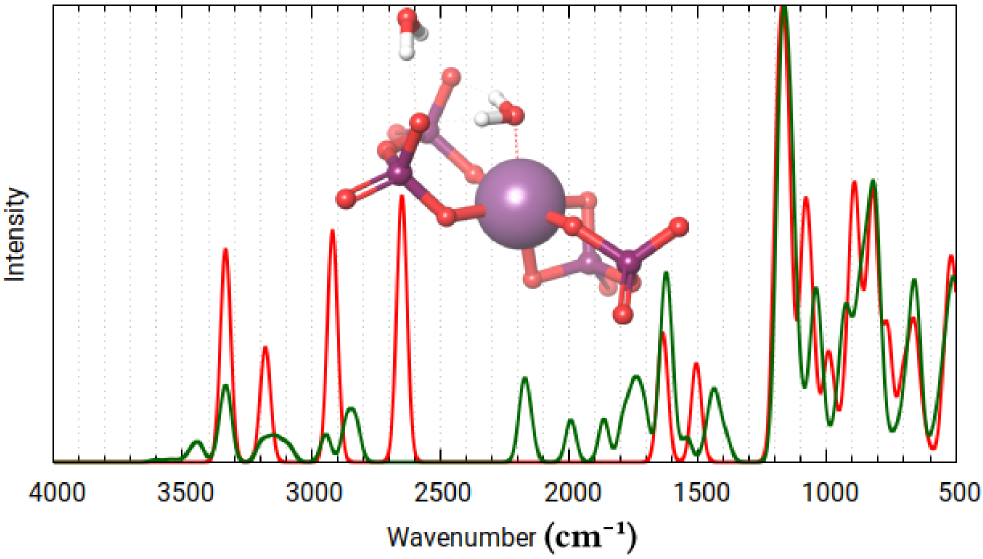

Figure S7: Calculated infrared spectra for the lowest energy conformer (red) and cumulatively all (24) conformers (green), of the monoaqua deprotonated form of Re-PYP.

	Average	Min	Max
Re-PYP (45 conformers)			
M-OP length (Å)	2.12	1.90	2.33
α (°)	86.12	82.74	88.83
β (°)	94.64	91.45	101.62
ϵ (°)	122.94	120.63	124.20
ζ (°)	71.34	64.50	74.51
Staggered pyrophosphates:	90	Eclipsed:	0
Tc-PYP (43 conformers)			
M-OP length (Å)	2.09	1.85	2.27
α (°)	86.15	82.95	87.77
β (°)	94.75	91.31	103.39
ϵ (°)	122.01	119.00	123.13
ζ (°)	71.32	61.66	74.56
Staggered pyrophosphates:	86	Eclipsed:	0

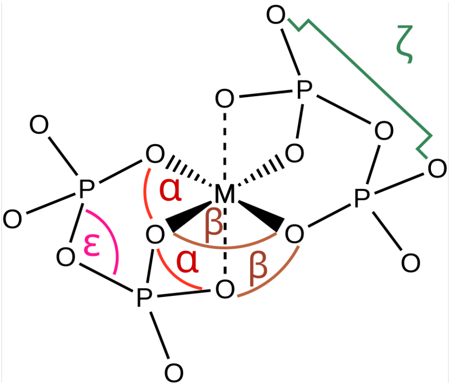

Table S5: General attributes of the tridental deprotonated form's conformational ensembles.

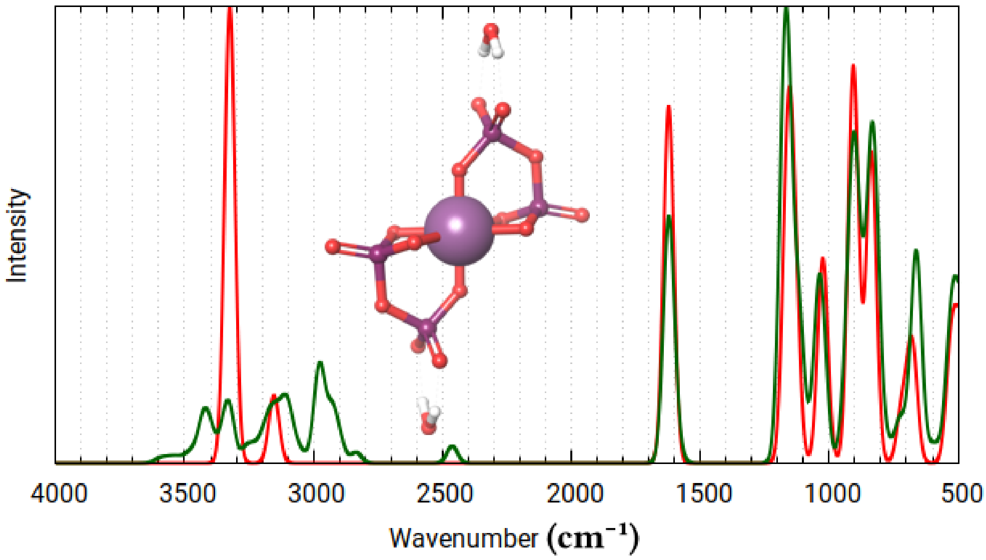

Figure S8: Calculated infrared spectra for the lowest energy conformer (red) and cumulatively all (45) conformers (green), of the tridental deprotonated form of Re-PYP.

	Average	Min	Max
Re-PYP (54 conformers)			
M-OP length (Å)	2.02	1.84	2.21
M-OH_2 length (Å)	2.20	2.14	2.31
α (°)	86.75	83.12	91.17
β (°)	93.41	82.93	114.16
γ (°)	89.78	76.64	96.92
δ (°)	173.57	166.32	179.62
ϵ (°)	121.75	114.51	131.31
ζ (°)	33.31	0.24	92.38
Staggered pyrophosphates:	47	Eclipsed:	59
Tc-PYP (53 conformers)			
M-OP length (Å)	1.98	1.81	2.16
M-OH_2 length (Å)	2.19	2.13	2.30
α (°)	86.47	82.85	91.56
β (°)	93.70	83.05	118.49
γ (°)	89.94	77.28	96.08
δ (°)	174.01	165.59	179.74
ϵ (°)	121.08	114.33	130.55
ζ (°)	31.33	1.25	92.06
Staggered pyrophosphates:	42	Eclipsed:	62

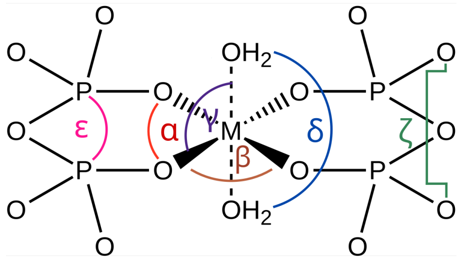

Table S6: General attributes of the diaqua semi-protonated form's conformational ensembles.

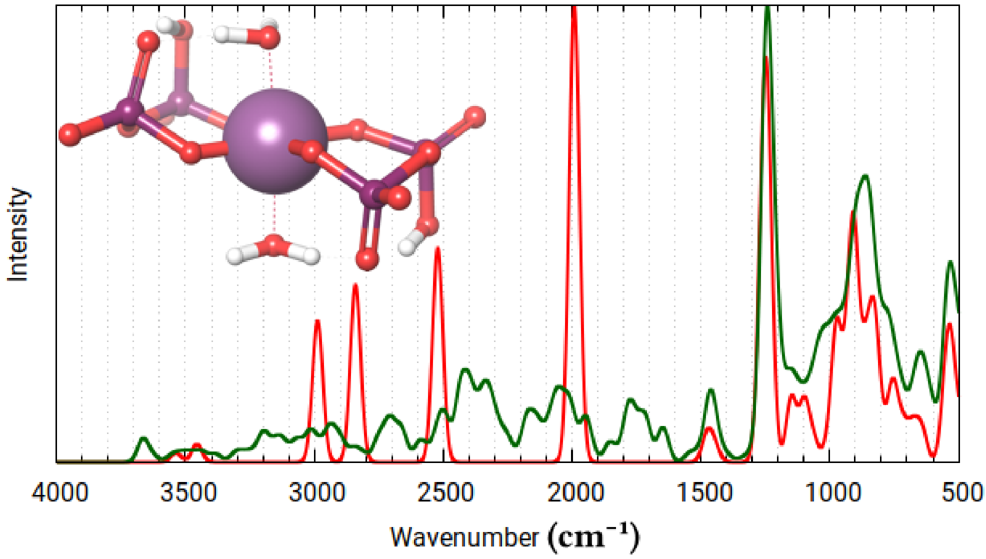

Figure S9: Calculated infrared spectra for the lowest energy conformer (red) and cumulatively all (54) conformers (green), of the diaqua semi-protonated form of Re-PYP

	Average	Min	Max
Re-PYP (6 conformers)			
M-OP length (Å)	2.05	1.87	2.23
M-OH_2 length (Å)	2.26	2.20	2.37
α (°)	88.18	85.97	89.61
β (°)	91.88	87.94	98.79
γ (°)	89.14	88.45	90.56
ϵ (°)	120.68	117.77	123.65
ζ (°)	21.82	0.15	43.98
Staggered pyrophosphates:	7	Eclipsed:	5
Tc-PYP (6 conformers)			
M-OP length (Å)	2.02	1.83	2.13
M-OH_2 length (Å)	2.24	2.24	2.26
α (°)	87.70	87.05	88.08
β (°)	92.29	85.81	99.04
γ (°)	89.60	88.99	90.54
ϵ (°)	120.69	117.28	124.25
ζ (°)	23.08	0.66	43.12
Staggered pyrophosphates:	7	Eclipsed:	5

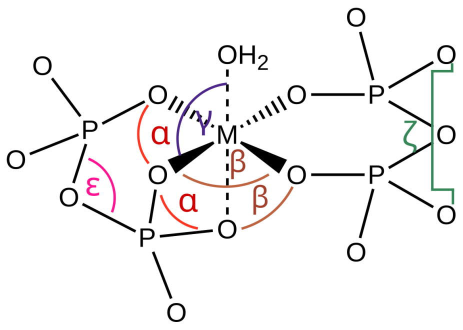

Table S7: General attributes of the monoaqua semi-protonated form's conformational ensemble.

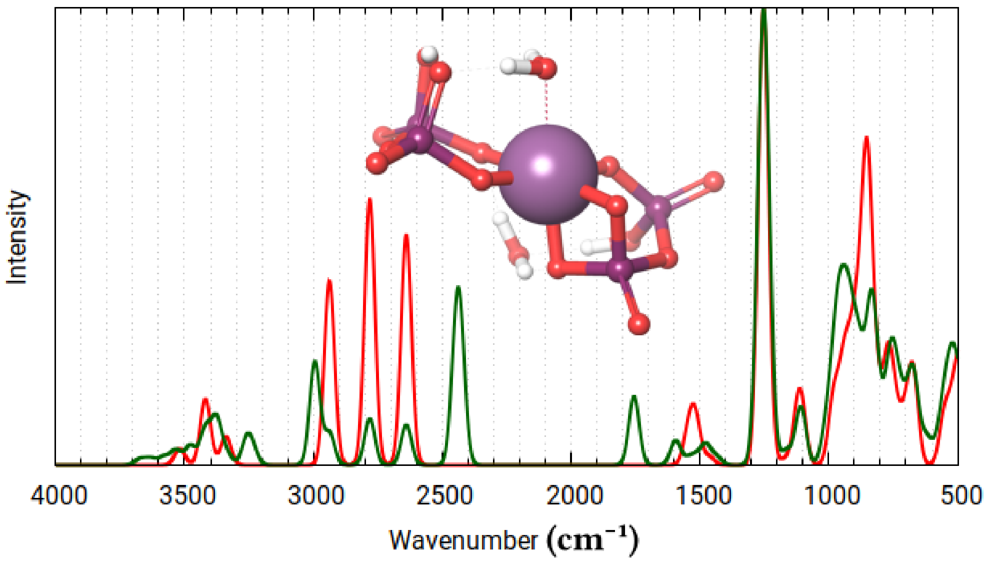

Figure S10: Calculated spectra for the lowest energy conformer (red) and cumulatively all (6) conformers (green), for the monoaqua semi-protonated form.

	Average	Min	Max
Re-PYP (22 conformers)			
M-OP length (Å)	2.07	1.91	2.22
α (°)	87.21	84.83	89.17
β (°)	93.01	86.75	99.74
ϵ (°)	120.05	114.95	124.21
ζ (°)	44.04	39.51	67.50
Staggered pyrophosphates:	44	Eclipsed:	0
Tc-PYP (22 conformers)			
M-OP length (Å)	2.06	1.86	2.25
α (°)	86.97	84.81	88.72
β (°)	93.24	86.22	101.40
ϵ (°)	119.64	115.52	123.49
ζ (°)	42.41	36.51	64.24
Staggered pyrophosphates:	44	Eclipsed:	0

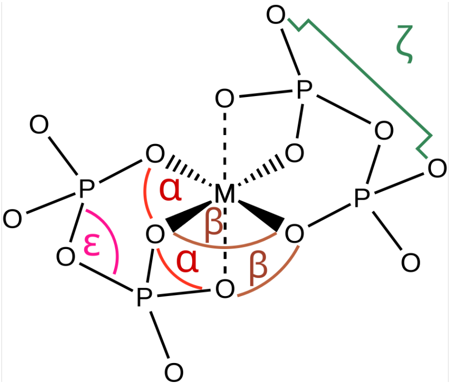

Table S8: General attributes of the tridental semi-protonated form's conformational ensembles.

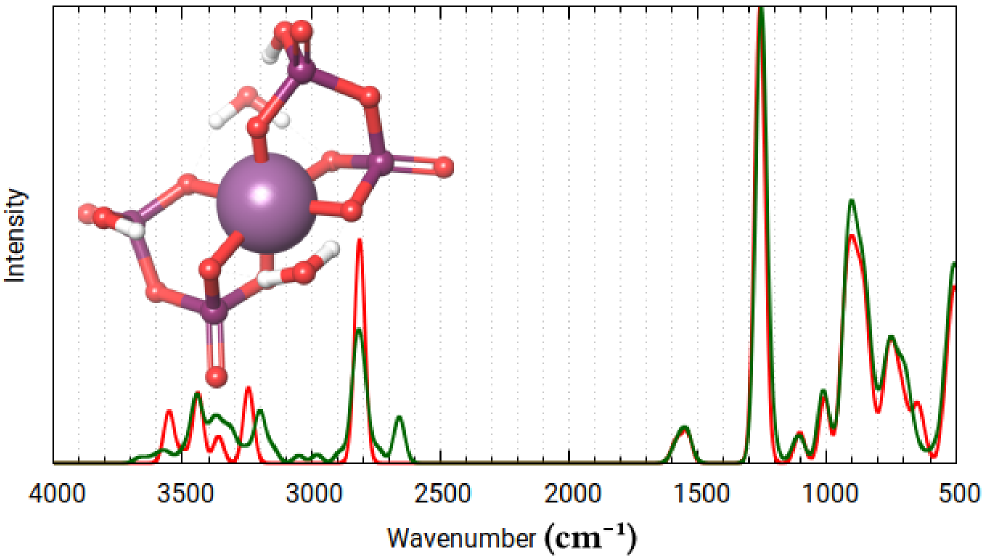

Figure S11: Calculated infrared spectra for the lowest energy conformer (red) and cumulatively all (22) conformers (green), of the tridental semi-protonated form of Re-PYP.

3 Attempts at crystallization

In order to analyze the solid structures of the resulting products with XRD, several attempts at crystallization were performed:

Letting the product mixtures sit in a desiccator at room temperature for two weeks resulted in a residue of colourless crystals encased in a very viscous yellow-green phase. This yellow-green material does not dissolve in alcohol. Further evaporation resulted in only colourless crystals, presumably because the complex is only stable as a hydrate.

Layering diethyl ether onto the product mixtures and letting them sit refrigerated (at 5 °C) for three weeks did not yield any crystals of the products.

Vapor diffusion was attempted with ethanol, methanol, tert-Butanol, isopropanol, acetone, acetonitrile, dioxane, tetrahydrofuran, ethyl acetate, dichloromethane, chloroform, and hexane, but likewise no product monocrystals were produced. In the case of polar solvents, the previously seen viscous phase aggregated at the bottom of the wells.

Complex salt formation in the form of $MRe(HP_2O_7)_2(H_2O)_2$ (where M = Li, Na, K, Cs) via minimizing conductivity around pH = 8.2 with the respective alkaline metal hydroxides also did not result in the crystallization of the product.

In case of very slow, dropwise addition of polar solvents (such as ethanol or those mentioned above) the phase separation into black droplets (as was seen previously) could be avoided, but it instead caused the solution to fade in colour. When observed under a microscope, colourless needle-like and bipyramidal crystals appeared, which were found to be potassium/sodium pyrophosphate and potassium/sodium perrhenate respectively (crystal forms identified via single crystal XRD analysis isostructural with structures found in the Cambridge Structural Database [5] ICSD 15389[6] and CCDC 2001936[7], respectively, see Supplementary Information section "Single crystal X-ray diffraction"). Perrhenate(VII) salts could be found even when only hexachlororhenate(IV) was used for the synthesis (in the case of Mixture H and S).

Crystallization by slow evaporation in a protective nitrogen atmosphere likewise yielded only the previous decomposition products (as verified by SCXRD).

This apparent oxidative decomposition upon nucleation is presently under further study.

4 NMR

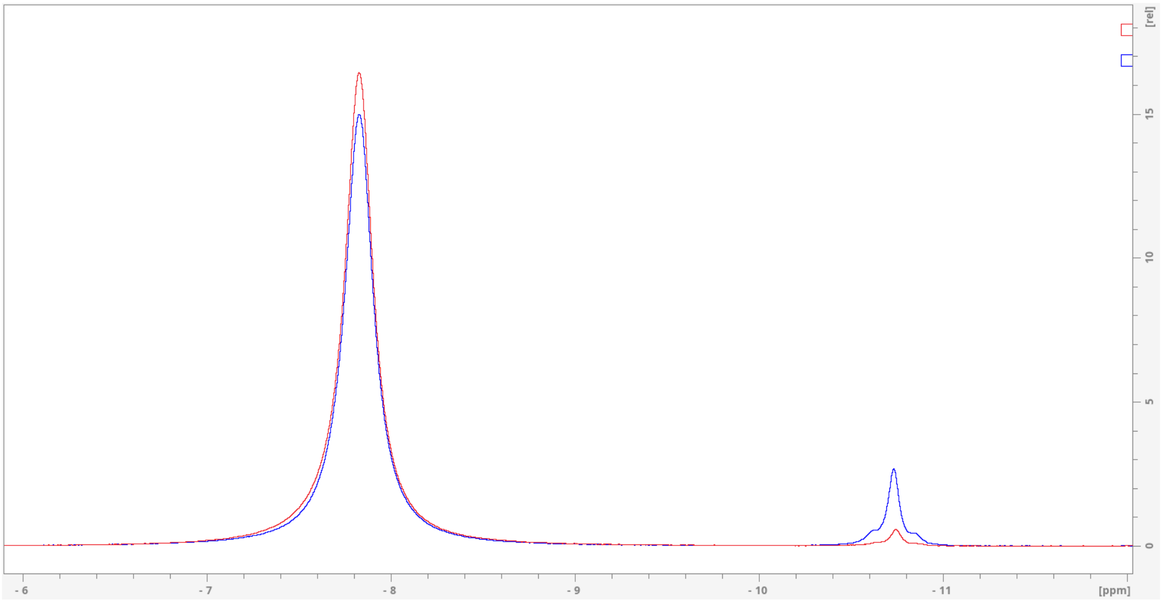

Figure S12: ^{31}P-NMR spectra of the reference mixture containing pyrophosphoric acid and tin(IV) chloride, initially (red), and two days after (blue). The peak corresponding to pyrophosphoric acid phosphorus can be seen at -7.825 ppm, and the peak corresponding to tin(IV)-pyrophosphate can be seen at -10.730 ppm, along with its satellites.

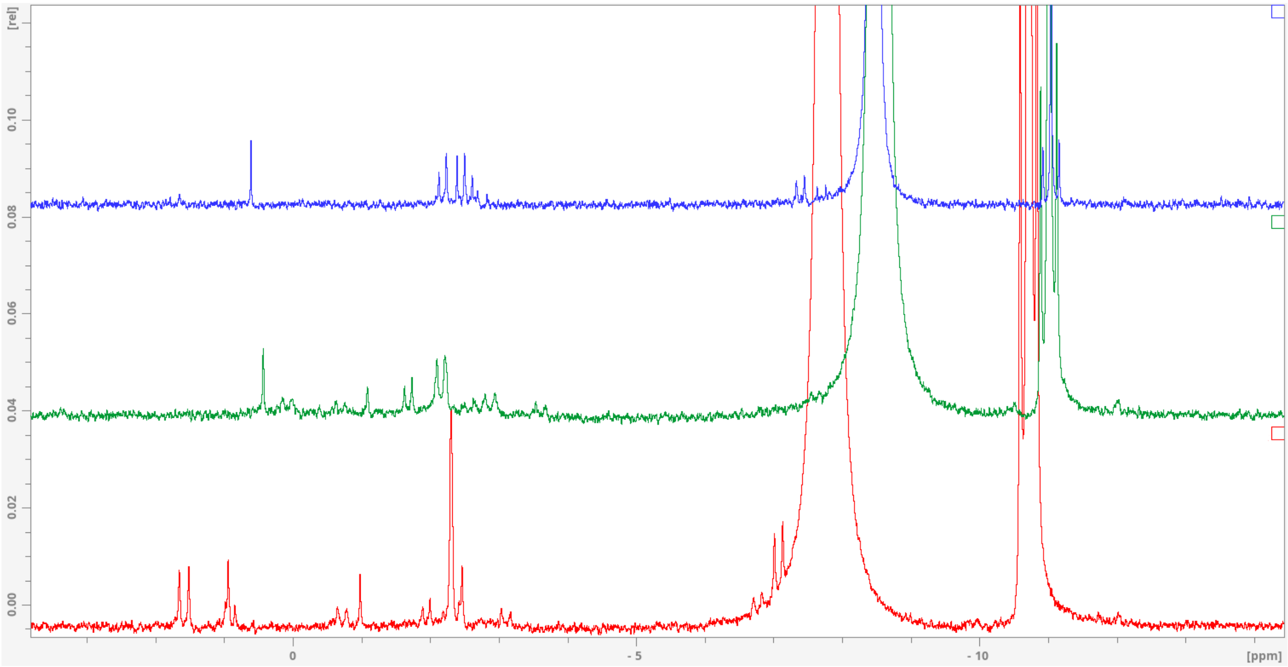

Figure S13: Stacked ^{31}P{^{1}H} small peak spectral regions for P (red), H (green), S (blue) reaction mixtures one hour after reaction start.

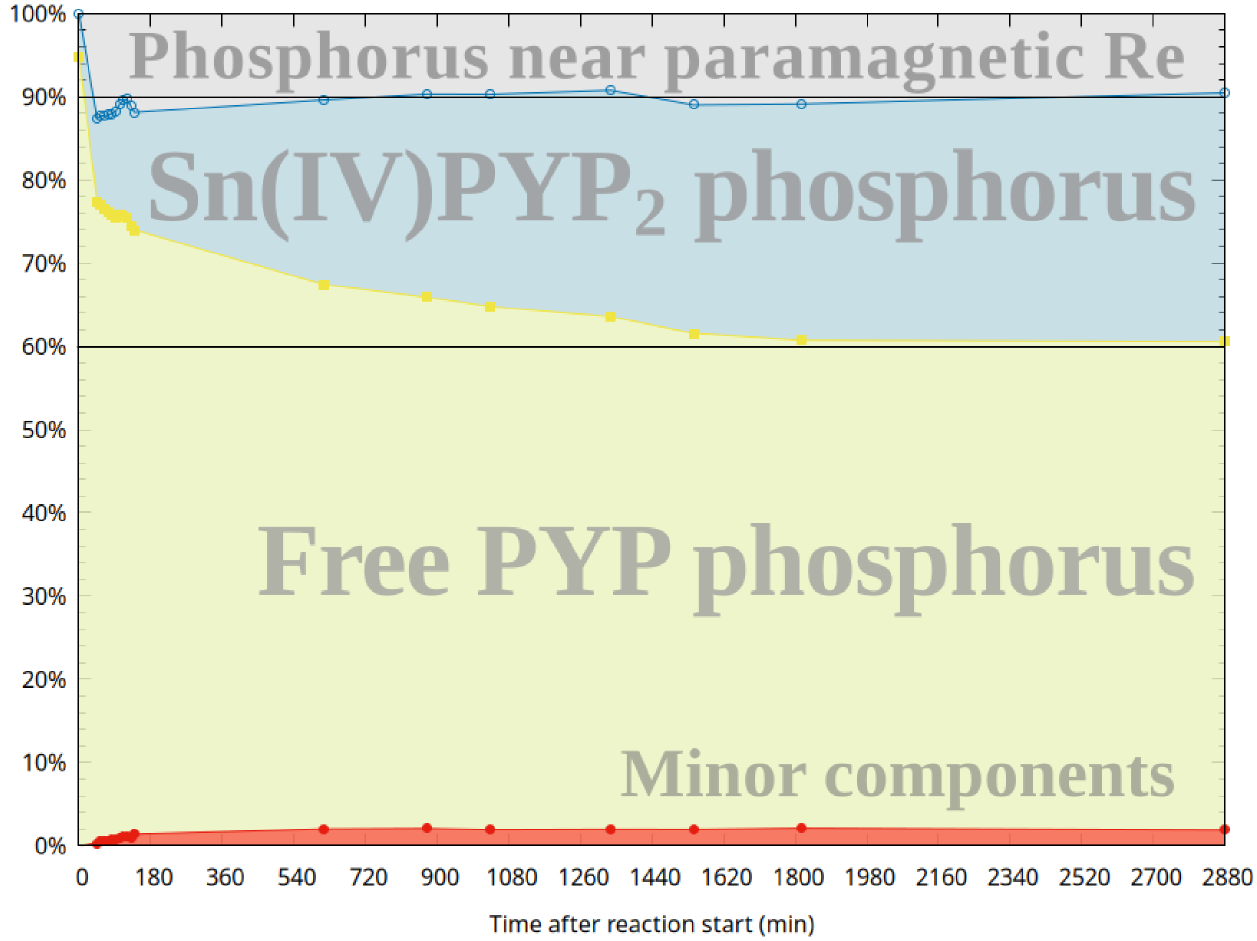

Figure S14: Stacked area charts illustrating the change of component integral (concentration) ratios over time, showing also the decrease of the total signal due to the formation of paramagnetic phosphorus-containing species. Minor components are represented in red, free pyrophosphoric acid phosphorus is represented in yellow, tin(IV) pyrophosphate is represented in blue, signal loss due to phosphorus taking part in a paramagnetic rhenium complex is represented in gray.

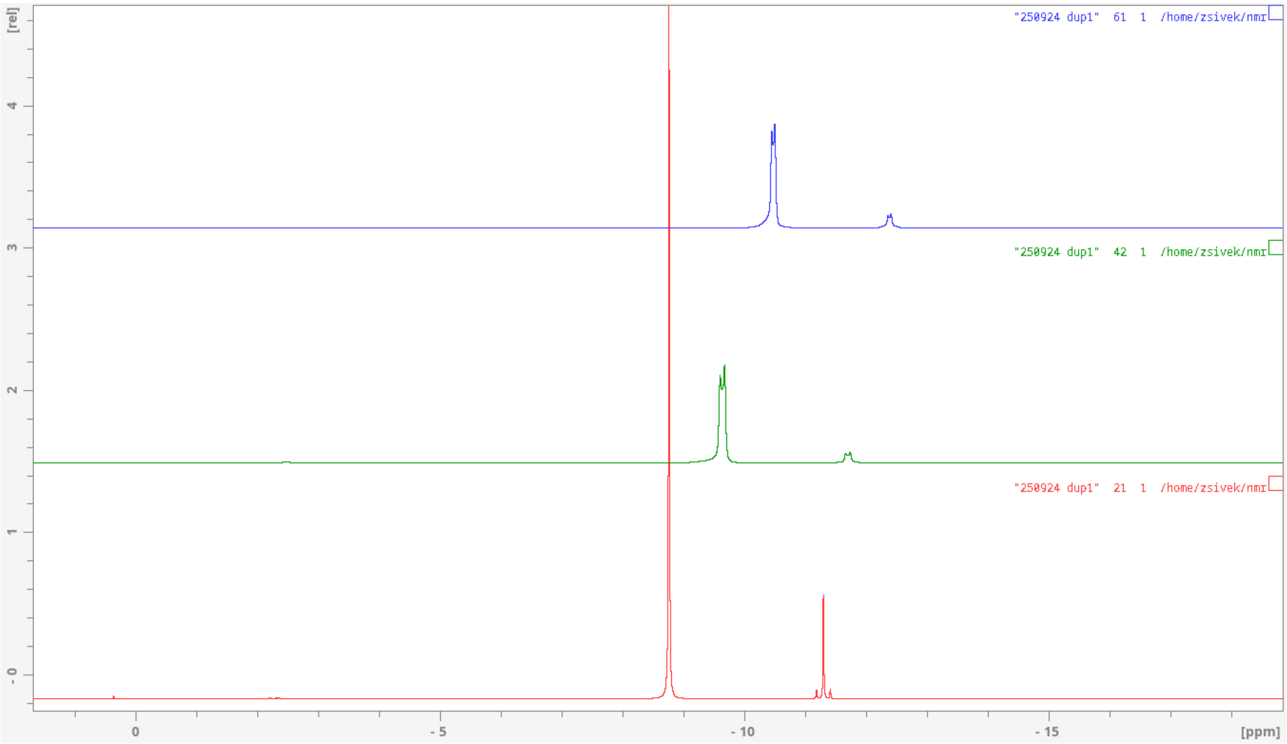

Figure S15: ^{31}P-NMR spectra of H Mixtures with increasing rhenium content. 1:20 (red), 3:20 (green), 5:20 (blue). The spectra grow more distorted with higher concentrations of the paramagnetic species.

5 Mass spectrometry and HILIC

Mass spectrometry was used in an attempt to further confirm the composition of the synthetized $RePYP_2$ complex seen in NMR.

Direct injections of 0.060 to 6 mM aqueous solutions of freshly synthetized Mixture S and Mixture P, to an electrospray ionizer (ESI-MS) resulted only in the oxidation products (ReO_4^- and $H_3P_2O_7^-$) of the complex. Co-solvents could only be used in the most dilute solutions, as all polar solvents resulted in the precipitation of $RePYP_2$ as a yellow-green viscous phase.

Hydrophilic interaction liquid chromatography (HILIC) coupled with UV-Vis spectroscopy and mass spectrometry was attempted, both to investigate the possibility of other UV-Vis active isomers being present in the product mixtures, and to observe the difference in retention of the complex and free pyrophosphoric acid. Almost at the start of the measurement, perrhenate was detected, but pyrophosphoric acid eluted separately, suggesting that the complex decomposed on the column.

These results clearly indicate that the $RePYP_2$ species observed in solution can not exist outside water-rich chemical environments as decomposition occurred both at aerosolization and in the thin hydrated layer of the column.

This is in-line with the observations, that fast oxidative decomposition occurs when the water is fully evaporated from the product mixture, and that the solution loses colour upon the slow addition of polar solvents as reported in Section 5.1 of the main text.

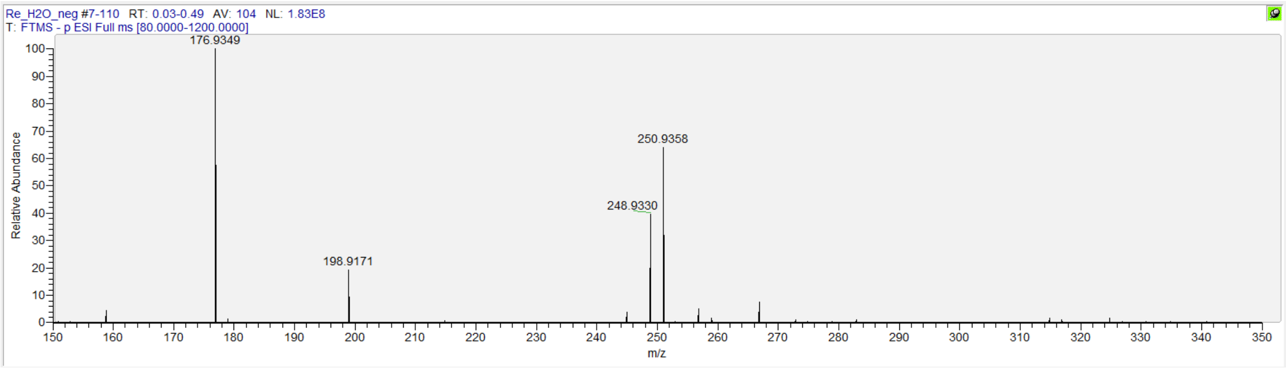

Figure S16: Negative mode ESI-MS spectrum of Mixture S. The peaks at 176.9349 and 198.9171 correspond to $H_3P_2O_7^-$ and its sodium adduct respectively, and the peaks at 250.9358 and 248.9330 correspond to the isotopic abundance of rhenium in ReO_4^-.

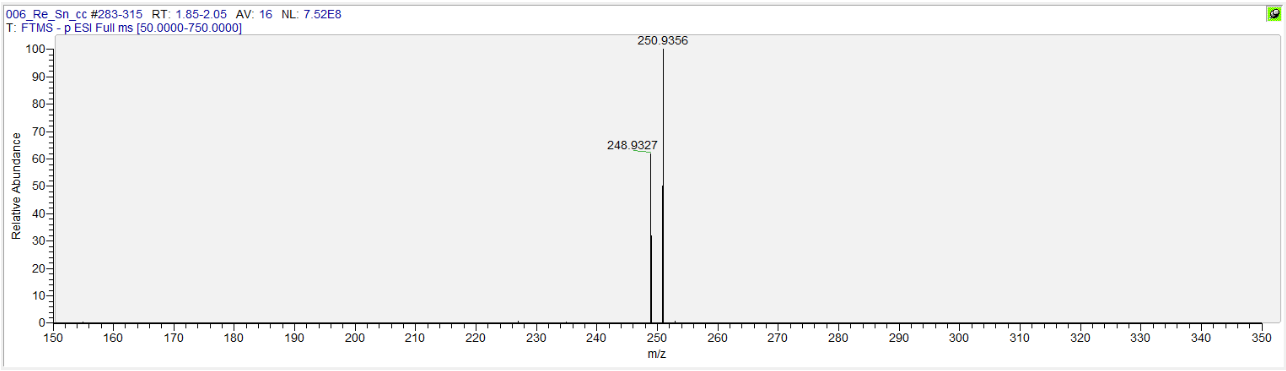

Figure S17: HILIC-coupled negative mode ESI-MS spectrum of Mixture P, at 1.95 min retention time.

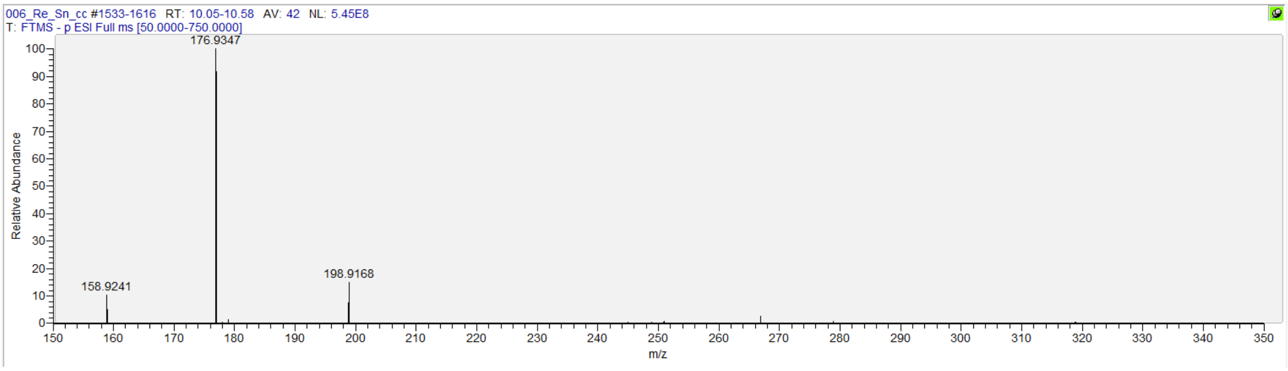

Figure S18: HILIC-coupled negative mode ESI-MS spectrum of Mixture P, at 10.36 min retention time.

6 Mössbauer spectroscopy

After slow evaporation of the solvent from Mixtures P and H, the resulting yellow-green residues were measured using tin Mössbauer spectroscopy, along with two reference mixtures (one at pH 7 and a second at pH 1) containing no rhenium. In all cases the oxidation state of tin was found to be +IV, however analysis of the Mössbauer spectrum of Mixture P indicated the presence of a minor component, with a relative area of less than 2% of the total, which exhibits parameters consistent with Sn(II) (IS = 3.1 ± 0.2 mm/s, QS = 1.9 ± 0.2 mm/s). Even with its limited abundance causing significant errors, the obtained parameters for this component are close to those characteristic of $SnCl_2$. Both references exhibited very close parameters, suggesting that pH value does not influence the surrounding of Sn significantly.

The difference of the Mössbauer parameters between the Mixtures and the reference materials suggests the changes in chemical environment of Sn(IV) nuclei upon addition of Re into the system. While no mixed rhenium-tin species could be identified, these changes may be attributed to formation of several Sn complexes with slightly different structures or to the formation of microcrystalline co-crystals as discussed in the main text (see Section 5.5.4 in the main text).

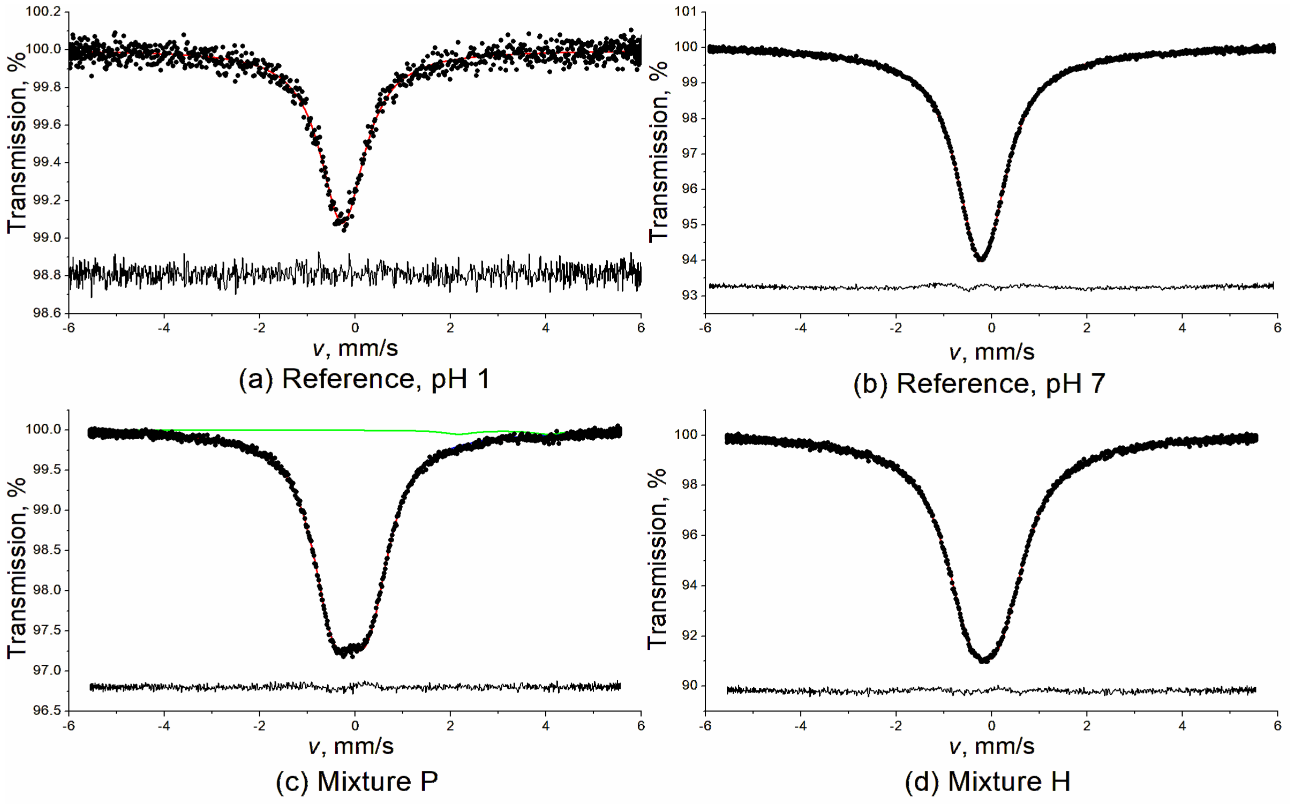

Figure S19: Tin Mössbauer spectra of each sample; all spectra were taken at room temperature.

	δ, mm/s	Δ, mm/s	Γ, mm/s
Reference, pH 1	-0.267 ± 0.005	0 ± 3	1.14 ± 0.04
Reference, pH 7	-0.214 ± 0.001	0.0 ± 0.5	1.250 ± 0.005
Mixture P	-0.095 ± 0.001	0.705 ± 0.002	1.049 ± 0.004
Mixture H	-0.138 ± 0.001	0.629 ± 0.004	1.342 ± 0.005

Table S9: Tin Mössbauer parameters of the main Sn(IV) doublet for each sample. Isomer shift (δ) values are quoted with respect to an SnO_2 reference (Merck) whose isomer shift can be taken to be equal to that of the $CaSnO_3$ source matrix.

7 Pictures of the Reaction Mixtures

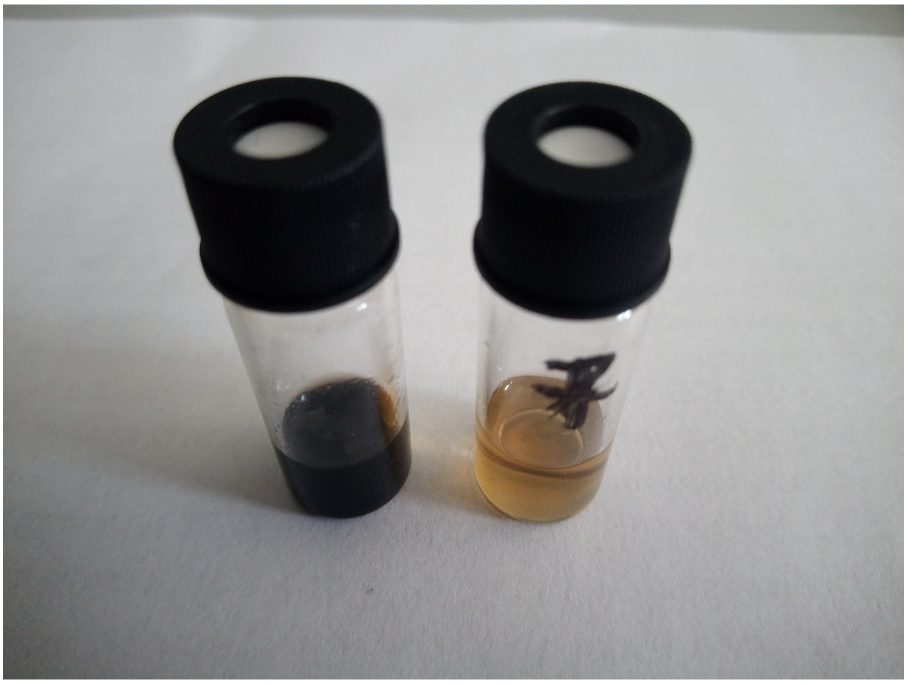

Figure S20: Colour of the product mixtures after two weeks in sealed vials, the one on the left had a pH of 5, and the one of the right had its pH set to 7.40. Both are from the same synthesis of Mixture S.

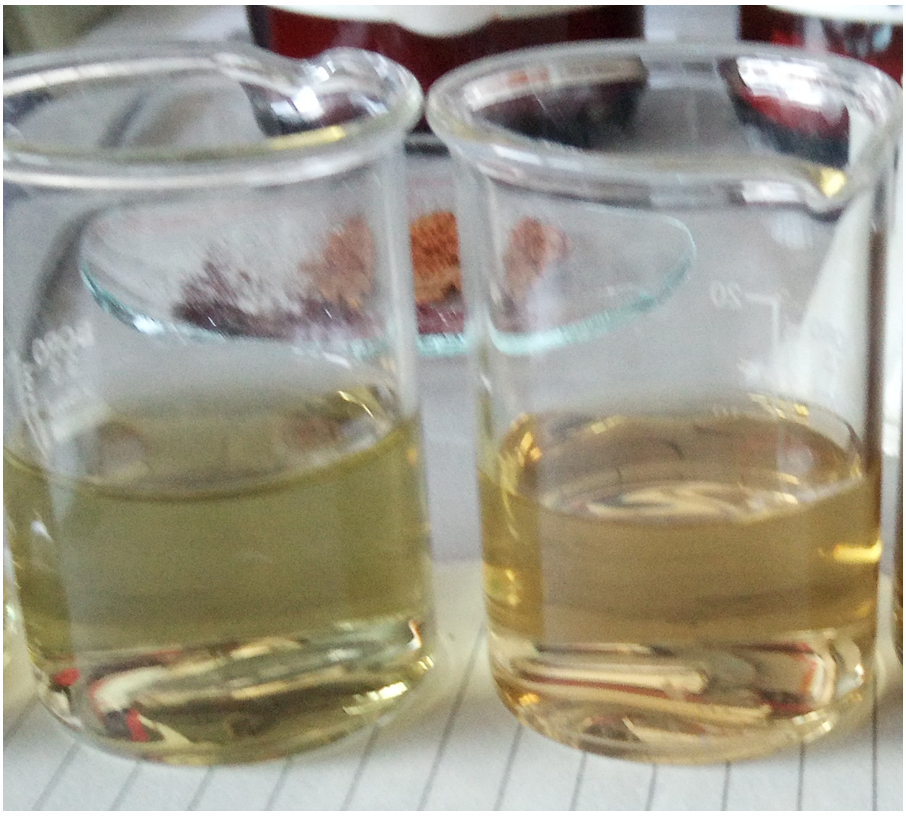

Figure S21: Colour of Mixture P: at pH ∼13 (diluted) to the left, at pH ∼2 (diluted) to the right.

8 Single crystal crystallographic studies (SCXRD)

Attempts of producing crystalline phase of the reaction products were carried out in several conditions. SCXRD was used for determining the chemical composition and identification of the crystal forms. Crystals of all different morphologies were tested. For crystals not diffracting to atomic resolution, unit cell parameters were used to identify the crystal form. In many cases crystals were twinned which made it difficult to collect good quality data. None of the crystals contained the product, instead they contained potassium or sodium pyrophosphate and potassium or sodium- perrhenate respectively. Representative structures are presented below (Figures S23 and S24). with the crystal data compiled in Table S10.

**Compound**	**$Na_4P_2O_7·10H_2O$****	**$KReO_4$****
Colour, crystal habit	colourless plate	colourless bipyramid ***
CCDC accession code	CCDC 2473193	CCDC 2473194
Empirical formula	Na4 P2 O17 H20 ****	K Re O4 *****
Formula weight	446.06	289.3
Temperature / K	301.56(10)	100.01(10)
Radiation (wavelength / Å)	Cu-Kα (1.54184)	Cu-Kα (1.54184)
Crystal system	Monoclinic	Tetragonal
Space group	C2/c	I 4_1/a
a / Å	16.97366(17)	5.64930(10)
b / Å	6.95678(6)	5.64930(10)
c / Å	14.84423(15)	12.5705(5)
α / °	90	90
β / °	111.8403(12)	90
γ / °	90	90
Volume / $Å^3$	1627.02(3)	401.18(2)
Z	4	4
ρ_{calc} / g·cm^{-3}	1.821	4.79
μ / mm^{-1}	4.303	67.616
F(000)	920	504
Crystal size / mm^3	0.619 x 0.397 x 0.089	0.182 x 0.148 x 0.101
2Θ range for data collection / °	7.766 to 150.448	9.258 to 150.948
Index ranges	-20 <= h <= 21<br>-8 <= k <= 8<br>-18 <= l <= 18	-7 <= h <= 6<br>-6 <= k <= 6<br>-15 <= l <= 15
Reflections collected	23098	2986
Independent reflections [R_{int}]	1668 [R_{int} = 0.0408]	208 [R_{int} = 0.1892]
Completeness to Θ = 67.684°	99.90%	100.00%
Data / restraints / parameters	1668 / 0 / 146	208 / 0 / 15
Goodness-of-fit on F^2	1.146	1.361
Final R indexes [I>=2σ(I)]	R1 = 0.0279<br>wR2 = 0.0789	0.0376<br>0.0977
Final R indexes [all data]	R1 = 0.0279<br>wR2 = 0.0789	0.0378<br>0.0979
Largest diff. peak and hole / e·$Å^3$	0.645 and -0.312	1.856 and -2.218

Table S10: Crystallographic data of tetrasodium pyrophosphate decahydrate and potassium perrhenate. *Isostructural with structure published by McDonald and Cruickshank (ICSD 15389)[6]. **Isostructural with structure published by Brown et al. (CCDC 2001936)[7]. ***Data processing revealed fourfold twinning (general twins). ****The asymmetric unit contains 1/2 of the structure. *****The asymmetric unit contains 1/4 of the structure.

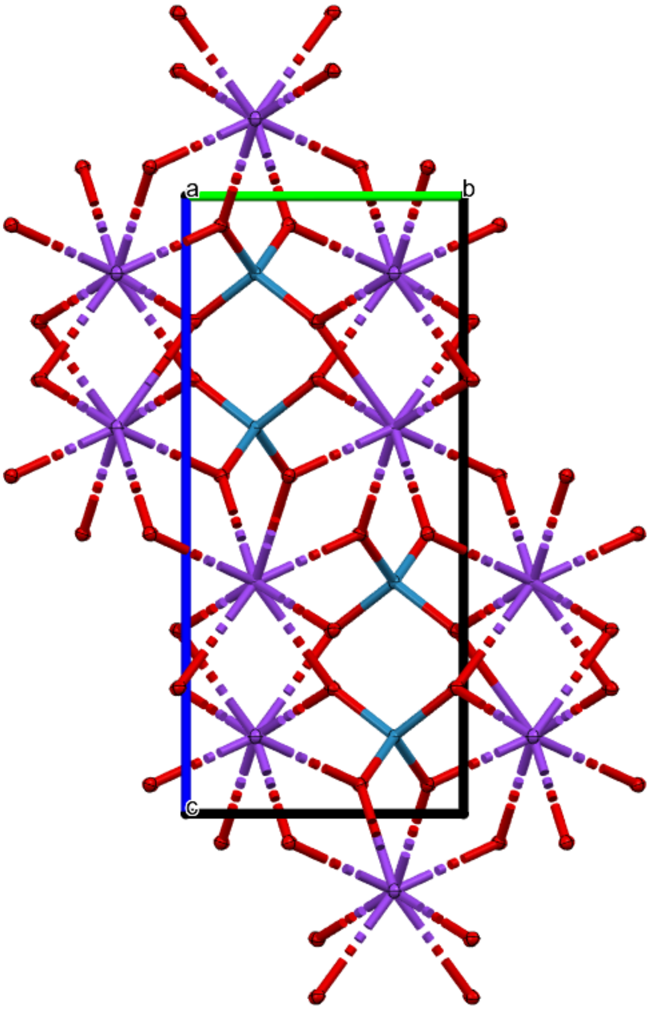

Figure S22: Crystal Structure of tetrasodium pyrophosphate decahydrate ($Na_4P_2O_7{\cdot}10H_2O$). Left: coordination of the pyrophosphate ions and water molecules. Right: crystal packing is shown, view along unit cell axis b. Atomic displacement ellipsoids are contoured at the 0.5 probability level (oxygen: red, phosphorus: orange, sodium: purple).

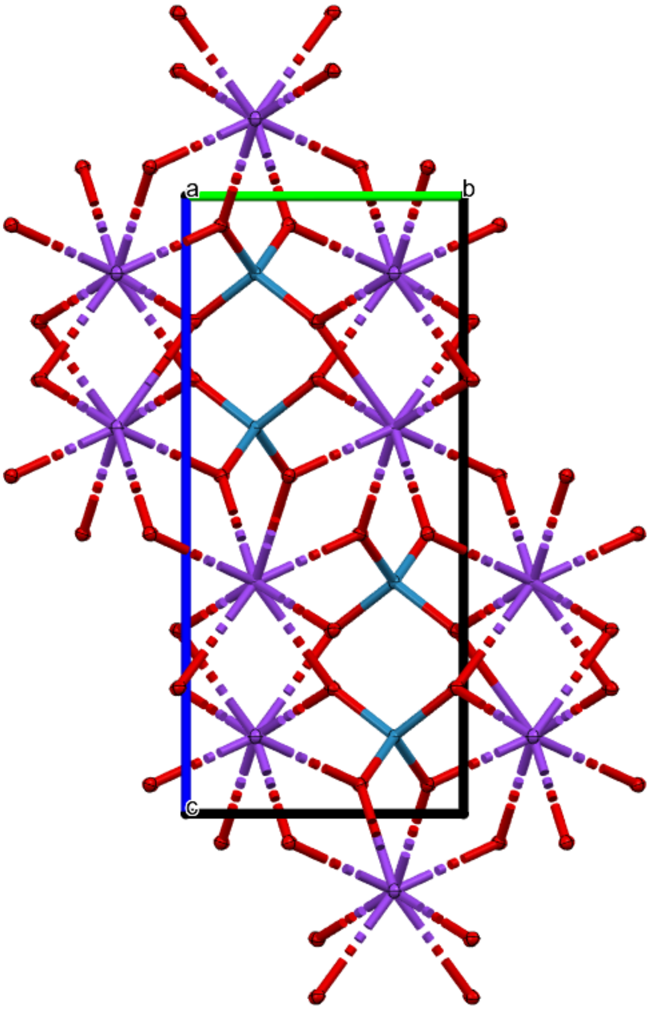

Figure S23: Crystal Structure of tetrasodium pyrophosphate decahydrate ($Na_4P_2O_7{\cdot}10H_2O$). Left: coordination of the pyrophosphate ions and water molecules. Right: crystal packing is shown, view along unit cell axis b. Atomic displacement ellipsoids are contoured at the 0.5 probability level (oxygen: red, phosphorus: orange, sodium: purple).

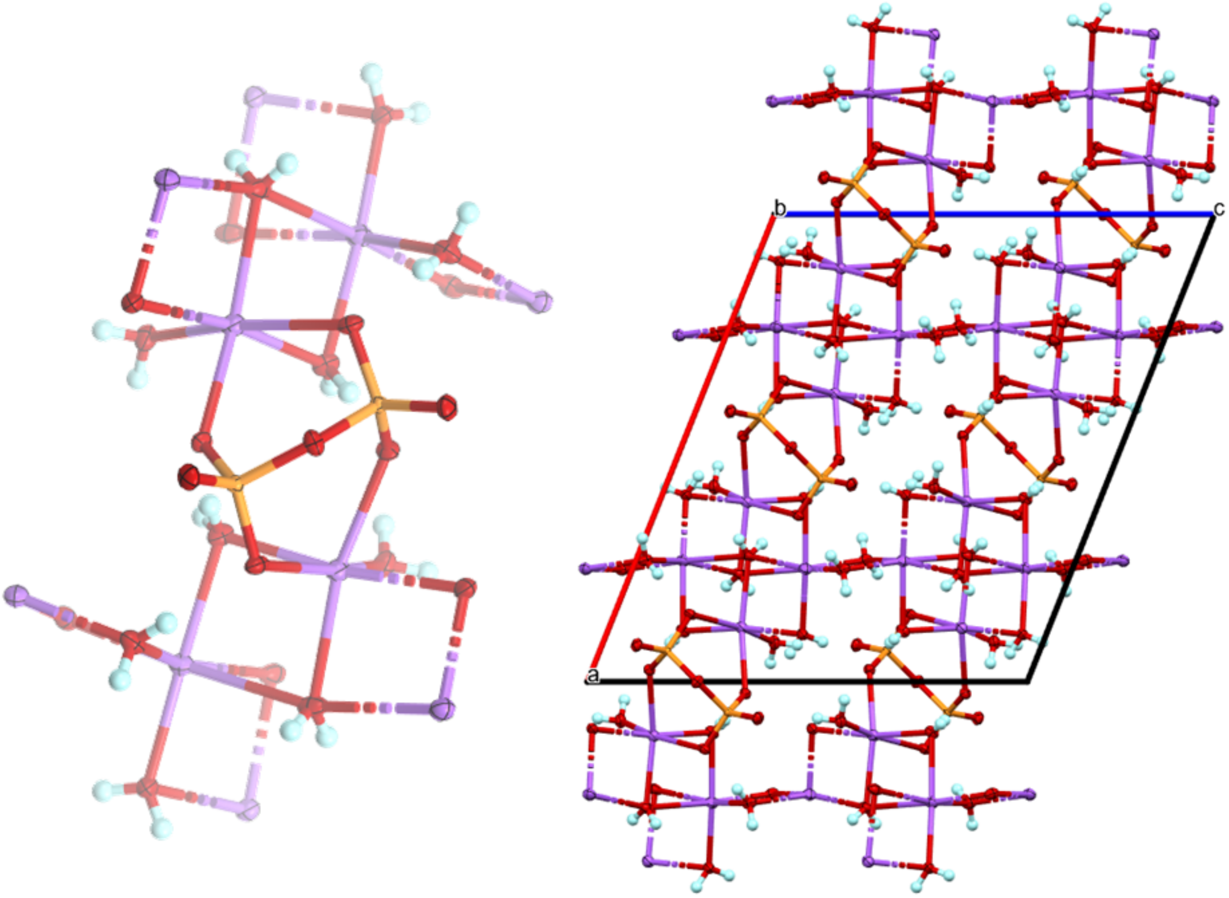

Figure S24: Crystal Structure of potassium perrhenate ($KReO_4$). Crystal packing is shown, view along unit cell axis a. Atomic displacement ellipsoids are contoured at the 0.5 probability level (oxygen: red, rhenium: cyan, potassium: purple).

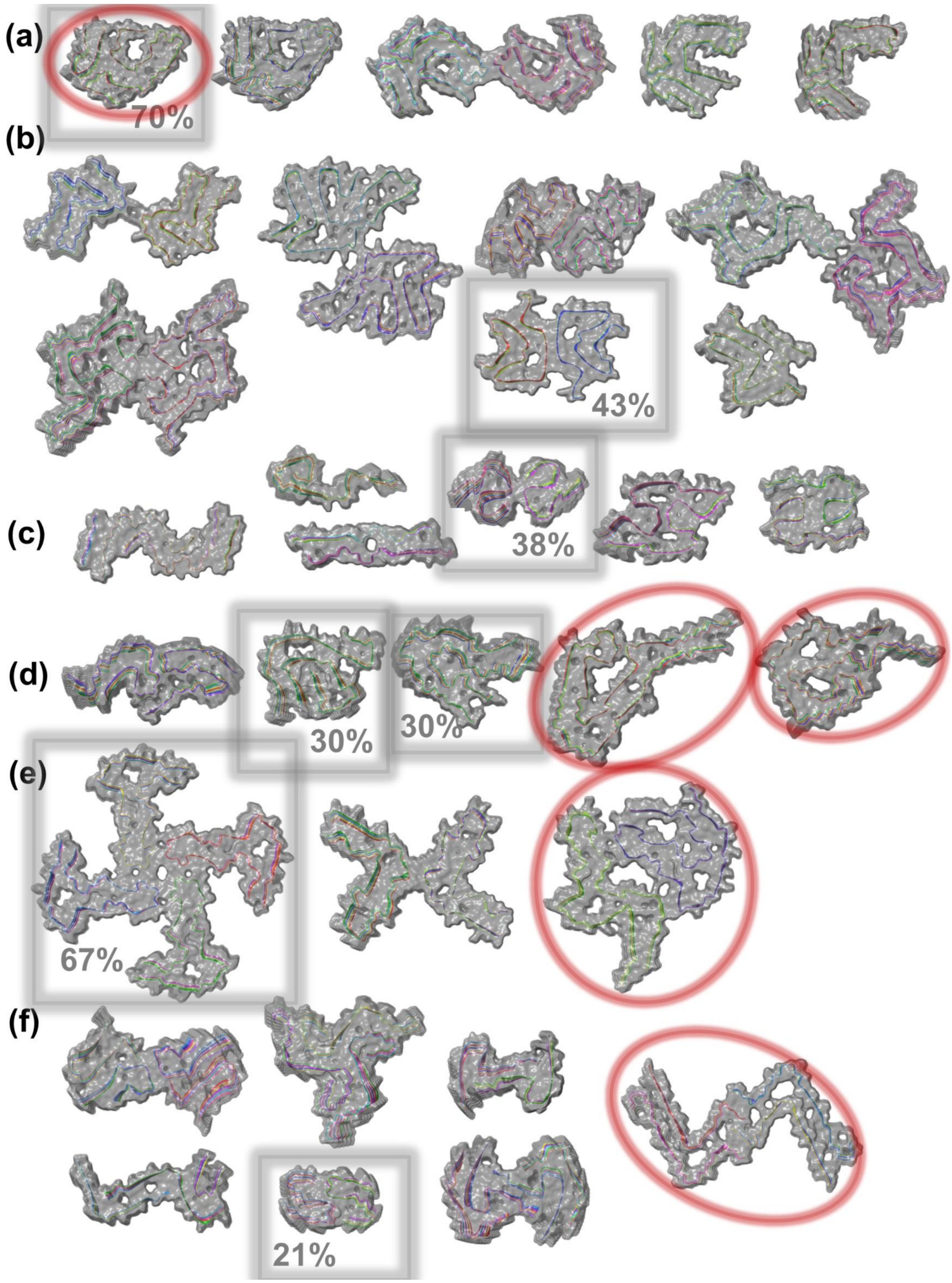
(a)
70%
(b)
43%
(c)
38%
(d)
30%
30%
(e)
67%
(f)
21%

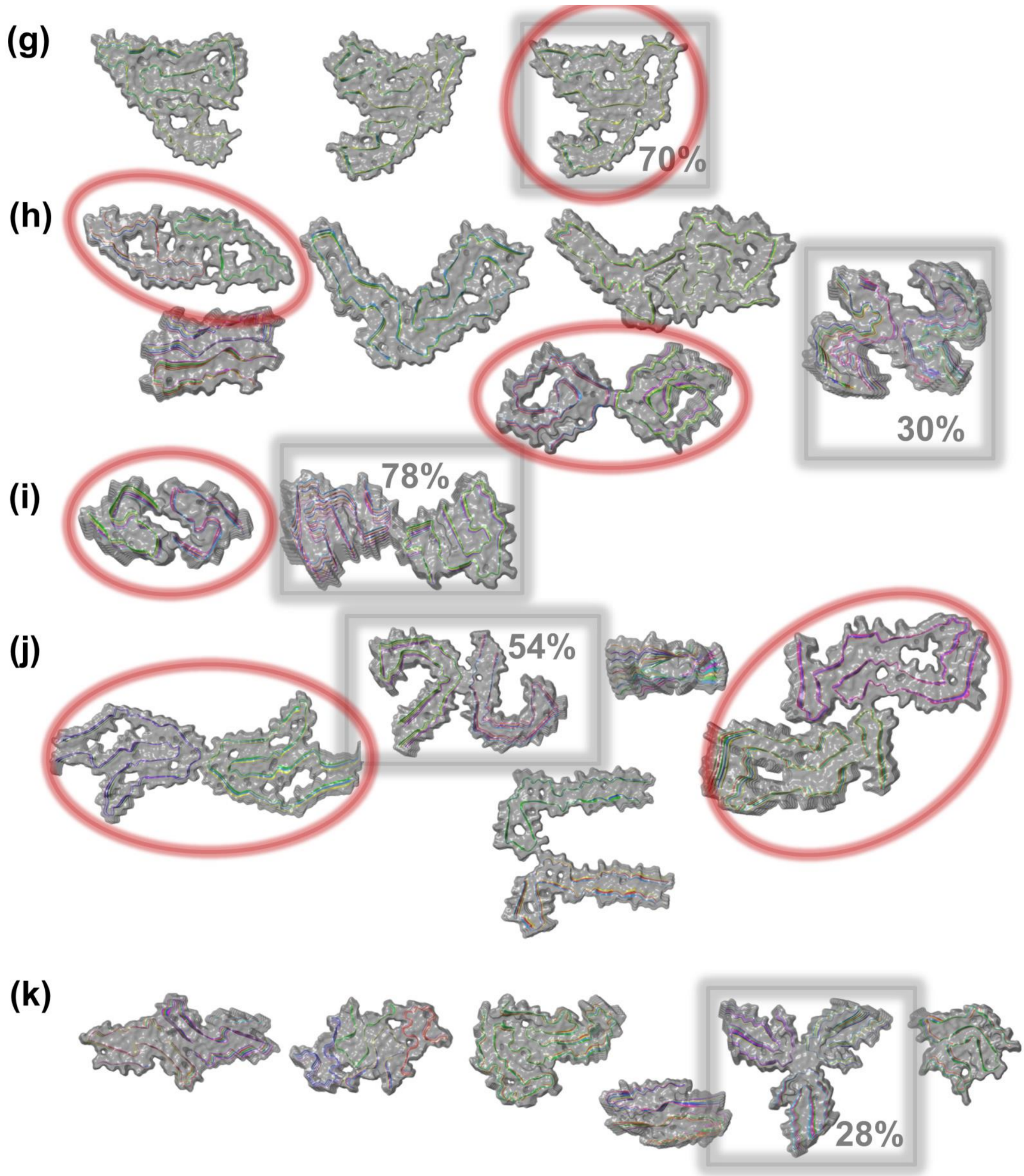

Figure S25: Cluster mid-structures (most typical structure of clusters) of the 11 different proteins for which amyloid structure is available in PDB. Grey boxes show the most populated cluster for each protein, red circles indicate the structures with inner channels comparable (or larger than) that of transthyretin. These are shown in more detail on **(a)** Transthyretin (7ob4, 8e7i, 8eke, 8pkf, 8tdo), **(b)** alfa-synuclein (6l1u, 7v48, 8a4l, 8x7b, 8y2q, 8zmy, 9d5c), **(c)** amyloid beta (7q4m, 8bfa, 8kf4, 8ot1, 8owd, 9k0e), **(d)**. Ig light chain (AL) amyloid (6hud, 6ic3, 6z1l, 7nsl, 9faa), **(e)** beta-microglobulin (6gk3, 8a7o, 8a7q), **(f)** human islet amyloid polypeptide (7yl7, 8az3, 8az4, 8az7, 8qj1, 8r4i, 9gzx), **(g)** Lysosomal type II transmembrane protein 106B (TMEM106B) (7qvc, 7qwg, 8x5h), **(h)** Prion protein (6uur, 7dwv, 7rl4, 7qig, 7umq, 9dmy), **(i)** serum amyloid A (6dso, 6zcf), **(j)** tau (6vh7, 7p67, 8ppo, 8q9c, 9czl), **(k)** transactive response DNA-binding-protein-43 (TDP43) (6n37, 6n3a, 6n3c, 7kwz, 8cgh, 8qxb).

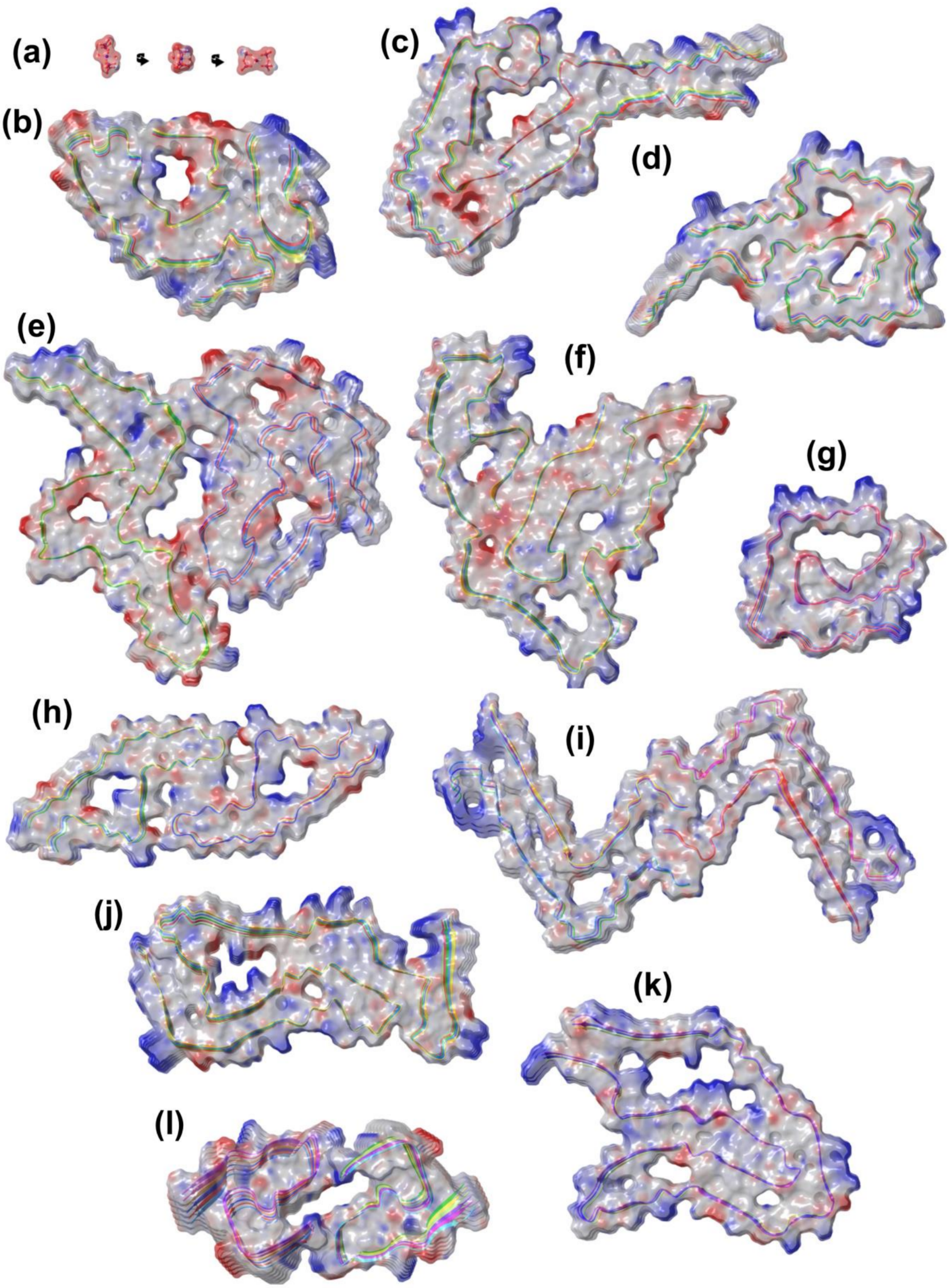

Figure S26: Channels of comparable size to that of **(a)** the diaqua complex of Tc-PYP. **(b)** 8e7i (transthyretin), **(c)** 9faa (Ig light chain (AL)), **(d)** 6ic3 (Ig light chain (AL)), e. 8a7o (beta microglobulin), **(f)** 7qvc (lysosomal type transmembrane protein 106B (TMEM106B)), **(g)** 7umq (prion), **(h)** 7dwv (prion), **(i)**, 7yl7 (human islet amyloid polypeptide) **(j)** 7p67 (tau), k. 6vh7 (tau), **(l)** 6zcf (serum amyloid A).

References

[1] Klaus Schwochau. *B. 99m Tc Radiopharmaceutical Applications*, chapter 13, pages 371–423. John Wiley & Sons, Ltd, 2000.

[2] Saiful M. Islam and Robert Glaum. Rhenium(V)-oxidepyrophosphate Re2O3(P2O7). *Zeitschrift für anorganische und allgemeine Chemie*, 636(1):144–148, 2010.

[3] Refat Mahfouz, Eida Al-Frag, M. Rafiq H. Siddiqui, Waed Z. Al-kiali, and O. Karama. New aqua rhenium oxocomplex; synthesis, characterization, thermal studies, DFT calculations and catalytic oxidations. *Arabian Journal of Chemistry*, 4(1):119–124, 2011.

[4] John R. Duffield, David R. Williams, and Ivan Kron. Speciation studies of the solubility and aqueous solution chemistry of tin(II)- and tin(IV)-pyrophosphate complexes. *Polyhedron*, 10(3):377–387, 1991.

[5] Colin R. Groom, Ian J. Bruno, Matthew P. Lightfoot, and Suzanna C. Ward. The Cambridge Structural Database. *Acta Crystallographica Section B*, 72(2):171–179, Apr 2016.

[6] W. S. McDonald and D. W. J. Cruickshank. A reinvestigation of the structure of $Na_4P_2O_7$. *Acta Crystallographica*, 22(1):43–48, Jan 1967.

[7] R. J. C. Brown, B. M. Powell, and S. N. Stuart. Thermal effects in the structure of potassium perrhenate. *Acta Crystallographica Section C*, 49(2):214–216, Feb 1993.

10 Theoretical structures

10.1 Structures from DFT calculations

10.1.1 Figure 3, Article 3

```
29
6c-ReIVPyp2-OH2-OH2; multiplicity=2
Re   -20.43769   24.98943   -9.05962
O    -22.37551   25.13516   -9.35085
O    -20.74828   23.43876   -7.78304
O    -20.00567   26.59140  -10.24818
O    -18.42758   24.72212   -8.90084
O    -23.28614   23.53134   -7.58965
O    -17.47211   26.63207  -10.27095
P    -18.79878   27.53011   -9.89260
P    -17.49138   25.00612  -10.13248
P    -23.73438   24.80550   -8.56937
P    -21.90321   23.55884   -6.73696
O    -23.96725   25.99693   -7.52255
O    -24.89169   24.48909   -9.41631
O    -21.89728   22.17884   -5.94475
O    -21.85955   24.80404   -5.87697
O    -15.98126   24.73038   -9.70503
O    -17.91598   24.28819  -11.38567
O    -18.71879   28.66468  -11.01032
O    -18.78065   28.04140   -8.48253
O    -20.33907   23.74327  -10.76293
O    -20.43804   26.30013   -7.46859
H    -23.36609   25.95066   -6.75235
H    -22.33233   22.23094   -5.08063
H    -15.60957   23.95720  -10.15564
H    -18.79032   29.55325  -10.63060
H    -21.07340   23.84689  -11.38792
H    -19.77401   27.05055   -7.51837
H    -20.77459   25.96339   -6.59519
H    -19.43135   23.87214  -11.21989
```

10.1.2 Figure 4, Article 3.1

```
143
6c-TcIVPyp2-OH2-OH2_exp_ph7_nagygeopt_var6
Tc    -0.52889   -0.27709    0.09670
O     -1.64688    1.19992    0.85408
O     -1.45998   -1.54974    1.29415
O      0.44370    1.06516   -1.09979
O      0.60531   -1.72767   -0.63712
O     -3.01765   -0.04424    2.63350
O      2.48249   -0.35123   -1.63455
P      1.93766    1.16243   -1.60367
P      1.52615   -1.70710   -1.94425
P     -2.98181    1.27215    1.67380
P     -1.90177   -1.25045    2.79198
O     -2.78155    2.49514    2.66514
O     -4.23824    1.33072    0.86975
O     -2.57843   -2.46175    3.38187
O     -0.70653   -0.69675    3.56925
O      2.43276   -2.90683   -1.93386
O      0.73314   -1.37863   -3.19263
O      1.84480    1.55902   -3.13931
O      2.83880    2.03832   -0.79415
```

O	−2.01931	−0.76295	−1.24440
O	0.86272	0.00478	1.64002
H	−2.09184	3.18300	2.31602
H	1.83513	2.55223	−3.36077
H	−2.21049	−0.35198	−2.14286
H	1.70072	−0.57663	1.59313
H	0.38275	−0.25427	2.49604
H	−2.09153	−1.76619	−1.33555
Na	−0.34251	0.53737	−4.04831
Na	−1.02702	−3.04661	5.01836
O	4.32066	0.62979	−5.37480
H	3.50508	0.81766	−4.89783
H	4.54159	1.44106	−5.87683
O	5.74680	−2.55732	−1.97543
H	4.98473	−3.11937	−1.79343
H	5.44181	−1.62708	−1.92051
O	0.14085	−4.83302	−1.77597
O	5.75317	0.17352	−2.12521
H	5.18978	0.94537	−1.95545
H	6.50707	0.29232	−1.51281
O	0.81585	5.43245	−1.69820
H	0.02505	4.91277	−1.46743
H	1.44916	5.23531	−0.96789
O	5.44060	2.97863	−1.68194
H	4.81814	3.71440	−1.64957
O	0.25828	−4.16674	0.96270
H	−0.67050	−4.33582	1.21124
H	0.26122	−3.24682	0.65260
O	3.08211	−3.77234	0.61353
H	2.92115	−3.52997	−0.32535
H	2.25855	−4.20184	0.89338
O	−1.12644	3.47738	−1.07787
H	−2.09611	3.27914	−1.07209
H	−0.66810	2.63728	−0.90508
O	2.56444	4.61806	0.22732
H	2.65348	3.68970	−0.06162
O	−2.56523	−4.32535	1.22924
H	−2.74101	−3.72144	1.97973
H	−3.04258	−5.14135	1.41969
O	−3.79091	3.01188	−1.25333
H	−3.94533	2.77183	−2.18756
H	−4.06078	2.25952	−0.68798
O	−4.20855	−1.64309	6.49160
H	−4.32202	−2.45390	5.98105
H	−3.26036	−1.63058	6.70973
O	−3.52639	−2.62090	−3.84677
H	−3.23810	−3.19171	−4.56850
H	−3.18141	−3.03397	−3.03195
O	−2.55005	−0.07427	−3.67005
H	−3.18117	0.65827	−3.83392
H	−2.97919	−0.93027	−3.90271
O	−1.80996	−4.28780	6.74573
H	−2.71146	−4.60590	6.87571
H	−1.31387	−4.60299	7.51072
O	1.22356	−2.91074	4.89177
H	1.70032	−2.06921	4.75165
H	1.88634	−3.52911	5.21852
O	−2.21896	−3.35793	−1.43466
H	−1.37375	−3.81297	−1.65681
H	−2.47141	−3.69252	−0.54934
O	3.05073	−1.33885	1.79394

H	3.09636	−2.25140	1.40881
H	3.15270	−1.39976	2.76301
O	−0.95557	1.71977	4.98911
H	−1.62805	2.12097	4.42221
H	−0.80067	0.85318	4.56946
O	−1.15318	4.21467	1.74505
H	−0.25118	4.23725	2.13295
H	−1.04543	4.06935	0.78620
O	1.40075	4.36582	2.79636
H	1.55257	5.02449	3.48417
H	1.80706	3.52421	3.10745
O	2.40260	1.85335	3.20145
H	3.19844	1.74486	2.62645
H	1.69733	1.47791	2.64783
O	2.87785	−0.66379	4.50283
H	2.68833	0.26733	4.26711
H	3.60841	−0.63824	5.13207
O	2.44614	−0.59730	−5.28694
H	1.91222	−1.05181	−4.60402
H	2.85591	0.13089	−4.80125
O	4.34278	1.15298	1.36662
H	3.90528	1.48310	0.55735
H	4.09859	0.21001	1.39413
H	0.93796	−4.38572	−2.10331
H	0.27356	−4.83999	−0.80737
H	5.93318	2.95974	−0.81267
H	2.18887	4.58256	1.12716
O	0.16439	0.55017	−6.32092
H	0.21364	1.31803	−6.90051
H	1.07666	0.20290	−6.24241
O	−3.69990	2.37987	−3.98285
H	−4.30790	2.68957	−4.66416
H	−2.81698	2.75280	−4.18964
O	−1.01187	2.86901	−3.85935
H	−0.31642	3.40239	−4.27629
H	−1.02255	3.17895	−2.93126
O	1.59999	4.02628	−3.81810
H	2.29143	4.44837	−4.34119
H	1.38827	4.63092	−3.05114
O	−1.93944	−0.34655	4.11960
H	−2.47552	0.27368	3.61373
H	−2.10997	−0.15411	5.06452
O	−6.61899	−0.91732	3.21361
H	−7.49315	−1.32227	3.25382
H	−6.74658	0.05417	3.24980
O	−3.88874	−2.87554	2.49957
H	−4.58010	−2.21921	2.68189
H	−4.35957	−3.73231	2.54198
O	−6.14564	−1.99117	4.01188
H	−6.71675	−1.24417	4.22564
H	−6.72921	−2.73829	3.69583
O	−4.46769	4.85855	0.83286
H	−4.18285	4.28146	1.55174
H	−4.27952	4.34549	0.02776
O	−6.84368	2.49000	−0.26301
H	−7.67508	2.83077	0.08429
H	−6.39431	3.24425	−0.69680
O	−6.06998	1.76135	3.15612
H	−6.49647	2.62703	3.25942
H	−5.41667	1.73174	3.88402
O	−5.91184	4.57344	3.63563

H	−6.38012	5.41531	3.59280
H	−5.57937	4.45868	4.57100

10.1.3 Figure 1, SI 1.2

26
5c–ReIVPyp2H–O; multiplicity=2

Re	−0.56078	0.17575	−0.39860
O	−1.67206	−1.44581	−0.08733
O	−0.89570	0.87804	1.60688
O	−0.13033	−0.84924	−2.22449
O	1.37188	0.54477	−0.01975
P	2.66227	0.27129	−0.86528
P	1.11540	−0.59716	−3.33292
P	−2.47726	1.13025	2.16452
P	−2.66284	−1.72635	1.11333
O	2.08512	0.26746	−2.44465
O	−3.13357	−0.19989	1.57048
O	3.81684	1.16312	−0.75692
O	2.92394	−1.29510	−0.60484
O	1.52939	−1.87106	−3.92882
O	0.52833	0.50993	−4.31026
O	−2.52413	1.35445	3.60678
O	−2.99306	2.29679	1.23860
O	−1.67185	−2.16909	2.30222
O	−3.82389	−2.58507	0.87194
O	−1.52579	1.49497	−1.00664
H	3.83249	−1.56685	−0.78689
H	0.35960	0.17834	−5.20165
H	−2.69024	2.21561	0.30334
H	−2.11395	−2.69611	2.97995
H	−0.62308	−1.67732	−2.36137
H	−0.19885	1.34083	2.09872

27
6c–ReIVPyp2H4–Cl–Cl

Re	−0.55639	0.11608	−0.43698
O	−2.04271	−1.22301	−0.39338
O	−0.70895	0.97745	1.38862
O	−0.17655	−1.10382	−2.00981
O	1.19536	1.04567	−0.17322
P	2.58927	1.06690	−1.23882
P	1.29906	−1.16406	−3.20265
P	−2.09244	0.91534	2.68698
P	−3.36621	−1.34554	0.75244
O	1.82224	0.43335	−2.77271
O	−3.24886	0.29521	1.55098
O	2.92472	2.60363	−1.62558
O	3.74857	0.06332	−0.71992
O	2.22876	−2.37932	−2.69734
O	0.64180	−1.11377	−4.67420
O	−1.58488	−0.11066	3.82082
O	−2.44420	2.45912	2.99219
O	−3.10631	−2.52674	1.82884
O	−4.76835	−1.24057	−0.05205
Cl	−1.61209	1.74518	−1.61360
H	−0.85306	−1.83471	−2.06151
H	0.13163	1.46324	1.61923
Cl	0.70746	−1.81565	0.93316
H	−3.14404	2.50477	3.64773

H	1.33628	−1.14094	−5.33645
H	4.49530	0.09758	−1.32226
H	−3.84438	−2.57038	2.44117

10.1.4 Figure 5, Article 3.2

32
tri−TcPyp; multiplicity=2

Re	1.19000	0.57528	−0.22032
O	0.37833	2.47662	−0.20796
O	2.06802	1.35901	1.47280
O	1.61152	−1.05958	0.82965
O	0.79862	−0.92947	−1.70064
O	0.49475	3.17916	2.23118
O	1.48093	−3.09266	−0.62250
P	0.92245	−2.48088	0.77816
P	1.81923	−2.09023	−1.88167
P	−0.48356	3.09340	0.95769
P	1.50516	1.98090	2.78320
O	−0.67478	4.62218	0.53664
O	−1.75883	2.36998	1.23819
O	0.46955	0.85552	3.31548
O	2.43030	2.51505	3.79004
O	3.26381	−1.70727	−1.92397
O	1.33498	−2.93513	−3.14388
O	−0.55765	−2.45254	0.91834
O	1.68333	−3.40748	1.82437
O	3.26072	0.62676	−0.63981
O	−0.88275	0.11743	0.14780
O	1.13770	1.48470	−2.22900
H	−1.56950	4.93296	0.72104
H	0.18082	1.02445	4.22014
H	2.07667	−3.30199	−3.64097
H	1.15044	−3.58001	2.61058
H	3.54016	−0.20620	−1.11582
H	−1.02845	−0.81279	0.45335
H	0.74705	0.82475	−2.82378
H	0.57737	2.27842	−2.18347
H	−1.39619	0.76727	0.69422
H	3.75145	0.73521	0.19142

26
mono−TcPYP; multiplicity=2

P	56.02420	59.66772	10.29023
P	54.72282	57.02209	10.59584
P	56.66998	55.72416	5.32032
P	56.32118	58.57773	5.16465
O	56.00806	59.50515	8.74464
O	56.07710	55.95338	6.74494
O	57.42119	58.37358	6.33414
O	54.87756	58.71815	10.90037
O	56.32396	57.06403	4.46835
O	57.34940	59.35686	10.94734
O	53.52547	56.55293	11.30546
O	58.14364	55.43056	5.30230
O	55.04319	58.53215	6.12680
O	55.56679	61.15204	10.64508
O	56.13376	56.50017	10.99705
O	55.79816	54.58999	4.61938
O	56.48364	59.63239	4.16418

O	54.67475	57.02464	9.00367
Tc	55.97877	57.74299	7.71322
H	56.34405	53.95645	4.13881
H	54.77616	61.43776	10.16902
O	57.60956	57.12448	9.06189
H	57.09862	56.66022	9.84430
H	57.92439	57.93717	9.50542
Na	59.15288	56.93301	6.57752
Na	57.05462	57.79657	12.54236

10.1.5 Figure 2, SI 1.3

29
Sn(IV)PYP2Aq2

Sn	−0.54656	−0.19840	−0.32111
O	−1.35873	1.10895	0.96152
O	−0.99049	−1.62348	1.01354
O	−2.08560	−0.34368	2.89199
P	−2.64273	0.75645	1.79706
P	−0.87596	−1.38972	2.55782
O	−3.00746	2.00042	2.71102
O	−3.77953	0.26608	0.98012
O	−1.38671	−2.70000	3.29951
O	0.47122	−0.90616	2.98352
O	−2.41705	−0.48136	−1.23961
O	1.32703	0.08335	0.60283
H	−2.28746	2.30377	3.27893
H	−3.18771	−0.23017	−0.66731
H	2.09578	−0.17676	0.03193
H	1.36659	−0.25438	1.53434
H	−2.46462	−0.15430	−2.17340
O	−0.09294	1.22731	−1.65548
O	0.27914	−1.50201	−1.60068
O	1.02489	−0.04837	−3.52716
P	−0.21700	0.98574	−3.20263
P	1.56524	−1.15131	−2.43090
O	−1.54002	0.46749	−3.63166
O	0.22018	2.31057	−3.95764
O	2.69929	−0.65586	−1.60948
O	1.93948	−2.39981	−3.33505
H	1.22204	−2.71143	−3.90207
H	−0.68042	−3.14201	3.78657
H	1.09855	2.63479	−3.71984

10.1.6 Figure 3, SI 1.3

29
Re_Sn_analog; multiplicity=2

Re	−0.54291	−0.21588	−0.31960
O	−1.27409	1.14544	1.00813
O	−0.99917	−1.67058	0.97644
O	−2.10251	−0.37394	2.82969
P	−2.58402	0.80904	1.79512
P	−0.89258	−1.42809	2.53200
O	−2.86007	2.02061	2.79021
O	−3.75219	0.41347	0.95469
O	−1.43312	−2.74580	3.24162
O	0.45103	−0.97177	2.98756
O	−2.39646	−0.36937	−1.21619
O	1.31061	−0.06433	0.57810

```
H    −3.78816     2.28270     2.79681
H    −3.17355    −0.11470    −0.65428
H     2.08938    −0.31369     0.01610
H     1.36159    −0.29760     1.53775
H    −2.44672    −0.13397    −2.17544
O    −0.09111     1.24246    −1.61125
O     0.19187    −1.57307    −1.65261
O     1.01729    −0.04276    −3.46821
P    −0.19586     1.00518    −3.16825
P     1.50149    −1.22849    −2.43547
O    −1.53948     0.54809    −3.62337
O     0.34178     2.32538    −3.87561
O     2.66777    −0.82706    −1.59534
O     1.78298    −2.43928    −3.42988
H     2.72231    −2.65706    −3.48246
H    −0.83146    −3.06382     3.92655
H    −0.27923     2.66542    −4.53170
```

10.2 Structure ensembles from conformational search

10.2.1 Table 2, Article 4.3

```
29
converged
Re   −0.164626334550043   −0.0329259767947932   −0.075260463671478
O    −1.35203480290168    1.24944750952759    1.03732889438911
O    −0.931106698107183   −1.51505323991271    0.729278516372309
O    0.651513853239346    1.40016243207123    −0.932811972071571
O    1.0639067033824  −1.28285743259601   −1.22939864410516
O    −1.34189507869024    −0.396034677710343    2.91650610731486
O    1.58178394445119    0.432777501198895    −3.00152106659501
P    0.687726104643543    1.77685479840531    −2.46274769186404
P    1.00054045632581    −1.02380148721513    −2.72630570826773
P    −2.28497599378623    0.532289786322457    2.00005546551046
P    −0.861378829341422    −1.84744846887218    2.29475673474437
O    −2.81480447653516    1.60157636836681    3.02453805513003
O    −3.39627553591827    −0.254744208248664    1.43239737856756
O    −1.92568475097036    −2.82992219605598    2.59661165825094
O    0.565326199783572    −2.0155946776923    2.68286050538314
O    2.06200292439164    −1.95558911030859    −3.42770249837275
O    −0.32007913585984    −1.1879324256031    −3.39267640331845
O    −0.705775733213942    1.53351908969333    −3.01099340841906
O    1.43467370523753    2.96262794704562    −2.84393302648937
O    −1.88235481793246    −0.14641335268795    −1.42741415809375
O    1.49226640485158    −0.0893895112832943    1.32850417240268
H    −2.14061202480476    2.24652103648273    3.23648890654674
H    −1.7307211335104    0.685300837143973    −1.94737845656293
H    1.22813978374235    −0.833951075172803    1.99394684553044
H    2.26491690800922    −0.429158345147696    0.857922970705115
H    −1.61883981835176    −0.848348868249174    −2.0530919473948
H    2.86911684645194    −2.0390782559493    −2.9218303628848
Na   −0.618833992609479    0.369719356277537    −4.48153509290622
Na   −3.43641465634112    −2.10035334171649    1.65110468570638
29
converged
Re   0.00539046947091343    −0.0134505580621439    −0.176737782930064
O    −1.09523089593844    1.38004375494965    0.898322216204869
O    −0.594820855425943    −1.50287727661084    0.754368181854362
O    1.4088955373442  1.39906407941943   −0.697729423541224
O    1.0773533743729  −1.34514494537857   −1.41829327792715
```

O −1.41367728581705 −0.224681943417603 2.77922033526593
O 1.58413633893278 0.519982264111682 −3.0418209591506
P 0.850222384029819 1.70912874522484 −2.08945976811884
P 0.964262400503952 −0.949559085617791 −2.87618602761028
P −2.17739285735913 0.702394675338044 1.71487913206524
P −0.883912145029637 −1.71360887123204 2.3233229251635
O −2.84806990520696 1.79501672029872 2.62638249405104
O −3.19836145306637 −0.0725704742522901 0.974709164785045
O −2.03410711870291 −2.64050288019654 2.42105952065322
O 0.434025098580458 −1.90426851673604 2.97547743841761
O 1.94593994385454 −1.85240491667688 −3.70971472142511
O −0.390603767708808 −0.969536193736675 −3.50619054961076
O −0.635481487344758 1.23140025794166 −2.01296283755259
O 1.068633680093 2.97688876740181 −2.77558395212738
O −2.02644415331235 −0.984243886707741 −1.32883194418571
O 1.54503199928939 −0.152869238779833 1.44946625820926
H −2.19495805523574 2.39762356754143 2.98090234362537
H −2.54419534508463 −0.175415069759182 −1.41405998217413
H 1.22220946284768 −0.821438152888826 2.15190359160748
H 2.34212437685432 −0.521854505416108 1.04899962684767
H −1.5798933885317 −1.16494995981535 −2.1820812821706
H 2.74918124702929 −2.05198142345405 −3.23094409977554
Na −0.925047750180228 0.85830914535301 −3.90269352531506
Na −3.27970983875635 −1.94629406223105 1.11197689510972
29
converged
Re 0.0569584781181737 0.0234533065747978 −0.192577498221035
O −1.03948296051882 1.43787857922558 0.852522207841267
O −0.572453738202166 −1.45519018160415 0.730272126728278
O 1.4938226548699 1.36774043055336 −0.768307915846469
O 1.08805868631118 −1.35698045493199 −1.41656622823371
O −1.26938318164313 −0.152733864916379 2.78269030655247
O 1.60303657560592 0.449708224325605 −3.1005212096727
P 0.924351223220011 1.67945360207825 −2.15572965398475
P 0.947486383807686 −0.997044958263577 −2.88181035211996
P −2.05625140329104 0.801149745342285 1.76155801583008
P −0.828277609993672 −1.66311168844808 2.30656490389162
O −2.59234606272036 1.94961455041669 2.69661437232645
O −3.16763937750257 0.0428205801270856 1.12142185814201
O −2.02214165486254 −2.53098491540749 2.43221944360123
O 0.49277744063825 −1.92712888124905 2.9233607027513
O 1.88280132344447 −1.94777859727536 −3.71521152359333
O −0.423455309729762 −0.997796522627097 −3.4760512226982
O −0.574308412111663 1.25100356637475 −2.04911951442317
O 1.17598964796718 2.92651060754325 −2.8666137269349
O −2.07176401805489 −0.876267788223035 −1.30318081115384
O 1.59333735248504 −0.133683760494035 1.43097772075048
H −3.3435211530942 1.65212128274805 3.20720209979821
H −2.51944628557677 −0.0306913257237261 −1.41831482359624
H 1.28304534120815 −0.819292195645576 2.11911696384302
H 2.40331340032441 −0.471839897894999 1.02901754628975
H −1.62398523831366 −1.10648089955618 −2.1433494648147
H 2.69313245883089 −2.15521111721967 −3.25186494062046
Na −0.902787569925878 0.838174307674016 −3.92784404658068
Na −3.2553669649126 −1.83521171689258 1.12122465439324
29
converged
Re −0.0567642303542282 −0.0979690656908457 −0.0456649454175852
O −1.40716588840585 1.20088665283875 0.868515161443753
O −0.787231197130844 −1.54868541213044 0.823727117691205
O 0.691131912703135 1.37933706159692 −0.943940447767006

O 1.33460868638742 -1.24362893139125 -1.02917125458249
O -1.40113614479451 -0.277956816025846 2.87806780701661
O 1.39495922946841 0.243154421664124 -3.04657678535672
P 0.589037538146622 1.5332070700247 -2.48263593142325
P 1.10605733849762 -1.25726508045432 -2.53662140145615
P -2.34830756696909 0.531222981884063 1.86001177605516
P -0.834646513598138 -1.7563418182857 2.42030919656932
O -2.94564635366851 1.66407822594206 2.77600142018853
O -3.41368445055602 -0.333267320342682 1.32218834207022
O -1.89677923016391 -2.74834415749376 2.69536778734169
O 0.561453617923685 -1.83898749228112 2.92122201353131
O 2.33067714597088 -2.02886355956708 -3.16769414124912
O -0.193337992631022 -1.77016921101689 -3.02050300717023
O -0.861005980093492 1.42877250116031 -2.93295798870116
O 1.20621358120866 2.7433293956686 -3.07267412488494
O -1.72474441908271 -0.452200481737762 -1.40157896749928
O 1.55299624702843 -0.0217532007215274 1.40940408603103
H -2.27722740258987 2.30461056350756 3.01716802271879
H -1.70347814206471 0.363130027443998 -1.96848472655214
H 2.3101699989327 -0.4285492079924 0.963252355175946
H 1.27280184007852 -0.689991072066217 2.13046570807357
H -1.357777254178 -1.13572501714856 -2.02666099677021
H 2.02340227043664 -2.61142165477981 -3.85958856905664
Na -0.51346828415127 2.99701479899386 -4.13815538242087
Na -3.27560834584829 -2.18542417969623 1.43490788781299
29
converged
Re -0.598440656710177 -0.188374512526925 -0.361451470528552
O -1.93077812334331 1.010396311756 0.68890846157014
O -0.855209585739241 -1.58578263773056 0.812811868421558
O -0.319151405420918 1.18066926885578 -1.63991092341929
O 0.738287874961102 -1.30547141984176 -1.48438275151551
O -1.14392737351781 -0.128144219028675 2.78729695488272
O 1.02371464450534 0.195564612806238 -3.47357669761481
P 1.03277679120601 1.38511021224896 -2.38175449327285
P 0.446709694743795 -1.2364994020053 -2.98403486491718
P -2.43602499139699 0.357617965109285 1.970840445998
P -0.446699349440252 -1.57483758424136 2.36937920557786
O -3.00691325198296 1.50480746308072 2.88720014063795
O -3.42228319706516 -0.72711784557802 1.80678317789615
O -1.22959318839354 -2.64118214342482 3.03280084293983
O 1.01859149610242 -1.40324695438056 2.49624006337822
O 1.42277386931913 -2.25140242183022 -3.69130817746622
O -0.956572636224011 -1.39648271118863 -3.40966606569756
O 1.18898180483772 2.69644078759323 -3.05492213809201
O 2.22145355419786 1.14176548762539 -1.46341584368742
O -2.37491413379515 -0.939787974355193 -1.36609696531008
O 1.09003212051477 0.513315975299862 0.758750771889036
H -2.42823434859534 2.26624207997218 2.89762560503972
H -2.98318972120573 -0.200720716301127 -1.48519290278545
H 1.79377752260624 0.554742289017387 0.0671046334403669
H 1.29977679413797 -0.201802677055674 1.43535097152473
H -1.99911066201724 -1.19122773053385 -2.27177953706598
H 2.26067166350793 -2.31585037920489 -3.23341059548794
Na 2.91859105896652 2.93539945211927 -2.02775565467087
Na -2.94959627013205 -2.49194058081309 2.16526593916425
29
converged
Re -0.0550582126698129 -0.121368111546902 -0.108196253975985
O -1.31548107761374 1.24362436302908 0.817263923861177
O -0.819835011816923 -1.54102213889874 0.77989182179362

O	0.763129373754609	1.29808934226148	−1.03826610551206
O	1.24404663270729	−1.34481667049056	−1.14654811685165
O	−1.19667421610891	−0.204421471879535	2.86000729857454
O	1.33185453334041	0.135390237030209	−3.15755722993121
P	0.600806511652114	1.46132952245515	−2.57135822649151
P	0.947255236281115	−1.35477120109813	−2.65408784805122
P	−2.16153054872476	0.682323489556239	1.93558718097923
P	−0.814614566908023	−1.7340159980294	2.37971670243015
O	−2.52625870300902	1.89901567542601	2.86958940375205
O	−3.36821650858717	−0.0964109243729446	1.55988195459863
O	−1.96473764870613	−2.60624027285072	2.70479056308212
O	0.586678301976575	−1.95833745248094	2.81645459581303
O	2.0817405192903	−2.20485349360735	−3.34396724232296
O	−0.401610476060625	−1.76190967273373	−3.06975464461464
O	−0.86856948979787	1.42099556708394	−2.96035065492292
O	1.25449634376596	2.63453969271653	−3.19593050725073
O	−1.81124414106774	−0.38602876222326	−1.38764164455673
O	1.60288271227613	−0.139121546680863	1.30442289198039
H	−3.29346495282028	1.69079655622693	3.40040072512083
H	−1.75366478411052	0.426588638834407	−1.95633239017362
H	2.33638034859633	−0.571384466385486	0.844791748918047
H	1.31419988877899	−0.800603968453639	2.02561083839428
H	−1.50626831031728	−1.08725165303751	−2.02472800659916
H	2.95727961309541	−1.98565481936218	−3.0264326349312
Na	−0.485003980376088	2.92360015634518	−4.23547471319893
Na	−3.30301734985924	−1.95588061609786	1.42591657620702

29

converged

Re	−0.588742908836935	−0.19180035326237	−0.404299420156377
O	−1.95710288997825	0.998726396070998	0.596168445581135
O	−0.868248249398876	−1.56774548707224	0.793902359072535
O	−0.297505442496233	1.13932762657582	−1.71258426656514
O	0.772904045762907	−1.32273371617334	−1.48168818769722
O	−1.01736272418698	−0.00889378849947362	2.71823907865621
O	1.0671530872884	0.129360070673964	−3.50877780649833
P	1.05947982385421	1.34515150314445	−2.44956300788035
P	0.493829099805747	−1.29394374577756	−2.98581642790487
P	−2.33671169000072	0.505732514928027	1.97713672726198
P	−0.425431228300829	−1.51326440872218	2.34294668837693
O	−2.73057488436097	1.78686382160348	2.80906622376694
O	−3.40214697178175	−0.527904242173368	2.02511168546612
O	−1.26567596833005	−2.50022440165194	3.0593480660495
O	1.04840524971586	−1.4297927825437	2.43976516212446
O	1.4774992400283	−2.32091022728476	−3.66202311450321
O	−0.906484630492592	−1.47317487998219	−3.4131669950621
O	1.19444709491764	2.65181545834504	−3.13471702171921
O	2.23620945500238	1.1419933644032	−1.50765864000115
O	−2.37018189641723	−0.93961462386171	−1.41914275410347
O	1.10448013707827	0.510240633831226	0.706826449215468
H	−3.27253102351221	1.54359672812938	3.55786881043797
H	−2.93509003707558	−0.175217189659779	−1.58476327353934
H	1.80402578804548	0.552004804524602	0.00973000000503704
H	1.32491347911367	−0.204245740864151	1.37694340684512
H	−1.97202480886021	−1.22720231007304	−2.30470512198403
H	2.32775390965383	−2.34436058782323	−3.22324016030508
Na	2.77037445918925	3.03586546060021	−1.90583493190188
Na	−2.9601595260914	−2.3374498860864	2.13262803308293

29

converged

Re	−0.262235817313558	0.0119304418100487	−0.0917295661562843
O	−1.55281348705571	1.21706904800941	1.01353212631057

O	−0.804835001351571	−1.5016859257242	0.813010962770863
O	0.31764316189986	1.56330399137707	−1.04472018290386
O	0.965218247363064	−1.07716564596917	−1.27679969873101
O	−1.29543208261328	−0.322120694642395	2.95616568358869
O	−0.142595269432722	0.230174348095278	−3.10717800365258
P	0.801472105380341	1.4518535428847	−2.50450430614234
P	0.373852873450599	−1.24821263527247	−2.68299840831777
P	−2.37136065854781	0.445886290783917	2.03935629305499
P	−0.675531531276944	−1.74234187326398	2.3962381548212
O	−2.98973258632661	1.49472688592131	3.03479262691322
O	−3.39995821038783	−0.485339013661255	1.53887571891391
O	−1.62232749386356	−2.81663013390379	2.76150798890379
O	0.772700566329597	−1.72850782314345	2.73928631267963
O	1.60871092774595	−1.49970079100188	−3.63384544145808
O	−0.764546859388936	−2.16708792234521	−2.82414132434606
O	0.581622003183674	2.599299382092	−3.41660385465336
O	2.21412498184271	0.923024510112734	−2.62320000452365
O	−2.09900638220489	−0.449291928777223	−1.27316075476142
O	1.48644850876916	0.121244118041731	1.15119688789558
H	−2.37053989471857	2.19806277693141	3.22822770148067
H	−1.80090750192485	−1.17896383659256	−1.88357565022541
H	2.20750303022143	−0.229175670663064	0.607241223543589
H	1.32400121859335	−0.579224866712625	1.89385832752104
H	−2.36861039716575	0.293802275531576	−1.82699499779769
H	2.28747219827819	−0.905718313142701	−3.28014401831007
Na	2.12703363355572	1.67351441522172	−4.53639932701765
Na	−3.17187028841414	−2.33052494596976	1.68040551555301
29			
converged			
Re	−0.562135885138158	−0.172813879843795	−0.355555349166416
O	−2.00622447831744	0.954265835202293	0.607074265067418
O	−0.831212387673859	−1.55778232246458	0.834756303885414
O	−0.306415828335676	1.18658327629692	−1.65773719362861
O	0.87964779841589	−1.24879851805542	−1.37240637066259
O	−1.05592144200776	0.000592549409300036	2.75142886310336
O	1.11976786326629	0.189368632243406	−3.41254677140056
P	1.05457831894011	1.42726227456439	−2.36157162037803
P	0.646105938531613	−1.24841478420489	−2.87437742131595
P	−2.38023456874723	0.478386157574914	1.99654286953179
P	−0.419981946086944	−1.49042272741701	2.3941741567325
O	−2.80427345953823	1.76464579176974	2.80492267245117
O	−3.42594401490776	−0.575092070012808	2.05229397141704
O	−1.25369012817805	−2.49589382345052	3.09327858998738
O	1.04767012186273	−1.37518251460896	2.52153758926875
O	1.7161307651735	−2.20673639857664	−3.53001583209212
O	−0.730899047593439	−1.5551482261663	−3.34019988999964
O	1.18903064522948	2.69277647275195	−3.12053160473873
O	2.22921204256159	1.27202750181651	−1.41219189811756
O	−2.28625765966084	−0.972957191761972	−1.43633012641961
O	1.07349517960938	0.579488392524665	0.787309093879688
H	−3.32940557467393	1.52195504593601	3.56590041604004
H	−2.85213683542223	−0.220183439855695	−1.64507426978431
H	1.78272756355613	0.679716148502229	0.104811890736205
H	1.32295559915384	−0.132435671289267	1.4449664642621
H	−1.83639810273151	−1.27124752116888	−2.29551985819681
H	1.29013349663963	−2.77623769811932	−4.16797629305885
Na	3.03690712070269	2.88630509077859	−2.26578341308346
Na	−2.93173110529468	−2.37182638163945	2.13652078238453
29			
converged			
Re	−0.749796867836553	−0.206563602110875	−0.37221712528438

O −2.0203774139308 0.758104279166991 0.683857747634441
O −1.04437137613306 −1.78517078268409 0.811083517946281
O −0.51663165377281 1.37810368209313 −1.68030703424983
O 0.515469496733745 −1.15226912988432 −1.43330719855114
O −0.989012104427099 −0.20410531066015 2.76015610141832
O 0.962109906147164 0.279921093927654 −3.41716357889723
P 0.868704306439779 1.51256526555703 −2.2890509627395
P 0.466194997824377 −1.15847158939857 −2.98348239286653
P −2.33937753163081 0.304740719020856 2.13615174003858
P −0.421982855500331 −1.7409005543885 2.26225606492073
O −2.67401458662867 1.60018661076659 2.96395122053953
O −3.4317676402417 −0.678394213183263 2.18966641086767
O −1.16692955012562 −2.71608866381153 3.09345217611065
O 1.05440586106099 −1.61282690060579 2.25161261735333
O 1.62079090526194 −2.12514860342532 −3.45309974750227
O −0.851455145813789 −1.50385297782425 −3.54299867659979
O 1.18362956843617 2.74299066583053 −3.05264425358868
O 2.0313122173252 1.18394457155264 −1.35216958212556
O −2.42885377363106 −0.778733879869981 −1.64706710121268
O 0.845025128426703 0.508694454697826 0.828246551306441
H −1.96957449445024 2.24735423911398 2.93254640505888
H −1.97653889600263 −1.18357514593719 −2.44758086815859
H 1.52572250709737 0.75570206264297 0.144830035506526
H 1.16565141090669 −0.265081449233202 1.39644058004243
H −2.78040183107349 0.0654177308845608 −1.95916534200962
H 2.33729732986611 −2.17463631165267 −2.81981438661469
Na 3.12945866875365 2.40872394457063 −2.56224649701928
Na −2.96918656199544 −2.49843020442026 2.40576356892122
29
converged
Re −0.227462568861924 0.0362118997818608 −0.115618156208119
O −1.50539717232369 1.27187902375229 0.960855295380325
O −0.799296180040453 −1.45936526218704 0.799879750329367
O 0.397718890367972 1.55346507784912 −1.0897777184776
O 0.969223285699652 −1.09876493504149 −1.29390661666428
O −1.14917072253998 −0.216265476843562 2.94351079700472
O −0.129723588485535 0.213045197458452 −3.12794193731187
P 0.85304226388147 1.41759443706494 −2.5556457546321
P 0.354568773856769 −1.2717958039245 −2.69027129938046
P −2.24221823026265 0.588361261008983 2.08959568386829
P −0.647228826102948 −1.68315415249975 2.3866594272396
O −2.70201023012428 1.72593428524769 3.07580551264957
O −3.36646324577833 −0.311793019974971 1.72583080790084
O −1.66406113867124 −2.68160624099173 2.78206621643778
O 0.804565231271075 −1.76584668696601 2.69527355702544
O 1.56619172283761 −1.57294253965143 −3.65459675991225
O −0.813063725245781 −2.15860618656661 −2.79903079726265
O 0.650341314781772 2.56125785789981 −3.47575866556246
O 2.24994570473137 0.850154401362023 −2.69082426285599
O −2.10171928429798 −0.396256813330935 −1.27470552837421
O 1.5343111869977 0.104500483279162 1.1145736891234
H −3.42134501224343 1.42258775915591 3.62747975002259
H −2.33916718956691 0.353585271773038 −1.83433376823146
H 2.24727109036314 −0.250985625183156 0.563004628766657
H 1.37171899122092 −0.594175736028822 1.85208499843963
H −1.81561008149073 −1.1348718200574 −1.88228513331374
H 2.26414226426805 −0.987874910867675 −3.32381794180419
Na 2.19247811181804 1.61209749309869 −4.58299384302166
Na −3.15608164672454 −2.16416924317325 1.62258806436184
29
converged

Re	−0.134800398621161	−0.363952104384566	−0.151875254296959
O	−1.13949002031538	1.2133004508415	0.775864867631385
O	−1.06154902733397	−1.60903599211148	0.843931273675878
O	0.746496894504426	0.92842957795756	−1.20126412856655
O	0.909690124171854	−1.78049696355703	−1.20818608191019
O	−1.11885286300395	−0.164979795048514	2.87609629249261
O	0.165028361408873	−0.467708330388507	−3.20046072990171
P	0.312405623306827	1.05199667883	−2.68571679775975
P	1.3082824440664	−1.54384633503469	−2.67504508903612
P	−2.00004458179425	0.799971963707959	1.94309645442597
P	−0.951395732921224	−1.74673728372838	2.44976042553205
O	−2.18326308835657	2.08410949660574	2.83842184621642
O	−3.30398465510706	0.155413100005136	1.6395419874401
O	−2.16407815347244	−2.46076648098782	2.90536206953269
O	0.442237439969154	−2.12397218092406	2.7936580051458
O	2.61143960529291	−0.629866002346046	−2.62762946573123
O	1.37500694307848	−2.69951290707409	−3.54877125632232
O	−1.05988933474675	1.6717951941476	−2.81987815183022
O	1.32254263062102	1.77675963809677	−3.51496572905977
O	−1.94502102230407	−0.386924362847286	−1.42539628328528
O	1.57734123215648	−0.549430754589876	1.13385044746089
H	−2.93065329996058	1.98104882577971	3.42523715702397
H	−1.87340200179064	0.482604037184114	−1.90662011085255
H	2.21373088628551	−1.08614546916073	0.634719509479955
H	1.26751617832421	−1.14383175300777	1.90838418146732
H	−1.78189989978654	−1.06159299075835	−2.099689416486
H	2.46739757613965	0.255727741346679	−2.98580830173274
Na	−0.176704410667503	3.07011912257084	−4.01696577128443
Na	−3.51858745451679	−1.69027612568077	1.74804805665134

29

Coordinates from ORCA−job Tc_Fiz_d E −82.465740048330

Tc	−0.15179361171595	−0.03483498299020	−0.06963118337664
O	−1.31890524294496	1.21622733463803	1.05665789172236
O	−0.88666250402193	−1.48432348896279	0.75252572638245
O	0.60520199422398	1.37294665178031	−0.95026965429153
O	1.04958448461296	−1.26270248167025	−1.23232934684941
O	−1.37203659809518	−0.40748689125057	2.94555778005632
O	1.57222297846254	0.43663768677274	−3.01319048449990
P	0.67165181888221	1.77230524419402	−2.47613459917876
P	0.99341820741857	−1.02131954643597	−2.73120060113112
P	−2.28492030414969	0.53018700193661	2.00515930459506
P	−0.85847311305992	−1.84093587989993	2.31864559622565
O	−2.81658309805767	1.61428201751903	3.01097252378164
O	−3.39310230715088	−0.24966162930796	1.42195001199546
O	−1.92095400067939	−2.83838131425211	2.57069470051507
O	0.56055527527547	−2.00093989575065	2.73378818950083
O	2.05939625936573	−1.95425036976892	−3.42217084069147
O	−0.32603790330586	−1.18807514871608	−3.39927788906208
O	−0.71416448571950	1.53506418170349	−3.04533107304937
O	1.42446547914672	2.96432161135633	−2.82142726666332
O	−1.86882451356624	−0.12982973846292	−1.42745583165392
O	1.50354969850468	−0.11238981621400	1.32699526212181
H	−2.13653872867210	2.24999022232675	3.23290913632755
H	−1.72611876606681	0.69894313720246	−1.95452775859113
H	1.23435545494801	−0.83096215214984	2.01387794787850
H	2.27300813825278	−0.47428197789641	0.86881624072578
H	−1.63002742269963	−0.83659753593514	−2.05689066197130
H	2.87060088318641	−2.02340636343502	−2.92035526791408
Na	−0.61714830842269	0.35972666951337	−4.50635589730841
Na	−3.42021974286591	−2.09805253452541	1.61569803994096

29

Coordinates from ORCA–job Tc_Fiz_d_job10 E −82.434660472050

Tc	−0.57155439920158	−0.18171911005082	−0.35122190728460
O	−1.96398310047030	0.94912189778960	0.63130010215595
O	−0.82555503047771	−1.53054085895984	0.83097825725011
O	−0.30278166007428	1.15087268345314	−1.62668065723964
O	0.83534393205260	−1.24714916091948	−1.38265364899123
O	−1.05164432212871	−0.01204753575142	2.78292681446442
O	1.10541795641644	0.19518685045738	−3.40595428225327
P	1.04267593625264	1.42600063638651	−2.34810547756333
P	0.63661592915369	−1.25322827943693	−2.88958805598408
P	−2.36114172329180	0.49552423075203	2.01916704167243
P	−0.42614338026573	−1.49869235516938	2.40076775576355
O	−2.76533588871464	1.79002296163356	2.82092816238578
O	−3.42441000800034	−0.54080558044754	2.06450240121296
O	−1.27753578344937	−2.51193494762886	3.06556773044921
O	1.04087106542596	−1.40158318348996	2.53278993395269
O	1.73725410453620	−2.19248889330033	−3.52093929148526
O	−0.72570264667974	−1.57754627894834	−3.38373877521097
O	1.14121054115384	2.69718965873826	−3.10147159655493
O	2.22842525577000	1.28670858408294	−1.41162296875892
O	−2.27573697356975	−0.98664453919502	−1.46781865358179
O	1.07420188590017	0.56974449580043	0.79336545458275
H	−3.30896123023179	1.55897810676304	3.57276222740929
H	−2.86135879779845	−0.25492885574767	−1.69419436564591
H	1.78101613227237	0.68354575702048	0.11195229140117
H	1.33438471483228	−0.13709880689062	1.44748819274804
H	−1.82704251765834	−1.29558999172528	−2.32279734647884
H	1.33006803359736	−2.79055876350101	−4.14522060941599
Na	3.00425435047728	2.91522938259229	−2.27642735005112
Na	−2.94735238649318	−2.34336810357177	2.10163863775569

29

Coordinates from ORCA–job Tc_Fiz_d_job11 E −82.434201057500

Tc	−0.76915315926725	−0.21907947524542	−0.37502462925896
O	−1.99701387457968	0.71530790095923	0.67553324547936
O	−1.05507342514838	−1.78054321430597	0.80682126705028
O	−0.50487414756273	1.35161016391576	−1.67014776314090
O	0.46052678548255	−1.13290126725939	−1.43526899153602
O	−0.98098324933673	−0.20892608317567	2.76974552682174
O	0.99649204560190	0.28153482830481	−3.39689142636658
P	0.87751298895819	1.52061129126634	−2.27533124228067
P	0.46486346794045	−1.15021787155909	−2.98807618569376
P	−2.32045580266971	0.31790532357942	2.14443751725892
P	−0.43094343680080	−1.75442887327989	2.26057958219067
O	−2.62153633599481	1.64289897624572	2.93511603204514
O	−3.43441318763190	−0.63892904850539	2.21561246467638
O	−1.19466906569041	−2.72055904632242	3.08475549262548
O	1.04502868177162	−1.64027456767160	2.25212752954305
O	1.61890229948660	−2.13411123868249	−3.41765008698650
O	−0.84023240432986	−1.48188380892915	−3.58152076973459
O	1.16221266637150	2.75446485766065	−3.04435659293944
O	2.04633500779624	1.22156004801431	−1.33602169243368
O	−2.43732272418704	−0.83547687789050	−1.65771788968671
O	0.84135522473028	0.48547187037329	0.81406932097843
H	−1.88967461116849	2.25989803267240	2.91736334493336
H	−1.98155029222346	−1.21678926533277	−2.46444966226962
H	1.51920747572186	0.75508263211320	0.13502201252432
H	1.17150535875856	−0.28158777519296	1.37980163477310
H	−2.84425015206900	−0.01571163106323	−1.96544693614377
H	2.34814173376350	−2.14774344156460	−2.79682540686469
Na	3.11735550572275	2.48491497762053	−2.53539622535780

```
Na    -2.99179335235995    -2.46989741600969     2.39684052003872
29
Coordinates from ORCA-job Tc_Fiz_d_job12 E -82.431467117300
Tc    -0.21523300271401     0.00498315923503    -0.11209790038772
O     -1.43652171783788     1.25261216144139     0.97984090393409
O     -0.76592301433972    -1.43558991756120     0.83768732513467
O      0.38766482108027     1.45422268647344    -1.12101115330675
O      0.94075880428046    -1.12903589217572    -1.30743044430484
O     -1.17282582126148    -0.21099409750494     2.98579702485901
O     -0.13420070599754     0.15711884330092    -3.17762579075459
P      0.81385854895211     1.36216866690645    -2.60431966525221
P      0.34802761689326    -1.32871881704583    -2.71204887636676
P     -2.22451559390330     0.61100264378049     2.09400533380806
P     -0.65333186630264    -1.66965507924249     2.43178178267926
O     -2.68192519430470     1.76918586212793     3.05591268355696
O     -3.35753543655386    -0.27014929576608     1.70902426043642
O     -1.68150800415666    -2.67084170856770     2.78845364113117
O      0.79206830890513    -1.75305586760171     2.76334870600062
O      1.56992454055013    -1.63145697789102    -3.65377468286392
O     -0.82399847956230    -2.20938658035587    -2.81550261365264
O      0.57441326405865     2.55169646219824    -3.45732925874813
O      2.23305513920843     0.86028056063400    -2.76496030834801
O     -2.08183195893365    -0.43441795810708    -1.26989130115046
O      1.54991087213282     0.06479022387774     1.11364964309544
H     -3.43566993570535     1.49314835005145     3.57524695671018
H     -2.36689173736753     0.30676896740395    -1.81842967440634
H      2.25742682801200    -0.32458090299512     0.57968465103609
H      1.37871793383382    -0.59824692289970     1.87567383258993
H     -1.81183930069627    -1.17028661558726    -1.88823912547487
H      2.23297830644916    -0.97261925282519    -3.39317902414921
Na     2.29053562523440     2.00755171619828    -4.44848729253942
Na    -3.15008885061859    -2.12429442205876     1.60192036227097
29
Coordinates from ORCA-job Tc_Fiz_d_job13 E -82.425984228950
Tc    -0.11908390836688    -0.36840888013849    -0.13973554043823
O     -1.12134432482199     1.17343896126118     0.81236596815898
O     -1.00184490010929    -1.59001167559580     0.86467027417851
O      0.67706610903106     0.92899410836761    -1.20064245039449
O      0.94271831137410    -1.71390501138467    -1.20660691595527
O     -1.15072528410548    -0.18089760758008     2.92148139367649
O      0.16024832044457    -0.46549653162462    -3.22259973723900
P      0.29772525265368     1.05162945854124    -2.70154381726870
P      1.30510006484826    -1.53881249194241    -2.69360212472106
P     -2.00697295785869     0.78992443872458     1.96742703294909
P     -0.94360254175244    -1.74812652203178     2.47607095267513
O     -2.18940251649654     2.08038068373668     2.85129733148346
O     -3.31128556236063     0.15377684059960     1.64698317425609
O     -2.15766974683745    -2.49067882891783     2.87673257073533
O      0.44721256926431    -2.11081930848811     2.84463574902674
O      2.61827904760910    -0.64405425258187    -2.71442516087929
O      1.33200357942185    -2.72787792351785    -3.52284103483531
O     -1.06445480107570     1.68049540454940    -2.87520265622433
O      1.34282257465248     1.77123357410299    -3.49187631483563
O     -1.93391994119384    -0.36472093429376    -1.42396957175961
O      1.58301866782431    -0.56704875385242     1.14857085847628
H     -2.96827307449132     2.00130933003406     3.39997600132428
H     -1.87624606518076     0.50011311771841    -1.91117183529108
H      2.21674787150262    -1.11739704533599     0.66164383059520
H      1.27575702731504    -1.13926775207345     1.93826798074740
H     -1.81144716416283    -1.04368855355373    -2.10242414935039
H      2.45499865344545     0.26242445016983    -3.01115241960469
```

```
Na   -0.13128799134859    3.07656775548580   -4.04916828887616
Na   -3.49063727459749   -1.69687605493486    1.70453890551085
29
Coordinates from ORCA-job Tc_Fiz_d_job2 E -82.465740048330
Tc   -0.15179361171595   -0.03483498299020   -0.06963118337664
O    -1.31890524294496    1.21622733463803    1.05665789172236
O    -0.88666250402193   -1.48432348896279    0.75252572638245
O     0.60520199422398    1.37294665178031   -0.95026965429153
O     1.04958448461296   -1.26270248167025   -1.23232934684941
O    -1.37203659809518   -0.40748689125057    2.94555778005632
O     1.57222297846254    0.43663768677274   -3.01319048449990
P     0.67165181888221    1.77230524419402   -2.47613459917876
P     0.99341820741857   -1.02131954643597   -2.73120060113112
P    -2.28492030414969    0.53018700193661    2.00515930459506
P    -0.85847311305992   -1.84093587989993    2.31864559622565
O    -2.81658309805767    1.61428201751903    3.01097252378164
O    -3.39310230715088   -0.24966162930796    1.42195001199546
O    -1.92095400067939   -2.83838131425211    2.57069470051507
O     0.56055527527547   -2.00093989575065    2.73378818950083
O     2.05939625936573   -1.95425036976892   -3.42217084069147
O    -0.32603790330586   -1.18807514871608   -3.39927788906208
O    -0.71416448571950    1.53506418170349   -3.04533107304937
O     1.42446547914672    2.96432161135633   -2.82142726666332
O    -1.86882451356624   -0.12982973846292   -1.42745583165392
O     1.50354969850468   -0.11238981621400    1.32699526212181
H    -2.13653872867210    2.24999022232675    3.23290913632755
H    -1.72611876606681    0.69894313720246   -1.95452775859113
H     1.23435545494801   -0.83096215214984    2.01387794787850
H     2.27300813825278   -0.47428197789641    0.86881624072578
H    -1.63002742269963   -0.83659753593514   -2.05689066197130
H     2.87060088318641   -2.02340636343502   -2.92035526791408
Na   -0.61714830842269    0.35972666951337   -4.50635589730841
Na   -3.42021974286591   -2.09805253452541    1.61569803994096
29
Coordinates from ORCA-job Tc_Fiz_d_job5 E -82.442076698010
Tc   -0.04968001180605   -0.09820851613600   -0.03413031660768
O    -1.36581078226323    1.17502213323490    0.91430619131160
O    -0.74174790758882   -1.51531267644305    0.85457341648285
O     0.62446366175972    1.34914017508991   -0.96789888588634
O     1.30456572212430   -1.22494204420813   -1.04593978453814
O    -1.43394171781035   -0.29610320215551    2.92043636754302
O     1.38722746836057    0.24639848422787   -3.06683168786057
P     0.56732679238201    1.52799890521028   -2.50851043126519
P     1.09436592863196   -1.25458727910690   -2.55605835620169
P    -2.34544673023078    0.52831003222276    1.87847014561812
P    -0.83341826171228   -1.75351893849104    2.44967718522922
O    -2.94960806410413    1.66806090803759    2.77896595761117
O    -3.40437241401903   -0.32695599793531    1.31284158464085
O    -1.88840108371913   -2.76589450810455    2.67024975331753
O     0.55376327658090   -1.82762564248891    2.97440208472362
O     2.32796057262977   -2.02428980068752   -3.17017313037558
O    -0.20160553469585   -1.76946487886712   -3.04686309693149
O    -0.87230070803214    1.43050955384552   -2.99074005990824
O     1.20050525216958    2.74692433645155   -3.06022131898298
O    -1.70631218317623   -0.43198137216228   -1.41000480133045
O     1.55812011787191   -0.03618257273290    1.42315427079685
H    -2.27915552894210    2.29980144954958    3.03794500700805
H    -1.70306203439424    0.37819087263997   -1.98420558124726
H     2.31844947171779   -0.45157784873890    0.99225940304984
H     1.27685100644669   -0.68391610143407    2.15811892222566
H    -1.35826301256872   -1.12036244948946   -2.04072693376722
```

H	2.02591078752503	−2.63491536072653	−3.84011946654611
Na	−0.48875208234360	3.00772751794244	−4.18265584988419
Na	−3.24213199029129	−2.18004515664168	1.38737942318707

29

Coordinates from ORCA–job Tc_Fiz_d_job6 E −82.438378234380

Tc	−0.60674007580630	−0.19469435775306	−0.35676019234370
O	−1.88772268805554	1.00374639325044	0.71139777492404
O	−0.85841265932375	−1.55377935581522	0.80928113901102
O	−0.31773440481050	1.14407839642007	−1.61270062093093
O	0.69982364522325	−1.30039564216131	−1.48412061643446
O	−1.14725736817897	−0.14312123766535	2.81660594403946
O	1.00546289023051	0.19585439209880	−3.46427808164226
P	1.01955575914884	1.38272098122078	−2.37121345535377
P	0.44119825875203	−1.24891540629810	−2.99074101130363
P	−2.42242558181028	0.37434059602895	1.98996974394306
P	−0.45584112300832	−1.58216445312954	2.37355166320398
O	−2.97427230961729	1.53394996288175	2.89941201824688
O	−3.42664154376561	−0.69241908193704	1.81661916391193
O	−1.25309284980874	−2.65823865548774	3.00256041341878
O	1.00931926827491	−1.42592233789374	2.50304134281913
O	1.45419063436726	−2.24604602693187	−3.66959324300572
O	−0.94849033294020	−1.43545889678060	−3.44600117964453
O	1.13972955667636	2.69774773489478	−3.04257031332659
O	2.22206172131173	1.15869329215025	−1.46776155337803
O	−2.36707019738982	−0.94650937447727	−1.39864582298910
O	1.09255284113153	0.50382609988552	0.76025360523445
H	−2.37663735973166	2.28064371926148	2.92161461676681
H	−2.99228666038700	−0.22671253929282	−1.54174794228913
H	1.79555759804088	0.55742982023174	0.07049091895975
H	1.31029091398055	−0.20674680184279	1.43357093506144
H	−1.99046366167266	−1.21759685009390	−2.29688343679110
H	2.28780016933194	−2.28257213352681	−3.20029444886560
Na	2.89202014944128	2.95898085266513	−2.05273706863516
Na	−2.96897459497748	−2.46851909445890	2.13537970822180

29

Coordinates from ORCA–job Tc_Fiz_d_job7 E −82.438902763590

Tc	−0.04744700715390	−0.12505527224645	−0.09847735476619
O	−1.27744616281220	1.21132121556207	0.86501071028905
O	−0.77401557455431	−1.51025634150802	0.81087717454940
O	0.68881495417726	1.27220346881153	−1.06093358860159
O	1.20983829546393	−1.32900115291641	−1.16112949029292
O	−1.22027722179389	−0.22208775497417	2.91267234339927
O	1.32387829900776	0.13643372637654	−3.17518579506187
P	0.57738448921803	1.45540130888524	−2.59859677169513
P	0.93548639317803	−1.35527770215225	−2.67223841641299
P	−2.15672401538269	0.67609459293726	1.96669026128233
P	−0.81128983948538	−1.73385434988921	2.41152495191816
O	−2.52302435616784	1.89923972668676	2.88916954728784
O	−3.36224881135280	−0.09319742439414	1.56748821360960
O	−1.96152221711627	−2.62364002997983	2.68259520550871
O	0.58403967794539	−1.95503990080647	2.86675867910115
O	2.08160124533423	−2.20435678944664	−3.34180155953525
O	−0.40840552453851	−1.76427294823435	−3.10151338917766
O	−0.88163391860714	1.42249137683726	−3.02481763826568
O	1.25126732846886	2.63561054598168	−3.18523423995644
O	−1.78585385214533	−0.36804941280082	−1.40106741604495
O	1.60656114190207	−0.15450735906036	1.31847395227102
H	−3.31183900460578	1.70693958271440	3.39401302736334
H	−1.74305349435345	0.43894426615645	−1.97973426504124
H	2.34367804832789	−0.59613731060421	0.87544082347682
H	1.31744951555984	−0.79529969334567	2.05388147565902

H −1.50284445849138 −1.07549369970832 −2.04157953249165
H 2.95376482271533 −1.97204154579589 −3.02356346964668
Na −0.45660105264325 2.93234590693401 −4.28601110009321
Na −3.27403766313344 −1.94725702928459 1.38498766748833
29
Coordinates from ORCA−job Tc_Fiz_d_job8 E −82.435526150490
Tc −0.59787457974095 −0.19923429526430 −0.40017137377211
O −1.91433558666323 0.99137921779506 0.62076947018610
O −0.86360798082385 −1.54055684901724 0.78811273977706
O −0.29687455350297 1.10723788390816 −1.68165748642894
O 0.73030479951601 −1.32148320154884 −1.48477747237196
O −1.01201394144703 −0.02377472554992 2.74882099714238
O 1.04877032699308 0.13177560030680 −3.49762333881446
P 1.04549545777587 1.34424776869223 −2.43664052941486
P 0.48602629990833 −1.30484927111066 −2.99491097214091
P −2.31663290253638 0.52052123461630 2.00047112848173
P −0.43152461362917 −1.52295980715434 2.34707344064404
O −2.68915394415814 1.81039712058663 2.82563131232593
O −3.39972303020682 −0.49513697112681 2.03938993736356
O −1.28930862943339 −2.51803712462954 3.02980443368624
O 1.04152298685628 −1.45762482096743 2.44637319365363
O 1.50625198479793 −2.31219834062843 −3.64498546496739
O −0.90044081037458 −1.50766424277007 −3.45375579024991
O 1.14571684216129 2.65326756330625 −3.12150149048453
O 2.23376336946409 1.15903346668633 −1.50708851956766
O −2.36061287232456 −0.94937125449940 −1.45409145407074
O 1.10406423561498 0.49944366987791 0.71257429416432
H −3.24784627767922 1.58009840635853 3.56647610242381
H −2.94262558565733 −0.20427556012888 −1.64385045372928
H 1.80267893353929 0.55745982192567 0.01735990669753
H 1.33440205858059 −0.21257134721983 1.37730447838791
H −1.96112557731825 −1.25497955804579 −2.33214983541346
H 2.34812392253750 −2.31436902135353 −3.18905301920508
Na 2.74768485305591 3.05707554683750 −1.92711630887921
Na −2.97560519597016 −2.31065089856336 2.09691208069685
29
Coordinates from ORCA−job Tc_Fiz_d_job9 E −82.435462151500
Tc −0.24991039850084 −0.01397890653254 −0.08576639016400
O −1.49197669647553 1.19689175068728 1.03074548568982
O −0.77920505841320 −1.47422782946638 0.84624356474725
O 0.31284077337392 1.47391945981714 −1.06692794543892
O 0.94352309134823 −1.09569390505645 −1.28915133739657
O −1.32606847887239 −0.31867949679198 2.99290433630455
O −0.13998774968297 0.18906742066893 −3.15299302480977
P 0.76780718465083 1.41350852685471 −2.54256058600705
P 0.37667215675493 −1.28904267150503 −2.70525376268939
P −2.36044212056626 0.46811889441245 2.04141301884906
P −0.68971716694147 −1.72982015499573 2.43610226578710
O −2.97555280423316 1.54248342792387 3.00965982353744
O −3.39601465904789 −0.44647142420302 1.52416786993102
O −1.64913151761879 −2.80575449544221 2.75950688372086
O 0.75105289350656 −1.71823198221869 2.80393769340587
O 1.62470940404528 −1.53882238362997 −3.63234422785763
O −0.76258510728888 −2.20559041895570 −2.84643558448156
O 0.50638080566006 2.59698785679740 −3.39604677974711
O 2.19891121173473 0.94245627532231 −2.68844028968554
O −2.07702528396699 −0.48262600366232 −1.27221117609951
O 1.49724666223841 0.08840600598144 1.15977113295689
H −2.34168283743940 2.22819900061467 3.21901488751041
H −1.79384134699302 −1.20464022718298 −1.89807663052804
H 2.21941445261904 −0.28734722401058 0.63617682015741

H	1.32511655192016	−0.58115901460624	1.92129932035017
H	−2.39882900666308	0.25272672327938	−1.80789812598733
H	2.27246761861144	−0.88475973081939	−3.32662185635498
Na	2.18084184885719	1.94231104147115	−4.46376734731678
Na	−3.16951442798998	−2.29603050872433	1.64125194656982

29

Coordinates from ORCA−job Tc_Fiz_d E −82.465740048330

Tc	−0.15179361171595	−0.03483498299020	−0.06963118337664
O	−1.31890524294496	1.21622733463803	1.05665789172236
O	−0.88666250402193	−1.48432348896279	0.75252572638245
O	0.60520199422398	1.37294665178031	−0.95026965429153
O	1.04958448461296	−1.26270248167025	−1.23232934684941
O	−1.37203659809518	−0.40748689125057	2.94555778005632
O	1.57222297846254	0.43663768677274	−3.01319048449990
P	0.67165181888221	1.77230524419402	−2.47613459917876
P	0.99341820741857	−1.02131954643597	−2.73120060113112
P	−2.28492030414969	0.53018700193661	2.00515930459506
P	−0.85847311305992	−1.84093587989993	2.31864559622565
O	−2.81658309805767	1.61428201751903	3.01097252378164
O	−3.39310230715088	−0.24966162930796	1.42195001199546
O	−1.92095400067939	−2.83838131425211	2.57069470051507
O	0.56055527527547	−2.00093989575065	2.73378818950083
O	2.05939625936573	−1.95425036976892	−3.42217084069147
O	−0.32603790330586	−1.18807514871608	−3.39927788906208
O	−0.71416448571950	1.53506418170349	−3.04533107304937
O	1.42446547914672	2.96432161135633	−2.82142726666332
O	−1.86882451356624	−0.12982973846292	−1.42745583165392
O	1.50354969850468	−0.11238981621400	1.32699526212181
H	−2.13653872867210	2.24999022232675	3.23290913632755
H	−1.72611876606681	0.69894313720246	−1.95452775859113
H	1.23435545494801	−0.83096215214984	2.01387794787850
H	2.27300813825278	−0.47428197789641	0.86881624072578
H	−1.63002742269963	−0.83659753593514	−2.05689066197130
H	2.87060088318641	−2.02340636343502	−2.92035526791408
Na	−0.61714830842269	0.35972666951337	−4.50635589730841
Na	−3.42021974286591	−2.09805253452541	1.61569803994096

10.2.2 Table 3, Article 4.3

29

−82.9937752304 converged=true

Re	−0.48261689951241737440	−0.20176734131144288820	−0.26251017839318740998
O	−1.68138013753609993906	1.25376693681255280133	0.67141608817962905587
O	−1.01339396702486039814	−1.47099312121516345719	0.98594091449556797713
O	−1.46720167313061744530	0.08731070435799605234	2.87239605999943226067
P	−2.50371878947114545610	0.65239870532037380624	1.79758175911455908569
P	−0.77940737254917713539	−1.40173678477236518525	2.56280138606389273903
O	−3.20521653552054308634	1.82975776834730230469	2.56995170735715827703
O	−3.50042790717338903050	−0.37510245881048553951	1.40537651996193435622
O	−1.67523788065050993623	−2.38940391063435919605	3.20402161841248833696
O	0.67261712301337506226	−1.25623382723927190519	2.83548476078462918792
O	−2.34089916392006536228	−0.62763966285384553689	−1.29226796349157857513
O	1.37692010205541515155	0.14004651968638368031	0.80913318556471480747
H	−2.62758945903619522966	2.58664681410267993655	2.66132064855920180690
H	−3.06019818641735952269	−0.32917309137827188703	−0.71156176295626527573
H	2.04505714035400831818	−0.38527966060593860087	0.34334668466284012922
H	1.22668371945762122444	−0.33871697624721180953	1.70096075989260131500
H	−2.32724769002378240046	−0.01363704638415085338	−2.10282429856022856640
Na	−3.35014633970076358693	−2.04255948102326767568	2.28530274379976194865
O	0.02943375923488303000	1.09408871580136124457	−1.49213353114342939243

O 0.72637653469450280674 −1.61382941176145555673 −1.20956440009863963603
O 0.11127709018535947294 −0.50782945275108348770 −3.39320253179971054180
P −0.38264748838936513131 1.04072308600514973342 −3.04105739765378402240
P 1.29479667262101050262 −1.13086408062457599577 −2.52474246394142198824
O −1.85911369937198123203 1.05149415920785171963 −3.16835527582523956980
O 0.53178711842661241516 1.94607033668676465687 −3.77423587189759235372
O 2.43225411372495292639 −0.17482030126192929509 −2.42075428423794258848
O 1.69280571714733052424 −2.42444756780526127216 −3.32999749350641094381
H 2.30976018701070229255 −2.22010776203844129029 −4.03045649843319253591
Na 2.20731191151599803746 1.62263119238980446646 −2.87901588491302584671
29
−82.9900769171 converged=true
Re −0.36327538824395727568 −0.23896309985519109675 −0.27017503434378897786
O −1.49028130868666508491 1.23858136022358555905 0.74291757871645480726
O −0.91503339898157842747 −1.51953975791975492982 0.94729072788467716393
O −1.43400651837367587582 −0.02400468062080329812 2.87797049024603390777
P −2.40157786070467116346 0.58831734678818881079 1.76704747858546595829
P −0.73872192317046747601 −1.51486517825115329927 2.53095528803015623609
O −3.16289352166077764039 1.72668047966730853204 2.54352615926501002264
O −3.37000065657457659185 −0.42048463264780022719 1.26607044911328303627
O −1.67177347140884235976 −2.50981786298275943992 3.10312471416984392292
O 0.70012542755418527562 −1.37599385583465938510 2.86423998309348126767
O −2.20347105223345618441 −0.67174191279163730783 −1.34048470511750794643
O 1.35634754326293038851 0.24631325362320738437 0.97950428136739220442
H −2.63187582038482537072 2.51398377607792467003 2.65178853853811347108
H −2.91213986374561306647 −0.45989653939969932228 −0.70814986585493988613
H 1.29831369050534140008 −0.43905142893763271505 1.73391767079176584154
H 1.14508463292624318441 1.09043199934622370151 1.39832280067677849900
H −2.23785820911118227272 0.02857817748365917843 −2.07520751308825257553
Na −3.30739269940849212048 −2.11118066211366528506 2.12986662157856265765
O 0.14843013179232444898 1.08534986820771939264 −1.45934972217884806334
O 0.76667800531715624146 −1.62613047686631251310 −1.30763603505788283954
O −0.00709742042048455701 −0.47065797136385123522 −3.40683315049876433989
P −0.34431929309278180851 1.09309993960577411798 −2.99154652605903015328
P 1.22138764128395238551 −1.20436630813179768040 −2.68672244932779813453
O −1.82087678076751191547 1.22477744649544462163 −3.02495832378028861598
O 0.58830856485902538466 1.96418906931290937479 −3.74303574109041026219
O 2.43802063393257517632 −0.35517659485680469489 −2.76255627590289298823
O 1.38590068507816210897 −2.52433022256205052969 −3.53003915544486090994
H 2.07886556365556840476 −2.41838131282680457446 −4.17968030722196193238
Na 2.28577065828133241254 1.48507279019778293261 −2.97781297133689504264
29
−82.9765621636 converged=true
Re −0.46178096562329284946 −0.14756820886579338770 −0.38381072548040251480
O −1.37711281009327279889 1.35368408813385232570 0.80370826757129409934
O −0.92040772932883507806 −1.46196104542444937913 0.86829700309912705158
O −1.36851194469074588511 −0.00273642083175759665 2.87666492831718834111
P −2.30776639361517510451 0.65965890275089111849 1.77461431420604309750
P −0.67713658265811560444 −1.48239436044844707929 2.43244983256852176368
O −3.09935924501175996681 1.75202809596476916099 2.58746524629169449838
O −3.27097255352646332227 −0.34370887155863150131 1.22502456205414911317
O −1.58616479286307909824 −2.48753599998616747868 3.03181567369332993778
O 0.77572595349411077592 −1.34869639697869780903 2.70107585744385048088
O −2.33118021635956340631 −0.56274136731736223993 −1.36070806463591109647
O 1.31722059017741921494 0.27948647928636749516 0.78270369984051968437
H −2.62978186624841114849 2.58402806248563532421 2.61508836692785573419
H −2.31408129312629640140 −1.44805276734746901646 −1.74824037054618397491
H 1.12526165656551646599 1.11886130906747216507 1.22465760222662201073
H 1.30388391702958195140 −0.41575465100885800052 1.53433602070191099642
H −2.98347254839807085602 −0.52994115302118172117 −0.62955165123914091918
Na −3.30834252969955944224 −1.91566550668001145397 2.34361466454412470384

O −0.04356702787879779271 1.15034784966938796735 −1.58673699634394571589
O 0.30186857295018032499 −1.70332282020548286816 −1.54005629432697466186
O 0.27066607815673715542 −0.31705182296908301920 −3.59358246544162129865
P −0.18410176123522070668 1.24231837280424484149 −3.19734721590455972517
P 1.23154617566288537844 −1.11153187072337300556 −2.59585741546354586973
O −1.58768992179980661383 1.42005901644040655185 −3.57038396418087744522
O 0.94068431860422463409 2.10882281770217350214 −3.64111292746229686657
O 2.34720474782633381494 −0.27717809739906618116 −2.11037807563006829525
O 1.75535775324215159721 −2.29970988479269111693 −3.48895406467369051384
H 1.06390320132594662361 −2.92774244790225646184 −3.69218167072089942238
Na 2.41874521918415474886 1.51479169763938537052 −2.59025913168321997304
29
−82.9752400814 converged=true
Re −0.33718643189615671307 −0.11218852175456674181 −0.27526253751950680027
O −1.39847364366574389294 1.34996160027634659606 0.81773568180274658346
O −0.87855583024955574700 −1.43178516794165067161 0.93923423877785494618
O −1.49504969944119414649 0.01634155076675553042 2.90050599564000766151
P −2.37841315832275679298 0.64544566153569760125 1.73192098040578645879
P −0.73517323880295404326 −1.44452195365152658013 2.51623720955086982443
O −3.23928517696444639995 1.73184016233952320363 2.47818303344891743834
O −3.28124695708240565750 −0.38618540737864676116 1.13816196904275690471
O −1.65057167574831131773 −2.47275806017142718574 3.06245426251549179852
O 0.69348801586231811900 −1.26698700381090345601 2.87944587454794609371
O −2.11108522365656714470 −0.58363193216746200864 −1.37004925921831088864
O 1.36683078892232234836 0.34155357569920263838 1.00331840731492727414
H −2.79840856853783170877 2.57956348499916598982 2.50121124585317700095
H −2.04673624237784546054 −1.46309849008389591951 −1.76833334404858266709
H 1.15916235954244806194 1.18671917729553766385 1.42532064063873686877
H 1.28518658694366871842 −0.34814954254507662901 1.76158540981591160701
H −2.83318649162483104931 −0.56050339016800843073 −0.70836339008451032484
Na −3.34097005832561366745 −1.95801002159736725616 2.25154170597882030691
O 0.18969180622953962789 1.17768540695601475754 −1.45143032340960265181
O 0.57216431938571188365 −1.63109476873505232497 −1.35466220784339697047
O −0.08948338560784097062 −0.35114042277558721317 −3.40693025650318936925
P −0.22389727602753667357 1.26707619524766901264 −3.01986759588971453994
P 1.06328118284333550392 −1.22104139076494089267 −2.72998889684180934267
O −1.62578331209379767230 1.66355955927121845761 −3.14576591769516156205
O 0.91262337377470881261 1.95209132349239089521 −3.69011270258666890243
O 2.38209598837441793862 −0.54542246439897879018 −2.78923315129057325379
O 1.04536540721765724982 −2.54171007424606187897 −3.59902501165719801435
H 1.74256985959090693328 −2.50566928338669603349 −4.25147021065421171215
Na 2.45168467850936133701 1.31285318030681574797 −2.77400684963039179110

29
Coordinates from ORCA−job Tc_Szimfiz_d_job2 E −82.473628122130
Tc −0.37330480330088 −0.23361673894345 −0.26986075780626
O −1.49552112800509 1.21165910618713 0.75206135949780
O −0.89130006924237 −1.47777640341185 0.94978575716678
O −1.45576425462765 −0.03279366946960 2.89703502961766
P −2.41658660907899 0.58296040318336 1.77829468874378
P −0.73501726670541 −1.50301040939447 2.53929356003351
O −3.16656946720396 1.73274580836437 2.54645455725633
O −3.38928718766523 −0.42312501602459 1.28191496598362
O −1.65895587535017 −2.52395007548375 3.07776072881727
O 0.70017860279289 −1.35659857134304 2.88095899475143
O −2.22312946636459 −0.63778340748179 −1.33158874205573
O 1.38101695564044 0.21428240232586 0.95571936165872
H −2.62393025353249 2.51108706503091 2.66352524544347
H −2.93542408487136 −0.40328960392533 −0.71261487141453
H 1.31985986828754 −0.45881247329800 1.71684112388099
H 1.23270793695393 1.06989984352574 1.37717631433642

H −2.25364182225513 0.03578659309212 −2.09109344492254
Na −3.29849693791214 −2.12617462547789 2.11273616146487
O 0.08822316593326 1.06021002636714 −1.46402879691755
O 0.74514819376085 −1.59245351538766 −1.30973626809952
O 0.00851526476760 −0.47213043137118 −3.43471800438016
P −0.35899133440810 1.07944490211825 −3.01417774483606
P 1.22531047056350 −1.20021718230990 −2.68608090042776
O −1.83470803770606 1.19800061341128 −3.08160539165453
O 0.58629418168855 1.97090624989414 −3.72359357315497
O 2.44116016779056 −0.34927547532629 −2.75146432567644
O 1.40042558734181 −2.53051495100850 −3.50841714894736
H 2.11012335854022 −2.43634472499181 −4.14182326721585
Na 2.27230283564771 1.49167727021616 −2.93639960539049
29
Coordinates from ORCA−job Tc_Szimfiz_d_job3 E −82.459890595890
Tc −0.47896252848567 −0.14860791643096 −0.38917315671303
O −1.42498855907196 1.30916140117377 0.78885217576879
O −0.92778211967239 −1.42263072238232 0.86405506824993
O −1.38638383488038 −0.00907234993862 2.88672965419834
P −2.34208836448312 0.64312415744707 1.78907721811470
P −0.67601623039420 −1.47270149410085 2.43050812080120
O −3.11041508125904 1.75299459527479 2.59829934361570
O −3.31959015088436 −0.36222954832264 1.27026692750941
O −1.56889155352717 −2.49898153573566 3.01600047183025
O 0.77691070402943 −1.33378426525212 2.69076034277845
O −2.38114415861653 −0.49317489734287 −1.32286209108693
O 1.34164657656044 0.22682663818315 0.72484658834168
H −2.62125233099324 2.57375632996876 2.63122231202106
H −2.45209582965258 −1.32197635437523 −1.81234850427690
H 1.22707297408351 1.07749141795928 1.16947390863924
H 1.32273013255214 −0.45569973158393 1.48440898115641
H −3.03368331894976 −0.47217803375160 −0.59285420764025
Na −3.30683425659811 −1.93790552078015 2.37192580828478
O −0.06802483777242 1.10973028631103 −1.59704575414833
O 0.35784178421247 −1.65016216101665 −1.51395314899356
O 0.32439681861236 −0.32853855288626 −3.60387432434269
P −0.18737399531609 1.21224256139548 −3.21476369712408
P 1.28974053792688 −1.09787163210433 −2.58710826976987
O −1.58935995455455 1.34522728413974 −3.60979161271717
O 0.91750230689963 2.11753498683073 −3.62806210749386
O 2.41020309521023 −0.25162910762603 −2.13576150276512
O 1.80148558001391 −2.31467463927922 −3.44529173902850
H 1.10675054781437 −2.94704207361409 −3.62358986130683
Na 2.39924404925898 1.55156387632354 −2.56759193814992
29
Coordinates from ORCA−job Tc_Szimfiz_d_job4 E −82.462354320030
Tc −0.31796671062936 0.22774503315263 −0.27958297621828
O −1.80600227488076 1.23348528292585 0.79953997765939
O −0.55214320458121 −1.22676082176829 0.83974892339408
O −1.59762766963935 −0.15495169471512 2.84883405242440
P −2.58677994627160 0.28183264104484 1.67645582313017
P −0.46399720719325 −1.32583490645236 2.41959023403759
O −3.70645599970754 1.08886445905505 2.43062803336874
O −3.19271031944592 −0.92052936214597 1.02525980442605
O −1.08123529588864 −2.60244376414956 2.84640184660569
O 0.85568488208518 −0.81491107783280 2.86629435972907
O −1.98562062089776 −0.59903043860688 −1.40630254835086
O 1.26284772063001 0.92401675516934 1.03602055940874
H −3.46256848143914 2.00540152816910 2.55223394598430
H −1.73068218263757 −1.40642117461885 −1.87966719046386
H 0.99338476372860 1.75707855915221 1.44272358126566

```
H     1.26733955132054     0.23702829499428     1.79789106212877
H    -2.65580419850575    -0.81223436332916    -0.72184252812846
Na   -2.82384422854342    -2.51382083134124     2.02069681277131
O    -0.01050388000819     1.59754921730242    -1.39281642866291
O     1.08514381497808    -0.84342375343890    -1.32290132825148
O    -0.14255169385814     0.04606387878332    -3.34307008209206
P     0.31265266188970     1.62312415954559    -2.96060771124312
P     0.76599118646232    -1.12101199256927    -2.79164019363629
O    -0.53808594562779     2.53141621024563    -3.71438412995451
O     1.80405345764292     1.62257823264405    -3.00044608974927
O     1.99370214579965    -1.35081938241140    -3.57567840435537
O    -0.24847178870340    -2.36499447538038    -2.85200545401707
H     0.00687536872116    -2.93076102345268    -3.57992611569345
Na    2.95601409197167     0.21255779263705    -2.70909283505583
29
Coordinates from ORCA-job Tc_Szimfiz_d E -82.477457835950
Tc   -0.49123983398091    -0.20355036389549    -0.26426164058372
O    -1.64990968116189     1.23254695428308     0.69500270135496
O    -0.97684827147065    -1.43175233284019     0.98759823817275
O    -1.47788980094441     0.07894516695759     2.89951567355513
P    -2.49727582779957     0.65386961633712     1.81113347644774
P    -0.76653472403969    -1.39040887011118     2.57456603939346
O    -3.19136092375755     1.84234834835367     2.57062086023695
O    -3.49710011339616    -0.36760359677844     1.41262994564549
O    -1.66045746809872    -2.40286309272013     3.17744480752087
O     0.68130647022770    -1.23743808765193     2.86008988357725
O    -2.36190073887199    -0.59797426284701    -1.29222254887087
O     1.38083923238807     0.11815810833274     0.79748132091025
H    -2.60273575017413     2.58930169100978     2.67482386677361
H    -3.08136966967146    -0.27390737105514    -0.72732135296588
H     2.04754951145256    -0.41752645549286     0.34318095168316
H     1.23769105111960    -0.33404959009551     1.70164779670389
H    -2.34470861995711    -0.01952062243456    -2.12666357903454
Na   -3.33063454583331    -2.05242434804575     2.25296999121897
O    -0.03906564583081     1.05361749792329    -1.50135933888695
O     0.67953687003551    -1.59835721140848    -1.23237047958171
O     0.13266834309046    -0.50797198253024    -3.43325883057828
P    -0.39850158328638     1.02075150173445    -3.06961435712527
P     1.29152137929736    -1.13078344598620    -2.53057226618695
O    -1.87062297089479     1.01483228888999    -3.23226357067317
O     0.52712012482117     1.95522341963442    -3.74924084245769
O     2.42255496290047    -0.17094744802063    -2.39538430043282
O     1.70996927430284    -2.42751170694252    -3.31753269540797
H     2.34802533681903    -2.22730326639975    -4.00030432288432
Na    2.18001161272825     1.63309246179959    -2.81398042752758
```

10.2.3 Table 4, Article 4.3

```
29
converged
Re   1.74108547339787     -6.22973855260052     -2.33479885271305
O    1.95917922444164     -4.47852616340671     -3.57096335131602
O    0.551173851142197    -5.45458850378051     -1.20174463638213
O    2.26199864499616     -3.70053513291658     -1.16770993443172
P    1.5653318224488  -3.26594918712373  -2.65948740601711
P    1.2586172874973  -4.46806350079177  -0.158803512978953
O    2.16513950362242     -1.98813485212754     -3.0652817929554
O    0.0692879398056352   -3.29396070590535     -2.45386287786829
O    0.320281129821726    -3.49960641806339     0.450643166290716
O    2.01770342047063     -5.40697902390816     0.727217271427572
```

O −0.210225134221194 −6.91131973379369 −3.26182411490461
O 3.87290435056592 −6.03430374058422 −1.39589386184129
H 0.138999947037048 −7.29643593008507 −4.10645473073849
H 4.36402215291484 −5.37312743676813 −1.90149057142637
H 4.17571488212918 −6.9333685909139 −1.66933439828092
H −0.47808183166825 −7.66303179825643 −2.71236861455309
O 2.98078501261241 −6.91825253498458 −3.56703238446624
O 1.63246915502309 −8.03392208645999 −1.12600322986944
O 1.64183568343871 −8.99627038395778 −3.45451059376257
P 2.40117652201972 −8.03321075787489 −4.52021167890897
P 2.37655628085272 −9.14747401311928 −1.926648653648
O 1.34945127215128 −7.30875330793456 −5.33400738377077
O 3.478612825789 −8.81376046877771 −5.17078325756684
O 3.8399704009037 −8.75240937736092 −2.10143543264927
O 2.13141175694038 −10.5152511802968 −1.45821448657721
Na 2.16437327412683 −7.31512983171855 0.62445527507635
Na −0.189151766252656 −2.23133828936563 −0.900056811762227
Na 4.29541404774893 −9.8164665056898 −3.67775165422426
Na 1.28493287477047 −5.22229199753211 −5.25140149198089
29
converged
Re 1.9916090058314 −6.41677352655776 −2.26182522151545
O 2.1462797566105 −4.65503919672707 −3.57917258895687
O 0.809196468765944 −5.66194368971348 −1.05469552273229
O 2.17850093631787 −3.6172865834992 −1.28104128398155
P 1.43150602119249 −3.50514263829012 −2.80622105703579
P 1.42187721259633 −4.5150718750687 −0.158847708036777
O 1.7094445771271 −2.16334941824623 −3.33189738773732
O −0.0312529764305844 −3.87260218553423 −2.61666786068788
O 0.37317659628201 −3.68638149714681 0.475749684008268
O 2.47568476988921 −5.22952993850743 0.660808344945866
O −0.0929744772993011 −6.75577643018936 −3.09467834498882
O 3.98815160215537 −5.72373149387442 −1.43924026365082
H −0.409318989435821 −5.83046273910601 −3.11719175531062
H 3.67987676149717 −5.3029771366162 −0.598322998404159
H 4.23681770263796 −4.99547375258841 −2.02759477008406
H 0.127771032291594 −7.05681458191443 −4.00303971689338
O 3.15185682870699 −7.19523047904983 −3.46191570834825
O 1.98575524220834 −8.15353988500223 −1.04589393007817
O 1.48387275056069 −9.02280839265986 −3.40120600978214
P 2.36449681117951 −8.16999617019774 −4.44058608933908
P 2.28257407795559 −9.40909938641445 −1.94400504877583
O 1.44333076539242 −7.2356492338932 −5.20042093619121
O 3.26057802503243 −9.05760201645929 −5.21543041729003
O 3.74722233157606 −9.40463183182904 −2.30603980726002
O 1.70912101188745 −10.6571081297051 −1.43198485645803
Na 2.48317403510675 −7.29477485095447 0.639812982392083
Na −0.499241659725844 −2.79586212892092 −1.06138826853619
Na 3.90759648446513 −10.3647157725498 −3.92481034946848
Na 1.80428729485997 −5.35282502371551 −5.31801311789425
29
converged
Re 2.01124320712644 −6.408637665029 −2.27098115202349
O 2.25795089430632 −4.62411099899825 −3.53834191380204
O 0.825627632085664 −5.65057199325033 −1.07164755803499
O 2.1630969635278 −3.58206513934441 −1.26377250870011
P 1.47641687019348 −3.49021281596039 −2.81464105834537
P 1.40235504911618 −4.49529821791777 −0.160099862487283
O 1.72536495798818 −2.14021627360719 −3.33096221581206
O 0.0121277110878544 −3.90216745835377 −2.70036990659086
O 0.320136495417483 −3.68658888359977 0.445027955631778

O 2.44628509061803 −5.19687026256184 0.679489356374265
O −0.095478816596507 −6.76106985822778 −3.0931766012942
O 4.00821422080471 −5.77789849066412 −1.37679646076867
H −0.421788322190531 −5.83927613865962 −3.1233245553677
H 3.67670196530159 −5.28093453055202 −0.590169288580131
H 4.41986307531113 −5.13162067631992 −1.96829248023438
H 0.103748392743562 −7.08248586516603 −3.99973396456709
O 3.14414990448622 −7.17867502083602 −3.49851144832641
O 1.96574963827226 −8.1499008787034 −1.05801252001176
O 1.47808167226927 −9.01378263623725 −3.40917448914817
P 2.34911247577374 −8.16476976286023 −4.46168408063874
P 2.28683172445005 −9.39570924630669 −1.95911555492829
O 1.40708026241713 −7.24588383197282 −5.21380606598373
O 3.23966890788762 −9.05696846958702 −5.2353243593071
O 3.75072017759998 −9.36473607273538 −2.32661692175246
O 1.73966662801003 −10.6574936865179 −1.45142397540593
Na 2.46388245037908 −7.28581705380145 0.645291719044655
Na −0.520022375538796 −2.82798199286962 −1.13746820970705
Na 3.90868812272919 −10.35043907685 −3.93771063285939
Na 1.61549502365756 −5.36001698214948 −5.12441125446457
29
converged
Re 1.9395377987133 −6.40510566289237 −2.22335141742606
O 2.28772401001528 −4.6215652788141 −3.50223541241761
O 0.738294927372925 −5.64288937676727 −1.06519702809992
O 2.18727689186231 −3.65731813119749 −1.19258043022483
P 1.52513560207415 −3.49120778316456 −2.7564913068918
P 1.31173374250783 −4.51103372928714 −0.109987129235958
O 1.821803477278 −2.12711735733904 −3.20858618493989
O 0.0489847353174937 −3.86685767671963 −2.69925958451438
O 0.234047674709695 −3.63150572458982 0.396873886331744
O 2.2436669317115 −5.25042333759671 0.800882077645972
O −0.126430679065435 −6.78844732743084 −3.10479796904539
O 3.96766793994217 −5.76418596500445 −1.38286398579494
H −0.473393487696896 −5.87786792385178 −3.15228885834647
H 3.77093536741232 −4.82533751130076 −1.1982381055441
H 4.56524257603541 −5.79823861370713 −2.14514494837261
H 0.11897598492309 −7.10788207353515 −4.00308994425943
O 3.12979465121826 −7.14884318797714 −3.43609046036797
O 1.91577858503307 −8.15526038909927 −1.03625061226605
O 1.49817164778427 −9.01985496186714 −3.39743722485407
P 2.3746088067778 −8.14196721706066 −4.42115929534688
P 2.2851279031905 −9.39246914961234 −1.93204759043396
O 1.42874095783931 −7.23650492718018 −5.18663750440344
O 3.30498789827939 −9.00809539834067 −5.17842876539122
O 3.75815797675626 −9.32037381015124 −2.26491768528086
O 1.7629684970971 −10.6741572890931 −1.450976306433
Na 2.51093841513881 −7.22517307538962 0.643160060116488
Na −0.510714150174537 −2.74593010804703 −1.18240069169861
Na 3.93718106387587 −10.3241862430458 −3.87454140951885
Na 1.60402424801371 −5.34240075486891 −5.0816761916607
29
converged
Re 1.81045601903327 −6.23109529837396 −2.38755112609763
O 1.98361471228527 −4.46360731052937 −3.58391090957756
O 0.573240190715483 −5.50893375751937 −1.24660768960569
O 2.23939653077045 −3.71018267437146 −1.16622291820428
P 1.54392933014564 −3.27504053448373 −2.66114031409912
P 1.23947169442264 −4.51464213028602 −0.183797751915707
O 2.09928836772874 −1.96687187253753 −3.02981994014147
O 0.0457595530296806 −3.35646569678933 −2.47196288936285

O 0.258348929547693 −3.5793205378069 0.412842352334033
O 2.00794244650918 −5.435310922829 0.713106939830935
O −0.193912366538787 −6.86984937448802 −3.31184890263585
O 3.90815104763274 −6.0337100962986 −1.39867096109234
H 0.024779547789612 −7.79544319535632 −3.53202160404973
H 4.40946396366354 −5.36840450782913 −1.88921786094001
H 4.21723981747854 −6.93048145371157 −1.67205407767506
H −0.809080709109421 −6.87297828271114 −2.56328778295034
O 3.06121801318735 −6.90783231626055 −3.58782560025007
O 1.63112697766977 −8.0375693001568 −1.16403934922959
O 1.64424346919404 −8.92463724302365 −3.52114890305959
P 2.49805743925042 −8.01141387263129 −4.57763022055817
P 2.36782080104479 −9.13515235987336 −1.98724066325818
O 1.54336164084067 −7.26399741125355 −5.45865395218868
O 3.58501621795306 −8.84947532612222 −5.13587321090722
O 3.8395795217972 −8.75341933292632 −2.11593368014074
O 2.10509275783398 −10.5191384164895 −1.57685156026211
Na 2.18503569825582 −7.33889527430664 0.591272969392245
Na −0.212064224780497 −2.26955609669938 −0.921538636273565
Na 4.31509823908654 −9.88047840469736 −3.64587668728451
Na 1.23929437279732 −5.29829701102802 −5.2722550831802
29
converged
Re 1.903610827584 −6.3143034715799 −2.35251127563317
O 2.02571437468785 −4.52425832840553 −3.61394881648046
O 0.691034332003221 −5.67480578304473 −1.11365072252409
O 2.09756695640917 −3.66937360504174 −1.23570086407036
P 1.34525229879304 −3.43501720445619 −2.73296029322728
P 1.25899212215904 −4.53752648945678 −0.149668382396394
O 1.57155299439879 −2.04060909439404 −3.1289065694952
O −0.131519678267025 −3.82115804009388 −2.5688587451563
O 0.164572846066511 −3.69103776354157 0.385899521445726
O 2.16976003507583 −5.27426575660719 0.778476078957358
O −0.17601305418452 −6.37877269082412 −3.36968826194222
O 3.98860819352597 −6.26255212133075 −1.33509097822353
H −0.717503976541009 −6.99627533759984 −2.86011098267439
H 4.53152785128764 −5.64719188103056 −1.84593496499513
H 4.22650546060374 −7.19560502912608 −1.62187101064887
H −0.420531259218861 −5.44598547869119 −3.08819893612168
O 3.11614868599522 −6.94988754707793 −3.5926008519215
O 1.77806271046945 −8.10506695236195 −1.09956178370835
O 1.69911289488815 −8.94750211998881 −3.48281239819802
P 2.54655101388953 −8.07961287249396 −4.56324094996747
P 2.45044241739992 −9.19420879498784 −1.98774085199016
O 1.64355397779478 −7.33596741212025 −5.49362848993305
O 3.63808018552815 −8.93068277485566 −5.09750384574509
O 3.92943632981371 −8.81541439355549 −2.1498963482015
O 2.21645332766279 −10.5895414298921 −1.59985752607966
Na 2.34294901243108 −7.22630545793663 0.719023186982353
Na −0.513435158554545 −2.60739773781518 −1.07075173065966
Na 4.30471889616021 −10.0322204422463 −3.64871360582908
Na 1.47976537608111 −5.37965399025042 −5.42574959907054
29
converged
Re 1.88053386325651 −6.31778987188955 −2.35940080500904
O 2.03430997188297 −4.52925094138521 −3.60960770778976
O 0.673925692296237 −5.6795580110718 −1.11614745308336
O 2.12024271372122 −3.70267531293641 −1.21999352653252
P 1.37402280391435 −3.43787571134703 −2.71568430235827
P 1.25867257095288 −4.55979097341065 −0.142757607660543
O 1.62490344663082 −2.0424720221273 −3.09211240186949

O	-0.109098674749151	-3.8025048703057	-2.55839889348664
O	0.178372397327342	-3.69543137506659	0.392497981146998
O	2.15097919739124	-5.31878764095765	0.785861850629717
O	-0.196119522718197	-6.35408405869344	-3.38543230626297
O	3.95504130213116	-6.21122980131149	-1.37132352047767
H	-0.749747662988094	-6.96097206611664	-2.87599540558172
H	4.43455357228533	-5.59870983030324	-1.94558935932318
H	4.21591842498019	-7.14452466889081	-1.63204022567061
H	-0.421237475508731	-5.41795171430841	-3.09788914144455
O	3.09974050229396	-6.9563474489786	-3.59522995671794
O	1.75547483588084	-8.11163453113952	-1.10786023554613
O	1.71662169797833	-8.97831758301427	-3.47902860312422
P	2.55300667390473	-8.10255971875482	-4.55964149034301
P	2.45917921742075	-9.19669935295551	-1.97444090574794
O	1.64271111638614	-7.37534974351531	-5.49637726055338
O	3.66309004298823	-8.93611173984958	-5.08366157762463
O	3.93244238243616	-8.79404054866559	-2.12751705006352
O	2.24692784408767	-10.5912646913	-1.57102573162262
Na	2.33455731608994	-7.26683764169637	0.702344080287213
Na	-0.478234503225317	-2.58994285300512	-1.05596701968135
Na	4.33966442391753	-10.0109323977766	-3.61827273976222
Na	1.47051582297791	-5.41855287474151	-5.43506868752615

29

converged

Re	1.91038940374252	-6.33948013756852	-2.23803622257651
O	2.15637561759448	-4.55084267485514	-3.51518648052162
O	0.729139712135577	-5.58705922573965	-1.07004821906634
O	2.06515161545914	-3.5467426138253	-1.21869371675035
P	1.3929970796562	-3.42540048121962	-2.76092843184196
P	1.25722273155417	-4.41796042591619	-0.1179017264365
O	1.6307908058821	-2.0642779051666	-3.2554353868406
O	-0.0777795727247932	-3.84261824613512	-2.68374644212449
O	0.132670382468453	-3.59596822178532	0.38927673440067
O	2.18857857210193	-5.12102741447606	0.816262022400936
O	-0.155512985784471	-6.65362016104867	-3.13040938819116
O	4.04802215108357	-6.28657764368862	-1.32240830402578
H	-0.487233559644131	-5.73175618628482	-3.17454150690922
H	4.26716422280859	-7.2144277339929	-1.62205211641691
H	4.57210835952869	-5.67341866015042	-1.85524337067402
H	0.0581230933669665	-7.00467652305702	-4.02466289867396
O	3.07581390334346	-6.98078249127471	-3.54493136938935
O	1.86379462142679	-8.09625086286899	-0.979756666460559
O	1.59549679077364	-8.95398191731569	-3.3519491725341
P	2.35743037135068	-8.05694243200074	-4.45989333922454
P	2.43751808777327	-9.21933343785853	-1.89855770942618
O	1.33869010913159	-7.24824714324253	-5.23510379066183
O	3.34070054825009	-8.90995021673056	-5.16811591195823
O	3.90513951085256	-8.88283964479163	-2.18495484540355
O	2.18279580910133	-10.6021759939741	-1.48887582513245
Na	2.37215358608591	-7.07503635576576	0.785459137532236
Na	-0.593050335906354	-2.68113772570192	-1.17341250887331
Na	4.14186360746056	-10.0426506094167	-3.75983390910079
Na	1.45441574977866	-5.29701689378764	-5.10207864850255

29

converged

Re	1.88617514302277	-6.34715598597897	-2.20651757642443
O	2.06526490215581	-4.60026118223802	-3.54957572862155
O	0.704996826478579	-5.58935806940943	-1.03798158984376
O	2.0552124633755	-3.55602409442967	-1.26205727945784
P	1.33993158078457	-3.45359790916171	-2.78650354493798
P	1.27290147411136	-4.40794795016536	-0.124928245029456

O 1.58520094003512 -2.10981015273923 -3.32565242198193
O -0.129890928022525 -3.84657957341797 -2.64547752967433
O 0.172125806907275 -3.57049112043755 0.408299307657536
O 2.23219572041922 -5.10125672033953 0.788582364639544
O -0.155361049345366 -6.64219476285554 -3.10506529345814
O 4.05942255157558 -6.32631641537086 -1.27853150386407
H -0.4673688936919 -5.71117387105263 -3.14694286835413
H 4.5795924356488 -5.71161645713609 -1.81239770450773
H 4.26221743622511 -7.2499275625322 -1.59782348381029
H 0.0784561500778425 -6.97339219107753 -4.00102263181563
O 3.07115502082561 -6.98931861038553 -3.48691207826492
O 1.87548478685411 -8.10589291773148 -0.975922050241797
O 1.59731336509785 -8.95670422001902 -3.36204138230869
P 2.36203614051555 -8.03255630333473 -4.44414845944199
P 2.43157218582887 -9.23436465652851 -1.90314768650612
O 1.36089846742228 -7.20025104351759 -5.21515914407181
O 3.35501523554146 -8.86128765398424 -5.16870429346158
O 3.90318622858043 -8.91477287070591 -2.18440989938849
O 2.15589575510094 -10.6142541073992 -1.49876256658996
Na 2.41470310475538 -7.05533072562043 0.803091630768949
Na -0.617472355336582 -2.7037036428813 -1.14398859431411
Na 4.14435512451965 -10.0186528980383 -3.78104540566072
Na 1.56575438506322 -5.21800633231803 -5.30101434912585
29
converged
Re 1.90494968645982 -6.31458549264544 -2.35622658636141
O 1.80802473342449 -4.60332940372945 -3.64383044508396
O 0.71060572974636 -5.58792097262742 -1.14026910713958
O 2.1768407112236 -3.60772318294563 -1.3217512607044
P 1.33216789464357 -3.38319545236524 -2.78515280609275
P 1.42078271278764 -4.50922135553116 -0.21965986507046
O 1.71624091278685 -2.07821673911577 -3.33281538068798
O -0.129942518124185 -3.58754100367369 -2.44312628454115
O 0.450057128951443 -3.67612110353819 0.526314360527295
O 2.44750915627495 -5.31951547345772 0.547677657874396
O -0.145334482630539 -6.83601008933388 -3.15238899215283
O 3.95158397889727 -5.78814550158461 -1.55824306996777
H -0.600021977860262 -5.98132796475696 -3.22658367451524
H 4.40935054140469 -6.64058035478274 -1.47955640686311
H 3.70342826362493 -5.45481080014648 -0.663527211632052
H 0.100185330007448 -7.16637160418128 -4.04861134653856
O 3.0983956026521 -7.04291398168148 -3.57176918131342
O 2.00529886452019 -8.0250962618846 -1.06887749592351
O 1.62397884543708 -9.01684924630524 -3.39150054828307
P 2.38325912117361 -8.11747430469355 -4.49324221690254
P 2.47039756004094 -9.24791900103203 -1.93055254757656
O 1.35903864961669 -7.30276050934717 -5.25905465759596
O 3.34953191903789 -8.9535276993111 -5.24156638227723
O 3.9324269616043 -9.05245433301363 -2.2769585699302
O 2.07877179413356 -10.5501333501805 -1.38178789882123
Na 2.16633893966184 -7.25730053163344 0.667418130125812
Na -0.366501420071824 -2.54530182032769 -0.852980059268708
Na 4.16722157981828 -10.0887788035727 -3.86816870074436
Na 1.636383779992 -5.36707366340766 -5.37896945533899
29
converged
Re 1.89337817068051 -6.32336943697446 -2.34463728832387
O 1.82625992460472 -4.6073104128242 -3.6408905324949
O 0.716894686127304 -5.59849206975256 -1.10812224236843
O 2.18085009104688 -3.6108835235014 -1.32686994931579
P 1.33170160428808 -3.39350157485914 -2.78662743651088

P	1.42785709065165	−4.49817538487407	−0.210964938166878
O	1.69556253310271	−2.08260378308908	−3.3345012692032
O	−0.128376462984219	−3.61680675769999	−2.4448417943765
O	0.457498021943001	−3.65389295149627	0.522051818495247
O	2.46403445223401	−5.29089162829196	0.56249621029491
O	−0.149134923524279	−6.82428610953301	−3.14737406381652
O	3.94943327383793	−5.82035481370787	−1.53951800103346
H	−0.597176934970961	−5.96549014261392	−3.22071909786077
H	4.40157674063397	−6.67687643995755	−1.46485333450842
H	3.71495844208955	−5.47305353398687	−0.645924402804786
H	0.0939966463415174	−7.15748041430401	−4.04295636709828
O	3.07856826224548	−7.04284562809003	−3.56465465977488
O	1.93460677128653	−8.0581507715959	−1.07832129831204
O	1.62609961230501	−9.03221256628707	−3.40636866839641
P	2.37870030514226	−8.11790065595853	−4.49797912419583
P	2.45727623867803	−9.25500147252387	−1.93442304680291
O	1.35255586800306	−7.30574312846626	−5.26318231528697
O	3.36306063347114	−8.93909921276576	−5.2395041380027
O	3.92137564556464	−9.00805949138142	−2.24681190942493
O	2.10366906337113	−10.5785882440717	−1.41332828722845
Na	2.21407372146057	−7.2150698174376	0.564837244826628
Na	−0.364904647586123	−2.53672607076619	−0.870552530489081
Na	4.18840065279685	−10.055437615724	−3.84276866569151
Na	1.62817451716042	−5.36389634746662	−5.37844991213122

29
converged

Re	1.90140656721769	−6.3139386483984	−2.35716623643674
O	1.89618664183775	−4.57384118419742	−3.63042400684735
O	0.733312500421563	−5.59428259064631	−1.11926811799962
O	2.18116519239953	−3.59579154139743	−1.31110119543676
P	1.35034415880339	−3.38327758725147	−2.78264563830003
P	1.43212018012989	−4.4982090973172	−0.206968888382254
O	1.68309613943199	−2.05430889397984	−3.30415194483906
O	−0.112341271228201	−3.65103843387972	−2.47627580019881
O	0.446042250604325	−3.66764919837498	0.521049879633977
O	2.46763521492684	−5.29294496463445	0.564201683511969
O	−0.148765425379251	−6.79791351124202	−3.1666240373661
O	3.95226474190187	−5.83338076685699	−1.54805191607583
H	−0.594568528055108	−5.93725563598917	−3.23868649864279
H	4.39452086445739	−6.6961952356559	−1.47767541312136
H	3.72285101040938	−5.48477061866187	−0.654326039182466
H	0.0808797622197699	−7.14760663556297	−4.05994923009151
O	3.06945925786362	−7.0309857705857	−3.59744728866796
O	1.90446843175204	−8.05343977046867	−1.08271540115858
O	1.62533775315011	−9.03234894493507	−3.40218323476103
P	2.37247315480751	−8.12954034718314	−4.50749577633055
P	2.45541229155292	−9.24183595390642	−1.92992648728835
O	1.334833814199	−7.3379240504966	−5.27933236065914
O	3.3601082073851	−8.95941540230631	−5.23422270310033
O	3.91774403976527	−8.96912346738382	−2.23443628080794
O	2.12673061806418	−10.5720510234179	−1.40882111475846
Na	2.2291324226817	−7.22309672599892	0.561535585789427
Na	−0.382780642259799	−2.56845841201586	−0.892736090223603
Na	4.19166105960896	−10.0543124591126	−3.81658330865467
Na	1.57023959585709	−5.40726312894936	−5.27333214240358

29
Coordinates from ORCA-job Tc_Neutr_d_job10 E −82.099512373310

Tc	1.90405929725863	−6.31423548246848	−2.35620517063677
O	1.78617858457243	−4.60221323345810	−3.60050010872101
O	0.74169487786975	−5.57235147648051	−1.16334766492506

O	2.17377716519932	−3.57012569204088	−1.28919791573230
P	1.34258906873632	−3.35276539587737	−2.76386722744878
P	1.41520502091056	−4.48885123142868	−0.20878843797238
O	1.76975152776682	−2.07220955069067	−3.33531059084916
O	−0.12210624227571	−3.51552025914153	−2.41938661465694
O	0.41799785050841	−3.68268598379760	0.52952130124469
O	2.43527585123332	−5.29749218905449	0.56775865542827
O	−0.11717247264120	−6.89244085326334	−3.15475062561694
O	3.92308821770115	−5.73303967079649	−1.55674814413910
H	−0.62300971411877	−6.06856772952247	−3.23198968323048
H	4.43107640062521	−6.55528251868905	−1.47722005754401
H	3.69633058134372	−5.39180982371057	−0.65954271521103
H	0.10815464717253	−7.23229354004759	−4.05268347907091
O	3.06585487245378	−7.05706674210501	−3.54913391960911
O	2.02396267299376	−8.02594377221612	−1.11260182114817
O	1.62835326683142	−9.05535436490745	−3.42430929705753
P	2.38904990172268	−8.13787610337365	−4.50437030473786
P	2.46032323840765	−9.27723856217028	−1.95091163636890
O	1.37049967740617	−7.32637303804514	−5.27986673496862
O	3.38349243120476	−8.94593523082484	−5.24428619930912
O	3.92495423906467	−9.12083814220624	−2.29779989404976
O	2.02757709508470	−10.55572484727657	−1.37910283957609
Na	2.18475453145667	−7.25501061416669	0.62797145957948
Na	−0.34956918059235	−2.48730340930751	−0.82467145428024
Na	4.14909921557192	−10.14495413672471	−3.89400612432463
Na	1.61972737576642	−5.37069640701441	−5.34041275786706

29

Coordinates from ORCA–job Tc_Neutr_d_job11 E −82.099298192970

Tc	1.89156168592645	−6.32259426861656	−2.34740974334396
O	1.79548390705717	−4.60643724416991	−3.59350017753142
O	0.74641039496512	−5.58282607260211	−1.13268454510860
O	2.17854311352841	−3.57218276235195	−1.29285363075691
P	1.34300673644196	−3.35804626478065	−2.76233919632566
P	1.42079437585040	−4.47884829725343	−0.20057238343074
O	1.75877207329825	−2.07511027262086	−3.33734589930547
O	−0.12044932577608	−3.52999405982103	−2.41472017634031
O	0.42478006477706	−3.66352022783239	0.52844991576556
O	2.45060964606945	−5.26984984226092	0.58240011081692
O	−0.12171006033395	−6.88770766762362	−3.15182819420468
O	3.91823090749188	−5.76238681670431	−1.53781341283046
H	−0.62811199088136	−6.06443059309361	−3.23420537888499
H	4.42564664710224	−6.58560811471627	−1.45940526925881
H	3.70133126850636	−5.40676677942415	−0.64264891569368
H	0.10287519986312	−7.23368739563537	−4.04741184483913
O	3.04759563399565	−7.05968947237563	−3.54272846896047
O	1.95858821113567	−8.05461076933402	−1.12584899978299
O	1.63126866218794	−9.07285724337075	−3.43929672458457
P	2.38684838935144	−8.14072884156716	−4.50877277958745
P	2.44864579946553	−9.28449819679961	−1.95500488361673
O	1.36701345313218	−7.33151865128223	−5.28348161807192
O	3.39825678848442	−8.93304061446265	−5.24286869177183
O	3.91638404700371	−9.08212389910056	−2.27351834043322
O	2.04981164310323	−10.58290385498940	−1.40528674311013
Na	2.22765236390503	−7.20596569624450	0.51648463351635
Na	−0.34017833667804	−2.47023910356456	−0.83303937577553
Na	4.17134781480487	−10.11598885075608	−3.87194647215668
Na	1.60996088622325	−5.36803812664703	−5.33656279439529

29

Coordinates from ORCA–job Tc_Neutr_d_job12 E −82.099048052920

Tc	1.90130960434848	−6.31377028731094	−2.35699656947925
O	1.85350329632059	−4.57887879981372	−3.58376535469329

O	0.76106804837685	-5.57922837206577	-1.14163368462737
O	2.17727797715917	-3.55849097112846	-1.27842231085986
P	1.35557899097903	-3.34952602618642	-2.75975588227769
P	1.42339735305701	-4.47800901643818	-0.19625779433145
O	1.74701316208086	-2.05050737073816	-3.31365782807698
O	-0.11047704599985	-3.55945869694642	-2.43857683042760
O	0.41412144545703	-3.67366585688737	0.52719352321382
O	2.45258661763811	-5.27147678177909	0.58396077883787
O	-0.11872470167473	-6.86221114307948	-3.17207756712421
O	3.92220245191434	-5.76634400805525	-1.54233924233902
H	-0.62412880557801	-6.03773407965333	-3.25323614680685
H	4.42701850299857	-6.59129256778919	-1.46195919032718
H	3.70725697276124	-5.40745049984358	-0.64845327745391
H	0.09676507922178	-7.22159124976196	-4.06569408723547
O	3.04185474034276	-7.04780848520426	-3.57241735097263
O	1.94818745287503	-8.04861175939716	-1.13014252679562
O	1.62790090929734	-9.07030074370916	-3.43512936674248
P	2.38086128561508	-8.15019343886957	-4.51740378825193
P	2.44899186801230	-9.27737633869852	-1.95311443906791
O	1.35105198579337	-7.35798656591908	-5.29809929960108
O	3.39136900620763	-8.95369192428621	-5.24010903484357
O	3.91501303943336	-9.06522628361269	-2.27318970490895
O	2.05935698789458	-10.57689012705971	-1.39907716843517
Na	2.23741364349149	-7.20800911572571	0.51025902160045
Na	-0.36138309849304	-2.49886940993059	-0.85121587580162
Na	4.16751333502853	-10.12687055245030	-3.86011666500518
Na	1.56706989996758	-5.42072952846622	-5.22433233996538

29

Coordinates from ORCA-job Tc_Neutr_d_job2 E -82.103870865840

Tc	1.99990539011839	-6.42268402403438	-2.25886246831395
O	2.11469098421967	-4.67221515699667	-3.52851138088447
O	0.84462601246927	-5.62528643598869	-1.10082928729739
O	2.18241326545787	-3.56456154915252	-1.25615453055880
P	1.44936407130560	-3.46592253101924	-2.79471641295721
P	1.42361652249109	-4.48504626857774	-0.16179195489255
O	1.80316598599272	-2.16149276517225	-3.36515712559773
O	-0.02464338469929	-3.76266332322529	-2.59438915979429
O	0.35299983432485	-3.68804096445157	0.47473976055283
O	2.46048213683961	-5.21203956666945	0.66639952930900
O	-0.06645306805420	-6.84138762027190	-3.08366588565395
O	3.96617884221141	-5.70650009020118	-1.42912380448018
H	-0.45953477738924	-5.95187905845340	-3.12025479417767
H	3.66939874532614	-5.28575341179982	-0.58305029991270
H	4.23677703514164	-4.98049023232514	-2.01065697499238
H	0.13330233400491	-7.15380199610166	-3.99391155342881
O	3.12608282220892	-7.19891794087268	-3.45472902263583
O	2.00218545480026	-8.14932182385413	-1.08411788507690
O	1.50154721146513	-9.05778526235100	-3.43397856308538
P	2.37037375792563	-8.18039260540501	-4.45920858670127
P	2.28145625737085	-9.42700507465444	-1.95987028053701
O	1.44100229355506	-7.25423824947767	-5.21779591761028
O	3.29696748347629	-9.03938519278431	-5.22799910424799
O	3.74791082178416	-9.44855917897164	-2.30840371919066
O	1.67346990404191	-10.64948709098181	-1.42840208815391
Na	2.46148114100000	-7.29670528231385	0.61032986678728
Na	-0.47519472167651	-2.72228859576904	-1.02955915530499
Na	3.93090181059608	-10.36538344257921	-3.94759427593811
Na	1.71649583292651	-5.33296525047558	-5.26449493331595

29

Coordinates from ORCA-job Tc_Neutr_d_job3 E -82.104357134650

Tc	2.01925734127280	-6.41097338597298	-2.27238498632000

O	2.22649406401691	-4.62527160227407	-3.49870875441871
O	0.86792146429262	-5.62103356842614	-1.11202502699070
O	2.17799340699877	-3.54145959613317	-1.23005783810201
P	1.49323431185985	-3.44896865130269	-2.79015497662683
P	1.41769109341476	-4.48063806168752	-0.15512012426925
O	1.81276762535005	-2.12263830123513	-3.32644998452514
O	0.01968981274994	-3.80126584650179	-2.65635771039247
O	0.32086579875432	-3.70275368378787	0.46064193529812
O	2.45014550100287	-5.20395710865606	0.68082694544215
O	-0.06137198523154	-6.82229212757854	-3.10120719474880
O	3.96784606019306	-5.70742969565797	-1.41831376624807
H	-0.45403579699889	-5.93185170347820	-3.14037586279260
H	3.66518220723512	-5.28576842234470	-0.57501055673146
H	4.24432297503201	-4.98209571004927	-1.99939283001071
H	0.11550459200850	-7.15754429297250	-4.00836966075867
O	3.12072621239764	-7.18723791722909	-3.49240864573791
O	1.99066480906902	-8.14264050875878	-1.10088710456745
O	1.49397770251670	-9.05548690190979	-3.43717749293693
P	2.36143874532338	-8.19057651754645	-4.47624831326130
P	2.27942631444853	-9.42246811054027	-1.96875727769953
O	1.41675522434816	-7.28412064894567	-5.24004799168986
O	3.28446920523189	-9.06150217056020	-5.23418332710208
O	3.74490235383284	-9.43690661800093	-2.32147806653047
O	1.68064749980683	-10.64610304039144	-1.42905513889385
Na	2.45238587300273	-7.30275669584796	0.60625570169939
Na	-0.48185466514696	-2.74914532934978	-1.08399624767825
Na	3.92080305016591	-10.38369090465476	-3.94888409965273
Na	1.61311920228690	-5.39362285784575	-5.07643161184516
29			
Coordinates from ORCA-job Tc_Neutr_d_job4 E -82.103007403270			
Tc	1.94402827570080	-6.40785573558097	-2.22416358456783
O	2.23246107848176	-4.62570069028128	-3.46870073954119
O	0.77237399319764	-5.61089736815017	-1.10898553299552
O	2.18988602462042	-3.61445968334104	-1.16851114426941
P	1.52580640879801	-3.44969704268213	-2.73802617099154
P	1.31196894536733	-4.48815065140376	-0.11173943075743
O	1.89352495844198	-2.11246653500225	-3.21598300046123
O	0.03983961133644	-3.76006220295963	-2.65750197019281
O	0.21611513122476	-3.63679945708273	0.40045442572922
O	2.22910682933009	-5.24353147485620	0.79948153981592
O	-0.09575288972059	-6.85304202963545	-3.10545746595073
O	3.94849695313363	-5.74213349501818	-1.37974608083682
H	-0.51926973051247	-5.97935660549591	-3.16061281715387
H	3.75828336570350	-4.80348188734248	-1.18644606768677
H	4.56362088857986	-5.77142856137276	-2.12787212688446
H	0.12543614912783	-7.18281333418392	-4.00735207953121
O	3.10294176955406	-7.14951515047341	-3.43486939166467
O	1.94511601453875	-8.14609223418759	-1.07855385088281
O	1.51524540372808	-9.05620501717968	-3.42645973743007
P	2.38106401610405	-8.15925477587226	-4.43824552804385
P	2.28845336208514	-9.41212455952693	-1.94714316271298
O	1.42629276565063	-7.27103158875406	-5.21250435157826
O	3.34256645772034	-9.00048682336465	-5.18332471498414
O	3.76163640430526	-9.37172664812154	-2.27532779600158
O	1.72739298228596	-10.66589427055615	-1.43921509980290
Na	2.49218719058606	-7.21745386683639	0.61999334299294
Na	-0.47840522152259	-2.64967542154049	-1.13757918074936
Na	3.94952792162489	-10.34698845282475	-3.89620160683359
Na	1.57102493447135	-5.37387442130458	-5.03516669470781
29			
Coordinates from ORCA-job Tc_Neutr_d_job5 E -82.106496414680			

Tc	1.90006008094981	−6.31225418967105	−2.35793078114799
O	2.10191665656171	−4.53077623299778	−3.49878924457235
O	0.68778395086322	−5.52492929261722	−1.26326434257900
O	2.25690569525865	−3.63007953397676	−1.10777113253791
P	1.63012984818221	−3.28172221605236	−2.67596864554942
P	1.28552030246367	−4.52878647275838	−0.16602796681457
O	2.26271409573671	−2.02672965053198	−3.09297091342274
O	0.13014428717265	−3.32372675878230	−2.53981013037070
O	0.24395204270422	−3.66626005605803	0.43170041820167
O	2.13302023544096	−5.42111688378726	0.69118844776300
O	−0.11729358986101	−6.94749985688614	−3.24022514784799
O	3.92129314956003	−5.67597124188421	−1.47812438784943
H	0.09349565287335	−7.86498085540024	−3.51278600559355
H	3.70650917297814	−4.76147694736622	−1.19898045798467
H	4.53653043494618	−5.62056393486428	−2.22393670882339
H	−0.73377498481832	−6.99403325919537	−2.49490469775662
O	3.11184965240234	−7.10089826085773	−3.45171250672144
O	1.69340208420423	−8.09473184262711	−1.21620374382192
O	1.54696710646849	−8.99760308221521	−3.60448254667557
P	2.52014414451743	−8.10240712947941	−4.54661911206037
P	2.17529875493026	−9.34121458599168	−2.03521757757996
O	1.67558836460617	−7.21544193077452	−5.41180641587218
O	3.56449572859106	−8.96692670354201	−5.13713261370343
O	3.67580964774299	−9.29047714910830	−2.16926419794395
O	1.55318362143510	−10.60202372675430	−1.61944547713006
Na	2.42755885556946	−7.34700790659183	0.45424470876052
Na	−0.31811234486648	−2.41511088476728	−0.93827232847380
Na	4.10688869823186	−10.22563068058577	−3.76588048700379
Na	1.38898865438964	−5.29181874526538	−5.17536601827145

29
Coordinates from ORCA–job Tc_Neutr_d_job6 E −82.101125204060

Tc	1.90326570611093	−6.31472502066761	−2.35460839732597
O	2.01061437161555	−4.52942481431235	−3.55734744012547
O	0.73788482120307	−5.64316463897801	−1.14315395764136
O	2.06623505934018	−3.59823150455578	−1.20546218746025
P	1.33185290719566	−3.40765541179082	−2.71755889690601
P	1.25621819496396	−4.50845681254946	−0.13971012908455
O	1.57734521630980	−2.03080772674166	−3.15689010807167
O	−0.14845929646885	−3.78393398299508	−2.55636578929876
O	0.12483169977752	−3.71181824853045	0.39471896264015
O	2.17255668083461	−5.24008874299815	0.78560259413248
O	−0.16612312053502	−6.34275112706159	−3.36464379593484
O	3.97516862366630	−6.29332079797697	−1.34255446975124
H	−0.73684939727249	−6.95359150908916	−2.87921180370627
H	4.54714483213508	−5.68362149058562	−1.82799965597349
H	4.20681234838118	−7.22976858564955	−1.63019974557204
H	−0.40160702515447	−5.40630397074026	−3.08041327122056
O	3.06892560866326	−6.98436848805894	−3.56632079159548
O	1.79474604525997	−8.09970254773196	−1.15655544893264
O	1.73436138098563	−9.02474035550088	−3.51139052060848
P	2.54736725096090	−8.11361383198573	−4.57424678729450
P	2.46853976650671	−9.22215129132248	−1.99981492401059
O	1.63449361109330	−7.37524165862916	−5.49759035030103
O	3.67640842491054	−8.91213439333443	−5.11090484892116
O	3.94998410579979	−8.85007446139547	−2.16017028522721
O	2.21802238479975	−10.59931293265348	−1.56472602684795
Na	2.35532963355701	−7.19722413681003	0.70264748531857
Na	−0.53906798252266	−2.59120839571086	−1.04690589255670
Na	4.33749020211885	−10.03859717745109	−3.67290073524656
Na	1.45747793970686	−5.41616594499957	−5.41108277998413

29

Coordinates from ORCA–job Tc_Neutr_d_job7 E −82.101130311430

Tc	1.90152039980050	−6.31278249409302	−2.36047166518867
O	2.01310830971354	−4.52674840371286	−3.55350675130672
O	0.73618420556764	−5.64252576499469	−1.14892780278693
O	2.07168999840568	−3.60233690608709	−1.19976480219779
P	1.33917025045193	−3.40354027593783	−2.71196526806047
P	1.25662413325036	−4.51388183375200	−0.13898640618962
O	1.59118035354192	−2.02608498656127	−3.14531712232574
O	−0.14312044643287	−3.77351886512286	−2.55410906314547
O	0.12623750384293	−3.71628168516126	0.39633993248562
O	2.16687740861068	−5.25385759721189	0.78540753155285
O	−0.17233857806074	−6.33157525873063	−3.37353340748154
O	3.96922625790701	−6.28198531789110	−1.35102066679001
H	−0.74339602457183	−6.94224267615529	−2.88811106222074
H	4.53759080470385	−5.66946036854512	−1.83717332872464
H	4.20675920438375	−7.21764773105019	−1.63619117624068
H	−0.40278735155382	−5.39614956417660	−3.08197042713641
O	3.06568147228817	−6.98599526357713	−3.57211685172012
O	1.79140948991375	−8.09895142974996	−1.15714881603884
O	1.73812041992355	−9.03278855541899	−3.50672251011595
P	2.54734015900377	−8.12342370930452	−4.57350670353125
P	2.47226189425486	−9.22057548469232	−1.99458730357890
O	1.63035595502863	−7.39399181001898	−5.49973888227619
O	3.67972472001188	−8.91931046310903	−5.10698941184811
O	3.95244608898399	−8.84313208045178	−2.15543051904695
O	2.22982352029693	−10.59786524143852	−1.55446233124964
Na	2.34187741543331	−7.21184635331454	0.69669359239348
Na	−0.53185917866442	−2.58763516177538	−1.04060158093940
Na	4.34655243619014	−10.03459869688028	−3.66333466102356
Na	1.44270917171784	−5.44146601659958	−5.41851253806712

29

Coordinates from ORCA–job Tc_Neutr_d_job8 E −82.104355739840

Tc	1.91828024686310	−6.40571637006870	−2.11705358163793
O	2.12793724070990	−4.61122783242052	−3.35074711549356
O	0.84386182333422	−5.55019288090084	−0.94631249869540
O	1.83218983179280	−3.30028581385841	−1.25708151450280
P	1.24546101314558	−3.45559147842525	−2.80982454671214
P	1.21847290904146	−4.24351392191175	−0.09363028776476
O	1.42189706620839	−2.19083659726535	−3.53380849673863
O	−0.20648889272051	−3.94497434859709	−2.70550531516903
O	−0.01031056774525	−3.59361346445180	0.41575888057222
O	2.31985608949171	−4.69111939417647	0.81194294480108
O	−0.07297356202180	−6.58414536957623	−3.06014055091564
O	4.11491088125183	−6.65733331820705	−1.08606100743229
H	−0.36450373030500	−5.62934179240484	−3.06122777459974
H	4.15778821633872	−7.60715314767105	−1.48579830874230
H	4.67352680964936	−6.13144043982352	−1.68072129220214
H	0.12634420226978	−6.88942102195415	−3.97303843312324
O	3.05880441373259	−6.99961067587743	−3.41657827186115
O	1.80728853519450	−8.17750381265102	−1.13031395634375
O	1.60213222441126	−8.98325365920640	−3.50824128274036
P	2.39971760244938	−7.98111651222589	−4.48066794222916
P	2.34574699703771	−9.32559981422992	−2.02420940659725
O	1.42118212770772	−7.11375412371443	−5.23405704294921
O	3.44585240157387	−8.74633796607991	−5.20034358662379
O	3.84733213348055	−9.03880552902543	−2.21473094217393
O	2.00707788730045	−10.70227365936972	−1.67680872527365
Na	3.02606468203036	−6.44470727834309	0.62697341334100
Na	−0.86422163782330	−2.87970670377652	−1.20432782419460
Na	4.13455624680036	−10.04406735273692	−3.87640655664724
Na	1.58318679745142	−5.17955570068980	−5.07279899073365

29

Coordinates from ORCA-job Tc_Neutr_d_job9 E −82.104716545890

Tc	1.90969787086969	−6.42098911607284	−2.09446359890543
O	2.08833767695525	−4.64686913341879	−3.36937766600458
O	0.83685605000700	−5.54955247149421	−0.93037420055288
O	1.82299085673376	−3.29924291146066	−1.29251340828751
P	1.21569385593301	−3.48237405295365	−2.83453037958445
P	1.23193612799775	−4.22991216404944	−0.10686649540615
O	1.38848477821847	−2.23575675370916	−3.59314031425175
O	−0.23553954142053	−3.96068721580595	−2.69386249842444
O	0.01503193819057	−3.57322049105577	0.42273807770022
O	2.35089973563001	−4.66751968878689	0.78217792078768
O	−0.07444924815032	−6.59611801109481	−3.03069056409200
O	4.12355470386187	−6.68398789841482	−1.05663007009825
H	−0.36279573775118	−5.63929397970767	−3.03290936080612
H	4.67852010575580	−6.16186186351810	−1.65768214934366
H	4.15777413348466	−7.62949789459811	−1.45969344634172
H	0.13817692213147	−6.88684069456665	−3.94473759526564
O	3.06571742368597	−7.00545006050915	−3.37905689870696
O	1.81342876469997	−8.19156215062069	−1.11661205690028
O	1.59941347659014	−8.97072750863530	−3.51233852437154
P	2.40256233002688	−7.95555150595799	−4.46762880836216
P	2.33764169141568	−9.33400937521278	−2.02841401652168
O	1.43851372679636	−7.06367465882547	−5.20893174202469
O	3.44813251174680	−8.71052941890531	−5.20011253049023
O	3.84121112793547	−9.05803630910438	−2.21219314271917
O	1.98211733899087	−10.71001750333716	−1.69629556912137
Na	3.02838767275609	−6.43391940569299	0.63156418785937
Na	−0.87556087208685	−2.89586609115061	−1.19098069227384
Na	4.13789665007850	−10.01494391480144	−3.88945816365011
Na	1.65633793344336	−5.09418775734575	−5.18274630193195

29

Coordinates from ORCA-job Tc_Neutr_d E −82.107568038660

Tc	1.84716109039423	−6.32028651330880	−2.29639689185408
O	2.09932204258920	−4.55323300198108	−3.48076145790049
O	0.67876800393697	−5.48387538396816	−1.21309009213243
O	2.27015926068814	−3.60968666063224	−1.11040156506463
P	1.64588460768000	−3.28387875027407	−2.68084053299645
P	1.29884387606399	−4.47583548183291	−0.13876457816196
O	2.30124295610847	−2.05172756547404	−3.13300439264401
O	0.14654698884884	−3.29733645078707	−2.54551954920550
O	0.28074196138411	−3.58684645849137	0.45850653671387
O	2.14224649220135	−5.36891425461493	0.72108110212370
O	−0.12496226557377	−6.97601321393513	−3.17456581846490
O	3.89269133240299	−5.69956612948335	−1.45823717655519
H	0.17319290636256	−7.30187550675859	−4.05781220918871
H	3.71162226368187	−4.76943398397490	−1.21074576142406
H	4.49710444654951	−5.69386184132054	−2.21456597916386
H	−0.39438372913606	−7.76255003916984	−2.67453870545101
O	3.04345520432534	−7.10457021025425	−3.43443197087044
O	1.71896922033374	−8.10066241274279	−1.16593504930789
O	1.53230277265642	−9.06076345798161	−3.52789077420732
P	2.41038932818302	−8.11038506934051	−4.48932766493947
P	2.19848386390578	−9.35949017812382	−1.97558833735688
O	1.45540778580278	−7.23760954768477	−5.27805255938774
O	3.44066291131066	−8.91092961024260	−5.18506465282577
O	3.69208353832384	−9.28784408233417	−2.16702218103497
O	1.60536243827852	−10.61403947113138	−1.50418608880532
Na	2.39407490120393	−7.30296365078175	0.50786582383036
Na	−0.31965529483074	−2.40079959667446	−0.94779891118667
Na	4.09146438524640	−10.15082076069376	−3.81192303849316

Na 1.43178671560441 −5.22640072210536 −5.15674752684453

10.2.4 Table 2, SI 2

```
25
converged
Re −19.0783251831254 25.4676889026955 −9.36610107797639
O −19.9791730517356 26.8853277244867 −8.19340610370435
O −19.5195013752914 24.1428354146375 −8.07760699076838
O −18.629505153188 26.893858860142 −10.5595768994279
O −18.0646614275074 24.174172754444 −10.6258983931884
O −21.391389934434 25.3034765364538 −6.91416610347014
O −16.9471458245359 25.8790763286538 −12.0703233662061
P −17.2020621805904 27.1113126131614 −11.1026991283988
P −18.0622849879169 24.6592344433026 −12.0764537465782
P −21.4552956178197 26.636183665171 −7.87208825817081
P −19.9624893379244 24.631060228508 −6.67127596623763
O −21.9017957613348 27.7606369400965 −6.86666489237468
O −22.3755501503595 26.4370765494646 −8.99415848937414
O −20.3524808011163 23.3481381920914 −5.84909345412091
O −18.9908067342712 25.5084479675237 −6.00103491529137
O −17.30556269132 23.5863938852937 −12.9359145387247
O −19.3360041087957 25.1082154383632 −12.6480996589721
O −17.2369150350954 28.3523884536767 −12.0665938945787
O −16.1950628351078 27.2397791224837 −10.0274204657793
O −20.7799900446508 25.0416684833613 −10.4848495897584
O −17.3573006221476 25.8352661656287 −8.19615763997548
H −21.2265332420235 27.9596852695068 −6.21644671634177
H −19.8916831572836 23.3300804484917 −5.01208633885289
H −16.5899582258232 23.1739361349304 −12.4519526759006
H −16.586799286603 29.0055745306659 −11.8117133214871
H −21.557512344642 25.5903343674746 −10.1980745167612
H −17.6366287589209 26.1977987293174 −7.334348301169
H −16.7448646774753 26.4470540745999 −8.69316912917529
H −20.560717451357 25.1219977687933 −11.4480254263666
25
converged
Re −19.1320957341614 26.0310907776892 −9.3588020381065
O −20.4060602662931 27.2614996411185 −8.2792625825224
O −19.7125932432855 24.5759891168689 −8.26032110171161
O −18.4491632691876 27.4062839752863 −10.4713529609141
O −17.8736930085937 24.6769325731763 −10.260990314439
O −20.9642019617316 25.6780657657785 −6.4269473882447
O −17.4800007473087 26.0275959718876 −12.3013568873548
P −17.1070122507542 27.1378178281751 −11.2103645096193
P −18.037790706272 24.5882249083977 −11.7676280577292
P −21.605756375993 26.5031441182436 −7.70597002444657
P −19.7817459270296 24.6596944584551 −6.72101611840813
O −22.5059766641909 27.5439508339235 −6.95128680875686
O −22.3386367740026 25.6050770768369 −8.60447798403623
O −20.3266567867264 23.2849561612615 −6.18653797106481
O −18.4938904818882 25.050831890054 −6.11045781173431
O −16.947609351589 23.581042175496 −12.2982366036532
O −19.382819400594 24.3106927094181 −12.3060572380442
O −16.8381447057735 28.3865180732793 −12.1298476324011
O −15.9966558736871 26.7940581320952 −10.3149902971672
O −20.735954683314 25.7295475547714 −10.6510817423293
O −17.4998931085722 26.405723053344 −8.08061566403959
H −21.9880231482036 28.2310557642629 −6.53160910962078
H −19.718524366581 22.8954100504705 −5.55977676996353
```

H	−17.2918658548536	23.0880977645846	−13.0419643808545
H	−15.9592867887958	28.7293920397637	−11.9762766733268
H	−21.5440489585591	25.505998511055	−10.1236327875643
H	−16.6756564231069	26.1689771235434	−8.54883053681789
H	−17.6179758242658	25.9048390479842	−7.22703167875049
H	−20.506267314686	25.0661929027786	−11.3586763263786
25			
converged			
Re	−19.1100381637194	26.0437312649319	−9.37148784558015
O	−20.2224932608143	27.3452972401712	−8.20457187897889
O	−19.7309001999738	24.6039571431584	−8.27324756983987
O	−18.3804830614635	27.393876835591	−10.4910992848962
O	−18.0063590373091	24.641947053234	−10.3811656423419
O	−20.8353986173837	25.7424377949681	−6.37295566117523
O	−17.6466492295414	26.0024431834612	−12.4204984718521
P	−17.1176567991653	27.0359353386011	−11.3229607956615
P	−18.244597828109	24.567317807733	−11.8929440624799
P	−21.4352837417893	26.6669558692733	−7.59585173631277
P	−19.7213118672551	24.6715011624867	−6.73110163357017
O	−22.2224312363208	27.7473267068641	−6.76888784165739
O	−22.2913077613405	25.8556633123015	−8.48390256921466
O	−20.3245383260515	23.3261236147776	−6.18209492372673
O	−18.3814706029777	24.9692764982945	−6.18337255258801
O	−17.2092827498183	23.5321487605589	−12.4681558070764
O	−19.6183209239757	24.3608209990716	−12.3575260153004
O	−16.7830185402263	28.288123707671	−12.2137618320077
O	−15.9879178602804	26.540710071732	−10.5224420749775
O	−20.7876949549237	25.8553201464093	−10.5881016322505
O	−17.3989080391083	26.3024438089689	−8.16716153500552
H	−23.1582918235194	27.551507901754	−6.76983476634228
H	−19.7004487306608	22.8780543748876	−5.61286781691623
H	−16.3462365896626	23.6117055970468	−12.0591281836979
H	−15.8950694901831	28.5967265128474	−12.0415718601242
H	−21.5777803868299	25.6903519140892	−10.0099468327935
H	−16.6102208273148	26.0156696338897	−8.66429515702592
H	−17.5175206511158	25.8091880940371	−7.3080885854097
H	−20.6563686962716	25.1821376499007	−11.3063754303284
25			
converged			
Re	−18.8624514951716	25.6602997691135	−9.17210022566963
O	−19.8566047316716	27.0370085990357	−7.99434566634618
O	−19.5131511560518	24.284340293029	−8.01329623661965
O	−18.260804657389	27.0588593377055	−10.2429858492859
O	−17.802038567182	24.2710906168164	−10.3144884670512
O	−21.3168449394915	25.4679310594187	−6.74011380451331
O	−16.5631702643616	25.97377649572	−11.672644951865
P	−17.1607947318881	27.4458037460556	−11.3041020246576
P	−17.6477124053215	24.7708647653287	−11.7363714895626
P	−21.3378824852604	26.7662917553964	−7.76016069612291
P	−19.9519176124605	24.6557573115531	−6.56940157788867
O	−21.89691768262	27.9067917544514	−6.8398841132598
O	−22.1810191855212	26.4729254056171	−8.92656497251953
O	−20.4289367787891	23.3419276476251	−5.85733823491794
O	−18.8851714107246	25.3589945285271	−5.84052550295725
O	−16.8846074838231	23.6461429987882	−12.5230135640429
O	−18.8642289922231	25.2782880390283	−12.4341681911225
O	−17.9827682449495	27.8322649726957	−12.5878823189129
O	−16.1436744358836	28.3775645360689	−10.8709299100153
O	−20.4952237615653	25.2524379791468	−10.4079051013664
O	−17.2458103648596	25.8978525843167	−7.80612784643632
H	−21.2444624725389	28.2257275462656	−6.21482680878168

H	−21.1317919430516	22.8849882446165	−6.31960470688755
H	−16.9255252606578	23.7736948747586	−13.4696665221949
H	−18.5430431949297	27.0826546919692	−12.8514458889544
H	−21.3035166940157	25.7705897440267	−10.1331577511514
H	−17.6713764451093	25.81949068092	−6.90464097859496
H	−16.5755517983311	25.2081117622414	−7.89843122926066
H	−20.2410008171376	25.3062282531843	−11.3592753628805
25			
converged			
Re	−19.0721470593221	26.0592762080474	−9.34025962725833
O	−20.2698115206498	27.3369937390081	−8.22524640502309
O	−19.6745013642916	24.6199623066968	−8.23005620801837
O	−18.3738489303407	27.4069830315918	−10.4758833673786
O	−17.8842745115821	24.6632711888303	−10.2776396291306
O	−20.8821334651308	25.742489566727	−6.3833858739629
O	−17.4828578601873	26.0040753900792	−12.3250364651435
P	−17.053792004826	27.1010142981478	−11.2410402285644
P	−18.0803147907146	24.5849841268536	−11.7820307534353
P	−21.4756784712623	26.625158056118	−7.63903131445459
P	−19.7137846662716	24.7133249947928	−6.6893235328472
O	−22.3227415710063	27.6922330463504	−6.85457476083705
O	−22.2786207251156	25.7714637635949	−8.5371334051947
O	−20.2810238113735	23.354008201657	−6.13432165829836
O	−18.4052070964497	25.0734632377173	−6.10674054651749
O	−17.0366429592217	23.5404984359474	−12.3337467987484
O	−19.4448846965036	24.3543493584498	−12.2929301637365
O	−16.7640523705647	28.3380693719666	−12.1696391950521
O	−15.9380728110848	26.7228290619587	−10.3668985950065
O	−20.7130756698412	25.8052155847645	−10.5951166460929
O	−17.4071891956068	26.3866540053736	−8.10136847883422
H	−23.2511626930359	27.4652336465381	−6.88123581524817
H	−19.6455908493937	22.9293648375923	−5.55961330506223
H	−17.4102100604414	23.0649664368313	−13.0747045154822
H	−15.8948486977291	28.692161398544	−11.9887174742811
H	−21.5135922871115	25.6131800116679	−10.0408925230432
H	−16.5956333507788	26.1206304734703	−8.57499420769152
H	−17.5266807963479	25.9101147575268	−7.23449867874989
H	−20.5256257109199	25.1367314671597	−11.3093398101629
25			
converged			
Re	−18.6775627266476	25.726635604207	−9.18987505844109
O	−19.4903674327112	27.132002349699	−7.88051180909312
O	−19.8081634921572	24.4607725026564	−8.32417537516783
O	−17.5637447807006	26.9776569056367	−10.0330548395803
O	−17.8709184226032	24.2791469782387	−10.3664418302478
O	−21.0111377864748	25.6709488391547	−6.53266473901014
O	−17.0399452475199	25.7874891827102	−12.1468890906645
P	−17.2717863740961	27.2561252859578	−11.554729022663
P	−17.9650542338319	24.4651484524777	−11.8949097860404
P	−20.9677180753165	26.9084877364391	−7.61396582650623
P	−20.0461156611341	24.4390159557561	−6.79751344758378
O	−21.4621972341355	28.1005396364647	−6.71421977807799
O	−21.8592940225199	26.6113227201573	−8.74964199750417
O	−20.9562501540181	23.2022348433013	−6.46496250298023
O	−18.8012116553511	24.478173284991	−6.00140080572171
O	−17.0645793216378	23.3529712747302	−12.5425238596571
O	−19.2985816246578	24.5649687563695	−12.4947172015995
O	−18.6773474999638	27.6954630839006	−12.1513613752587
O	−16.1808432138306	28.1629054273646	−11.8635094460371
O	−20.1615443646028	26.1022888033868	−10.6030135127795
O	−17.150394990116	25.5604086633831	−7.65101648019078

H	−22.393416480592	28.2642864183633	−6.85380665676218
H	−20.5431259205416	22.6340399827085	−5.8166954691869
H	−16.2209851357854	23.2579802007522	−12.0989102507456
H	−18.5725710763884	28.4925511729397	−12.6711246204224
H	−21.0290750343944	26.2158572161874	−10.134529429595
H	−17.6173239063329	25.0769010963458	−6.90669459115354
H	−17.0365310506104	26.4664424061103	−7.33380419079235
H	−20.1802130784335	25.485935218322	−11.378737021544

25
converged

Re	−18.7952361747847	25.7322790112843	−9.07741371373495
O	−19.7436992928418	27.1936763456078	−8.00837678765596
O	−18.9423950761341	24.6206964751882	−7.57327822489078
O	−18.6774202056985	26.9126703030866	−10.5857519833692
O	−17.7547899139172	24.3654556891471	−10.2326126169988
O	−20.8816948706744	25.6525300148192	−6.44283108169538
O	−17.1332610141902	25.7975175258931	−12.1772968657912
P	−17.3733048395139	27.1456481920181	−11.3710147414669
P	−18.0091622041754	24.4713260881772	−11.7370057080485
P	−21.1662664106565	26.8190342161425	−7.54735373505717
P	−20.1920306759311	24.2286408428125	−6.70431980322856
O	−21.6708772380746	27.978521132487	−6.61145132295967
O	−22.1354101333388	26.4386726171384	−8.57662936183217
O	−21.1866715037862	23.5145401140948	−7.71481646954172
O	−19.9495396874778	23.4954934409366	−5.47555521383172
O	−17.1879951205004	23.3313942900746	−12.4364045252201
O	−19.3945398090517	24.5899378304039	−12.2016192600675
O	−17.6828907547629	28.2169139996595	−12.478068155153
O	−16.2167389450587	27.5077989722465	−10.5201217162583
O	−20.527822416409	24.9027689650568	−9.8936882091482
O	−16.9986148508695	26.5947756430301	−8.25964597455438
H	−21.076893213987	28.1676565071371	−5.88504044391992
H	−21.4417111951292	22.6572585407403	−7.37390676759405
H	−16.3640281144949	23.1449250335698	−11.9861458616858
H	−16.9492124883758	28.8175647859221	−12.5991673279518
H	−21.3287653508053	25.4379819420223	−9.66045995403683
H	−17.2397619842174	27.3536766728119	−7.71268902608035
H	−16.4911912545815	26.949186218124	−9.051572078099
H	−20.4060752523751	24.7901585784961	−10.8711630851348

25
converged

Re	−18.757325030889	25.7580023445613	−9.02577913706082
O	−19.7958661657729	27.2104915107888	−8.02893423953811
O	−18.9205632822518	24.6863395596538	−7.49399283673828
O	−18.6373293843432	26.910699958256	−10.5567163084075
O	−17.6268951863252	24.4071538712217	−10.1110143066359
O	−20.9412399699791	25.6696380691866	−6.46692766107007
O	−17.0608247221661	25.8013280391195	−12.1113769177992
P	−17.318803822125	27.1523207057493	−11.3174361504809
P	−17.8529993288016	24.4522653013675	−11.6104816430251
P	−21.2224693241179	26.8036991143685	−7.60363470136103
P	−20.1846285278491	24.2678537126873	−6.66016614470145
O	−21.7872888543005	27.9682900994977	−6.70974110986097
O	−22.14769159564	26.3758284835977	−8.65469667391861
O	−21.1112196819843	23.4829673106349	−7.68393851514951
O	−19.9638463729678	23.5815730978818	−5.40045408928267
O	−16.9573742360263	23.3346734065306	−12.2587298435152
O	−19.2402700212272	24.4660452242102	−12.113416461519
O	−17.6154954344283	28.199368569066	−12.4530515303312
O	−16.1907055623872	27.5511523233053	−10.447550643381
O	−20.4420344343246	24.8589187899221	−9.8681712389345

O −17.0055177007289 26.6753398088858 −8.182159025272
H −21.2155000129222 28.1960803225407 −5.97656152047306
H −21.3462767328652 22.6288532103238 −7.32130117149781
H −17.3694502836741 22.9904888474876 −13.0496579582287
H −16.9239461012568 28.8572041738355 −12.5055694925136
H −21.2675431581788 25.375106590019 −9.68385022641249
H −17.2618307550219 27.4494627178559 −7.66485473412669
H −16.4752251916579 27.0043446332376 −8.96827369401966
H −20.2778391228922 24.7132101976275 −10.8369620397517
25
converged
Re −18.6241291334655 25.5211306329479 −9.10707507016919
O −19.4086838314991 26.9405739660774 −7.8156791262865
O −19.6722003710665 24.2248603467097 −8.1979572927351
O −17.6020062158645 26.8978093005641 −9.89973977344635
O −17.7140803289985 24.2167407190523 −10.3907986472521
O −21.2571383607094 25.5830028015191 −6.86966849421895
O −16.8603243090464 25.8999398443255 −12.0301894907022
P −17.3473739108502 27.2867985103642 −11.3928020231859
P −17.7341100078254 24.5309156844789 −11.8895674567831
P −20.9239561540656 26.9449180402955 −7.72462811462825
P −20.1189730789459 24.4779733133751 −6.73916396582486
O −21.3204260007959 28.0704061385523 −6.69490577522104
O −21.7047707171977 26.9965940939495 −8.9717061296836
O −20.9459447603114 23.2285990521509 −6.26577430828037
O −19.0033071482725 24.8229550584982 −5.83554943461195
O −16.7724057651236 23.4852657821683 −12.5629552142003
O −19.0289430459755 24.6688717798573 −12.5841124132086
O −18.8283289963195 27.4735919915371 −11.9616280918286
O −16.4598792535715 28.3981348060864 −11.6785794357753
O −20.1963610939985 25.5427787126779 −10.4727672227881
O −17.13767589297 25.513037242062 −7.52879296852527
H −22.1598380900466 28.4592178366751 −6.93580978412722
H −20.6099798272976 22.8877026406414 −5.43851884786181
H −17.061513712112 23.2892468607872 −13.4533135955076
H −18.8682435758148 28.2511361877101 −12.5189075532857
H −20.9127567095743 26.185261277821 −10.2534720826489
H −17.6823028090961 25.2312367393526 −6.73993819089438
H −16.9438648929095 26.4484271247598 −7.38016794723341
H −20.0184820192563 25.3515735084234 −11.4312315376333
29
Coordinates from ORCA–job Tc_Maxprot_d_job2 E −82.767675730630
Tc −19.13837785956627 26.03667195756623 −9.35857896083665
O −20.39076320806389 27.23079877598775 −8.27711810681203
O −19.72926566880317 24.63204356736877 −8.26680769356485
O −18.45482396427886 27.33367759187871 −10.50460477256237
O −17.89882761272899 24.70472390036262 −10.26642414037365
O −20.96485924840081 25.67920176909975 −6.42386423588114
O −17.41884731366852 26.00674309606158 −12.30757501163499
P −17.08987654458486 27.13625061042364 −11.22302099663087
P −18.02509154242103 24.58406072300319 −11.77964926816134
P −21.61531560955979 26.51613136132152 −7.69421183877400
P −19.78374219342872 24.65980856078955 −6.72104075029707
O −22.46663833240289 27.58639917244812 −6.92548544199071
O −22.38578889460380 25.63961197931375 −8.57833513803614
O −20.35166648016259 23.27460665224523 −6.23948149014086
O −18.49920513352957 25.02064796121532 −6.09273162814879
O −16.92842163097454 23.55583853529344 −12.25412840701845
O −19.35715424079494 24.31753120458432 −12.34718075611626
O −16.87123979299207 28.39251645727066 −12.14427663656924
O −15.97531827122659 26.83704206239059 −10.31902127981718

O −20.76538122813925 25.70631901521337 −10.65668743349968
O −17.49122125307979 26.42818065469805 −8.06416135230440
H −21.92113560124481 28.24170338910858 −6.48929919162525
H −19.74158369180991 22.84325600983187 −5.64241556362826
H −17.26868308433765 23.02463184267145 −12.97312056701482
H −16.02001064216082 28.79060099098900 −11.96857030707898
H −21.56908729847993 25.47775591439620 −10.13455858396204
H −16.66202796885351 26.21394554491604 −8.52975669987209
H −17.59088102945840 25.91250232814618 −7.22433834388855
H −20.54276466024408 25.04549837140410 −11.35895540375928
29
Coordinates from ORCA−job Tc_Maxprot_d_job3 E −82.767162280600
Tc −19.10026445415509 26.03810651973934 −9.36486632580618
O −20.20171645846262 27.30290263933408 −8.19547312010160
O −19.73221130000008 24.65091187047801 −8.27221824086763
O −18.38258669788082 27.32414788000288 −10.50266364914758
O −18.00998249639985 24.66793369588230 −10.38475066610955
O −20.85410631494347 25.72756162541190 −6.37494156157242
O −17.62525548694981 25.98793074137135 −12.43672349494129
P −17.10985050042067 27.03478021532949 −11.34562646366372
P −18.23131604863836 24.55657316702194 −11.90188049312337
P −21.44366866993866 26.67428607720295 −7.58855337358614
P −19.72839669436506 24.66681220356050 −6.72479181867351
O −22.17580505267557 27.78232091329502 −6.74808292748077
O −22.33486954362990 25.89904392521061 −8.47044817206423
O −20.34844365399026 23.30694646494626 −6.23205698607044
O −18.39906201628234 24.94773888030509 −6.15115346814950
O −17.17473358411083 23.52006830791119 −12.43521826203938
O −19.59473135053366 24.33379517594249 −12.38037578706184
O −16.84289144970831 28.30641866349499 −12.23061718740253
O −15.95617465797058 26.57314035852988 −10.56318167383054
O −20.80157371820174 25.82700692764885 −10.58441484811128
O −17.37433961692896 26.30150051615212 −8.14013274424788
H −23.12181292700441 27.64349035104137 −6.76672617653680
H −19.73209517179861 22.82971772473132 −5.67797581289265
H −16.31419612251794 23.62676038390428 −12.02616969751353
H −15.96971268147919 28.65740394918262 −12.06272666343615
H −21.59467259712450 25.67712231461987 −10.01336705334903
H −16.58082539557254 26.00421876908358 −8.61587301871827
H −17.49726758946761 25.81374532550908 −7.28455620966047
H −20.68543774595366 25.14631441186838 −11.28983410297331
29
Coordinates from ORCA−job Tc_Maxprot_d_job4 E −82.768194193040
Tc −18.82723595319046 25.68452530974044 −9.15094107219090
O −19.84522551056688 27.02345711479280 −7.96691533734842
O −19.50080168565217 24.34625424568727 −8.02479468415197
O −18.19455786106983 27.03360663476021 −10.21365038706057
O −17.76523038028647 24.32125437422986 −10.31283060430274
O −21.33172372653416 25.46314201181287 −6.74461662347181
O −16.57287675106393 25.99387316884538 −11.74520840063594
P −17.18006831080446 27.45254538866617 −11.34804161556591
P −17.63355102323149 24.76559043960015 −11.75406180185458
P −21.33498246315976 26.77894372532163 −7.74879488595538
P −19.97107673291548 24.64729057467039 −6.57220852378950
O −21.87786506740628 27.91351756724386 −6.81328882264966
O −22.17381037834661 26.51097509428255 −8.92271821796171
O −20.45736953974752 23.30100265749363 −5.93266864800005
O −18.91960044105242 25.31953495037030 −5.79533295014658
O −16.85115067704376 23.62965966664422 −12.50356359552016
O −18.86473563608216 25.22168220710584 −12.46266622445006
O −18.09954299971293 27.80740632987508 −12.57345182391709

O	−16.16722907001610	28.41668147600286	−10.98392671606620
O	−20.44671468853403	25.31832264031062	−10.40538290809658
O	−17.24769925649451	25.89860222524990	−7.73864930632647
H	−21.22732919875023	28.20717997665103	−6.17360790209676
H	−21.16210100616743	22.87112234001224	−6.41812411062440
H	−16.93206437932882	23.70168734545375	−13.45367622876926
H	−18.61783961439248	27.02580637638047	−12.83464443485686
H	−21.26650546038250	25.82484273978499	−10.15881245030530
H	−17.68634142911284	25.82069654743595	−6.84670269000811
H	−16.55698705188758	25.22685875329626	−7.80442605248965
H	−20.20578372004708	25.30263811169947	−11.36169297522690

29

Coordinates from ORCA-job Tc_Maxprot_d_job5 E −82.765488575990

Tc	−19.07785618113535	26.06684664350639	−9.33906695757686
O	−20.25678804370028	27.29515384770053	−8.21686862314598
O	−19.68628727535781	24.67104660590359	−8.23986991039190
O	−18.38264102243553	27.33498816346765	−10.50860747772946
O	−17.90958837666027	24.69442157026452	−10.28049187967340
O	−20.87888218204667	25.72970439131063	−6.37959196201757
O	−17.42205828194845	25.98330914515572	−12.32982895148539
P	−17.03560953640524	27.10104431731055	−11.25165445314625
P	−18.06895798439179	24.58293936555684	−11.79211187146453
P	−21.48488676278521	26.62392640575874	−7.62075731019976
P	−19.70832720722008	24.70269626571694	−6.69310262436687
O	−22.27961690620630	27.71673782475178	−6.81748360661457
O	−22.32508594779875	25.79889721826961	−8.50690668221577
O	−20.29499598615427	23.32955259850283	−6.19481915229828
O	−18.40479822772544	25.03571384055424	−6.09161242002534
O	−17.01892257527291	23.51675237973878	−12.28790744312100
O	−19.42057504068134	24.36397576557597	−12.33315627053701
O	−16.79786139928103	28.34833945548373	−12.17997531601931
O	−15.91311771716435	26.76676886963904	−10.37077170390645
O	−20.74638120555580	25.78672201325955	−10.59885847925819
O	−17.39494139688026	26.41115222263475	−8.08459316009142
H	−23.22002092360241	27.55436902774044	−6.87816979593521
H	−19.66727739475470	22.87544981939760	−5.63399351192341
H	−17.39018313204613	23.00115861086670	−13.00293989678775
H	−15.95189345351426	28.75021806334342	−11.98792618662110
H	−21.53989168094404	25.58453445012023	−10.04850921928101
H	−16.57866182975948	26.16637383482880	−8.55689438687394
H	−17.49689563918488	25.91625306829877	−7.23301649869885
H	−20.56499668649196	25.11965421934497	−11.30591423184971

29

Coordinates from ORCA-job Tc_Maxprot_d_job6 E −82.766362738000

Tc	−18.66420570177587	25.72853031153412	−9.19011526763500
O	−19.50925654553751	27.11676321345544	−7.90777266590019
O	−19.76681814856316	24.49417639009592	−8.31605434173934
O	−17.59689623698408	26.95636797102624	−10.04523818597764
O	−17.84813451883473	24.30683395371416	−10.35270442468762
O	−21.02938893945977	25.67123588024675	−6.55456689529138
O	−17.04457644576994	25.79344776830404	−12.15571957998696
P	−17.29131259738661	27.25620182510027	−11.55996706580979
P	−17.95586393950696	24.46234696491391	−11.88350109769092
P	−20.98761528328910	26.91817265101448	−7.62859465880608
P	−20.04534423050428	24.45216842614935	−6.79471030791230
O	−21.45300201399414	28.10849710069129	−6.71191663317024
O	−21.89652962409468	26.64282071112690	−8.75573313590133
O	−20.95269069245380	23.20442633647894	−6.49968336751923
O	−18.81673535538712	24.49526235143399	−5.97527599787542
O	−17.04514249526027	23.35198385848005	−12.51848862570946
O	−19.29266452521403	24.54394122269541	−12.47665347224999

O	−18.69563383113601	27.68803268494795	−12.16270885175115
O	−16.20415112136835	28.17424936317750	−11.84631025380343
O	−20.17709663879356	26.08659176556624	−10.58700187330074
O	−17.12908541332719	25.51629807457743	−7.64911764424788
H	−22.37454606243952	28.31021129299665	−6.86684635068946
H	−20.53579760310975	22.61350823291219	−5.87424389400494
H	−16.19930220959798	23.26991377328663	−12.07605197970060
H	−18.59843256979658	28.49981459771550	−12.66110235879823
H	−21.04441964437024	26.22739701054950	−10.12599535240216
H	−17.60268016447478	25.05663296299497	−6.89805294177407
H	−16.94945726593575	26.40999892094792	−7.33020917035039
H	−20.21122017873932	25.47287438257791	−11.36106362032099

29

Coordinates from ORCA−job Tc_Maxprot_d_job7 E −82.766738170490

Tc	−18.78941331511588	25.73238191737876	−9.06672202123284
O	−19.74324703017882	27.16907598510604	−8.00938532193320
O	−18.97432145361994	24.63512813996817	−7.60540410936468
O	−18.64523224360407	26.89023574091128	−10.54459370274681
O	−17.76540683777176	24.38991225827046	−10.23151803949245
O	−20.88867463416744	25.64947370074328	−6.43412875963367
O	−17.15482336516001	25.79516352324119	−12.18813093110366
P	−17.36464620504296	27.13728172026170	−11.36718320171517
P	−18.01512040594815	24.45867135948212	−11.73839433581136
P	−21.17072445991979	26.81705937566444	−7.54364778479253
P	−20.20558605546878	24.22824099231594	−6.71495452286711
O	−21.65287512751604	27.98547695094322	−6.60758113655122
O	−22.14707605744855	26.43855501555443	−8.56508954532488
O	−21.20923868871343	23.51949320565475	−7.71922323887444
O	−19.93608734598959	23.48551766438152	−5.49796741598376
O	−17.16854768153559	23.32243768200017	−12.41176965543216
O	−19.39808787668504	24.54823383040818	−12.21452648194336
O	−17.71657314851054	28.21476004216212	−12.45531162528713
O	−16.19001470705547	27.49690367440359	−10.54244019132983
O	−20.52662388870964	24.91761402874613	−9.90780867196391
O	−16.98356194517630	26.61249815298777	−8.25856472230066
H	−21.04973803410405	28.17333198270148	−5.88813952327193
H	−21.46630503802449	22.66277795417616	−7.37762316416249
H	−16.33842694848458	23.16557215458553	−11.96078535057318
H	−17.00113406615460	28.83718324116829	−12.57680466978815
H	−21.33644849238504	25.44075565727773	−9.68750205123020
H	−17.20863231530120	27.38206104147611	−7.72115824019710
H	−16.46497116182756	26.94680467735574	−9.04778440917056
H	−20.40646146219423	24.77609831880202	−10.88125719092827

29

Coordinates from ORCA−job Tc_Maxprot_d_job8 E −82.764790282210

Tc	−18.75207740945682	25.76037698231248	−9.01486405267553
O	−19.79353586879085	27.18689270194081	−8.02794306650425
O	−18.95201394698801	24.69740053998441	−7.52977362316514
O	−18.60489736134222	26.89464838765665	−10.51249031293777
O	−17.63984765419612	24.43428037055126	−10.11481963176519
O	−20.94622197254708	25.66603784174965	−6.45716268395025
O	−17.08476682181499	25.80456310358483	−12.12345834908542
P	−17.30997646270657	27.14948080592141	−11.31276589620862
P	−17.85891692020705	24.44550882670375	−11.61587924757752
P	−21.22497395370923	26.80130749880820	−7.59913158163075
P	−20.19734481144952	24.26590778411409	−6.67203968237327
O	−21.76911570295594	27.97511184043220	−6.70488471248645
O	−22.15747198055539	26.37439264361349	−8.64256280190492
O	−21.13575173702817	23.48926413446128	−7.69004055229196
O	−19.94754586879186	23.56877690642399	−5.42442086134846
O	−16.93535285745376	23.33675171400651	−12.23886544148160

O	−19.24202015696168	24.42663509513991	−12.12823068913581
O	−17.64928888398180	28.20308468583861	−12.43010032720468
O	−16.16291907351237	27.54450263137151	−10.46842104677978
O	−20.44016640136074	24.87414485682298	−9.88515331310250
O	−16.99172497760589	26.69710507510650	−8.18019732980522
H	−21.19101170403803	28.19832933781375	−5.97498895395063
H	−21.37089114469722	22.63399905966870	−7.32945632267034
H	−17.34925441818285	22.95113658219731	−13.00975289505211
H	−16.97837589528000	28.88237041753439	−12.48018926455593
H	−21.27329366773088	25.37714760545083	−9.71143228965658
H	−17.23290637694902	27.48315838496451	−7.67494419529515
H	−16.44880996910709	27.00460777546975	−8.96224685335165
H	−20.27752599770390	24.70177640377620	−10.84918403705925

29

Coordinates from ORCA−job Tc_Maxprot_d_job9 E −82.764418521200

Tc	−18.61234500205677	25.74418733626747	−9.13619265113050
O	−19.53315736576292	27.13307460606017	−7.90595934901165
O	−19.70700905978995	24.50003949838286	−8.26692802435342
O	−17.56787474751159	26.99123542498600	−9.98920644433173
O	−17.72912384935955	24.32304437815362	−10.24887483920308
O	−21.06053134928099	25.67382541631542	−6.57336104017581
O	−17.01145324792802	25.81445674070595	−12.10062773631498
P	−17.25763609959724	27.27562394220072	−11.50637581034010
P	−17.83332570030338	24.44967746219787	−11.76906874526520
P	−21.01450884387719	26.90376647614178	−7.66565631709420
P	−20.02854014596026	24.48366945863664	−6.75264564501632
O	−21.53234875571570	28.09842866282980	−6.78339328616942
O	−21.88346959285320	26.59124602991004	−8.81445517780231
O	−20.90579743872363	23.21514876569131	−6.45392288529158
O	−18.82778602226324	24.58657867951449	−5.89923280734678
O	−16.86224161289275	23.36797051411445	−12.36920701415435
O	−19.17452462269241	24.43719676355538	−12.38318644350971
O	−18.66237211227048	27.69306654912820	−12.12607631033529
O	−16.18314495382847	28.20674806436790	−11.79676872141986
O	−20.09701881219261	26.03045419159240	−10.58256207995939
O	−17.11225220439224	25.59482262764456	−7.55849813894162
H	−22.45240733552330	28.27890084383132	−6.97022783259171
H	−20.47385438507936	22.63766695120687	−5.82601844952329
H	−17.20166062725243	23.04399876173470	−13.20275089270556
H	−18.58871837882586	28.55741084919077	−12.53110999841128
H	−20.98014416265460	26.16340206184294	−10.15021902801568
H	−17.59223825270736	25.14800303277702	−6.80524211376508
H	−16.93832008225361	26.49598712028797	−7.25859158102639
H	−20.09419524943132	25.38906878415172	−11.33904062534163

29

Coordinates from ORCA−job Tc_Maxprot_d E −82.769152504880

Tc	−19.07055939091276	25.47244066978129	−9.36163843678431
O	−19.97578953174936	26.85790235329713	−8.19187086993576
O	−19.54964308436664	24.20522580213340	−8.08418146466367
O	−18.61738245621986	26.84144752310156	−10.55780743361042
O	−18.06139932358582	24.21119703910574	−10.61466131096115
O	−21.40412479373848	25.32234808668443	−6.88960050819354
O	−16.95418096940595	25.87765341090442	−12.08795903571079
P	−17.19924604206336	27.10705347327451	−11.11553070600370
P	−18.05600766647102	24.64138747490284	−12.08389461071971
P	−21.46115716991613	26.64958692379858	−7.85849645300123
P	−19.98299464990194	24.63114196681379	−6.65531753127948
O	−21.86309936196126	27.78892346360904	−6.85079247052275
O	−22.39397939993132	26.46522151521036	−8.96916182013532
O	−20.38517889116051	23.31774906949224	−5.89038025001565
O	−19.00995790073300	25.47904162829667	−5.95411642491792

O	−17.26815665143139	23.55489724595656	−12.89616997704275
O	−19.32555847373940	25.05567982764278	−12.68469853907569
O	−17.29256282033509	28.34454854057414	−12.08026479902532
O	−16.17777589794157	27.25729712212200	−10.06058805188540
O	−20.78989217878834	25.06371989175361	−10.50179903588578
O	−17.32523691673768	25.84474115488118	−8.18559521417806
H	−21.18351031705493	27.96020429899049	−6.19668198264736
H	−19.91729470612040	23.25354751445253	−5.05935990480659
H	−16.54134454412315	23.18162349076311	−12.39656717486210
H	−16.67115633705951	29.02667304009430	−11.82900113647744
H	−21.56214383840181	25.61882217678928	−10.23412083354750
H	−17.58782250546863	26.22023678868971	−7.32805653279982
H	−16.71691276928106	26.45086045975762	−8.68430106832708
H	−20.57393141379633	25.12752804054638	−11.46278642211502

10.2.5 Table 3, SI 2

25
converged

Re	0.183905700977355	0.0839601562905277	0.422780649839216
O	−0.107835182916888	1.59548294196846	1.86474229947851
O	−1.04509999694219	−0.987237356371762	1.48928531546673
O	1.41406148752954	1.15311895305592	−0.643820443911307
O	0.475485166340873	−1.4283008806165	−1.01991912768982
O	−0.737972525720838	0.0432242145925064	3.77954445047823
O	1.1068212266131	0.124262237217877	−2.93466096793232
P	1.32052239923538	1.55866357959424	−2.18933411607162
P	0.208997678199311	−1.23415037486432	−2.55215218341608
P	0.15792496268946	1.40235867066146	3.39713090982761
P	−0.950979670711018	−1.39146493395206	3.03489424992208
O	1.6202038428077	1.07839649448686	3.68298974538223
O	−0.429545402705922	2.50335062659524	4.2240176471032
O	−2.23343604527711	−1.96031071645268	3.55151170672313
O	0.288322163227323	−2.25158928659697	3.23499098773607
O	0.794291098756969	−2.33533316335947	−3.38025772994762
O	−1.2531241879628	−0.908166919673361	−2.83691857526972
O	0.0820396273353759	2.4199820543311	−2.38879320112095
O	2.60360894211958	2.12666953400515	−2.70558595698379
O	−1.42866311398214	0.839996906673117	−0.929261064066024
O	1.79780470556387	−0.669966884133781	1.77467272265541
H	−1.45538247441814	0.107488321267899	−1.63777654676732
H	1.2886619899693	−1.38181674507067	2.27111410728211
H	1.82355662469194	0.0617350220761902	2.48392822514363
H	−0.917969019689475	1.5507475482846	−1.42632310487712

25
converged

Re	0.18463296700513	0.0849259674810217	0.422359867162528
O	1.12632282225557	1.4160956903692	1.81003587173037
O	−1.0661828704911	−0.467416397731561	1.78009728475482
O	1.43533512601419	0.637480963214798	−0.935209141523459
O	1.33482897997761	−2.66934797613572	−1.18568882735812
O	−0.447051134939705	0.940683930315224	3.74531637081186
O	0.822304187452229	−0.774512468898805	−2.89887244388332
P	1.14866040363075	0.770453261839298	−2.50236559019612
P	0.190367393525821	−2.00558673490091	−1.94406197987977
P	0.179114559640582	2.17343694009276	2.78952372674874
P	−0.780039628192908	−0.602638031001184	3.34667817144893
O	0.906318045697005	3.06180335272101	3.75561756984554
O	−0.968002377759783	2.83449624600389	2.03254112867308
O	−1.98952476698012	−1.00420203303182	4.13434859047775

O 0.438811465810256 −1.49563736796724 3.51090603024662
O −0.538072795706783 −2.89200176599629 −2.91083101662143
O −0.755918768786042 −1.24523033855787 −0.966038873624641
O −0.0725579520354662 1.6597762586448 −2.66701201602539
O 2.35718689225209 1.17518533586094 −3.28999310062746
O −1.29302821381523 1.60408753557031 −0.256956370245507
O 1.65956979113184 −1.43635249733566 1.10272652476254
H −0.838570628971267 1.84260574197257 −1.10909345409281
H 1.50641630784778 −2.07679506074292 0.335069686427922
H 1.20581190501357 −1.6741110262904 1.95598284831137
H −1.1405317032619 2.2439004745141 0.511719131076884
25
converged
Re 0.185198601540983 0.0845024271667998 0.422348911555899
O 1.12638819368152 1.39385800671625 1.80025973245604
O −1.08089405384249 −0.481348152586341 1.76101285658337
O 1.45084585814649 0.649933876599001 −0.916825400145161
O 1.33075933727775 −2.65867645349426 −1.16526198843922
O −0.461898316246678 0.93166427343786 3.72235031200146
O 0.831102413674768 −0.763642513693762 −2.87847496449646
P 1.15296021205635 0.781735674064163 −2.47709769143798
P 0.190838030355916 −1.98963686722462 −1.92601389243085
P 0.178403078481344 2.15745880382661 2.77196389044895
P −0.785964545993553 −0.614069901902828 3.32148758279795
O 0.905561640050487 3.04254612476116 3.74159244201645
O −0.961021370557444 2.82786746394979 2.01119239956754
O −1.98893018687121 −1.02261515745764 4.11574116292651
O 0.439543822734427 −1.49830814102422 3.48851464430178
O −0.536254076850714 −2.8754566050627 −2.89477458894123
O −0.757340128856063 −1.22530246046883 −0.955223346195302
O −0.0738768947959898 1.66479944447451 −2.64149227016121
O 2.35425643165286 1.19261756654811 −3.27332382649293
O −1.28676001399391 1.57788261643515 −0.256281425168606
O 1.65686681078582 −1.41050090902399 1.10350600817945
H −0.842743180988169 1.8174621049993 −1.11654748730274
H 1.51073902938126 −2.05997336352815 0.341624505260737
H 1.21037218874689 −1.65069136710156 1.96211483109052
H −1.14195287854831 2.22899351489154 0.504407601009508
25
converged
Re 0.186498303911605 −0.224003473779732 0.378628820629692
O 1.09664399578377 1.08015368262739 1.83685107380856
O −1.08561642823882 −0.757908686200571 1.73941985204912
O 1.47428826107046 0.399639465849302 −0.885141017395312
O 1.29073694933411 −2.53177946470669 −2.56102660952993
O −0.576682749517115 0.695996228926181 3.71889164076897
O 0.377072293105947 −0.164327986823614 −3.0840626684237
P 1.21508566393571 1.0002348371517 −2.35594716160896
P 0.0176533376673739 −1.73721074176095 −2.56769428246712
P 0.154343337117784 1.88966663722474 2.77806905611004
P −0.889599406597564 −0.838475077558668 3.31705279285928
O 0.912841067719074 2.72622800305645 3.76582272894052
O −0.952713785057131 2.5895930059808 2.01098728598462
O −2.1231705178963 −1.25562110554885 4.05076947498682
O 0.348781519768478 −1.6962643383644 3.60317813607269
O −1.05215157417216 −2.19671183830683 −3.51797270399225
O −0.574028674159552 −1.50280827752189 −1.12664080600124
O 0.286205267333221 2.20104564040901 −2.21033525834121
O 2.49309174280342 1.26641081412654 −3.08599405869734
O −1.22864167222039 1.32809509874191 −0.287486169394073
O 1.72316511412023 −1.48948839543712 1.48138966934964

H	−0.687409322434452	1.746977135976	−1.03718530999871
H	1.15318181680408	−1.66119314738363	2.3372119928186
H	2.19124556061862	−0.676844363205084	1.73275719372672
H	−1.14462009977806	1.90969634124579	0.535256332020813
25			
converged			
Re	0.502487474327441	−0.0362573579287961	0.380229095370985
O	0.930049814150583	1.33894091681228	1.96904432042885
O	−0.758188723264171	−0.902116811766019	1.57324828704247
O	1.78882437532594	0.683739248374718	−0.811548861131659
O	0.0361084160844668	−1.44845972724224	−1.14028163402432
O	−0.50621214322473	0.283373325796126	3.77570648379569
O	0.475435204658841	0.271750152988633	−2.96493447646368
P	1.92655438590378	0.788221396210098	−2.4279211518967
P	−0.582904842095026	−0.914594536645179	−2.47539625666576
P	−0.285659451268552	1.70031246410924	2.88961164733178
P	−0.685766034057335	−1.19441395868815	3.13012746961866
O	0.0885259386894773	2.71412806023226	3.93137231130268
O	−1.55808282545659	1.95527874532881	2.10893337229136
O	−1.92251013881613	−1.81865189843157	3.68943305088292
O	0.607351211686787	−1.97294255621618	3.41872008226757
O	−0.642044277588037	−1.96297501757854	−3.54314857305364
O	−1.90512382756753	−0.178790352992949	−2.23822718642629
O	2.05418430514231	2.2223350035349	−2.84935560269315
O	3.02774249489116	−0.133542884321815	−2.86171648041774
O	−0.991274866953933	1.29412619470487	−0.395022073314538
O	2.14375270879562	−1.07423156031767	1.63037802587492
H	−1.45096437848762	1.50660045501036	0.466426343237172
H	1.51682265557055	−1.52746546690228	2.34077176874771
H	2.31177138620286	−0.216011742429045	2.0545639415807
H	−1.51467886691959	0.722747918951213	−1.08421389940975
25			
converged			
Re	0.540354384966565	0.187834501462493	0.425708105054814
O	0.867358367362632	1.71763768444233	1.88751638908815
O	−0.694713618616412	−0.636867117886516	1.7083478052811
O	1.7640634204842	0.799292349736882	−0.899462847927534
O	0.102224507204354	−1.43334045586093	−0.861947175745746
O	−0.379139104448204	0.659974458181989	3.8339535402971
O	0.421119283220994	0.0329256894310232	−2.91285884154143
P	1.92375778819661	0.500039628943896	−2.48418089746335
P	−0.613053850087271	−1.06304553087121	−2.20291655479691
P	−0.315791299984082	2.03577804476014	2.85831403776746
P	−0.491809653949227	−0.849237549005974	3.260987557521
O	0.0112381469375545	3.14129318797215	3.81842298955676
O	−1.64782149305888	2.14575565384829	2.13638154819929
O	−1.63087286644076	−1.53870681669855	3.9416264052308
O	0.868379276001623	−1.54400514073533	3.46997652382701
O	−0.747903346295952	−2.24273068152073	−3.11833815024394
O	−1.90364569373546	−0.28116177457892	−1.96996724383224
O	2.20319723883358	1.76562786385644	−3.24033673686163
O	2.91558348751625	−0.614717259427098	−2.64644285000362
O	−1.02194771043424	1.47258674386557	−0.330610010128805
O	2.0597560857798	−1.25437096327924	1.28500873911089
H	−1.47368628881597	1.67556992672959	0.543487589107331
H	1.77272820251411	−2.00822792427872	0.753642458558505
H	1.59466910221783	−1.36679013808775	2.24039919543921
H	−1.51784436536964	0.799985619000188	−0.929911575494237
25			
converged			
Re	0.300909756687803	−0.0867171843604692	0.449142520161776

O	0.954056169406947	1.35396428648453	1.86090907693594
O	−0.752690191333124	−0.970219425140387	1.85213483527193
O	1.45418139731144	0.644700929976762	−0.881827226450609
O	−0.329488174997341	−1.50247259366539	−1.03911899914229
O	−0.733168955750397	0.788284159490678	3.66719724985118
O	0.904512408852811	−0.549441854961059	−3.03166499041321
P	1.98236679398056	0.513733896237102	−2.39755298392991
P	−0.601230536424197	−0.937014900034597	−2.47677834316786
P	−0.111723986509825	2.06625099950057	2.73900955735898
P	−0.513340557478519	−0.788263155369147	3.41473618397615
O	0.477930190927439	3.01917520963277	3.73525111285936
O	−1.27350289344005	2.61463564571952	1.92261402675472
O	−1.52745807269779	−1.51134097620212	4.24665458382208
O	0.938691833496362	−1.20731424062403	3.68669913279341
O	−1.09597690344389	−2.00163606160345	−3.41100746847494
O	−1.43630538367269	0.347557305889706	−2.45706541107536
O	1.87280586742166	1.82986418005432	−3.10742856438665
O	3.32350281919366	−0.160037049472222	−2.43846828153988
O	−1.48094484839425	0.928204756306315	−0.0406962280853478
O	1.97085320989089	−1.41990991597811	1.38356803399964
H	−1.44143856659854	1.77061755699512	0.509076574346055
H	1.59921380682512	−2.26384349745046	1.10587036751762
H	1.61310698892576	−1.27417665802742	2.36584869740932
H	−1.48866216586571	0.89649856544393	−1.09030344682408

25
Coordinates from ORCA–job Tc_Deprot_d E −81.210069094770

Tc	0.18427399845344	0.08413608542524	0.42267660852375
O	−0.11045480117653	1.57639659261229	1.83581409797585
O	−0.99514225544937	−0.95134436410682	1.46139401444603
O	1.36368866238374	1.11953257384657	−0.61604049500567
O	0.47890008655589	−1.40822827420149	−0.99041569799982
O	−0.72699206143787	0.04118286947245	3.76131710416367
O	1.09558990149568	0.12697213963576	−2.91590895899828
P	1.30914945985431	1.54910298023649	−2.15553196473459
P	0.20580793076420	−1.23623726940309	−2.51891470726196
P	0.16267745740986	1.40442245614290	3.36433429508555
P	−0.94052004276154	−1.38096828468413	3.00082120321466
O	1.62554487883122	1.08532805714795	3.65429976112283
O	−0.42829097462313	2.50639515855430	4.18829205180436
O	−2.23779453574952	−1.94449384551653	3.48627081223511
O	0.28659944670645	−2.25484721435048	3.21029811103789
O	0.79672063064965	−2.33826627364070	−3.34281687930138
O	−1.25701232202927	−0.91698854542703	−2.80890823053026
O	0.08207587839734	2.42302769807208	−2.36505482476929
O	2.60647063464808	2.11256767603997	−2.64091689068412
O	−1.42781647944681	0.83513669100947	−0.90882002410176
O	1.79624951271308	−0.66699857934678	1.75412754840894
H	−1.45999556722210	0.10728230023976	−1.61796126805581
H	1.29440779583980	−1.38065888530138	2.24829792163585
H	1.82789863907878	0.06041867591738	2.46372715255194
H	−0.92583587815482	1.54822958163539	−1.40358074177902

25
Coordinates from ORCA–job Tc_Deprot_d_job2 E −81.185424146960

Tc	0.18422450462800	0.08406798136551	0.42267058367126
O	1.08200783016870	1.38479265999746	1.76838781566022
O	−1.02354177400907	−0.44813022605094	1.74994911596629
O	1.39209994904009	0.61637758337004	−0.90456595952152
O	1.34250603163749	−2.68264779430000	−1.18514298987453
O	−0.45354192335687	0.93938874634303	3.72276702149852
O	0.82225046780150	−0.77137173450752	−2.87731605351557

P	1.13725245105725	0.77252855576258	−2.46755806734165
P	0.19396468775324	−1.99693601127627	−1.91620239990066
P	0.17456150924576	2.16501559645195	2.76155027484614
P	−0.76870018146996	−0.60443723914341	3.31293727703234
O	0.93603829197054	3.03790509865999	3.71561634004371
O	−0.97409712618771	2.85054312879847	2.03050645011401
O	−1.99049393886192	−1.01552120924490	4.07729007563105
O	0.44969545981930	−1.49254671036467	3.50037030565596
O	−0.56764440857996	−2.86968940953262	−2.87028267007474
O	−0.71340191178803	−1.21661604095668	−0.92301720445379
O	−0.08121415743411	1.66051143663491	−2.65508788865040
O	2.35901002931682	1.18364488706144	−3.23195486266648
O	−1.27518088797462	1.58549977360922	−0.24405092004638
O	1.64355706327190	−1.41740828510943	1.08938405085876
H	−0.83811203577085	1.82878866540473	−1.10207030174464
H	1.50400314912101	−2.06859849399959	0.33425019428588
H	1.20642229926894	−1.66079826075065	1.94735285044256
H	−1.13546537235331	2.23673730178688	0.51101695048457

25

Coordinates from ORCA−job Tc_Deprot_d_job3 E −81.185583654860

Tc	0.18436540346363	0.08409870527219	0.42279260904803
O	1.07105108576642	1.39974988550173	1.78209860521711
O	−1.01403255498020	−0.44935866513868	1.75977512075390
O	1.38271318022517	0.61741216299584	−0.91430159546668
O	1.35920661583260	−2.68576913966246	−1.20357332581271
O	−0.46442694381383	0.93980684916931	3.73728087471923
O	0.83266716967645	−0.77161354061927	−2.89185113153553
P	1.14167500989356	0.77315513160983	−2.48375393511170
P	0.20810803952956	−2.00442762873620	−1.93428226964758
P	0.16010105037992	2.17259525430230	2.77978878135544
P	−0.77322679127049	−0.60507134655082	3.32926513515913
O	0.91871869369555	3.04458707264596	3.73670518110945
O	−0.99099518144619	2.85379299381944	2.04890683917035
O	−2.00136097965787	−1.01813347485128	4.08191188409868
O	0.44465203452956	−1.49159225637732	3.52082091051972
O	−0.55067758436304	−2.87622957566519	−2.89122611634341
O	−0.70267854738916	−1.23165227218341	−0.93638986262491
O	−0.07609909141951	1.65987409721266	−2.67513672987945
O	2.36973310567475	1.18615038396988	−3.23659211762508
O	−1.28643364310722	1.60387510647188	−0.24542437775383
O	1.65531225195752	−1.43581613207353	1.09115074701824
H	−0.83837277923379	1.84615519839239	−1.09562431651689
H	1.51139601184262	−2.07611203199462	0.32799873929482
H	1.20574402963458	−1.67978560608770	1.94004265077804
H	−1.14093958439825	2.24540883387841	0.51641769906010

25

Coordinates from ORCA−job Tc_Deprot_d_job4 E −81.183188682990

Tc	0.32410929653792	−0.26843790934086	0.41395857083601
O	0.98752530533497	1.15642923079508	1.83057280030745
O	−0.92177306720422	−0.83734912900610	1.70389232116347
O	1.60205571767370	0.45280419669221	−0.75683298207002
O	0.33096830392309	−2.84409985114138	−3.23067494771825
O	−0.69656035770987	0.68001326663926	3.67134881838358
O	0.90439172628277	−0.39935924673403	−2.99945424127689
P	1.41299362159441	0.94243723793748	−2.27426950764731
P	−0.21341887114826	−1.59577414390621	−2.59208499415505
P	−0.04968741151042	1.91166600420198	2.71009588430458
P	−0.83479660050626	−0.88132079147359	3.28934082699873
O	0.59603379994540	2.84111302612999	3.69567947448272
O	−1.18650816937560	2.48025396170863	1.88474329231214
O	−2.06596566407430	−1.41043203595699	3.95063706902304

O	0.45878881369272	−1.61226702235556	3.67079400769628
O	−1.56842502960225	−1.15957672008324	−3.06024991031756
O	−0.03723259829696	−1.67567063147371	−1.02068760401788
O	0.33485992190009	2.01418017781942	−2.28822334164775
O	2.71937949825791	1.31889210068824	−2.90194476572122
O	−1.16794772429948	1.07976249613473	−0.37441867531879
O	1.93939932677567	−1.33183594954455	1.63809817612731
H	−0.66462988135508	1.51345319834079	−1.13613120666668
H	1.33317375581759	−1.53519182448193	2.45916275120662
H	2.26994347250259	−0.45344329323652	1.87966572431029
H	−1.20047718413381	1.71485364636463	0.40378246368143

25

Coordinates from ORCA–job Tc_Deprot_d_job5 E −81.181563246880

Tc	0.50778438563457	−0.04369651227286	0.37717111927533
O	0.91707651114690	1.31174171923214	1.93574716752869
O	−0.72629075697064	−0.86245386960535	1.54978290780558
O	1.75492766966293	0.66200414614896	−0.79070801353438
O	0.06025629240804	−1.43582361891492	−1.11077577406935
O	−0.52368105077394	0.30261341840306	3.75410158669951
O	0.47395459045804	0.26165416701785	−2.95373338230684
P	1.90485243939135	0.81117236932757	−2.39787882367044
P	−0.57007655299645	−0.93049234036211	−2.44921027334203
P	−0.27494300559813	1.71293661882461	2.86503811362030
P	−0.69504420466598	−1.17183831643765	3.10269995453951
O	0.13438841260802	2.71781761848376	3.90238153606213
O	−1.55005534549328	1.99912160094574	2.09951320530804
O	−1.94849541846506	−1.79090074505895	3.63015570870546
O	0.58555964074653	−1.95879974496334	3.41425948077973
O	−0.61436283977794	−1.99033549416566	−3.50723486561651
O	−1.90113268596642	−0.20999118111159	−2.21782293482220
O	1.99500494701793	2.25677724389253	−2.78846517113990
O	3.03307306862573	−0.07000791251163	−2.84698003606627
O	−0.98295912362742	1.25880951324098	−0.37252297574234
O	2.14051433180403	−1.12107577422957	1.60687951729013
H	−1.43547923993636	1.50233982043699	0.48418785659476
H	1.50791120723579	−1.54688666854772	2.31735852139291
H	2.33135375720282	−0.26552557511794	2.01905427791423
H	−1.51793703394049	0.70193952793816	−1.05619869892980

25

Coordinates from ORCA–job Tc_Deprot_d_job6 E −81.181471351110

Tc	0.55624616418580	0.16685377094339	0.42941021159473
O	0.87326602098920	1.67116257366498	1.85721821929090
O	−0.65325446001276	−0.60537168436425	1.68088409290643
O	1.75056843552670	0.73306560609710	−0.86895140633840
O	0.11390473840517	−1.42473914925655	−0.83207159827462
O	−0.39513291519039	0.67619424723024	3.80924368291886
O	0.41634994242516	0.01467906482438	−2.89963850160765
P	1.89607593762566	0.54519797706142	−2.46877596595935
P	−0.60452506551779	−1.08306787399328	−2.17910842754986
P	−0.29149492905186	2.04282621332672	2.82548147924540
P	−0.50494893758512	−0.83266240029524	3.23720489783009
O	0.07144803108724	3.14469332332242	3.77677245771051
O	−1.62300113726202	2.18881243157623	2.10865555473956
O	−1.67590931192676	−1.50713021394692	3.87792485167593
O	0.83719110902204	−1.54244388499504	3.48731131755332
O	−0.71857134371297	−2.27628304814300	−3.08052296522679
O	−1.90475525121257	−0.31678282514106	−1.95549512050975
O	2.09331771691477	1.86639467321490	−3.15209941194577
O	2.94196826907065	−0.50207716138191	−2.71884998645484
O	−0.98969099762039	1.43236804175496	−0.32176047369567
O	2.04872746871844	−1.30080210941071	1.29977795240077

H	−1.43952815265817	1.66916361350450	0.54421816259900
H	1.72711990958483	−2.04539566037326	0.77851540646363
H	1.58458796963861	−1.38740323434490	2.24350811593806
H	−1.50375921144347	0.77384770912489	−0.91205254530452

25

Coordinates from ORCA-job Tc_Deprot_d_job7 E −81.170911171290

Tc	0.33626399785065	−0.08715676187774	0.45973515321162
O	0.94882125556832	1.32250556162864	1.85757319966913
O	−0.73237415757145	−0.89906391879703	1.80915299842186
O	1.47276355533642	0.58486721946933	−0.85193009003223
O	−0.27645801391032	−1.49888805646656	−0.98544244505574
O	−0.73589489760164	0.79574265941182	3.66098482330550
O	0.89781171685629	−0.55271253771280	−3.00835941701741
P	1.92989108747671	0.56451290268154	−2.39219389991672
P	−0.58653115901154	−0.98726426559170	−2.43147577813666
P	−0.09060754671587	2.06675251436796	2.74157947234674
P	−0.54417601991062	−0.77920474178574	3.38072661937098
O	0.53238095379948	2.99307063338869	3.74347449892709
O	−1.23652021953424	2.65155622576411	1.93290928741309
O	−1.60347251992156	−1.49751684602143	4.15931473102275
O	0.88622154968607	−1.23691690185312	3.69119660698505
O	−1.04502823423416	−2.09330696735813	−3.33550312385709
O	−1.47159086216744	0.26374695546166	−2.44016865421773
O	1.69230693165260	1.89733627079448	−3.03601864097332
O	3.30925921561378	−0.01165390833367	−2.53326459785440
O	−1.37026902941584	0.97077526809367	−0.07081878091389
O	1.95783787136249	−1.46770710911092	1.40171759940570
H	−1.36450772263332	1.82287437696394	0.44619150139213
H	1.55169007726911	−2.29092627862302	1.11494402867933
H	1.58915642299888	−1.31327030746892	2.36765959318454
H	−1.44077424652867	0.88294799181739	−1.11518467579227

10.2.6 Table 4, SI 2

25

converged

Re	0.53175589091593267188	0.54945177854140592189	1.15575751076269650497
O	−0.52059898018306804968	2.03375257866803771378	2.38876503407741669349
O	0.68016534702782027733	−0.47970915254137164174	2.75858525959985678710
O	0.14445591583614955655	1.57708306268088738200	−0.44316137040788416979
O	1.79522944983000121155	−0.64416323326672231264	−0.10443360237760546116
O	0.99700580489925760830	1.48729710946493720591	4.37074569764667586469
O	0.79460482701727574995	0.03405734837923552610	−2.35162159997559916391
P	−0.28316278090156377090	1.17902362221557588384	−1.93145231102964132219
P	1.40985987741396634476	−1.25841141538158751700	−1.49129040680331126367
P	0.81602632071248482060	2.44199095433906210317	3.06183483803503131426
P	0.86765130386987043831	−0.18029301375666800800	4.32493343089900861287
O	1.88095056021391471290	1.95598693064257145302	2.02628556731827824677
O	0.94806110836297130540	3.84365887518232707265	3.55688401676191290335
O	−0.36483080592103722672	−0.55264238241950447428	5.09083359073467889289
O	2.16822881686473589724	−0.73024196043787825694	4.82525863630349682865
O	2.60086158817390389331	−1.72143215405032101728	−2.26602406881936069283
O	0.28004994859014059605	−2.28336884774033643808	−1.36138049696611229678
O	−1.67462637210547304889	0.55429536532950340888	−1.86537343267334687624
O	−0.12274455386690660830	2.30784629260403217899	−2.89516331964087836326
O	−1.13209033945622405959	−0.86024002375930552766	0.32265551186045304810
O	−1.99318148728917110546	−2.02104978141994262586	−2.88537549222001921123
H	−0.58491701357157899199	−1.51745594187685695076	−0.21274625395563151464
H	−2.00528641390698236791	−1.06930966317388564946	−2.65931434130305710539
H	−1.15520175123539892148	−2.26785598565951040939	−2.43511351984355606248

H	−1.4720662549769116814	−0.2771703678458982689	−0.4132888842908108983
25			
converged			
Re	0.5709415342525796521	0.2564851149302836419	0.3429382665816064212
O	−0.3588095979311313743	0.8568631183645838422	2.2896796260603906603
O	0.8546043874844413412	−1.4320286554711532112	1.2160927900274358659
O	0.0350556425583899156	1.9153434460593903310	−0.4778343882389795571
O	1.7372311235792252670	−0.0667505213137159075	−1.4101912758690779981
O	1.3967009810055546204	−0.5144979907974365218	3.5460284821358079021
O	0.4497061465411609226	1.5017507056153278632	−2.9511318445221337292
P	−0.5610176899074383882	2.2683553239075702201	−1.9298259842258096430
P	1.2094029515415158915	0.0115193752060451376	−2.8889690487495092163
P	1.0554421077927482741	0.9582595313300534822	2.9242092764888201195
P	1.1516264535065596774	−1.9321637342612296706	2.7050131353877873152
O	1.9890643543872756904	1.1129171240879265347	1.6846313243865704834
O	1.2529833044938318842	1.9309305159682494945	4.0361581285818752817
O	−0.0701418390973268524	−2.5887939125475032398	3.2933488285947696283
O	2.4239558050949003842	−2.7141604976957647288	2.7822473054134131409
O	2.3248335510448487895	0.0985748155199025893	−3.8829100989646745389
O	0.1570453136293993301	−1.0512276948447800695	−3.1728871818860922005
O	−1.9449528578832555592	1.6483124397366140101	−2.0194276134426796254
O	−0.4677742428881901948	3.7272676233417958435	−2.2411683707096390350
O	−1.1404800236491365872	−0.6416031949308854676	−0.9521561932819464502
O	−2.1266123171727500107	−0.8540434253988150592	3.7870954269047989448
H	−0.6337465988739121591	−0.9192079153311798478	−1.7862506443778023435
H	−1.4438378251034800214	−1.5511237618570445917	3.6511075424935781619
H	−1.6987965440598538080	−0.1304994398983876369	3.2959258099836601374
H	−1.5562241193236245884	0.2106216155814779611	−1.2749232944959292268
25			
converged			
Re	0.1896289393606115925	0.1219685179829178151	0.0675259755113395976
O	−0.3195818710755102820	1.1059240637775156201	1.9954905202870076852
O	−0.6264423019412772575	−1.4365582030316019768	0.8289047176221865376
O	0.8064089471531591391	1.8017637930700935999	−0.6541271906811132463
O	1.0300196919660591365	−0.7586791714814842269	−1.6885878141897729953
O	0.1313431471377484727	−1.0779977744326427746	3.2532640484495840560
O	1.0046962849125244954	1.3359487314970222016	−3.1430859968425739481
P	0.6090043338480620738	2.5088889380550094543	−2.0845418688402510554
P	0.7260201428469412743	−0.3146488111522838249	−3.1662417996195304503
P	0.7945181283615001532	0.2989339738254266354	2.6937689196061112006
P	−0.7701456642625207349	−2.1078833299464196926	2.2747912281888411634
O	1.7477683914399793074	−0.1023469887110680792	1.5328827905713873214
O	1.4455726054534796531	0.9266196104244963649	3.8961044136308515817
O	−2.1836383758952182532	−2.0436793326988547114	2.7640867974231304593
O	−0.0910755335623994744	−3.4410727596669401506	2.3281870210419328692
O	1.7197097202758844236	−0.8661873465221781032	−4.1393157337657990879
O	−0.7375579984425559843	−0.5239301265850038191	−3.5314492649233772958
O	−0.8652650360999241119	2.8497057303223174429	−2.2289883681973949869
O	1.5696365021655589444	3.6332152442024656657	−2.3009424535821616508
O	−1.6424473003657633629	0.4893232402689324800	−1.3153013531845634265
O	1.0540136062626930257	−0.5719071950068487231	6.0607917639665336295
H	−1.3594977937073129403	−0.0012869395483014745	−2.1590871326345686398
H	0.5575517328337015188	−1.2211100739958724581	5.5496562074745270365
H	1.2603103618192346147	0.0623766064889556548	5.3200644695127987660
H	−1.4443506647540937937	1.4337195975821306337	−1.5870498978406626378
25			
converged			
Re	0.1037701818471488679	0.5797963924853851702	0.5868084706200448197
O	−0.4617920177791855485	2.2831874757235164352	1.8775579273472116081
O	−0.8658618171042092859	−0.4984445529870757396	1.8387125692674251009
O	0.8983400584873796335	1.7619213072246180384	−0.7102027759585058941

O	0.95936246756893017729	-1.02797479394764756933	-0.55807783629634877265
O	-0.25022367164941283768	0.81543277339036068785	3.95307383065796669186
O	1.18951628328051617345	0.27755707355117154034	-2.74365143783086429252
P	0.83869575276839913869	1.80811485389050430328	-2.31654591358087769137
P	0.77111533789552377804	-1.21440598151864120879	-2.11248950603456631114
P	0.55867949402771321932	1.82495106913382665326	2.95188012090399531928
P	-0.96604786318486801644	-0.59659149714097536865	3.43319207905893053479
O	1.54258236677887006216	0.92684912997789381528	2.14005890475371129966
O	1.15556775626734942186	2.87450432679173628614	3.82875005639310517225
O	-2.38488826359686267864	-0.55445901114231199713	3.90845044646662742593
O	-0.12084059120170217994	-1.74143699338763946471	3.91654153905098212363
O	1.75936530777187960517	-2.17226115490239468642	-2.69458070140250427471
O	-0.68642037719297399612	-1.46670983434810420221	-2.47280068639082983850
O	-0.59031777579691724345	2.13909051084176793367	-2.71508962706270162712
O	1.90209071560042652926	2.68149599902062485413	-2.90079722308671295039
O	-1.62919263489653198818	0.42613960587113719480	-0.97756602503431611062
O	1.38449751775900264938	-2.94774561738550122669	1.84181787772368577016
H	-1.33865770031619035763	-0.38932993453058611921	-1.51200078766013024101
H	0.87324169255399308742	-2.47437592870262701439	2.52933799605900722085
H	1.29446280690103199262	-2.36847983479389068151	1.07003094230100459505
H	-1.33084502576698038290	1.15427462218617726819	-1.59561023069732965673

25
converged

Re	-0.27065939430375696162	0.49211775137023650029	1.12686835341484337114
O	0.03332317600638559640	2.44726382942423725808	2.04066324232706453046
O	-2.01091583623820424620	0.53255179075047343051	1.90555389281283327207
O	1.44878576647942236555	0.63549745897357001745	0.22716694996673064688
O	-0.27687513216660286641	-1.52233991163040394134	0.43433722168835348088
O	-1.33924064785166718750	1.73079754605897440101	4.07036693819430794861
O	1.29060764593192911498	-1.17875933169186186689	-1.54927229542748978730
P	1.85797205225324479905	0.32043586083502523243	-1.27704736445479882789
P	-0.11448592093086149002	-1.95776790845304393685	-1.06186907757421900023
P	0.16329058895889081438	1.78952179306627279232	3.43991848756172169388
P	-2.65833000793816864871	1.09867192861179074903	3.25862279988070202563
O	0.53829936042620929459	0.31491549044914624034	3.08927177256327833987
O	1.00755375671223035283	2.47920535803055219759	4.45913542382715188950
O	-3.58774968527548976382	2.23779593455759417964	2.96799365982558605026
O	-3.19703217591869615077	-0.01381316441577935816	4.10550514551506751104
O	0.19812069518180763783	-3.41391747837166237289	-1.20852093615514899483
O	-1.24125154046439689637	-1.43059573681867679973	-1.93919655701293369710
O	1.16201467440602934644	1.31138067463162744097	-2.18990363842407065320
O	3.35148923482255067796	0.22195791402292278094	-1.44874800659888536281
O	-1.15552855623620498449	0.96542923066989272396	-0.94816818499145427701
O	4.02189469278589495360	-2.27047488753792947591	-2.21164114796680566499
H	-1.30526229105107183237	0.03302862734094097474	-1.32588934025217852763
H	3.14076037498929272473	-2.62036755325688908158	-2.03197702009914982213
H	3.86596230481278757551	-1.32769382358679233214	-1.94026002307584666262
H	-0.31654313436922154601	1.22625860697934196963	-1.42611030185196963060

25
converged

Re	0.19691527018740645483	0.12347638787204939792	0.06400762740818127794
O	-0.40405360703915804699	1.05825082690309280053	1.96090607005632810989
O	-0.54592546859645163515	-1.48605843846775576012	0.78561553840474873667
O	0.74892791862947660775	1.84040491581202458882	-0.61796034778722397363
O	1.12068870634210604464	-0.70055112217378445738	-1.66007927009910338789
O	0.14936728250150829722	-1.09410910205506928250	3.22602514896346814766
O	0.98619010750259616938	1.39386643393448905748	-3.10642557750792658311
P	0.51963641500031376719	2.53740243319538683053	-2.04320962000574102291
P	0.80567260435816867581	-0.26915741002491999634	-3.13774625777575444374
P	0.74260681552889207246	0.31982302006099905256	2.68315145246236586019
P	-0.66933884304162993839	-2.17020963746177164566	2.22369357692555169592

O	1.73945014913851236926	−0.02147449114878541288	1.54187859078915145972
O	1.32773031142349573486	0.98389223195214692286	3.89982610909507787511
O	−2.09198023015145384562	−2.19612335038836192780	2.69125669366788233816
O	0.08922729449959322656	−3.45993280450781170998	2.28334164204569267653
O	1.83760773077943850851	−0.75739877929948584701	−4.10495532096063708849
O	−0.64037289906465610922	−0.56146094039261351671	−3.51880877450456441835
O	−0.97131833365784558953	2.79540302576769184029	−2.19907279840255398540
O	1.41457142518044021173	3.71639901924248761134	−2.25270672640272717757
O	−1.61389913556146424867	0.39665846686140415756	−1.31435920843237874500
O	0.96658394566326355513	−0.53330984648168977547	6.05516657354522269685
H	−1.30392943829263741051	−0.07332461529175721138	−2.16236610172363086235
H	1.15562308599048302327	0.11175528446590113352	5.31899806960015286705
H	0.51812732870651490025	−1.20769434540700393299	5.53247346235870374187
H	−1.47190844029635226065	1.35457283704269326741	−1.58185054744404651750
25			
converged			
Re	−0.14212489276875489574	0.12936440562416157141	0.34105708809603452192
O	−0.19170082866456694681	1.46109385454587403785	2.12621236916696076946
O	−1.64258173444356714299	−0.74171284898891620685	1.15052245558949595150
O	1.24110148769175787642	1.20721539858475246376	−0.46579810862030252006
O	0.23257719249914252102	−1.25122954069184166848	−1.24517547039969578293
O	−0.90478055749704489052	−0.51257951464940698649	3.59155551797587868634
O	1.26788011260999811114	0.41080475568357333049	−2.87812321150391881019
P	1.45515032414616340972	1.74694852422275714865	−1.96553423521583292555
P	0.23000281568015357170	−0.89875742692014215507	−2.77788703319744412212
P	0.35770491761065220659	0.31025587453758862733	2.99480797909659601785
P	−2.16681008620603510195	−1.06347205581665660290	2.62984645181242848722
O	1.06182563515371675145	−0.62821150105426581955	1.96581243726493060109
O	1.18657950525761535232	0.68480027909144558507	4.19244540281835575968
O	−3.37195814149839456775	−0.23996177496624246372	2.96517997993188009431
O	−2.26545562744466977279	−2.53944867664742979230	2.86091785358727701549
O	0.86724532241439589164	−1.96956570242261519432	−3.60586000134787498794
O	−1.13989529158766744743	−0.43046479952238270172	−3.25034885709537757492
O	0.33351792448854272743	2.72863069145955750017	−2.26499475444922104828
O	2.84490087741167307911	2.24331643977518924160	−2.19938538898256474852
O	−1.51451676475293406376	1.15197751080597710072	−1.22229903720716381876
O	3.42393568576379303536	−0.77130721638726706946	3.94908536130070819681
H	−1.47641646277900329132	0.48800962380868662116	−1.99045183758043542355
H	2.63713167679112991948	−0.20530164814635457060	4.15684685927376396108
H	3.15865594077310474219	−1.05752990952022174476	3.06731269970937292513
H	−0.87576901904332404847	1.84822525760373523873	−1.55894453162293022963
25			
converged			
Re	−0.04163047981235296069	0.37926634694299071748	0.67878215085198356782
O	−0.32196039942601406558	2.09131284162131603210	1.99301425977424373492
O	−1.36101204094678451817	−0.49699427785122796131	1.74422198143592321884
O	1.15238795764519408316	1.40738988028989586709	−0.43688741481304660397
O	0.60244830728948117748	−1.30126643844568201658	−0.48232973711998272659
O	−0.66824195652713513915	0.53383049740885124557	3.98704201263642143971
O	1.29890504404936124594	0.01184155738591712026	−2.55150838539283775219
P	1.25124803398335293636	1.55633599747741624419	−2.02788013154007185079
P	0.51419778803400284506	−1.37349329234776407205	−2.05400264128373777339
P	0.43587870818348573021	1.36923126893955404704	3.13471815952241739112
P	−1.82483920006059441299	−0.44915723695441978069	3.27594796301075685818
O	1.27020345719780247684	0.29293067098373531953	2.36036890045595448839
O	1.17661738170926155611	2.20762735149909516963	4.12135969859181194863
O	−3.14605023463425093055	0.24472308171383017061	3.41170480082048444004
O	−1.69639460024867161358	−1.79341904412191088802	3.92417587090926556215
O	1.31739356072054536462	−2.50623122335110437575	−2.60867110232892285282
O	−0.92538682596842891659	−1.28515415335061300439	−2.54073449605722601063
O	−0.03038060622857427506	2.21522504182347335089	−2.51568444010413339029

```
O    2.52575190541759164731    2.20228505915705330409    −2.46627507877369023248
O    −1.55912966853115153576   0.70102213151236081323    −1.00325748793625391109
O    2.68832259098163506295    −1.97124873012382706428   1.33900499521834381866
H    −1.41629654034487484360   −0.12837682811443529296   −1.57447781485159787884
H    2.30781407887655998223    −1.24077066397243318541   1.85301284370110752597
H    2.11275063806011420553    −1.94542679042876831552   0.55327354743871104414
H    −1.05639689839721584441   1.37961694702448234295    −1.54811846047323031783
25
converged
Re   −0.17671296871255101624   0.09601363687855062057    0.68302686843594462296
O    −0.14349895466984666226   1.60394589348842497856    2.25437809955034795806
O    −1.75051024843014091381   −0.57148576215971813586   1.52996241698321000513
O    1.34382251812484154740    0.92372235234930799574    −0.18963710733420302557
O    0.08483255986639474944    −1.48109954456035097792   −0.71894533233524371418
O    −1.00527933215199882788   −0.11727952151775679346   3.93651817797962078060
O    1.27543338269146566688    −0.11541320227176145308   −2.50690211751159885978
P    1.59987304096751636884    1.27921266874669292690    −1.73338350266641638697
P    0.11136912518495609148    −1.29684165294668019008   −2.27788304644996397741
P    0.32672867558223261941    0.55349533302352693642    3.29309605490613765966
P    −2.28970398604771263606   −0.69662695908955618584   3.03040424216645387645
O    0.96609431504146470182    −0.55280245280874207303   2.38515381532289438837
O    1.14693197587884454514    1.02884461153639228392    4.44355009034315706629
O    −3.44685015332989097558   0.22781490363294409907    3.26044588579008820517
O    −2.48083176516411141321   −2.12969864296616728794   3.42397395990045794179
O    0.63250985724378661068    −2.50498356780025410728   −2.98922581913158680322
O    −1.20548162904355704583   −0.74293751279650133146   −2.80950104500768826910
O    0.58828423386519357319    2.33562691852555559535    −2.14667083735664299127
O    3.03630125740627931918    1.59967727760920697122    −1.99437510710915910650
O    −1.40928336635788120645   1.06916975748539044844    −0.97659755811739779041
O    3.57362383354583323936    −0.21628829411027972185   1.34939074992596763636
H    −1.44290206125118714020   0.32938979583389427219    −1.67723796264684432700
H    3.23442493978354850270    0.17843545061654653150    0.53282237411795219106
H    2.74257014358225426065    −0.36052499379545399627   1.84233989117183050155
H    −0.70554538199985594726   1.66173351239811450952    −1.37790319194285104665
25
converged
Re   0.34564833652680942988    0.73002150316780478612    0.14170260147847957288
O    1.90399397940383940941    1.42989537304882396995    1.40729980203838911379
O    0.23589086318853902391    −0.83799137271858381570   1.30422220307407465967
O    0.18786210407604270189    2.20940002664691892420    −1.01909111640786775865
O    1.36543155891769130150    −0.30050782756596866596   −1.44011995354171018491
O    0.85203357710660876378    0.36397874465707891201    3.47628884639594248895
O    0.71591250048755350477    1.27286545716153831620    −3.32695945218103084073
P    0.04290339282109439933    2.57863114976621110586    −2.59107522970742376600
P    0.82740730691294861465    −0.31389613864595095061   −2.91202177893006242115
P    0.91438802799132656851    1.73419052477096680143    2.58050860888935718052
P    0.30491401589806577110    −1.07690447809389744371   2.86894457918396916796
O    1.30929435729082621620    2.81394450830301101618    3.52941501258897449844
O    −0.44556414922764242537   1.85238682175822266807    1.85356641966152047019
O    −1.07027110556007398756   −1.33594162285720763350   3.43142275615962821789
O    1.34019985636263605500    −2.11204397148114431104   3.22903157472357138502
O    1.79459065030008391162    −0.92195748585853398716   −3.87705036101780553537
O    −0.58469138226398342795   −0.92135885353774704676   −2.97213869683076969963
O    −1.40177695645756839049   2.69737777021828817325    −2.96501926593571907276
O    0.92550800533447152851    3.74304488285977443596    −2.92538684681415706024
O    −1.57094857898889039838   −0.26080764330330785672   −0.78431044865060772775
O    −0.31997195797138078266   −3.56196897697915915160   4.80425268538939498342
H    −1.48500532416922026435   −1.07196150356506958445   −0.26966592077703221975
H    −0.88160765075880775932   −2.83508009773883085103   4.46021043071494105448
H    0.48711953912045469206    −3.26488404853776925663   4.32932160708726065934
H    −1.18706095473554729125   −0.50933274675768691608   −1.76654805760685240656
```

25

converged

Re	−0.17473124927571623277	−0.29717426778806660392	0.70623990074883835621
O	0.82529932685535178916	0.63164308963254012230	2.46732250844110145849
O	−0.35029128315182656017	−1.99518146611045432692	1.71606642100684192442
O	1.49079089839467293643	−0.31302031044311340313	−0.37144250184460658737
O	−1.14383626887684819451	−1.11652195753108962606	−1.00318857717838838539
O	−0.59990693729192401396	−0.83145451794527303058	4.00945283860403733911
O	0.53159107471375199871	−0.27945243278898168615	−2.75202029256710467564
P	1.79375661736570823912	0.21040795518185018143	−1.85138830225999573287
P	−1.09733897057649198992	−0.49839717007043116137	−2.44620216304886950454
P	−0.52473424225024423162	0.57832829846733413426	3.23197859334099213413
P	−0.41976148146770830838	−2.33583491402733889331	3.26866767759689880180
O	−0.77808292706509019077	1.63995619013029214628	4.24777597420643182602
O	−1.56696640876934045750	0.49258316058048567943	2.05125891217816214152
O	−1.67340467037436457787	−3.09647800855162858014	3.58317560827971259840
O	0.86409700420175417079	−2.92463504541736751818	3.76936798328702149519
O	−1.57523488080653817356	−1.45619363105116494950	−3.49046040747388586212
O	−1.74514328037061550170	0.88088372623880495382	−2.48039072103152813398
O	1.79849475650941692528	1.73431538022319475800	−1.77396679117914013446
O	3.01883344123576646822	−0.41479662597560457149	−2.43230470025051515037
O	−0.46268065728357532507	1.73866025443333516165	−0.39575204640399630485
O	2.60025051361087200519	2.44739478463777482276	0.97318975418499020780
H	−1.05803206319081044562	1.44021516458873666267	−1.16384946226129626368
H	2.02049826577112545678	1.87765002567740157957	1.50821555673985785440
H	2.42295875158719686837	2.13969528007818032833	0.06715189589826148220
H	0.40977466623604341667	1.84850703254836723488	−0.87209765473758693588

25

converged

Re	−0.11776674324599076260	0.27876682737727637162	0.64525385470191010118
O	0.93219910366055824813	−0.34513619201946871273	2.38900491497828593168
O	−1.73888638323089894655	−0.38219458009545276278	1.45588763437391843780
O	1.33594113574628647889	1.08326914587021305714	−0.26370541692799287503
O	0.07363176575908507437	−1.34843251253697338221	−0.75473365077422627234
O	−1.09238083639515548562	0.14624869732333584116	3.86670050499341577677
O	1.17181106818126368196	0.04727625843025318186	−2.59848167400029161911
P	2.15431100080813653364	0.94074820276751058845	−1.64556908966517312365
P	0.04275430844486877158	−1.11966443727208719849	−2.30013166778705135940
P	0.27884794242616239845	0.78676409573132377595	3.26372583366918789238
P	−2.39478967219443594772	−0.28699742316464127434	2.91090795612941022341
O	1.06191592944153589961	1.25809943856309325305	4.44014694933087294970
O	−0.13407439374184540459	1.82412997928447850882	2.19242811876303678176
O	−3.40768815097161237304	0.81932846973524764422	2.94596181226749820325
O	−2.84757299498198745979	−1.62827732960669169238	3.40242376552355496599
O	0.40687245123707294070	−2.33431158168442864920	−3.09002892732088740857
O	−1.28889070380921100245	−0.47656341545743530208	−2.73777153112336701923
O	2.31529654749437296957	2.26260827224256910739	−2.32905665699689112813
O	3.41515701887280398452	0.17481037032324148184	−1.35146704265992934069
O	−1.68649337975728741412	1.14702394829140508214	−0.89959620213309843262
O	3.68685288091053298132	−0.81854629574813841941	1.23778487544490056749
H	−2.43095765664449814381	0.76192417667595269126	−0.42222174096767139950
H	2.80929366401608460180	−0.67265895827577337762	1.62329031292788750207
H	3.56462710335745391887	−0.49126833505427447424	0.31961279647677598437
H	−1.50381100436096493134	0.47415318889256596879	−1.73356573023961146873

25

converged

Re	0.11432320640197735084	0.49637999447106107009	0.34203801858306598360
O	1.54623769285996992551	0.38855071033438876027	1.94278687365307489721
O	−0.95247715885836292671	−0.72203123953260339718	1.41822489586211930579
O	0.94650010769768422314	1.76701796155205648198	−0.77887765220464966198
O	0.70005658372113388488	−1.00367438472330627874	−1.07623161064943251297

O	−0.30308246799789773096	0.23101280849825228669	3.70633171339657607746
O	1.25273549508457882062	0.56539001798103183383	−2.99794434131371900776
P	1.22883738930808816825	2.07319372136836399534	−2.34353869188852970140
P	0.47204521430531781734	−0.82917191916877686086	−2.61689587721069694126
P	0.64780904782705761580	1.26903802540313148661	2.88013748792165369395
P	−1.22954227053974518569	−0.94242447234086446084	2.97026161935991650509
O	1.34851229974372177622	2.08913484921772507619	3.90771335164262945128
O	−0.27776019076645042105	1.99671621236778640274	1.87620623313214096584
O	−2.66551284810294841776	−0.67104242349633258513	3.29946797886895115681
O	−0.67894383468811758675	−2.26648144101521120675	3.43401412248715587339
O	1.12695456838615370998	−1.90022028030851664049	−3.42884936782813554146
O	−1.02305620014885345626	−0.63592341173745170479	−2.92950984156028670924
O	0.10604196741618626687	2.87427630505652276227	−2.92688931176331923467
O	2.61248974878097817154	2.62190513045346085264	−2.51998935504320176193
O	−1.85442796463616144109	0.56432600639872965154	−0.92045490208574398672
O	2.06646234576081688772	−2.17753271067404696737	3.35314962129983351602
H	−2.27505237383193792411	−0.15538893401467079869	−0.43560920338926018003
H	2.10835246802383302622	−1.35279847417725496150	2.84285863256981441438
H	1.08940360427951388189	−2.28612104477474042952	3.40959118444693043415
H	−1.50070642900420381238	0.10696899287082527186	−1.84119157930241961196

25

converged

Re	0.06185792196279001065	0.47828934310787546424	0.62341599071704456581
O	1.38906390448014893657	0.34895415004370711198	2.28438289864954668928
O	−1.23763208478742159357	−0.44761585544675785986	1.71847573241813234013
O	1.12264478243683152314	1.48872492247128729304	−0.57237215152062970169
O	0.55477467746238295909	−1.25521071925504923428	−0.56479133690649607757
O	−0.52631663790197835695	0.56202485442409133576	3.95537220817840351472
O	1.39872085368345944190	−0.01265421845294745218	−2.60910111859942306367
P	1.52509082872995449520	1.55946666735059169540	−2.13921893380368510762
P	0.42816423717657969839	−1.24588084231377260735	−2.13324243471548724571
P	0.56893118943457676728	1.41177290837918878452	3.10456380501019779672
P	−1.74928090160641036555	−0.32248745518440979918	3.22707389087230600566
O	1.33705832749234754253	2.21179495321829966414	4.10111667188963124886
O	−0.19508735312852418242	2.16415880130464133302	1.99080864089829590036
O	−3.01456920891501667370	0.48139200724137898302	3.28116847860571159146
O	−1.76860472303334304023	−1.65342780190870186630	3.91472128248656492389
O	0.99279751404433180806	−2.48217669694731268848	−2.75588086530103293370
O	−1.01042899755408144280	−0.92258220245304467522	−2.55850789028737679587
O	0.54402937618335955872	2.40379026183009392170	−2.89182800563028230556
O	2.97368447999203144860	1.91600870825987534829	−2.28248835369490388203
O	−1.78631622786902233280	0.67538121159952924089	−0.80605161864703278596
O	2.55363357292671722121	−2.09321726466130320077	1.27906327589085755392
H	−2.35246626449099860423	0.11596258008921762894	−0.26099715575193999095
H	2.24980433327472084670	−1.32582555612547281498	1.78922973537689888879
H	2.00182371109797330178	−2.00250407483579717649	0.48113551366272483412
H	−1.45517730477730289351	0.04696132356611967124	−1.60924827668887782117

25

converged

Re	0.23834908288737791104	0.81832636514359435864	0.99179680954944449134
O	−0.23198779615888412420	2.93831057821532493080	1.35232966419836619920
O	−0.82495555340898707453	0.48934420186617272330	2.55343114263616399739
O	0.97519583420342137448	1.34977658268228473126	−0.72127232585617684357
O	1.03023671584337117579	−1.09937993654604770910	0.58585893507056030494
O	−0.09449451201573597081	2.55518928541802736731	3.87270685229991551424
O	1.47341199325314931023	−0.81992182322380480208	−1.91457377745823920101
P	0.88574497250232364642	0.65918595307990845988	−2.16610831531979597386
P	1.07653612977308199561	−1.94743502562952319757	−0.73775069030090900668
P	0.75774065233817977649	2.96073927401600567322	2.54357193742347220677
P	−1.23912730142810034728	1.33283747636473437481	3.85192341044367791980
O	1.68640424090942886437	1.73179311509780453626	2.25031022302636962706

O	1.44257686123610073814	4.24603830260957959553	2.86616639619211532874
O	−2.58684645246785915873	1.95929562758633801778	3.65756535826306405923
O	−1.02709255064278481839	0.54579834824297579488	5.10852272477360180858
O	2.27078406398623577900	−2.87566627705762600797	−0.71445426220063490419
O	−0.21956391615264150841	−2.60622929663974334247	−1.12693463907877000629
O	−0.59420603121045278971	0.58963652797224441748	−2.54051878184339141598
O	1.76869432936293735636	1.32529897929904949549	−3.17059480381375768943
O	−1.69049051203345346295	0.32116750598284976581	−0.27014189460819743038
O	0.94841850341715960848	−5.06148743797130507005	−1.55153534462103004365
H	−1.25520523662093430595	0.31041627911591779254	−1.21414110770406180606
H	1.64747477191313840272	−4.43317085146099731929	−1.24964035916651172720
H	0.23530837754929273542	−4.39190158141320008411	−1.51347612128996389380
H	−2.06670667130479612794	1.20313780609190912330	−0.17624103692261788856
25			
converged			
Re	0.49500778298254266563	−0.37393902253698663163	−0.23075193574263014318
O	0.27075622866780812981	1.66265839302175777448	0.23050846647686229485
O	−0.95581729137750959246	−0.70097589978548524581	1.01786239735645334115
O	2.09497393677141774404	−0.34915583220289381217	−1.28472430663061887302
O	−0.61993035639827120953	−0.60868937929313371438	−2.01810966663993651693
O	−1.03127370998884715370	1.40575199650802939999	2.41054678322023407944
O	1.25978753760856321264	−1.92295523827831260633	−3.12023769000555262210
P	2.64865075878268507736	−1.21268116843823969830	−2.51126853349341327970
P	−0.09282428338731912865	−2.06949517809018157521	−2.22173293712149799717
P	0.13819868880483790474	2.35058494629375358542	1.64207839694907398176
P	−1.14145086023833197686	−0.19787671192055333869	2.52455009038859978787
O	1.37827172391230012849	2.36920280273804895188	2.47442686162185010446
O	−0.56532819769519448982	3.68483246160508937095	1.48847817496218381983
O	−2.50083571768869639129	−0.50237709520332329838	3.06745644704725650698
O	0.02710675813433835457	−0.75350486422677287468	3.33874019673033251010
O	0.36175160767431652209	−2.47535077611275866616	−0.79719708145820278311
O	−0.98714190662383316521	−3.00181219881641325742	−2.96783936490963107246
O	3.17924550027846564149	−0.33842906369814446110	−3.60659828070894494800
O	3.54952040391360634786	−2.30332418474135103281	−2.01372574749149979567
O	−2.92194199588857639327	3.78771748986304856999	2.68078539663929005243
O	1.86957566118764106911	−0.71998985650201485775	1.60273411964352630044
H	−2.94369224513480354233	2.83645289690612800726	2.83374783275349617639
H	1.96736561733071857461	−1.66747330646455371728	1.45477382640424313109
H	1.16261706758346461399	−0.63176434053282082282	2.36229942342880949724
H	−2.03639270289721485696	3.83369313520941279450	2.20999713485595172813
25			
converged			
Re	0.21775522922091711253	0.71705235858025495599	0.94971132528668023021
O	0.89775184749193148726	2.70084831918396828243	1.64040213944481783059
O	0.25413478809587075613	0.08729023209787215021	2.80238404327381296355
O	1.70287806958513621147	0.36745784846876250462	−0.19365910835738220364
O	−0.59782178613806213896	−1.11811203548136850472	0.23437398832153280570
O	−0.32985828365028951881	2.37089295599997917918	3.86027492213576461921
O	0.51141754499226721453	−0.90073425163667353299	−2.06333324396525519262
P	1.99961191200430832282	−0.60734381235357248752	−1.45969927404148558381
P	−0.88081603282088183349	−1.33759774073290804708	−1.28418442652527797776
P	−0.45451856147168839817	2.93321953051716066696	2.35054046016754858428
P	0.30915842036486462785	0.85082368898606497432	4.20792650794293976446
O	−0.95146894096134060348	4.33916207288840816148	2.44586387156200935067
O	−1.42479095695224766693	1.93652552609588934907	1.60671724939801663368
O	−0.66209880799605791424	0.22734517865872638787	5.16370847491642948057
O	1.71040140334038004433	1.04509836251508780869	4.69441719625588493869
O	−1.17038058335280692468	−2.77641028725080474260	−1.60374421202385319951
O	−1.91551637630705928750	−0.35121264198569479564	−1.82908328848703916059
O	2.74285904601491115073	0.14651911274391909989	−2.51649858551911798088
O	2.62764933570167924515	−1.88004573103637873110	−0.96887806006980392581

O -0.7449904868862176066 1 1.74943177781569758622 -1.00341015011523193579
O 1.23104812839292487858 -4.21955389424893922978 -1.64406577867538827853
H -1.39617680490449846964 2.14784161169423404303 -0.40638652957467535920
H 0.35391597892158815908 -3.78149990410926983841 -1.62704888772448685863
H 1.79015939307010341075 -3.45436209116846937306 -1.39504300212331511233
H -1.21410346944162972349 0.90846381376761098370 -1.39448562722689128890
25
converged
Re 0.13103753292041930845 0.47716867821933151017 0.10213947361461223062
O 0.94608074405096431736 1.83855080104389356777 1.65742956025127385900
O 0.36512959140350009779 -1.02421803380446241327 1.39145366615492216411
O 1.52405167678300967893 0.72087903587633639368 -1.17295312526721406954
O -0.90054813729264848732 -0.77781279997542041027 -1.28639510827455616493
O -0.12215119868520582513 0.40243657120936288107 3.48702669505623674695
O 0.50512136468833412906 -0.23783218608041450937 -3.32310889556655109089
P 1.76150892132254477396 0.66872267859306444571 -2.78504702752252208953
P -1.04583372185380785169 -0.40385406406950058456 -2.79950324512706316682
P -0.34289180451413991957 1.65573110119388777761 2.48325197812924658436
P 0.52753903687069514383 -1.04663022996502741435 2.96301144011807382483
O -0.80430930699895453184 2.80312111551734766124 3.31986250748479916339
O -1.38924870889370333238 1.15085339219625604201 1.41321144572749712864
O -0.36274500324607994450 -2.10812468194799151888 3.56233633542725280918
O 1.96179021348514992518 -1.09762433840049733469 3.41257538613499367131
O -1.64299302220664533714 -1.50736041594787484321 -3.61265879129060962782
O -1.75446408332905567917 0.94966321831844857648 -2.96637827345358306985
O 1.69133369919777476476 2.04780149160481750314 -3.35901840704567122842
O 3.00149188061913463343 -0.12172583667319873679 -3.07112304985856443551
O -0.84283652362629557508 2.27048457565835448690 -1.05633298933868946534
O 1.51177272941215812274 -3.16568248147861197594 5.17668029346307623229
H -1.59725915856796008008 2.31080793646999271829 -0.45514959338632343666
H 0.66435702551238384750 -2.97696193546153908827 4.70928960053880363290
H 2.01537473631460928303 -2.47558498609037869187 4.70183516865380379812
H -1.19510849292738940974 1.74829138871160605362 -1.92563504563878007403
25
converged
Re -0.32248480315765348792 0.53491643840253544351 0.99848965654233001743
O 0.83571323097633620769 1.73142169791195232875 2.44282607509289739767
O -1.48189717943009990897 -0.05209114192353107853 2.45732697538041211516
O 1.13034611472187851433 -0.63988815211547456219 0.46587993899607271775
O -1.57922274504447690546 -0.42727322205712636727 -0.44630015601605643738
O -1.28070816994893532659 2.13040802164477494074 3.82234273727314377567
O 0.21353898681802152559 -1.74715066499237359210 -1.66760676910841332266
P 1.57411361272167438230 -1.53316609537323755852 -0.79245506961199518869
P -1.09536365527865742209 -0.77095602331786095007 -1.89082898661086917791
P -0.36025312569766798632 2.69450168093307462769 2.61906131602798009084
P -1.79324216543727343876 0.52952241039838376935 3.91451297064331615871
O -0.05981999336247459387 4.12279659708025825182 2.93677052217817902147
O -1.19437635782372808535 2.46415389336790457975 1.29988342500696729687
O -3.27354529013162443007 0.59786412150209378158 4.13698718739492043994
O -0.98057657900546735252 -0.14832014515570018931 4.97279666941508491362
O -2.08747704908674691637 -1.57990223555010866896 -2.66191966590988915442
O -0.61780814041270915027 0.49592465841234062429 -2.62110672107133080289
O 2.61009549677202956630 -0.81746637443413450175 -1.61594161167476468854
O 1.97957391796589265986 -2.90565600261265766235 -0.31662553381314173517
O 0.47390566480091422807 1.79639661992497612353 -0.80368164105807959885
O 4.38262106867338463445 -2.90221674241750982048 -1.58241178148058314612
H -0.15389531701687622633 2.51443334671209983711 -0.65153963989775909305
H 4.00679730458069638388 -2.02071340208922434201 -1.78660563387838489113
H 3.61579423106137909372 -3.18859508066392605130 -1.03916127467379015847
H 0.06437096393160809948 1.22215581229734371327 -1.62389297957823930396
25

converged

Re	−0.04514629448019419344	0.07437859275274642801	0.27299310738578885793
O	0.95403772301945166667	0.94199656823982480525	2.08208491416872654156
O	−0.36008972621097473654	−1.57373386553661465115	1.34503998257196788479
O	1.46408460185515476581	0.03556877465211876616	−0.88279432892722042858
O	−1.27227297446129994363	−0.64320671294975595433	−1.31186686719776690602
O	−0.68038409590772830349	−0.33273141910390252596	3.58310122338891812177
O	0.39253664761846046583	−0.31355068767325167123	−3.18938906606651295661
P	1.81043927584601149761	0.09799005468812321817	−2.47468176807844875142
P	−1.17738829918245624384	−0.06816655434853244322	−2.76446425402498086044
P	−0.42754047663020161574	1.04095631647383068952	2.77197398562044305947
P	−0.34405266686237684359	−1.83928493168919970913	2.90882388245232492707
O	−0.64778835026504011374	2.16788377851267943797	3.72470148403966971884
O	−1.43516121460246504071	0.99960159166346551629	1.55760585396592454721
O	−1.49001987139020619644	−2.71690355291962148954	3.30521863937814019963
O	1.01119345249913283880	−2.25891698529266182760	3.41319347116009641496
O	−1.99944663757812524096	−0.84351250845560965530	−3.74424528645029885965
O	−1.44763410858996510733	1.44377042189825477436	−2.77991750161755213355
O	2.19984782233263764795	1.48921084709539952584	−2.86026776546195815598
O	2.77825425911356216346	−0.99551174565907507574	−2.80867355618420422303
O	−0.34820845460019461681	2.20812505901341715742	−0.67041328905208641409
O	2.87313297370470888126	−0.32265550403459275497	3.82601914376773688531
H	−1.10753125876229585600	2.39500043134575424375	−0.10409433532004580547
H	2.40149118458624100114	0.29182800224043281556	3.23825316906329385702
H	2.26493796462298613292	−1.09460973698862673587	3.74098834103430410991
H	−0.76109147994426051831	1.91757376079318131445	−1.61238917004824910961

25

converged

Re	0.36468420552294772419	0.36340156597350770085	0.06678673971752741023
O	0.93280538571317983543	1.75232621419803358265	1.70772930954032720052
O	1.15693209370887362120	−1.11991082460101587515	1.12563898272402096445
O	1.47333472489829486740	1.12697180722276080189	−1.28204508891004831383
O	−0.41852235873118526222	−1.00324239154617989023	−1.38178179606679485403
O	0.55111786327012024511	−0.11008865152387002639	3.42197519711007780430
O	0.54852805962849415788	0.11038642282160351926	−3.44481463370091445242
P	1.54042397329975422338	1.29858168315986799435	−2.89990075102353017655
P	−0.82525755680535728409	−0.54691505500273573936	−2.82240151266588545909
P	−0.13859604470301781221	1.11395947788048932381	2.61215477947662044755
P	1.56692318224977666929	−1.20993959895651648218	2.65206383042898696800
O	−0.80184097746358751202	1.97715929654327293719	3.63450388227112730632
O	−1.11994234519020596785	0.42697484897568616091	1.58385398088090489921
O	1.13836625880180020154	−2.54627051541035420712	3.21777851623982735774
O	2.98407134273157614857	−0.81854295135023946717	2.91298659540587800976
O	−1.17575459526294467949	−1.69438109699134753505	−3.71492554473191249897
O	−1.89853777696802672459	0.55244566880049140285	−2.76690552968881453566
O	1.01277851674396957016	2.64029458369812219942	−3.29742378847662243047
O	2.91679661631951248069	0.94470670997628014565	−3.37517083938994000292
O	−1.21258376288951907895	1.88219220055716784934	−0.77191186616237350560
O	−0.33723732241133064225	−2.24663407497370526400	5.39678647510621978256
H	−1.87676279305477167192	1.62935661452328406362	−0.11833687129367764790
H	0.23182657448993621019	−2.46433715149574927850	4.60157646121714947185
H	−0.52000361342432832590	−1.32490745549759769695	5.18438183004452124436
H	−1.48734966532694756758	1.36751267824007127771	−1.67579836965175843488

25

converged

Re	0.42292700604786015450	0.75855008452286676324	0.69985611124363922286
O	0.81298453040576035278	2.76271128452515446483	1.52210405243094015759
O	1.21762602539918329825	−0.02786466614662834462	2.31015587703108193907
O	1.51107339003611329531	0.94361761671552313313	−0.86544735687335316854
O	−0.20156287407097522868	−1.17857761869303234725	−0.00341914997929732012
O	0.46186644859331160706	1.86647403324826499649	3.89148840041133192713

O 0.80447250269534986700 -1.03905238472014471718 -2.32956585357329704067
P 1.65228566771504548072 0.37335175700284578548 -2.37843998098006581543
P -0.55431204979809800548 -1.42136582515452980502 -1.50593404577754697016
P -0.26047370149394855066 2.53237591126719374657 2.60846334153581826953
P 1.53745228615139550854 0.57533894503472193804 3.75664936359253243126
O -1.01617976091385875392 3.72247793192079212687 3.10272102486485357176
O -1.15312567830422141313 1.38720593842332196033 1.98666598468346111694
O 1.11652976273124027173 -0.39550705287637760721 4.81771112137427071076
O 2.92683823184388947070 1.12222778659552990810 3.85107978437981968156
O -0.79068792060756820650 -2.88457313747350951871 -1.78449522653954018026
O -1.69601666834358955072 -0.52059505544833062363 -1.97605857814341101530
O 1.02142034301505324478 1.31862111322207486985 -3.35017878715992800664
O 3.07679199574752759716 -0.01278598038741843301 -2.63470999662410720887
O -1.23380389970096526753 1.62856532377936691347 -0.75812161171611647248
O -0.66107139768405676072 -4.33409829584615202691 0.43799910860870799878
H -1.89484967752745570024 1.61206716084265799793 -0.05387421134261283218
H -0.36350213613688830971 -3.60431875079989483268 0.99187142898768998478
H -0.72005020838420785712 -3.85098557800552887542 -0.43247162389888754719
H -1.41043221639356719521 0.76723947962787619748 -1.33724919342683712387
25
converged
Re 0.11251646276981672190 0.49835867465584560154 0.65417813198446628231
O 0.95013808644318265006 2.13670679266591223566 1.87261371325839731661
O 0.04804737784046639315 -0.62540803175237180689 2.26504824239878610470
O 1.61289810622069595958 0.27933543564705992646 -0.51638558667813061476
O -0.90484818606589823009 -0.96204046844484403245 -0.53714652862568379454
O -0.42518233788395753958 1.34632924151027677517 3.87810653709729846739
O 0.44326616508646088377 -0.52850613976346072498 -2.65988221656685741578
P 1.89702918456275315684 -0.47879991995704679342 -1.92521617570027081534
P -1.08801953102829762621 -0.77644749052973405234 -2.07687226811583958153
P -0.41161829012735040578 2.30582001410426196131 2.58078210562635268488
P 0.07887345449945559750 -0.26119485483050919328 3.82643234571989987458
O -0.78455751025821407474 3.67807980635639930611 3.04121646944618717612
O -1.42608185712652435484 1.64922354370076429397 1.56654586392752870339
O -0.99128830935425671367 -1.02487585526086122378 4.54437139833251624310
O 1.46022352182576731217 -0.31936560948684794559 4.39432111396220737021
O -1.66601297547160887014 -1.97844715637704426925 -2.75029295292804931705
O -1.82126048281917740645 0.53295480824457874824 -2.41175639490430837597
O 2.77904206717677215721 0.35305069367880015374 -2.80158986233981055491
O 2.37858664463575930625 -1.86711293364689323582 -1.60940774944416875591
O -0.54499185598962318622 2.12643406192187267578 -0.96313574662258982784
O 1.87173481640852901187 -2.98293198424015049142 0.86352176357443644061
H -1.21136223620620664398 2.43668152259421999517 -0.33373492214559047353
H 2.00496143867015907958 -2.57859120787035145383 -0.02293893444682570948
H 1.31172330054353070139 -2.33415005199750158482 1.30864577583028451890
H -1.06761706920521848296 1.47599711437895253496 -1.62062413953108763742
25
converged
Re 0.56505616421199245103 0.43037074587479717813 0.60126240428232813340
O 0.82312644646352484123 2.17492190365140114494 1.91742456467489175154
O 1.81734479570264340076 -0.55245489854180995781 1.74779005952750532416
O 1.32193767018361674204 1.21055154278827692416 -0.98849838474315898651
O 0.02686373037300959776 -1.34653124648983557599 -0.51475632716822894430
O 1.14211103950213854752 0.63406649747687138241 3.93534401899215913190
O 0.74885921829160584373 -0.49998812579615403706 -2.80432707845571194127
P 1.32653854044900865183 1.02963157444246622418 -2.59757377525260224616
P -0.44888382007654187289 -1.27869775179267342047 -2.00999004851132800908
P 0.06774140234177401798 1.45145762878150219599 3.05154300407481837709
P 2.35707897058443816007 -0.30269087441848863129 3.22956910445640366092
O -0.73436148093377751067 2.28563139409287696679 3.99502096859477129343
O -0.75742214423542897350 0.34523361580697081719 2.27494264155668846072

O	2.35400093771869700632	-1.58692051551076729332	4.00194972430276418862
O	3.62231807751486334368	0.49587203323162271085	3.25055485440026892263
O	-0.56082787699130531855	-2.63176777104009618213	-2.63541858957244379269
O	-1.73075052639506021812	-0.43384628052467366643	-2.10393108782722393357
O	0.41090022080882582234	2.02656630519410230207	-3.23465787955062245018
O	2.74067883527190891968	0.99820160766611853997	-3.09371497792406779936
O	-1.44020284281749888500	1.23662764386147783213	-0.29257481458077866376
O	-2.16788223878857388982	-1.88511333687520332703	1.27893491716019092053
H	-2.01671588913537291887	0.86401816508338491474	0.38575298341591024442
H	-1.58554470431427718680	-1.91159839349821991661	0.49774490371847135428
H	-1.71696774437604826602	-1.21877467189848887053	1.82465473362156460624
H	-1.55879678033183144414	0.56633321373586908987	-1.15024591491632843798

25

Coordinates from ORCA-job Tc_Deprot_m E -81.217910508600

Tc	0.52687321502804	0.53573148988550	1.14066800013173
O	-0.52497067867940	1.99587182472422	2.33362731374364
O	0.66087130767450	-0.45901754554279	2.70426953567182
O	0.17081786827665	1.55205559976850	-0.40797608723507
O	1.76921749598359	-0.62717795831050	-0.07270805287071
O	0.96684540239711	1.48051347968263	4.33415905654507
O	0.79138163818630	0.02634740280953	-2.32545284098976
P	-0.26888146468940	1.18280936975697	-1.89251202472573
P	1.40701101041354	-1.25612247019194	-1.45429815969587
P	0.79735656410249	2.42596119123432	3.01750216859455
P	0.86875720246821	-0.18660174852809	4.26787420635945
O	1.86858501219623	1.93514072657381	1.99818950343385
O	0.91117774596991	3.83168416826338	3.50736107308451
O	-0.34731625535951	-0.58847058585168	5.04565936053381
O	2.18343666893246	-0.72495374924268	4.74313647523490
O	2.60654737822672	-1.71802099052971	-2.21714675927723
O	0.28139561766942	-2.28816332849705	-1.33319192742947
O	-1.66838375369240	0.57453436636373	-1.83639533060111
O	-0.09472374685855	2.31653266654905	-2.84859106374152
O	-1.11219487450462	-0.83921119654334	0.33978838309706
O	-1.98985164594627	-1.99954454256432	-2.85643698455249
H	-0.58416760611997	-1.50756058538543	-0.19506737534230
H	-1.99761823135016	-1.04807136757609	-2.62767435282217
H	-1.15398027546966	-2.25207861726807	-2.40506150355679
H	-1.46198558854114	-0.26108760486216	-0.39292261989747

25

Coordinates from ORCA-job Tc_Deprot_m_job10 E -81.201928093220

Tc	0.34307310022159	0.72506898297152	0.14098510007573
O	1.89684055438432	1.42650693431316	1.38133265214679
O	0.22735826090093	-0.78547309333786	1.28320994649646
O	0.20627096012012	2.17201198427746	-0.98759899405448
O	1.35133457775692	-0.28713362483695	-1.39566823975479
O	0.86027323852743	0.37456971535387	3.46075427441023
O	0.68767455025915	1.24610902994580	-3.29943905056467
P	0.03430825207726	2.55179608647652	-2.54948524146891
P	0.83326575575530	-0.33444113171741	-2.87020872521453
P	0.91764460392126	1.74006210357594	2.55649056395541
P	0.30489822443788	-1.05339960075815	2.83922578059683
O	1.31317537604167	2.82553862924609	3.49923976525764
O	-0.44173635459273	1.84332976932038	1.83244727926021
O	-1.06968227851353	-1.31554369022375	3.40268440007155
O	1.33550704705062	-2.10026674358894	3.17794440163773
O	1.82635078496451	-0.92709475784554	-3.81948294202024
O	-0.56307960240391	-0.97284250028212	-2.94378319292893
O	-1.41602020764148	2.67665107382107	-2.90027352479559
O	0.91471282121931	3.71437528625754	-2.89646343808750
O	-1.55451800409332	-0.23129497917275	-0.77836495093423

O	−0.32379130891436	−3.56017776122517	4.74424572125052
H	−1.49305590461054	−1.03433699290221	−0.24994991613433
H	−0.88345434738436	−2.82620974305223	4.41170442779338
H	0.48182098787564	−3.26125987912480	4.26772697086306
H	−1.18297107575381	−0.50544510277370	−1.74047306887287

25

Coordinates from ORCA–job Tc_Deprot_m_job11 E −81.199680531880

Tc	−0.19384511888292	−0.30415371468942	0.68759797076448
O	0.81290239912838	0.64415362067153	2.49854802694258
O	−0.37131626554731	−2.00151620101221	1.69651715826041
O	1.35935382259604	−0.34086594108609	−0.35392037669127
O	−1.16018538849752	−1.09881537536325	−1.01005289549567
O	−0.71885760770017	−0.81756822546539	3.94443159158357
O	0.49945639159001	−0.24754792240361	−2.74764411248841
P	1.76623498638621	0.15874556918416	−1.82549117153229
P	−1.14037001707206	−0.45408919233722	−2.43577528504599
P	−0.56949082950486	0.57930543145458	3.17013867530863
P	−0.32593167329989	−2.31655216185845	3.24895183834922
O	−0.93399391030629	1.65710031778078	4.13469947172708
O	−1.52766192432257	0.44866626413735	1.90301965436391
O	−1.46300948421108	−3.21974135352748	3.62882405180108
O	1.03003939131846	−2.70965959942255	3.74717104364343
O	−1.62748849357174	−1.39453238149437	−3.49242445758972
O	−1.76410941523772	0.93361579877131	−2.46093078061321
O	1.86998713347455	1.67393858147488	−1.73873703698360
O	2.96317513490092	−0.55418952103713	−2.36013660916145
O	−0.43891412557800	1.77523585599647	−0.37965765664244
O	2.64310238678959	2.39491850335497	1.02267565553256
H	−1.03867187720800	1.49790305408642	−1.14107978861851
H	2.03779941621811	1.84585026526490	1.55204643361854
H	2.46302851346506	2.09206002509152	0.11614472684728
H	0.43496655080337	1.85883829714610	−0.83811612360397

25

Coordinates from ORCA–job Tc_Deprot_m_job12 E −81.199855530420

Tc	−0.12575577568999	0.27804843313255	0.63685574476237
O	0.92094094821549	−0.35560482441249	2.35405458312814
O	−1.70904638957357	−0.34277086012921	1.42894444357105
O	1.31087166815780	1.04615702150817	−0.22958275302514
O	0.06094210440263	−1.32616054291296	−0.71500517470501
O	−1.08906886075698	0.13900767054272	3.84628829289843
O	1.14483312445146	0.04818913007160	−2.57122499437053
P	2.11866744646304	0.94538639585573	−1.61420036488616
P	0.03015594307114	−1.13042446046840	−2.26091257068907
P	0.28251163402287	0.77288773255617	3.23682340345961
P	−2.38147593449572	−0.27658966801127	2.87342041118228
O	1.07237231497472	1.24032161729214	4.41089092718039
O	−0.13741565382206	1.80549266727174	2.16703189890605
O	−3.39389703433219	0.83003408649097	2.91440749272862
O	−2.84221255765930	−1.62515880900883	3.33632390507355
O	0.41719653329600	−2.35370108394301	−3.02760259118201
O	−1.30588471027413	−0.51316368401186	−2.71854544669268
O	2.25088944646557	2.27992567275834	−2.27950002799931
O	3.39653043507905	0.19846645956843	−1.34387508468821
O	−1.64178143503627	1.15187438274665	−0.89960297067659
O	3.71017046466957	−0.82016334296141	1.22709881175022
H	−2.39805656046967	0.80511026451068	−0.41401264702478
H	2.83213643085587	−0.68305392758109	1.61435120549386
H	3.58070686089235	−0.48614836967448	0.31188732948367
H	−1.49813044188534	0.47313804940222	−1.71751382469430

25

Coordinates from ORCA–job Tc_Deprot_m_job13 E −81.199604401290

Tc	0.10824066171858	0.49439077664924	0.33684132860354
O	1.54039569530659	0.38386927434753	1.91839832479371
O	−0.93819444873366	−0.67612575774892	1.38858825379559
O	0.94971681054782	1.72605850771870	−0.74554685169709
O	0.68823038058381	−0.97910968403259	−1.03507991600037
O	−0.29928048719997	0.22420019528762	3.68794766712500
O	1.21856245313718	0.55957523329152	−2.97604886476818
P	1.21308434328790	2.05822327858746	−2.30353993376294
P	0.46577461396349	−0.84573701275545	−2.57753207639600
P	0.64868417954983	1.26174664388822	2.85786996653201
P	−1.22231574725358	−0.93519099380832	2.92986386708826
O	1.34666808796439	2.08745741859011	3.88341559145906
O	−0.28252475551595	1.97563491603047	1.85225490771150
O	−2.65982093076593	−0.66787159382217	3.25685715433413
O	−0.67692240158660	−2.26900202105026	3.36878366900561
O	1.15113416521292	−1.91660384896389	−3.36525175194652
O	−1.02836159627292	−0.68802228373399	−2.90784750092855
O	0.08807369044625	2.87257618575032	−2.86448912836953
O	2.59711164680533	2.60226545879941	−2.49275321289956
O	−1.82160902608806	0.59198418859483	−0.92568722668134
O	2.07359117561238	−2.15864201604094	3.33143129309488
H	−2.26982812428189	−0.09698490902185	−0.42363212985535
H	2.11290791488656	−1.33380313603339	2.81999419046741
H	1.09760222784434	−2.27700065295710	3.37348717968426
H	−1.49472052814649	0.10721183244300	−1.82152480140507

25

Coordinates from ORCA–job Tc_Deprot_m_job14 E −81.197236608120

Tc	0.05445283950985	0.47940936432440	0.61222409271976
O	1.38189738217794	0.34821440373263	2.24900638030632
O	−1.21293589777243	−0.40616958263416	1.68418201043499
O	1.10687555251165	1.45920177591639	−0.54133706727608
O	0.54223739151645	−1.22437946969674	−0.53712130848678
O	−0.51842230751011	0.56127178115460	3.93299865678724
O	1.36543351150253	−0.00548193066740	−2.59577282381526
P	1.50502089425036	1.55719403611875	−2.10304965326238
P	0.41749878172907	−1.24912155916242	−2.10128587606311
P	0.57221540575311	1.40950783859333	3.07491111095450
P	−1.72989361672008	−0.32226189466778	3.19009244313923
O	1.34320787192473	2.21281686009099	4.06711251470400
O	−0.20152641838781	2.15057003683932	1.96345164223293
O	−3.00364841147964	0.46736747251783	3.25467542129454
O	−1.73850365871686	−1.66635469253173	3.85122364669428
O	1.00635541233129	−2.48511257495083	−2.70315986051286
O	−1.02349255780923	−0.95706751092919	−2.53966089977211
O	0.52710218074703	2.41835827881345	−2.84090062063132
O	2.95515764805152	1.90717353033448	−2.24758701360811
O	−1.75643512622804	0.68967524616156	−0.80972922030748
O	2.55307249569341	−2.10371042956702	1.28476123510687
H	−2.34005921945880	0.15750257646722	−0.25812871928942
H	2.24981319744195	−1.33306442860636	1.79020411580431
H	2.00315915842139	−2.01162030828807	0.48637503232007
H	−1.45238250316516	0.04718118593808	−1.59668525636498

25

Coordinates from ORCA–job Tc_Deprot_m_job15 E −81.196949268000

Tc	0.23611108266997	0.80909189799267	0.97208743860069
O	−0.24847347109922	2.88955172641639	1.31564943576947
O	−0.79945029525386	0.49271390803784	2.49134255231138
O	0.98275951976346	1.34664568432474	−0.67890226810669
O	1.03601507757911	−1.04654947215693	0.59361659313575
O	−0.10114154685342	2.54089916405116	3.83392157582108
O	1.45911059088109	−0.80070086517079	−1.89948822728830

P 0.89999267370673 0.69419754136173 −2.13263597528533
P 1.06290928440634 −1.91390317363194 −0.71393109514616
P 0.74594848319866 2.94386739210100 2.50073734043370
P −1.21310043554841 1.29182348241705 3.81131366520606
O 1.67963023805364 1.72495842269409 2.21243192533767
O 1.41004998234426 4.24349412662264 2.81118997630251
O −2.57808331298110 1.88976136306014 3.64745327887297
O −0.96673844293503 0.48678409187526 5.05016319923389
O 2.24686527244460 −2.85567877992503 −0.69320815310893
O −0.24335494046559 −2.56365583666795 −1.08745079333272
O −0.57358894556942 0.64562054919314 −2.53499848912828
O 1.80726974187728 1.35966912156980 −3.11605615824600
O −1.63590031680895 0.27586464166390 −0.26568453494335
O 0.89409587550264 −5.03019006231321 −1.50595456579490
H −1.21878114886868 0.30811890032866 −1.21289982086321
H 1.60264251112451 −4.40786347798687 −1.21338099526798
H 0.18944010019816 −4.35144841908833 −1.46518603193993
H −2.06802758163620 1.12802805207332 −0.15332987888069
25
Coordinates from ORCA-job Tc_Deprot_m_job16 E −81.195080369600
Tc 0.49910632108045 −0.37148645432790 −0.21374364669955
O 0.29120563485478 1.62017635912498 0.23850328587094
O −0.92414513898367 −0.68189080834639 0.99336263010014
O 2.05405433769786 −0.37984733155781 −1.24673307434200
O −0.61707459584034 −0.59445067515188 −1.99310678237675
O −1.00041044537189 1.39288979983005 2.41526636393478
O 1.24572640750648 −1.92546234343233 −3.09984716735532
P 2.62289847580001 −1.19616890052040 −2.49556763102981
P −0.10776128098843 −2.05656239758621 −2.19979320388725
P 0.16086650832037 2.33162997679463 1.63507957561104
P −1.13796307246080 −0.21087496035927 2.50267479776953
O 1.40306224348921 2.36671024125719 2.46542850482389
O −0.54580755277192 3.66278708908608 1.46726007649802
O −2.51408737037411 −0.50085658215475 3.01034674763442
O 0.00297334813825 −0.78975043728053 3.33751048041495
O 0.34857467501239 −2.45588156130872 −0.77590768470074
O −1.00785160245048 −2.98607927681217 −2.94312661112003
O 3.12644105818262 −0.28614446616166 −3.57415930379859
O 3.55239519285571 −2.27619609711949 −2.02835390987383
O −2.91001383843362 3.76255528161046 2.64500483594552
O 1.86509401130944 −0.68715821955552 1.61653951601409
H −2.93231473014962 2.81108073902657 2.79682888425399
H 1.99620128425678 −1.62865865230189 1.46728594235303
H 1.15646436041546 −0.62845761156571 2.36593800239142
H −2.02143422478081 3.80919729411400 2.18010937584435
25
Coordinates from ORCA-job Tc_Deprot_m_job17 E −81.191039845100
Tc 0.22499458917855 0.72108844689486 0.94711479708854
O 0.91808664954474 2.70230414957306 1.66926786053415
O 0.24823392075237 0.10008207868430 2.75529902076764
O 1.64654874946841 0.35805081921257 −0.17167232224018
O −0.59838310371980 −1.09058451455090 0.24064800846874
O −0.36803192097077 2.33656076072170 3.84688872759485
O 0.50363294057750 −0.91138183165928 −2.05582538138517
P 1.97870540966227 −0.59561450975959 −1.44446517625006
P −0.89373299874538 −1.33135158985754 −1.26767217478016
P −0.44703822424002 2.91925119545416 2.34243252438627
P 0.31576638557856 0.83336140753081 4.17537172962428
O −0.96950898526865 4.31644547134052 2.44096003138887
O −1.37781662319469 1.91628395816759 1.55704024081901
O −0.63061921592523 0.17860748252177 5.13485456415721

O	1.71765465196423	1.05149473918702	4.64771490298875
O	−1.18006088488810	−2.77602246418548	−1.56811976604894
O	−1.92313113118683	−0.35157469432859	−1.82877537404664
O	2.71837206892278	0.18023367032273	−2.48748394668249
O	2.62738264982962	−1.85839344706162	−0.95503733818690
O	−0.71549470128008	1.75074768068325	−1.01627248835503
O	1.22542806542046	−4.20414945151286	−1.61613202582294
H	−1.36075754271442	2.14248132814690	−0.41094214422474
H	0.34677971257500	−3.76804465606995	−1.59586870298993
H	1.78374737610130	−3.43674511479157	−1.37300652584953
H	−1.18455783112772	0.91796908534569	−1.39951903667937

25

Coordinates from ORCA–job Tc_Deprot_m_job18 E −81.189614279520

Tc	0.13225758111336	0.47947150590947	0.10332876160510
O	0.94513947267339	1.83557386374253	1.69464166391446
O	0.34312279523432	−0.99686688731790	1.36409663850933
O	1.45973527188974	0.69360920880486	−1.14877098033697
O	−0.88434380089107	−0.76214286067789	−1.27298529816066
O	−0.19236424791089	0.37561010920582	3.45998731700469
O	0.48484579061832	−0.21531570911949	−3.32459236288340
P	1.76369676449856	0.62487728374699	−2.74860435530378
P	−1.06341050212908	−0.38755928468657	−2.77758570378361
P	−0.36591512857549	1.64362007771434	2.46696203848478
P	0.51752913656994	−1.04233358785221	2.93274481444458
O	−0.87640771987276	2.77603567856842	3.29659810618987
O	−1.35796644517864	1.14301624707821	1.34201419976050
O	−0.33758785991898	−2.13859264476961	3.52042947964299
O	1.95080450771873	−1.04873183704208	3.38650942487957
O	−1.66288095132995	−1.49491829043468	−3.58540601084972
O	−1.77132196749499	0.96363204221320	−2.94183927070109
O	1.77985606484450	2.00326420221781	−3.32824275440474
O	2.97645649655403	−0.22218173459380	−2.98232644678995
O	−0.82002452700847	2.29829097738855	−1.04523987263570
O	1.56755110869551	−3.13455820852473	5.14094415453538
H	−1.56377199504493	2.32928708910700	−0.43243591908756
H	0.71536365534145	−2.97697426760336	4.67141311371288
H	2.04590685763878	−2.42502260523187	4.66779434676688
H	−1.18007036759661	1.78000962687476	−1.89263508552935

25

Coordinates from ORCA–job Tc_Deprot_m_job19 E −81.189347077670

Tc	−0.30836886886371	0.51434663383160	0.99315413198823
O	0.84050253192109	1.72348574672252	2.45877470294540
O	−1.45423888908145	−0.03828110923464	2.41692685065606
O	1.07449013703082	−0.59315120859689	0.42551746843027
O	−1.55356326546838	−0.45477865023050	−0.42658146739034
O	−1.31257635583492	2.12686213998544	3.77597284839057
O	0.23043893749176	−1.69475435013369	−1.72407122024451
P	1.55673877013807	−1.50893879925639	−0.80718638375164
P	−1.11723656133569	−0.74239782559866	−1.89295383605832
P	−0.36109546547036	2.67405786471038	2.58920624696018
P	−1.74764175440762	0.50529036267758	3.89154332988287
O	−0.09560992717806	4.11601681546788	2.87792008782026
O	−1.15855485889960	2.39436429386391	1.25519163582738
O	−3.22264619097355	0.51315402497739	4.15507557113017
O	−0.87981624612604	−0.13948021150638	4.92518410587415
O	−2.10619027122709	−1.57227398989289	−2.64759109927189
O	−0.68361215595971	0.53801440347230	−2.61641190723373
O	2.63359002732642	−0.81298250555026	−1.59343590714731
O	1.92485938790176	−2.87980642619648	−0.29995146442080
O	0.48686519732306	1.81382952708913	−0.80139596207609
O	4.36299755675425	−2.92697172927170	−1.49959258193085

H	−0.14570048304854	2.51426173687241	−0.60074401944775
H	4.00691947953512	−2.03994181613423	−1.71636834475884
H	3.57818403939928	−3.19833370859042	−0.97529529371096
H	0.05746525124251	1.26950879640748	−1.59608748289447

25

Coordinates from ORCA–job Tc_Deprot_m_job20 E −81.189337153690

Tc	−0.03339817195010	0.07609373045581	0.26674393061657
O	0.96992346523007	0.94084013725699	2.09969055639790
O	−0.34710934731028	−1.54486308459387	1.30923709393969
O	1.39964311086425	0.03402926453035	−0.88056936639001
O	−1.24521854811287	−0.64164947154438	−1.29736793041956
O	−0.70128061217379	−0.34642417180141	3.54541840876087
O	0.37463369271771	−0.28261105099425	−3.19798285800377
P	1.79206639038703	0.07032646729170	−2.46176187928979
P	−1.19463243641996	−0.05586937750857	−2.74377044070673
P	−0.42370649779784	1.02934384479359	2.74881252536583
P	−0.32426376579052	−1.84043650299944	2.86632529992121
O	−0.69224689971977	2.15351324687954	3.69345509307158
O	−1.38416158037913	0.97753736634334	1.49594014127212
O	−1.45382419915577	−2.74450143007114	3.24898125191865
O	1.03590745301075	−2.23694254951623	3.37516018738043
O	−2.02147494905613	−0.83831380025923	−3.71507701984600
O	−1.47480608309858	1.45212022221421	−2.75358559479540
O	2.24430693211749	1.44259695223905	−2.84729487908724
O	2.72448345441753	−1.06173249651248	−2.76461903007788
O	−0.32963491287745	2.22202029875044	−0.65754950614777
O	2.86309240347277	−0.28636802568833	3.86394587878382
H	−1.08072281201574	2.39233302429104	−0.07765272236412
H	2.38948936374766	0.32363576879864	3.27191362289146
H	2.27211006602021	−1.06847264550839	3.75683303882414
H	−0.75297552039701	1.93489427787082	−1.57842579244798

25

Coordinates from ORCA–job Tc_Deprot_m_job21 E −81.187408774120

Tc	0.37299532195987	0.37886938189115	0.06836446003172
O	0.94009750289966	1.77516884584085	1.73254344300970
O	1.14718060600771	−1.07134178853801	1.11201761017044
O	1.41668917038473	1.09787598605427	−1.26326194736086
O	−0.39225019671229	−0.98629463087959	−1.35542122228072
O	0.51290553166941	−0.11550900906043	3.40229637406264
O	0.51007312147795	0.12404343137196	−3.43947671038195
P	1.54730523626440	1.25716247966565	−2.87873188645683
P	−0.84615674889578	−0.55168438935684	−2.78381779748711
P	−0.14821459193032	1.12371498334741	2.59303266525847
P	1.57503564230003	−1.16958526797413	2.63165764709392
O	−0.85926019450448	1.96074556518701	3.60630449164299
O	−1.08403930727931	0.44698318639051	1.51406173218643
O	1.19071662298214	−2.52461735244817	3.18425865121341
O	2.97653244056278	−0.73336510304022	2.90116271471992
O	−1.19038643679357	−1.71228796931049	−3.66312334580409
O	−1.92857881190734	0.53421773267419	−2.72450744717317
O	1.09899964726998	2.62058889970131	−3.29958872081913
O	2.91919992353846	0.83454185063716	−3.30650642453399
O	−1.20536030404046	1.91301669860712	−0.76050486991275
O	−0.33086154073766	−2.28983086499681	5.33860170558914
H	−1.84891491556565	1.65549557637337	−0.09033015528339
H	0.25926380460707	−2.48386222350494	4.55271325487010
H	−0.53668213862226	−1.37230680362775	5.12898422520101
H	−1.49008939978807	1.38936079029674	−1.63392845915499

25

Coordinates from ORCA–job Tc_Deprot_m_job22 E −81.187044749150

Tc	0.42563172721286	0.76168015836053	0.68945204549596

O	0.82213330240324	2.76721936198999	1.54089043736575
O	1.19909813292570	−0.01304743277482	2.26506027671279
O	1.45731899972734	0.91167577626857	−0.83598765180281
O	−0.18871072757340	−1.15874811636242	−0.00285128635258
O	0.42326705446981	1.81848250734044	3.87942426358593
O	0.77676410840594	−1.03170454740959	−2.33979835954659
P	1.65319648123109	0.35769668074254	−2.34914089656889
P	−0.57328442982493	−1.40931879049335	−1.49054446411761
P	−0.26760116887148	2.50979886594761	2.59456268311708
P	1.54127161157573	0.56781285768928	3.71377631225838
O	−1.05718812498215	3.67488602357317	3.09722854231142
O	−1.12219720846444	1.36432275306928	1.91900698701512
O	1.16378391574163	−0.42861856421401	4.76719359182877
O	2.91499788140608	1.15314048231494	3.79717949963472
O	−0.81060645716825	−2.87462021985014	−1.76248837884040
O	−1.71590766131102	−0.51041179083994	−1.95610571386772
O	1.07601871073375	1.32219381150142	−3.33486492956917
O	3.08023721965321	−0.04850531255267	−2.55227220336098
O	−1.22535294833789	1.65746341159587	−0.76159343263567
O	−0.63785676153534	−4.31514066805203	0.46059827140314
H	−1.87011275501377	1.64005963986775	−0.04426315603470
H	−0.33331565887697	−3.58188638218904	1.00617467725762
H	−0.71402967262313	−3.83409179690340	−0.41034278894275
H	−1.41135556988129	0.80076131255667	−1.32349434323767

25

Coordinates from ORCA–job Tc_Deprot_m_job23 E −81.185116882110

Tc	0.09794745022669	0.50720147763748	0.66448861484379
O	0.97286880823413	2.12537747234359	1.91459982467523
O	−0.02878529602657	−0.58178653473792	2.24385878438862
O	1.52501055512863	0.23981475158289	−0.47555631577751
O	−0.93335379702846	−0.92822290319522	−0.50355885266950
O	−0.49439839575167	1.35884474499836	3.86185299396863
O	0.41326953374619	−0.57787225577336	−2.63250273109086
P	1.85633211581857	−0.47483533478458	−1.89569314866423
P	−1.12960091188617	−0.76738521146490	−2.04062689351382
P	−0.40275491670503	2.32171035199623	2.57062230028252
P	0.02822233922038	−0.24276701305217	3.80755055899140
O	−0.78132441687953	3.69751011073960	3.01710021117399
O	−1.37936018768193	1.67594393052333	1.51007783773312
O	−1.03031484148712	−1.01442855130260	4.53417261729576
O	1.41466481376318	−0.29684392747908	4.36246367179707
O	−1.72879498514461	−1.97384662770617	−2.68890727996371
O	−1.82731658023100	0.54942695528002	−2.40507293899883
O	2.71260922318839	0.40541375726219	−2.74899982374311
O	2.39575787849059	−1.84769447185418	−1.60096233231685
O	−0.50034155478738	2.14231645493723	−0.97487223524974
O	2.02705916968608	−3.07831031384594	0.80657158739230
H	−1.15977316709474	2.45860310615943	−0.34367429504695
H	2.12735689823611	−2.64608088908937	−0.07432526614726
H	1.45797052150068	−2.45484739376873	1.27356829673867
H	−1.02675027138840	1.50385831989522	−1.61537520298961

25

Coordinates from ORCA–job Tc_Deprot_m_job24 E −81.184442257180

Tc	0.58326888068565	0.43774072186467	0.59199040123046
O	0.85427715161563	2.17972647031372	1.93240453949771
O	1.77748778928163	−0.53811519851785	1.73209971471189
O	1.27407982779662	1.15422854581382	−0.96487182433066
O	0.06003229596930	−1.32607997909363	−0.51028220206863
O	1.09733179878520	0.59387312937908	3.92121961279334
O	0.71682496375544	−0.49603047198001	−2.81628650617139
P	1.32181208233537	1.00929593328112	−2.58092191617558

P -0.46505412959556 -1.27832414047159 -1.98353475509107
P 0.06252070176466 1.44625071515044 3.02432172924868
P 2.34187590362703 -0.29287166096173 3.20490803446577
O -0.76984678916383 2.25671896435352 3.96299836823965
O -0.73681726657757 0.36276235014134 2.18831055454081
O 2.37943332565397 -1.58353834733501 3.96493925953859
O 3.58381986969962 0.54002760614597 3.22548165415182
O -0.57158375992278 -2.63773238453063 -2.59870832321552
O -1.74693784416840 -0.44117100595646 -2.06746123892142
O 0.44179741513246 2.03758560800030 -3.21687152063364
O 2.74777817619854 0.96216575579494 -3.03794864886273
O -1.41844528338499 1.27890620053782 -0.28345249848664
O -2.14031527536887 -1.91614469330027 1.27603994252614
H -1.97580608898694 0.91837324418646 0.41559681402032
H -1.56986623889417 -1.94176846613960 0.48693313369061
H -1.68999006951981 -1.23427422201689 1.80190330489162
H -1.55147743569586 0.60949533064177 -1.10200762531387
25
Coordinates from ORCA-job Tc_Deprot_m_job2 E -81.217119468270
Tc 0.57200257025813 0.25461204799501 0.33446161222698
O -0.35808933742024 0.85202162668650 2.22963156357614
O 0.84144037767612 -1.38492250762836 1.18673235182727
O 0.06640528390355 1.88160144907747 -0.44719316872961
O 1.71414413084967 -0.07203960604751 -1.36437329602111
O 1.37837492533040 -0.50019293520499 3.51903542141912
O 0.43219924612784 1.47152277651371 -2.91837210828040
P -0.54900788296075 2.25131608622850 -1.87789893734905
P 1.20053264075833 -0.01109745478885 -2.84458293803169
P 1.04346703673887 0.96890470883527 2.88535283594736
P 1.15073713002533 -1.90687194962964 2.65993557675318
O 1.98512631724314 1.11228878176022 1.65662384558872
O 1.22119908791433 1.94692081114941 3.99674823296343
O -0.06294589305058 -2.58463639878169 3.24232175128938
O 2.43040066805457 -2.67854042984162 2.72261256483197
O 2.31915077294062 0.07544484986877 -3.83573991139919
O 0.15467385797591 -1.08077420819100 -3.13178795119265
O -1.94399096655105 1.65394182905089 -1.95579905848209
O -0.43893013930984 3.70955570208051 -2.18971826410206
O -1.11491233883305 -0.62643953564243 -0.90567530263031
O -2.12257430014917 -0.85198566919727 3.73557095538223
H -0.63395907910535 -0.91975025367355 -1.74536932175855
H -1.43884061270605 -1.54816298300568 3.60035643428421
H -1.69631817080155 -0.12941576511486 3.24130930900602
H -1.54408532388685 0.21779903280249 -1.22738219284306
25
Coordinates from ORCA-job Tc_Deprot_m_job3 E -81.214409390140
Tc 0.19026192735369 0.11869039067964 0.05644758819512
O -0.31320508430802 1.09969595394758 1.93699613030781
O -0.61346719020779 -1.38930051809765 0.79976263148556
O 0.81535035661951 1.75263578994651 -0.62531397227942
O 1.00435759297220 -0.75179796293015 -1.64349497403625
O 0.12403109829984 -1.06201788403685 3.22308583022037
O 0.97467329283599 1.32017543648527 -3.11268734406114
P 0.61241009333529 2.48494564575532 -2.03295762458799
P 0.70551985264347 -0.32983646839991 -3.12397492295310
P 0.79613399786669 0.30646001532688 2.65465769510141
P -0.74951630997201 -2.09396994220953 2.22428175906813
O 1.74538320320711 -0.10827741790073 1.50181317267478
O 1.43722311496938 0.94584908823437 3.85709478329941
O -2.16559914308643 -2.06438835565325 2.71054951690692
O -0.04777628863462 -3.41602347311031 2.25566617497618

O	1.70318613832531	-0.88483297276456	-4.09170961710201
O	-0.75603406741354	-0.54678491751361	-3.49632376513212
O	-0.85686892579185	2.85309722638422	-2.16412014191260
O	1.58851849677213	3.59666192911830	-2.24895119593448
O	-1.61251842377495	0.48667113422305	-1.27187155694052
O	1.03001468923167	-0.54380951677754	6.02576650575703
H	-1.35905103032417	-0.00102680639424	-2.12205573280633
H	0.53677582757347	-1.19255273078004	5.51075852056788
H	1.24339825337882	0.08806738679377	5.28488516244787
H	-1.42700147614062	1.43276896439124	-1.54150462427807

25

Coordinates from ORCA-job Tc_Deprot_m_job4 E -81.214393091730

Tc	0.09915857248625	0.57587120003091	0.57038092436800
O	-0.46333402489293	2.25174552900759	1.80907966326320
O	-0.84717886353517	-0.47016035848252	1.78654710695533
O	0.89985118314150	1.73060253515564	-0.66926075734321
O	0.93722463151501	-0.99417200571074	-0.52121670008910
O	-0.25882551194946	0.82598904211713	3.90479312043722
O	1.15358035676044	0.27797850621792	-2.71662106850838
P	0.83235151650767	1.80784540053846	-2.26516999658687
P	0.75000808258245	-1.20790147704935	-2.06656072235737
P	0.55446056669714	1.82484727324503	2.89722242932069
P	-0.95627601100711	-0.58731380834232	3.37266603026275
O	1.53674519012432	0.91995983462622	2.10222027500501
O	1.13609692182771	2.89135117806816	3.76487390251920
O	-2.37896465651683	-0.56010050999181	3.83948906343750
O	-0.10807089744123	-1.73000492583550	3.85777157264801
O	1.74294689873734	-2.16640629126855	-2.64132124815752
O	-0.70596907043947	-1.47526992795341	-2.42751160888871
O	-0.59192107655514	2.16534285320170	-2.66074920032990
O	1.90629933370356	2.67129678991521	-2.84632998930536
O	-1.59955716253115	0.43881155723547	-0.93606041197467
O	1.40961728683850	-2.97995133467040	1.84796179066316
H	-1.33903490834498	-0.37659003298527	-1.48032044147399
H	0.88786397299390	-2.50212125906210	2.52626237033825
H	1.32685929628581	-2.40007601365345	1.07634391830945
H	-1.31773162596579	1.16952625094731	-1.55769001294469

25

Coordinates from ORCA-job Tc_Deprot_m_job5 E -81.215256862800

Tc	-0.27058557440408	0.48672688314729	1.11247817186590
O	0.04123129605561	2.42547250627964	2.00911858575376
O	-1.95928015794272	0.54204368700779	1.88541269588153
O	1.40092077779981	0.62679461838226	0.23730929286917
O	-0.28675920387395	-1.48324994123846	0.42930464259231
O	-1.32418082327861	1.73678599752570	4.04722503493326
O	1.26814490712910	-1.15877904889829	-1.55555860295646
P	1.84186002062225	0.33228777400012	-1.25896094617515
P	-0.12926930967422	-1.94154766475159	-1.05756120516446
P	0.17701693873251	1.78339373728018	3.41289292762671
P	-2.62776256129491	1.07577276366464	3.23875085215985
O	0.54016561036569	0.30874321767716	3.06600172985128
O	1.02566393384884	2.47894007092781	4.42529775211822
O	-3.58496899909019	2.19372682610206	2.95582118537293
O	-3.14037456215315	-0.05589183443429	4.07593558683220
O	0.19170516214923	-3.39763770290832	-1.18850884012979
O	-1.25957084997780	-1.43326649439460	-1.94196749104690
O	1.17128519467848	1.33407861817472	-2.17753988394468
O	3.33788195783128	0.22392958607388	-1.40291278307406
O	-1.15089466718221	0.96248248287394	-0.94081912179490
O	4.00196946461049	-2.26921377887769	-2.16848023879641
H	-1.30615789513624	0.03942176482453	-1.32541351430792

```
H     3.11717057489195   -2.61434119594758   -1.99765669397897
H     3.84911819812604   -1.32642992575232   -1.89529919036658
H    -0.31812943181086    1.23085705327097   -1.41806995242813
25
Coordinates from ORCA-job Tc_Deprot_m_job6 E -81.214165131870
Tc    0.19279761471314    0.12260626012984    0.06068597067429
O    -0.39390711527987    1.07319561510800    1.97874214058977
O    -0.52841476041008   -1.42981467509122    0.80470285633088
O     0.70997667178090    1.79286613762854   -0.62748154290970
O     1.07789584736426   -0.69031684231376   -1.65382750491803
O     0.14362961524478   -1.08562082599947    3.24133217192754
O     0.98032291604479    1.39138520752422   -3.11166712178116
P     0.51489715934398    2.52774397950677   -2.04243129912638
P     0.79925509097003   -0.27244369712909   -3.14176561916115
P     0.74622410348611    0.32681764848817    2.69643007185475
P    -0.65706226061265   -2.15601794297108    2.22462210866958
O     1.73540899162153   -0.02185492359916    1.55217190583224
O     1.34046090426459    0.98102716477797    3.91475861694189
O    -2.08182926311595   -2.20488748062292    2.68161947489882
O     0.11274249163882   -3.43914940151738    2.25757265211177
O     1.85481692352443   -0.76552790662441   -4.08076704574213
O    -0.63609996515228   -0.56505382289352   -3.55697106022910
O    -0.96681570445888    2.81309635017536   -2.21685491746552
O     1.43573878082323    3.69235152930933   -2.21495105403828
O    -1.63320587397554    0.39450109115917   -1.34742001359119
O     0.97079913479141   -0.54609228472931    6.06540150250753
H    -1.31333904107792   -0.07231826132669   -2.18403051689369
H     1.16316646309396    0.10252498901083    5.33348719148138
H     0.52124771667537   -1.21544405808976    5.53697059526994
H    -1.48250644556760    1.34752615009913   -1.60352955895779
25
Coordinates from ORCA-job Tc_Deprot_m_job7 E -81.212676589380
Tc   -0.13663942783907    0.13244208988880    0.33315063148395
O    -0.19205673428379    1.46399992994696    2.05861750192573
O    -1.59650164828318   -0.70977634934877    1.12481592529470
O     1.22562813723295    1.17479785441308   -0.43316022708114
O     0.22746861572117   -1.22527579629264   -1.19335750711767
O    -0.89741282973666   -0.47205909986131    3.56425935368479
O     1.24319286096809    0.41339288843243   -2.84633305940041
P     1.44526718328350    1.73556292553783   -1.91542641585987
P     0.21945199163749   -0.90278745527829   -2.72857417639178
P     0.36551999387056    0.34156248152077    2.95604078382722
P    -2.12871595788980   -1.06037412085770    2.58905524989506
O     1.06695172737572   -0.61037109492715    1.94448974633946
O     1.18677733241496    0.74606412287035    4.14938654353741
O    -3.36133630969696   -0.27443628265560    2.91618289432658
O    -2.18866870562456   -2.53990153045595    2.81147548163867
O     0.86395863027465   -1.97909662464899   -3.54433563578440
O    -1.15379543454877   -0.45179457632260   -3.21139516671787
O     0.33512197524403    2.73203537954724   -2.21134355771650
O     2.83959167545192    2.22139889793048   -2.14756544015256
O    -1.48689671946827    1.13278064854022   -1.18055850883794
O     3.37507919979765   -0.80264637890161    3.88334933901233
H    -1.46827276442844    0.48155676989713   -1.95577039594921
H     2.62147719318727   -0.20433186587269    4.11114652449393
H     3.06405486186248   -1.08092059065119    3.01273259516319
H    -0.86304483491706    1.83927777755874   -1.52008249121274
25
Coordinates from ORCA-job Tc_Deprot_m_job8 E -81.213063633200
Tc   -0.04482849695242    0.38191927892921    0.66717866580083
O    -0.31621995344187    2.08920866598304    1.96266738816525
```

O	−1.33039835361656	−0.45391496927709	1.71953589666081
O	1.11692635190814	1.38318537987346	−0.41699604787393
O	0.57728498175194	−1.26847546777763	−0.46385839789391
O	−0.67231917183059	0.55642744077240	3.96706732468835
O	1.27459054533150	0.01782348034819	−2.53870175208461
P	1.24148360921828	1.55648494159222	−2.00118518783069
P	0.49959310707924	−1.36867996918841	−2.03073628159803
P	0.43839512336138	1.37840376981418	3.11002318139850
P	−1.79695212591259	−0.45302147215135	3.24863360202236
O	1.26184663374612	0.29504482408453	2.34164356222327
O	1.18034979964218	2.22121154743737	4.09292358020898
O	−3.13770610848368	0.19957572190060	3.39687381191136
O	−1.62925338432853	−1.80418434405940	3.87212642324822
O	1.31748652730257	−2.50130356475728	−2.56495243549954
O	−0.93547191654182	−1.29911446853459	−2.53374563756811
O	−0.02269169141198	2.23564774702362	−2.50343533026650
O	2.53152784639399	2.18870683190384	−2.41355753207999
O	−1.55030060510425	0.70113259935214	−0.99984822148577
O	2.67152991823968	−1.99363946445947	1.35495872926304
H	−1.41655188443024	−0.12350405156486	−1.57135532666617
H	2.28804304302308	−1.25610492425695	1.85669198137478
H	2.11529485829794	−1.96339266505764	0.55696336055253
H	−1.05545865221915	1.38166312678767	−1.54211536297833

25

Coordinates from ORCA-job Tc_Deprot_m_job9 E −81.211918555290

Tc	−0.18592461582315	0.09876896946830	0.67169429470324
O	−0.14253388690680	1.60110483638319	2.21325398523853
O	−1.71529529430473	−0.53894559573583	1.51603224687428
O	1.29661814452525	0.89983783852410	−0.17292224854407
O	0.06002945607114	−1.44450120624360	−0.69945725845795
O	−1.00501128482016	−0.08858026985119	3.91907219627720
O	1.25534396108622	−0.10910639010156	−2.49342677170199
P	1.58696434736606	1.27358854765095	−1.70212034790658
P	0.09400981769827	−1.29014484620446	−2.25872348552004
P	0.33008177358294	0.56778540848284	3.26600439057074
P	−2.26508773574942	−0.70277896213472	3.00692091765574
O	0.95859439527316	−0.54646920951061	2.36687965206484
O	1.15041183119926	1.05628557827279	4.41132805136654
O	−3.45042039885136	0.18479482046293	3.23904522811582
O	−2.41756439290600	−2.14473439787657	3.38314154869631
O	0.62254405527116	−2.50663890014563	−2.95065548443607
O	−1.21929697872747	−0.74698391213212	−2.81054096990277
O	0.59855354574361	2.34650605212145	−2.12665346626051
O	3.03265849673365	1.57465055840635	−1.93568573098400
O	−1.40581526014619	1.06589372540296	−0.97205552800538
O	3.58125128939135	−0.23461984933298	1.36045068807480
H	−1.44571500655599	0.33631709713497	−1.67555332223195
H	3.25771494134071	0.15622315915228	0.53660767466012
H	2.74133692889292	−0.36953276925886	1.84144288091542
H	−0.70724811777853	1.66237972236634	−1.36727914227779

10.2.7 Table 5, SI 2

25

converged

Re	0.18423552663128409113	0.08404491422456233929	0.42267562326777813331
O	1.51694021594744832271	1.24301212367408520088	1.70924524864886717701
O	0.37680398227632416752	−1.33023438461940757271	1.70802983884451564656
O	−0.00833369927248898620	1.49832412184792707066	−0.86267900600802471800
O	−1.14847127793465864087	−1.07492387440619041605	−0.86389112236597076500

O	0.57079285360781828462	0.13311332171558551418	3.80280346998828866489
O	−0.20233662147806422138	0.03497654787185089376	−2.95745150125061462276
P	−0.19251560959754060454	1.60976262677756087882	−2.43592517997183444223
P	−0.04589882479364469636	−1.24445933339525671713	−1.94948817434457755127
P	0.41436494161718917528	1.41255050522225356069	2.79483972415972869996
P	0.56100176143081614821	−1.44167089904847656179	3.28127513243107005181
O	0.47571964486124845850	2.62192582666024165405	3.66414151703092860757
O	−0.89252612850910795217	1.16638295416889725686	1.98591067975974944204
O	−0.62238307144126892023	−2.11264138821589497752	3.92902933908339058178
O	1.90002392153793109308	−2.03240239124258570413	3.63938484637845727221
O	1.26099398599624334949	−0.99828946678443442053	−1.14056183164788893514
O	−0.10725315280781286265	−2.45383540831056690124	−2.81878931565262824677
O	−1.53152155846434068209	2.20052035254054123570	−2.79404956167500140296
O	0.99088830086532730945	2.28070887687886658668	−3.08366891507740614742
O	−0.51298838549650738194	4.00000778210668794799	−4.56538697929310544055
O	0.88156234044434411690	−3.83193300322722807394	5.41070365055787672048
H	0.26961231847835304132	3.53896640092658598675	−4.19456410083026209890
H	0.09894053320520727890	−3.37090933782235069316	5.03990707496547152289
H	1.52938329864565436544	−3.32540301895533696452	4.87564598027148043968
H	−1.16083531060273714708	3.49350616729695451212	−4.03033643828582288648
25			
converged			
Re	−0.20246379455959684357	−0.30632104732964871019	0.58872981187157180649
O	−1.44125849173363018707	0.81125589369205142187	1.98589433416385974240
O	−0.42780112196860564655	−1.81169800782836132313	1.74839116789905357763
O	0.04085935324873099517	1.20485261537864962733	−0.57337893739569156804
O	1.01794775763153699799	−1.42072656110858153156	−0.83892730599272369307
O	−0.58335457895352194058	−0.53510946436393513892	3.96545561790054046369
O	0.07741417185123711242	−0.07711751220783699678	−2.79264862301832250679
P	0.13453245798590135474	1.44768679992990834116	−2.13841852823839051467
P	−0.12652577825961891511	−1.42730506019342340274	−1.89328136109773303986
P	−0.33777217812237259054	0.81519724983358476944	3.08292467084312082903
P	−0.52216794893642803910	−2.07330667172164861967	3.31975684044190444411
O	0.96118055589079942624	0.56512871178684231932	2.25291576123075643068
O	−0.33391392400427710729	1.94844375733081998092	4.04988580815404208835
O	−1.79938750144963233168	−2.75642629951977369274	3.69276756824381546807
O	0.74544297039511253633	−2.70828850479716187749	3.82890165769399803963
O	−0.17187933157885265656	−2.55836900647406206843	−2.86366990746930571987
O	−1.38748800294380103537	−1.17600482696557695483	−1.01571222469495836549
O	−1.06679741516429471559	2.19681416588321054562	−2.65421130096166102419
O	1.46520686668313482315	2.03740274984100500077	−2.52886808875769020233
O	0.39756450996518205576	3.99469939396510786978	−4.08325696714033448131
O	2.88974215507118881163	−1.00159072101124313470	3.74476608089926488177
H	−0.37689914803982005331	3.52629172248385147626	−3.70515147527507116010
H	2.17870560638264398889	−1.67876785853080812139	3.81669396425648344007
H	2.41737908825974212590	−0.34378198652812386182	3.20587967962167930125
H	1.05793370749625670335	3.42814048963179640594	−3.62863822831842997019
25			
converged			
Re	0.18422054536575185879	0.08402514230730373512	0.42267269583619998530
O	1.09169261139864359755	1.64624608158906382549	1.63465697497617945544
O	1.41452945739532665037	−1.12854262488470324932	1.24858178757025495287
O	−1.04605491829293306694	1.29664367577283412558	−0.40326104122265926666
O	−0.72334082654277453983	−1.47811059292064839177	−0.78932586166602403122
O	1.56096206775296897362	0.02123297833761772502	3.53461275596197754822
O	−1.19252989983210122560	0.14689742543457359747	−2.68930049171016483101
P	−1.66664476094541891271	1.51480814953850018156	−1.85558599730040318931
P	−0.10683022867977956527	−0.91943243507124683411	−2.10344801678679749202
P	0.47519937455824479144	1.08754596211402820849	2.94876435962530347723
P	2.03518158292096451589	−1.34660941644767140346	2.70091698301855887010
O	0.20535993341261588041	2.05524761646543741023	4.04917003464887592656

O	−0.72615879604193023233	0.24183207731571984556	2.41838635068188345656
O	1.38542505235108048822	−2.52107580546320875214	3.38574120461753969025
O	3.53057222056111186248	−1.34368169929081493486	2.67450917907155982789
O	1.09459152381958002564	−0.07381467374415906502	−1.57305869157772759692
O	0.16294928264635233361	−1.88719058673788708802	−3.20382334998502660284
O	−3.16204039730758701410	1.51200060832278349032	−1.82923356056318331397
O	−1.01676778978013770960	2.68921750858631680714	−2.54038904977323554135
O	1.59406513439092001327	2.24633223586570096586	−3.22701372534067365550
O	−1.22538062555568583178	−2.07839398118932550119	4.07247857249347067210
H	0.66195069903691994284	2.49359152454929899889	−3.02568381770166849876
H	−0.29325615198636267866	−2.32554396455830891810	3.87103399897165845189
H	−1.28079705238433638748	−1.26524854811466758164	3.54136995462100978571
H	1.64930195746912833243	1.43312336869187939570	−2.69597126535775943879
25			
converged			
Re	0.71164579649105341730	0.40400074179268496977	−0.48153104965970044971
O	−0.58140612587171913717	1.74441840320881702553	0.71738700924225806688
O	0.43689391386145226548	−0.85635337291618429667	0.96303733689439741106
O	0.96332879424652517741	1.65664195775060663074	−1.88961326677151308928
O	2.00860148452461917401	−0.93197416153106515946	−1.59873144907020758865
O	0.38694900467433268609	0.82787522803343382982	2.89578194111874287486
O	1.13213254075455060921	−0.02369566038957147958	−3.81699753103331262949
P	1.21833489829306662600	1.61383220310205244274	−3.47188348280668446222
P	0.91928170349374127568	−1.17642662413160969948	−2.68764220060332759132
P	0.56015005138124440709	1.98776500736649075485	1.75161496416179796043
P	0.18491001162142503556	−0.77118270647131093032	2.51992677670390019529
O	1.83531205611283265711	1.60102949691617912897	0.95377948830136971559
O	0.56989228886488130232	3.28573030074209615492	2.48147037209100496113
O	−1.24342150271036144815	−1.12650383818555521742	2.86836393315412729876
O	1.22376999752103166053	−1.53407831158518948023	3.29973161544802717771
O	0.95141424430124721390	−2.47750009370250090512	−3.41632037876876637483
O	−0.38768977624861600262	−0.80646702194503394612	−1.92293700400461764488
O	0.09918973024892641099	2.27342204857223340042	−4.21770433751569395753
O	2.60849551710779703484	2.06298701227824743398	−3.80367266942558712728
O	−2.99272335169076342254	0.80972655952052285944	1.98366005689592506123
O	−0.46897325199255357475	−2.88546794845530030216	4.87971954753996506327
H	−2.44989184867607256990	0.07214152871495582708	2.33811986987675091143
H	0.33374553019277336530	−2.51445806266862792455	4.44012007363180316588
H	−1.10263955456198403660	−2.39833086229312586468	4.32337333181155969442
H	−2.31110216150063640939	1.26396818157808366045	1.45774706235579087910
25			
converged			
Re	0.64642060965098557812	0.03017706205490768012	0.78861613689481724343
O	1.32598128774459866897	2.79212989905814579217	3.78113630191225080779
O	1.24959883494675971782	−1.26423408756051158797	2.05114391198206913458
O	0.04129485689920434077	1.31286381128594054069	−0.50894820882631774950
O	−0.03911055765416271424	−2.79376887920791627806	−2.14095884439404660071
O	1.67910532862324068937	0.33183947980488348595	4.00880517279518766571
O	−0.46223059305676861275	−0.34831218249882933691	−2.39281119005672504585
P	−0.40289770349655451565	1.27045698682355556741	−2.03066211439888455459
P	0.07075991841527839998	−1.51620766620088764931	−1.38250844751504686769
P	1.20550361603540445721	1.52707375257611555064	3.00015325652871123197
P	1.56655367936605505186	−1.28538318876627921661	3.61535713538237057918
O	−0.20238002456841372445	1.12119828879904570584	2.47692862664331414635
O	2.08714043693076023445	1.38730800096860606985	1.72189055545925073787
O	0.38860128805552973663	−1.85954063842895611636	4.35257618172720306404
O	2.90564979325112515696	−1.88400456704783580975	3.91370388354354048488
O	−0.78105383609099265918	−1.37206465839565816545	−0.08198592548545791114
O	1.48267248697760245513	−1.07490900819280721379	−0.90543960824787528274
O	−1.80253208657458552366	1.79989367358310259348	−2.21335583531289525894
O	0.63311180785462550791	1.89697442795766924029	−2.92754376325770415690

```
O    −1.16380775935089508799  3.44377365930185952436   −4.27344432891129866192
O    −1.58725701741969849046  −2.72798570179989541273  2.51052719955904546367
H    −0.31011621620168150493  3.03935930246659991028   −4.01042948738349469551
H    −0.90464547793222049865  −2.39228807168269241856  3.12735742568339958325
H    −1.33457591458997848477  −2.30213936629372151188  1.67644329497063115930
H    −1.68558675150111336727  2.96888965552980055307   −3.58975133559935155603
25
converged
Re   0.87221724125176658404   0.16779839472330207450   0.44829157200256336546
O    −0.54838220456126596236  1.78221663308293498673   0.96895712559553981436
O    0.83062719833685338955   −0.23904674547857956979  2.31836512846266584731
O    0.87366350005359083575   0.56965735532254191398   −1.42383585562760472953
O    2.32516848053502522831   −1.37755131136707831274  0.01381349797536052249
O    0.64334877319027194975   2.16288490316364301336   3.19792014564914595098
O    1.34217130631197245627   −1.79825199784946732073  −2.29153746080980802446
P    1.01630236605373447389   −0.23753554469509324809  −2.79118209273476391985
P    1.28511357282089866416   −2.26462467398035194321  −0.73050523181316950883
P    0.59153243439555491801   2.61649854835407680298   1.63273953975902275104
P    0.66779940655072278499   0.56521133684321034352   3.68550390235197689037
O    1.86877231072485239238   2.01599077350046940182   0.97659706271916690845
O    0.45522595964322998263   4.10032345062385239487   1.63313676068567592559
O    −0.67363494610658103401  0.28659999016539267069   4.31167719924062353698
O    1.85968410070133627343   0.39563852070811522665   4.57353920325842544514
O    1.51387452998568949880   −3.73703821553215087548  −0.72635817752917186674
O    −0.06971031582395839643  −1.73815487319683636969  −0.16163081914672788875
O    −0.30024481749636966477  −0.28722610730142239532  −3.52152249681987594698
O    2.19650685974573933734   0.22380693868866397001   −3.58651055140601027205
O    −2.71778329185066436224  1.43251929714075010303   2.88547869382159172247
O    −2.11233770287274591482  −1.89566149349496115839  −2.23353436030706564708
H    −2.15932401490410574851  1.61940980801995304539   2.11181640635629941372
H    −1.58232429367187443781  −1.94848627364648008253  −1.42006179952998201621
H    −1.52471141545242905302  −1.31108089647221626173  −2.76443502560209930508
H    −2.04735502595536633663  0.99720218797906046149   3.46007763772445908756
25
converged
Re   0.69316518341215727084   0.78549007149397143124   −0.18903351619869551703
O    2.07605149917287690187   1.56868139846617893340   1.32121053199775695397
O    0.51882960911560571660   −0.73963623567504543921  0.97761657382895883472
O    0.87183984044275208980   2.27801602607764674957   −1.35390384097943994313
O    −0.70757529102100846785  −0.06064124420332370202  −1.62607800572853422594
O    0.72205300604521549523   0.50867002877994771382   3.20664125841810943029
O    0.56375961239980965090   1.00971905590404897879   −3.55760864642908103406
P    0.92506189664750138046   2.52337669974226042058   −2.93659558046942192888
P    0.43558889939779465994   −0.33848462705300486064  −2.65692574760927513466
P    0.91118155250611765350   1.86894282728690774853   2.30881330424270636570
P    0.33064380892556727298   −0.95015124053592980857  2.53225412947629058280
O    1.10794871827838381506   2.97511279662503946852   3.28895108340068142994
O    −0.32407960019021253562  1.93331143559371443352   1.36890240837067378443
O    −1.11683089818846403851  −1.23884847657144181454  2.84631593305052765785
O    1.31324034271075196223   −1.94492565025508046261  3.09681117636873048937
O    1.69218675458416534241   −0.38529552830675900532  −1.73920636803755934707
O    0.23668526483923407744   −1.46075299097241195767  −3.61726466446132732813
O    −0.17411977155533850392  3.44020096481200354788   −3.37926998280690638055
O    2.31335892352170668929   2.87186572050867949812   −3.37924539422069880601
O    −2.59022460078790928151  −1.28457766476969448277  0.39279649017419304702
O    −0.44606610795760603949  −3.37310802122075070741  4.52419705560820162304
H    −1.98890630032286197526  −0.89763501692870462101  −0.26303883508083941711
H    −1.05897320879598910714  −2.75975319482406256100  4.08235921924511124814
H    0.36738216343368068628   −3.00209607578417125850  4.10292158349027857867
H    −2.06600130617514166076  −1.22638104230514666249  1.21517983333402335155
25
```

converged

Re	0.03627706266532754187	−0.48985051796627743226	0.21641236488964932239
O	1.18330815269497380271	1.13022073731002148378	3.83981061499508546930
O	−0.02050327160554785144	−2.14405068664889997265	1.16550789891472916793
O	0.09478482722308949338	1.17991075025524216890	−0.73686248578811275323
O	−1.11072629252016130685	−2.12926041547605127136	−3.39221633045771309156
O	0.68278983272866611287	−1.27215114741065060677	3.47401700253767442561
O	−0.60941645647987430223	0.28800136539421372639	−3.03470728765251651637
P	−0.17676510077519397290	1.67017789622153367546	−2.22088085643243005052
P	−0.69091710744151346724	−1.18036394587308768322	−2.32164173346215640947
P	0.75976404958895660258	0.18238172192917939674	2.75244165306509547264
P	0.24386691341619609608	−2.67484606804693569870	2.64897360687010641200
O	−0.62068950095160380087	0.44120778195522153409	2.09467245320363559813
O	1.71097314997910876500	0.04049064746634679218	1.53574081098147519064
O	−1.01834041594613111670	−3.16559288936111293822	3.28733004320422184108
O	1.43031678612211488044	−3.58655620109589623468	2.70032299222087424084
O	−1.63175898343389103040	−1.01311416187797798116	−1.09233252307455241947
O	0.69443752612816012082	−1.41304235844528691679	−1.65003387461129324976
O	−1.35654373366910085963	2.60511374884181634570	−2.28843867931351541145
O	1.07993170598323984244	2.18639819519029199313	−2.87261063003182082198
O	−0.07574572461948753110	4.46808574974578842642	−3.80433455758723582107
O	1.50600835245323838585	−0.19293248573518378963	6.13622650794881785430
H	0.59890160759947319580	3.77782838829280365189	−3.62799816785547957210
H	1.26090732366556634858	−1.02305964664810145059	5.71156715014861404711
H	1.41812680417821845857	0.40135711257108358518	5.34290272969173063444
H	−0.78278750596149104801	4.01474642412970261773	−3.29706870342041513666

25

converged

Re	−0.33879399677312482808	0.06197054921449915432	−0.39646710243038868482
O	0.65092038964555976044	1.71191325355706802647	0.71070736750856333686
O	0.83863368126241211176	−1.09244481344477040174	0.61431878452147992142
O	−1.48818282005772473120	1.21129488532403972734	−1.38404111280072639190
O	−1.30407267551653816007	−1.59119107726870678832	−1.42286470343881199518
O	0.86732643212088178508	0.25295720910441771290	2.79851901938710589945
O	−1.60214714603023700157	−0.18422483412800805258	−3.53146327803181003802
P	−2.19703759675019494324	1.21299645973833336576	−2.82165709301641776108
P	−0.61371370313087780790	−1.23235701641648653926	−2.77410380761332309163
P	−0.09450803025084450470	1.33068952774912996340	2.02435928581740531840
P	1.57969126509095292299	−1.02899621720576917028	2.00968835406031809399
O	−0.36691824692256247431	2.41016187008811577641	3.01413735848894592806
O	−1.30700753715209305739	0.50803407248607401758	1.50829614149922819344
O	1.27649645127408439826	−2.24077556775848174553	2.85550938173016355393
O	3.03915386900993667041	−0.70993576911103839500	1.84643747586650230907
O	0.61933047712988753553	−0.40743137945068025330	−2.29659837542648892494
O	−0.37166375875544754859	−2.34261459710668340506	−3.74087530635491027908
O	−3.67987365124087606461	1.04449839985255299446	−2.69024357457692353535
O	−1.71922634941001284403	2.36018820698120412871	−3.65820947751289260452
O	3.50571091534175760529	1.87796320883704037641	1.07954574280991200119
O	0.38851660779065244622	−1.51099010124183585901	5.25368752363986324383
H	2.56938321200459141025	2.02919208255044969391	0.86229817622765136953
H	0.73411102693772867234	−1.88481408931815330732	4.39508667980264977615
H	0.17560838806846756910	−0.62388725911432252325	4.94414530033441401713
H	3.44446279204418326358	0.93890301196688219498	1.36658723849295560271

25

converged

Re	0.01672417709340856540	0.30965253754583810242	1.43962580384858074289
O	1.54199331509970871679	1.76436775814715174349	4.97867771692740390677
O	−1.05379188060904027679	−0.82313326367265493566	2.53213709007469045531
O	1.10137568777240946183	1.43175336371993289220	0.31599395267588514802
O	−1.57250553425127281848	−1.06022473357675850458	−2.12986145899354717415
O	−0.19691132467925376304	−0.01379689564050272377	4.80866542824606657547

O 0.16634200166129653464 0.70722282622873566815 −1.96192030199137557034
P 1.34953229130533425284 1.65502472842914971629 −1.23726975172823316917
P −0.76014251354249351422 −0.32146437362986829411 −1.10558662386975914416
P 0.76368109450347176903 0.99250246671821695799 3.96675012667627191476
P −1.29870422789424022625 −1.06066035317464413268 4.09713689761529842315
O −0.16970139308414705637 1.79630688344272937407 3.00940771648121829784
O 1.56126854124676373026 0.10542509142405648292 2.96298314699910614323
O −2.66572971058725016746 −0.60235771319836439730 4.50433997078418890680
O −0.90180215995966839237 −2.44658219965925383121 4.50416024106215484579
O −1.54424172173619189685 0.53440434141048198668 −0.08130929032441752691
O 0.19445352913703028985 −1.16736632245420413057 −0.21979248972249593175
O 1.07753183046669276379 3.06400151715291535481 −1.65695175574746622615
O 2.67223844749636407769 1.07471570046024833545 −1.66189006550302265097
O −1.19251399731751650535 0.03281060225565409633 −4.53755818194326199233
O 2.74593641080347206085 −1.65914785526215835532 −1.48992178722138679525
H −0.52402036544337859958 0.62761854702364228142 −4.17907472507889554691
H 2.81105981753515887789 −0.68167666416242089777 −1.58602527703717566254
H 1.90238199810918806598 −1.70972172149414602949 −1.00858909333885260473
H −1.41825432797882999836 −0.44857425214890583876 −3.69732730049005375861
25
converged
Re 0.81434990601752144102 0.20884224111151361569 −0.15101313637310359583
O 2.13073608656701862785 1.72858658519624874828 0.69361053679387407733
O 0.97288441156951988020 −0.68437520641013915856 1.53921111415372058318
O 0.63092330044227495911 1.08043059184099021763 −1.83250611775642990864
O −0.47748480639450163743 −1.37176174369654768626 −0.92784079242525385744
O 1.16413509654348046318 1.39096135098992790269 3.02511768796236735213
O 0.45939879651904236280 −1.03043412928847799570 −3.27302652031318430659
P 0.57799466214153072396 0.64018748729342844772 −3.37042473870310033135
P 0.64342742830333898851 −1.87367122230637161984 −1.89620034993254504840
P 1.02682935534294816904 2.26540547228165722871 1.64882966844285672181
P 0.89240150273577567575 −0.25025729939958291093 3.06569323014971351071
O 1.11446000813440182498 3.69613382656658062686 2.05561004769498856248
O −0.28468961545795901102 1.78029753712013572020 0.96101746999959991591
O −0.51503299597988561054 −0.46879521876806912184 3.57018363389996862267
O 1.98994751992980400956 −0.84176315992820227585 3.89057342668991745782
O 1.92855271505097869422 −1.33074069100350000916 −1.20492917230342455603
O 0.62244564587509032538 −3.31278358300345310283 −2.28366217621031752927
O −0.68033292912874743230 1.11541362986085745845 −4.03035920355807864013
O 1.86634320211217197283 0.96042020543616712924 −4.06544518259269604954
O −2.29255472601006804467 1.55413166589293938458 3.00756774952792582667
O −1.78866518829190579432 −2.19971242972033076413 1.59870601179290328098
H −1.76081585574178056675 1.82511299748939626042 2.23952104498453197223
H −1.35377669970469050043 −1.97067353156773150680 0.76029782511177956827
H −1.33696829005481032659 −1.61336813550990987309 2.23284578119598542401
H −1.73830853478998759165 0.80351279128266095420 3.31342215016892005508
25
converged
Re −0.54153873191813839405 0.25192204547550750560 0.04392128696873154325
O −1.91309881343847321133 1.64907479595640404924 1.02468089380756488538
O −0.64443090114740875229 −0.78282542045321601432 1.66179243552713140630
O −0.44324534541779575525 1.28305642589412061128 −1.55239118826632194192
O 0.83185418756654394556 −1.14890267745354845985 −0.90779487847808137957
O −0.90474394856862261349 1.16037236658002851364 3.31548868650392014601
O −0.17696457521441083527 −0.66281457487907768922 −3.19496468259803734213
P −0.39766268896885598183 0.99929240835168708923 −3.12690531960195094996
P −0.27523537328010605485 −1.63806269994400954815 −1.89985574067775120355
P −0.82053300663484951549 2.13849763985115526310 2.01376483784040249958
P −0.76589269476643750956 −0.50424293973265943425 3.22284976180895110787
O 0.50238927780054076777 1.75216355446497007442 1.27304545429022852865
O −0.93144500510763283962 3.53066569226515269975 2.53465131539901156188

O	−2.01145454092128606050	−1.08340808349901918106	3.81269243017954018171
O	0.52604443789508037455	−0.85792940237256787306	3.91711296263351460567
O	−0.16784719491409599845	−3.03184619556853318656	−2.41666712360293001183
O	−1.57630965968916081366	−1.24874012046151228184	−1.13967535942749442768
O	−1.71787068934364595130	1.30332309751439900936	−3.76697393857587137234
O	0.81438000035039581626	1.61505875182453273453	−3.75598171563993865618
O	2.54209998499750788881	0.33443615353935929324	2.57278785656206876453
O	3.30245376272327861855	−1.43156730729252146261	0.54872482748724171753
H	1.95902442967673140473	0.88565654513650993174	2.01607902191578247653
H	3.05657694779074651237	−0.83684843739550340835	1.28817834839965250637
H	2.49881912903706338014	−1.40595734287383322680	−0.00046158620714564780
H	1.86083100722359517931	−0.16927428021003873559	3.08270141802801633801
25			
converged			
Re	0.11513653937749895872	−0.23907549598602195751	−0.33554307930930526993
O	0.31624293199663000564	3.70600876343637253285	0.88879908737325852286
O	1.24445348633435237673	−0.58473866313918232418	1.17204096452691608476
O	−1.00043376700754338415	0.09519215612390030667	−1.84162037724041671716
O	−0.05178246515018605778	−4.20089927000114737865	−1.46449028967256955625
O	1.41588509999431266806	1.81430638234940566633	2.06472037669426988771
O	−1.21884999206635824542	−2.33242958075537787366	−2.63073334420763060848
P	−1.62339049124800216539	−0.75751859358141582224	−3.04490103673413425867
P	−0.24001387326375353548	−2.72388384102563163580	−1.39452794202964458492
P	0.48150167181119696291	2.21456014016419056034	0.79033046585891608693
P	1.74543935466079402907	0.21390951181375589263	2.45141263021013866563
O	−0.79941465946136225362	1.35094202943384122939	0.87761836013482463770
O	1.19386934501488917171	1.67779745977760930131	−0.47834752561054705300
O	0.89565274031442576597	−0.14472463632362453367	3.63767926963330978651
O	3.22879019853897508696	0.13950706269799861836	2.63295945617022741914
O	−0.91392366517541212545	−2.14363156684570688171	−0.10747375524361879595
O	1.02689612937073970755	−1.82702082609273008806	−1.52281911483835297183
O	−3.12009618003844346745	−0.68841229131190762036	−3.04190564330353208078
O	−0.93636223343816382680	−0.44489948770388448862	−4.33923065244357530901
O	1.76559017116418903726	4.65206459134878258510	2.91735573641012102541
O	−0.92691380571105042030	−2.19658830315259390176	2.93349287758036858520
H	2.04809964662523169565	3.76113336739806047859	3.15381725008396962551
H	−0.92418433340450611979	−2.18850196029482235716	1.96360544481930188176
H	−0.30982833722299774060	−1.46655338105795163273	3.14699915570523369723
H	1.19383645725624321088	4.41455643802941022358	2.13756167912516303886
25			
converged			
Re	−0.27215287864834414311	0.83328627968342861010	0.44786516949836002732
O	0.97883216236186509907	1.87109841025154266525	1.90407528079377019736
O	0.31397630671815279779	−0.79210763139859907689	1.29396636731545422450
O	−0.84980222114511028142	2.44485739866646589746	−0.38761766089140897718
O	−1.53315876067407619310	−0.20475472104329611733	−1.02416363296977963593
O	0.48441433099777342353	0.11596172651178862645	3.68711925189993827701
O	−1.03554174700745926785	1.57547465540800635253	−2.78928909471707431322
P	−1.27341492034370107334	2.94761664568122228047	−1.84565851828378235666
P	−0.56104150622215409605	0.15373691157908853100	−2.17829065147222422638
P	−0.00552210723088314426	1.54512174330739027184	3.06566073282357809404
P	0.67839950252121516971	−1.25450856395489873663	2.76749665924238108516
O	−0.05977536541460157227	2.49257867573167857245	4.21550477282146296432
O	−1.33147455601153441584	1.25327153335276753054	2.30375194758026902164
O	−0.30376748303435885390	−2.27227093019379511318	3.28692985662553471826
O	2.12682777487442242759	−1.65257331555595565753	2.88110735880152413202
O	0.78837009411694924932	0.39695191826556258663	−1.43972769925319377116
O	−0.51109238924903843682	−0.78686222851614018303	−3.34877989766339423383
O	−2.73470000097069521061	3.26931237605368973931	−1.90581281626915810534
O	−0.32712075648283622797	3.98925088095308311154	−2.35705610041796465026
O	2.14752013338035085965	−1.18670018739309135469	−3.60866274785135399128

O 1.60697493505586574969 −3.97005208250127994418 4.21794575637718160976
H 1.16406120950535618697 −1.10929071239687182526 −3.64883227823169775661
H 0.72633766736341665027 −3.58802005487636943570 4.01377999438113874930
H 2.10689563530529078150 −3.24481747847822177278 3.78668910631763067087
H 2.28215495184001282425 −0.72546125971118335940 −2.77120115747272688367
25
converged
Re −0.42032807500594737693 0.88919884795709902559 −0.57015981744006882792
O 0.63196380017572195698 2.57276013151946347790 0.36006902616978780918
O 0.47598332719395392010 −0.21641488144983128672 0.74995318350196016333
O −1.30205574502780097035 1.97906629403213218943 −1.85633966690459972781
O −1.45547869704800847401 −0.80477537250279440606 −1.47786355611534880872
O 0.50419600765510885765 1.48971939705689382372 2.66743478573721182201
O −1.32986287547048531721 0.28797514523281053656 −3.78136852885272745084
P −1.78995839104674070441 1.84758756294233150719 −3.37833795100030620517
P −0.57123284558497966490 −0.72854874167400296425 −2.75998621570105662215
P −0.26388576040512801146 2.50753652863999176859 1.62822633638245761745
P 0.92672621977635893931 −0.04449168890320055747 2.25318970079768066128
O −0.47781869268752275026 3.76790846884638153114 2.39452688334494290245
O −1.51848180215373074198 1.74045204763676109039 1.12686739650748535269
O 0.13683156231834173178 −0.96351100804453904125 3.16410055177107762958
O 2.42070405099549379457 −0.15792811768373543613 2.40261337478301539861
O 0.68903165385493014838 0.02879968336838541892 −2.24086951439847448242
O −0.35227213591308426111 −1.99108067081938910903 −3.52340323339132721614
O −3.28415494316330569546 1.90150207546890470667 −3.47110272960145893606
O −1.02384562969461190285 2.77546021427760658540 −4.27068837028765013741
O 3.52521519350022671802 −1.94639671084489718211 4.06621045808115955111
O 1.09323180242995654510 −3.31476237539568607815 3.50687666821207066903
H 3.20296506727362650935 −1.21069763304289823580 3.47908466822509865324
H 0.69642074654847185844 −2.36951023955439499247 3.44812813194026590580
H 1.35790456870282838509 −3.42813343807286674547 2.59092068780219841884
H 2.73440157792334037623 −2.51061549781387949309 4.05871770825398403559
25
converged
Re 0.00019992959248854580 0.43081506644379530258 0.55688482519497095602
O 0.77595437663234312353 1.83852820425516338965 2.02063232064767950291
O 1.41669145381582417542 −0.74397053476930197213 1.09193360504894187990
O −1.41841627190422636673 1.59005612797806716863 0.03153016175113326769
O −0.78327087978254561484 −0.96213669095646403573 −0.95230886025883365598
O 1.54966975044946009277 −0.03585623330850656876 3.55434495220828194917
O −1.49246093973539850630 0.97888035536525075386 −2.45555573122855941293
P −2.21151680926068161526 1.97260096471780421723 −1.30207698043374908714
P −0.29469828570462197614 −0.05063593094544899254 −2.10758881849388401974
P 0.30863080882682875083 0.96591481471834383932 3.22164969269995182799
P 2.15596387839881797888 −1.13500735232724769830 2.44961715881965380603
O −0.01597687139138534440 1.66770862221416393822 4.49524356541047076519
O −0.80627143078299545742 0.08289243830867340512 2.57568911690359314548
O 1.72266776938283050491 −2.49995415462405956575 2.91752826726621439235
O 3.63059163053647848685 −0.90022189419309783798 2.36856150720733804604
O 0.84649912850314179558 0.77459485464516142361 −1.44267530088951190237
O 0.04998907621318482430 −0.71845402929342172538 −3.40911096667188173726
O −3.64957445203594366134 1.56336196779232072807 −1.21896318704312323078
O −1.93037684480022408984 3.38464054105970246766 −1.71431217961346815670
O 2.51308298262708973425 0.26622880757682659825 −3.89795909533348439169
O −0.87952691720346209969 −2.56251729885872725490 3.76566693924223061529
H 1.61634916175051257525 −0.14678293008741019610 −3.86012205462429580649
H −1.07724476292374293784 −1.68170824002693675148 3.40382349220242774734
H 0.06312486947418656513 −2.63252832758877675090 3.48841250657254153822
H 2.51611965034437412569 0.65465088366431212208 −3.01404493760017277992
25
converged

Re	0.49382248024582986456	0.28488969093035837465	0.47146635467201558622
O	−0.06656759826582592243	1.94376735149616663989	−0.83853333612624103566
O	−0.96959652256377004687	0.84648498871609012539	1.58123364177478120673
O	1.94203172636402987550	−0.28250061666852294140	−0.63535200281773884790
O	1.04750655066972520046	−1.37743715424618295984	1.79518692937665580445
O	−0.78338232851791422817	3.36876027017926915619	1.15092316947346984257
O	1.77448640178065364381	−2.80643474172580864590	−0.19674617228416746428
P	2.65755649265236293388	−1.64903396051593764682	−1.04823613253354808172
P	0.52757204527971635066	−2.41891474073240075882	0.77091499114086337041
P	0.47156858715539812232	2.99954666047133811091	0.17221899385939565263
P	−1.62070530013147040549	2.22339049346890682557	2.03432653968044174775
O	1.46470180145291806184	2.16493912066326066324	1.03070739572183223132
O	0.94408936269942034247	4.30264677449246057961	−0.37856817610972515187
O	−3.05378663129440353075	2.32574891300838171304	1.56638273421433682486
O	−1.38626446883674425692	2.49921718780175483587	3.48468322954830433957
O	0.04472320488934813931	−3.73179951751971872298	1.32285299675448397849
O	−0.47983757159534845549	−1.60536980146726815377	−0.08245889876189338441
O	2.46277292257346847038	−1.95285204883834984102	−2.50133171395019360261
O	4.05806110115969520535	−1.71337885170865300566	−0.52254788721819112052
O	−3.47349444024187814506	4.44037052302501411560	0.00431761374150940735
O	1.52921773453719422875	−5.73278140878999220575	0.36788943106817395545
H	−2.54110437821971713035	4.51679532146531492742	−0.22505927497310262986
H	2.06496492886381100007	−5.11484454609302918726	−0.14263835804741054059
H	0.91263809943078988418	−5.07167602405886519534	0.78472233654134404546
H	−3.41477420435672662791	3.64156611136419749997	0.60044561011438624121
25			
converged			
Re	0.34674705705603792172	0.10807114709414014642	0.72888272885490434927
O	1.08547052406920552237	1.84245606456517818650	1.82664589807206323435
O	1.73942321070355743196	−0.87615253970875772715	1.57745128632242281874
O	−1.04046661194303280418	1.10775090079685645250	−0.14134801422596227072
O	−0.45307945350070977231	−1.63637506157101575432	−0.30194797182055960194
O	1.74378449058636686431	0.38058114261339626161	3.81322525619763430527
O	−1.16664160296676477913	−0.25416855416455375050	−2.30803961022752535115
P	−1.72691646210405358453	1.14403858953822457067	−1.57797457909459937220
P	0.05043472334209295194	−1.14163961813521064315	−1.69445390277340401930
P	0.55486338221381481528	1.28463923312070726901	3.17871989278075961494
P	2.51578576533357400891	−0.85560898749313007983	2.97666285771006444705
O	0.16849368371562439828	2.26778793051728611019	4.23150696725910968610
O	−0.54583688771450233901	0.27909555038079092437	2.70374940159955601260
O	2.26970184568207100639	−2.11448088476408146974	3.75006167726964134701
O	3.94307943151066053744	−0.43782395477489638447	2.79894436747203290139
O	1.18073858924103558543	−0.14136861009786333421	−1.30440274689386459528
O	0.36792788794752501191	−2.18470675567783922943	−2.70912351905753956416
O	−3.21410043831765834810	1.01700369533399825883	−1.49092789790296520458
O	−1.20553383193000951401	2.29970467076908047233	−2.39205983892273721381
O	1.38712677119976302009	2.02813064985569280196	−3.20132928137090111420
O	−2.08735795557602710204	−2.08959542931553476919	2.04014847248126995893
H	1.54889457149209919251	1.28319648320924262208	−2.59706796625266767009
H	−1.67333447714852678878	−2.06868147975409844008	1.16222290320670751385
H	−1.62924441858351487511	−1.34699697283906316692	2.46522801154370796439
H	0.44624021729724805407	2.20624281696986690449	−2.96797439853445554903
25			
converged			
Re	0.19265640301456335437	0.08457258449992714755	0.42161781280673982231
O	1.54816382823954756809	0.47224635247828283813	2.10509705050561413131
O	0.37792244616159309523	−1.76404022767321388976	0.86777743801843720828
O	0.00793680827335627170	1.93330609708576450778	−0.02454224257214843163
O	−1.16271735467961767618	−0.30314848231979985016	−1.26065164245778249530
O	0.60144824635996785300	−1.51084180041397719840	3.41259988714200401816
O	−0.21840365681087900440	1.67989138438450025248	−2.56912340478577050362

P	−0.18390847142440339135	2.82823061238185102795	−1.33201576917658104371
P	−0.06769687086819983768	0.08796337883818526604	−2.28787333874549059232
P	0.45229893175890256707	0.08109385578528474636	3.13101369790712213970
P	0.57234845853437643637	−2.65860157186436829591	2.17513528418522650298
O	0.53216933784605424673	0.72368382427420585579	4.48811886546656957364
O	−0.86358799390591556300	0.28685611283393130932	2.33531676854506020646
O	−0.62192562659161132466	−3.52451364730463057029	2.43149345964604668424
O	1.91178675309933199244	−3.32755956178378031751	2.18804097224475402328
O	1.24861458142519832215	−0.11738668228229201529	−1.49321075588870710860
O	−0.14910433977466733269	−0.55490406264818958881	−3.64485946374148372939
O	−1.52111222542040791517	3.50180987045735436780	−1.34532914155952965096
O	1.01334451325146646106	3.68979581575800441584	−1.58861962231406295132
O	−0.52760395921802283326	1.30688133897201264055	−5.52022947492904236810
O	0.83121305097282915941	−1.14250338850378208022	6.37318712709325740917
H	−0.39100134932216390471	0.53106097939291108112	−4.91202458805360464567
H	0.72272955633866975411	−0.36541154107628059933	5.76090173529749094428
H	0.81646894543414316736	−1.84160238358296024508	5.70952972797083102563
H	−0.51584000638000349070	2.00422116348770273930	−4.85455037832871383330
25			
converged			
Re	−0.02756712192141574924	−0.01355599249469029155	0.05183115180881847639
O	0.43358381871382278838	1.47759674080187575207	1.60054325900850047404
O	1.75199588025820873760	−0.66415808144352295184	0.31173541019779732464
O	−1.80370129313202332000	0.61549249305633746410	−0.21987185260303604428
O	−0.45331830666806960384	−1.49259850571914109985	−1.49405905352311196843
O	1.96973457100300342049	−0.16344542357081137673	2.81325871647679104370
O	−1.91271548783446476705	0.18849211835732312958	−2.74578763504321265998
P	−2.83821937410481162090	0.69359811777756996687	−1.43989220772229775669
P	−0.40918368399151072712	−0.41081938901679587728	−2.61340705066900236275
P	0.45033421559873920703	0.39732810307917904025	2.70875035479540038352
P	2.85437713176590124320	−0.59506139718583339615	1.45666845817690981946
O	0.08806335577415523586	0.82718526307074413673	4.10250868904551424521
O	−0.41676802510363819554	−0.74763016292679840280	2.10727927422452232520
O	3.44786595927538819240	−1.93441350271295631202	1.76097355040974701268
O	3.81540300013507893695	0.52360173563499212701	1.16520449150962668305
O	0.44516497167213153752	0.71253138509542279788	−1.93837760744611564689
O	−0.01160912058700808158	−0.84671770598680484721	−3.98228315751091699326
O	−3.94716905569974896295	−0.29901032824784629094	−1.26979473424501709822
O	−3.23537111592623016776	2.10985655380943981640	−1.72449507546890679066
O	3.25082419081240914238	1.68921906126580223528	−1.34894464673134129029
O	−1.76384663228785520950	−1.01583885462315071102	4.76459657661704927278
H	3.41071630545425907854	1.29206340257985119457	−0.46768505111449371059
H	−1.75806776465206437976	−1.32109412854149743666	3.84829973556776527843
H	−1.08807512163601205479	−0.30123653420520324353	4.67010980012794441052
H	2.35374870410408831845	1.37971501098898285953	−1.55036139690446983508
25			
converged			
Re	0.44830721958907993052	0.00377767341750195884	−0.23673199187559118561
O	−0.24334715225711220365	1.96085499979251332014	0.82096143052494863213
O	−0.78412946591298404719	−0.80988588405204875453	0.99866326765570034851
O	1.67416857519636219642	0.83299542807251047982	−1.43642121842399839160
O	1.10902703254320034176	−1.91723192081879445503	−1.21026052176247778114
O	−0.60358062653764532612	0.74259717401467162379	3.03366737212391290868
O	1.47835462858938826436	−0.73345769912664704471	−3.44992645193831704020
P	2.26647968176170477861	0.64465661847601529733	−2.92956487435094059535
P	0.39749681985621254654	−1.56685170879662849508	−2.55314177597978142487
P	0.48449803709804972751	1.59849647734443345293	2.15111250038691714792
P	−1.36806982985452241230	−0.61741932832603907411	2.48010419924920366697
O	1.57674082528362835554	0.57311831781157396293	1.72134295665514414075
O	0.91901958908441239160	2.73031454983637589251	3.01545284383425427066
O	−2.83805318068693823719	−0.31022369386496440047	2.43853515521994124171

O	-0.94881469338907309385	-1.75214444313427808453	3.37104317910233719502
O	-0.04675613023745903152	-2.70560334239091426767	-3.40840604173121075604
O	-0.69038061605383438391	-0.54406836413848080891	-2.10249194490738977592
O	1.83518413571700200748	1.76789638994741560296	-3.81986717862562974446
O	3.72965218534211118140	0.33266002905182262550	-2.88626696650182035242
O	-3.08508751183510243621	2.22834321628146936334	1.32998680678235170483
O	1.79703571959328978203	-1.50459438288052549382	3.73379546615210022864
H	-3.08501493841276230867	1.33136127922130631518	1.72731903877308545781
H	0.83189507387414318718	-1.66316791845010136797	3.65651950473597597835
H	1.90284383403497425746	-0.80941061468600661311	3.06037378427053052832
H	-2.15126922194733394278	2.28808714740737384474	1.06100145432344250551

25
converged

Re	0.58597014734286589910	0.44051032298536768606	0.91948085599839957105
O	-0.36930323799110653660	-0.74342490022995311172	2.47571879525656157739
O	2.15708760386846476464	-0.59568122943912182876	1.20467127805086504644
O	-0.99459737803676140366	1.48166351438353949277	0.65252387012622437013
O	1.45670021509305325225	1.68583077251861435109	-0.68001570937444055787
O	1.84158850410208740911	-0.97108957568983411690	3.72110664439250138358
O	-0.79587302055567110681	1.82133733607039682845	-1.87873959537260959429
P	-1.83538428180779633614	2.03159981290623425920	-0.58082095005128575593
P	0.58856172758629443642	0.98350139694282101566	-1.75648119838713312468
P	0.49629169929330063660	-0.07020935148296733597	3.59119625321086299863
P	2.91913221878547846089	-1.23229419575378029705	2.46138169947747664779
O	0.91508016537698100290	1.26459069967256443334	2.90687890278587657278
O	-0.08734289964792626981	0.00761445714963683573	4.96059312601174706714
O	3.05230584103668656226	-2.71767149070686953038	2.32040589519809747898
O	4.16869918427004648720	-0.46788802589227712625	2.77610193448557396323
O	1.14753408599011375024	0.93620861661895671268	-3.15083218141676946189
O	0.24219504966540392199	-0.38281996481482205574	-1.10401551195392078064
O	-3.02818150545630038195	1.14624408719925141575	-0.81033170564525791590
O	-2.09177751005936629625	3.50303095316168944251	-0.48803004430758373244
O	0.96275970902148766406	-1.67309675204877872368	-3.78489233460214036242
O	-3.14774252059572923201	-0.82474965174740710872	1.22613851536852869195
H	1.06099521457048817652	-0.69437269503365373069	-3.68384830227005144465
H	-2.26722596031797785088	-0.79699062800202558066	1.63192790220014982694
H	-3.06884463584602640296	-0.15444538933362139677	0.51636667309922168467
H	0.69757156945892651034	-1.87629812471617873904	-2.87968481329643166688

25
converged

Re	0.36609790485430221407	0.29650453276086535803	0.30599503086927737350
O	1.71088475659017968944	1.00979933307725167069	1.87159534094917123781
O	-0.61539908169471713162	-0.58584867057386313949	1.68910131818063846687
O	1.33195228685614996955	1.16185631939077915753	-1.09069561821816241043
O	-1.00512072625995885211	-0.37599529359978411946	-1.25597857094596965233
O	-0.20480351985601882858	1.12050429832203701785	3.55391824375304743455
O	0.91715638131395438304	-0.58960365234030409631	-2.91680304034965232063
P	1.65227898750764357061	0.89471187458048373475	-2.63423279970874624212
P	0.04384803415316723685	-1.39137589866675259742	-1.81149142639010052314
P	0.63524303697443651462	1.97083316988194057195	2.44730104958999339360
P	-0.73292907259931827646	-0.44091408696962075542	3.27169350395367519724
O	1.09723904278473471940	3.21044231936686719209	3.13589429230898453227
O	-0.33194836017973183839	2.15383263538262248460	1.23153827329225706855
O	-2.15148305940969830630	-0.48545503896818198442	3.74595224258904879733
O	0.23244860268926376135	-1.38452853745421999854	3.93456929305291680521
O	0.97729180055530517901	-1.59987085175597232301	-0.57758179563689227010
O	-0.48006268499342052980	-2.61012338283688238860	-2.49067952190602248663
O	0.96450076670662099332	1.89628292722304458984	-3.51051357155266341437
O	3.12028130608288600811	0.69514514008885175400	-2.85805503276392736112
O	-2.61704217519582549301	1.93996722883674799220	-0.54342060010233095291
O	1.47766061583911834099	-3.05432772876612723678	2.02592829129366114316

H	−1.96750434585092892625	2.14111484065766877194	0.14951041839505213593
H	1.32479125800745767272	−2.59750947660132691652	1.18401284129996420269
H	1.05786388860110225352	−2.44418098586884635637	2.66773538098634244164
H	−2.19704565303791410358	1.16983900071759339134	−0.95849353866332442919

25

converged

Re	0.34202468799897711715	−0.03657922812189520756	0.00814164054096294665
O	−1.23833977902817538030	1.44894369134868483684	0.34211486184312056569
O	0.86033197748221668100	0.30384610069436884805	1.81542737114255503528
O	−0.18339411415338455202	−0.37089909439127599633	−1.79486996058950776245
O	1.92374233115079329437	−1.52158441272455724480	−0.34058477112357571359
O	0.26960946916605960100	2.80272563269270369446	1.90066975345217570315
O	0.40530623233288792795	−2.86190522956784088038	−1.90044125836639477001
P	−0.18990137952570182445	−1.58846431358510864484	−2.82937994346315635497
P	0.89370951170815071674	−2.68134247451708018772	−0.36978456363976580645
P	−0.20267983150920729218	2.60370867600139588305	0.36052302715464801253
P	0.90000425573459919004	1.53887226970571644458	2.82638785430144912070
O	1.00117230531520973180	2.00742517762489125488	−0.41510671674386950825
O	−0.68760285089075456888	3.95852377743796601806	−0.07560540486375444402
O	−0.04655259957420421202	1.34306048141964540221	3.96942956308636230034
O	2.30624358482219804500	1.92582612923876506095	3.16445009596792425910
O	1.37950691630054822490	−4.03731567690131143422	0.05855520226777494175
O	−0.30787671656544468579	−2.10233066067661278353	0.43479143081019339112
O	−1.58543723494981847466	−1.96365443461339173048	−3.22182169872124513077
O	0.79081411298790704745	−1.36644447947284586320	−3.93914205747574364125
O	−0.41833144671505850809	5.71108343770925497296	1.91976761331215395323
O	−0.44481810336176264009	−4.73308708727410909489	1.92074931193512865590
H	−0.56833252263759492795	5.15174042288081324159	1.11051546224822028641
H	−0.80874389664521606491	−3.83978107463909701380	1.88377750177654457175
H	0.26815376045866867738	−4.61754702109090064965	1.24678670037201455578
H	−0.05240865300424338291	5.02627938553960618151	2.49144898376025025755

25

converged

Re	−0.09510634277330226594	0.41291246031827721907	1.69213453154564441583
O	−1.46872489821199203597	1.62722617411172554291	2.87276491546297751967
O	0.05783983995618011131	−0.70365678905673145760	3.22624207976447685553
O	−0.24159747955419830223	1.53559728011702834038	0.13042296519803336929
O	1.28126949146913471367	−0.79679885633864167005	0.47602509154876276876
O	−0.16288084876555919411	1.12726132565029546484	5.00832656918426000203
O	−0.02531260231293105956	−0.29750644111292501304	−1.66078431671813597781
P	−0.31786035047327404479	1.34321455253707422450	−1.43769999776276091730
P	0.11685778234519837715	−1.32149192312225993717	−0.40226096716559456290
P	−0.31154422485095062401	2.16828133030033809447	3.76648438469681767771
P	0.03759138940143375018	−0.52722455356015907046	4.81944287837429818921
O	0.93372541007423759396	1.94401022103345888503	2.85435237472526459612
O	−0.48382742329032507600	3.51800151352403034366	4.37810108126835384468
O	−1.16740042631897478209	−1.18511343664960078215	5.41897101346922305254
O	1.36580724563579125608	−0.87506237953215926240	5.41842985474979688121
O	0.29967740169459911037	−2.67233151224551868097	−1.03552011423542422719
O	−1.13028007732650803696	−1.12529397092655503165	0.49235504041136135545
O	−1.68137196182483905638	1.62667629207097497179	−1.98025265984155107724
O	0.82953545648007720459	2.03863106155262396868	−2.13387134378838272397
O	1.03616769854017509722	0.58251441329080488885	−4.35604716755223098090
O	1.51623337694091908645	−2.78329482852395893033	−3.39711423662412137503
H	1.04202551513633201452	1.20351113163557066343	−3.57482558524818649914
H	1.05838459798461048678	−2.79926536006129467182	−2.50958545557138190674
H	1.64726403368596630017	−1.83689449935836801586	−3.51265202445845226009
H	0.46972739738053254088	−0.10280321093624342210	−3.98663890715681201726

25

converged

Re	0.47983733092094704720	−0.29670078320827325546	−0.18824547961414628272

O −0.9451659588374963227 9 0.7580825571768167181 5 1.1120146169613724929 1
O 1.6243315246205780599 8 −0.3345165999402039402 9 1.3444733649362889949 0
O −0.6610697086418781953 7 −0.2443721554148827190 8 −1.7153548418983535217 6
O 1.8844033177690404645 7 −1.3987263741040807385 5 −1.4442953388485157972 8
O 0.9426509637128046570 4 1.9133160863237295146 2 2.3804049881410702660 4
O −0.0298122007773094221 9 −2.5222941642766754100 1 −2.7076360873947855623 1
P −0.9517126232702477484 1 −1.1481154191239488771 0 −3.0035171694570266254 2
P 0.9816623517655540531 7 −2.6668287726089237033 0 −1.4473045328668698861 2
P −0.0587202909491782443 2 2.0368459744672011702 0 1.1128186280767651261 7
P 2.0122263933042274786 1 0.6169933453387098643 0 2.5636663303640396804 9
O 0.7897380990067809358 7 1.8803509572922454395 9 −0.1726378024605463856 7
O −0.7732207280657148462 7 3.3457675254542276199 7 1.3021942120103304763 0
O 1.6770617005877355154 6 −0.0099994239507872756 4 3.8809112588788741149 4
O 3.3922240627778452726 2 1.1761866975257655454 4 2.4077492126046751153 0
O 1.6492873884953636043 3 −3.9921321210618296859 7 −1.5949425312043736813 2
O 0.1095491379534828113 6 −2.4456476812246630281 3 −0.1665427665822440950 2
O −2.3800953317843478451 7 −1.5966506362182222300 6 −3.0420176619142749174 8
O −0.4084436218466925838 7 −0.5115717021139611908 6 −4.2459738498330468203 8
O 0.1311918501184477781 1 4.5625670359510355211 8 3.4947530439416585323 9
O −2.0697921798351006650 3 −1.8717049258396940558 7 1.6714477829035128131 4
H 0.7335936122770663203 6 3.8310164281812029152 3 3.6722161898732395002 3
H −1.7998319573566505802 4 −0.9399488563797612217 2 1.6593222726375127518 1
H −1.4434015750537434463 8 −2.2459650597503655333 8 1.0317219538010051760 3
H −0.2802915664527185768 4 4.2051480516395765008 0 2.6615742059283000742 2
25
converged
Re 0.4790955512479236899 6 0.4275547713671224991 9 1.0635374876129684018 6
O 1.8631385940317153338 0 1.4490702531852901469 0 2.3924577628641725546 3
O 0.3421958083107524695 2 −0.9324990592020487856 2 2.4087582183489546494 6
O 0.6047055561169080162 3 1.7712337674131759524 0 −0.3143168095076074308 7
O −0.9068803555632249935 3 −0.5925966238029962696 0 −0.2903583134261351617 1
O 0.5909758610626879571 4 0.6277529254459754959 3 4.4417900476965153089 3
O 0.3602552126450039304 8 0.2171355663237754252 1 −2.3493543854960750039 1
P 0.6574680997021923323 2 1.8069889377474104286 1 −1.8976402499944273749 6
P 0.2448267077571600336 4 −0.9861224510247228458 6 −1.2598379420617242807 6
P 0.7165041730281630494 3 1.8458691771145632198 9 3.3703189622869311747 9
P 0.3722321472385434759 6 −0.9753589767792281151 1 4.0170156836337591599 3
O 0.8951815741153550387 6 3.0889174828995753507 9 4.1761852066184106391 2
O −0.5411061024102618022 3 1.7533444702427278816 3 2.4549341327803029244 6
O −0.9580606792663362014 7 −1.3931176150361008758 7 4.5617682762828177800 4
O 1.5751311300096049183 8 −1.7216451979654696646 8 4.5043142040416128679 7
O 1.5032893329101815194 1 −0.8958730311431521986 5 −0.3523874302801481839 3
O 0.0595404335909857918 0 −2.2426729133276328553 8 −2.0462924325444036277 6
O −0.4993567230627891095 9 2.5976167268262444487 4 −2.4639328903199229081 6
O 2.0129923371390141539 9 2.1783866235721034421 1 −2.4062748351844369665 3
O −1.8863128971120146637 5 1.1964332247150095689 4 −4.2451364273681839733 9
O −0.0106131409548171906 6 −3.4917207338050548770 5 0.8327468858983313460 0
H −1.4078776086997166761 0 1.8067043644075855279 9 −3.6145923980744969483 7
H 0.0908121165338276886 7 −2.7771302339931112079 8 1.4825569621592733504 7
H 0.0235563123397677098 8 −3.0132967860739285903 5 −0.0077891812138472515 9
H −1.5754934555636075366 2 0.3561253361942132711 3 −3.8916705357681760091 3
25
converged
Re −0.5546011099525987031 5 0.0927138890904279783 2 0.1201728887293095349 2
O 0.7954652830346857772 5 0.9945861737697433424 5 1.6324994394129321850 8
O −0.3092851584642897777 6 −1.5073057103642868437 3 1.1525536115068351072 9
O −0.7717926660287899309 8 1.6864400096751119839 7 −0.9030651303014147757 2
O −1.8715386686366832780 2 −0.8614450207501241907 3 −1.3256076117336872410 8
O −0.2566037247510016272 6 −0.4266127103406597287 4 3.4780776305363434453 0
O −0.9677908954593106294 3 0.5994241007004844634 7 −3.2157777331514436625 1
P −1.1007504477912684048 8 2.0737832884261622190 5 −2.4208822445643121135 1

P	−0.79036574364170752993	−0.81675183952990393355	−2.44320102527802918146
P	−0.35011373765184944373	0.99914902703940766671	2.69621199754053186837
P	−0.07454422899222068466	−1.88490186581482643824	2.68172277273882198045
O	−0.30485965441440410206	2.05771001751894111464	3.74371657709809246839
O	−1.63132005555862802382	0.91178023979414146716	1.81923329254660726129
O	−1.13943348869678962032	−2.80053452345411768576	3.19728521890228201130
O	1.34918649127969425550	−2.32111115623214070425	2.91455664868592823069
O	0.52948810611807795024	−0.69074388973988765095	−1.61239959382466868654
O	−0.83579812720605772824	−1.87595331867614500965	−3.49224320222255002477
O	−2.51968988530949067339	2.53206579483385274543	−2.56490322175296059370
O	−0.04183591346088430529	2.96018771521320989137	−2.99987591533495034923
O	2.91101749690450262165	0.44770325053722431763	−0.52479340141417196719
O	3.01409093068753408673	−0.20632674056237537696	3.31702175216359673726
H	2.19209276448080725430	0.09316315577053843611	−1.07395796839449375604
H	2.41630770279299023073	0.42961314346961648747	2.89897173396976803872
H	2.48120679275050859403	−1.02872520688364565977	3.19638760657958398426
H	2.43766793898950773212	0.64319220297766566130	0.29509587125474262548
25			
converged			
Re	0.18270777469825555395	0.08677444412767962301	0.42127229733948107215
O	−0.27508106383188124511	−1.20582479433694889082	2.14232880183139196006
O	2.01954976344500103380	−0.14347614885604603741	0.88116568330631173112
O	−1.65454971096292702271	0.31813592790873385097	−0.03896928626282070152
O	0.64048885577405334502	1.37957294168081201491	−1.29915403187591338430
O	1.74279856602367977381	−0.30320858953989082707	3.42533455480830806295
O	−1.37730605046356302523	0.47952788129486501045	−2.58339216817452577146
P	−2.53765270648683571864	0.48344091804913247312	−1.36059256027414710033
P	0.19894198447592273404	0.26820077742593817494	−2.29746929953105150091
P	0.16598888918902096612	−0.09404409017952715388	3.13993415717874446713
P	2.90214477928223368863	−0.31504098843208089287	2.20252939633251720863
O	0.01129624424095404533	1.20396775397212030256	2.30442404479312124010
O	−0.49437520211531527092	−0.12101459873774476905	4.48959848701228825263
O	3.55672470351272451694	−1.66094032305228678226	2.24909113288249740137
O	3.77875870967142279966	0.87716629469993800949	2.43008214521217213999
O	0.85998009390362906146	0.29523746076740137179	−3.64667209074316556183
O	0.35293716044243972352	−1.02968094495869277871	−1.46147816007560704676
O	−3.40695221508161605328	−0.71410411759565006751	−1.58843442037678128642
O	−3.20023730449304011358	1.82531913522001754124	−1.40874959938174226792
O	2.29044311385502874501	2.57582511489273269234	−3.52479648615145357837
O	−1.90538671190016950518	−2.41991834780024506202	4.38324652252258051277
H	2.07377689241412399568	2.64593784733659154185	−2.58623802687554604773
H	−1.41977409052774539155	−1.57903100133845319952	4.56525016440464437295
H	−1.70041395501322778472	−2.49063893058028318350	3.44268213537147094172
H	1.80139147038662006395	1.73891637274967214566	−3.71419339428830941330
25			
converged			
Re	0.54276118728280908776	−0.52220252491793228700	−0.26553036049882416947
O	−0.63735222935780622144	−0.37261084698912949609	1.61674404527995463354
O	1.95771371898983992388	−1.17168309704431039542	0.84107876125166447512
O	−0.87181963130125583739	0.13471566834403614354	−1.35924288652160418778
O	1.72046417177596611126	−0.65826000978192289459	−2.08450924998527042931
O	1.47046736415837431267	0.18998589803459986491	2.95384137690743298066
O	−0.34613228721276045885	−1.24999354058071321383	−3.45631877635370177160
P	−1.41323260093985902941	−0.10395489448098144125	−2.84854492596444286434
P	0.88806108055051791172	−1.87155442361504609217	−2.60212511116928757460
P	0.24662519613867850410	0.80296834575750852814	2.10823232152267303618
P	2.52787900272906185251	−0.96390455248132000943	2.31547676300484184253
O	0.81891928799642499381	1.41317306647936069552	0.80264357908361216865
O	−0.42751170620976708392	1.77981806010593257561	3.04536048963573868775
O	2.36250439359504493808	−2.19783647945057136752	3.14684279861845928039
O	3.88474475427769494473	−0.33180005247937016666	2.29532731314593618777

O	1.5710342518902682407 1	−2.8430083690500671522 7	−3.5048760391091926536 0
O	0.2747703210265625140 0	−2.4234768611712471653 2	−1.2785445825691672538 9
O	−2.7688720712674044222 0	−0.7405563437695054096 5	−2.8352916282171514161 8
O	−1.2481687106364136585 8	1.1261514448374705388 3	−3.6868111258499398630 3
O	0.3669552387944535198 9	4.1013601399175119155 4	1.8608963314978486103 3
O	−2.6888735181584166866 7	0.3490354415161126544 5	3.5659367564624768753 8
H	0.0284996011293761064 6	3.3742791934340967685 6	2.4282300600836941306 7
H	−1.9309828812638676343 0	0.9721547614634428180 5	3.5218698050959620360 3
H	−2.4070818563198796979 9	−0.2481083689194190922 1	2.8586051740013260769 0
H	0.6848279286464615811 5	3.5564083342674774890 2	1.1275091096314264760 3
25			
converged			
Re	0.9376821451770731563 6	0.6141063305936989014 1	0.5211950552985338269 7
O	−0.4030128476481908972 3	1.6480184773291703948 0	1.8972681115836256271 5
O	0.9236252777925860746 3	−0.8494881185819778135 5	1.7585419989571700050 1
O	0.9592563317647591070 2	2.0443694204480409304 4	−0.7381310557963627339 0
O	2.2624588074673943616 2	−0.4519186651638529284 0	−0.8557193962485356575 3
O	0.6834580880929183033 2	0.5211427194947330088 2	3.9108405826655565640 2
O	1.0863897949589014224 6	0.6131962611793139172 9	−2.8597923896463623982 8
P	1.1345819041087059631 0	2.2021079837815702440 2	−2.3204475684269043611 6
P	1.1135931275955028851 9	−0.6794674066422470293 2	−1.8793242637196674316 2
P	0.7265048301351315629 4	1.8423627479016113639 4	2.9481618298341123463 0
P	0.5957811256855948256 5	−1.0293085710520766262 7	3.3070559613263661447 0
O	2.0152026219673881968 2	1.7062361644333190469 1	2.0821805876604160268 1
O	0.6403927936817106481 0	3.0160616134006343891 4	3.8652798969250081739 8
O	−0.8260131583228336182 9	−1.5061731241944098513 8	3.4526260383283773336 4
O	1.6440435920112614898 2	−1.8188149269756841430 7	4.0272540541099797195 6
O	1.2056330775840804481 2	−1.8761999790408132593 5	−2.7627872401346960096 2
O	−0.1602293931831715312 3	−0.5590173215158618091 9	−0.9736156427196920315 5
O	−0.0473940985162244754 5	2.8902605962167040587 5	−2.9323971936209365374 3
O	2.4869348194998792678 2	2.7506134502765706706 4	−2.6609541355683674623 8
O	−3.2148035335702256354 3	−0.9574296757271484326 1	−0.8926746357045125535 0
O	−1.6103774478108536794 0	−2.2842035013263655152 1	0.9106860897450501646 2
H	−2.7766308743213996557 1	−1.4862276217670393752 0	−0.1929576669309730485 6
H	−1.3709113132025485537 5	−1.9577518412903747169 2	1.8083343667354547790 2
H	−0.9408187567679464624 6	−1.8482002044053265077 4	0.3549518593068190508 4
H	−2.4591469025736181208 7	−0.4431748073626369244 2	−1.2087752396832254220 3
25			
converged			
Re	−0.6556390971358744268 5	0.1487554095747713467 4	0.5836970666690589615 3
O	−2.0245712044226640280 4	1.0508532129581100722 5	4.3166000860470559175 0
O	0.6551557905874717535 6	−1.0497303150673671368 1	1.4946526031717388693 9
O	0.3910937113063355230 5	1.1693984950846074699 6	−0.7749677733316815420 0
O	−3.4086834477418124222 9	−0.4384394351033674142 0	−2.3658703119763040412 2
O	−0.1044559985513923983 1	−0.4864253666458552838 3	3.8916512496802728726 7
O	−1.2653668886587365882 7	0.7930271907258041874 7	−2.7118008859656810294 3
P	0.2421893290999070358 4	1.3959223785437684384 2	−2.3359884381732336500 2
P	−2.1730664572830029612 8	−0.0273879688785231417 7	−1.6398780601121436539 8
P	−1.2129639852956117795 9	0.4838581033375927154 0	3.2018957788770752159 7
P	1.0538303913610655993 0	−1.2960337869716054282 1	3.0080114321846904701 4
O	−1.9661945806777469947 2	−0.4059589132454682336 4	2.1582502293187721953 4
O	−0.4357150973107558611 4	1.4839984638046805898 2	2.3019224439697434370 1
O	0.9052505083564603705 1	−2.7529806513628489739 2	3.3707427803088867435 1
O	2.3887583612501845920 0	−0.6933669879003585556 6	3.3538153271544124400 8
O	−2.3554498511872292709 8	0.9314408162608218688 7	−0.4168529744713148077 5
O	−1.2750476197931974731 1	−1.1549130118744557194 1	−1.0601343303030494880 1
O	0.1897828405771161963 7	2.8679644044409302594 5	−2.6608891591667918064 0
O	1.2537751733070905402 2	0.6114303403434877548 5	−3.1275206974145977056 9
O	2.0046314533016604819 7	2.7659067781114399231 5	−4.6581195631788165911 8
O	3.3235969071292177368 1	−2.9870124930483172853 7	4.5461639177601762540 3
H	1.9849680038556101191 5	1.8437627320216785076 2	−4.3324831332124373872 7

H 3.32627352217296889947 −2.06123180658089966144 4.23072619376097502908
H 2.42261269136373869060 −3.18324311936206560958 4.20089674657225664589
H 1.34143554541152631998 3.09150554142653977152 −4.00772052389282595897
25
converged
Re 0.12492240683871880247 0.69406115665671752613 0.24704922991524536080
O 0.66961750858071789061 1.88907949659024532174 4.27932210034908777629
O 1.29731016643148855394 −0.74926230489076584007 0.77761306280193165374
O −1.08103501697316306540 2.08185220991931929646 −0.26872349849600635219
O −0.22098534553450538409 −0.37073371444133651487 −3.82189366657505802394
O 1.76324674761624189401 −0.09368592279977644433 3.21794318255705480425
O −1.39100522875682863777 1.56376421877102522728 −2.75776179260876297406
P −1.84993525840665196114 2.65119159089839984134 −1.56932889372046879828
P −0.38726683500775815894 0.30721886081876298435 −2.50254551978417172364
P 0.71414010707570085845 1.14454292188052741963 2.98884245580718843627
P 2.03528943819623542311 −1.29559798667832115626 2.10967853874242727841
O −0.61260585342769191541 0.51095642302414123748 2.47789448323008221209
O 1.25316292850805366399 1.92122758521129188836 1.74855326794167442728
O 1.34504440212467324400 −2.53486772457016362736 2.60889880957182684895
O 3.51184511574022950597 −1.38366047504824685177 1.90334995183663058249
O −0.99539820033674542010 −0.54186787268145231167 −1.34884369041994922611
O 0.91004051075418357364 0.89390127369758465292 −1.85696655551704781217
O −3.33339340257011773616 2.53306204681221425190 −1.41261946436681240513
O −1.31008753663549448731 3.99079061611750640637 −1.96284842343617782490
O 1.74978577870258766858 −2.19763174482786549646 −1.74766103439504894013
O −1.11138268570159715054 −2.02872787739833615461 3.72799386225353845958
H 1.14044893967734428664 −1.57761130748563460635 −2.17021086785103500816
H −0.24478015972705752246 −2.31094852968497832535 3.35799467062799061878
H −1.13775921359140497202 −1.12233308544100784943 3.37271244595648722608
H 1.76698067156985993975 −1.87361987560738096903 −0.83164264485261574222
25
converged
Re 0.51581737679882466718 0.63233613886540507298 −0.50376038201744088862
O 1.42405062432158402430 1.94306207168477973468 0.99458357292161247987
O 0.48975534224578365361 −0.80643099150941854880 0.92329039934215606511
O 2.11091220732799556004 0.31287037695646113811 −1.59054379898069031540
O −0.05349080376604972709 1.99391056581100745149 −2.03494127771688315676
O 0.17910362294701479002 0.81917927524009381823 2.90699901775243585433
O 0.93605125467583527588 0.52855976788908276554 −3.86948818326399690548
P 2.40374661668089562028 0.13762072562522564834 −3.15562192087386206296
P −0.41431909786395049178 0.83674719839536659816 −3.02383007284782179269
P 0.09562887780644145630 2.00319526016473270502 1.79235298145664145864
P 0.36084983988670060739 −0.78019835731816067348 2.49141806766390239147
O −0.19694656964235227048 3.26228794000569655154 2.53203506550520085483
O −0.97453586470997743962 1.52984790769131606680 0.74746461885925741253
O −0.91516938415717408173 −1.46617008327269204848 2.93588716775854186025
O 1.60145043342171256029 −1.27964848001039754877 3.18340751007333633282
O −0.62706850002650704745 −0.36737145535913429129 −2.05997639399216847522
O −1.48273854223257939289 1.11148859114984843721 −4.02701410582735341848
O 3.38783426596406167519 1.16470175740194337166 −3.62704063404492371703
O 2.70542927253252862840 −1.28900645902536781229 −3.49715510691336062266
O −3.07147914935032328998 −0.00168942984361862558 2.06492771817616871388
O 0.36402994103364849643 −3.03650905756839906147 4.80650021938541449629
H −2.53743518995043748632 0.60360688211779334189 1.52374200265902848273
H −0.38357867606649864722 −2.67867641359038533011 4.29097101372720501899
H 1.04590030386681109853 −2.48916005739665635588 4.35174061826664981822
H −2.35759819542988280361 −0.58345367409496862177 2.41085189133187327570
25
converged
Re 0.42295186256795636037 0.06378936572782682723 −0.26559665516966463050
O 1.71644020725175971975 0.04522992414009222722 1.52526653649746202213

O	−1.09064906462390798225	−0.35517145566322194661	1.02091020793610831596
O	0.91857191951830219345	−1.64899417062458941707	−1.05537159235853805761
O	1.67045647184064338653	0.93521121673640950078	−1.75828021109718091175
O	−0.12437404264459703263	0.99931784246298982488	3.00183967113955851858
O	1.00323889846160740902	−0.81017507875314220822	−3.48766720055849832605
P	1.09105937082064419208	−2.19973731775139835776	−2.55057914252188933091
P	0.62581061992703057584	0.64781398879262508395	−2.88343988248617710823
P	1.09170834979580977375	1.38767998653762902883	2.00407152543233157971
P	−1.34326837363828599514	−0.06074513578213978915	2.55136325199058999758
O	1.98123267600383634601	2.32610371392234283050	2.74150036366334637350
O	0.40667046021776720455	1.94550962851977171653	0.70620902134514140780
O	−2.62643487112519080640	0.70521519511537300584	2.73961134016353868859
O	−1.18862895353379927066	−1.28763936086365182732	3.40761428285028600271
O	−0.69263783193809091987	0.46773594746788293985	−2.07491267499847520384
O	0.59902222454969955567	1.58762339814100950086	−4.04158850421672077857
O	2.46827165526962089004	−2.75262680485503752337	−2.75559266835960992026
O	−0.05543970408364426450	−3.08003309845071671091	−2.94175450998530330082
O	−2.20748570057007098555	3.12096023220016594024	1.48048620274564846078
O	1.46921821515455497398	−1.95172441310484590105	3.63506302512361978430
H	−1.34832348792598955001	2.88091544599540627658	1.09783628024921120492
H	1.74634952142967536659	−1.36436907988687683435	2.91151269270728940697
H	0.50037321404526502455	−1.78154273209539781853	3.61425228677907517394
H	−2.42793362516471811219	2.28075275678927580358	1.94404635740508613395
25			
converged			
Re	−0.22284328960301577616	0.26175282185343656804	0.51921494119868460970
O	−1.12997391238026856364	1.97700823196683428407	4.10295324816458339967
O	0.54072465642519840223	−1.24984543241312406892	1.58362748449612977808
O	1.12974040078445003843	0.65794655624930309390	−0.88567709284644824397
O	−2.99444280413123742335	0.49902026388019377068	−2.47035108350903653829
O	0.04939487744634010746	−0.21165550811217503924	3.88833868781826552663
O	−0.54381986607885346441	0.75552843453318285771	−2.84210688272897238349
P	1.09046315571757057583	0.67782255126820312174	−2.47212431211109429086
P	−1.69840740251773736347	0.46302601390584047225	−1.73303794602529182001
P	−0.59812431374748165069	1.03775997086524807145	3.07438566051065009788
P	0.83826188968173431793	−1.46659286599518789984	3.12270944122148819133
O	−1.63767500568533508343	0.40806734994067117572	2.08689658053456250641
O	0.50161452351636492963	1.57245596223795036650	2.11626034386079231453
O	0.17897879748788816934	−2.72818253838647661880	3.62430497904491266681
O	2.30266513805707928597	−1.34925796876301329341	3.45005831750401892322
O	−1.49076806585753707246	1.53896802878484817434	−0.61658294758460219231
O	−1.31170813737139102528	−0.85805085350835152980	−1.01500104979946303985
O	1.65777359771954135859	1.97807695458899535090	−2.99586365920094754856
O	1.65531033339470989851	−0.56617783281033173814	−3.07641730689243297903
O	−0.05311770719200245572	3.24663493398981550797	−4.58062174967004853698
O	2.35271219930774266871	−3.68491753821121337253	4.90141608427430242045
H	−0.80397637702514568314	2.75483291078186454115	−4.23108141371380153117
H	2.68738561955782762070	−2.85970819423303224127	4.49731407509889802299
H	1.44055935864980821925	−3.57940868860486993697	4.54471598791153219565
H	0.66547232957431012856	2.82599644149271567173	−4.02653039386399136390
25			
converged			
Re	0.27943921232766044671	1.04319457408421301103	−0.12306401524555965477
O	0.91097176651046940510	2.22889295204174908704	1.62986976448801379469
O	0.83041160732443475290	−0.58101871062873011731	0.97872795271938173300
O	1.81269398206799570161	1.36003762635106828505	−1.29639205861818718191
O	−0.74612926972477655596	2.44417049558552568200	−1.37583321269204361670
O	0.09126101989350954291	0.42581795295913293931	3.24077947643274688616
O	0.43041553900714235592	1.56178357577477822105	−3.45989982357121261813
P	2.00027122577178495533	1.59723650246080128845	−2.86896969314560390529
P	−0.88384878743236561238	1.39636233829152378583	−2.52363706676894583580

P −0.34723394598966550451 1.74814612729145846259 2.40162996737156841931
P 0.61423852403645606834 −0.96352124776108738491 2.48512632634928776199
O −0.94115975948122343464 2.69539152803556358862 3.38766120997730535436
O −1.28257871113008770081 1.23007385756276743649 1.25516790983571491225
O −0.50271042404846100204 −1.97548807110690427535 2.61005611936599235534
O 1.89386843769218771172 −1.35588821221797517502 3.17989035357294236306
O −0.71223713428413126802 0.04284399690115974629 −1.76295526754781262113
O −2.06334069262995001282 1.52421170025291519146 −3.42588918253111707202
O 2.53320182960667050764 2.97199875697036297595 −3.13671228471598251275
O 2.73431340830923685914 0.45618823695920873718 −3.50378822763020725262
O −1.85413233302690638737 −2.26941430548673528378 0.16073785936264778362
O 0.98037199867364177575 −3.61618399503782050886 4.29497562354355721936
H −1.52103855379689223426 −1.54425946480580345010 −0.38601353511114866457
H 0.19506526704502957781 −3.34436329469481741228 3.78610575267539806177
H 1.53253664791555799241 −2.84718584384001527354 4.01271503782512262148
H −1.37845085890675722595 −2.12792707593278551670 1.00651102362615096908
25
converged
Re −0.74961163396873498055 0.55565127210524911661 0.17352205646283802576
O −0.82080967772484125788 1.65164767910047283195 2.07277330710545948378
O 0.67129582308175417271 −0.59289033048843164941 1.03637611702774656841
O 0.30585131820874350561 1.81547797469939586890 −0.91997180063629269142
O −2.44094547335898326423 1.35087151375019542954 −0.85558448562061539810
O −0.39218733302273534047 −0.49470887006527597407 3.38788638904010097619
O −1.15428693589079345294 1.61499080412155859854 −3.02933247268001082375
P 0.35352559483925227735 2.10290653016178596957 −2.48967267598520036742
P −2.08204700859853275574 0.58894669112422659563 −2.17236511150524513880
P −1.54000682808018374281 0.44839449986028850903 2.73677124409095196000
P 0.97824548158450830293 −0.95180046618473146403 2.54929115296691399806
O −2.51674105335022790797 0.74592689045107829493 3.82420549223920591686
O −2.08537201726740795138 −0.35384564842839938414 1.50393344096730552373
O 1.07206909071677136680 −2.44622411924953242490 2.73216249900502017312
O 2.13276951050193019910 −0.16960679479448625973 3.11208270106384787823
O −1.14453633266468735030 −0.54348025047101644613 −1.66716190175703560250
O −3.21933794399953043808 0.20473925544617657679 −3.05789097076967930633
O 0.42786688190084898320 3.56999538255647586027 −2.77725205843389222338
O 1.38529625224422803242 1.23317877391380092966 −3.15131269041718509172
O 2.47028465909908589992 −0.80214816770402519452 −1.58030638437834469734
O 3.29545674766786422794 −2.38398458841155802546 4.25827571191724896238
H 1.93633315140078821059 −0.80361939182417396754 −0.77419201651125413299
H 3.17486952083005746772 −1.44384142435305951047 4.01798981136389254942
H 2.50691196265792859421 −2.69244736175573118331 3.75523107054383853409
H 2.04130626009054694237 −0.10302982709186811472 −2.11865843140692344804
25
converged
Re 0.17860444101942882233 −0.06402370462120147987 0.06675043455418960825
O 0.66922656018884563167 1.96174825496246696410 −0.55381735936272380449
O 0.61400649817513919260 0.49761032932527182737 1.89965555168386224238
O 1.80622234385116620103 −1.04165862959834987223 −0.59254773149180839376
O −0.67081615427008522978 −2.07595019532803659601 0.42581825001666523400
O −0.17196587041355915204 2.94523497338379813826 1.64723606235756681038
O 0.51002602929408491494 −3.00180166850860308259 −1.64532037577038847687
P 2.04723239022979797141 −2.49523457521822944472 −1.18489438919877065892
P −0.83425253345895478141 −2.27508725640232922771 −1.11143184870621003490
P −0.59980705679767121197 2.50785151798093775000 0.14862673137163867620
P 0.63567184097125939424 1.88578942317179754085 2.68913950679201096960
O −1.22715430909551304062 3.73218816509657003166 −0.45983412701756937180
O −1.51277878256813336222 1.25893326623578460932 0.28206616073064638694
O −0.21073065571180976674 1.81749817757497811321 3.92228471258011346379
O 2.02822629071712245974 2.40986664728114963197 2.84868179439769964034
O −1.98639981696688305846 −3.09772851783822300575 −1.57604059805665319693

O	−0.76205279323869001828	−0.80554262763512018974	−1.65134952775990839768
O	2.85583315551993655035	−2.43769151978748332965	−2.44329709181744414437
O	2.53183395137707645262	−3.46323357469490522575	−0.13716759600043265666
O	−1.27101118522258071941	5.70998257684517529498	1.32658132898485070506
O	0.66690854927155296394	−4.34276435663601212411	1.64316373956265260325
H	−0.83886323807213480919	5.15355162352354145838	1.98467694330588950713
H	0.05067588255322391655	−3.62736633681646081229	1.41326859233700408147
H	1.40670891105615569749	−4.09740925118872922894	1.03923332958783243285
H	−1.30914443680289926952	5.04633726419353845216	0.58531751648729624105
25			
converged			
Re	−0.41496207610183766246	−0.00998740398769690230	0.66394739239387545737
O	−0.73805741349164266651	2.48758361261438087908	3.86566960408495718937
O	−0.30043742138159157395	−1.49712932858400860070	1.98959811097023098192
O	1.01814442006855365719	−0.37912719091980962016	−0.65598874805162266100
O	−2.88670360334232345423	0.60362349731155273691	−2.52293587312873146544
O	−0.44543986164685278606	0.01512632810303778108	4.06376467389330731095
O	−0.51175541633396370855	−0.15443968756083173433	−2.73180144174431882576
P	1.06155690033654437343	−0.45380851403000543876	−2.25103125524682523562
P	−1.69020077688884962086	0.27171330510509822398	−1.69803731555864079183
P	−0.54731854534293356007	1.26606392787380683806	3.03179592719790225175
P	0.01109841732581584584	−1.51394942885112993025	3.54917964270370189794
O	−1.71311461814311094543	0.90227220975181099405	2.04739507122261876759
O	0.70098109466168845305	1.20112246247509246722	2.11076411794180662795
O	−0.88866441842143462093	−2.46551755370908498577	4.27625583039965562193
O	1.48607074199102262568	−1.66596033541135013500	3.80366432892357009266
O	−1.09628385548901885471	1.41488355444714231091	−0.81104909300783956105
O	−1.83111671093351802142	−0.91411456334074436825	−0.70271456628092909824
O	1.88456123223786731202	0.68313247062406090571	−2.79822624324311952648
O	1.41559203937957445163	−1.82279220173592348964	−2.73450204242509897767
O	0.65626770407171675092	3.09623321443773180661	−2.42405861301213843007
O	3.15721375783659885528	−1.42239995817100672859	1.61628047004782815499
H	−0.01799643376282342166	2.73097167368686344346	−1.82707554036647579210
H	2.54733600094057521801	−1.47212462387580456102	2.38662386324214903510
H	2.58233761362236480252	−1.07239715476398989047	0.92330400266233025874
H	1.16709122453813929354	2.27212169910391104111	−2.60402229934195439398
25			
converged			
Re	0.51149923042736011869	1.07225640248356168804	0.97730092512776001179
O	−0.87841221146901760086	1.77160173810290433316	2.50306238196708763510
O	0.82005949548055301346	−0.43182170682242831949	2.10946882050695094790
O	0.19161550242875977079	2.56447521227872776706	−0.17781681154924328103
O	1.91706015302563614888	0.37250684987058751974	−0.57747520288371312347
O	0.56782433487418815243	0.78114473189818733889	4.35717068256634476597
O	0.46373788332613230523	1.36977800966010709871	−2.43025497549822899757
P	0.15458242192011864558	2.88057302881440691067	−1.74059835941289531291
P	0.75694426577149442892	0.05085597928686636654	−1.54846124812644547752
P	0.28649704754183313860	2.12003198150422544899	3.47980781365220259715
P	0.89772971575198090566	−0.72116195943194294493	3.68314020611134829153
O	1.49172748336728178131	2.26847855933447606347	2.50090712514380042464
O	0.05758868830641486469	3.21900923922402082411	4.46275471818408586699
O	−0.20250576792111860125	−1.64064356117931109402	4.11614797102307949928
O	2.29001264116514580138	−1.08725718122561865187	4.09705521997117561028
O	0.98639033260711794782	−1.05563990620766934292	−2.54483079755003682720
O	−0.46652905042115799805	−0.15837118699283964096	−0.60228600091174611109
O	−1.21882766801314579297	3.27543012027412272502	−2.18841996929817605988
O	1.28487291722108887271	3.77527086669007116981	−2.14694970520869654607
O	−0.79957286823369422279	−4.52398414011569904858	−0.66322054648102746199
O	−1.28985779911992359814	−2.32429587716962204880	−2.09945789177640662615
H	−0.99818950383990223685	−3.75931925998367155728	−1.26790441268462927837
H	−1.40964828283397203279	−1.66000434598441715117	−1.39565119993117847663

H -0.41470367853638134825 -1.98240820244905768810 -2.42134292120357974554
H -0.39369528709623358154 -4.07540539714219995915 0.08465418783017303328
25
converged
Re 0.34600974317423954796 0.18394323550609570361 0.40501002239334915611
O 1.64964376955282610027 -0.11211761010065221522 2.16267448565110864678
O -1.04788365452393650301 -0.80355743656395128838 1.46661186112140828897
O 1.08612594244717297620 -1.14967399281522175691 -0.83003782999133779441
O 1.43060230698078294509 1.60801218478205809603 -0.80370385819015432993
O -0.33786866495494649598 0.15244641047531951039 3.74950115696180574787
O 0.94933573759460887498 0.31557074973507870386 -2.94735281119142022987
P 1.30390623818448236904 -1.24435989632945487315 -2.41119271566502924031
P 0.40527316550229453007 1.46874280171283588814 -1.96118740401674540585
P 0.82153197633199082084 0.94838163825174937038 2.94890681453330838480
P -1.26612156952438370539 -1.04788498987930345230 3.02025056297774696645
O 1.54898147662794749557 1.78047352844915574011 3.94802349956748699356
O 0.08591280211464717709 1.71986931414985355637 1.79928965467677515200
O -2.68240091364662580631 -0.76851887169676635914 3.41563571355358330806
O -0.70753984868951469434 -2.37143669612796204049 3.47363925346274537986
O -0.86782402005659775579 0.91527281713679631281 -1.25726294684985662187
O 0.19429270675709084393 2.68216176438754816402 -2.82312780383494299485
O 2.74681270942981203831 -1.47288512590630338295 -2.74002969192808087939
O 0.30830881042517904733 -2.16308601361672847574 -3.04782308253818712274
O -2.47882871147588401683 3.00354584528372781094 -2.70116581714921899504
O 1.99788176884483692497 -2.52737792455655441159 3.72753341506866675559
H -1.50128897029026142640 2.99124480087883304336 -2.84634810361720003158
H 2.13273677047471510448 -1.73246500174104967051 3.18505026017747816525
H 1.01334414454082932799 -2.56532436120433660065 3.68596869686182238013
H -2.52474372009074077283 2.29012281921524873951 -2.05206333834242204972
25
converged
Re 0.59774968713745013638 0.53858703696200449507 -0.20013104243502910506
O 1.00671663932670973907 2.49250727397704974209 0.66516310608121054226
O 1.28247508257117992514 -0.26009821092755414096 1.48757553925976004905
O 2.07781868293977556306 0.51982518353730156058 -1.51314644452851632117
O -0.64338990592397893309 0.92942501633872165456 -1.87287371055706608480
O 0.52654866716987314135 1.66682354431730206379 3.02283781505346205165
O 0.71660199643248390089 -0.61036923859001868120 -3.38673829442173790483
P 2.24967167921970823130 -0.03840728577103272312 -2.99805670622634057310
P -0.54753293147555293086 -0.54874301248576518031 -2.37866718637537477576
P -0.14879402231586053018 2.32984053690775239076 1.69389739506811509706
P 1.09875983135669308410 0.10035645796801050122 3.02077772490603724265
O -0.82746764161256347592 3.57970126855335779581 2.13725335691653794967
O -1.03738107250027722372 1.21175421955489914794 1.06175478258899835815
O 0.01151454943158308764 -0.77402663370564062095 3.60290551127780034335
O 2.39672197367885964070 0.11774869371404952934 3.76258095326521413426
O -0.12855457715282164743 -1.31499788263180406034 -1.08433315871539126540
O -1.71685463449264741342 -1.07908567520472642443 -3.13499591520788545296
O 2.54139856289727905647 1.07963935419315837372 -3.95413228254466009304
O 3.18190654928990213079 -1.21130236487961950864 -3.03911901301299103295
O -2.55521446383963457549 0.13550178546358529474 3.28195738321390528824
O -0.63423428042684837358 -2.75209412953803633783 1.53431400161834230467
H -2.29762844866656479326 0.61773128920807296449 2.47919466858055059788
H -0.38541753836634262687 -2.06921296192018910887 2.18369581349701036288
H -0.47747100057105618109 -2.30402513009417786449 0.68867879693459377322
H -1.68174339366855951639 -0.25597915081246480629 3.50640691533146409142
25
converged
Re 0.32992939215353955795 0.84153078614818177527 -0.46834833419527766019
O 0.50856386953673848872 2.33090163088050639217 1.12348882802618321719
O 1.40350477154982100636 -0.39278360124191974423 0.70864707645785807166

```
O    1.58531610403796774378   1.63914643782323898868   −1.74919727056820928368
O    −1.19446923959189188480  1.64909282103702103939   −1.70709847293814087621
O    0.53017916573529144664   0.55131031266792784873   2.94736591322896357426
O    0.13941531240072890796   1.22369376201849422436   −3.83663391681669141420
P    1.65404491252824215053   1.75767733326389241633   −3.34289462059047659537
P    −0.95114883675916983563  0.57936235600587071559   −2.82395321893358408616
P    −0.42791470746540882786  1.51110011595295112841   2.04875570481472468742
P    1.63195889048571207702   −0.50530186551226519143  2.26840780912723749552
O    −1.28114239912208738303  2.27251776977992792084   3.00318617667663634307
O    −1.17096116795117821674  0.55304506831375888076   1.05131509419441226960
O    1.19438924866603679575   −1.88042806572362830586  2.74239962238813506090
O    3.00135252545670594415   −0.09170569047513781047  2.69619361147061020745
O    −0.22980212196202268182  −0.55720174876564387834  −2.04237465599415957129
O    −2.12618172162248519896  0.19901319240624071316   −3.66018102957589297475
O    1.74683973681528392774   3.19375263686002774932   −3.76255125725476302634
O    2.67241826047852315540   0.82320512379512456036   −3.92047895522215794628
O    −1.51338330287925759698  −2.02866072907134320502  2.19733609945780195005
O    −0.22475857470806989702  −3.00999020313080922762  4.70591467062930135512
H    −1.60651188163179581281  −1.15955176091114520176  1.76760831149312136468
H    0.46997421205338940631   −2.58804745187991080968  4.13924180841946487419
H    −1.00046921319880222079  −2.75679366641490553036  4.18825921023440450597
H    −0.53494323398347842780  −2.05378456910866846741  2.29239180503850903392
25
converged
Re   −0.45472850500006556373  0.29548470210739830044   −0.67322604667506902310
O    −1.32921835154682921321  0.16940908576496938043   1.35044666433201188127
O    1.07423559994686823593   1.27505745621551058733   0.20142666601203434995
O    −1.61626810804537357491  1.67246696146551809825   −1.44377059470646873507
O    −1.63085842186294716072  −1.08208120721456557689  −1.80562775396005914530
O    0.98295150573982537079   −0.01446076476892317608  2.43679275918680771085
O    −1.88130943223585100910  0.55018779465018252672   −3.74598327446732204393
P    −2.22406554487054641811  1.95984544193076470897   −2.89823702197638066735
P    −0.96170693568439535870  −0.64567281216137539790  −3.14929971864018343197
P    −0.23584873459273611451  −0.84217364669898953089  1.75856437688432176714
P    1.61785273305343690886   1.35671404597811773307   1.68112542670245246335
O    −0.61654462377505803161  −1.88014000373842526947  2.77713521435191834641
O    0.30163247654606806236   −1.38525037946814721934  0.40106819188453207703
O    3.11238733095621267211   1.12692955312416076552   1.70744201459076805349
O    1.13521600034977798721   2.55781374436599850952   2.42583404172936667464
O    0.36624738840789766714   0.00939218205244636561   −2.66434632035237317638
O    −0.86177951840480992995  −1.67650021936162429981  −4.22307553308694227923
O    −3.71810775211640853755  2.05634628280729581107   −2.83746953591983031018
O    −1.50405042673147737453  3.08171678383454006322   −3.58032737429479253066
O    4.00970551559571486422   −0.91008302013803821229  3.11710782089927862515
O    0.73261579623126782401   −1.55169491698021966286  5.04643099534460759514
H    3.16384887297675954088   −1.36646517736570127788  3.15980289348103404734
H    0.17753594057062993539   −1.74731956703528390484  4.24289051466610533936
H    1.22695779153378592419   −0.79653997764391637482  4.70973565853499565037
H    3.73949940398058933155   −0.11188234700391028187  2.57235994504719522880
25
Coordinates from ORCA−job Tc_Deprot_s E −81.223976711550
Tc      0.18426738598732     0.08404687778732     0.42267095286179
O       1.50899431912157     1.24003564552632     1.68305312420891
O       0.37250018814155    −1.28228293658794     1.68354648383013
O      −0.00398530925935     1.45037563516330    −0.83820420565646
O      −1.14044819304001    −1.07196064331216    −0.83769801463105
O       0.56822850774583     0.15215158546236     3.78543742088259
O      −0.19970984593203     0.01593795163850    −2.94009531377295
P      −0.18846195817282     1.58488585779493    −2.40749919810033
P      −0.04204490342802    −1.25503572515163    −1.91917843969370
P       0.41058212458617     1.42312789698533     2.76452047749758
```

P	0.55695331668187	-1.41679587458142	3.25284490655882
O	0.47076238576604	2.63943823690433	3.62545973414880
O	-0.89047514348710	1.16374401590298	1.95855816081040
O	-0.62709342105543	-2.09362572688725	3.89254146828561
O	1.89512562296253	-2.01345082706309	3.60295674024124
O	1.25901600368417	-0.99563677663392	-1.11322674776313
O	-0.10221535212973	-2.47134863711073	-2.78011435274209
O	-1.52665052635534	2.18151107020229	-2.75759472020311
O	0.99556344043667	2.26174260405141	-3.04720887808054
O	-0.50727500831869	3.99208304458189	-4.51717087427890
O	0.87569918780279	-3.82400529199787	5.36251307036984
H	0.27493291680969	3.52811133866735	-4.14924373984332
H	0.09350017823403	-3.36002002940455	4.99458564668317
H	1.52360891240987	-3.31455435660777	4.83038302878011
H	-1.15517484404456	3.48262508055491	-3.98503673140895

25

Coordinates from ORCA-job Tc_Deprot_s_job10 E -81.220710721800

Tc	0.01639969881085	0.31080580816846	1.43386660026281
O	1.54906985690204	1.76785452259464	4.94401210761971
O	-1.02293342311937	-0.79328883092147	2.50308520148652
O	1.06983770126229	1.40265244258284	0.33791888334734
O	-1.57241818504531	-1.06240330134441	-2.11061727752651
O	-0.18794911180553	-0.01255252970990	4.78623582363955
O	0.16800453278522	0.70418324914654	-1.94927474954223
P	1.33990403396176	1.64786675634330	-1.20650946417585
P	-0.75718855994465	-0.32338841628423	-1.08751296489708
P	0.76634339057924	0.99408136048729	3.93593513717255
P	-1.28645365403072	-1.04957573948842	4.05973617413621
O	-0.17296687937897	1.78743390358489	2.98299391046418
O	1.55207763716730	0.10662803484195	2.92937290338362
O	-2.65643379134181	-0.59335189541771	4.45809253558589
O	-0.89567548283453	-2.44038762209286	4.45495395407836
O	-1.53182990153882	0.53529575432478	-0.06429884284278
O	0.19828778107679	-1.16133399909117	-0.20334967628967
O	1.06769178912985	3.06080074606080	-1.61156236891495
O	2.66969105929536	1.07731085813745	-1.62051826764395
O	-1.19489428476981	0.02880571785729	-4.51969429493813
O	2.73577104296861	-1.66202111257911	-1.48146507123577
H	-0.52718082061040	0.62407166767391	-4.16041326765248
H	2.80625716869697	-0.68447680697596	-1.56698268603563
H	1.89277642039171	-1.71159039721112	-0.99812993920572
H	-1.41998803346105	-0.45232015480289	-3.67907437187506

25

Coordinates from ORCA-job Tc_Deprot_s_job11 E -81.220591007330

Tc	0.81626452877382	0.21050448678691	-0.14747129257732
O	2.11997806698586	1.70858842342343	0.67848017329286
O	0.95953313183780	-0.65323822810879	1.50273737371134
O	0.63723132078963	1.05237916656090	-1.78975813757675
O	-0.46787967191500	-1.35118092453195	-0.90923809343263
O	1.15989961116682	1.39237372041545	3.01161455460646
O	0.46576798295609	-1.02855690503325	-3.25467027986105
P	0.57915976899667	0.64087882287134	-3.33382228968599
P	0.64809123045751	-1.86339841793816	-1.87129185423757
P	1.02222828868040	2.25810329838312	1.62867847410711
P	0.89323563774279	-0.24848937479866	3.03577402065211
O	1.11513313175093	3.69144178768647	2.02731752673918
O	-0.28483160148782	1.76751733471209	0.94741976269224
O	-0.50799416241728	-0.47905531461225	3.55117758223924
O	2.00147384423855	-0.84916839868114	3.83907455663499
O	1.92807913111224	-1.31373692793131	-1.18536523733875
O	0.62621763422292	-3.30483374977540	-2.25279929532877

O	−0.68347602139851	1.12303256377188	−3.97970127587999
O	1.86326495021604	0.97634219256414	−4.02874242446221
O	−2.28097877760844	1.55671635401053	2.99449412417568
O	−1.80338361631810	−2.21002415315575	1.59517657955510
H	−1.74897588778923	1.82288281225597	2.22400754964529
H	−1.36991646031621	−1.97892703837344	0.75661518124778
H	−1.35116488777218	−1.62238595295605	2.22761752273284
H	−1.73075717717472	0.80333445421413	3.29947518674971

25

Coordinates from ORCA–job Tc_Deprot_s_job12 E −81.220328030850

Tc	−0.54613847839949	0.25036969665301	0.04849492568194
O	−1.90737306120080	1.63111087096661	1.00943827201172
O	−0.64634570278561	−0.75164992840625	1.62631238550872
O	−0.44828479303173	1.24878852106217	−1.51110955673151
O	0.81757082049537	−1.13695147149826	−0.88427992351688
O	−0.90494566926547	1.15892323998792	3.30175982593231
O	−0.18439809929867	−0.66676966676259	−3.17403947517338
P	−0.39165118357337	0.99413200474857	−3.08845438070406
P	−0.28407214036056	−1.63432311938443	−1.87183504936136
P	−0.82012362077775	2.13052898240129	1.99324321506247
P	−0.76917969732365	−0.50241430176275	3.19043729492375
O	0.49805350405860	1.73739126587536	1.25775559608567
O	−0.93351824947160	3.52505141273273	2.50925062153293
O	−2.01706494140149	−1.08884673392362	3.76743820763076
O	0.52051007991695	−0.86978857470487	3.88103190269454
O	−0.17794611024632	−3.03101939705421	−2.38288963773331
O	−1.58034886505639	−1.23485771492880	−1.11732725890765
O	−1.70401934966321	1.31861705875543	−3.73380286124567
O	0.83016959444699	1.61074749046457	−3.69658142988980
O	2.54683678447801	0.34123109024637	2.56615574623258
O	3.31076891870781	−1.42146509369645	0.53643036877329
H	1.96410001467957	0.88835690099193	2.00492345122151
H	3.06760299524430	−0.82476147914826	1.27477101648225
H	2.50207034509063	−1.40137129898232	−0.00571292455270
H	1.86392690046842	−0.16992975991538	3.06538967231812

25

Coordinates from ORCA–job Tc_Deprot_s_job13 E −81.219788765740

Tc	0.11672529062405	−0.23751497696549	−0.33352467760583
O	0.31195221629165	3.69328293868444	0.86799050059641
O	1.20474889467870	−0.57094636823221	1.14451043585652
O	−0.96491601705301	0.08471117340594	−1.80553715629010
O	−0.03970765808378	−4.18266122300480	−1.44462691454304
O	1.40624138877527	1.80710966914261	2.05700128049874
O	−1.20357455700404	−2.32055162498479	−2.62275008255381
P	−1.60167628589681	−0.74141440404344	−3.01746335797593
P	−0.22909952606310	−2.70484581885267	−1.37937430398707
P	0.47523097315926	2.20034173914573	0.77632778881939
P	1.73459748055693	0.20421469055743	2.42390588926049
O	−0.79944312934181	1.33576233852232	0.86520348305583
O	1.18881953793970	1.65266458265997	−0.48212208717087
O	0.90296044762479	−0.16344106362248	3.62002532701746
O	3.22000286661867	0.11725443160046	2.57887106865098
O	−0.90731407339640	−2.11710325012470	−0.10322038967154
O	1.03001710715685	−1.80463410309465	−1.50601633084936
O	−3.09742019551622	−0.66064898683803	−3.00404433221601
O	−0.91956215823636	−0.41489583417149	−4.31053524834988
O	1.75335166214868	4.64532933229385	2.89986637707276
O	−0.93843917325117	−2.20655292524084	2.94808827031111
H	2.03359595156798	3.75425447848509	3.13863834839596
H	−0.93860366411999	−2.19524736688931	1.97834530941094
H	−0.31691261216050	−1.47957779917826	3.15889560777040

H 1.18462520225236 4.40621037604664 2.11834518818915
25
Coordinates from ORCA-job Tc_Deprot_s_job14 E -81.219597930060
Tc -0.27194864658332 0.82877497029596 0.44885814641803
O 0.97620923689919 1.86427759357246 1.87544550157840
O 0.29739009019533 -0.74787728786904 1.28150016320083
O -0.83577967318825 2.39356368472549 -0.37521200910472
O -1.52909898656038 -0.20854487743473 -0.99238322216724
O 0.48744794733039 0.12865611615332 3.67281011232388
O -1.03915955184998 1.55199140543450 -2.77300008493584
P -1.26096253625989 2.91856003559469 -1.82247822931074
P -0.56249229752167 0.13479526289285 -2.14983580469295
P -0.00256324433992 1.55474508490048 3.04028732238499
P 0.67010761208439 -1.23314746974825 2.74354796616633
O -0.05232662140100 2.50961305057264 4.18531478849295
O -1.32400115322524 1.25519837112662 2.28289301031596
O -0.31350637546028 -2.25233354229168 3.25675596581824
O 2.11715602634505 -1.63937452216247 2.84161827267571
O 0.78205496499859 0.38785468962800 -1.41471314970566
O -0.51457850372550 -0.81415181157508 -3.31475692338388
O -2.71935123674844 3.25439671351890 -1.87244280012162
O -0.30725822302011 3.95654245306481 -2.32711729275617
O 2.15757611492139 -1.16545867936981 -3.56009761933900
O 1.59542002115294 -3.96255307371484 4.16824962187261
H 1.17397011747675 -1.10962680617434 -3.61112674721371
H 0.71528707242581 -3.57608550784703 3.97037243255875
H 2.09597626019178 -3.23716420288979 3.73807249999597
H 2.27063159746825 -0.69155166097765 -2.72576192208666
25
Coordinates from ORCA-job Tc_Deprot_s_job15 E -81.219208569940
Tc -0.41607070805566 0.88912084662666 -0.55506525236291
O 0.62543246777938 2.56498364486993 0.35066225485138
O 0.45231735592175 -0.17353218255513 0.73742303402602
O -1.27302766802992 1.93830137389056 -1.81988165116484
O -1.43988784165428 -0.79588351854328 -1.43519331404888
O 0.49452756488875 1.50558948146858 2.66431095716426
O -1.31087582505148 0.26642106598164 -3.74804866592945
P -1.76661868524736 1.82395101580559 -3.33883472757119
P -0.55309667589783 -0.73667516227317 -2.71119949011660
P -0.27284766036279 2.51424936806644 1.61237609399247
P 0.91260054076330 -0.02426349700389 2.23826145625485
O -0.49083715744722 3.77990240364789 2.37032031791182
O -1.51733960243568 1.73791089076466 1.11378877488824
O 0.12430007938689 -0.95131122689354 3.14168038574965
O 2.40667181740141 -0.14406105398524 2.37792659150319
O 0.69595487977854 0.03218271297485 -2.19485906854137
O -0.32801757239546 -2.00656459690355 -3.46212159568743
O -3.26071549986340 1.88344244685547 -3.42659454080692
O -0.99998829229268 2.75566268000451 -4.22638915418622
O 3.51347686660705 -1.95071630717532 4.02128165859532
O 1.07763018666974 -3.30873535979741 3.45685528373706
H 3.19077973609072 -1.20901867000760 3.44227040443280
H 0.68281022860512 -2.36260869323078 3.40876776083737
H 1.33760087674267 -3.41401886255247 2.53854794479626
H 2.72142057324547 -2.51322877885876 4.01051451949249
25
Coordinates from ORCA-job Tc_Deprot_s_job16 E -81.219400175280
Tc 0.00442564732666 0.42578352137543 0.55358902410971
O 0.77519628481512 1.82532041703720 1.98914847657689
O 1.38215620976671 -0.71727542984192 1.07984229692927
O -1.37972408740121 1.55135019203375 0.03579819102001

O	−0.77226251061208	−0.96503488756038	−0.92533674341275
O	1.54534270476543	−0.03415162394048	3.53559003424984
O	−1.48842107953495	0.95718447668184	−2.44210616408592
P	−2.18602683447564	1.95318852188538	−1.28159386936257
P	−0.28676854025990	−0.06720515584412	−2.08703754569860
P	0.30491193495125	0.96766374992923	3.19469476641072
P	2.13779369574631	−1.12634402467311	2.42086097219411
O	−0.01769254486277	1.67751455953837	4.46534430582858
O	−0.80367467033882	0.08365393789451	2.55154487215227
O	1.70605403857660	−2.49421135548497	2.88042041818805
O	3.61180303601159	−0.89458716012280	2.32351081634796
O	0.84595417880881	0.76530255006516	−1.42667362481121
O	0.05744141224488	−0.74220969531325	−3.38583027517341
O	−3.62670036334972	1.55704769389513	−1.18331901328218
O	−1.89618843802051	3.36500074953588	−1.68761203640536
O	2.50724801991200	0.29491536392274	−3.86296268703637
O	−0.88857645925172	−2.54341764537680	3.76254962928253
H	1.62045041231070	−0.13779118869853	−3.83536805336112
H	−1.08405732643805	−1.66371461672409	3.39557932435034
H	0.04992007227039	−2.62100061458889	3.47504174100476
H	2.48759520806126	0.68411769613490	−2.97887485703108

25

Coordinates from ORCA–job Tc_Deprot_s_job17 E −81.218893360060

Tc	0.49603146667656	0.28812984245052	0.47421004025316
O	−0.05764289100269	1.92354704985215	−0.82732994826680
O	−0.92438638811762	0.83963295915043	1.55679596644688
O	1.90427050271860	−0.26967025826375	−0.60440995781201
O	1.04268342505540	−1.35308979897661	1.78952779066898
O	−0.76920350451803	3.35755538450919	1.15215690810522
O	1.76404746425170	−2.78879166136317	−0.19394004729032
P	2.63316865481488	−1.61999734140832	−1.03921965713659
P	0.51762106209044	−2.39393974099774	0.77340550378984
P	0.48537795651649	2.97960140972427	0.17369780184808
P	−1.59641383314536	2.19934203877888	2.02389932485722
O	1.46759481804846	2.14147994383624	1.03338522832555
O	0.96222409804175	4.28120751524267	−0.37909246368485
O	−3.02935315038524	2.28667390630912	1.55329413848702
O	−1.36856693517784	2.46349317208106	3.47721482769119
O	0.03112062410451	−3.70612144591990	1.32662954738073
O	−0.47963259519008	−1.57632533504858	−0.07976081706920
O	2.43346978777627	−1.90843869894228	−2.49450742420456
O	4.03694806453509	−1.67559115379224	−0.52180646096411
O	−3.46428001400338	4.40004399970213	−0.00627718019623
O	1.51145680669339	−5.71041991824908	0.37314554405150
H	−2.53209920844130	4.48225621708340	−0.23461518345089
H	2.04638497761902	−5.09257732570900	−0.13845794296671
H	0.89600580534600	−5.04792755298501	0.78995197513782
H	−3.40062699857645	3.60102678765341	0.58890250085886

25

Coordinates from ORCA–job Tc_Deprot_s_job18 E −81.218817278010

Tc	0.34718345169309	0.10909713016595	0.72394139634125
O	1.07998948606769	1.83286175312738	1.79234797194763
O	1.70650113312283	−0.84466305973139	1.55508020870845
O	−1.00580915364803	1.07686566810214	−0.12612212306411
O	−0.43955078957854	−1.62398081296989	−0.28831366701379
O	1.74268991064031	0.39390633101474	3.78841826218493
O	−1.15328903529434	−0.25462274081597	−2.29815837412231
P	−1.71091276151743	1.13259046686688	−1.55129884113875
P	0.06679584921337	−1.13623600385539	−1.67772416965394
P	0.55083862887254	1.29156466615476	3.14752115519405
P	2.49010089507028	−0.85143714333811	2.94825123139156

```
O   0.16699436684165   2.28253070369054   4.19470907384215
O  -0.54268738260655   0.28178659029808   2.67971237403300
O   2.22517927501551  -2.11103358751822   3.71332545098253
O   3.92354086702567  -0.45792470608645   2.76609581092985
O   1.18497133173670  -0.13036891488937  -1.28536612165341
O   0.38975726002310  -2.17980206871494  -2.69109585501418
O  -3.19626913754103   1.00086872426715  -1.44669702917277
O  -1.20126480199039   2.29945309783347  -2.35582802505324
O   1.38924885340489   2.02976646883432  -3.18550839579240
O  -2.09443355462831  -2.09998456717359   2.04894554746378
H   1.55298437287019   1.28517544473904  -2.58070424269764
H  -1.68092133623397  -2.07857495381559   1.17143038488372
H  -1.63600020889066  -1.35617126408937   2.47078517578475
H   0.45056249193730   2.20943280437224  -2.94694720561842
25
Coordinates from ORCA-job Tc_Deprot_s_job19 E -81.218540236320
Tc  0.18884607107153   0.08437968957991   0.42219522599419
O   1.53748427951093   0.47116814723998   2.07920610492554
O   0.36925563248818  -1.71724892049501   0.85894291999113
O   0.00902182789906   1.88607110503939  -0.01461223908251
O  -1.15967724621502  -0.30219973218303  -1.23412800731977
O   0.59674162217511  -1.50340841955654   3.39671931723956
O  -0.21993742648823   1.67210018490218  -2.55224934750638
P  -0.18419603484505   2.80424108023409  -1.30307434002877
P  -0.06960905313326   0.08008938362319  -2.26383041072068
P   0.44707063331412   0.08862892067779   3.10821595009033
P   0.56404285859878  -2.63530629145728   2.14727214612896
O   0.52774067214248   0.73503036570312   4.46453128072552
O  -0.86360114310999   0.28699980496735   2.30988288435272
O  -0.63158890639354  -3.50270625911779   2.39142748628368
O   1.90201339527530  -3.30713175224764   2.14761774837859
O   1.24128176201294  -0.11836049547953  -1.46616893219383
O  -0.15127425569027  -0.56652047610764  -3.61996343092150
O  -1.52087552759767   3.47868944892591  -1.30344590108885
O   1.01309929982129   3.66921949313345  -1.54751513960536
O  -0.50820620897217   1.29424289156810  -5.50000161923739
O   0.83963839225675  -1.12875595555968   6.34915549257475
H  -0.37959981189957   0.51791638298353  -4.89024791453850
H   0.72612216209104  -0.35144598500735   5.73765192703426
H   0.82780097385303  -1.82586300755385   5.68316940233123
H  -0.49539396185166   1.99127041736400  -4.83395059953070
25
Coordinates from ORCA-job Tc_Deprot_s_job20 E -81.218431214150
Tc -0.02985237580950  -0.00894767969607   0.05919161657269
O   0.42211912011109   1.48215950369323   1.58281385369881
O   1.70776608930911  -0.62814604003817   0.31829656489031
O  -1.76141832397108   0.59808879589880  -0.21284941886259
O  -0.44842146131519  -1.48735965953272  -1.45811099472866
O   1.95834176460675  -0.14212517069194   2.80796591475944
O  -1.90770976279414   0.17429451058390  -2.72595713535891
P  -2.81379843856249   0.68885264226341  -1.41399525228722
P  -0.40140464979433  -0.41889309697316  -2.58384996740087
P   0.43560566287484   0.41176048872070   2.69419480556431
P   2.82426846983024  -0.58867883427678   1.44792512408273
O   0.06754803230431   0.84322095257639   4.08678443804888
O  -0.41899437154637  -0.73567164553892   2.08965508268583
O   3.39305496010529  -1.94102717105661   1.74107696007209
O   3.80513470226044   0.51050605442478   1.14917083741959
O   0.44131859094042   0.70799187974883  -1.91028637808147
O   0.00024813593829  -0.86129075385348  -3.95036166198773
O  -3.93001035549563  -0.29240350245391  -1.22822951781720
```

O −3.20002265331201 2.10931428535255 −1.69161647845254
O 3.26427269468419 1.69086549735624 −1.36280009410526
O −1.75905672812488 −1.04131927883344 4.72270245335680
H 3.42181329196603 1.29273426134393 −0.48155681365435
H −1.73214168007205 −1.33696108991856 3.80298059607818
H −1.09676799541510 −0.31341563715507 4.64406453112970
H 2.36430728230411 1.38755066689853 −1.56040906663811
25
Coordinates from ORCA−job Tc_Deprot_s_job21 E −81.222722341710
Tc 0.44002916300377 0.01412907358837 −0.20327120645853
O −0.23778093085471 1.87242455280482 0.71841613561573
O −0.77182381051601 −0.82262011554575 0.95735992088770
O 1.64228620781573 0.85780559684160 −1.33435077659444
O 1.09123837231424 −1.83478120504614 −1.08400170785699
O −0.56849625960028 0.74558346050832 2.96946717179508
O 1.44000846461016 −0.70636384729469 −3.34121332240298
P 2.22912467981236 0.67217405250854 −2.81060693517680
P 0.36806458454797 −1.51660839687472 −2.42297442146886
P 0.50704727775412 1.55701176509239 2.04542344059589
P −1.34151867362958 −0.62118303899012 2.42281864411221
O 1.55909272965482 0.50101742474649 1.60575033744872
O 0.98021251688736 2.70700068827395 2.86472016082497
O −2.81099535762639 −0.30374997510440 2.39822744686789
O −0.92277887878609 −1.74449528460038 3.33031258038822
O −0.09812620640029 −2.66560056576336 −3.25416072963920
O −0.69085299407769 −0.47487911349381 −1.96423011619983
O 1.80339020473297 1.79410949379578 −3.70731014632605
O 3.69249773060835 0.35378020890576 −2.77524681011261
O −3.07665943160625 2.20647414803943 1.25625819795586
O 1.81798144868959 −1.52871780779647 3.67296947347894
H −3.07229809545631 1.31563452382643 1.66966319623542
H 0.84949925143908 −1.67719534053688 3.60573042400691
H 1.92570632508222 −0.85389218254541 2.98004899385917
H −2.14864832796035 2.25404188466980 0.96700004185626
25
Coordinates from ORCA−job Tc_Deprot_s_job22 E −81.218020262090
Tc 0.58792480919064 0.44243453695821 0.91343873016130
O −0.36072023458018 −0.73844370376112 2.44293257641055
O 2.12122179310219 −0.56166974024930 1.19823507320831
O −0.95787541263396 1.44546053243746 0.64440136852867
O 1.45508676200305 1.68089338113351 −0.65653821326842
O 1.83804374810967 −0.96019727468807 3.70341035634043
O −0.78440345990123 1.81610303411038 −1.87254623377624
P −1.81059980479376 2.01932971735972 −0.56680334474395
P 0.60216673165716 0.98059860615215 −1.74068099883532
P 0.48912079143459 −0.06433593684780 3.56396419767163
P 2.90136838468580 −1.21268596179791 2.43368661363412
O 0.90823578849328 1.26517412318328 2.87992861858236
O −0.10163071292550 0.01012669107294 4.93140913851366
O 3.03045339860021 −2.69638936023117 2.27464959613969
O 4.15567623619682 −0.45292713685911 2.73941713263469
O 1.16939792579031 0.93750958417448 −3.13267264520996
O 0.25231092425825 −0.38076788281296 −1.09054410533151
O −3.01268724492542 1.14619540024024 −0.79393139180502
O −2.05508268558186 3.49100131040955 −0.45030512946589
O 0.95385404785823 −1.68176746505138 −3.73855044047773
O −3.16207947363930 −0.83798231849134 1.22405686308726
H 1.06533314092978 −0.70377127009677 −3.65420027596211
H −2.28028268207313 −0.81512739337073 1.62717121597862
H −3.07985541185421 −0.16494411455665 0.51709481128427
H 0.68122262574561 −1.86271736369982 −2.83022351431495

25

Coordinates from ORCA–job Tc_Deprot_s_job23 E −81.217627316570

Tc	0.36818911384355	0.29582623528762	0.30659857022427
O	1.71090452054185	0.99623143387741	1.85118935846586
O	−0.57841215265455	−0.56232907164330	1.66193547134579
O	1.30364898657636	1.13674802386857	−1.05912782167309
O	−1.00005700174175	−0.37317316576274	−1.23494463017490
O	−0.19130905643559	1.12004452405650	3.53993786883534
O	0.91224033553155	−0.58728659582721	−2.90076715382169
P	1.63380500255540	0.89776413342656	−2.60399355104783
P	0.04473928948236	−1.38741685036950	−1.78738343465604
P	0.64609198981542	1.96378060353053	2.42399236295894
P	−0.72625984627110	−0.43367203052845	3.24107224776611
O	1.11126580277170	3.20575710487681	3.10724581056982
O	−0.32218093738563	2.13712865548105	1.21259935446915
O	−2.15307418383526	−0.47283080606162	3.68955753597730
O	0.22047778279774	−1.39009520116726	3.91117712917479
O	0.98208082861816	−1.57974298005277	−0.55838586768940
O	−0.47374429780978	−2.61156217904909	−2.46200216827743
O	0.94134865555766	1.90571158313439	−3.46820861334117
O	3.10422146365609	0.71453155368404	−2.82299420265918
O	−2.62949788838893	1.95390763927864	−0.55498260561223
O	1.48739137089413	−3.07760460890847	2.03501308172401
H	−1.98032429312637	2.15198547490574	0.13836905058155
H	1.34077519310053	−2.61782621666093	1.19369913852381
H	1.06393853245371	−2.46687070303651	2.67379112094052
H	−2.21005922010847	1.18209345954485	−0.96658804832808

25

Coordinates from ORCA–job Tc_Deprot_s_job24 E −81.216955050000

Tc	0.34894885547608	−0.03858423585599	0.01030757382530
O	−1.22455858295170	1.42120456250763	0.34292212332496
O	0.85757578507793	0.29905916055661	1.76877471073594
O	−0.16467144243127	−0.37018716992980	−1.74534651244121
O	1.92323515581450	−1.49685800521034	−0.33325357684671
O	0.27516334372663	2.78649846191355	1.89198399120364
O	0.41657011502807	−2.84960314798268	−1.88619066708137
P	−0.18761703318571	−1.57068305691238	−2.79626158739647
P	0.90375092431236	−2.66033235703160	−0.35501686197646
P	−0.19980848610449	2.57983170651393	0.35255246219707
P	0.90699635061616	1.51436176236083	2.79976387570895
O	1.00145062727119	1.98319865646706	−0.41799309898254
O	−0.69076653655439	3.93321379619551	−0.08497930566554
O	−0.03407647122736	1.30298587440498	3.94428702493832
O	2.31588129294683	1.89224887661716	3.13623886728759
O	1.39356352184995	−4.01465860106155	0.07716130795091
O	−0.29658311211035	−2.07921069149342	0.44131367757493
O	−1.58844400147465	−1.93775453949156	−3.17679364421729
O	0.78062430110134	−1.33641155780980	−3.91428304087533
O	−0.43611361526215	5.68630223032902	1.91094202768295
O	−0.47310028052211	−4.69053524928620	1.91056090479452
H	−0.58009947534468	5.12459114421994	1.10171993563954
H	−0.82859195099138	−3.79426330416413	1.85382965069288
H	0.25403131482862	−4.58743666294765	1.25080163050758
H	−0.06716058299177	5.00412234180868	2.48375853040232

25

Coordinates from ORCA–job Tc_Deprot_s_job25 E −81.216441338980

Tc	−0.09995601304107	0.40981832401301	1.68319449707917
O	−1.46304264987282	1.61301689686138	2.84169696805962
O	0.04994442621159	−0.67234453729759	3.18199827868083
O	−0.24663866038978	1.49595623171780	0.16070013921146
O	1.26563338393003	−0.78915401428394	0.48644900126257

O	−0.16764120613140	1.12681794736909	4.98077025891449
O	−0.03692514928464	−0.30123716372199	−1.65106337796076
P	−0.31978034486697	1.33556390410563	−1.40929130808900
P	0.10598330439120	−1.32080149988350	−0.38666038664928
P	−0.31160652781463	2.16095759821108	3.73138386778393
P	0.04815465695348	−0.52137394206686	4.77537934605093
O	0.92879363365086	1.92499049850832	2.82387740272070
O	−0.48325773498747	3.51429198785385	4.33757623017208
O	−1.14377976969683	−1.19764445422319	5.38025827644237
O	1.38632851802317	−0.86480878779398	5.35397389945684
O	0.28883281521902	−2.67402507183969	−1.01711970392878
O	−1.13646590445193	−1.11557591560286	0.50443283950838
O	−1.68016219437730	1.63656226523083	−1.94945151125443
O	0.83351016202017	2.03582329572644	−2.09010551010229
O	1.04894997815404	0.59481175693611	−4.32243186852774
O	1.51176638450834	−2.77994692917228	−3.37576864565551
H	1.05222127214131	1.21116876243206	−3.53772558424565
H	1.05124850081075	−2.79757784332348	−2.48937391780420
H	1.64525753474693	−1.83335192662345	−3.48668848012619
H	0.47883158517631	−0.09083738841500	−3.95921070672329

25

Coordinates from ORCA-job Tc_Deprot_s_job26 E −81.216101654090

Tc	0.48237353218840	−0.29337683605705	−0.18704713776414
O	−0.93699173473982	0.74696036073362	1.09661273694202
O	1.59487182404554	−0.32936470464727	1.30886357151482
O	−0.63070041576768	−0.24585166031957	−1.67561367526774
O	1.87966561506047	−1.36418471978347	−1.43033758843518
O	0.94308698428145	1.90499702696790	2.36755427134595
O	−0.01721785549119	−2.50520911202018	−2.69616991631917
P	−0.94511569822144	−1.13234496766372	−2.96764273833671
P	0.99448510485204	−2.64016908514178	−1.43347028774539
P	−0.05508583173241	2.02403774176341	1.09498356726581
P	1.99449417057885	0.59583895606683	2.54224934101138
O	0.79755271329384	1.85639525587580	−0.18189236590042
O	−0.76692220777493	3.33637712912117	1.27802499181664
O	1.64723696232648	−0.04436982592338	3.84989098182068
O	3.38262113895135	1.13741801062075	2.39727135999856
O	1.67324853660317	−3.96059742363906	−1.58202224040767
O	0.12051795063111	−2.42089098788733	−0.15843915986422
O	−2.37408248204792	−1.58017709309457	−2.98796719447232
O	−0.42164627843777	−0.48308761996638	−4.21193699097663
O	0.13164280424345	4.55669123604235	3.47025477991622
O	−2.07977642949718	−1.87978614504235	1.67969711650527
H	0.73008174298987	3.82282729227818	3.65156296705462
H	−1.80880233646483	−0.94863013280636	1.66657737960949
H	−1.45185701955781	−2.25068996581057	1.03993567799210
H	−0.27748079987425	4.19828725446729	2.63586055168048

25

Coordinates from ORCA-job Tc_Deprot_s_job27 E −81.218662923790

Tc	0.55029626052892	0.57993049051637	0.96964043699785
O	1.82861961562331	1.57214601347528	2.38116567696414
O	0.13784648029982	−0.60863554230526	2.33492333455005
O	0.94957358371784	1.76092359132809	−0.42618650839836
O	−0.71827106607557	−0.43586174847243	−0.52669749429505
O	0.24603582538346	1.02162394312623	4.29934375352583
O	0.94717319790534	0.11746418949626	−2.38370958404931
P	1.08165642387649	1.74624588095144	−2.00870861601976
P	0.54044499744038	−0.97214503196291	−1.25562540022462
P	0.61493434888022	2.13667198520643	3.17352838127157
P	−0.00624780405894	−0.59209348112972	3.92890713492618
O	0.80237993586968	3.42037785576107	3.91190673207990

O	-0.51243926240699	2.06922119841600	2.10512132133114
O	-1.40589920008892	-0.92006648611243	4.34873994474744
O	1.09590235219681	-1.37260033151330	4.57643343817921
O	1.63182600247714	-0.91715549085901	-0.16183514460833
O	0.39549319897735	-2.27295758024669	-1.99442812808311
O	-0.09360118779213	2.43184976851263	-2.65332863228333
O	2.43685327876970	2.18351292687818	-2.46487017175441
O	-2.43034737930328	1.02720484601311	-2.34187271694597
O	0.03748688981603	-4.38079159962495	-0.40981610217131
H	-1.64302938633297	1.59142088675204	-2.51475310370558
H	-0.00188152495415	-3.88172630854465	0.41152339026024
H	0.16828991227602	-3.63547701263970	-1.05830977855379
H	-2.04689550787854	0.43201704227926	-1.67429216475613

25

Coordinates from ORCA-job Tc_Deprot_s_job28 E -81.220321732300

Tc	-0.63106033201449	0.21936738820144	0.25512838620462
O	0.43236044862549	1.27792605356020	1.83815077389652
O	-0.25716574747505	-1.32832567224700	1.23916771082799
O	-1.00163145938411	1.75601275677075	-0.71480173983715
O	-1.67493734937250	-0.85648698416124	-1.29142226784084
O	-0.41797645090045	-0.35633656619230	3.59538687349253
O	-0.83236548985073	0.77375407733727	-3.06205002507919
P	-1.24580433207469	2.17315847026611	-2.23891983077178
P	-0.52353422502486	-0.63263967099177	-2.30819896673184
P	-0.74142546500925	1.05399090735860	2.84004207873154
P	-0.06284164286691	-1.75549190314480	2.75644186001022
O	-0.93053908496045	2.06471786760306	3.92032991068411
O	-1.93425543350007	0.77411365224760	1.89169632969226
O	-1.04693545600689	-2.79338943203044	3.19048090431883
O	1.38876575810952	-2.05901447280506	3.03094025519981
O	0.68138823569266	-0.35222512412902	-1.35719972115593
O	-0.33686580010111	-1.64832588495130	-3.38403676009846
O	-2.69730636465645	2.43743679580288	-2.49900610463740
O	-0.27886790454861	3.22984883683335	-2.67701235979606
O	3.43578904206800	-0.61972981589257	-0.53504050257604
O	2.90369444337638	-0.01328853094659	2.14426125977748
H	2.53388916918921	-0.56325541506267	-0.89773215482141
H	2.14526930469491	0.57035835657203	1.95005541116210
H	2.41890114554066	-0.81387371390484	2.46522478292604
H	3.27965499147216	-0.43720194962532	0.41491389011473

25

Coordinates from ORCA-job Tc_Deprot_s_job29 E -81.215375205950

Tc	0.18366849324108	0.08453156757770	0.42229216442134
O	-0.27241629825276	-1.19520346509782	2.12314162012657
O	1.97349782803348	-0.14035580294642	0.87322463108423
O	-1.60624992729760	0.30940013959866	-0.02853459032619
O	0.63984513138064	1.36431678593071	-1.27833227952392
O	1.73857556470216	-0.29499823223050	3.41162986992494
O	-1.37142363417815	0.46480560718426	-2.56688061551208
P	-2.51424566489812	0.47627462373022	-1.33079389223920
P	0.20508098862251	0.25388139227766	-2.27418903397575
P	0.16198108612223	-0.08448810055555	3.11875056626905
P	2.88132371637690	-0.30786700646814	2.17556615195640
O	0.01516306401263	1.20450831614751	2.27509778366640
O	-0.50149211920512	-0.10593589051992	4.46780642035191
O	3.53860587567885	-1.65248558958349	2.21389837426611
O	3.75965796110514	0.88608644227382	2.38585744974148
O	0.86852408903207	0.27545006177858	-3.62325757231327
O	0.35190196807240	-1.03521706564563	-1.43067075776434
O	-3.39150026466597	-0.71843198755478	-1.54130951363382
O	-3.17275989802550	1.82029807271829	-1.36902024643954

O 2.28190654506033 2.57316569082986 -3.48749755957376
O -1.90805194651625 -2.40806942366425 4.33553295556618
H 2.06017809472580 2.63593414810457 -2.54958558650572
H -1.42707136420726 -1.56777973524749 4.52961430283005
H -1.68959300667067 -2.46970357908247 3.39679715842169
H 1.80109370818996 1.73298302516239 -3.68233780183429
25
Coordinates from ORCA-job Tc_Deprot_s_job2 E -81.223682676720
Tc -0.20370377585392 -0.30744913725470 0.58478326531445
O -1.43110977125677 0.80510816681280 1.95521253508040
O -0.42689967041486 -1.76663420880920 1.72151561959668
O 0.03836675716803 1.15601834065547 -0.55431559379964
O 1.00857498919489 -1.42057177472356 -0.81417724738482
O -0.57952840064458 -0.52223196871584 3.94489979823764
O 0.08299084081561 -0.09281649811161 -2.78024538985894
P 0.13422037710801 1.42434869590774 -2.11315239762420
P -0.12839002515056 -1.43695492479988 -1.87086257432523
P -0.33174196264735 0.82169691216118 3.05103149180386
P -0.52213802385795 -2.05314149165014 3.28635865715099
O 0.96102159856160 0.56080132104687 2.22373239415244
O -0.32840446465812 1.96145998163197 4.01141402906331
O -1.80179208313811 -2.73690928932081 3.64888358594648
O 0.74310627475757 -2.69834816072244 3.78728488449333
O -0.17307242131251 -2.57370135611163 -2.83567760016087
O -1.38554059272199 -1.17152295928443 -0.99998182956754
O -1.06752202127954 2.17770050406286 -2.62058273472207
O 1.46432104807690 2.02195244288611 -2.49223977161512
O 0.39630643970140 3.99052819864133 -4.03229749279663
O 2.88612010855118 -0.98390965458651 3.72799755539471
H -0.37794583888042 3.51786427651634 -3.65926905880881
H 2.17842368473309 -1.66458896010347 3.79277995934836
H 2.41393018526955 -0.32900859731124 3.18457924580038
H 1.05660673302586 3.42141016235944 -3.58087131585937
25
Coordinates from ORCA-job Tc_Deprot_s_job30 E -81.214938478640
Tc 0.54128085856666 -0.51648737552113 -0.25568909181523
O -0.63327288827660 -0.37631782147176 1.60368035922196
O 1.91922014365427 -1.14829362421386 0.82454631379311
O -0.83717887604624 0.12137801222977 -1.32412989355925
O 1.71074496994615 -0.64177968786190 -2.05209994724461
O 1.46519519338959 0.19035071343259 2.94680045681321
O -0.34374244422010 -1.24237201547787 -3.43085540347879
P -1.39895123383755 -0.09617902067885 -2.80642653336783
P 0.89029387794184 -1.85750911352503 -2.57103342975502
P 0.24190356011849 0.80021806498276 2.09510368544183
P 2.51048947332071 -0.96167343590118 2.29150552857896
O 0.81805995070181 1.40239553237533 0.79247584752613
O -0.43422015790621 1.77848296763488 3.03054476477633
O 2.34767533813137 -2.20311654403822 3.11138603976830
O 3.87036522016329 -0.33744736495977 2.26034916728928
O 1.57783525481142 -2.82941014483976 -3.47109770759367
O 0.27469128553092 -2.40092077638827 -1.24958999146835
O -2.75792327015668 -0.72453320821333 -2.78360566963596
O -1.23536493456965 1.14070769700047 -3.63438684239451
O 0.37424828375150 4.08696650752003 1.82247576179963
O -2.68782011192258 0.32618406139370 3.53767073844057
H 0.02952653475768 3.37076158283719 2.39893954523873
H -1.93677965813033 0.95744966295728 3.50075696695759
H -2.39248707037780 -0.26460035220787 2.82973658906828
H 0.69241070697215 3.52684567236081 1.09974274458378
25

Coordinates from ORCA–job Tc_Deprot_s_job31 E −81.214636044830

Tc	0.91206861251618	0.62452358014166	0.52217091155682
O	−0.38652104873112	1.68183440467546	1.89018427453224
O	0.85541464687271	−0.79756671969316	1.72983752153553
O	0.97343016784831	2.01749686801210	−0.70155614557556
O	2.19534823658011	−0.45989930816463	−0.83990170980059
O	0.67600169537887	0.53628474584867	3.90021104700402
O	1.05854798745374	0.63649883946007	−2.84414240164518
P	1.14746795561602	2.21144930485997	−2.27818201502596
P	1.04784017566907	−0.65808319576319	−1.86377019589615
P	0.75164986482985	1.85205317924781	2.92948354854682
P	0.60146162477835	−1.00762477217001	3.28683433614750
O	2.02290593713428	1.67105920136819	2.05428212473318
O	0.70473800656946	3.03047644421067	3.84487872064977
O	−0.80217145017840	−1.51765576562840	3.49032945609468
O	1.69611637259568	−1.78517439240117	3.94844503770931
O	1.10749835179610	−1.85555486298869	−2.75019528719341
O	−0.21945173421541	−0.50052818184627	−0.96163140143911
O	−0.01774509251031	2.94337136541058	−2.86998709176187
O	2.51224662159050	2.73355023426296	−2.60943175942074
O	−3.11171435117982	−1.03273429819435	−1.03579937771005
O	−1.65044187417575	−2.29479475216963	0.97533244587901
H	−2.81279433983121	−1.49307670839323	−0.22920222887671
H	−1.37981150728829	−1.97731955022070	1.86682885322489
H	−0.99415080535003	−1.85646914160597	0.40841316280778
H	−2.28173404216299	−0.60101651824918	−1.29663182179997

25

Coordinates from ORCA–job Tc_Deprot_s_job34 E −81.209951510970

Tc	0.49570522467353	0.64660129178093	−0.50522738117816
O	1.45850506513189	1.90615903248840	1.06340624847117
O	0.46874075864582	−0.80269470469132	0.92783192979623
O	1.97233492956197	0.23405291525239	−1.55185293836193
O	0.02623072911234	2.08461950728039	−2.01515860847625
O	0.05351494927722	0.81184237057348	2.88422768779581
O	0.93272827826368	0.56957027358557	−3.84691163370121
P	2.33536405423515	0.05564055457811	−3.10923123216205
P	−0.41045522628469	0.97706622693700	−3.01231231794426
P	0.09597619261686	1.98575369688553	1.77109088421110
P	0.35867511924749	−0.78450271562470	2.48487014725167
O	−0.25934476565245	3.25384860844066	2.46825065788301
O	−0.90335561160546	1.50579355778599	0.63898561987066
O	−0.88102794151069	−1.53098317858712	2.94543799197510
O	1.61861400509185	−1.19578498775696	3.20002750067087
O	−0.70490687768598	−0.22048712005671	−2.06729821085417
O	−1.43691380457968	1.32734020659759	−4.03670941904286
O	3.41510814200037	1.00905406944889	−3.51675752944809
O	2.53768531488262	−1.38626125367431	−3.45309022882963
O	−3.02563236130761	−0.12639151514893	1.95525433071636
O	0.44617036451382	−2.97094519601674	4.85939949943598
H	−2.49866419494128	0.48804763688169	1.42108909080570
H	−0.30043050853176	−2.64770214599995	4.31619895948166
H	1.11727901170805	−2.40961666124521	4.40745675011042
H	−2.30570084054896	−0.67892046970510	2.33782218992379

25

Coordinates from ORCA–job Tc_Deprot_s_job35 E −81.209781410860

Tc	0.43269746520639	0.07077168648276	−0.27719102581001
O	1.72114604528971	−0.00559249147745	1.56416709959130
O	−1.07957568451003	−0.36387886228594	1.01962000130274
O	0.81149825314595	−1.56337996142431	−1.07125238034743
O	1.74956282321512	0.91109163705971	−1.74050862667808
O	−0.13694106489601	1.03793726729700	2.95932779003236

O	1.00788450983870	-0.78465343295640	-3.48476392536795
P	0.99586110240324	-2.16593381032230	-2.55188392037646
P	0.70890936378202	0.69824629605404	-2.87205571325104
P	1.10347318567305	1.34726554005281	1.97827912497839
P	-1.32540483639464	-0.08491713334714	2.54140932106241
O	1.98154185369353	2.33327780110685	2.66627143279566
O	0.43510705327711	1.83683167287149	0.62899589693926
O	-2.62586846093297	0.64985278771218	2.74871967348883
O	-1.12239342224848	-1.28547556806279	3.42578875801356
O	-0.62069757765469	0.56889832175350	-2.07804416609629
O	0.73967398373927	1.63516440438236	-4.03283904196686
O	2.34283163650133	-2.79984566198483	-2.70666540941226
O	-0.19120656633599	-2.98370040288938	-2.95247902286994
O	-2.28718802182180	3.03245569517062	1.43809734430992
O	1.52453360965691	-1.90363518020330	3.72812632467992
H	-1.43421389750775	2.81236544830797	1.03658665531573
H	1.79378361545564	-1.33244102745945	2.98760128754483
H	0.55187678908359	-1.75194318315111	3.68814155644650
H	-2.47069174605333	2.19233815203091	1.92335096995113

25

Coordinates from ORCA-job Tc_Deprot_s_job36 E -81.208920137370

Tc	-0.26048789706916	0.21714000735405	0.46376248386397
O	-1.20013867246252	1.94989099079248	3.96569485617345
O	0.49736503540360	-1.31437787170661	1.55337134039389
O	1.01160882610178	0.44878873328368	-0.88768617155488
O	-3.02334634867957	0.62913780435002	-2.53085497173755
O	-0.08872314355178	-0.27985841228820	3.81781002986908
O	-0.55385358170655	0.66016763540414	-2.88396245557980
P	1.05167277050934	0.52225339464537	-2.48261703219223
P	-1.74641195906383	0.52096539430112	-1.76625733679417
P	-0.59257948891291	1.00551063371667	2.98384620429109
P	0.83314047876107	-1.48012513339337	3.07526885873775
O	-1.58283427059328	0.40713944494878	1.89588476406906
O	0.56452557791377	1.50784818082331	2.10519487794480
O	0.26653602587513	-2.77441119554784	3.61393757934300
O	2.27500056735146	-1.23899052694299	3.43494706898493
O	-1.44807327576088	1.60222510169790	-0.69863313278566
O	-1.45796936484507	-0.80477694256360	-1.01304258403656
O	1.69231089955986	1.80984462543867	-2.94110355568690
O	1.59353350266964	-0.73187390027219	-3.08391796717976
O	0.02055903426107	3.36839175022417	-4.31266785120490
O	2.47367299574304	-3.52253228142714	4.94950720088432
H	-0.75849409415045	2.92978604944097	-3.95443804400118
H	2.75425695944398	-2.68409098701944	4.53005948896289
H	1.56359175859685	-3.48888553304389	4.56919675527893
H	0.72133766033600	2.84193304308525	-3.83650041235088

25

Coordinates from ORCA-job Tc_Deprot_s_job37 E -81.209332817080

Tc	0.19141344089901	1.07838396971921	-0.19930328086635
O	0.87079378338774	2.05942006196957	1.71020543374758
O	0.52775676144759	-0.65972789751002	0.83576915423847
O	1.70865611865316	1.17116303714756	-1.26647022278131
O	-0.53510629246055	2.72485186441488	-1.36460509838475
O	-0.34207783749324	0.25735204879648	3.06609486492265
O	0.59724334530984	1.82806380974162	-3.45958420378886
P	2.08359791317362	1.44422982118001	-2.80665178891726
P	-0.78458999874264	1.81051817431108	-2.59367808029049
P	-0.49251809557129	1.67643005299477	2.30527157019183
P	0.50422128853952	-1.00358273715833	2.35647419085220
O	-1.13506339073324	2.61699382694879	3.26768725777106
O	-1.33116833562640	1.33126313172842	1.00397014401510

O	−0.37025056077388	−2.22266047991484	2.60178149187794
O	1.84944431089086	−1.08288406367679	3.02638844633386
O	−0.86145090116149	0.39796823612628	−1.94506378930403
O	−1.87057828339025	2.19732039672207	−3.54094628308766
O	2.94360056770715	2.66286104727135	−2.93611664202814
O	2.56700282781231	0.19721015727511	−3.47685154648499
O	−2.07931753257233	−2.65346861766962	0.56111532053705
O	1.41901489702706	−3.41749710268592	4.30597463209186
H	−1.80920682309848	−1.91558798862406	0.00244302229296
H	0.59508398346566	−3.29555522082239	3.79477704360319
H	1.83321754641264	−2.58330081609856	3.98397811171095
H	−1.47351873737179	−2.51866471217713	1.33414026131514

25
Coordinates from ORCA−job Tc_Deprot_s_job38 E −81.208421245630

Tc	−0.77240251016315	0.55260078180682	0.15945902722355
O	−0.77454007717001	1.61365545578457	2.14345396133412
O	0.67957878273482	−0.61806885527891	1.00265704085142
O	0.27945871659725	1.66896527236565	−0.89558803950182
O	−2.44526575701640	1.46096168393917	−0.84895787295345
O	−0.44830544466364	−0.64186755020568	3.30805491878950
O	−1.13135321211036	1.65830499862356	−3.01283756834884
P	0.37823945087745	2.06070330459319	−2.44770158153772
P	−2.13747438670808	0.68960136208668	−2.15868763883977
P	−1.52065760793560	0.39227800261081	2.70108947115743
P	1.00167185993827	−0.95559395874046	2.50192150123429
O	−2.56424641472659	0.61535625781016	3.74443493149912
O	−2.02375493714149	−0.31166730995585	1.36651966212477
O	1.20683064745259	−2.44090355561342	2.68598875979440
O	2.06227153625220	−0.09457638123923	3.13167863544402
O	−1.25999322865551	−0.49068163671456	−1.66023980770474
O	−3.28142401041125	0.37915554121125	−3.06549237866179
O	0.51923109807030	3.53674429860234	−2.64289483801396
O	1.38696782780641	1.18409943661579	−3.13120289554384
O	2.42869316090925	−0.92913539900097	−1.58034127222683
O	3.34399387533337	−2.22270873066023	4.30452306684692
H	1.90270210104220	−0.92171804139492	−0.76767821400427
H	3.15630954477115	−1.29096856643927	4.07142308847292
H	2.60261684208074	−2.58096451634881	3.76163832955461
H	2.01705215973373	−0.21247186798927	−2.10442029329735

25
Coordinates from ORCA−job Tc_Deprot_s_job39 E −81.208341785160

Tc	0.18357657839946	−0.04486985388632	0.06463402177039
O	0.57279484631058	1.97071839487224	−0.58812698210537
O	0.65414690936034	0.54080395813770	1.76849825835115
O	1.83308485811464	−1.02945588854508	−0.57267343827446
O	−0.63017168212441	−2.11458009520945	0.44751088250698
O	−0.18508509963454	2.94404031707994	1.63728848378808
O	0.53799901222442	−2.94139815157079	−1.67460837678764
P	2.07852610366861	−2.48218294534842	−1.12796117871510
P	−0.78783966630162	−2.25014216977399	−1.08282595290838
P	−0.68128824735343	2.49666483255254	0.15115494404499
P	0.67202134070828	1.89653026130620	2.62712350139484
O	−1.34152432444336	3.72159061965907	−0.42133645518565
O	−1.55728514354722	1.24093211454377	0.32739840752984
O	−0.13015290733161	1.75648927541203	3.88149920353615
O	2.05847097408279	2.44059055305289	2.75748336548144
O	−1.95869115012162	−3.01109091569208	−1.60388832497933
O	−0.69037632283869	−0.73705502657965	−1.54303236750091
O	2.93720973591080	−2.45798595923022	−2.35543709274018
O	2.48554193404668	−3.48111685481076	−0.07509481752704
O	−1.25773499018788	5.72765281402710	1.33273068366429

O	0.59714630974501	-4.43867589188527	1.61194589999492
H	-0.78784709400613	5.17856649227305	1.97053800737441
H	-0.00377697105251	-3.70658747886842	1.39143561486464
H	1.35453685806162	-4.17154725894350	1.03681735856963
H	-1.34708185008433	5.05320886272873	0.60572636342032

25

Coordinates from ORCA-job Tc_Deprot_s_job3 E -81.223530264760

Tc	0.18425346632616	0.08406683786215	0.42269037949604
O	1.07934363036811	1.63368436457450	1.61115140408711
O	1.38647357808053	-1.09096410126250	1.22989697513305
O	-1.01796070484834	1.25910424080967	-0.38452315023656
O	-0.71082279857269	-1.46555467074223	-0.76574308840735
O	1.55436208049918	0.02577112206127	3.51867913253247
O	-1.18588436761707	0.14233217811129	-2.67328725564270
P	-1.65196552691974	1.50177425807220	-1.82510511796245
P	-0.09618649864029	-0.91845510226075	-2.08151810546511
P	0.46467927525706	1.08657455441213	2.92690597985269
P	2.02047109861927	-1.33364760270651	2.67048300099425
O	0.19448501339555	2.05902893636768	4.02391799504878
O	-0.72749965790268	0.23524071799938	2.39899240556284
O	1.37533277206670	-2.51584633255508	3.34527595002808
O	3.51531476334817	-1.33190307007341	2.62966994056145
O	1.09599843014476	-0.06711382736695	-1.55363285024906
O	0.17399923706667	-1.89092539188811	-3.17851817374367
O	-3.14680827997457	1.50005530842316	-1.78428632281890
O	-1.00680859154062	2.68394601233418	-2.49992853056671
O	1.59740706710136	2.23471506069856	-3.21950832946683
O	-1.22892281539366	-2.06674731318856	4.06473841574216
H	0.66921076103046	2.48657704442164	-3.00858791839755
H	-0.30072210219532	-2.31857921759753	3.85380961497394
H	-1.28510721816785	-1.25516748728434	3.53046457220171
H	1.65355738419942	1.42313350724657	-2.68523694014854

25

Coordinates from ORCA-job Tc_Deprot_s_job40 E -81.210391003480

Tc	-0.41565860612830	-0.11127997526048	0.46200520803188
O	-0.19393448607674	2.19189623071331	3.75256785510521
O	-0.31542436259035	-1.70352118556273	1.70371527431638
O	0.67893012067738	-0.60531663111840	-0.96570666914130
O	-3.07053960159301	1.10018207779860	-2.41118068718299
O	-0.21061642755384	-0.30368225884655	3.84098116203047
O	-0.89078183628309	-0.03217208679201	-2.89200971207088
P	0.62340682760989	-0.63220259695654	-2.56682570527733
P	-1.86870691596296	0.55354718829222	-1.71878005900999
P	-0.14587606574966	0.98444870145995	2.88065350391778
P	0.03837457550220	-1.86634493317020	3.22811409182141
O	-1.37233740458754	0.80406798616386	1.88964059454637
O	1.04785723691143	0.82501781335247	1.91652089223814
O	-0.96675811001031	-2.72822780139001	3.92930245550864
O	1.48136664367427	-2.21584237086424	3.48947358655260
O	-1.00244053475394	1.53027800077297	-0.88087257778478
O	-2.08858932660322	-0.64134499534413	-0.75081805176114
O	1.60723106527441	0.35053297309032	-3.14120678940619
O	0.68476306804400	-2.02712122561669	-3.09797829504023
O	0.81838359954237	2.93844472204979	-2.62582179712233
O	3.35125318341732	-0.27257387952883	3.24559235042748
H	0.15047518970057	2.66194832800037	-1.97408436815082
H	2.74921269703287	-1.04358366803057	3.38077952950142
H	2.74096249472305	0.28968817682926	2.73852392794023
H	1.17564697151378	2.05426142055135	-2.86578571571379

25

Coordinates from ORCA-job Tc_Deprot_s_job41 E -81.210277423670

Tc	0.51310717233966	1.06665198516811	0.96635576798218
O	-0.87636347569753	1.74668043003149	2.46543408695439
O	0.81604054640743	-0.39906434543335	2.06853574793794
O	0.20149930248598	2.52015146367598	-0.15802667651701
O	1.91787356301389	0.38417447355937	-0.56182140762133
O	0.55832658840656	0.76742805391479	4.32859804436319
O	0.47494682917384	1.37131188321563	-2.42219074249787
P	0.15464945026212	2.86734938785473	-1.71214087836376
P	0.76878322158289	0.05355575929066	-1.53585944460010
P	0.27569758583540	2.10458707263803	3.44735049986000
P	0.89556769802101	-0.72134881566058	3.63297681535908
O	1.48046010205098	2.25139862288581	2.47451580660287
O	0.03733757055537	3.20274298178564	4.43001888384829
O	-0.20059140508754	-1.65372299843818	4.04732892602420
O	2.28933509051551	-1.08895297412723	4.03995522280022
O	1.00611365578355	-1.05286796453153	-2.53142626875282
O	-0.45458284792420	-0.15206874861713	-0.59583967230639
O	-1.22357181855255	3.25895646332136	-2.14682488570780
O	1.27599049052778	3.77862781385034	-2.10524156191235
O	-0.81352819502924	-4.49704012264020	-0.61461052991154
O	-1.28125734868639	-2.31005649777546	-2.07668635452830
H	-1.00285547030668	-3.73562362159928	-1.22674969803003
H	-1.39070829073752	-1.63808640677972	-1.37656813323554
H	-0.40483812124500	-1.97894977318143	-2.40314744547011
H	-0.41123189796476	-4.04473412769006	0.13286390729062

25
Coordinates from ORCA-job Tc_Deprot_s_job42 E -81.207163069820

Tc	0.32488384147640	0.17677700034177	0.38640594173697
O	1.63768484260150	-0.15227390142722	2.18499490929482
O	-1.05497138463359	-0.85160235539308	1.45175702258504
O	0.96228854620310	-1.08424140565442	-0.82346098431280
O	1.46455897823768	1.59781677312713	-0.81222243513930
O	-0.39669753888809	0.16935417492189	3.70362027409768
O	0.93226556408852	0.31526292350656	-2.94880670293412
P	1.22648424944554	-1.23862614937250	-2.40047850178495
P	0.43541611116597	1.49796939240516	-1.95541716083819
P	0.78480904510910	0.91385750116449	2.90693998975041
P	-1.24916725568021	-1.11556185731231	2.99194214126938
O	1.46716837537309	1.82636200698770	3.86757629290450
O	0.06294162410953	1.61619994253761	1.68345737741613
O	-2.67557208928213	-0.91249363892458	3.40218140306902
O	-0.61150615089992	-2.39010874056829	3.48260314791120
O	-0.84549028831563	0.97069278596853	-1.25003409366807
O	0.25272745453431	2.70654029455428	-2.83094503738809
O	2.66738949681511	-1.52540261864626	-2.68145546878170
O	0.21298582282010	-2.13796517196061	-3.03300544914306
O	-2.42386445690723	3.05136441896035	-2.69860264572522
O	2.07125976477760	-2.47368473185237	3.82248941931908
H	-1.44840324722873	3.03586732188609	-2.84973115830592
H	2.18783334154362	-1.68826519094452	3.26112395145840
H	1.08790895129806	-2.54258230351839	3.75663951012627
H	-2.46673360203313	2.33584351863902	-2.05077174922479

25
Coordinates from ORCA-job Tc_Deprot_s_job43 E -81.205431596950

Tc	0.59486049912786	0.57474562200346	-0.18830318085313
O	0.92641692157213	2.51033255897769	0.66827914021231
O	1.22478155297576	-0.14912560954417	1.41868745319903
O	2.07588216565196	0.52673841247561	-1.51126061113211
O	-0.57931240769434	0.97060728646267	-1.74307900771400
O	0.48776648597798	1.68535141279470	3.02198270205648
O	0.67184749688629	-0.49168277534668	-3.39290688916660

P	2.26480257113913	−0.12930293122709	−2.94186199023110
P	−0.54634615842772	−0.49417830020949	−2.34138361800089
P	−0.22728283239080	2.34474556064085	1.69556343790181
P	1.10180246314841	0.15012516546277	2.98161005366016
O	−0.89798264294330	3.59119780552060	2.16315732794894
O	−1.09914349351864	1.22246398839018	1.08411513291620
O	0.07213512357988	−0.77778948831134	3.57579226599706
O	2.43491794276285	0.18871050321375	3.65483985848362
O	−0.12035218805906	−1.32132709675907	−1.10679140525151
O	−1.76160173253506	−0.92506400956562	−3.09001632904616
O	2.73048335259799	0.89693310840927	−3.93439622915942
O	3.03388812316397	−1.41464858949042	−2.91182000403567
O	−2.53568846180873	0.07854865058963	3.28694290583838
O	−0.55373170662574	−2.78547220392026	1.46205331391784
H	−2.28933761630622	0.56889900969727	2.48324178354127
H	−0.31571148844382	−2.09988226982439	2.10939397159041
H	−0.43168717929897	−2.32670299634446	0.61515846933638
H	−1.65520680009304	−0.29312282996123	3.50780145755871

25

Coordinates from ORCA−job Tc_Deprot_s_job44 E −81.205418795720

Tc	0.31347756805425	0.84268743958119	−0.46384212964928
O	0.54737049211819	2.34603167394797	1.16407598678765
O	1.40069799424750	−0.37446391522484	0.73749463148779
O	1.52516205473406	1.50910981195809	−1.70555248214493
O	−1.19131423146257	1.73501038200593	−1.69533751508821
O	0.43420824447577	0.52156492248557	2.94142897632035
O	0.12946444050114	1.22283071472891	−3.80967880328482
P	1.65383493010546	1.66267666256419	−3.30099221519043
P	−1.01323070692724	0.65074511237731	−2.79220701798436
P	−0.43437970584856	1.52246696923653	2.01369606371664
P	1.62860724719217	−0.46482655214904	2.28602563662559
O	−1.36450153297107	2.25394273678234	2.92006784489036
O	−1.11350444311524	0.58231803859657	0.93294285057731
O	1.25054256940769	−1.85740690456166	2.76911298619398
O	2.95838125283772	0.02505218368801	2.75748245465220
O	−0.34303593619105	−0.50987239628690	−2.00656622870587
O	−2.19264512590033	0.32497824795282	−3.64654032093622
O	1.84194105262194	3.09737708918822	−3.68608080021491
O	2.63195679563302	0.67775810725898	−3.86028818264307
O	−1.43671587217815	−2.04293779393170	2.11748578691617
O	−0.17723452913889	−3.02717544152933	4.67903239077151
H	−1.53489297260857	−1.18159729015654	1.67833267961462
H	0.51102949839581	−2.58062750888518	4.11781328950007
H	−0.95639252250092	−2.78613774550754	4.16251379793959
H	−0.46262656045979	−2.04840454940214	2.25638032941628

25

Coordinates from ORCA−job Tc_Deprot_s_job45 E −81.205624285710

Tc	−0.46209355799507	0.29428101061280	−0.66877127492427
O	−1.33799559041137	0.23875100096471	1.40385207967868
O	1.06816755031024	1.27776923532608	0.21510057785258
O	−1.48224641669553	1.62762600731483	−1.46529909505168
O	−1.71742856246741	−1.07152757217964	−1.75757233526182
O	1.00413324025474	−0.05297777895556	2.40456360248310
O	−1.87547169894670	0.53305732667349	−3.72654012327206
P	−2.12236651098774	1.96169137211160	−2.90440016497284
P	−1.02574369288506	−0.71265124400693	−3.09880912421104
P	−0.26732128048341	−0.80059317398904	1.74957844428637
P	1.56821742213948	1.38892605725058	1.69719137770592
O	−0.63482637302805	−1.88732451548399	2.72210288930429
O	0.21541942313744	−1.29462391078930	0.33753628217000
O	3.07381102534967	1.23637869462011	1.75952140480576

O	1.01107547084424	2.53653531622172	2.47314041814869
O	0.32684491591936	−0.10703329299727	−2.62670531927231
O	−0.98250804621179	−1.75670594216250	−4.16411968453003
O	−3.60356967751062	2.15026928935977	−2.80021824049241
O	−1.34869345143295	3.03603272218415	−3.60127738189202
O	4.03429310043219	−0.80465282177810	3.10327819035808
O	0.76831614325088	−1.67050528133532	4.97415945235498
H	3.20636822246227	−1.29423608919479	3.12122416417901
H	0.19479084472240	−1.82574247134498	4.17499595245718
H	1.26098654057459	−0.90378252517866	4.66195973727843
H	3.73404096068055	0.00213858147401	2.58230818038540

25

Coordinates from ORCA-job Tc_Deprot_s_job4 E −81.223146455300

Tc	0.71090073106870	0.40460051198070	−0.47096205259599
O	−0.57980978793441	1.73862282097314	0.70399236079722
O	0.44585422247777	−0.81309258785546	0.93947628198160
O	0.95254649631592	1.61613495534390	−1.85207590281325
O	2.00213524681615	−0.92167604372501	−1.56803050639073
O	0.38385664130041	0.83779957255256	2.88624439176602
O	1.12095858492209	−0.03709666979189	−3.78921732716273
P	1.20927698118501	1.59606122917751	−3.43216900654774
P	0.91346815456691	−1.18036658590273	−2.64770819105880
P	0.55761851063302	1.99121972646908	1.73321140709652
P	0.18970980725309	−0.75636635484691	2.49536801579405
O	1.82726114624211	1.59344746660424	0.93930680695883
O	0.56990024210956	3.29309656304385	2.45753549822044
O	−1.23678091213227	−1.12484410098344	2.83544820297059
O	1.23202746994770	−1.52423320103530	3.26493073404078
O	0.94275993706467	−2.48713159864670	−3.36771558367739
O	−0.38504847630143	−0.79988104932671	−1.88146470282213
O	0.09030122105005	2.26365527618796	−4.17064227313605
O	2.59927453421860	2.04937716416473	−3.75764483134854
O	−2.98734505243015	0.82735879676087	1.97503108215170
O	−0.45548840404517	−2.89849080507921	4.83145796695310
H	−2.44788521541952	0.08443937521924	2.32174278351523
H	0.34599841038617	−2.52065680778929	4.39579441344327
H	−1.09042531131060	−2.40923242767455	4.27843255923591
H	−2.30486518754560	1.27835477948076	1.44645788219610

25

Coordinates from ORCA-job Tc_Deprot_s_job5 E −81.222593366220

Tc	0.64588834690951	0.02907563082673	0.78110674381418
O	1.31790042168577	2.80465200247020	3.73322531341921
O	1.22151088146473	−1.22390723880278	2.02541642087983
O	0.06158249596388	1.26866058536327	−0.49093775441659
O	−0.02765401269412	−2.80269214507962	−2.11666880839484
O	1.66591761498262	0.34687453473469	3.98678465883779
O	−0.44748608084617	−0.35903257583198	−2.38811348522236
P	−0.38792490553673	1.25355829670340	−2.00990021619448
P	0.08164348555099	−1.52018500454955	−1.36517060817540
P	1.19502970797035	1.53212037371845	2.96359108866369
P	1.55705716186132	−1.26623323508945	3.58277108665769
O	−0.20660349732435	1.11892211535751	2.44309316559214
O	2.07351438158737	1.37266877491277	1.68997807379417
O	0.39006049050534	−1.85342256592137	4.32688882359354
O	2.90150492414269	−1.86319145993398	3.85873275671224
O	−0.77091706930381	−1.36061378426383	−0.07208543835391
O	1.48637198683990	−1.07207548875706	−0.88765689421155
O	−1.78721072106790	1.78731186217684	−2.17896999953696
O	0.64563798837863	1.89246513423740	−2.90011589644760
O	−1.15712115320247	3.45095876416093	−4.22620830931188
O	−1.60345200681565	−2.73801844589671	2.51549516366982

H -0.30198905326159 3.04549518413865 -3.97009446566122
H -0.91886076093414 -2.40000339281705 3.12896760490584
H -1.35301298925323 -2.31122789781926 1.68136079448896
H -1.67518763128883 2.96893996009602 -3.54468982540965
25
Coordinates from ORCA-job Tc_Deprot_s_job6 E -81.222600815690
Tc 0.87570761762835 0.16866164129497 0.44907879448962
O -0.53985016766451 1.76307086824765 0.95280693449158
O 0.83201589401741 -0.22363853980523 2.27115260590509
O 0.87560827115333 0.55551235545506 -1.37470114956752
O 2.31671648140087 -1.35813634785753 0.02164161278934
O 0.63837206194285 2.15500799774899 3.18275352221053
O 1.33284826551437 -1.79074557236519 -2.27618952133963
P 1.01215473263728 -0.22512198230852 -2.75606433136649
P 1.27862687483873 -2.25003290486157 -0.71145000875079
P 0.59191890922394 2.60200263034457 1.61419424419182
P 0.67002202316138 0.55393469989261 3.65152647647721
O 1.86727162730192 1.99515931544263 0.96937968678792
O 0.45637499729722 4.08656609228023 1.61007462041349
O -0.66862764988980 0.26040100327811 4.27591301355811
O 1.86523188844611 0.37484171085571 4.53280073058503
O 1.50699170517419 -3.72316333436696 -0.70370879199264
O -0.06912988490390 -1.71656282191802 -0.14473054898921
O -0.30616280432873 -0.25819184253459 -3.48336909440144
O 2.19211439329337 0.24601160471225 -3.54534061931964
O -2.71157132516062 1.42904509310447 2.86019479230057
O -2.10829456479854 -1.89569291919996 -2.21258077375103
H -2.15132259648108 1.61385003601893 2.08694842419026
H -1.58002822895285 -1.94587792438945 -1.39748730036992
H -1.52603695717930 -1.30163033581258 -2.73815583956082
H -2.04475155206611 0.98582948204475 3.43211252529480
25
Coordinates from ORCA-job Tc_Deprot_s_job7 E -81.221946338730
Tc 0.69225117095319 0.78300119642058 -0.18308987595643
O 2.06666582474349 1.55890322980181 1.30095893152014
O 0.50922940934869 -0.69177266277309 0.96131512306627
O 0.87128156621114 2.22963590867524 -1.32610139003136
O -0.69898575087969 -0.05396062468870 -1.59980297053978
O 0.71700814375234 0.52203868286479 3.19749231502154
O 0.57377999387451 0.99466398656453 -3.53794718291204
P 0.92842810224498 2.50169541779793 -2.90220080846571
P 0.43955148828934 -0.34645513525306 -2.62560102984236
P 0.91018107967922 1.87553969208839 2.28733641760193
P 0.32951080519599 -0.92971764430333 2.51083872497156
O 1.11349273731022 2.98719104518801 3.26088813564497
O -0.32180198967399 1.93165935800432 1.35036380423167
O -1.11488687851250 -1.23031791926327 2.82661486640599
O 1.31934822819490 -1.92740977605952 3.05663588501776
O 1.68998251834470 -0.38504618597016 -1.70665564172434
O 0.23862949680080 -1.47448608239248 -3.57990167023505
O -0.17307759653659 3.42058007177563 -3.33357479646713
O 2.31633049998899 2.86116887962311 -3.33603691837484
O -2.61590714594052 -1.28135504855998 0.39193429080896
O -0.42423919069025 -3.37195409643185 4.48736229859844
H -2.01345624159417 -0.89490100256745 -0.26313246629876
H -1.04212823925153 -2.75822899615123 4.05309012348125
H 0.38490631576527 -2.99552089198834 4.06291240982623
H -2.08989435717973 -1.22385138651700 1.21310142363559
25
Coordinates from ORCA-job Tc_Deprot_s_job8 E -81.221279774020
Tc 0.03438190769072 -0.49126527680365 0.21248106144967

O	1.17511102371976	1.14434624896792	3.80772202396727
O	−0.01851216480648	−2.09845051313212	1.14409359486155
O	0.08917657631849	1.12951572035556	−0.72155018114469
O	−1.10587413943152	−2.14607286749193	−3.36762940033954
O	0.67814946007492	−1.26052432889898	3.45525734305847
O	−0.60832943848902	0.27381753100386	−3.02357399460058
P	−0.17510022314636	1.64499924686128	−2.19626731968453
P	−0.68685683167168	−1.19163198460683	−2.30057821141602
P	0.75210149693366	0.19070691452610	2.72382979100795
P	0.23887110779324	−2.65317283471743	2.61770883690043
O	−0.62495951065610	0.43807425121949	2.06452376629278
O	1.69745647603859	0.03952374575990	1.50907412471295
O	−1.02649095023403	−3.14866337866302	3.24555201280546
O	1.42253241516311	−3.56863912780402	2.65982446389730
O	−1.62167100308858	−1.01456012243219	−1.07356002824922
O	0.69491974700271	−1.41209099881429	−1.62761511036170
O	−1.35155087217637	2.58436390495249	−2.25352559851049
O	1.08516803938116	2.16620814212324	−2.83639589744471
O	−0.06402267025013	4.45846695655517	−3.74985563248000
O	1.50625119942200	−0.17070577606344	6.10728161292235
H	0.60885073792058	3.76535148059311	−3.57802412293192
H	1.26343787624575	−1.00199861428900	5.68348825824892
H	1.41593157524967	0.42136167694493	5.31223837744806
H	−0.77277183398174	4.00213999857162	−3.24769977142528

25

Coordinates from ORCA–job Tc_Deprot_s_job9 E −81.221240151960

Tc	−0.33592356141769	0.06334747043622	−0.38845245784448
O	0.65043625638121	1.70464080487143	0.69433591338422
O	0.80926026541136	−1.05540591985049	0.59818825032484
O	−1.45312684529995	1.18015692304916	−1.35853213716607
O	−1.29704005410160	−1.57857770222993	−1.39349823501226
O	0.86540059267989	0.26000316510990	2.78784786714784
O	−1.58682879143446	−0.18863218421174	−3.50999455444025
P	−2.17584549942531	1.20224265840576	−2.78657950534547
P	−0.60169365406613	−1.23303924496791	−2.74068747195396
P	−0.09632848439364	1.33427388332380	2.00497691173525
P	1.56290597136176	−1.01952018003433	1.98583220873139
O	−0.37281929032331	2.41647205350250	2.99176758201456
O	−1.29954481323527	0.50525029147731	1.48760366739153
O	1.25240412436695	−2.23781579485151	2.81956782644475
O	3.02401952405160	−0.71323755660480	1.81552521519892
O	0.62192428963090	−0.40323774462029	−2.25819067300890
O	−0.35416832435442	−2.34725110937755	−3.70268486333649
O	−3.65799977247888	1.03711780836941	−2.64474241675466
O	−1.70145895824166	2.35625996904357	−3.61563968112996
O	3.49639932539301	1.88351271482446	1.06884531552076
O	0.37678286175165	−1.51993881492909	5.22659948840669
H	2.55982326579454	2.03352013066115	0.84981227410589
H	0.71684177085235	−1.89111665207036	4.36487860599788
H	0.16652284490494	−0.63067725162929	4.92138473612958
H	3.43625695192273	0.94275229818751	1.34863613244285

10.2.8 Table 6, SI 2

27

converged

O	−0.999633133067635	−1.2892684089924	1.63273629739193
O	0.0228049994036996	−2.07812478570389	−0.856644058371808
O	−1.161008016541	−3.60946525639456	0.704418500739299
P	−2.06169421962495	−2.41906765993339	1.48701032071984

P	−0.740169557130967	−3.45390861511716	−0.827703821884051
O	−2.11750389483565	−3.22819256770455	−1.58834064306334
O	0.0268743501403185	−4.59550348763117	−1.32729047349146
O	−2.54087884103953	−2.99378536832125	2.75053451534253
O	−3.12399233622383	−2.00471197012344	0.446475827191548
O	−2.24559572524366	0.0262814718112429	−0.693332183302321
O	1.88610218281101	−0.598816151933231	0.717666324680778
H	−2.72412712565222	−0.788033750404132	−0.231904814283647
H	2.41026765852976	−0.550608876825928	−0.0965345801407259
H	1.99438504725663	0.317710870342309	1.14607410101227
H	−2.47937849020437	0.809206633764224	−0.180778058678554
H	−2.69070390853809	−2.7581082977663	−0.926054956091951
Re	−0.174404385531504	−0.368514214462279	−0.0140846489946231
O	−0.355438143068835	1.33979787594111	0.770570105813567
O	0.699660577539658	0.547438737634171	−1.70784178260523
O	0.900173196045608	2.86302216757627	−0.714894818921921
P	0.721829489395903	2.51656640480753	0.885116239033581
P	1.56699981011542	1.81133294652171	−1.76234263758181
O	2.97954616013476	1.48234040021728	−1.04794047580351
O	1.7480034321288	2.42695881959021	−3.07850911358907
O	0.220191740403246	3.71041869562849	1.57492290935822
O	2.03385061323269	1.87733956096112	1.33685388008012
H	2.84323852255766	1.70219481551336	−0.0962819661953779
27			
converged			
O	−1.42960297671932	−1.15319802388931	1.22055282484714
O	−0.75099275015574	−1.85207163655798	−1.27930345550668
O	−0.230308752270577	−3.33608127297695	0.644937629976941
P	−0.965136827489432	−2.47072798138919	1.93903884626936
P	−0.990373240923826	−3.313769729174	−0.760260598531596
O	−2.52737947238535	−3.41659527630312	−0.366816787962569
O	−0.539775642207866	−4.36851745039746	−1.67354568529884
O	0.201679480772538	−2.23076970606021	2.85051149290554
O	−2.15197647676007	−3.27078668284224	2.33735782824025
O	−1.87301810522708	0.814861524758326	−1.22964742497082
O	1.46623024505959	−1.17974842267404	0.85083306928623
H	−1.84504816683093	1.7468070463227	−0.714356900924455
H	1.06097285525545	−1.52556395539524	1.72949502024299
H	1.65828015002258	−1.98227513831078	0.350414760532433
H	−1.44465773835562	0.995861345269726	−2.08014893504444
H	−2.59040477463663	−3.29230991556689	0.606917765510129
Re	−0.304642632209674	−0.273398732613451	−0.217427009222919
O	0.234971480879896	1.2816782864092	0.683342888697702
O	0.803870773821944	0.473252450857507	−1.90522441336384
O	0.724565937436618	2.87629168786	−1.17671725425813
P	−0.0257348799325668	2.8321541627388	0.294268940472487
P	1.69698132111878	1.7140205733247	−1.75659582989878
O	2.72530670809059	1.45005857101667	−0.546903285619025
O	2.38569773514191	2.17557372295971	−2.96271084778337
O	−1.50057654625732	2.96103105216703	−0.0220684993349888
O	0.623707453000867	3.76113452687821	1.22764876797992
H	2.22676484475415	1.27758897845894	0.258307096998944
27			
converged			
O	−1.459704123535	−1.14007101223403	1.17940710909999
O	−0.630212630872653	−1.95646196707129	−1.22819859119758
O	−0.371856384252621	−3.41908106145093	0.76072383207451
P	−1.12688374680868	−2.45321111283749	1.97374143584005
P	−1.02162866998717	−3.38077717432588	−0.696919686975789
O	−2.58789302130657	−3.35599344523496	−0.417730796624107
O	−0.591090554909516	−4.49323678142431	−1.54960453518014

O 7.91979978500834E−05 −2.25209605021094 2.94339815534688
O −2.3849430139199 −3.15951471038755 2.32623920309009
O −1.73570873571809 0.792727456035468 −1.21912576667902
O 1.45316206256956 −1.36158743411293 0.990712223735854
H −1.79647031429374 1.62068574203683 −0.631829095157935
H 0.972317198074863 −1.64559623219619 1.85466897827061
H 1.6183621418408 −2.19083822533217 0.524538708335145
H −1.27337079926104 1.09349181698568 −2.01522562372568
H −2.71082237107684 −3.1887128666352 0.541709167986102
Re −0.163702730805474 −0.3709122001164 −0.181627556588927
O 0.300242621161809 1.20441355732652 0.700137606610284
O 1.16476310698667 0.242042588874165 −1.75544973888559
O 0.416198575327981 2.60361764093119 −1.40090266920766
P 0.0489434483761707 2.72094851013524 0.216530920126892
P 1.68349103400128 1.68062080452907 −1.85691674485928
O 2.73652278705737 1.95028363964144 −0.676714323340807
O 2.19147299745612 2.08893285478843 −3.17153048534556
O −1.43249167852956 2.95229362306225 0.225931044307466
O 1.03183030352314 3.62394296798769 0.856118754085903
H 2.30879330918793 2.48858907610694 0.00981846851433584
27
converged
O −1.44183732751658 −1.21404554174775 1.14489218759352
O −0.760902613292007 −1.99026293044192 −1.33758268283901
O −0.423434830825159 −3.49491492191928 0.620653314317679
P −1.07022271625246 −2.54755780759311 1.88952061029292
P −1.14333944813105 −3.42062217268475 −0.794352262327428
O −2.69388236131311 −3.34049326780825 −0.428850297057156
O −0.815969913848695 −4.5284526553535 −1.69509074005113
O 0.119046893080934 −2.36553454674189 2.79092761218702
O −2.31123947644295 −3.2354005885566 2.32864552751264
O −1.77517507778546 0.795362283297357 −1.24695651503988
O 1.47693097735334 −1.38579627469755 0.845660760115029
H −1.80534192204323 1.58926950172335 −0.61696544334762
H 1.01655383772002 −1.80838449869371 1.6714076280948
H 1.95910825910529 −0.610536691711495 1.15921249512047
H −1.30295107568818 1.1243331967969 −2.02551839550392
H −2.75869050536076 −3.1862704317666 0.536105350838498
Re −0.207500988622401 −0.464079012471941 −0.291475624519394
O 0.283575044591422 1.07151151727963 0.67186806976592
O 1.10215317853011 0.240867810807497 −1.85770497290938
O 0.459286006705334 2.59687313065512 −1.33054335521453
P 0.0665746778212632 2.61924985357843 0.284170281110787
P 1.69870396175242 1.64865969670145 −1.80865201045044
O 2.69402399238171 1.74700420071298 −0.543484013810857
O 2.30105983480852 2.14944723808102 −3.04902502279793
O −1.404721574889 2.89438496913299 0.29748798670272
O 1.07743772860389 3.44330087625725 0.990113816813927
H 2.30015545842502 2.36658706674324 0.0974356943505683
27
converged
O −1.47307462684624 −1.08682057851728 0.957574118554637
O −0.41232737526588 −1.97574380816726 −1.33174518221755
O −0.386320007383672 −3.38793950635132 0.705048680797389
P −1.21442613602846 −2.37866626841433 1.82026741260094
P −0.90670969332472 −3.36561986607784 −0.806285977589406
O −2.4919790857102 −3.26132759945502 −0.651229634228372
O −0.463155173371609 −4.5250062535638 −1.58553614397281
O −0.170942796130341 −2.15108719925012 2.86850246090516
O −2.51569385020574 −3.05061823628874 2.08061308987484
O −1.79436667915528 0.666517298180084 −1.17723377371347

O 1.50699622889754 −1.36517358125905 1.01259728332446
H −1.72734822828387 1.60410903086102 −0.778622318508383
H 1.65347039021396 −2.21301522315372 0.57470882276041
H 0.94336889721085 −1.59894271853644 1.83009853469127
H −2.48195985702303 0.219815934609039 −0.662422134133139
H −2.67698263769072 −3.13821120280134 0.307330702689103
Re −0.0255110289649433 −0.355538795308701 −0.272275413686169
O 0.293734342226633 1.22282676293149 0.673535536046491
O 1.47671722626865 0.271748420330627 −1.65017732670483
O 0.522048136362277 2.58843657442704 −1.42718305974903
P 0.0342009296821446 2.72679005576972 0.140265336493935
P 1.87684935177504 1.74973988564548 −1.77470816976219
O 2.81913760607416 2.11267042173567 −0.523333510971987
O 2.46778684905943 2.16496959549188 −3.05048500316837
O −1.45273238753675 2.91718712446046 0.0579749208813845
O 0.932661485042509 3.6688210659192 0.847144085686852
H 2.30595812310118 2.63457864519505 0.117476656754877
27
converged
O −1.5083167595941 −1.11473584754866 0.882701099889329
O −0.58129854543881 −1.96633940053131 −1.50031133627861
O −0.399431194772954 −3.38068936519052 0.542172000782731
P −1.13483212881961 −2.39509762416858 1.71642877319339
P −1.02018406743923 −3.35430330357371 −0.922955736933942
O −2.59441204725693 −3.22674127712713 −0.650422266761084
O −0.670390847552211 −4.51888209049731 −1.74000208983925
O −0.0124077596090052 −2.12588086517294 2.67469958362896
O −2.38943808246898 −3.09142940890743 2.10958575482051
O −1.85764339971818 0.625954738896809 −1.19904236228646
O 1.52215603282004 −1.32806485688273 0.762293998673928
H −2.54951475777164 0.18723890324784 −0.682428209759484
H 2.04850852491599 −0.564738725553132 1.03420254575555
H 1.01290001868507 −1.66516103597284 1.58871951273237
H −1.75862328463674 1.55318855890251 −0.7778351643222
H −2.70850531327967 −3.13101535527675 0.319429665523158
Re −0.107052147911752 −0.438630650153507 −0.400583451547782
O 0.25058109144589 1.06629445805238 0.66129787031804
O 1.39563643473851 0.200555816869198 −1.74949109948704
O 0.602039338708685 2.54402144095407 −1.33199024557916
P 0.0298131003015191 2.59164795669 0.215236191461541
P 1.91357517171211 1.63511794315884 −1.64136632622767
O 2.72751022083067 1.74357375605492 −0.243912333991345
O 2.68435398132569 2.1575116590324 −2.77413836933773
O −1.4450934171331 2.83123338724493 0.0785117311501755
O 0.923426676065276 3.46676350761668 1.01359244316999
H 2.26604314897104 2.39310767412308 0.317507820199918
27
converged
O −1.46386035262444 −1.19629262079467 1.10379281376006
O −0.739209056848043 −1.96357893045961 −1.37810159534872
O −0.397769582948598 −3.45430345967531 0.592470178646279
P −1.06454610508163 −2.51572980044445 1.85421212475493
P −1.1102010047742 −3.38875527398053 −0.827072169850497
O −2.66405775108969 −3.32131467304504 −0.467956840247863
O −0.771858823927615 −4.50133005860829 −1.71873821904366
O 0.120602429104776 −2.30454285940127 2.75651087176318
O −2.2889512335651 −3.22866506770785 2.2996891219297
O −1.77728777796326 0.761222882305863 −1.23937623954825
O 1.47378635298257 −1.37461318962575 0.799536498441592
H −1.31714772816452 1.10832809562647 −2.01700459019765
H 1.02382208717865 −1.77209649614878 1.64671634778369

H 1.99045329175976 −0.608685621169798 1.08023498473305
H −1.82620286915574 1.55298722131496 −0.604798240651786
H −2.73622968192277 −3.17512354966841 0.496914350356476
Re −0.203625372840243 −0.461358858370256 −0.294995315718765
O 0.264135436646512 1.05098053275957 0.711095651211816
O 1.12754197566138 0.211796127087112 −1.81787155904405
O 0.439393467461171 2.55569468189281 −1.30052965575142
P 0.0276821535235038 2.5854382151432 0.311714164101763
P 1.70170571299561 1.62626948009888 −1.7481799926056
O 2.66349521208493 1.7253859708413 −0.456473657195281
O 2.33319650260204 2.14114333299838 −2.96836828997818
O −1.44618794269143 2.84914076691687 0.305877458371569
O 1.02010377249424 3.43263733711101 1.01744827635329
H 2.26061687621973 2.35986580399894 0.165153516630365
27
converged
O −1.47312048416992 −1.06637989986939 0.948417011488692
O −0.506927407554902 −1.88695976391999 −1.43701825640568
O −0.240884478982307 −3.27328168326491 0.603387875069215
P −1.0594014721603 −2.33449262980665 1.77849431749229
P −0.871520794419953 −3.29284400220989 −0.866382078054584
O −2.44499372843599 −3.28373092648023 −0.594519038947156
O −0.417557529466939 −4.43405752684363 −1.66690658048686
O 0.0332724212629975 −2.03290779848162 2.75733103956778
O −2.28420858856034 −3.10355023151953 2.1270184623589
O −1.89699121695435 0.62615099579594 −1.21208559364572
O 1.53630953157166 −1.17407738434173 0.813075743450325
H −1.81217772869561 1.63071135179342 −0.849729632516605
H 1.71681144653458 −2.01216114305855 0.369898852022654
H 1.0457342561024 −1.44599575813533 1.66780312658534
H −2.60071262866048 0.224370465623386 −0.684475072165601
H −2.56907085850393 −3.18957879948518 0.377234640106431
Re −0.150487777525439 −0.291515515851251 −0.350713797813676
O 0.226006602337961 1.25589211663669 0.648584695081752
O 1.22135882349059 0.390738937368763 −1.80811833367419
O 0.723368844879877 2.79747803335479 −1.24120738611363
P −0.0777129203832683 2.77837276928888 0.194761194685418
P 1.90380228307102 1.75512075553074 −1.63644855033068
O 2.78961766743721 1.68135399135782 −0.291020675185894
O 2.69314767097682 2.2533100948748 −2.76301544508195
O −1.54669209382283 2.84651096208074 −0.170501359435131
O 0.494914170620149 3.7569146733721 1.12975854331087
H 2.20751599300418 1.5191079157687 0.458276308169054
27
converged
O −1.59650840739403 −1.1653105649904 1.10662073594744
O −0.370691228218008 −1.89273575312357 −1.16420549827903
O −0.424596346357818 −3.42211485386694 0.797721563016981
P −1.39315786283425 −2.51223630135999 1.89421251057825
P −0.812039690107686 −3.33777762477232 −0.752810199878823
O −2.40276139983226 −3.36611207669509 −0.746567944729959
O −0.205242070830999 −4.41253963971885 −1.54659016296228
O −0.462496897711511 −2.34208887488162 3.05771437855944
O −2.6854808360694 −3.23867104529591 1.9894228665392
O −1.50781073474486 0.813368402404023 −1.22780458046108
O 1.31371960941353 −1.39377434809441 1.40533899810953
H −1.00590826797066 1.08999254699726 −2.02428735166615
H 1.47433019487772 −2.22225670936477 0.935103897007504
H 0.697928283719161 −1.67459648319714 2.17153972280049
H −1.57151736512821 1.69429643139553 −0.696785904513546
H −2.69312439420844 −3.22428604909223 0.180327094767837

Re	−0.120594957914636	−0.325480048448644	0.0137650653511929
O	0.23665339754068	1.19831008374807	1.04821116905378
O	1.43034849980518	0.371340195878438	−1.27470610257695
O	1.26102886341509	2.7932163981926	−0.576376383707315
P	0.143130280336186	2.76070059270601	0.665879736285504
P	1.3309500708575	1.79560305348445	−1.83053201425906
O	2.81854344865449	2.13476546925021	−2.35501326194334
O	0.328485956260509	2.06627659069298	−2.87946089306342
O	−1.22030006051196	2.97058108590991	0.060247546267305
O	0.657859612951958	3.65906601966948	1.70714400590395
H	3.41865229441207	1.87696351273537	−1.6562089984914

27
converged

O	−1.60077782289826	−1.20791932918165	1.10576815047448
O	−0.12086869051415	−2.04397898456595	−1.00133733460126
O	−0.665082805055611	−3.54476304592515	0.923465940759591
P	−1.39606002009419	−2.47187857371961	2.0405324006395
P	−0.884196754208052	−3.36616940603191	−0.646626792820103
O	−2.43234745824102	−3.00581457928298	−0.810951115854522
O	−0.485142783343082	−4.5518189876458	−1.40706618994011
O	−0.279328645168096	−2.24175489902455	3.0330866023463
O	−2.67936044641785	−3.04564669308005	2.47647711149359
O	−1.47150221021494	0.622832123668679	−1.27430730744761
O	1.4053678044648	−1.38970354861492	1.41029680278997
H	−1.58924257735563	1.52883220599651	−0.805358311768498
H	0.759046561463964	−1.72028787432276	2.20001575234482
H	1.61104947793491	−2.19606878725146	0.923031708654924
H	−0.955466643556411	0.872193990979606	−2.07069030515592
H	−2.62383691395096	−2.32623329083209	−0.149289454479134
Re	−0.0478242488683346	−0.371241180894376	0.0692515342324009
O	0.18144838830522	1.22749848558767	1.01595292697547
O	1.52337659011739	0.307876812004619	−1.19904676830057
O	1.18859960035214	2.75798740698784	−0.677822970728314
P	0.0369051741534501	2.7601568401539	0.532981805186914
P	1.35612114289545	1.6823806673366	−1.85597324060348
O	2.83634677630266	2.07385087536373	−2.36287065224576
O	0.372167284584962	1.81943411447613	−2.94739103651655
O	−1.31585946537898	2.87410687595379	−0.118306131522636
O	0.487000423923391	3.74115284981931	1.52828913025918
H	3.42886825846837	1.909475926332	−1.63021224993307

27
converged

O	−0.866151665174639	−1.2470758008625	1.70277659450619
O	0.29517382103187	−2.30283655720538	−0.610318834443703
O	−1.34583568495217	−3.51250373434001	0.812513583369146
P	−2.12220904278998	−2.15801030521318	1.47943805126608
P	−0.75388959959067	−3.47917025991782	−0.66814913005079
O	−1.97340033198733	−2.99247992465068	−1.55804584709725
O	−0.198719770592396	−4.76846798581497	−1.08827291104664
O	−2.75363730877439	−2.60760139292343	2.7300403867616
O	−2.99879938078136	−1.60547897558568	0.361885808922283
O	−1.55607029627057	−0.145467681011652	−1.16178532757577
O	1.83076827412193	−0.833864162771722	1.41704837104285
H	−1.51237098324712	0.814205885041702	−1.3674562213252
H	2.11961695667884	0.179027227425809	1.57799666940426
H	1.39394066406488	−1.12123684462693	2.23105706003037
H	−2.31368172614833	−0.411802772074154	−0.572781730780003
H	−2.51451420252199	−2.42044260909738	−0.943117408872964
Re	0.151131787693679	−0.527981366562952	0.0938315349362001
O	−0.0172413721276811	1.24715657633566	0.74616045037199
O	1.24972952975643	0.332866319398011	−1.53039335409229

O 1.6198123795352 2.60826785173368 −0.492032583409979
P 1.11639822612224 2.31728708593244 1.07308973040047
P 0.902258765781761 1.81328963865095 −1.68887273974504
O 1.82767336652508 2.3036078489793 −2.91954562010391
O −0.505137696815966 2.20221684788679 −1.91274602075744
O 0.634270861361247 3.57193200276758 1.66422169746438
O 2.26467408157493 1.58311347663654 1.74114490309044
H 2.66561035581117 1.85594960615707 −2.81578710273392
27
converged
O −1.56446613741778 −1.20357484474541 1.12169285661159
O −0.531525766781009 −1.89447596841893 −1.27260734596974
O −0.482689983362867 −3.46917190654992 0.65526120196622
P −1.36681007500917 −2.60022540808774 1.82366845294094
P −0.953677398558449 −3.35336574126771 −0.864289690652963
O −2.5424552077926 −3.34393756673945 −0.770643170729986
O −0.424589401002604 −4.4138141076623 −1.72730211278925
O −0.398598630316982 −2.51555154613158 2.96668664417103
O −2.6825641663686 −3.28546453900975 1.94262407710725
O −1.52131845640837 0.824919795314662 −1.20966860154968
O 1.34258772692796 −1.37787852603961 1.41043609690796
H −1.03537014004126 1.12853795033752 −2.00698972625038
H 0.729085611555053 −1.89648390148919 2.04214925649111
H 1.5728272105425 −0.577220871020637 1.90025523459872
H −1.5305865709616 1.67539010018727 −0.633978535365789
H −2.777763970542 −3.23701741562152 0.18014589856044
Re −0.133383278390325 −0.419874636725728 −0.0693870697900769
O 0.326123478066282 1.05276065141065 1.02923372799485
O 1.39646453616366 0.302934019471803 −1.3714904924521
O 1.35580783444883 2.68718082476391 −0.554431485063003
P 0.269514174436185 2.63115155055176 0.716427047003044
P 1.3259024473668 1.75365144961018 −1.85847872336492
O 2.79884265012457 2.05962784181047 −2.44159092186341
O 0.287267899268483 2.11487827327698 −2.84279688939106
O −1.09978461406624 2.92060725744978 0.166130206274326
O 0.856307527713665 3.44353285714787 1.79216693076479
H 3.42425269810701 1.68738440775551 −1.82132285133676
27
converged
O −1.60619775202344 −1.19679997827373 1.06479794048989
O −0.096211478095557 −2.0094376214763 −1.04201476545518
O −0.632274112597597 −3.51268069952012 0.881702976830036
P −1.37914350513489 −2.45357263004897 1.99793471933866
P −0.850270960050925 −3.33254131195045 −0.688753373652605
O −2.40099394921794 −2.98302223453454 −0.856506050538269
O −0.444284987579458 −4.51718501549362 −1.44781966856493
O −0.265568543568772 −2.20654404787823 2.99187675842781
O −2.64950701442814 −3.04994397771695 2.44160041872481
O −1.47647844887758 0.587381370219812 −1.25870241227789
O 1.42137611649212 −1.36890532644074 1.37844165199731
H −1.61459957083638 1.48616741797256 −0.779697666579409
H 1.6350001976777 −2.17502258818486 0.894613159093078
H 0.773207791270666 −1.70154084775205 2.17436448782004
H −0.965082478539334 0.854517331710062 −2.05352032006951
H −2.59696932906313 −2.29865609298704 −0.200732266432506
Re −0.0394617488723632 −0.373567736043406 0.0652911951867214
O 0.164046472735647 1.19565246314797 1.05784772881413
O 1.54792918666712 0.298325753922707 −1.1437578101139
O 1.13737075557506 2.73842437774356 −0.641423288153716
P −0.0104454412574202 2.71898117828752 0.575792661130105
P 1.34271698677127 1.6620538765694 −1.80855115925391

O	2.81405688603195	2.09318191654533	−2.31143710615269
O	0.36233979874811	1.76616614932713	−2.90759272605624
O	−1.36537456957671	2.81810517536331	−0.0738073604276605
O	0.427449961523913	3.70975591315887	1.56732668799927
H	3.40676974980252	1.94520715216106	−1.57537439200854

27
converged

O	−1.5761558082294	−1.16139873545498	1.10838307520757
O	−0.525194691455258	−1.88251404206988	−1.27606502371886
O	−0.492706959936354	−3.43076199083153	0.673237519070048
P	−1.37191081335999	−2.54397739858143	1.83073323757678
P	−0.962351851134406	−3.32684004843127	−0.846863896452091
O	−2.55211249664817	−3.29854786560384	−0.751017218393986
O	−0.44922129051874	−4.4068892997107	−1.69562968003796
O	−0.395313340263085	−2.44058397842479	2.96657652186775
O	−2.68458157513289	−3.23027103356277	1.97113966335698
O	−1.52127162131408	0.795729253890773	−1.19992032244481
O	1.34278273409189	−1.37353859020085	1.37577437123767
H	−1.53430612140119	1.65107123400541	−0.630112121169763
H	0.727474707077117	−1.86301976315464	2.03359832405063
H	1.61674681853526	−0.576819720216931	1.84930113751421
H	−1.0425256804056	1.09648067557563	−2.00339458470981
H	−2.78620662466875	−3.18387068304786	0.197807150469876
Re	−0.125504757118621	−0.42021503079935	−0.0660467332372195
O	0.336560822403829	1.04467729030544	1.04040540640659
O	1.42164918428648	0.255198924876006	−1.34083277139639
O	1.34482966790716	2.65443030894317	−0.578134005735914
P	0.268770534656897	2.61232491329232	0.704354112251545
P	1.32867431975601	1.6929082016732	−1.85901673009674
O	2.79767772447129	2.00829460959883	−2.45014757815628
O	0.286573385793044	2.01860084290365	−2.85245675800261
O	−1.10422440578107	2.8961905839973	0.159782756799154
O	0.863428958114162	3.44575683610673	1.76009485782959
H	3.4278191938509	1.66208452037654	−1.81965070584635

27
converged

O	−1.53836645517885	−0.810692070606516	1.40778180798397
O	−0.772989692272818	−1.83825184297884	−1.0319303911511
O	−0.683673200545666	−3.15506921016487	1.11054356059374
P	−2.05829474372855	−2.27420874924604	1.62769936785464
P	−0.536978728315884	−3.28196105156794	−0.466904425508479
O	−1.77929757715102	−4.12050766907918	−0.932377799174557
O	0.797246711803475	−3.83120469560362	−0.801494039692907
O	−2.32919874260815	−2.62184090866568	3.03129828674382
O	−3.0978434588784	−2.58068897145944	0.569527195156191
O	−1.93861853167113	0.885535388951922	−0.553365988586444
O	1.65931665260561	−1.51177741452218	0.440811705882993
H	−1.71471154928683	1.81212670298329	−0.180033452429033
H	2.22637950804224	−1.09321318753791	−0.230895755352196
H	1.45017926645964	−2.41268500969317	0.0940238956352459
H	−2.573349618399	0.492349597275645	0.0713040102045665
H	−2.49537519041433	−3.60282962080034	−0.414977759911173
Re	−0.186740126570633	−0.30950479931571	−0.0376884279444358
O	0.500863071755386	1.19052290229252	0.871681236455405
O	1.2142702616347	0.14765280160184	−1.58679161219441
O	0.551109483599316	2.5512213432739	−1.22841350523501
P	0.310847494710543	2.71422008018069	0.401667529723489
P	1.74389293419138	1.58174825013392	−1.74051927470817
O	2.86819617313774	1.77924176354549	−0.602040348535179
O	2.23282481545763	1.95499853187275	−3.07197915838502
O	−1.13852998670614	3.07742225316209	0.539112599540555

O 1.405817703802 3.54965543295445 0.951714521185027
H 2.52242350635356 2.40224016317537 0.0641462155045292
27
converged
O −1.52939070624092 −1.05347055904601 1.51351944892484
O −1.03371947249082 −1.68075399699654 −1.0705860951688
O −1.30359414029511 −3.43068464147175 0.724363090628985
P −1.14096202509789 −2.44666100290794 2.12461560213316
P −0.672928073664684 −3.14475769904505 −0.691647868324835
O −1.62753289505264 −4.04477296937718 −1.62923443642797
O 0.743224962217821 −3.51439771579573 −0.861934155472179
O 0.334544105399612 −2.49285193769621 2.46910401046131
O −2.12337696046846 −2.98173159673605 3.07727241818617
O −2.1427207427818 1.05601329519883 −0.76188166816679
O 1.34573875045967 −1.20963608923991 0.620398100104706
H −1.843177661441 1.12490586635871 −1.68044809862714
H 1.44139302492892 −1.9294887364834 −0.0312474778000619
H 1.00441654693304 −1.75283828394012 1.50711449102859
H −1.88584018682638 2.00797821142958 −0.361442354276888
H −2.52995917896912 −3.78488578538579 −1.44658607596412
Re −0.483811337766599 −0.126504688991086 0.0215727466468607
O 0.245719072006339 1.35805335135459 0.87932605354868
O 0.381135930875252 0.67196195579132 −1.79278503429032
O 1.05964741085037 2.92521543868857 −0.857449651139463
P 0.166322064293334 2.9366680748248 0.51784808384556
P 1.56526455245835 1.64776207461398 −1.71828540667784
O 2.69855034910283 0.98017193924711 −0.795160082717078
O 2.11773264379394 2.11791024654294 −2.99116862228089
O −1.26891526390226 3.20934108974015 0.107764408707806
O 0.807970857825231 3.80087888888263 1.51571678444061
H 2.31366835567879 0.451075264726657 −0.0868582070832964
27
converged
O −1.55889534727883 −0.900153088721799 1.26668295565717
O −0.846704918577348 −1.8023777368496 −1.18820921627369
O −1.52383042407119 −3.33787541249659 0.672962859513967
P −1.33961371889558 −2.27252212005903 2.01315178578024
P −0.687886897654481 −3.2576747217474 −0.66130710385484
O −1.61147009106142 −4.13860641452914 −1.64772000236232
O 0.687407135454739 −3.78244230288136 −0.61533806165595
O 0.0925101083730072 −2.41782267240752 2.45362201817674
O −2.44851136026887 −2.61438888005989 2.91797454045472
O −2.09781027011655 0.917605334284234 −0.694493151299061
O 1.42696139845824 −1.36640848654895 0.600164252013694
H −1.82328010392567 1.89475575380582 −0.41211924114309
H 1.51383933201945 −2.13783106990428 0.0102360656489884
H 0.986051645582034 −1.78164815914514 1.45012533930796
H −2.67911517008139 0.612548763862314 0.0169717531999198
H −2.47469586031096 −3.7278411525021 −1.67739670028807
Re −0.282519707724283 −0.210426500170831 −0.159926832294169
O 0.391409769204069 1.31713247850598 0.708285871295097
O 0.916838649319176 0.395464887089531 −1.84950940188125
O 0.759415490213653 2.84158987082119 −1.22986837145417
P 0.192223536404026 2.88542912891041 0.310812542692359
P 1.74702854992722 1.68583289452753 −1.81079530125404
O 2.83998270597982 1.52654427163571 −0.637173416300985
O 2.36741056573515 2.11629437126727 −3.06468439831336
O −1.29740405666592 3.11418564627091 0.183517797928844
O 1.00993209527496 3.76916006278714 1.15345679319077
H 2.38012694238806 1.36597525912625 0.192476627754128
27

converged
O −1.30487681441326 −0.838956771753723 1.44895514819098
O −0.786454086281105 −1.78526998422666 −1.09333605934874
O −0.440904469828692 −3.15496941836944 0.98742999548261
P −1.77405596204794 −2.31005549620984 1.69782659725785
P −0.484279352222884 −3.24998765375127 −0.608674309166622
O −1.77587361595588 −4.07613412111094 −0.931689973819108
O 0.801560994062183 −3.77832098558577 −1.10747438190454
O −1.81373088338966 −2.69497098855437 3.11814886275228
O −2.93349013688213 −2.64406062539308 0.787996223751203
O −1.99908105564009 0.880843068773053 −0.573308806298698
O 1.62812857672623 −1.47138879240226 0.41902073692037
H −2.58230227120499 0.503604199008001 0.10441022446267
H 1.23961053263046 −1.92466588844983 1.18463166669242
H 1.64610976166719 −2.15543038040091 −0.281621368500227
H −1.75973256210226 1.8631329775964 −0.236992793630087
H −2.42581142696031 −3.59090683264354 −0.303418354937251
Re −0.190385787618474 −0.218769521574248 −0.173004772141477
O 0.51588274730719 1.29057387212808 0.721007172165941
O 0.944540200248269 0.415856463511872 −1.87349490717307
O 0.701562723449968 2.84423544544314 −1.22237066333129
P 0.260898093938672 2.85405217889757 0.363136550071763
P 1.71235733446512 1.74542437196608 −1.86539017998177
O 2.86410236343253 1.62587628665401 −0.740809451717592
O 2.25872396089098 2.20723956505894 −3.14148611820928
O −1.23826381745124 3.06115315012305 0.354999259439442
O 1.13482571836348 3.7387751775284 1.14742253687407
H 2.44033923251779 1.5576206980244 0.119987165045972
27
converged
O −1.56646613310167 −0.863519069446491 1.25082768186251
O −0.886725105268124 −1.76335832987398 −1.21914795770147
O −1.49193280081217 −3.2996703799304 0.665760881487901
P −1.32593497803656 −2.22697470728091 2.00087658409241
P −0.682983519313388 −3.20531552202647 −0.683558696136742
O −1.60142401563465 −4.11911997757443 −1.6458155500141
O 0.703781614005524 −3.70244656686861 −0.657014407329956
O 0.10596656566859 −2.35125674994838 2.44748998635742
O −2.43018923354399 −2.58548032135879 2.90558545281639
O −2.08220887273277 0.897658011620515 −0.705206076450039
O 1.42596794549704 −1.33148383732166 0.572512266258018
H −1.81881112475145 1.87848044269878 −0.401194167269421
H 1.51002263551203 −2.11070219573485 −0.00989018814656891
H 0.999560135903891 −1.73745881806054 1.43290037330873
H −2.67657825869802 0.582159054089051 −0.00860369087957407
H −2.47581502547836 −3.73212090219676 −1.66354069132036
Re −0.288686914973762 −0.209951057415167 −0.167645047392166
O 0.400219778599689 1.28590117171657 0.74040186752595
O 0.907335441230854 0.347103180411083 −1.83115191238189
O 0.741177105804851 2.78999224971725 −1.21310968653329
P 0.187709584615384 2.84160980684269 0.333599673816848
P 1.73457123008512 1.63815427536338 −1.78656195749965
O 2.82287315162056 1.47592819530709 −0.610345978821441
O 2.36166254266308 2.06990323981669 −3.03679724477463
O −1.30354101642927 3.07217425312745 0.212609017154937
O 1.00694714553547 3.74014194714492 1.15949767401567
H 2.36290211444146 1.31415261205263 0.21942180348588
27
converged
O −1.61795527964714 −1.257464752796 1.08467784123171
O −0.0301304170650764 −2.05623751329707 −0.951048718864639

O	−0.69060303291041	−3.59888243614089	0.901214089439475
P	−1.46682253025633	−2.5450944723193	2.00263660702413
P	−0.819241046284036	−3.38177963828858	−0.673931802629841
O	−2.35413662277449	−3.00665537485764	−0.915838268488195
O	−0.386892706979894	−4.55146259365469	−1.44064492272537
O	−0.401783195953522	−2.3437935984562	3.05454043729355
O	−2.77658297858984	−3.11504973539228	2.35815360010392
O	−1.3898156113023	0.5999787116859	−1.25292359248888
O	1.36405780956927	−1.41820652056081	1.55392735689961
H	−0.831399093554798	0.873392553328853	−2.01628261834747
H	1.63830105201846	−2.19971078096031	1.06054958482115
H	0.682562045242538	−1.78514827348845	2.28871192397279
H	−1.53540808118833	1.49730507199665	−0.780602623693915
H	−2.57721870914359	−2.33863589613542	−0.252299212683189
Re	−0.0153197157564285	−0.395581308068972	0.145807570055216
O	0.154214911287318	1.1976940441185	1.11309104901766
O	1.63760306755982	0.29305848969087	−1.03879250992315
O	1.24884602078861	2.7457259791215	−0.515728551613068
P	0.0368771383165901	2.73423669721412	0.637497271313243
P	1.45886944331655	1.66250149739175	−1.67108900462317
O	2.92400201042707	2.09017752490322	−2.20269608733384
O	0.485807249681851	1.80106696823674	−2.78843942595694
O	−1.27842993793678	2.8513591335478	−0.0876715618682227
O	0.422179807134625	3.71242606711069	1.66148928238805
H	2.75781840170139	2.62928016094068	−2.97240771866457

27

converged

O	−1.58701070007006	−1.19367848848205	1.13222979956894
O	−0.477732218541917	−1.95214522707338	−1.19976343004881
O	−0.558266565873772	−3.50009808579329	0.750866252816398
P	−1.45000157010534	−2.57856547876378	1.87460624061867
P	−0.967766019790247	−3.38931651833915	−0.785681489911253
O	−2.55861828145971	−3.31904701049213	−0.750593477300114
O	−0.451531090260287	−4.48058725328415	−1.61728710116626
O	−0.513621944421315	−2.49202228546055	3.04316702420225
O	−2.78856563273371	−3.22097569159808	1.9688402409036
O	−1.42179503880786	0.79023800494157	−1.22554785553306
O	1.30550879316108	−1.43907543846609	1.51108845509748
H	−1.44628704287206	1.64390682901314	−0.658892309316715
H	1.58453826812346	−0.644646078226468	1.98426781280324
H	0.660039417637349	−1.9138493079469	2.14469699069742
H	−0.891974429068698	1.08872799003683	−2.00003615838375
H	−2.82376404104159	−3.18384299409861	0.187065470228408
Re	−0.0895450365489669	−0.458289386049047	−0.0148357763417039
O	0.343675766432488	1.02856406475131	1.07417733418544
O	1.52530748837226	0.212575643400046	−1.26703209319163
O	1.45870399199362	2.62397214288211	−0.497813819877016
P	0.327817721888987	2.6021303807629	0.737690528426541
P	1.46324663696882	1.64759271661799	−1.7613472248358
O	2.92841258082266	1.97314846871485	−2.36357536100459
O	0.440744115469672	1.99469166373422	−2.78532531870223
O	−1.01361646132244	2.90555534001364	0.127498875204878
O	0.879865123489698	3.42861081063992	1.82023470710301
H	2.76163618213433	2.52092517756051	−3.12679831200372

27

converged

O	−0.889955138970588	−1.24527245253108	1.77132648647945
O	−0.0363120721666822	−1.94255316967176	−0.765172545369588
O	−1.15536474684591	−3.52748267582957	0.796437353394736
P	−2.00375431238152	−2.3249616902593	1.62978422691804
P	−0.778962927335608	−3.31924950761719	−0.740379345737715

O	-2.18620126367796	-3.08420665251382	-1.4546395363209
O	-0.00931607921708762	-4.43542472392303	-1.29491136737584
O	-2.47348986931132	-2.92140342633991	2.8854287445592
O	-3.0678950122941	-1.8668487543017	0.608955807521897
O	-2.19390346538867	0.124423079065991	-0.604798220134107
O	1.92392769131059	-0.773118868822782	0.674868487603716
H	-2.13307870172016	-0.139425035145128	-1.53090223234817
H	1.97007518136706	-1.61002596134773	0.191980944012313
H	2.42187296407432	-0.0980810468896239	0.145746363041935
H	-2.68757551591842	-0.644072615824205	-0.0873417182566965
H	-2.72831213649205	-2.65078813257687	-0.736249470947294
Re	-0.180030528836778	-0.212737931667974	0.146339282077031
O	-0.242638443931062	1.50144276119656	0.862486680745047
O	0.564432832236887	0.565946240037173	-1.66312056992904
O	0.844278293730526	2.92051269776859	-0.825690243476486
P	0.68162235207606	2.82292888297892	0.820951979600987
P	1.56091075457469	1.73719016600491	-1.66751542529336
O	2.7950602245354	1.31739939291203	-0.716642588283854
O	1.98412254869553	2.21417472121468	-2.98788473344679
O	-0.00517568244702132	4.02315573285036	1.31067348162274
O	2.04916552016984	2.44930128096296	1.34889773951205
H	2.61589754244872	1.81367768455662	0.183270418778529

27

converged

O	-1.62693446137356	-1.21788718213264	1.05038069273312
O	-0.0242311728413016	-2.02210404342324	-0.98536451383749
O	-0.671283951493136	-3.54436269077612	0.886196543530217
P	-1.45676755202764	-2.4905379253264	1.9794081557448
P	-0.810433160583221	-3.34130015231102	-0.689903967037838
O	-2.34648804853501	-2.97047397732505	-0.928918188550922
O	-0.382957198333121	-4.51977278778497	-1.44673264805754
O	-0.391064773181893	-2.26869217143922	3.02749075388096
O	-2.75599673496271	-3.0754621816687	2.34955025840804
O	-1.39604336103248	0.563890862265639	-1.23875243404421
O	1.38061547468621	-1.39574229431829	1.51598054580536
H	-0.84342963818637	0.842832067305207	-2.00592130210418
H	1.65089464352024	-2.18571125741405	1.03425364445333
H	0.700196705409391	-1.74789863313619	2.26360941017421
H	-1.55244713403591	1.45985148735073	-0.765572499634152
H	-2.56782388666982	-2.29211126000927	-0.274862656282685
Re	-0.00718188425616324	-0.396233090413184	0.137432188598249
O	0.147820779685269	1.16888338310703	1.14313372791776
O	1.66322948613941	0.25218208393905	-0.989255538865851
O	1.22199441181132	2.70304612978861	-0.507491374795268
P	0.014890325800274	2.69229906825168	0.65567055552498
P	1.45544555961382	1.6060798623194	-1.64123929828033
O	2.91385709733942	2.0521789172853	-2.17772922328683
O	0.483655363466308	1.71127360557955	-2.76422822000925
O	-1.30269671514786	2.80460556868353	-0.0662366809716454
O	0.40236649779298	3.68515555579988	1.66540529080173
H	2.74021333038843	2.6205110553816	-2.9244032334503

27

converged

O	-1.68183047732355	-0.915807400221799	1.32384045920403
O	-0.701490590786583	-1.77907565567938	-1.05108332789123
O	-1.60775358417756	-3.35128789241538	0.701981651225986
P	-1.66517474264587	-2.31069559721053	2.06319170163457
P	-0.641363343529341	-3.23693676705833	-0.541371230366277
O	-1.4713890072582	-4.07985156771609	-1.64034671283875
O	0.708472278921858	-3.80727223006865	-0.356854793224771
O	-0.353077457708983	-2.51411416554877	2.78136723967743

O -2.93101189045347 -2.63945132186576 2.73600359762253
O -1.65777738785582 1.01503077556392 -0.843542001194676
O 1.26501975796583 -1.5393730794381 1.14466199423894
H -1.2410561328753 1.26073848077014 -1.69842684612883
H 1.37386830763964 -2.31003679668903 0.552605393441123
H 0.699605662739216 -1.93053583584039 1.93446105862353
H -1.57093208537734 1.8843465573436 -0.30034657550783
H -2.31956678463459 -3.64727317240807 -1.73479214046312
Re -0.202811328644807 -0.259396660181546 0.16380104739071
O 0.46220364080558 1.20316022567407 1.1754167513916
O 1.24789808009429 0.263355073201166 -1.26560468236792
O 1.30677317481107 2.7117478233703 -0.632735128648808
P 0.417377556097547 2.75822567670009 0.793059994376931
P 1.15846902674467 1.67920401335032 -1.84181231977545
O 2.57768740976145 1.91176636809003 -2.57562313208043
O 0.0420936258215161 1.98116944134354 -2.76300063960882
O -1.00164836528111 3.10045954984055 0.418654908387903
O 1.17668208695791 3.61522209042664 1.71419799688135
H 3.25013257847663 1.63118204507924 -1.95580425976011
27
converged
O -1.59190395513219 -0.83977225388343 1.31730397142252
O -0.900177028961456 -1.77573172778301 -1.11724565685825
O -1.78840854273018 -3.28126842432546 0.693724412288293
P -1.5262126595838 -2.2309851654645 2.05123938532701
P -0.90337032463175 -3.23396348299342 -0.588022260237544
O -1.83219556411426 -4.06644891947629 -1.61670313710992
O 0.442855186896058 -3.85665104633692 -0.525011570638668
O -0.122443219994178 -2.52841662497626 2.50535029107714
O -2.67402427061704 -2.47260929699681 2.93821398915009
O -2.00150385333352 1.05326664411055 -0.549829070049669
O 1.34959731437482 -1.56528371990947 0.703331750610333
H -2.57807296340492 0.669436108591489 0.129232378868574
H 1.36319294700545 -2.34466459560034 0.11326435187233
H 0.858111974734045 -1.93817413191556 1.54100924220478
H -1.71940727224455 1.95200881952273 -0.179152897332799
H -1.22570249972102 -4.43610848012885 -2.25421344141737
Re -0.213569605705539 -0.245067183414428 -0.0759036254338646
O 0.493862415633425 1.2462322605669 0.799338972793961
O 1.08317940735685 0.249157242662982 -1.71393894629824
O 0.456518882161898 2.65204777789204 -1.28753763259008
P 0.346007229908743 2.79756129644997 0.349650260024138
P 1.59830749104959 1.67980705739566 -1.93354205791298
O 2.84039792688585 1.92508391702733 -0.946354513875653
O 1.91136564881044 2.05222513243009 -3.31776470228498
O -1.07357427366439 3.20554454222241 0.605035992876941
O 1.50845623668965 3.5709629286671 0.847934771397302
H 2.53811336474132 2.45631129349376 -0.187510269509126
27
converged
O -1.16729718381565 -1.24786098157129 1.7147509583162
O 0.278140575485401 -2.33074974566473 -0.418392935993522
O -1.53427577997209 -3.53573671535192 0.805753574866877
P -2.38208713402338 -2.18089409806859 1.38063732889264
P -0.769409800643023 -3.50176938594844 -0.590260001224547
O -1.87227258978396 -3.00712657540474 -1.61964299412436
O -0.170668919994198 -4.7899310024238 -0.950908646434595
O -3.1433795116655 -2.62465817153582 2.55743284542869
O -3.15568446570783 -1.6551439865083 0.170967847314294
O -1.51371560597511 -0.203572619668875 -1.08104278041043
O 1.94391135368976 -0.801026038644339 1.41638917139181

H −2.35156062466222 −0.473345363166687 −0.601852208194864
H 2.57179954030995 −0.838433105430484 0.682430147405549
H 2.06125648045524 0.181279665270845 1.78493477049844
H −1.42642889964149 0.741935537717743 −1.32983362972216
H −2.49494663921902 −2.45287413971482 −1.06929053963375
Re 0.104586787160488 −0.555044330214956 0.273095964423158
O −0.0472690643914431 1.23151800315716 0.857327169854972
O 1.4883751393403 0.175671404589538 −1.21296936980113
O 1.71127556577833 2.52490342744139 −0.299739217858909
P 1.06568654570823 2.31031304452955 1.23358616581366
P 1.13515286931148 1.62368446758659 −1.49052093811727
O 2.14472502618105 2.07431823127799 −2.676428280716
O −0.252821836224551 1.98870472364669 −1.87572996620453
O 0.540852800120086 3.60174551134286 1.69508257731352
O 2.14416151479145 1.60684906076096 2.02860574914057
H 1.73129384979706 2.83174318157534 −3.08248276856826
27
converged
O −1.6303265033754 −1.3596688844138 1.07522927171685
O −0.431927407191179 −2.07304304683677 −1.28888170664408
O −0.39064965769023 −3.52410633370656 0.750334586863811
P −1.06453107113987 −2.53900026371888 1.97697159259341
P −0.914976010203254 −3.46736983747108 −0.743976006175189
O −2.50420420252973 −3.2936678809681 −0.618314807213153
O −0.539175270759293 −4.62800261142808 −1.55039535107336
O 0.133013384566894 −2.09776261230755 2.77180578178597
O −2.16975396341514 −3.2857137698339 2.60558965998366
O −2.07647220730138 0.536597474267656 −1.61204492886122
O 1.26314822110677 −0.890271553634658 0.867403022220804
H −2.17960373514994 1.33281812993653 −1.07033258898272
H 0.916622931677521 −1.37996376845348 1.71839462576181
H 1.58979145714123 0.00837437862352486 1.13881171741268
H −1.40002082032658 0.754584160429571 −2.28387540943408
H −2.65565806611322 −2.70328618926181 0.133878337685347
Re −0.457196939052911 −0.40481021355771 −0.291001575466264
O −0.61234484968049 1.26215976229839 0.603101366727866
O 0.553119955102998 0.535234448461418 −1.87994828829075
O 0.635683536452268 2.81022132918246 −0.826950131531411
P 0.503031361943411 2.39120887407094 0.770963193795299
P 1.37894048930141 1.83732692844164 −1.89994118048706
O 2.798054302151 1.57881437545499 −1.2352599981168
O 1.45109342459077 2.48951779172903 −3.21226847798424
O 0.0355469829372892 3.56970596196459 1.51159363249678
O 1.78932693364855 1.68461001754639 1.15424670710519
H 2.61886772630141 1.54999334334767 −0.263233057524881
27
converged
O −1.14639705635054 −1.278185707045 1.77636325526282
O 0.0559436805706167 −2.06208530099825 −0.61952165702213
O −1.38301998929004 −3.54667800732507 0.760865689052497
P −2.29580696120084 −2.25100892508969 1.35478048733465
P −0.710061197166165 −3.42779813534771 −0.68403128040768
O −1.93850029842616 −3.18345018826367 −1.66594184048058
O 0.0951583029367614 −4.59882298391958 −1.03744568116994
O −3.09548906692756 −2.7644840136379 2.47637769171277
O −3.03971537699861 −1.73101091885131 0.11374737581254
O −1.77104020771638 0.317006767371273 −0.592260986885331
O 1.89885363605614 −0.80928093640567 1.38950543927706
H −1.60213630016104 0.588281769715798 −1.51344968464464
H 1.65255267657502 −0.871724245309142 2.31941524387672
H 2.30506857859666 0.142316719879795 1.25177336481401

H	−2.42767593383017	−0.454539845810113	−0.45622939959165
H	−2.5422355297524	−2.61408689974394	−1.10831298526975
Re	0.0221198598559503	−0.383926811502663	0.331361747865888
O	0.0876543896580761	1.29068956144338	1.20684509128619
O	1.24987256364854	0.357360245624684	−1.19845901870811
O	0.947304944389604	2.78956006665793	−0.568500633564035
P	1.24509736657333	2.3683895275463	1.00939276837001
P	0.840975653673481	1.71317854187628	−1.75975790134772
O	2.09117673829538	2.15290566606549	−2.68570397360447
O	−0.431568952842829	1.81830051646232	−2.49851576569149
O	1.10261560591356	3.5868852114452	1.8196146189167
O	2.55400445345747	1.60119823256489	1.05600204691834
H	2.87464842874681	1.94551008159172	−2.17601402904016
27			
converged			
O	−1.72688555418476	−1.17114381673561	0.838965267556491
O	−0.294405349547415	−2.1730682488517	−1.21868056853603
O	−0.504045698492909	−3.41133716401741	0.931704533267434
P	−1.55295554720249	−2.40282991056222	1.82525464860836
P	−0.768263381434687	−3.55863510985326	−0.630597873383005
O	−2.35789863138383	−3.55599478985066	−0.748348348856048
O	−0.140280470423438	−4.73459852060076	−1.2398242094175
O	−0.768998030398553	−2.05171112668523	3.0408078462639
O	−2.85739744611162	−3.1265403931236	1.87053607955154
O	−1.65920953681515	0.473250314355266	−1.77459535257192
O	1.12312312442062	−1.05997181030421	1.30691001361842
H	−1.07513090535139	1.2521487886414	−2.04430105962082
H	1.50206112153446	−0.169045567627554	1.62306803633057
H	0.542728732445497	−1.40539298271236	2.03706276088488
H	−2.30853400891042	0.826471659596344	−1.15674035559316
H	−2.70913424498766	−3.34404777882682	0.151034431539308
Re	−0.209094095716967	−0.462060777342119	−0.289425689671869
O	−0.2022645698073	1.27895120365471	0.488153532620392
O	1.41325586565045	0.312276606545486	−1.54989330810245
O	1.76854804269802	2.53015223653563	−0.330917752956361
P	0.944060961758798	2.21182410215465	1.10577330221774
P	1.31265908660515	1.81562750641481	−1.67745853525166
O	2.51982302304374	2.26194540760519	−2.6556222338004
O	0.0332728524037038	2.37603992619721	−2.2210197215255
O	0.451293326694041	3.49631404681303	1.623814972489
O	1.84809427519926	1.33106647759439	1.91934433845041
H	2.31497706130773	3.15480970998073	−2.92310476574746
27			
converged			
O	−1.4696791036487	−1.18122479773358	0.991090240383809
O	−0.638002430114467	−1.79079223380747	−1.51635535978673
O	−0.214351822421798	−3.31670897257447	0.403291278256079
P	−0.996741488627265	−2.51594477475821	1.68002516424371
P	−0.904384846983958	−3.25577961045213	−1.03337635657374
O	−2.46501740182008	−3.32393211206348	−0.698161467408281
O	−0.456822025464001	−4.31232373665301	−1.94531320944592
O	0.112606204514549	−2.28126438577808	2.6581583112518
O	−2.19966595625242	−3.33365947037119	2.00339875162597
O	−2.03785372198298	0.684251261390369	−1.11495334088906
O	1.48084651887069	−1.0755310119525	0.793531241332563
H	−2.73014017140537	0.355647134074666	−0.527436564252218
H	1.03173840214436	−1.59805564445312	1.54317142973405
H	1.77913744127401	−0.250796143013628	1.20469945277156
H	−1.84953716662782	1.69949211930906	−0.808622029710159
H	−2.54844037964447	−3.29913113535486	0.28412793039724
Re	−0.276118517329794	−0.278009650007199	−0.354562604739799

O 0.101704103205324 1.20526613342056 0.739449044981764
O 0.896318865513133 0.610683649809941 -1.89362974849393
O 0.987303462369169 2.92439933629965 -0.825364497313684
P -0.0968736817336692 2.76001986792007 0.414537027406313
P 1.82261001583619 1.77202407335667 -1.53503014021117
O 2.73558109299193 1.22887016197338 -0.28285212469199
O 2.66125998303424 2.31616461661034 -2.61087313580382
O -1.47272478197019 2.91180382627972 -0.209676836894311
O 0.292938325237345 3.64902993135939 1.51649159674355
H 3.09370908402894 0.390001561456249 -0.573864059257552
27
converged
O -1.53058216143229 -1.21921726078604 1.16446104740838
O -0.826053544543791 -1.5724680962645 -1.43134274327195
O -0.115606436532918 -3.15549974490405 0.365974288507216
P -0.873314988972338 -2.53468953961105 1.75551568433835
P -0.925869567526529 -3.07233884640399 -1.00206508902079
O -2.45024919294899 -3.29030184370267 -0.566710386590635
O -0.478863651274581 -4.02714900696077 -2.02165586159275
O 0.255005188240275 -2.24283796219496 2.68575187346073
O -1.97002759790247 -3.49152965686048 2.07636422360131
O -2.45270596661934 0.552452713424311 -1.43396368831342
O 1.20702237577183 -0.568069030993565 0.843669552458176
H -2.53452767714002 -0.341131553717221 -1.80432549839835
H 1.50182851656268 0.318326606681205 1.32373571609417
H 0.966051791625782 -1.20995308559739 1.58338438736119
H -1.89586121508329 1.05639864475723 -2.05299932207232
H -2.45710827587751 -3.35967266891321 0.418781304879651
Re -0.640844580447475 -0.0246030610937431 -0.162839778945239
O -0.546593333279386 1.60973832289116 0.751380799313241
O 0.157521650484662 0.987570381182532 -1.82679526264104
O 1.21918143881603 2.82314831309488 -0.473930667678293
P 0.718052238140704 2.5635776739687 1.07243272252644
P 1.46140317806643 1.80441012337352 -1.69902787634873
O 2.61627489872697 0.810734207716418 -1.21068763488441
O 1.86190221895248 2.55623516377468 -2.89056428557037
O 0.335210335480345 3.85780893628123 1.64949136277843
O 1.74858769307477 1.69347919890126 1.75516694755732
H 2.28956666863085 0.17008105566008 -0.567301816008474
27
converged
O -1.55568192502354 -1.09059120958046 0.932119788811426
O -0.602066325256392 -1.58764434881758 -1.59095776003473
O -0.214707848100428 -3.14766467651517 0.305323989772848
P -0.990719495853978 -2.39903681110756 1.62002272569953
P -0.927094416449147 -3.03242949328403 -1.11056357826806
O -2.49950682473001 -3.02418461487659 -0.746850365388648
O -0.570322152531091 -4.09956339578683 -2.05021091627142
O 0.11328466961646 -2.11123188269243 2.57522522739176
O -2.16191579353753 -3.27219469938479 1.92509801561988
O -2.61066695001217 0.145987247088309 -1.22093671215019
O 1.29632860288907 -0.491475990954599 0.754428192239108
H -2.97859859679096 -0.307620078303724 -0.442338038035009
H 0.969426425917282 -1.12538765209296 1.46061729915417
H 1.51451144501345 0.405371795942147 1.28498668624423
H -2.45914101265797 -0.554169239662611 -1.87677588622214
H -2.54738876533113 -3.15463505430562 0.231970748910125
Re -0.497385682519812 0.00331854504606444 -0.363121451122812
O -0.534840284497791 1.62829898528215 0.574986691536144
O 0.524482981471042 0.98663965964457 -1.89913903668854
O 1.33145088675675 2.89291292177988 -0.469658978400858

P 0.682676200859253 2.6052857123418 1.0095576701216
P 1.75599285802305 1.88464702720396 -1.66088601485749
O 2.92032377001443 0.96990102777059 -1.04053824796046
O 2.24530232490934 2.65751986005678 -2.80351201377606
O 0.223436732485218 3.87907724730993 1.57468622330835
O 1.65517281301085 1.73876069519271 1.78404061567053
H 2.55704636002688 0.29460842228494 -0.455674865771923
27
converged
O -1.76181135821354 -1.16567022128372 1.0004364552852
O -0.466937417818081 -2.0189502277138 -1.20500997279101
O -0.512520950627063 -3.38178792421552 0.891107698709721
P -1.51344782338664 -2.44425647111542 1.9099530382769
P -0.880128527039567 -3.43740380790195 -0.655207503509328
O -2.47487759307703 -3.4491945141868 -0.6727806360619
O -0.286677285635929 -4.56637849003755 -1.37913594479788
O -0.658626507615487 -2.14827865960944 3.09285661345794
O -2.79964652719135 -3.19384391098128 1.99808603510237
O -1.72642314714565 0.513440287557848 -1.73822804589335
O 1.0890382064058 -1.03872921866522 1.32220383647825
H -1.53203244274685 -0.0779744247979392 -2.47435501110087
H 1.5246920824382 -0.176420535141659 1.63827987571546
H 0.566521644093911 -1.41650703112681 2.08148358367673
H -1.20278831139637 1.35688111835331 -1.90889835841535
H -2.77154166865277 -3.29511822331608 0.256766932528653
Re -0.329374939361429 -0.36193561045358 -0.154718340635561
O -0.173509494963938 1.35120740591145 0.621458128450036
O 1.22476969266203 0.333779057347516 -1.56052054163826
O 1.78414616974836 2.51181890480383 -0.32085925327311
P 1.0469573332022 2.2328313614279 1.16909754162843
P 1.21048109780052 1.84319017236692 -1.64591880226466
O 2.3953344783985 2.23192349464428 -2.67908252064361
O -0.0604609737199145 2.47317861356485 -2.12439043408055
O 0.659424525508902 3.53697058064517 1.72310949027421
O 1.96047791620392 1.30580174178421 1.91900604917262
H 2.32836182512218 3.17592653171835 -2.80283990941151
27
converged
O -1.56679453270382 -0.888663837128678 1.47271167003872
O -0.654435879886478 -1.77207554036022 -0.937035684138067
O -0.614267606536477 -3.19892154677959 1.15619966157448
P -2.02979639573006 -2.36481649848515 1.71013111429495
P -0.445932982259634 -3.24067541088581 -0.43510176176134
O -1.68118769512401 -4.05566836791998 -0.947879345727583
O 0.899070027848452 -3.76955715590997 -0.757568114364587
O -2.18949140446461 -2.71921220255765 3.13171467328901
O -3.07985046278872 -2.77401268417391 0.706922571423946
O -1.64068739527092 1.0434602130506 -0.734424634396639
O 1.47441717973318 -1.57549483012599 1.01772162921037
H -1.27062133506995 1.30107390600087 -1.609558842341
H 0.977223933411435 -2.02410351704632 1.72158406897405
H 1.55454377922918 -2.25400082105243 0.313053724237381
H -1.51721499382432 1.91295685919149 -0.188712015202632
H -2.40890668071469 -3.60710045697317 -0.36892786996113
Re -0.161963145755381 -0.255536188237131 0.148936672198097
O 0.530227010777206 1.17590912700001 1.15355903117864
O 1.25505707936267 0.281228452556889 -1.32700012053217
O 1.38882420924933 2.70367395172781 -0.624451156539673
P 0.53149734995412 2.74450307630126 0.812090707284791
P 1.14347364232257 1.71090343877155 -1.85788033582003
O 2.51419082212937 1.9554702448228 -2.67362422750693

O	−0.0289381874713803	2.05705810602543	−2.68733429835854
O	−0.890211867458419	3.1196911923083	0.480497914310328
O	1.32927013198469	3.55462484035834	1.74273778157313
H	3.22190539675779	1.63378565968307	−2.11646282457329

27
converged

O	−1.71187778892324	−0.915068580804752	1.32106708232755
O	−0.633422634313611	−1.8001589250453	−0.998178454221769
O	−1.66853765341924	−3.3519839516838	0.69711230267977
P	−1.74251475799448	−2.30741070716151	2.06151186942676
P	−0.642105282240409	−3.26582783452129	−0.495981623384584
O	−1.44838448365006	−4.0724628546562	−1.64008264560113
O	0.681077911538284	−3.87471527049666	−0.260076522419314
O	−0.458074018687939	−2.54675364312097	2.81555005637964
O	−3.03607936249911	−2.61087068299751	2.69279114940183
O	−1.57726185895852	0.981985770729453	−0.854411278120129
O	1.24454747994356	−1.58935919603482	1.25306972086061
H	−1.11226358946352	1.23468602845055	−1.6867736611765
H	1.3845909612599	−2.35239188632594	0.660762110319613
H	0.640926321142938	−1.98156828682182	2.01200654708784
H	−1.50773057632743	1.84645657764903	−0.310135816011425
H	−2.28555413154459	−3.62362787360348	−1.75510280986016
Re	−0.16197762667036	−0.293994665523668	0.210369458564389
O	0.458067934805419	1.15993176514196	1.23415562062098
O	1.38614413560126	0.180771890540477	−1.16919028203356
O	1.42382937464461	2.64283840040935	−0.534056245458476
P	0.470635793289195	2.71615774874893	0.84660531216713
P	1.29337089046838	1.58815486456863	−1.72271782623459
O	2.69308152653323	1.84513427044106	−2.49243259373301
O	0.17586563000205	1.9068930328616	−2.65681511604164
O	−0.922373562985241	3.07925300824324	0.400622103041066
O	1.19182761410402	3.56816228083812	1.80140566322259
H	2.50359175733779	2.53026871975418	−3.12917411227211

27
converged

O	−1.77133687859946	−1.13766588694222	0.963096506903759
O	−0.434441819509148	−1.99027168497579	−1.23108411080985
O	−0.506144588485429	−3.34268730296764	0.871016212993354
P	−1.51567156714752	−2.40590690811949	1.87981413925695
P	−0.862178021628579	−3.39746995757665	−0.67692286494852
O	−2.45837543662078	−3.3960253432677	−0.706170230289562
O	−0.27630868538159	−4.53668845463696	−1.39177227706666
O	−0.664996869396666	−2.09939002002585	3.06383647443487
O	−2.79563951999869	−3.1657358649398	1.97076426146792
O	−1.73063815745308	0.479053637757727	−1.69736514053075
O	1.09675641113722	−1.0376683333398	1.29997919429374
H	−1.57580183005541	−0.132198188234515	−2.42688735157545
H	0.57452975434452	−1.41155218821535	2.06235467293082
H	1.53855301340124	−0.179437732703818	1.6205270724909
H	−1.207891270336	1.31542546333659	−1.90667274210596
H	−2.76089985486257	−3.2541461377166	0.222758083787471
Re	−0.316881603879259	−0.354749395393092	−0.154751035543538
O	−0.197984056115905	1.34649917533202	0.652056196413843
O	1.24757149503539	0.302372450406636	−1.50128500301043
O	1.74760341918012	2.51624961064188	−0.307564618440682
P	1.02506196538772	2.21889729798471	1.1901828769437
P	1.20565653831454	1.80727204516309	−1.62164827000889
O	2.39889698099952	2.19103815230077	−2.6476428547747
O	−0.0664114503622838	2.40838606291524	−2.13379933768801
O	0.645794058663031	3.52025743806161	1.75688308172952
O	1.95776336853384	1.29441374337767	1.9195275297573

H 2.34281461311991 3.13622833193977 −2.76733049412217
27
converged
O −1.21783745612442 −0.982018199760574 1.43659027642452
O −0.0110605004103132 −2.27007054818251 −0.731640243762095
O −1.81919892014139 −3.26746921282554 0.658342935778866
P −1.21434897816158 −2.44748924521701 2.00138173036536
P −1.21053056059912 −3.26116529308216 −0.806976870516967
O −2.32262420311768 −2.48389412791594 −1.68237676044191
O −0.968684534909992 −4.60118141339847 −1.33729899777742
O 0.219383654019663 −2.91608782491613 2.19440180238864
O −2.18855185387711 −2.6736962701656 3.0797450666666
O −1.82418894003903 0.188823849176284 −0.816350740440085
O 1.79308163213296 −1.3018590758089 1.20424209594496
H −1.78426179386671 1.20394491727381 −0.665660835976795
H 1.18167241990108 −2.09782605329302 1.66176239366394
H 1.91706760560481 −0.650232278582473 1.90139972604382
H −2.28673897085262 −0.121654280298884 −0.0141025987300753
H −2.21432075581207 −1.5280509151677 −1.60840571698002
Re 0.16995923659256 −0.509516734604014 0.00552384577864114
O 0.366571965646204 1.22662778047497 0.693141537222549
O 1.54893721734428 0.000390524699031281 −1.51450100548904
O 0.367919577164538 2.21264489055524 −1.61136767981761
P −0.0692182228705816 2.57770989674572 −0.0680138850310638
P 1.79810404364492 1.46936220559593 −1.87031820699228
O 2.71714550860189 2.10911421901089 −0.715622959935141
O 2.31442753329167 1.74548326564687 −3.21426546506332
O −1.56766805458623 2.64322791810139 −0.105589171852121
O 0.769177117538703 3.69968796550929 0.421325061263789
H 2.17518622629491 2.72969404530016 −0.193465339079693
27
converged
O −0.899900949015007 −1.27664163798997 1.72229543863269
O 0.0959964726731022 −2.1455591018599 −0.710343281067323
O −1.28806886419949 −3.56222475247068 0.7958716832051
P −2.0914619270678 −2.27972573726873 1.54601264113901
P −0.794963125573657 −3.43252024157199 −0.720963795607217
O −2.12521413775826 −3.04630791555147 −1.52002366846391
O −0.15470461958647 −4.65204651330101 −1.2157955690107
O −2.63950403085423 −2.79056732643056 2.80943304128269
O −3.06333161307356 −1.77170920278696 0.472533555601429
O −1.9021249923782 0.000970006730718787 −0.931621874608455
O 1.77548288349547 −0.768659775431887 1.29369077661869
H −1.81377025061558 −0.40470872191023 −1.80221541433026
H 1.39534122965254 −1.21429844836492 2.06402438537146
H 2.02285422166674 0.221157780063557 1.63469680815431
H −2.4977607816093 −0.608877182394662 −0.369024818504003
H −2.67610037512827 −2.57862585551453 −0.82658535576655
Re 0.00488834970556179 −0.374027872717402 0.115769172014067
O −0.0505974177122404 1.38337195151244 0.760131093852113
O 1.00309100248 0.382607210175209 −1.63035692000308
O 1.9504580809933 2.35739416870016 −0.322052546497389
P 1.17581826773027 2.35333273109167 1.13526685390737
P 1.23992987780342 1.884012253787 −1.69136100260852
O 2.512279057687 2.0424113873346 −2.69183000310688
O 0.157323338594622 2.77555410056121 −2.14342733173102
O 0.785780451485362 3.71436821493077 1.5211444396797
O 2.09089141880772 1.56221762559971 2.05440655374381
H 2.42676842949807 2.92360285994852 −3.04777487882464
27
converged

O	−1.20114470541104	−1.31055049492033	1.76354451889057
O	0.104392151672485	−2.11934789089876	−0.54108985642006
O	−1.42715937059163	−3.58951126988086	0.753493592467257
P	−2.34703013637982	−2.28026745357805	1.30763593975833
P	−0.683010442137699	−3.47418115165479	−0.657320182622465
O	−1.85714199926939	−3.19771047410912	−1.69518307692261
O	0.116673094860713	−4.65714760336283	−0.980928443058346
O	−3.18496597257278	−2.77420842503055	2.40979600926051
O	−3.04250916368547	−1.76267343256956	0.0387725903890598
O	−1.7258837526326	0.296488412427882	−0.579127953101012
O	1.90829983769246	−0.842399206963203	1.51897949340637
H	−2.38571654324315	−0.473121294112393	−0.508844033605698
H	2.31387834403508	0.122133900238446	1.46130464057384
H	1.61620003701865	−0.960014045057718	2.42987812558614
H	−1.51374574839604	0.645896703189401	−1.46617303235841
H	−2.48053146089032	−2.62786503300393	−1.15674303581551
Re	0.0644794216394781	−0.412085217740594	0.374253182880508
O	0.124016049246026	1.31223882496839	1.14470411716275
O	1.41587505877515	0.307814801418322	−1.15168383331293
O	1.3189022527717	2.75439442399265	−0.490257895156568
P	1.32734910098823	2.37264934261845	1.12768541514836
P	1.02946555103735	1.6844289192479	−1.64727790606197
O	2.15415888431318	2.10942684520226	−2.73506792558671
O	−0.309297448152054	1.85766606064346	−2.27317391008942
O	1.06269858185894	3.61614678882323	1.86470013535071
O	2.57942535024179	1.57768995361643	1.43207777971749
H	1.66172303549542	2.51860801078299	−3.44205445753236
27			
converged			
O	−1.69578690461529	−0.913335016322762	1.23779911231436
O	−0.62650341133158	−1.82208141061957	−1.07930883620159
O	−1.63679760829911	−3.35551627249301	0.637972355827682
P	−1.66228842159437	−2.29668676602961	1.9973161995184
P	−0.609585924908555	−3.28345120374778	−0.555274009551746
O	−1.39880858341719	−4.12163153573929	−1.68622704849097
O	0.724781302391045	−3.86276858436011	−0.309357900503544
O	−0.34026513887848	−2.4946126940683	2.68991747387759
O	−2.92759450192639	−2.60495286940978	2.68344715230873
O	−1.8042778499304	0.92642670711824	−0.81913602264646
O	1.3197801582639	−1.50506226625488	1.07439622764018
H	−1.50955382719745	1.91030636583717	−0.579322473614294
H	1.42644317268067	−2.28778520348625	0.500195222431841
H	0.734049410208274	−1.8822275695201	1.84715046408314
H	−2.52550472442835	0.721702543291576	−0.207294098838562
H	−2.22307264826439	−3.66537593762532	−1.8515435816897
Re	−0.188232315960899	−0.239915107162909	0.056592486120304
O	0.338371072507968	1.26386062083659	1.0607800367998
O	1.22938322013907	0.36052414579591	−1.38421162396274
O	1.47139393248034	2.74234369670091	−0.56800663902458
P	0.356485682821474	2.82516351849776	0.639537951211132
P	1.38355978903236	1.80673721887218	−1.88701318974453
O	2.94297320388002	1.872573527006	−2.3240414031848
O	0.525188056286231	2.33406031013517	−2.94891814258047
O	−0.995268645081248	3.11800143171367	0.0245768127761007
O	0.895037951295649	3.710173788471	1.68054102657445
H	3.43549355154783	1.43802857272595	−1.62866754721012
27			
converged			
O	−1.61479664079059	−0.890536770320933	1.43969291576266
O	−0.583395258421262	−1.79713803128261	−0.909015392553513
O	−0.665355501790526	−3.21489474421114	1.18340989345158

P	−2.08324843106985	−2.36399365849846	1.69951648431117
P	−0.446101006283091	−3.27554184043369	−0.40432786634897
O	−1.69850059720264	−4.04108053660657	−0.951764989577198
O	0.878791693563412	−3.86440393097731	−0.690947989920502
O	−2.26500133246071	−2.69156793168257	3.12461403838963
O	−3.12437389720407	−2.78014956934809	0.690261258804586
O	−1.55959487179783	1.01651754768796	−0.755831177589166
O	1.44037599409068	−1.61629919895309	1.14772439730076
H	−1.13245848660977	1.27236600946848	−1.60981249121075
H	0.880044801898729	−2.13718875545177	1.75004553372405
H	1.62955158287609	−2.22640182814577	0.409618926741301
H	−1.45496145019929	1.88562624374021	−0.2114533799108
H	−2.43066412061202	−3.58705306422326	−0.381797334774524
Re	−0.127536346703947	−0.284642936550884	0.200395971476911
O	0.524320826983855	1.14684976096371	1.22874117945117
O	1.4010785002322	0.195163252558928	−1.20101565800752
O	1.4969904501388	2.63963171326919	−0.522694216825264
P	0.569191468566611	2.70945913686574	0.871958314016845
P	1.29477662955987	1.61218369369373	−1.72679729177837
O	2.65629032294497	1.87354497578753	−2.55896576698384
O	0.13343423466471	1.95698710549243	−2.59391690142106
O	−0.826159027250797	3.10230404951891	0.456938083607448
O	1.32243358325724	3.52739493065014	1.83154522733109
H	2.42426688261211	2.52736436069275	−3.21422176322816

27
converged

O	−1.60189095672255	−1.12353504526928	0.981999184435805
O	−0.209567813202432	−1.97338634374158	−1.15178250282746
O	−0.495696726166271	−3.42454188858475	0.846598219381707
P	−1.49627999266888	−2.44649855331712	1.83656416838384
P	−0.766080445481834	−3.37167396204686	−0.73237991607334
O	−2.35708557197834	−3.29613868379033	−0.831263150039319
O	−0.185082177093668	−4.51474088681701	−1.44491144320004
O	−0.651172764426616	−2.25481496139706	3.05603703481743
O	−2.82556388924961	−3.11492691703757	1.85575213649445
O	−1.44820031314989	0.674588358449443	−1.25851089556571
O	1.30063668760803	−1.41469183618637	1.49504583663838
H	−2.29574049348053	0.395078648261104	−0.887183553149333
H	0.624482289264127	−1.64742928769998	2.21493686047538
H	1.46782269427685	−2.26020632011081	1.05894703158613
H	−1.32079109172397	1.68975997118339	−0.959351615510524
H	−2.69415439261992	−3.1752531570077	0.0852949103732771
Re	−0.018880991537879	−0.347313076503203	−0.00992622088656318
O	0.181561559465024	1.20083444763191	1.04579121753276
O	1.56994700423127	0.340690209407499	−1.19613968243437
O	1.47735087911455	2.73825898223312	−0.401763027726546
P	0.16000501771202	2.7455162467739	0.591106845501647
P	1.62441018907153	1.78415214009132	−1.69355379066767
O	3.2025819667046	2.0192630236633	−2.00487564271188
O	0.838615655963848	2.23577671831745	−2.85405083686507
O	−1.07378764086559	2.91465949761598	−0.273790654641759
O	0.422066939723939	3.69793501923113	1.6769607225809
H	3.20989438022508	2.6231376509372	−2.74365122637078

27
converged

O	−1.73488391506806	−0.843364228216376	1.37710314159369
O	−0.806104984888679	−1.71289990766248	−1.02337564755241
O	−1.82702068244249	−3.26839615961795	0.685199232656422
P	−1.80304942409798	−2.25442492313297	2.07828317355453
P	−0.825864699852668	−3.16295979746462	−0.51535190693219
O	−1.60577615260375	−4.00999888446386	−1.65422213467672

O 0.50787167055712 -3.78910860601042 -0.304155490556754
O -0.499021721997514 -2.57049889085644 2.76863323450419
O -3.07621960780007 -2.51896056375286 2.76427189813145
O -1.61826135367357 1.11316854125248 -0.745518575716811
O 1.18327215591867 -1.6074812325994 1.18311188854234
H -1.19356075125032 1.36012960841384 -1.59925058848447
H 1.25965262793097 -2.37323503329385 0.577377646687109
H 0.599633924556332 -1.99196045822589 1.95563255021393
H -1.4835103802225 1.96205175558184 -0.191048601915183
H -1.01082909976179 -4.72176774177453 -1.87680287039271
Re -0.220074919901452 -0.252897784873506 0.215421908674589
O 0.523175035367464 1.14746177219068 1.24189288511739
O 1.27721688752883 0.194163346628828 -1.22761519197902
O 1.47132513475443 2.63226749870648 -0.535895144308025
P 0.587292955803405 2.71218529301614 0.890718080941607
P 1.22979503794292 1.61925688535713 -1.74286764720131
O 2.60552239170302 1.82444884615615 -2.57220154825622
O 0.0901167532315795 2.01877181351438 -2.6155759119015
O -0.805379560676278 3.15121796647547 0.520663481775841
O 1.39267686578906 3.50328725669419 1.83071242671501
H 2.42140580556268 2.53404362753644 -3.18324028499518
27
converged
O -1.73093883759791 -1.24178230734241 1.25312629804804
O -0.84898230374964 -1.6230055796013 -1.2434550016461
O -0.360042099112292 -3.28378655750949 0.530949167041543
P -1.26075250476054 -2.63803514810018 1.83061145850304
P -0.995765657412418 -3.15001550821001 -0.924252884834178
O -2.54771900098335 -3.41611092036982 -0.699351654190934
O -0.388437639635374 -4.043738967071 -1.91746052527256
O -0.228031005086171 -2.52563880631941 2.90524684936071
O -2.45848985825431 -3.51527243229266 1.97097938749818
O -2.00413074410185 0.92087074056161 -1.24921447776444
O 1.00985906140098 -0.88922916439701 1.27503936606031
H -1.65290356186519 1.88725093303585 -1.04200097729208
H 1.8497796956982 -1.03254569305703 0.812881333907566
H 0.720185285214227 -1.60279246158493 1.92250682236107
H -1.61702462183544 0.732899009403604 -2.11512482841352
H -2.70135969220679 -3.4090132967987 0.278836445496288
Re -0.560084713770188 -0.146277847761629 0.0005363208959248
O -0.234494475537113 1.39838919704804 1.00237987578495
O 2.84194889113734 0.347829498487793 -0.284285768448323
O 1.56155733670905 2.57660128284286 -0.211257172625707
P 0.0582676422824618 2.86855390022611 0.441282850917963
P 1.91268383157985 1.2379247610265 -1.0191313473283
O 2.70090948024364 1.85576379116927 -2.2995909878703
O 0.627192230133446 0.672011657402821 -1.58449645269904
O -0.910998321510518 3.10741586767379 -0.696115660950879
O 0.233823254648878 3.8996369630642 1.4728757912609
H 3.62334832607215 1.7065970933438 -2.10961422356053
27
converged
O -1.56399422381981 -0.810245932935548 1.27287764220259
O -0.945039224706285 -1.78655008219695 -1.2238676539419
O -1.75996101840725 -3.22370153946217 0.666697790599332
P -1.41222590942545 -2.20031086502222 1.98063017267532
P -1.00407474566694 -3.25099069416387 -0.711510928353957
O -2.15068607173267 -3.92764982716397 -1.64799505011959
O 0.210945257011474 -4.07681578406697 -0.788223421446657
O 0.0377892702021854 -2.46485427893961 2.33946637082522
O -2.45558551024402 -2.47296524223116 2.9808118180814

O	−2.01876865262718	0.980321837590788	−0.651418692960685
O	1.33981191592068	−1.54218047483908	0.474554461872462
H	−2.61165053572934	0.600432823125788	0.0162031358074743
H	0.8587811769984	−2.01289724386006	1.33691893681792
H	2.04165193394975	−0.960794771508465	0.788255666712592
H	−1.74237019043429	1.87990436325184	−0.264942608635613
H	−1.70402598003443	−4.64609226490276	−2.08860231530113
Re	−0.26424394039606	−0.300157584158296	−0.209473937062083
O	0.433660010535416	1.14219787883277	0.767595457809803
O	1.04834452979602	0.222303149341784	−1.79765647734751
O	0.538079417232525	2.62337994168894	−1.25469114263517
P	0.301016964311844	2.69721414513956	0.375974008783912
P	1.68135443022041	1.61507999960976	−1.82769602265421
O	2.79177837912819	1.67402049666782	−0.656707104166854
O	2.20965721358546	2.07293395054786	−3.11782247861298
O	−1.13207509275296	3.10593607524237	0.542568504026303
O	1.42812227692531	3.45034542874915	0.981320109370832
H	2.48310831786015	2.30663648465805	0.0186337671845501

27
converged

O	−1.59837615853465	−1.21040525772146	1.18580632212343
O	−0.844797283681477	−1.6440356972656	−1.35305011580839
O	−0.177637933929608	−3.18865494693452	0.48269471817056
P	−0.975061714799113	−2.51048584497816	1.82863006165464
P	−0.959885598513369	−3.13355611218036	−0.910069289790027
O	−2.49014371853123	−3.36403216761438	−0.507071601649151
O	−0.473451783206525	−4.10748768552722	−1.89325329188453
O	0.156988330331006	−2.20054030497992	2.75981951172911
O	−2.07163765862297	−3.45260918259361	2.17711516536187
O	−2.31885699874037	0.580236329286017	−1.47319597840523
O	1.16859207596892	−0.662040368728438	0.95173307319963
H	−2.44175474456561	−0.315567221468369	−1.82804796636859
H	1.69078638987952	−1.21864296615084	0.362035440484569
H	0.871505345122674	−1.23765667145168	1.75241419311122
H	−1.70383379251742	1.0286326574307	−2.08603184943838
H	−2.52849835782887	−3.38100544084658	0.476068437442825
Re	−0.606084004462748	−0.0453390474194589	−0.125469644949812
O	−0.442140132361175	1.58049691066608	0.75154916803822
O	0.275643395299606	0.800899498468833	−1.8246933266263
O	0.898814971303656	2.96731182541672	−0.748434586060078
P	0.682738833301277	2.73938433681214	0.882042331511251
P	1.46596851387739	1.78408887879195	−1.7021971406735
O	2.60747502774899	1.09683563035837	−0.843554717285968
O	1.9198869782349	2.32876165422567	−2.98824220248739
O	0.139151517045439	3.97627284822098	1.45958947957909
O	1.93912328343964	2.10742103598005	1.40496990012632
H	2.45488521644325	1.37621729919844	0.110743902550672

27
converged

O	−1.58123359491419	−1.14488868969756	1.06909300755959
O	−0.0245382084022427	−2.16228170561908	−1.03135933735869
O	−1.18657467429426	−3.58501240767645	0.662135528582366
P	−1.13243138450755	−2.40870052456274	1.89552605823277
P	−0.934704288566824	−3.4258398843144	−0.880278623675214
O	−2.35693376161052	−2.96483795766323	−1.51296994751138
O	−0.480672204478729	−4.66696616418851	−1.50560377142969
O	0.317204764739601	−2.28647219051642	2.28717923258911
O	−2.14157574182008	−2.82984750051358	2.88205375026744
O	−2.49457278261697	−0.0393509342121084	−1.02980359031306
O	1.50526971753369	−0.675698121896181	0.779770929341346
H	−2.71820296566931	−0.426680399236232	−0.15061255721121

H 1.13511186127037 −1.35029325335595 1.48971088493952
H 1.6405796332137 0.210055913257669 1.20893722610117
H −2.4772364468913 0.918123665515851 −0.890271530937138
H −2.46373127574193 −2.01173560527171 −1.50007192662656
Re −0.277764713964415 −0.393892727772517 −0.333224801241796
O −0.625277483323404 1.32863379909129 0.344202524991388
O 0.826549895347335 0.497156357627435 −1.87429925253479
O 0.84756275563725 2.8219487952168 −0.930116304488451
P 0.426857140636004 2.51336470308244 0.6307754951809
P 1.71109489687939 1.76056497769185 −1.81951177260822
O 2.98717147113512 1.49810807525363 −0.901049730649717
O 2.05762645555331 2.33997240956024 −3.11975969094696
O −0.220600985441875 3.70882542700664 1.1885185912585
O 1.62522368377721 1.8864650025777 1.31568468882724
H 2.67519823422174 1.58377895077754 0.0272449186093543
27
converged
O −1.70165631575583 −1.03995303422801 0.841834790794271
O −0.329094306295011 −1.87375128718556 −1.36202703214006
O −0.526189463968213 −3.28053502463675 0.688196694396021
P −1.45955687164038 −2.32461611815772 1.74202152623535
P −0.903694348696793 −3.23390502049755 −0.856106025855807
O −2.5049697299797 −3.05030888214318 −0.849158938526505
O −0.472677780310697 −4.41243777459775 −1.61496435759619
O −0.560501483502415 −2.05134121928988 2.89419568333781
O −2.76976814063335 −3.0326367761894 1.84719756097734
O −2.12111271789157 0.154623678841937 −1.54210904087344
O 1.21142413922102 −0.808678874674687 1.20035846677288
H −2.6999703054116 −0.170664665695835 −0.832348515409278
H 1.5532368895711 0.0824344975721639 1.59552531081626
H 0.681128670198041 −1.25666988112947 1.91363389158073
H −1.96679533533958 −0.603935491887795 −2.12568206359398
H −2.77074692442623 −3.05401870741507 0.102798750662353
Re −0.248051372642287 −0.197155867124391 −0.245695131881258
O −0.242658407760019 1.50197726005286 0.535078759158419
O 1.28867711695291 0.506554950304132 −1.54233130191972
O 1.80521527602862 2.63682499283367 −0.270499856312307
P 0.954272242181453 2.41540018054796 1.12780063259277
P 1.42120149833279 2.02822689321318 −1.71741008006671
O 2.86354093179435 2.18864479047048 −2.43497572864543
O 0.38424669702475 2.77867206196073 −2.42912351578303
O 0.502006009033686 3.71214771169535 1.64682328212983
O 1.80671228725412 1.51166630937591 1.98655404053152
H 3.44518174436196 1.56793529756356 −1.99768780243401
27
converged
O −1.75042869766883 −0.796354826417256 1.26008072398559
O −0.724614595981434 −1.74409773651901 −1.05966709050713
O −1.83809123272928 −3.24218264495673 0.637804249222041
P −1.81750484901693 −2.17982392490177 2.00743097689907
P −0.790070889694682 −3.18706674590018 −0.514913480439549
O −1.55787781456387 −4.04869167949523 −1.65146379234143
O 0.535930907642345 −3.81050882959224 −0.259956748130628
O −0.521966131462262 −2.47823328235102 2.71196571351868
O −3.10541828322819 −2.41341765259891 2.67983317436676
O −1.6808920860735 0.995558222960433 −0.756869260070226
O 1.23777937927484 −1.58302660867547 1.1455982994388
H −1.37267155189091 1.95548112672667 −0.469007427771042
H 1.30631650207688 −2.37433913791848 0.572622498047779
H 0.627887983584775 −1.92524192138933 1.90745219376409
H −2.43949538378976 0.784320001018776 −0.19233914398233

H	−0.862294655450647	−4.48193758625445	−2.14028008558179
Re	−0.168334877002531	−0.253708844672574	0.122671764280375
O	0.43875604718314	1.16689783265229	1.18651591789405
O	1.31970094583356	0.212981820981003	−1.26892730315161
O	1.59777019548788	2.59819491139787	−0.470045454106613
P	0.533461303694756	2.72333989632885	0.788766759703414
P	1.36632798343948	1.65747740582319	−1.75632359255543
O	2.84297161002228	1.78054998767359	−2.43055141205085
O	0.367752311029225	2.18622003437133	−2.70160584323239
O	−0.812875124864753	3.11333978758465	0.21951627244254
O	1.15991381736639	3.56716886601421	1.81521195843361
H	2.74736718678203	2.47160152810978	−3.08161986807578
27			
converged			
O	−1.93741127472715	−0.80504426187973	1.06455375882254
O	−0.679872557092091	−1.90857227128282	−1.1420196929396
O	−1.76210022257012	−3.26188170904278	0.672979841738356
P	−1.81971336007599	−2.1129766384872	1.9459801331045
P	−0.703920499640905	−3.31571990143436	−0.483297071885212
O	−1.50846516663009	−4.2111978412583	−1.58433690689075
O	0.575038487777803	−3.98573749272705	−0.196387828005698
O	−0.490072230766792	−2.18869549184301	2.63976312389473
O	−3.06265688965596	−2.41766709042991	2.67594917379916
O	−2.05896299430308	0.825844528628315	−1.33056629794531
O	1.08219940765482	−1.1278209592374	0.960504658555221
H	−2.56759467230309	1.20115822838771	−0.603090219268125
H	0.593385554399687	−1.58916081031641	1.7318943554763
H	1.58053278894894	−0.332466525516219	1.32540145151306
H	−1.47898597961331	1.56424117319594	−1.69075659877851
H	−0.979163439469573	−4.99957925547946	−1.67665393904013
Re	−0.465842753773607	−0.245275427982087	−0.176183282057779
O	−0.216898085967727	1.44126008137104	0.667760277173608
O	1.02595098696606	0.39952942302585	−1.56870374020985
O	1.69714557027153	2.56032142106734	−0.411195350441964
P	1.12283467303035	2.17766784740787	1.1366181825045
P	1.00975536780494	1.91954303382504	−1.69159660737638
O	2.13021827424548	2.25387416054246	−2.80786063762415
O	−0.276486559197133	2.56325064304061	−2.07089326635571
O	0.879841612862199	3.44527879702842	1.84098957059141
O	2.07549936379769	1.15420011496563	1.67810000908134
H	2.87514461689535	1.69012622930116	−2.60505311965483
27			
converged			
O	−0.69781459538681	−1.42785293566673	1.84284140882539
O	−0.0952285581841414	−2.01405586182004	−0.847757687387318
O	−1.24398868136611	−3.57822325910661	0.71583796649428
P	−1.92182412170505	−2.41116607574395	1.73425464011637
P	−1.02077760763497	−3.30120691234581	−0.83885331251072
O	−2.43784701299095	−2.81026582995328	−1.35059001780909
O	−0.491056938249927	−4.45567703473325	−1.56152541919056
O	−2.24560251675835	−3.07218434643801	3.00561974960537
O	−3.04453342891371	−1.77317295826826	0.922434677633974
O	−1.99723324040803	0.0605079869780209	−0.51877532881757
O	2.04903060472719	−0.861605025614279	0.757058722147562
H	−2.03426770841698	1.0655689403648	−0.479555235503929
H	2.33348652078988	−0.0749635077578439	1.24183209349927
H	2.42489838797163	−0.682557782551381	−0.208851524502385
H	−2.62128757352469	−0.40020911317952	0.11076109520662
H	−2.85322580951555	−2.36716707089637	−0.555928606939802
Re	−0.0354022866796225	−0.425032040962505	0.185401465659312
O	0.0042761314618593	1.26622108551629	1.08665487751777

O	2.61212966037443	−0.107778705620905	−1.54877813734367
O	0.620359683110374	1.40681676096158	−1.25197977780477
P	−0.0434018775241319	2.38329624429532	−0.0654465925848135
P	2.28843943440568	1.35823240221084	−1.47257897153455
O	2.79768761893817	1.84943733068397	−0.0123153644766913
O	2.66548208099148	2.28192735027673	−2.55328835640395
O	−1.48122356447687	2.58065259764672	−0.433965967151511
O	0.829374365196004	3.54385873357679	0.2325962570546
H	2.27895102617852	2.66109901714301	0.1667973504404
27			
converged			
O	−1.69210348029464	−1.30782616377959	1.20324379150776
O	−0.0832454970316371	−1.30678382399959	−0.921708234685381
O	−0.0601808698242913	−3.20261967880466	0.674543284698383
P	−1.28008803128014	−2.72975270826317	1.77390003084326
P	−0.300330758960828	−2.85978633950008	−0.870221518274418
O	−1.84937597957079	−3.13509296751694	−1.08689887446167
O	0.576022551757682	−3.61356628496517	−1.76845952390624
O	−0.521396231877517	−2.59616857144284	3.05794193461741
O	−2.40922515812576	−3.6709744535999	1.57995899182287
O	−1.78070311232413	1.0956536532175	−0.703582112911487
O	1.14095391126521	−1.00067563019847	1.84931948425662
H	−1.66653580964959	2.03749131842181	−0.384890422149014
H	1.58327772412718	−1.64060635370998	1.27721126328801
H	0.555242503492895	−1.57256385835931	2.46171567392628
H	−1.25656165644537	1.18584088755442	−1.60742011607238
H	−2.264628567098	−3.25515416293216	−0.208621203844791
Re	−0.35907476819721	0.0412826451042359	0.50791681877743
O	−0.134633555877011	1.51052036969319	1.59770046492166
O	−0.249328341883092	1.51955397244647	−2.62178041314339
O	1.08805937881977	1.58857220819175	−0.432916904578658
P	0.458540465762838	2.71414257991942	0.627677632424803
P	1.16582280081103	1.6322401298269	−2.11791186304984
O	1.88866416360171	0.200926737622691	−2.29629631730056
O	2.04360153728226	2.7194031498162	−2.56193003510214
O	−0.748993326465266	3.36819551800715	0.0341016105996066
O	1.52675155664728	3.4689495154978	1.2895184565255
H	1.26886854903855	−0.496701678085269	−2.05021190507359
27			
converged			
O	−1.61762239280128	−0.875008034842701	1.35952624089612
O	−0.628734585721558	−1.86851296968565	−0.998914969193846
O	−1.99829254999406	−3.23326374530027	0.604197634171467
P	−1.70188516277509	−2.30622221824553	2.00709335087368
P	−0.984426979846933	−3.32674126688038	−0.589963565056953
O	−1.99760262347501	−3.82284739220681	−1.76319776616222
O	0.123636440504262	−4.28913883523318	−0.478844863531558
O	−0.341934196663623	−2.74658220383435	2.50575612858268
O	−2.88471109141364	−2.49804911593816	2.86051812070265
O	−1.68027832004776	0.896131718014136	−0.79714352586741
O	1.36799982833681	−1.54656459220465	1.1751520106002
H	−1.37234514995687	1.85909866707047	−0.529421376181614
H	1.7480004671011	−0.919131022000523	1.80003442237374
H	0.697853446536641	−2.15182015753588	1.75603611661628
H	−2.42156920426998	0.690082856303495	−0.209494968350887
H	−1.47188324693657	−4.41469305444938	−2.29560682003611
Re	−0.115748023177381	−0.332054712933973	0.0753635819759664
O	0.452411301608022	1.13578868436559	1.1189607960353
O	1.33864868639126	0.255856537623136	−1.35224509112478
O	1.62048535939453	2.61643439083008	−0.488860436815127
P	0.514064572591949	2.7027548420229	0.732994069019506

P 1.43248940859334 1.70828369961273 −1.80848312104802
O 2.94136446992775 1.82668170596878 −2.40785800693048
O 0.4895310730054 2.27505638348111 −2.78854062598754
O −0.826510642882001 3.05545455017662 0.132547162940528
O 1.09352431667591 3.53821908506386 1.79464854840617
H 2.86293480757946 2.46528621092096 −3.11235305325169
27
converged
O −1.65481607945154 −1.18403967513532 1.04947241691086
O −0.685527117106367 −1.42747357871032 −1.47475160395056
O −0.193562936539896 −3.11911539252839 0.277635331060077
P −1.01019316552504 −2.50440509361297 1.6325037073718
P −0.910231716264477 −2.92147184696931 −1.13421442148249
O −2.47960010228261 −3.07978250481505 −0.785025971573973
O −0.465601536270719 −3.87621105281074 −2.15618509054644
O 0.107709337137905 −2.22656759245835 2.58935483330384
O −2.10975666884129 −3.46731686192865 1.91558573752607
O −2.66747293274491 0.286302332419921 −1.10648300987786
O 1.12828078984593 −0.39786295912694 1.06848948963091
H −3.0959375787248 −0.288867831121784 −0.451561936689912
H 0.842774239203324 −1.14521302106477 1.71155010775595
H 1.85923148054241 −0.666867548784828 0.493947134475494
H −2.40267106820301 −0.300031701972485 −1.83541663689587
H −2.52539648031738 −3.25177439171735 0.18053574389471
Re −0.606064191009061 0.116442569158036 −0.104636434871247
O −0.639112779964282 1.71047193533639 0.818189151615941
O 0.50292136881024 1.07242416317243 −1.60262138679933
O 1.77987551168169 2.20865630890303 0.264432326676373
P 0.392065830754893 2.98001199046133 0.791517476999054
P 1.87114447217294 1.61872397414754 −1.21688817542174
O 2.82280545344576 0.299909596928438 −0.914997626068471
O 2.58972328083433 2.46481583304295 −2.18196634240611
O −0.112355856503336 3.84805391684667 −0.297813733974231
O 0.667122547602157 3.46808948182238 2.1551531707103
H 3.63404590071004 0.477598939513485 −1.38390426371708
27
Coordinates from ORCA−job Tc_Felprot_d E −82.220840887340
O −0.96330790802709 −1.29639868889926 1.60077721685892
O −0.00573188040220 −2.04432998300951 −0.83607463461276
O −1.15975457730267 −3.61303432925711 0.69575798263315
P −2.04192081970050 −2.41214502880235 1.48943436853558
P −0.74312491990712 −3.43247281499674 −0.83237400883507
O −2.12406224497028 −3.21099210397199 −1.58704797013756
O 0.03939578174560 −4.55415908932933 −1.35172951181254
O −2.49845418287936 −2.97710717356614 2.76535727895921
O −3.11818419588976 −1.99956843362313 0.46467431883341
O −2.23690161497081 0.01780311567867 −0.70444849557785
O 1.87452943042359 −0.59528976333204 0.73327928385632
H −2.71814883414524 −0.78103350228287 −0.23431186287199
H 2.42673372960393 −0.55126595400489 −0.06113840631737
H 1.98056406989254 0.31572249947318 1.16633106985109
H −2.48031327271932 0.81190373783265 −0.21634800343047
H −2.69580273388914 −2.74813196938316 −0.91817316809929
Tc −0.17387171121348 −0.36921081590731 −0.02044802016878
O −0.32847255577354 1.30849411396961 0.73647420363162
O 0.65817950707769 0.55828967692409 −1.69427110115277
O 0.91318893032351 2.86562252421161 −0.71388637621903
P 0.71934992162959 2.50719579957421 0.87959186665302
P 1.55581276428106 1.79645028781577 −1.76013183651775
O 2.96171354942475 1.44078444408755 −1.04260182460189
O 1.74919021883154 2.40605726261889 −3.07723917525058

O	0.18226721683195	3.68446924072852	1.57064431637139
O	2.03399706157322	1.88592252740650	1.34461393053987
H	2.83252927314447	1.68092440903990	−0.09481145275359

27

Coordinates from ORCA–job Tc_Felprot_d_job10 E −82.215936343920

O	−1.57407529687269	−1.17087181621753	1.08443810339545
O	−0.13398091830695	−1.96964546330381	−0.99054321851802
O	−0.64141543716269	−3.50234729880156	0.90720023082622
P	−1.38569012617768	−2.43342799777504	2.01904718269596
P	−0.86020210568311	−3.31120855256767	−0.65980430781481
O	−2.41553513803603	−2.98766834407009	−0.82468352268717
O	−0.43150298587684	−4.47889968949427	−1.43226227537204
O	−0.28402398251839	−2.21130761259057	3.02810865072535
O	−2.67704590132483	−3.00928400151938	2.42999929853709
O	−1.47547243343483	0.60798776570933	−1.24541267833863
O	1.39738308137497	−1.36344140559913	1.39581751094323
H	−1.60167967317612	1.51396784193307	−0.78252788996292
H	0.76911152268939	−1.69130378607958	2.18714847944634
H	1.63626994244308	−2.17105398590347	0.92787017215342
H	−0.98371076366892	0.85737401873638	−2.05631068621019
H	−2.62965521388961	−2.32530103836966	−0.15380982099894
Tc	−0.04908099546259	−0.36779616473626	0.07148345847258
O	0.17790952391267	1.19044490428519	1.00090836928514
O	1.50399701473306	0.27207546224927	−1.15704222245393
O	1.16655070270946	2.72395519197515	−0.68462275359793
P	0.03129054175308	2.72368957001645	0.54053473480868
P	1.33822585363227	1.62894999377634	−1.84136987206609
O	2.81958355444213	2.01319064789053	−2.35250997984852
O	0.35743670560769	1.74891164152731	−2.93820120861234
O	−1.32693176316404	2.85616551650586	−0.09409397111996
O	0.50109225844956	3.69277126447022	1.53880477004249
H	3.41055203070909	1.85857333224005	−1.61626654949087

27

Coordinates from ORCA–job Tc_Felprot_d_job11 E −82.216947737580

O	−0.84840142593065	−1.26431387791625	1.67311866521261
O	0.26999976846319	−2.26225986381625	−0.59549358458154
O	−1.34821182437278	−3.52256422407594	0.79499477863638
P	−2.11504558218442	−2.16834036776715	1.47847376326259
P	−0.75796666956112	−3.45489521142340	−0.68380007229479
O	−1.98274862557656	−2.96611071432595	−1.56363215288224
O	−0.18162067779705	−4.72600827174395	−1.12877002866774
O	−2.71807778066206	−2.61779612636058	2.74260592228776
O	−3.00952368055557	−1.61628024213493	0.37683455963775
O	−1.55665419985240	−0.13856357454786	−1.13976748959891
O	1.82796566128613	−0.81959075591677	1.43728028288937
H	−1.50773033280490	0.81420075401366	−1.37176677330856
H	2.12168412574622	0.18634592525334	1.58820348212728
H	1.39724403366690	−1.09740086665175	2.25652060556538
H	−2.32464586440975	−0.38672298904576	−0.56328120352689
H	−2.52434021918096	−2.40575628265158	−0.93635965039309
Tc	0.15492945771998	−0.53372162846740	0.10297182978395
O	0.01044349404769	1.20623915034195	0.73216591938416
O	1.23692810870137	0.32275561783145	−1.50618841332900
O	1.61022073309687	2.60521163125239	−0.49790406522632
P	1.11254398202019	2.30954358170525	1.06517182918907
P	0.90031143659350	1.79853033445989	−1.69376267569946
O	1.84056662756596	2.26890245206013	−2.92048444543439
O	−0.50247392989638	2.19428852350409	−1.93334425347783
O	0.58975722239704	3.55094800524375	1.64928131356541
O	2.27055989885993	1.60277734994035	1.74258636850176
H	2.67368627090427	1.81508166552636	−2.80375450209134

27
Coordinates from ORCA-job Tc_Felprot_d_job12 E -82.215477743900

O	-1.54594763044056	-1.17653121592747	1.09300776967417
O	-0.49093125153440	-1.85506979822271	-1.23050398564110
O	-0.48454477678528	-3.45789713480448	0.66512026667251
P	-1.36631208754373	-2.56146210875745	1.81542536126477
P	-0.92344927743739	-3.31523363358630	-0.85836752813003
O	-2.51393152975585	-3.29530680289204	-0.79243810085729
O	-0.38781054065118	-4.36483579063654	-1.73018323989799
O	-0.40158199145082	-2.47001601197323	2.96105870784596
O	-2.68793689776004	-3.23458631046455	1.93320233873865
O	-1.51899033163275	0.79987016204128	-1.18498189036425
O	1.33419060635106	-1.38759815640388	1.36339524936580
H	-1.05451536089935	1.11271804235447	-1.99063143597446
H	0.72984468451627	-1.88488950469086	2.01903623000770
H	1.62276509352393	-0.60410201113011	1.84771530452383
H	-1.54673695758346	1.64962611088655	-0.61348201180275
H	-2.76439518382586	-3.18234835223201	0.15254377684526
Tc	-0.12377677727564	-0.41622823656454	-0.05958894483688
O	0.31099089664149	1.02717359465578	1.00784584695177
O	1.38745193718717	0.27958535025676	-1.32576292178489
O	1.32467703065502	2.67206155310600	-0.55827556106978
P	0.24915038630677	2.60550897474393	0.71917026393471
P	1.30824538124701	1.71455472940365	-1.84416538862010
O	2.78144544162857	2.02181420047064	-2.42738310762604
O	0.27145963772580	2.05346400472166	-2.83875417473331
O	-1.12266857913202	2.90307538629462	0.18246062787919
O	0.84635628636291	3.40503208356878	1.79895568245542
H	3.40635178926347	1.65612087536096	-1.80251911999773

27
Coordinates from ORCA-job Tc_Felprot_d_job13 E -82.215937625650

O	-1.57392141928058	-1.19235081578099	1.06937525091342
O	-0.12009864029624	-1.96166746839136	-1.00633226291448
O	-0.61366005544143	-3.51250733469878	0.88137126919036
P	-1.37746430406398	-2.45938760773537	1.99598584383706
P	-0.82873561926683	-3.31496768027501	-0.68571928125510
O	-2.38773636000886	-3.01187282300563	-0.85394874108680
O	-0.38082378614922	-4.47191539647089	-1.46327023982090
O	-0.28451466595982	-2.23285747666969	3.01355833123769
O	-2.66500442484445	-3.05245409450948	2.39390366426682
O	-1.47812425789931	0.60979659322847	-1.24586115043571
O	1.39593668025966	-1.36288858664791	1.39219108175578
H	-1.61376991998138	1.51123430864967	-0.77584262948912
H	1.64183667374337	-2.16631741681495	0.92070331209554
H	0.76743691192815	-1.70029079686847	2.17972493678174
H	-0.98522833442451	0.86988476071384	-2.05243874328811
H	-2.61395300643690	-2.35851581934612	-0.17838018446377
Tc	-0.05177187486923	-0.36759962767217	0.06878114114091
O	0.15768250920360	1.18377032042728	1.01361923012529
O	1.50134146498239	0.29599222194808	-1.14588138779179
O	1.14561325178396	2.74076060120937	-0.65173932895972
P	0.00261907265130	2.71996994241427	0.56669422189086
P	1.33021089762363	1.65814212425152	-1.81834678334381
O	2.81145999288489	2.05666072327380	-2.31797891818744
O	0.35397063062077	1.78252393690259	-2.91859432577471
O	-1.35245293822118	2.84781291274115	-0.07580330089290
O	0.45854324351175	3.68359423017222	1.57638761666429
H	3.40000829152689	1.89995023678285	-1.58025860208050

27
Coordinates from ORCA-job Tc_Felprot_d_job14 E -82.215478504890

O	-1.54748417859442	-1.16033011527061	1.09834019660008

O −0.49589272342022 −1.86431131573278 −1.21974366271262
O −0.50107589832507 −3.45182257313260 0.68989438467314
P −1.37346585212959 −2.53892661028861 1.83461355800886
P −0.94159526966487 −3.31696457295646 −0.83448119705072
O −2.53185204694471 −3.28197514637301 −0.76515196771075
O −0.41789848359492 −4.37900809281518 −1.69833200545651
O −0.40390125583499 −2.44128455614174 2.97574355188013
O −2.69857016703131 −3.20254866290605 1.96432260937837
O −1.51583235816325 0.79406997057307 −1.19076144865113
O 1.33502022190706 −1.38474401142040 1.36794305157890
H −1.53957869156952 1.64830028183102 −0.62641849732569
H 0.72904503694512 −1.87257286996450 2.02967232994627
H 1.62857422329600 −0.59843411060436 1.84448493222278
H −1.05256198596141 1.09859383244257 −2.00007278287477
H −2.77933894609405 −3.15961724837822 0.17890426880217
Tc −0.12165039971626 −0.41765832522556 −0.05954064853610
O 0.32013477722607 1.03172686784463 0.99679393344621
O 1.39248636288258 0.26169189578982 −1.33123086081430
O 1.33760185426632 2.66131458814402 −0.58425275577297
P 0.26336990928354 2.60778988001984 0.69606738921130
P 1.31579581745022 1.69254967817835 −1.86178255481394
O 2.78860485461565 1.99087842266757 −2.45091811655446
O 0.27748163900374 2.02609120298693 −2.85648817873424
O −1.10774320869475 2.90628769450100 0.15823028772886
O 0.86530050871016 3.41305203415778 1.76890880084411
H 3.41442627372932 1.63235187752764 −1.82284461307337
27
Coordinates from ORCA–job Tc_Felprot_d_job15 E −82.214904340910
O −1.53096687911453 −0.83439294532288 1.36392372712701
O −0.72767304634414 −1.82079513041149 −0.99796518781380
O −0.69284749266178 −3.18511037738358 1.11422927291733
P −2.05685374588865 −2.28697758846014 1.62973186421059
P −0.53171877003381 −3.28256121408372 −0.46213814486589
O −1.78408820225076 −4.08362882095247 −0.95913382881465
O 0.79792950011393 −3.84167754393839 −0.79737878885715
O −2.31346141934120 −2.59916142781881 3.04364326026165
O −3.10959483508973 −2.61181658385716 0.59022538109721
O −1.93485665755680 0.88245022469762 −0.54605665007380
O 1.65306015726238 −1.50999224136873 0.44318885439334
H −1.71508112006819 1.81390058356506 −0.18774108740576
H 2.22551879952956 −1.09919762728208 −0.22662533693408
H 1.45152131097673 −2.41502080342733 0.10687921806913
H −2.56953571646787 0.49771191661440 0.08226049528725
H −2.49851128710379 −3.57786259015917 −0.41695174118881
Tc −0.18686799423757 −0.30699311284803 −0.03847049723917
O 0.46163799583353 1.17638043558034 0.83449434730749
O 1.19441550725847 0.16678562621302 −1.56159422187973
O 0.56486256726312 2.57980934131897 −1.23355201428792
P 0.30720144517331 2.71225230420959 0.39325612895514
P 1.74135690400060 1.58802099112768 −1.74304412155331
O 2.87476496315246 1.78911714049030 −0.61342161228371
O 2.22682318968204 1.93938057574927 −3.08118374501421
O −1.13960212167175 3.08556408546767 0.52628078871329
O 1.40620667123011 3.51971629825147 0.97539336783628
H 2.52576025818015 2.39859849419102 0.06365026569234
27
Coordinates from ORCA–job Tc_Felprot_d_job16 E −82.213558115150
O −1.50339540035064 −1.03040858910644 1.48393182697195
O −1.00933867492936 −1.63454233756878 −1.04904562322289
O −1.27815878424617 −3.40326214152373 0.71459695100016
P −1.12861736597043 −2.41757115034842 2.11387973984487

P	−0.65491884200584	−3.10185943284456	−0.70010332422835
O	−1.61902071757926	−3.98362286086293	−1.64533375025573
O	0.75789457320450	−3.47885948876055	−0.88214702460373
O	0.34059365170204	−2.46919447823205	2.47657311708778
O	−2.12769504926922	−2.94389577331433	3.05412788795667
O	−2.14748155985839	1.02935386031205	−0.73134292658479
O	1.34123969348163	−1.19989777168246	0.61293520570394
H	−1.88566784409721	1.09313711086023	−1.65998151716892
H	1.45543781032128	−1.92032953605637	−0.03524778631158
H	1.01970602062126	−1.73468990543867	1.49773741816514
H	−1.89313014358364	1.98970105749676	−0.35017566571964
H	−2.51992984497066	−3.72310064234869	−1.45555245052975
Tc	−0.48185189139975	−0.12690616470634	0.02534034994046
O	0.21700051039775	1.33514431631140	0.85788749802397
O	0.37210147914329	0.63144301487623	−1.74833992628935
O	1.05482478448109	2.89288304729617	−0.85653736813375
P	0.15212138476504	2.90978310059550	0.51002522540546
P	1.55652913263908	1.60226575743153	−1.70056399400768
O	2.69640292734792	0.95122192118453	−0.77624572763444
O	2.09906168276674	2.05633609911086	−2.98377766002268
O	−1.27802313736519	3.19574078359752	0.09009045488566
O	0.79175988061385	3.76595157100819	1.51620336683742
H	2.31195570596611	0.40967862700045	−0.07703429287059

27

Coordinates from ORCA−job Tc_Felprot_d_job17 E −82.213380681990

O	−1.53617385199212	−0.87544807223751	1.25197910446982
O	−0.83054044899306	−1.74414163257151	−1.15896704282635
O	−1.49820055769842	−3.30768012512344	0.66913041697957
P	−1.31605221884720	−2.23813068587687	2.00846804625297
P	−0.67267399910536	−3.20665499166628	−0.66685175811343
O	−1.60381038554600	−4.06626906710922	−1.66471352712854
O	0.70111069748667	−3.73578908109030	−0.64210471181902
O	0.11416847206155	−2.38642181964849	2.45016858469520
O	−2.42721579326884	−2.58006543238894	2.91035847056405
O	−2.09038657998487	0.88677484180484	−0.69133283404253
O	1.42127456985262	−1.33693208749521	0.58281604916460
H	−1.83086712643743	1.87223031708053	−0.41889049411951
H	1.53711937488116	−2.10723533449001	−0.00186948674133
H	1.00216058292227	−1.75638198899974	1.43791806893565
H	−2.68080255002987	0.58950070812538	0.01497514054984
H	−2.46867411619566	−3.65789515056781	−1.68112574934264
Tc	−0.29227554099773	−0.21024355257981	−0.15718334921680
O	0.34681895710523	1.27878757896180	0.69581967154224
O	0.88723361235935	0.35355080607922	−1.80496990834369
O	0.75432274507955	2.80262800943433	−1.22315703153312
P	0.17167477773361	2.84463826527433	0.30750506043459
P	1.72908464133340	1.62869345874351	−1.79063934063002
O	2.82581880695716	1.47859580619290	−0.61883677506915
O	2.35150661330157	2.04062122415790	−3.04978054452571
O	−1.31483877844465	3.08987160133259	0.16629689200934
O	0.98773117787693	3.71735564044462	1.16338624368472
H	2.37188691629125	1.32054076908385	0.21350080840886

27

Coordinates from ORCA−job Tc_Felprot_d_job18 E −82.213938646760

O	−1.36372288653389	−0.85775072783293	1.35313558576527
O	−0.68768150394130	−1.77597425041675	−1.07215549542654
O	−0.53766976166317	−3.20127793972022	0.98769521986517
P	−1.86767477121207	−2.31032366879166	1.63280227207397
P	−0.49543596340286	−3.26223933875099	−0.60987940356947
O	−1.80398593054466	−4.01586986496861	−1.02155403173903
O	0.79005001724453	−3.84312056677496	−1.04839587993868

O	−1.96296880295626	−2.65794869035313	3.05997788492185
O	−3.00858567628621	−2.63777255431417	0.69625172976108
O	−1.87927253192941	0.88721845188634	−0.55942053884117
O	1.60212979907471	−1.58309030315845	0.52885648331481
H	−2.47988277013279	0.45097519474747	0.06833288865069
H	1.19282121864235	−2.03930453941861	1.28073626511405
H	1.64574798703897	−2.26237359516629	−0.17495535696788
H	−1.68570385998817	1.80815693063996	−0.15316813099477
H	−2.46658966465831	−3.53152028126070	−0.39590253960001
Tc	−0.10044101010145	−0.27304894659094	−0.12237115475767
O	0.52075701750025	1.18706277947467	0.79567600569032
O	1.16405548237392	0.28536420869639	−1.67612960671112
O	0.47074107240743	2.65878471424732	−1.23708411241261
P	0.30775345239603	2.73149798749490	0.39860504191898
P	1.64936658943170	1.72494944327041	−1.86657955532233
O	2.85349970518310	1.97539016969093	−0.83042122170411
O	2.00235657283226	2.13261258215856	−3.22985955816589
O	−1.14327601107384	3.05047662765823	0.62133912484417
O	1.40631474440202	3.55392604159008	0.95862553383455
H	2.51669748359825	2.49970013025027	−0.08225745065580

27

Coordinates from ORCA−job Tc_Felprot_d_job19 E −82.213384735930

O	−1.53721489650859	−0.87556391623697	1.25074969256588
O	−0.83358270090236	−1.74308602357129	−1.16119252272358
O	−1.49746152499358	−3.30741529695911	0.66748879083118
P	−1.31306966207892	−2.23763261823536	2.00657366425177
P	−0.67470204302056	−3.20603526863469	−0.66990099986054
O	−1.60754572124428	−4.06633548080581	−1.66586933582747
O	0.69962739777105	−3.73432558974963	−0.64920007897464
O	0.11871501644087	−2.38432196409782	2.44443196796304
O	−2.42042617613080	−2.58233008640705	2.91224803695409
O	−2.09261788437412	0.88837359683400	−0.69018937996194
O	1.42039949176680	−1.33480036109358	0.57393124436157
H	−1.83174103925287	1.87478349506603	−0.42009541273181
H	1.53571980085729	−2.10559488813221	−0.01040417394254
H	1.00328756988069	−1.75526895684801	1.42998735851229
H	−2.68131124142581	0.59216612253628	0.01795519733072
H	−2.47368730748835	−3.66045855468754	−1.67731248663998
Tc	−0.29485251707775	−0.20974337638235	−0.16086793524347
O	0.34395185098381	1.27884634676130	0.69493305795505
O	0.88322646376079	0.35916680034137	−1.80734416166854
O	0.76407282125935	2.80696127208391	−1.21707279548209
P	0.17161745970784	2.84550471672306	0.30976220800600
P	1.73415610357301	1.62861421050556	−1.78373843310517
O	2.82466750614888	1.46686961018653	−0.60828764874849
O	2.36484139625879	2.04181312891860	−3.03847110360997
O	−1.31426114933649	3.09205107374413	0.16038610992554
O	0.98153731494728	3.71730429084972	1.17257513913926
H	2.36605366288738	1.30495772216161	0.22082401025522

27

Coordinates from ORCA−job Tc_Felprot_d_job20 E −82.212561820790

O	−1.59164390761989	−1.21671488846992	1.06173293168945
O	−0.04341466917990	−1.97701015968932	−0.94468807135636
O	−0.66315466080544	−3.55095785300477	0.88305048089582
P	−1.45425629085469	−2.50174465426327	1.97879479557017
P	−0.79375243552911	−3.32152732319762	−0.68824020774096
O	−2.33617397397787	−2.98328368636885	−0.93085976292816
O	−0.33172369720260	−4.47315743015834	−1.46559469588505
O	−0.40467146312667	−2.30747258569851	3.04582846093407
O	−2.76899521553387	−3.07619435850186	2.31056319393975
O	−1.39555460780542	0.58311087084648	−1.22320023320689

O 1.35677757439587 −1.38814813868704 1.53380140927773
H −0.86100611466751 0.85705477728112 −2.00242384575943
H 1.66805627838857 −2.16858844147325 1.06297565139837
H 0.69449282365000 −1.75482735815814 2.27287388230937
H −1.55050259388774 1.48026716964584 −0.75689138062701
H −2.58197450735755 −2.33198102537673 −0.25998994738755
Tc −0.01836170058912 −0.39185380621547 0.14705609430242
O 0.15023026036249 1.15731453396010 1.10268584714456
O 1.61295442602059 0.25593189677005 −0.99308844612164
O 1.22493018512204 2.71020277045346 −0.51698680535575
P 0.02721906801024 2.69385904831670 0.65071386496521
P 1.43891581070462 1.60942186834528 −1.65160363636213
O 2.90692929490122 2.02723421478926 −2.18503074007683
O 0.47224080848778 1.73325473647845 −2.77683972126230
O −1.29359355335366 2.82919877474829 −0.05951945053667
O 0.43008123993701 3.66014322974721 1.67965639476020
H 2.74535161921174 2.54096782275151 −2.97287606892434
27
Coordinates from ORCA–job Tc_Felprot_d_job21 E −82.212655913340
O −1.57194390316832 −1.15973500432945 1.09839151872858
O −0.43889353969022 −1.90278519589067 −1.16413673389673
O −0.55492499889415 −3.47529202147509 0.75598137627487
P −1.44746796167398 −2.53103155343631 1.85978939565567
P −0.93124710926688 −3.34252649924933 −0.78412737372117
O −2.52257792061673 −3.26979835256525 −0.77770396637718
O −0.40229695986060 −4.42313475992847 −1.62152315681125
O −0.51625334347384 −2.43395909569072 3.03211946067025
O −2.78850080511475 −3.16828497257495 1.95189219741107
O −1.42367575993282 0.76645394420643 −1.19574069751173
O 1.29661226230527 −1.44579115250334 1.45662277523729
H −1.46700644966528 1.61907236365200 −0.63396579785174
H 1.64244048252502 −0.67263203892337 1.91869637648657
H 0.66370688757285 −1.89398249889701 2.12015913591018
H −0.91507827495537 1.07449011806519 −1.98013390232097
H −2.80355104031344 −3.13146504793912 0.15406202174926
Tc −0.08355926192656 −0.45231022036709 −0.00445856731553
O 0.32546513375293 0.99966891098254 1.06105455365107
O 1.51196543820167 0.18732812744519 −1.20942621353495
O 1.42318402462435 2.60580933033063 −0.49419641151402
P 0.30241163545324 2.57248860048717 0.74978304111931
P 1.44255270974941 1.60643780062420 −1.73654782376080
O 2.90874411883968 1.93230950031147 −2.33751781383721
O 0.42491535960866 1.92774564083998 −2.77445279902548
O −1.04106208554940 2.88692362940074 0.15242109305447
O 0.86509703774079 3.38575485248114 1.83709763302485
H 2.74034433730494 2.43274558393886 −3.13223931725504
27
Coordinates from ORCA–job Tc_Felprot_d_job22 E −82.213166343850
O −0.88099832329212 −1.24919066521932 1.73472529651949
O −0.06738633886306 −1.90953798568310 −0.74369756999826
O −1.16062593781260 −3.53129828916434 0.78535624955688
P −1.99976668987607 −2.32565928494993 1.62512306910693
P −0.78379353211532 −3.29739443402545 −0.74661630143347
O −2.19285944284871 −3.07169396124176 −1.45947417538219
O 0.00431543012243 −4.39298268093122 −1.31557201469828
O −2.44749781278288 −2.91294478588252 2.89267300542272
O −3.07749296028713 −1.87449109711428 0.61708777719918
O −2.18662759445413 0.11677887314296 −0.58849481669940
O 1.92100100198738 −0.77621562456106 0.68122828473566
H −2.14745603704711 −0.12871619992457 −1.51982884072772
H 1.97708535242242 −1.61581656291896 0.20715365303922

H	2.42832501936637	-0.10650680364739	0.15753732104253
H	-2.68679293725973	-0.64667860832448	-0.07922899107835
H	-2.73705885391011	-2.64786712348088	-0.73660588260160
Tc	-0.17767888827721	-0.21814967385701	0.14720482485052
O	-0.20784050343030	1.47051170829066	0.83167571883520
O	0.56220408902258	0.56206258504299	-1.63296208543960
O	0.85487904072525	2.92241758636582	-0.83530874631615
P	0.67714231964942	2.81802351176588	0.80728415550123
P	1.56458879298692	1.72405955735876	-1.66187357035690
O	2.79960207695611	1.31531261130959	-0.70568203576331
O	1.98784567789448	2.18020348319568	-2.98938833067302
O	-0.04966115453446	3.99573277997300	1.29374824341938
O	2.04576374643359	2.47555939456611	1.34982999578675
H	2.62018446750869	1.81898168420193	0.18600576510039

27

Coordinates from ORCA-job Tc_Felprot_d_job23 E -82.212554812980

O	-1.59079755395282	-1.21618571366726	1.06082908514167
O	-0.03560155830131	-1.98253661627688	-0.94051578320628
O	-0.67442747407219	-3.55492360384352	0.88101457331252
P	-1.45546516890719	-2.50066749346157	1.97873046690474
P	-0.79742121125134	-3.32162164606914	-0.68977511799031
O	-2.33606059974087	-2.96885378221875	-0.93672773758176
O	-0.34170044890232	-4.47480158072118	-1.46818679601988
O	-0.40046006514942	-2.30920248251415	3.04105188390883
O	-2.77080296369565	-3.06928589262328	2.31690967419303
O	-1.39405431740828	0.57441200824612	-1.22756282001596
O	1.36274528804029	-1.38718713656087	1.53590378053407
H	-0.85778035552985	0.84932458597340	-2.00516129333389
H	1.68071567729294	-2.16500910440776	1.06554128740088
H	0.69765946276669	-1.75795044507588	2.27206320231476
H	-1.55290872100158	1.47077912020436	-0.76162967396943
H	-2.57594225232720	-2.31245753126200	-0.26844886480917
Tc	-0.01394576025593	-0.39514369621355	0.14669762985544
O	0.14562721562925	1.15505764511427	1.10132033760262
O	1.62042411462444	0.25539662745643	-0.98826052601132
O	1.22064821689909	2.70929789106362	-0.51616268877481
P	0.01898923601956	2.69070335825013	0.64840172721343
P	1.44110328569304	1.60714112181634	-1.64861456182518
O	2.90906326018196	2.02892629434600	-2.17883964867618
O	0.47508204549487	1.72647065992236	-2.77455910736192
O	-1.29955612039215	2.82138869554725	-0.06629051666247
O	0.41690797658267	3.65865675641325	1.67739512813179
H	2.74735879465618	2.56277196014111	-2.95322365191091

27

Coordinates from ORCA-job Tc_Felprot_d_job24 E -82.213099871880

O	-1.66365845892110	-0.93436418953464	1.33482422193885
O	-0.65762257131568	-1.74105713573088	-0.97373685914970
O	-1.60213482748784	-3.36095547031335	0.69852413587153
P	-1.65660595393832	-2.32419785258458	2.07330609971364
P	-0.62767210624555	-3.22260487856739	-0.52909698207257
O	-1.46203456551924	-4.00176513483929	-1.67013029515929
O	0.71472244429084	-3.81353970823040	-0.36439447154491
O	-0.34850301155845	-2.55202531917557	2.79013199942138
O	-2.92781168923079	-2.65284936526612	2.73594512233995
O	-1.65665145248056	1.01112648165491	-0.84328848833652
O	1.25376516108934	-1.53463125046432	1.16632210469340
H	-1.24767236234770	1.25797709998317	-1.70037520800453
H	1.40097732906342	-2.29205046914404	0.56999485992422
H	0.69485215293992	-1.94893876011870	1.94653434371320
H	-1.58362159777621	1.88043538427965	-0.31072594860120
H	-2.30882501634790	-3.56200389862836	-1.74395422761449

Tc	−0.20832983368178	−0.25785356016890	0.17961786444117
O	0.41157137283640	1.19033802810134	1.11580563828187
O	1.22751728878692	0.26263526883930	−1.25724098204595
O	1.30728841651233	2.70985389175864	−0.63953098455945
P	0.40949171164706	2.76061366106641	0.76715879970283
P	1.15018174129268	1.66732189417154	−1.84671953676535
O	2.57300843776339	1.88770582838511	−2.57702088909550
O	0.03780065599999	1.98257810306521	−2.76815921106208
O	−1.00287351792085	3.12301013992137	0.39350368280430
O	1.17387840328912	3.58011380432822	1.71704066129124
H	3.23836185754523	1.57962738562344	−1.96243544588644

27

Coordinates from ORCA–job Tc_Felprot_d_job25 E −82.211850912630

O	−1.56776154817142	−0.81261837945120	1.30562925584136
O	−0.88609992723274	−1.71770845101609	−1.08562574988719
O	−1.76591182640033	−3.24792440718030	0.69510852148892
P	−1.50456032530978	−2.19416302531864	2.05105367122510
P	−0.88903861930932	−3.18160222312735	−0.58868452109978
O	−1.82702020816588	−3.99169325608853	−1.62728470645758
O	0.45520590685800	−3.80918036439382	−0.54534777814907
O	−0.10404872715359	−2.49691651558427	2.50826370331432
O	−2.65573327671649	−2.43159930427972	2.93460647782835
O	−1.97531903330044	1.00991384015498	−0.56383377530414
O	1.34647525741237	−1.53602045107875	0.69391564863186
H	−2.57310798855354	0.63162699150981	0.09902617697641
H	1.38737497014815	−2.31592433461343	0.10775995746290
H	0.87658904797142	−1.91560970640381	1.53640311318369
H	−1.71833509336499	1.91687020927548	−0.19254719719472
H	−1.23034781499008	−4.32664985575723	−2.29288496038941
Tc	−0.22078402854822	−0.24730969722143	−0.06452354078561
O	0.46481013887217	1.20508122923306	0.79750694947126
O	1.06122880436744	0.20623311172779	−1.65324000437190
O	0.44333752009807	2.61465659792704	−1.28222923442909
P	0.32578761595238	2.75084763786568	0.35317367295622
P	1.57533163154253	1.62410964028337	−1.91477655352832
O	2.82470741897665	1.88867854873398	−0.94085242067492
O	1.88297824746835	1.96771683975874	−3.30770373475783
O	−1.09474818447344	3.16177531612476	0.60106738821019
O	1.48592766000381	3.52288365113605	0.85929665219861
H	2.52246237442829	2.41902632561207	−0.18137702339533

27

Coordinates from ORCA–job Tc_Felprot_d_job26 E −82.211252355740

O	−1.13040041943451	−1.27599624261366	1.70171008625645
O	0.26066409311012	−2.28910527975883	−0.40294942183475
O	−1.52925298037111	−3.54349023316768	0.78376455121741
P	−2.36406799785755	−2.18900827584520	1.38100744980886
P	−0.76293236521970	−3.47307271560272	−0.60909162600505
O	−1.86954799362538	−2.97137197771505	−1.62899360586918
O	−0.14284650487560	−4.74404603398819	−0.99311274547237
O	−3.11061762366437	−2.63715295229197	2.56535540586364
O	−3.14659498452228	−1.64947708876035	0.18518171553432
O	−1.50219778128384	−0.18257079376230	−1.06380235481784
O	1.91194483195590	−0.81235249046099	1.48614471937093
H	−2.35131670705678	−0.42422805079499	−0.60308776131018
H	2.57113106652981	−0.90941971386008	0.78842578196996
H	2.04867351703755	0.17429985466503	1.81387404564641
H	−1.39766276145501	0.75238650595394	−1.34827961582211
H	−2.48985168163817	−2.42432095652589	−1.06599490586748
Tc	0.10150310639051	−0.56050743664972	0.29066510866192
O	−0.02017581295812	1.19694796313145	0.84522985516073
O	1.45254517690240	0.17994824707089	−1.19736226430554

O 1.68581506149563 2.53669076454473 -0.31589957256010
P 1.06812128972766 2.30340250134685 1.22136634674635
P 1.11567234591162 1.62322038637665 -1.50140158069212
O 2.13498199013712 2.05619331786897 -2.68513978955982
O -0.26691452355637 1.99505909905574 -1.90293942939673
O 0.51248676748528 3.57702140664555 1.69608350493624
O 2.16494211910563 1.61703140904414 2.00357880005935
H 1.69529876413891 2.76841878567256 -3.14243270406324
27
Coordinates from ORCA-job Tc_Felprot_d_job27 E -82.212145234100
O -1.63682859817164 -1.23229995402918 1.08240208236991
O -0.68288439405990 -1.93113241855357 -1.29703370463188
O -0.21509180967251 -3.30903411895014 0.71830989385627
P -1.11091770423177 -2.47232294909705 1.90569513906988
P -0.83550449779237 -3.39289345768033 -0.74249215781277
O -2.39602229939351 -3.58091923483617 -0.48085829660776
O -0.25315962172389 -4.43977311889391 -1.58566212634170
O -0.08422853052665 -2.08222433002460 2.91687337251971
O -2.27231034660071 -3.35211261349094 2.21858356282037
O -2.18688791246973 0.67589021297408 -1.58963628337584
O 1.13092686216642 -0.85584641830410 0.88847118849234
H -2.27253730481908 1.46134864913966 -1.03318426672428
H 0.80087467909216 -1.29209189016723 1.73130292979822
H 1.51792276382463 0.03480903374457 1.13322023045194
H -1.51756491212899 0.89237693570886 -2.26597121982404
H -2.54174892874302 -3.46320839380371 0.48821686512514
Tc -0.56620983785807 -0.32359327128216 -0.30870391002806
O -0.53916565584713 1.32743369915247 0.54758343840162
O 0.42817864959315 0.60288539653350 -1.89137455717442
O 0.80342945924190 2.83635023126325 -0.82781300657890
P 0.62072992254635 2.40380473983502 0.75549361836843
P 1.40826804084678 1.78985158132959 -1.92156904689313
O 2.79543418165266 1.36157255449513 -1.27440152111047
O 1.54341959358096 2.44256481385362 -3.22825246706672
O 0.19416466823977 3.58916818987058 1.50924734460685
O 1.86595262074820 1.63113426487177 1.15361946183462
H 2.64116091549891 1.37276187650342 -0.30016657518106
27
Coordinates from ORCA-job Tc_Felprot_d_job28 E -82.211649471320
O -1.07282803254055 -1.31235816805998 1.74533776778409
O 0.10087748681171 -2.06581556506191 -0.57030172232407
O -1.39545687613124 -3.55417481967508 0.72143556334674
P -2.26204296252847 -2.26679659479007 1.38922600674606
P -0.72841972029539 -3.38232953894812 -0.72219980935943
O -1.95623363296735 -3.02896103459685 -1.67363961265867
O 0.01879617830689 -4.56657335048790 -1.14941444138777
O -2.98797864401842 -2.78803645423779 2.55570614175413
O -3.07734172342194 -1.72418902866239 0.20603563814498
O -1.75662366685027 0.24382468286779 -0.63754497821919
O 1.89515195795702 -0.78432927849238 1.41900269140400
H -1.63310755343797 0.45546584633869 -1.57570547251956
H 1.65204052433190 -0.85935484148836 2.34776775260165
H 2.28906809613553 0.16983597559769 1.29208139676336
H -2.43681425738857 -0.48455986322430 -0.43269622291172
H -2.56392823367429 -2.51746939740531 -1.06550702465081
Tc 0.03493035068095 -0.40242588752979 0.32970209530691
O 0.05754648030287 1.27373039115180 1.09814547933801
O 1.22333403904822 0.35479042954434 -1.18728796541686
O 0.98919214974639 2.79612691084594 -0.58503745656361
P 1.21034026573876 2.37322324016610 1.00035089018520
P 0.86049021626177 1.71371075573601 -1.77257300904332

```
O    2.14113540340273    2.10635880872569   -2.67789947934706
O   -0.39331297259874    1.85489870610008   -2.53213612249768
O    0.99225664800702    3.57483005594441    1.81792282184333
O    2.52865944726596    1.63186599268396    1.11334317357436
H    2.90966904014015    1.88321201595304   -2.15221411882055
27
Coordinates from ORCA-job Tc_Felprot_d_job2 E -82.218100916860
O   -1.41055786853207   -1.14229822857666    1.20737332740839
O   -0.72962092967823   -1.81624027942573   -1.23665050949642
O   -0.22806511722156   -3.33835068126591    0.65443926964404
P   -0.96514633794766   -2.45991109495696    1.93744757790912
P   -0.96608154725023   -3.28976933113821   -0.75980133593943
O   -2.50774305033509   -3.40308266149234   -0.38895447518498
O   -0.50025368373650   -4.32330069306191   -1.68894471811343
O    0.19538915078563   -2.22502445866862    2.85708810912198
O   -2.16333209883954   -3.24804493239445    2.32520955058171
O   -1.89462108131455    0.80262321889109   -1.20023716017786
O    1.45524388485239   -1.17284569850899    0.84721398864743
H   -1.85759788167451    1.74081385045812   -0.70908621458216
H    1.05991884591990   -1.52295541005619    1.72400670386676
H    1.67716393887852   -1.96946184991947    0.35119586825456
H   -1.49928785547045    0.96800265138653   -2.06788761523698
H   -2.58508543669211   -3.27549359120216    0.58312193325741
Tc  -0.31024319839017   -0.26643448203547   -0.20670310110903
O    0.20933058560560    1.26769418809631    0.65251119796703
O    0.77610715630581    0.47100895685595   -1.87283996854510
O    0.74911737294502    2.87740387950687   -1.16508335216818
P   -0.02760719640953    2.82721855894873    0.28831526769454
P    1.69214148995089    1.69335284961950   -1.75170075403805
O    2.73443223535190    1.42251602464820   -0.55581965016868
O    2.36960539202379    2.13902775831564   -2.97005771339508
O   -1.49573858966361    2.97438162354417   -0.04651394258135
O    0.61832584995225    3.73376997511628    1.24580619948710
H    2.24360597357703    1.22989986218633    0.24845152113626
27
Coordinates from ORCA-job Tc_Felprot_d_job31 E -82.210044307190
O   -1.50959692655476   -1.20626362512133    1.15112453464289
O   -0.80120835182947   -1.53653846678158   -1.37761707585977
O   -0.10960978705230   -3.16200867415021    0.37877246137674
P   -0.87684409234936   -2.52620358035027    1.75521976017930
P   -0.90024814893057   -3.04637164185317   -0.99621467442441
O   -2.42947028793704   -3.27725748178122   -0.58606202401877
O   -0.43971135651389   -3.97293893574166   -2.03500151474974
O    0.24108626307028   -2.24008716985787    2.69931635588043
O   -1.98702990421738   -3.47104566733875    2.06463587818519
O   -2.46392091264584    0.55399493262022   -1.42909893969010
O    1.18996238438450   -0.57092613916022    0.84675900911889
H   -2.55509084963959   -0.33979086685997   -1.79163630110131
H    1.50031894645615    0.31532239840126    1.32033488226711
H    0.95694983248256   -1.20791565427084    1.59017741666901
H   -1.89943588580787    1.04485108138795   -2.04922761999382
H   -2.45021597981478   -3.34549451205846    0.39960232395817
Tc  -0.64581410962311   -0.02144961314723   -0.15408988845766
O   -0.52710067083233    1.57913572959542    0.73189570039093
O    0.14586378245287    0.97374421517391   -1.79418829003820
O    1.22956857587336    2.81721383964070   -0.47741920474430
P    0.72132905423706    2.54773498471853    1.06145388527465
P    1.45067173334060    1.78820024774014   -1.69940526851061
O    2.61110601843438    0.79817639390128   -1.21687488495158
O    1.83255742813976    2.53256789318178   -2.90191017999318
O    0.32179844990895    3.83369833668254    1.64460891457691
```

O 1.75363699296519 1.68208526506685 1.74884661035630
H 2.27984780499553 0.15206669406576 −0.58210186739522
27
Coordinates from ORCA−job Tc_Felprot_d_job32 E −82.208793476970
O −1.52234493098474 −1.08675406191436 0.93588802445316
O −0.57698280347757 −1.55032828859747 −1.51141243129645
O −0.20225640568320 −3.16173531479976 0.33209508324129
P −0.98303565039940 −2.39787093810677 1.63672822923056
P −0.89422909757821 −3.01308943356259 −1.08795819241033
O −2.47018133837346 −3.02009221812194 −0.74633806425018
O −0.51945344163106 −4.04670640993559 −2.05666097217801
O 0.11089356310602 −2.12121605658510 2.60548646917426
O −2.16841155659694 −3.25720171837700 1.92796569073539
O −2.60696938403463 0.18000217077832 −1.26067357355633
O 1.27661667073047 −0.50196943130503 0.75745041947139
H −3.01065740766216 −0.28586980925960 −0.51200753266597
H 0.96248541881682 −1.13072758711519 1.46993824086903
H 1.51410756083036 0.39400040522231 1.28463295129497
H −2.44071525514391 −0.49995706289156 −1.93042585736361
H −2.52936637507312 −3.14196541474536 0.23393277983387
Tc −0.50485952155173 0.00955313447479 −0.34619638377337
O −0.51255436865787 1.59551612608885 0.57069928733738
O 0.47632008798804 0.98660328409642 −1.87166119922969
O 1.33436849159048 2.87991642675751 −0.46403988495729
P 0.68922753589817 2.58343565533170 1.01104037160605
P 1.72021000350847 1.86716914122010 −1.66725576114737
O 2.88891174302224 0.94179084099032 −1.06940748796633
O 2.19263919585725 2.63867952536125 −2.81799983051344
O 0.22031239271062 3.85016869633295 1.58358438725844
O 1.66785641342123 1.71902890242662 1.78130236351697
H 2.52746845706896 0.26411943581506 −0.48680711718305
27
Coordinates from ORCA−job Tc_Felprot_d_job33 E −82.208678985630
O −1.74376348716980 −1.13141638865879 0.97363496824247
O −0.44509464834171 −1.94634020567038 −1.18152270460593
O −0.49698051212652 −3.34502166344060 0.88549522735334
P −1.51060884463998 −2.40857891394043 1.88543213651218
P −0.83545766493921 −3.37556201864572 −0.66700411735396
O −2.43037640558441 −3.41005933199310 −0.70880867103416
O −0.21694284928117 −4.48566423043701 −1.39891916178826
O −0.67834079771271 −2.11142134960531 3.08398053665391
O −2.79745498135597 −3.16016179118942 1.95205978296624
O −1.75092543081261 0.50484944258581 −1.67925710520015
O 1.07707347524512 −1.03398589125671 1.30753818543476
H −1.60973691980793 −0.09815077417845 −2.41721328096609
H 1.53614547381752 −0.18453174003686 1.62158570209866
H 0.57163341015576 −1.40719627349299 2.07762729447992
H −1.22693526343010 1.33690046531739 −1.88996246195668
H −2.74290589947763 −3.26978069879744 0.21849989887003
Tc −0.33892602894501 −0.34696427389195 −0.14036376370946
O −0.16770010138724 1.31801400290801 0.62535970924512
O 1.19998126708592 0.29439855256488 −1.49789520476199
O 1.75958257100469 2.49700136917840 −0.32023895172357
P 1.04359518292511 2.20498690830070 1.17473555823825
P 1.19267614047333 1.79529507884975 −1.63120217894694
O 2.38616619856297 2.15218713515236 −2.66635559553729
O −0.06953387428775 2.42360630853644 −2.13500469226886
O 0.64268474741968 3.50174206786807 1.73714268483785
O 1.97369862564527 1.28928704449400 1.91661993095396
H 2.31784661995730 3.09106716905823 −2.82406372179379
27

Coordinates from ORCA–job Tc_Felprot_d_job34 E −82.209724568080

O	−1.54897941105249	−0.90798442936169	1.42922793981851
O	−0.60922949395526	−1.75234558941230	−0.90882624177667
O	−0.61069965811050	−3.22562600232500	1.14653169241926
P	−2.00569603095307	−2.37532024258690	1.72028143564858
P	−0.44529362737726	−3.23960842290340	−0.44565304641229
O	−1.70729617265068	−4.00147185246650	−0.97142693063392
O	0.88150813118862	−3.79905170764293	−0.78517029915176
O	−2.12480440811451	−2.69558433966504	3.15365488514689
O	−3.08452684081093	−2.80239352485430	0.75560427913444
O	−1.62451670723952	1.03672363328040	−0.74262476501263
O	1.45559588434058	−1.57916896602843	1.03288273277157
H	−1.26852336624374	1.29681107685454	−1.62095708409751
H	0.96041356774620	−2.03813149721760	1.73050942483917
H	1.55944857378935	−2.25090122241926	0.32923905562897
H	−1.51693460370745	1.91016524321111	−0.20419990730415
H	−2.42271248852806	−3.56994296304306	−0.35783789506285
Tc	−0.15844153092296	−0.25277973905843	0.14926432728069
O	0.49598544732112	1.16980952760217	1.11158709024343
O	1.23486677231622	0.29644892022892	−1.31189632335591
O	1.38656176042903	2.71673476788406	−0.62246071656870
P	0.51449483669507	2.74439614846199	0.80180640733333
P	1.14396396250950	1.72031035209459	−1.85538122476084
O	2.52520754907458	1.94658285685374	−2.65745519041648
O	−0.01745045611766	2.07654929275473	−2.69554388328871
O	−0.90201891615328	3.12876536026038	0.46070698307320
O	1.30530667471799	3.53186113704507	1.75682949482752
H	3.22317054951023	1.60965219261555	−2.09679225195885

27

Coordinates from ORCA–job Tc_Felprot_d_job35 E −82.209112481790

O	−1.68236258805516	−0.93010842531614	1.32642340976444
O	−0.59816354240585	−1.77298532146291	−0.93622440515634
O	−1.66454760726254	−3.35819829209931	0.69408075408341
P	−1.73577340111554	−2.31943359809213	2.06735999734785
P	−0.62982459548960	−3.25469958593102	−0.48710846448630
O	−1.43802856027302	−4.00979318773159	−1.66366128260858
O	0.68407123360059	−3.88492447623295	−0.26207737317712
O	−0.45924216129056	−2.58213653718470	2.82535418715099
O	−3.03849307078631	−2.61042314525896	2.68476813408181
O	−1.57394147135798	0.98089887441133	−0.85002198303716
O	1.23157566575658	−1.58872476417384	1.27235502733325
H	−1.12128776764006	1.23196944133798	−1.68760550904608
H	1.41023107640408	−2.34053050683744	0.68007283348835
H	0.63652545902375	−2.00061133328813	2.02253496304482
H	−1.51329518892003	1.84721564386113	−0.31602116819890
H	−2.26983955049145	−3.54842912951685	−1.76915782348660
Tc	−0.16747857312064	−0.29487700319066	0.22454504791712
O	0.42568610484097	1.14814297500332	1.18290814618415
O	1.35594294226095	0.18553331371501	−1.15738904017089
O	1.42015635974899	2.64692417028583	−0.54104493232281
P	0.46403725562757	2.71522399662111	0.83032847148339
P	1.28181354007556	1.58426725586342	−1.72569288611868
O	2.68743615822140	1.82367476497419	−2.49024724207922
O	0.17067684576111	1.91330797896615	−2.66342142882007
O	−0.92383489321525	3.09539843213250	0.38710342097623
O	1.18960355674663	3.53565173886590	1.80831495671726
H	2.49775677634869	2.48216671985763	−3.15457580133297

27

Coordinates from ORCA–job Tc_Felprot_d_job36 E −82.208678233620

O	−1.74856232701377	−1.13229319180187	0.96573941809793
O	−0.43913649003010	−1.94534935143755	−1.18390868227880

O	−0.50217710892476	−3.34610226579425	0.88174404958612
P	−1.51842686273714	−2.40913655598416	1.87871116673699
P	−0.83433937663534	−3.37407344791533	−0.67190574364096
O	−2.42941913257650	−3.40510123349719	−0.72012428883097
O	−0.21572803896508	−4.48499696200736	−1.40259939921581
O	−0.68876486865858	−2.11165971315643	3.07924846169137
O	−2.80534295895431	−3.16049856559210	1.94254019028277
O	−1.74526473639691	0.50419973703189	−1.68424073019186
O	1.07230708191816	−1.03620642990088	1.31030033756505
H	−1.60355863352614	−0.10132550864271	−2.42003627693647
H	0.56389450671516	−1.40883716093872	2.07917053008556
H	1.53170531133304	−0.18689906160409	1.62451238454218
H	−1.21953107809893	1.33505951836449	−1.89604692584059
H	−2.74540719450773	−3.26630773120724	0.20598266485607
Tc	−0.33811968509848	−0.34688778438570	−0.14034439093699
O	−0.17176238750802	1.31721816232821	0.62873969712684
O	1.20823440566588	0.29664549294182	−1.48689851235495
O	1.75212946622990	2.50544769410222	−0.31279719133861
P	1.03834302782176	2.20388000577604	1.18121221829834
P	1.19809888425954	1.79695043885312	−1.62555296306680
O	2.39893049821690	2.15320078576214	−2.65242353629809
O	−0.06174699181640	2.42067828309283	−2.14080854472161
O	0.63585640010318	3.49719942307474	1.75045043451902
O	1.97116004129002	1.28664714947558	1.91779717465578
H	2.33602825617932	3.09304828322491	−2.80656156990253

27

Coordinates from ORCA–job Tc_Felprot_d_job37 E −82.227701954830

O	−1.34936948368889	−1.12478845910826	1.33409462677221
O	−0.17438035783442	−2.10965881006918	−1.00458045185870
O	−1.16693457549907	−3.58556455131352	0.78902645135759
P	−1.22596983368467	−2.48175486641179	2.01980730934760
P	−1.19282210015423	−3.26386891202049	−0.77816001101505
O	−2.61610736422691	−2.58924059603649	−1.07070092991719
O	−0.99294220361115	−4.49674012146014	−1.53856799641782
O	0.23981002942833	−2.62264375698958	2.63722627244545
O	−2.26749340922380	−2.87040653191985	2.97214928175808
O	−2.05464249633452	0.21451530447093	−0.90475290870873
O	1.72532430805506	−1.11761573429685	0.88898708651673
H	−1.91216190480512	1.20737478258687	−0.91910251572158
H	0.87887653892300	−2.13357453125728	2.09349354027089
H	2.52402253404010	−0.86683091927385	0.40120700001776
H	−2.40557027608342	0.06399766320492	−0.01501108974004
H	−2.52221742362214	−1.62836617363206	−1.10999834199806
Tc	0.18360650655522	−0.51444801489228	−0.05481259648958
O	0.04500192098306	1.20396683688343	0.55551244723810
O	1.67854814549117	0.03530741973852	−1.43563134881319
O	0.59815353924429	2.28874418021588	−1.65293793654316
P	−0.12253424461450	2.60757865555539	−0.20457947822518
P	2.02194562050103	1.51106022305248	−1.65324350073476
O	2.72072357550540	2.04834582183800	−0.30504358016767
O	2.81307012976874	1.81464759761024	−2.85174995817232
O	−1.57478431504932	2.77501360408296	−0.50918899930494
O	0.67915434080931	3.64404186716972	0.49945224507143
H	2.10909279153677	2.68540802714294	0.11900537668819

27

Coordinates from ORCA–job Tc_Felprot_d_job38 E −82.207141767770

O	−0.88810819937982	−1.28138945025973	1.69674774526112
O	0.08304335349441	−2.10188611722406	−0.68151742588265
O	−1.28693139875645	−3.56115863032894	0.77942746298300
P	−2.08394716532306	−2.28135718431597	1.54140166129496
P	−0.78676558004868	−3.40004768525242	−0.73050915465211

O	−2.11619000534949	−3.01360733077884	−1.53032082184075
O	−0.12769296760794	−4.60139130304884	−1.24388102259716
O	−2.61379515376696	−2.79505357852818	2.81108418261653
O	−3.06868512252366	−1.77514083035327	0.47964898040311
O	−1.89829528390397	0.00451004683815	−0.90974974102759
O	1.76978891470692	−0.76811774121477	1.30435412897211
H	−1.83493386970266	−0.37113886230560	−1.79472758807442
H	1.40093757890803	−1.20703022885993	2.08240838196323
H	2.02794832554646	0.21760043518017	1.63309539732912
H	−2.50034673540639	−0.60295728018659	−0.35747856844366
H	−2.67327689872939	−2.56099005981416	−0.83120850243015
Tc	0.00429480270704	−0.38097929379966	0.12904187035551
O	−0.03544548582462	1.33624998177859	0.76128517419739
O	0.98927347008833	0.37728188296150	−1.58703176027461
O	1.92033888386582	2.38327648102776	−0.32621659211418
P	1.16317333932213	2.34166153045907	1.13470822825242
P	1.23458406076419	1.87000705618449	−1.69414055825892
O	2.52549350139405	1.99868630522544	−2.67531335519737
O	0.16470371299545	2.75370761666318	−2.19133518053147
O	0.73325310225636	3.68616429410591	1.53607687077387
O	2.10332035216254	1.56760254885685	2.04166054908798
H	2.43366046581248	2.85999740186049	−3.07561037909280

27

Coordinates from ORCA–job Tc_Felprot_d_job39 E −82.211649932920

O	−1.02563393405893	−1.35095510952886	1.74617094570320
O	0.08486534407159	−2.05460472454059	−0.61603236829402
O	−1.37629391734063	−3.57051243530711	0.68318164890756
P	−2.22408369960821	−2.29705200491340	1.40081536507166
P	−0.74802428512986	−3.36842330571902	−0.77341834208465
O	−2.00037278250516	−2.99597769231694	−1.68471761083387
O	−0.01195074456286	−4.54272191269469	−1.24511842281379
O	−2.92045838663949	−2.84361779240934	2.57371201579916
O	−3.06906494987456	−1.72910126768696	0.25077320922823
O	−1.77019422397278	0.25426702345415	−0.59106019039005
O	1.92789379703828	−0.81687218925592	1.35840314754425
H	−2.44441444402104	−0.47834902690163	−0.37888210260817
H	2.31977221030133	0.14012197700769	1.24403729135398
H	1.70380340734758	−0.91253878740514	2.29008957223736
H	−1.67159829105935	0.47572108236068	−1.52949614554021
H	−2.59123089856750	−2.49719905299361	−1.05007469795909
Tc	0.04443010946944	−0.41104443777582	0.32073483976972
O	0.08471991060294	1.24703337113493	1.12755764128408
O	1.19273329522881	0.38183186163691	−1.20660806674648
O	0.97554852195543	2.80844644315447	−0.54149152373572
P	1.23526979763809	2.34857435883375	1.02742765896526
P	0.82222227426654	1.75556520855126	−1.75199241741025
O	2.08723340359762	2.16696096871262	−2.67141686331920
O	−0.44474564157698	1.91699810794076	−2.48459646856650
O	1.03815382680553	3.53058773059081	1.87816639133380
O	2.55576707340907	1.60484664246020	1.09036553189088
H	2.86505323546976	1.93251495789791	−2.16463003983933

27

Coordinates from ORCA–job Tc_Felprot_d_job3 E −82.217922945830

O	−1.44535147470512	−1.11734879882742	1.15862726938423
O	−0.60933447927647	−1.91230811458840	−1.19325548706352
O	−0.37668038412857	−3.40908840712132	0.76458543389346
P	−1.13279888702559	−2.42610646302542	1.96335629874563
P	−1.00170199005614	−3.34597598334330	−0.70002826518990
O	−2.57155014692026	−3.32510790924854	−0.44419483119224
O	−0.55712640658715	−4.44163835952412	−1.56677192310164
O	−0.01210990964242	−2.23177666929200	2.94120458022284

O	−2.39997292785679	−3.12136419083488	2.30576951625336
O	−1.72730664677802	0.76593968529020	−1.19515531924606
O	1.43906871054410	−1.35549973834847	0.98057679554194
H	−1.80071316359651	1.59277206822959	−0.61067583849943
H	0.96981398494118	−1.64492811743675	1.84560736863121
H	1.64839883963485	−2.17926735158012	0.52442497840907
H	−1.29774863033742	1.07800711342955	−2.00377373076592
H	−2.70787794393053	−3.15634534169408	0.51322508232770
Tc	−0.15784478157418	−0.37358570548250	−0.17203398054123
O	0.29488295560254	1.16883863515845	0.69039825119010
O	1.16876548800100	0.21566549525412	−1.69551894817989
O	0.40213767430027	2.57677977283904	−1.39531225875522
P	0.04542878958325	2.68655533562898	0.22296591166850
P	1.66872961483387	1.65190004736617	−1.85081800685000
O	2.73985765901387	1.96380051494441	−0.69820913227114
O	2.14908308291392	2.03273586702859	−3.18400987984805
O	−1.43413607666880	2.92480518177840	0.24533291099883
O	1.03584955114079	3.58330046882484	0.86101507235548
H	2.30963750685900	2.49374096944560	−0.00543187446208

27

Coordinates from ORCA–job Tc_Felprot_d_job40 E −82.206333301840

O	−1.67524658376612	−0.87914131010487	1.20844646801901
O	−0.59265545282637	−1.76172814539634	−1.04678298208974
O	−1.62425549590999	−3.31778598361611	0.62260166711152
P	−1.66265614663481	−2.25200771381529	1.97888485507624
P	−0.58966131448433	−3.23080270805372	−0.55967678676266
O	−1.37474629103623	−4.03962130636953	−1.71505151423157
O	0.73851623495058	−3.82560272431191	−0.31769857362172
O	−0.35323506884774	−2.46191723575249	2.68991192712563
O	−2.93999441123153	−2.55157442944363	2.64640095728046
O	−1.76518814817384	0.90127590440198	−0.81892194253968
O	1.31599413186539	−1.48825582875467	1.08081995286323
H	−1.49906255413780	1.89016701663613	−0.57231477812128
H	1.45054770713866	−2.27402996964799	0.51869068976310
H	0.73682144385511	−1.86526643520921	1.85352771563604
H	−2.50320785393335	0.68652076491426	−0.23156754407759
H	−2.19415122712379	−3.57472616693503	−1.88133478163474
Tc	−0.17419098081923	−0.24465042180308	0.06434138990480
O	0.32273373380964	1.22145983626813	1.05006812548672
O	1.26133561249873	0.30580989527439	−1.30713822857056
O	1.44391682280541	2.70692916236395	−0.57235305458911
P	0.34683987202356	2.78115072409236	0.65198151743136
P	1.34480664950404	1.73415755295666	−1.86212486330237
O	2.88478225726574	1.84320828156133	−2.35629790075001
O	0.42917835156663	2.20181462575475	−2.90396968427161
O	−1.00909368818265	3.09262605042415	0.05601942413753
O	0.90482232263469	3.65279748357016	1.69417002632432
H	3.41645007489073	1.44369309115807	−1.66873207735771

27

Coordinates from ORCA–job Tc_Felprot_d_job41 E −82.206485622760

O	−1.61643462690764	−0.91758130759354	1.37516762891144
O	−0.52309501324063	−1.77825203551351	−0.88487511413037
O	−0.65340221284511	−3.24316190250831	1.16328514999579
P	−2.04552128179261	−2.38319250009938	1.72899573377223
P	−0.46153876222343	−3.27812717157566	−0.43296711478851
O	−1.76535581791422	−3.95410663820509	−0.97457159513257
O	0.81639323660691	−3.93677073824668	−0.76391843691180
O	−2.13591093737333	−2.65764077787228	3.17356576621699
O	−3.14218044367853	−2.84063521551886	0.80066307076808
O	−1.54105232057578	1.00617976790222	−0.78300583160358
O	1.41014956591353	−1.60464324440544	1.18209660160722

H	-1.11499493492307	1.26233462043345	-1.63516014905692
H	0.83007512184206	-2.22183772141417	1.66742053794625
H	1.72913325148672	-2.11475528367027	0.42102342731369
H	-1.45959859054131	1.88149948740670	-0.24838560287774
H	-2.47219744962318	-3.53553209597616	-0.34231701095301
Tc	-0.13383522099393	-0.27992342871254	0.20860215239428
O	0.47088696772309	1.14758573417494	1.19448200754604
O	1.38969051744920	0.21672003728600	-1.15908713799991
O	1.49152186075070	2.65962171660678	-0.50181969171568
P	0.52988264059314	2.71556048353643	0.86740698955606
P	1.30951861610487	1.62685123509038	-1.70438775048783
O	2.69255010555022	1.87187095214858	-2.50534394096993
O	0.17173332204180	1.97049669886004	-2.60168598447101
O	-0.85459450647482	3.11189156771073	0.42195937825074
O	1.25811721455023	3.51613024293021	1.85952157615658
H	2.45945970148802	2.45391750092897	-3.22476465509694

27
Coordinates from ORCA-job Tc_Felprot_d_job42 E -82.205250385360

O	-1.58919996319549	-1.08872350364358	0.95805149845004
O	-0.20200934282065	-1.90406380761754	-1.12167013649498
O	-0.48748045476055	-3.39127605705896	0.83869587448118
P	-1.49307719190278	-2.40355798265435	1.81670446165405
P	-0.73854482208611	-3.31601712143928	-0.73840226931109
O	-2.32813348353216	-3.25575928378209	-0.85594446085177
O	-0.13504347201985	-4.43852092928561	-1.46484568119540
O	-0.65695275447830	-2.21461102988912	3.04224864281380
O	-2.82213403896284	-3.07395887734353	1.82478877982239
O	-1.43456607397381	0.66045611396832	-1.24019983751361
O	1.29405383374698	-1.39130977403735	1.47342038233878
H	-2.28808793497303	0.37456222505347	-0.88943352713298
H	0.62805463673706	-1.62533224185071	2.19945482105353
H	1.49604388004441	-2.24064444713162	1.06220621602275
H	-1.32671115861289	1.67597065723773	-0.94210637250826
H	-2.67633714913631	-3.13913388796262	0.05792910196999
Tc	-0.01703626963235	-0.34609599045363	-0.00424670255414
O	0.17268169782604	1.16028919681624	1.03219408161081
O	1.57078010372329	0.30224561874306	-1.13233619533966
O	1.45742208685760	2.70778281583002	-0.39965803149962
P	0.15078292588654	2.70652444472619	0.60560258949828
P	1.60086747104477	1.72749186525316	-1.66966912619140
O	3.17181834488629	1.97687645198516	-2.00800306626643
O	0.79288695718444	2.14109205819557	-2.82920154820806
O	-1.08856486326909	2.89599881313102	-0.24603855600631
O	0.42762474527055	3.64264706302379	1.70200323226230
H	3.16026229314112	2.55156760447338	-2.76964416137285

27
Coordinates from ORCA-job Tc_Felprot_d_job43 E -82.204927899180

O	-1.71207958545509	-0.85908473248304	1.40094211623667
O	-0.76137783379361	-1.70178908267238	-0.92343354100496
O	-1.83654945550187	-3.28406577949586	0.71375646615704
P	-1.79648669348598	-2.25871226877016	2.11276672480099
P	-0.82252547132549	-3.17286770299877	-0.46285748914985
O	-1.61975644473038	-3.96465701176552	-1.62879988007365
O	0.50732472878588	-3.81157842143974	-0.27768080064302
O	-0.49584070917210	-2.60020779261571	2.79542640907502
O	-3.07304426676535	-2.50809266716858	2.79706633019921
O	-1.61635235681535	1.09266014695447	-0.74759565950809
O	1.17478333885515	-1.60880666742058	1.21647397991969
H	-1.20080407526406	1.33486037738382	-1.60551745722782
H	1.28677114271462	-2.36931111670367	0.61431897674909
H	0.59715807177555	-2.00806675535120	1.98268134125517

H	−1.49505595695916	1.94747193186227	−0.20802455164499
H	−0.93899474476948	−4.39603771027990	−2.14008903090131
Tc	−0.22344559694013	−0.26075573465501	0.23846521674872
O	0.47643420772641	1.14003369551058	1.18814622742118
O	1.25526282218077	0.17888563593445	−1.21011358849533
O	1.46475167477668	2.62195143522350	−0.55390770307627
P	0.57234269136741	2.71076635596641	0.85840902957536
P	1.22091692425909	1.59140140092658	−1.74929738233008
O	2.60247212891889	1.77810596099496	−2.57332464738941
O	0.08817121776398	1.99004011854511	−2.63147695468644
O	−0.81335219904394	3.16320406048139	0.48356046673134
O	1.37851529053740	3.47857030828606	1.81637460632371
H	2.42016114276950	2.47058201532945	−3.20436920082237

27

Coordinates from ORCA−job Tc_Felprot_d_job44 E −82.212232297070

O	−1.66648593903156	−1.17952607973588	1.08926502856700
O	−0.82384114771128	−1.79672427214061	−1.32584052669155
O	−0.49309892600866	−3.38664110490262	0.58042950348455
P	−1.26569691276842	−2.51397784443225	1.83589579337468
P	−1.11793299245360	−3.27565075378933	−0.87207605073150
O	−2.68985980438201	−3.32696353764190	−0.61034734703696
O	−0.65091308542544	−4.30269944178556	−1.80894553090742
O	−0.18397913081300	−2.30091823440504	2.84305441022498
O	−2.51038126135922	−3.27768238157693	2.12982262012220
O	−1.92475825241598	0.84186065708184	−1.31942188323952
O	1.17727328778817	−1.29924710036704	0.79767489138648
H	−1.74999159976684	1.79061677817865	−0.90421445293374
H	1.87955087619828	−0.61104585084488	0.83580139978938
H	0.86204510131377	−1.59093825638758	1.70031237555892
H	−1.49273538159386	0.89165071453737	−2.18108239838606
H	−2.82056897520368	−3.22761392856929	0.36002592856436
Tc	−0.44599563438694	−0.32389349678592	−0.21085407861930
O	−0.00875327974703	1.15910006248244	0.79600976178969
O	2.75027329262895	0.73062473786098	0.07018221506328
O	1.27568906715743	2.73502729038273	−0.58969531904013
P	−0.00573836264457	2.72637363731210	0.48407732175732
P	1.92390417989721	1.33105360605675	−1.00835128226175
O	2.90416723474181	1.83444300011263	−2.19731920380511
O	0.85885852656910	0.50307517718105	−1.69395175087212
O	−1.23602937140985	3.02945037807420	−0.34164543108077
O	0.33959258678855	3.58229419524819	1.62548089282417
H	3.75480590173981	1.95245205372654	−1.78238688266148

27

Coordinates from ORCA−job Tc_Felprot_d_job45 E −82.204269072030

O	−1.53433433381007	−0.82852655579715	1.27110646921601
O	−0.88533342990392	−1.79043923095603	−1.15445954397581
O	−1.76973601758192	−3.24130666942553	0.68495269144114
P	−1.40410740609380	−2.21111195695276	1.99636901822461
P	−1.00347478810708	−3.26551230481792	−0.68146407072854
O	−2.16366836194605	−3.88058396127156	−1.64177674489928
O	0.19267026435080	−4.11617308342915	−0.77498491931562
O	0.04040119269952	−2.49844889538483	2.35148773055093
O	−2.45540407766055	−2.45993366744125	2.99401949545602
O	−2.02005281605311	0.95627859339436	−0.64447614140982
O	1.35025884452887	−1.53867059229038	0.50546626074103
H	−2.60723773327773	0.58960065050872	0.03375262202781
H	0.87585857809339	−2.02150074352280	1.35165677113623
H	2.05935456592587	−0.97286826627084	0.82824737318159
H	−1.75006208716313	1.86576343347849	−0.28151326205389
H	−1.70154782124085	−4.48332967513170	−2.21928838064645
Tc	−0.26069040113671	−0.30800373823866	−0.19607575202363

O 0.39171653084327 1.13846410443272 0.72547687063825
O 1.03535700285729 0.22601040171839 -1.76155439714538
O 0.54600795773965 2.63913996860135 -1.27532400437198
P 0.28626025953254 2.69902874872837 0.34859531927685
P 1.67841428330290 1.60847243258358 -1.83530964060246
O 2.79812598091482 1.69339570674673 -0.67443817509200
O 2.19926513619359 2.03418174747615 -3.13918977579276
O -1.14641282293724 3.11282017128525 0.50322842661752
O 1.41021654558696 3.43487847842145 0.97998473485302
H 2.47755495204382 2.31287489255035 0.00741103422797
27
Coordinates from ORCA-job Tc_Felprot_d_job46 E -82.202588456820
O -1.57191312385502 -1.19965072536319 1.17120907130250
O -0.81240421879211 -1.61285734330988 -1.30674616147248
O -0.18085470197936 -3.20442472146989 0.49191159631118
P -0.97382711788782 -2.50443977703121 1.82607276770702
P -0.94400757117408 -3.10967460006904 -0.90700460963009
O -2.48130487517893 -3.33306780358468 -0.52402058894080
O -0.45918988279397 -4.06551273607317 -1.90784455229260
O 0.15741125771773 -2.20236847339053 2.76066244070603
O -2.08698538292006 -3.42706670125998 2.17319425322786
O -2.33222412611477 0.56558241704531 -1.46762674861816
O 1.14972640627552 -0.66371557821940 0.94327734261333
H -2.45685753223484 -0.33255368164018 -1.81064311934520
H 1.71980853332651 -1.18382415939050 0.36583200722899
H 0.87086740080512 -1.24749755419998 1.74118644857362
H -1.71783942144283 1.00432978617733 -2.08455350367391
H -2.53108718939764 -3.35059783223428 0.45817482813918
Tc -0.61302423317602 -0.04319776057041 -0.11926321097097
O -0.43585407299527 1.55993082419856 0.71776956433405
O 0.26443990986438 0.79280242799519 -1.78762304015552
O 0.91254799236658 2.96383663286511 -0.74800482528918
P 0.67999977861205 2.72283638451248 0.87618310247387
P 1.46043962647940 1.76864400821093 -1.69751618009749
O 2.61166581584936 1.08883443540083 -0.84475207504515
O 1.89874314956198 2.29802039134606 -2.99500717791336
O 0.12131419192963 3.95015134504174 1.45935932093731
O 1.93257268087016 2.09245009610966 1.40914337618578
H 2.45723670398543 1.36753068789850 0.10852966736024
27
Coordinates from ORCA-job Tc_Felprot_d_job47 E -82.209884028260
O -1.61229833005444 -1.12891981151363 0.80709644668964
O -0.44501820729916 -1.92976335786552 -1.45119837491866
O -0.27432319652882 -3.28093758765899 0.60758436003504
P -1.16898010469328 -2.34563229582758 1.71034832210367
P -0.81170218873653 -3.33904835557403 -0.88718523315787
O -2.40471931876001 -3.33244966698511 -0.70637665830031
O -0.32138100689330 -4.48238241906707 -1.65970244137568
O -0.16805418112429 -1.94913528007668 2.73973898540237
O -2.39347291311908 -3.14766124627776 2.00186697136948
O -2.42148525386940 0.32205647545133 -1.37739715359760
O 1.25403859161436 -0.76195157947479 0.77973314864699
H -2.88959934277572 -0.28202447280748 -0.78095678637243
H 0.83909296190961 -1.22157439737626 1.56729457865820
H 1.55444175336276 0.13908353829767 1.11223292833865
H -2.43005947254139 1.19200399621205 -0.95772091239780
H -2.57038337204559 -3.27293068770160 0.26546932759186
Tc -0.39262671541276 -0.28143386717871 -0.50437073573330
O -0.51908180615406 1.35934372526199 0.36198414213439
O 0.76340799285580 0.64217527805183 -1.93059670546808
O 1.00239923162827 2.87133067941686 -0.82918512286893

P 0.59430698563687 2.45786159498198 0.70764483905716
P 1.74675707806360 1.81980735424395 −1.83335267523488
O 3.02935544367297 1.38621070897328 −0.98599308814602
O 2.10175501285230 2.47294889943543 −3.09564092722381
O 0.03792218477688 3.63793479286747 1.38259008751227
O 1.77981240926436 1.71379676667248 1.30020999354792
H 2.75929576207117 1.43579122568128 −0.04221731734448
27
Coordinates from ORCA−job Tc_Felprot_d_job48 E −82.211548584440
O −1.56783014199466 −1.05410186181509 0.94978595405183
O −0.59866030736216 −1.72135285935068 −1.44402739825589
O −0.35300584508822 −3.23168282254723 0.50893206428739
P −1.11310647562001 −2.34070358928081 1.74464908986858
P −1.00196083085220 −3.13668595684282 −0.93477695026241
O −2.58320729276447 −3.03091442125651 −0.63309789857721
O −0.66991322812560 −4.25107419101897 −1.82581483084216
O −0.02203266191998 −2.05270236633777 2.71582418672616
O −2.34557411621079 −3.11654114150464 2.07051315608759
O −2.51213936356207 0.16939368010164 −1.31552963504317
O 1.27688879538036 −0.70884868468039 0.76704625315270
H −2.95118544673115 −0.20179143551941 −0.53434208630483
H 1.64909066558384 0.15578380493980 1.12570260024420
H 0.91122651680344 −1.21630586903260 1.54851598090275
H −2.41167293079929 −0.56902227720566 −1.93417904886971
H −2.67104524802213 −3.08383870180474 0.34971858551158
Tc −0.43910470206725 −0.11791694380334 −0.37111272775082
O −0.39684080079165 1.48458959486332 0.53245021514334
O 0.62962349462174 0.76964198771501 −1.88884685926322
O 1.12044206591830 2.93846253864398 −0.73394891553059
P 0.79354188948837 2.52617469204383 0.81820896474412
P 1.70539867541452 1.86531273415304 −1.81831946860332
O 3.00499986152950 1.30630509925536 −1.07487304690093
O 2.02701802078427 2.52986313531847 −3.08379173220573
O 0.36902164869942 3.71993149781017 1.55763451221164
O 1.96967197853701 1.69834223858762 1.31820152915831
H 2.81975577685200 1.36418211814727 −0.11262249473237
27
Coordinates from ORCA−job Tc_Felprot_d_job49 E −82.198392957920
O −1.71334820687717 −0.81548630751444 1.28021898557555
O −0.69212034453911 −1.72933504937777 −0.98939928068264
O −1.83620992425674 −3.25449651673473 0.65111717212370
P −1.79797059992325 −2.19680123982118 2.02802997277494
P −0.78356032723361 −3.18830913730642 −0.49345065164666
O −1.56684514898390 −3.99689300416897 −1.65726038061569
O 0.53401358726393 −3.83323230104243 −0.25627214988808
O −0.50387378845341 −2.51688639254246 2.72411958328993
O −3.08783314951092 −2.41611356117866 2.70052243656599
O −1.69630589096483 0.98297254030373 −0.74286600529627
O 1.23212876918549 −1.58510152301237 1.15014089306396
H −1.39160204689365 1.94989623452614 −0.47816019878000
H 1.32716728782825 −2.37225352232818 0.57932788080616
H 0.63666469920482 −1.94216335384086 1.91233724451718
H −2.45879712551856 0.78732842570019 −0.18056869601238
H −0.87989602419601 −4.33186468805089 −2.22906160660093
Tc −0.17821651590504 −0.25515921537027 0.13699785797514
O 0.40238888983811 1.16791753488545 1.13713018491890
O 1.28311470180588 0.22370336473690 −1.25031149485014
O 1.58451892603012 2.61167800240438 −0.48428583271221
P 0.50894495352577 2.72882088224478 0.76055901712496
P 1.36931761561287 1.65372937369235 −1.76251204953558
O 2.86388696738371 1.74279227436126 −2.40139342249606

O	0.40352703015559	2.18747709623269	−2.73916307532704
O	−0.83217835778575	3.12217650333911	0.18130260665669
O	1.12279834401484	3.55991055610449	1.80424198251651
H	2.78968567919258	2.41019302375816	−3.07944097346593

27

Coordinates from ORCA−job Tc_Felprot_d_job4 E −82.217532616740

O	−1.43639546435864	−1.18187253487008	1.10890557843588
O	−0.72236622925565	−1.95221077721555	−1.30844693570253
O	−0.42585983873312	−3.47553188120236	0.63538861867056
P	−1.07793842518320	−2.49962141266642	1.88078542687016
P	−1.11327298950233	−3.38495711347517	−0.79159129655652
O	−2.67059227716134	−3.30230956364424	−0.45613437362559
O	−0.77202761561366	−4.48446874832582	−1.69698421742529
O	0.10573947631580	−2.30693041282467	2.78750212247826
O	−2.32431351404441	−3.17758195753502	2.31939728417520
O	−1.76906293334589	0.76079975577791	−1.21844025904803
O	1.45443118260183	−1.40330067170433	0.80651780951564
H	−1.81601678745438	1.55169157845935	−0.58695344146665
H	1.01364745610533	−1.78826286082381	1.65888643525692
H	2.00568922354701	−0.66033113616562	1.07928380609352
H	−1.33427018685021	1.11306430856329	−2.00714373993249
H	−2.75279658178411	−3.14027608590550	0.50560688491186
Tc	−0.19912303767001	−0.46590481984784	−0.28646667799687
O	0.27716453517128	1.03408028136292	0.66289172746283
O	1.11286826374542	0.20617551597469	−1.79311085777817
O	0.44037210097881	2.56331299300660	−1.32323718317000
P	0.05380377095903	2.57837489187677	0.29196330669469
P	1.68735787282324	1.61970505473439	−1.79154644350765
O	2.69476951159735	1.76015739797961	−0.54106347695361
O	2.27024768291617	2.10048951124782	−3.04899071901032
O	−1.41660560737171	2.85515933878640	0.31680634775720
O	1.06589488888358	3.40382751238523	0.99584464084592
H	2.28805554155205	2.37122183563033	0.10222963195291

27

Coordinates from ORCA−job Tc_Felprot_d_job50 E −82.196559887900

O	−1.90337942566704	−0.83220968250610	1.07965561102481
O	−0.67730899450033	−1.87777694053658	−1.07943357994081
O	−1.72151606844508	−3.28881540221188	0.70541876387490
P	−1.79619560974224	−2.13176182566074	1.97251696177171
P	−0.70130242807617	−3.30975985907609	−0.47997673295518
O	−1.54511291922969	−4.15644679504641	−1.58876506621836
O	0.58325016490251	−3.99520898452054	−0.26037111736724
O	−0.47497472472469	−2.20474539450492	2.68206325791841
O	−3.04734193917326	−2.43534822520576	2.68780169271194
O	−2.06147260142233	0.83540470812721	−1.33041123998915
O	1.06782320613831	−1.10331184014356	1.00345107866278
H	−2.57082776541860	1.23709409860824	−0.61932373999759
H	0.59196762755139	−1.57072907079681	1.77806942691728
H	1.56620739203925	−0.30833677520427	1.36417611022954
H	−1.47530883489403	1.55681886435102	−1.70823182873766
H	−0.96642154469457	−4.87862459715128	−1.82059833985055
Tc	−0.47687291400321	−0.24733053859612	−0.15724461199075
O	−0.19958955674520	1.40029016706798	0.63709922359796
O	0.99537840954212	0.38015123197395	−1.55495332096545
O	1.70010782997854	2.53885183816338	−0.42123204686600
P	1.11116729166115	2.18210168657615	1.12025743296142
P	1.00816276613204	1.89377917542595	−1.70132467676936
O	2.14219923693425	2.19719067685061	−2.81230861689521
O	−0.26482593642447	2.55688564115100	−2.09112043490540
O	0.81849257789301	3.45547464378972	1.79453788267321
O	2.06697396239748	1.18746785543697	1.70570644649621

H	2.87012081685908	1.61339534850952	-2.60355855761070

27

Coordinates from ORCA-job Tc_Felprot_d_job51 E -82.192045617140

O	-0.70069727432877	-1.43925988129662	1.80942292754147
O	-0.10207398734441	-1.98424352647923	-0.80335417050539
O	-1.25776739236566	-3.58347501491270	0.70357252925538
P	-1.92555709540261	-2.42734300242072	1.74125682523279
P	-1.03106817715006	-3.26677757939047	-0.84185457933719
O	-2.44477762274777	-2.76019257893897	-1.34363359641893
O	-0.49793562054977	-4.40094472625720	-1.59286067241956
O	-2.20236162410225	-3.09211749950010	3.02125625820521
O	-3.07498674368956	-1.80276875685296	0.96117987805906
O	-2.01692428436591	0.04379724416108	-0.46607232944692
O	2.02874953214282	-0.85282696842306	0.73211430381272
H	-2.04967625213937	1.04989037415800	-0.48314041463602
H	2.33994014987038	-0.09465437179266	1.24248554418865
H	2.42916801012687	-0.66490726995165	-0.22147794045284
H	-2.65531443571359	-0.38401419334520	0.16331406928935
H	-2.86607995793669	-2.34510133678256	-0.53637326951508
Tc	-0.05227256123176	-0.42769575677099	0.19893058542410
O	-0.03729788845674	1.24624837850823	1.04971195801262
O	2.71569739157489	-0.08600837491940	-1.54285104201105
O	0.65581716305276	1.34709676107362	-1.24695765376107
P	-0.04447727592473	2.35150479196100	-0.11445174205849
P	2.32848860082201	1.36213335150873	-1.45863243204878
O	2.81008240978831	1.86185158297388	0.00830528323093
O	2.67105364180528	2.31235876046199	-2.52827023611995
O	-1.47179827103398	2.54318640258988	-0.52248364994102
O	0.81050239461257	3.52003498777060	0.19944623859346
H	2.28096716309704	2.66872819186282	0.17331733206613

27

Coordinates from ORCA-job Tc_Felprot_d_job52 E -82.190910756040

O	-1.66898736369098	-1.27527347100253	1.18281181427828
O	-0.04190968396837	-1.27013417482620	-0.86372274180384
O	-0.06955804007988	-3.19530162459426	0.69605184402620
P	-1.31139197131431	-2.72044835134824	1.74859496079994
P	-0.24927794145285	-2.81522070502102	-0.85348565369330
O	-1.78686557684967	-3.09929628357261	-1.13142779294348
O	0.66414557176752	-3.55358791314675	-1.72727717149989
O	-0.61163059390364	-2.60896880413020	3.06541905954282
O	-2.45699740005832	-3.62749291272719	1.49469324240068
O	-1.78470491444872	1.06504434895840	-0.64587466601114
O	1.11073597688485	-1.00751752810146	1.90674845626615
H	-1.67178203941719	2.01135866098941	-0.34706433996711
H	1.58789443441179	-1.63276802184259	1.34813324267550
H	0.52561404380366	-1.58362128110613	2.49971047901966
H	-1.28889113982131	1.14626884528094	-1.56231930066498
H	-2.23617434152581	-3.23355012072046	-0.27129142776802
Tc	-0.36640834914292	0.04082720762205	0.56719996375712
O	-0.05676307852899	1.49242379854456	1.59543269485503
O	-0.31253502171310	1.49287486565379	-2.60943627987983
O	1.07746140978655	1.53601150249249	-0.45737118392289
P	0.50757803048110	2.68759209045882	0.59136222556879
P	1.11285497851136	1.61813068356823	-2.14142972054428
O	1.86060822455523	0.21013014525582	-2.37356913412062
O	1.95739663215538	2.73561574060380	-2.57706734214750
O	-0.72219656030647	3.33604047515616	0.04172901245526
O	1.59816657728066	3.44399558144259	1.21174368325916
H	1.27301813428555	-0.49863274372501	-2.08639393028167

27

Coordinates from ORCA-job Tc_Felprot_d_job53 E -82.190858008620

O	-1.62195420029576	-0.84599775240220	1.31009579308622
O	-0.62644744273577	-1.80974180229841	-0.98612273492666
O	-1.96414772273732	-3.21479149557794	0.59647263940650
P	-1.71194843875005	-2.25897951329381	1.99000685332891
P	-0.94278711231384	-3.27579739264437	-0.58988553600236
O	-1.93348103628822	-3.79597329708203	-1.77203304121113
O	0.19286451839517	-4.20513211795739	-0.47522950479892
O	-0.36474271078847	-2.68090789452145	2.53253779879170
O	-2.91746604621468	-2.44318780643025	2.81325122408808
O	-1.67533308621844	0.90115901565789	-0.76807560470382
O	1.34055246192755	-1.55065695200780	1.13653849950942
H	-1.37435538056120	1.86597427160716	-0.49995995453912
H	1.78188591422472	-0.93726649904554	1.73338840944485
H	0.68982107610901	-2.12503597675223	1.75106612444247
H	-2.42281035602731	0.69506233001504	-0.18900664686742
H	-1.37827403136064	-4.34018372725765	-2.32520346182592
Tc	-0.13185332060661	-0.33006561501207	0.07523482373367
O	0.42955128953243	1.09561822707277	1.10349943662236
O	1.33214918522422	0.20925081308833	-1.28943331586865
O	1.60488287229648	2.58088629242221	-0.48550238591745
P	0.51350099186674	2.66134794891769	0.74935899727190
P	1.40290580452540	1.64682766667490	-1.78300570881862
O	2.89874536853154	1.77097654358996	-2.41455634767829
O	0.43327062810631	2.18240154333770	-2.75456386166740
O	-0.82530677839317	3.05040414744254	0.16706104187471
O	1.11658729686502	3.47016992385820	1.81894381210577
H	2.79359026397156	2.37813912876119	-3.14297735522476

27

Coordinates from ORCA-job Tc_Felprot_d_job54 E -82.214551205210

O	-1.46759096366681	-1.17738007854785	1.13821599014016
O	-0.79159627320103	-1.68405293325466	-1.30219172733538
O	-0.22924051369632	-3.31538083407459	0.45640862695823
P	-0.99626061125153	-2.53233540084899	1.78606357162500
P	-0.96959618068671	-3.19329459271262	-0.95387631454546
O	-2.49415254416899	-3.44692938440591	-0.60886281247623
O	-0.44793423074569	-4.11226098678520	-1.96708042968097
O	0.14760639107652	-2.32564699462132	2.72930298814341
O	-2.17231070051011	-3.37252900552258	2.12061058780705
O	-1.91885757697170	0.93081480473461	-1.08772804887530
O	1.49058958962516	-1.21787958221082	0.81386016045696
H	-1.54495238932076	1.11121925420953	-1.96067155135810
H	1.06328416129118	-1.56605026941385	1.67968507545145
H	1.58778554829175	-2.02397437621236	0.28617199569786
H	-1.84531270482047	1.86783903339285	-0.53780846212921
H	-2.60375626482369	-3.34352018801615	0.36337701913130
Tc	-0.34521056143264	-0.19839212361816	-0.16710565179206
O	0.17978796611067	1.26713343887235	0.79302335956880
O	0.77529945848263	0.55480533409421	-1.79873157527752
O	0.78073198585719	2.91746805068666	-0.96975701917174
P	-0.00933438495507	2.84741871911118	0.46607176570129
P	1.70986568196182	1.73670582545047	-1.57233365256300
O	2.71689126062884	1.36505896113196	-0.36564165585296
O	2.44233442788843	2.25669391784840	-2.73166524998655
O	-1.47922039999160	3.01287292105954	0.12458236038709
O	0.64446582332158	3.73851741512098	1.43039721975658
H	2.41608400870023	0.59757906352764	0.13758342387537

27

Coordinates from ORCA-job Tc_Felprot_d_job5 E -82.217337860300

O	-1.46457435415117	-1.05758902305491	0.94153159911071
O	-0.41340537475514	-1.91553591164858	-1.30122709166147
O	-0.38088349761767	-3.36056042001566	0.70250483838348

P	−1.20920694388637	−2.34064930160638	1.80977588338562
P	−0.89144346082287	−3.31816139154097	−0.80748030179723
O	−2.47774902638006	−3.22721356204969	−0.66077803776839
O	−0.43359713816091	−4.45910669921814	−1.60541880774074
O	−0.16698558272857	−2.11378783579330	2.85878874524108
O	−2.51044041804906	−3.01474551459631	2.06805946231142
O	−1.76481828944503	0.64189529948462	−1.17454092013425
O	1.49451968352987	−1.34583144389985	0.98482630973226
H	−1.71990409234427	1.58031675400269	−0.77316607569234
H	1.68184746850870	−2.19455482099348	0.56652405972571
H	0.94848056262593	−1.58195425141896	1.81159442037735
H	−2.46888528018167	0.19503100650614	−0.68371846352737
H	−2.66798559823302	−3.10976823967854	0.29850893692609
Tc	−0.03348289808389	−0.35800460262301	−0.26605798719301
O	0.27546207500267	1.17885624988976	0.67243444983922
O	1.45255683460747	0.24092578752078	−1.58818084258388
O	0.50647672968483	2.55845417754566	−1.41445116479919
P	0.02412841122459	2.68155701783998	0.15461072546443
P	1.85219015336475	1.70943003214852	−1.76186825188332
O	2.81299681143694	2.09992346633837	−0.53325293892060
O	2.42827231588394	2.09470839275895	−3.05387784187776
O	−1.46198729168769	2.88090828704783	0.07531375236106
O	0.92731239570227	3.61679764138912	0.86542426912515
H	2.30050580794836	2.61315888407719	0.11602126725200

27
Coordinates from ORCA−job Tc_Felprot_d_job6 E −82.217696609620

O	−1.48175127623707	−1.12017887229036	0.88142059378333
O	−0.55913049253397	−1.95102897261054	−1.44160184044100
O	−0.41121917195438	−3.41104699804836	0.56251833708925
P	−1.14201181532897	−2.40229703643176	1.72573861285192
P	−1.00419333409872	−3.35648332122591	−0.90986386848298
O	−2.58132329949770	−3.23160581659767	−0.66391525069900
O	−0.63709928275207	−4.49788889176517	−1.75030324369547
O	−0.02396773529000	−2.14985833685601	2.69177392835430
O	−2.41394022446095	−3.07574993999123	2.10255106603373
O	−1.85893376181173	0.62787852965689	−1.19317008137500
O	1.50544946612436	−1.34497914714886	0.76798826743893
H	−2.55313243869645	0.20434896719827	−0.66925830622304
H	2.05387171961405	−0.59999198603521	1.04255941148971
H	1.00445982566920	−1.68275908384414	1.59529241217317
H	−1.75540524194420	1.55779486952864	−0.78423528282146
H	−2.70881738458358	−3.12422974396540	0.30322237544800
Tc	−0.11052813596511	−0.44157487967244	−0.40113039857017
O	0.23331087827643	1.05223638214739	0.61341102516803
O	1.36692868115936	0.22434251139983	−1.72468508631279
O	0.61229100174525	2.58032708621071	−1.33436029361849
P	0.03128593329348	2.59203081324063	0.20746281782011
P	1.90813480384767	1.64968051910852	−1.65387476194993
O	2.74491704772351	1.77385335612620	−0.27199130975596
O	2.66538631745458	2.14468609090230	−2.80728505389701
O	−1.44152990358176	2.84049581989065	0.07132281731507
O	0.92567257769020	3.43787286301418	1.03558367770811
H	2.27067523325616	2.39862521234593	0.30672943411648

27
Coordinates from ORCA−job Tc_Felprot_d_job7 E −82.217532414610

O	−1.44236898168523	−1.19696990865709	1.09194177139158
O	−0.70091090105103	−1.94978951521407	−1.32303702727667
O	−0.41248660935819	−3.48092367996145	0.61580018779391
P	−1.08030847039864	−2.51553109302208	1.86088879463213
P	−1.08804667565036	−3.38704278547932	−0.81619481903348
O	−2.64880234011390	−3.31410097443365	−0.49378137031509

O	-0.73396112094310	-4.48065177476084	-1.72391448666390
O	0.09565513853450	-2.31875123089985	2.77683659546550
O	-2.32481371434319	-3.20409480523594	2.28791016580864
O	-1.76772703672718	0.75682078127598	-1.22688031099967
O	1.45420445701478	-1.39744540544927	0.81082692794933
H	-1.32853176584753	1.11477694356519	-2.01052372634170
H	1.00890343223549	-1.78970652452174	1.65780416672369
H	1.99882812299763	-0.65234116247351	1.09105114339777
H	-1.82359930424708	1.54448781483309	-0.59164309831706
H	-2.74062678733731	-3.15754743786614	0.46782806745834
Tc	-0.19739511674392	-0.46514905957853	-0.28849179480281
O	0.26041839450693	1.03242250251268	0.67270101667359
O	1.12278351896441	0.22374673469939	-1.78117053010140
O	0.43448248227639	2.57409085193933	-1.30175586242453
P	0.03210947081514	2.57779466372005	0.30930408003591
P	1.69073377538449	1.63964768625766	-1.76304955330137
O	2.68409793678755	1.77573140854734	-0.50079210702717
O	2.28550588424880	2.13202343496638	-3.01050998102899
O	-1.44015515303465	2.84592636246553	0.32143630637509
O	1.03249528611326	3.40473473496600	1.02794919523568
H	2.26891606471953	2.38234142680023	0.14136624234872

27

Coordinates from ORCA-job Tc_Felprot_d_job8 E -82.216580728920

O	-1.45164532176010	-1.06836162060438	0.94943751973845
O	-0.49991028379608	-1.85985000780377	-1.37755991941056
O	-0.24389492089724	-3.29495137835737	0.62031949308397
P	-1.06428800152548	-2.34071718774008	1.78583541614081
P	-0.85115721480442	-3.28882410051435	-0.85533186538902
O	-2.42668243863051	-3.30039541776300	-0.60744257327820
O	-0.37090923378254	-4.40010321129240	-1.68111986990317
O	0.02179485179527	-2.05411266104939	2.77425743070630
O	-2.30312430262645	-3.09594216036981	2.11520301099141
O	-1.90773982448643	0.63468931940430	-1.19893518206254
O	1.51847910575166	-1.18244189530976	0.81518889369327
H	-1.81415298678722	1.64002968159904	-0.85934841086616
H	1.73516924438582	-2.01663474060355	0.38281849306575
H	1.03700583755690	-1.45865412690371	1.66876518579721
H	-2.61487303318858	0.25348181354520	-0.66278084143259
H	-2.56509845165081	-3.19475496690023	0.36216689234616
Tc	-0.16078853866211	-0.29026621275248	-0.34985745101930
O	0.19526763342904	1.24043823891975	0.60094341222844
O	1.18173461996598	0.40602316082682	-1.79302303252748
O	0.74318626816290	2.82087228224561	-1.23661263943612
P	-0.07628905896436	2.78008702032016	0.18450287467082
P	1.89582638403830	1.75097549118233	-1.64461567146247
O	2.79831747083335	1.67288295864204	-0.30952582190059
O	2.67820771358713	2.23104013941978	-2.78378529913032
O	-1.53976382437514	2.87202758111982	-0.19364373500888
O	0.50077896688629	3.72449382601386	1.15112572628693
H	2.22394934253772	1.51346817430445	0.44491797360921

27

Coordinates from ORCA-job Tc_Felprot_d_job9 E -82.216503019400

O	-1.57335271173448	-1.13445159999923	1.08648518893321
O	-0.35675971027025	-1.83879863098320	-1.13173143029534
O	-0.42202374466132	-3.40066604563337	0.79494116753213
P	-1.38801434556065	-2.47701224316434	1.88065480729028
P	-0.78919082753124	-3.29123307723605	-0.75643765153100
O	-2.37922213533093	-3.32501651913172	-0.76973720581576
O	-0.16830961421211	-4.34940676090517	-1.56140884975412
O	-0.46372341763863	-2.31185967922635	3.04926883377319
O	-2.68653063338462	-3.19384967514030	1.96734994315308

O	−1.50752947740881	0.79055022338339	−1.20032665167334
O	1.30998654583340	−1.37859993631144	1.38185010806087
H	−1.02958498397369	1.06984376347877	−2.00963982361752
H	1.50355429098067	−2.20861972774341	0.92884750038242
H	0.70690575924299	−1.66103846056990	2.15325305363664
H	−1.58414523251233	1.67191163390716	−0.67626513335563
H	−2.68066671895649	−3.18354322501928	0.15392093078247
Tc	−0.11496586135293	−0.32377916007300	0.02078727049763
O	0.22959233567934	1.16697576729744	1.02909677127047
O	1.41508165988494	0.33999873101749	−1.23345448066585
O	1.23532604213346	2.76792433924504	−0.58455536806633
P	0.13204736127304	2.72924538747007	0.66926104777995
P	1.31221359921755	1.74806332691629	−1.81869299569738
O	2.80007437837212	2.08331597444289	−2.34654216245500
O	0.31194930194368	1.99958043436340	−2.87486208725040
O	−1.23541740066760	2.95149242670389	0.07957907494744
O	0.66218324590125	3.61568766064968	1.71320744418072
H	3.39992228714298	1.83778508242372	−1.64294930838677

10.2.9 Table 7, SI 2

27
converged

O	−0.536105626602572	−1.82656225875921	2.24566191891226
O	−0.0298962626990633	−2.32088556172036	−0.401740726680225
O	−1.9792953422293	−3.36885857097317	0.841424080814163
P	−2.0130192105748	−2.01961722933605	1.79589110914142
P	−1.0523197026837	−3.51209872732411	−0.473435050088623
O	−1.99681538928352	−3.17140931163751	−1.69558750133691
O	−0.481029734362794	−4.85631703943657	−0.564807062554561
O	−3.07173611442856	−2.19873125699647	2.79157289534293
O	−2.12297375785445	−0.857738474903917	0.749743402767355
O	−2.29029956466229	−0.553428904730836	−1.94017692793704
O	2.33706587674851	−0.622277861788447	0.5578046508757
H	−1.41366836804006	−0.137119329928943	−2.01591892548088
H	2.50293421745686	−0.520649342871812	−0.388814089343411
H	2.44892214459215	0.307446671945277	0.937800395552517
H	−2.48471713290165	−0.487542314740215	−0.988874064553968
H	−2.13265105541309	−2.19303608066537	−1.83700249308798
Re	−0.0328438410221407	−0.663365837764507	0.592921691025925
O	0.0219342476944847	1.01858083947649	1.43130735011905
O	0.236005605270841	0.4398628903178	−1.32806753597101
O	0.471024378686653	2.74951209202504	−0.294502466154721
P	0.9105709944547	2.35359054674099	1.24083500443588
P	0.862296551250839	1.82963346306817	−1.57479292237719
O	2.45560463521712	1.67731183283724	−1.40948323888781
O	0.488197785457382	2.47515568332305	−2.8346769936109
O	0.556376863599402	3.45140177162748	2.14438875119432
O	2.36111645591497	1.87869199679236	1.16660192335749
H	2.62472132823989	1.82295031500247	−0.446173160650645

27
converged

O	−0.932906853691215	−1.25389698119526	1.62842859213153
O	0.303019601033649	−1.96778668333167	−0.763610510512658
O	−2.00282910830703	−2.7459731097312	−0.0961673654504187
P	−2.19830318190943	−1.31899715730906	0.744981974877083
P	−0.918763287654977	−2.77742836360467	−1.29426452607413
O	−1.57875376219819	−1.86546707954273	−2.42280369017563
O	−0.666174594457021	−4.14604669669938	−1.74006899794079
O	−3.53535691698092	−1.35781797057627	1.35445218473178

O −1.87234227638243 −0.244866467024825 −0.336180349405578
O −4.63402928315141 −3.85794952769129 0.81335389737292
O 2.28542194857403 −0.535905296071971 0.856958269931307
H −3.86089382335081 −4.1054579908915 0.298646633745203
H 2.79886038420185 −0.472815683239869 0.0380817344871527
H 2.41474214836912 0.37429206192725 1.306621502552
H −4.40559586051333 −2.95763964407016 1.10669831992964
H −1.76544825460908 −1.0095393932109 −1.99777816498077
Re 0.158047110224704 −0.273922398549606 0.167960601678995
O 0.0666040483576556 1.37413797102346 1.05835225921029
O 1.00766536400885 0.703183813676771 −1.50313630005326
O 1.2647008455017 2.97453129645161 −0.412393644869481
P 1.13671331347029 2.55899768603105 1.17529458620938
P 1.87753255432352 1.97132190699036 −1.53214310223924
O 3.31259311284515 1.59437040526301 −0.888111220049839
O 2.01497582306287 2.63828208951679 −2.82767282216768
O 0.68355370780761 3.72339000102416 1.94092637836477
O 2.46563802347067 1.89509005786115 1.54953240607299
H 3.22072921036353 1.7784131578454 0.0759413621557935
27
converged
O −1.28661722875835 −1.33593706380645 1.2967937040235
O 0.364901271165052 −2.27332837104945 −0.739317633054946
O −1.9729465355207 −3.12788958432208 −0.33213504983257
P −2.3854774526246 −1.62646606123073 0.24075248729731
P −0.70625511213576 −3.24319531830135 −1.3283466833419
O −1.21525296923103 −2.54664963167 −2.67004801663985
O −0.300956744219619 −4.63292937691427 −1.52596589684177
O −3.79553514350617 −1.66943598168417 0.650027120860879
O −1.94155632954395 −0.683900827289399 −0.919194709850417
O −3.20866799534352 −1.79997246954141 3.43190202563124
O 1.97770973046246 −0.476181312904454 0.91918298955087
H −2.31885177490126 −1.56723866120871 3.14321231863858
H 2.61022441107911 −0.487217369402178 0.185863486913618
H 1.98177114165127 0.495404910900664 1.24148718580458
H −3.68934527267752 −1.79719383792733 2.59110697851283
H −1.51695892970332 −1.65880977725801 −2.40775460992186
Re −0.0235148552832008 −0.484717357289366 −0.111849554898315
O −0.35149181686017 1.2546758999419 0.507206590447585
O 1.01985455556541 0.314418595568794 −1.76146840614358
O 0.963458261127786 2.72721468733643 −0.998455476660885
P 0.614654178099509 2.52864643464293 0.597752522343236
P 1.80444211837229 1.63382477402966 −1.85347758378169
O 3.14126397846762 1.470493187011 −0.958094778121565
O 2.10196351244523 2.12291396263349 −3.20058717655094
O −0.0211627101054122 3.74781620163532 1.10361678943538
O 1.90634491204636 2.03334803412243 1.2556649736134
H 2.88740278705008 1.77680630826385 −0.0559735773178175
27
converged
O −0.830678456588861 −1.26499573319891 1.80851850785594
O −0.0922297834085307 −1.71111671289816 −0.840558441683752
O −2.28523337536059 −2.47635246894523 0.152820083232367
P −2.24700169249666 −1.16185403334866 1.19041124957427
P −1.44410422114808 −2.37717820886528 −1.21759730342353
O −2.23877177704689 −1.30700637251773 −2.08738094505093
O −1.37069126416251 −3.68794989880558 −1.87315780020479
O −3.4453808710885 −1.22693625001747 2.02827832033193
O −2.04669010095154 0.0259571969444863 0.195235333089676
O −3.10347618876361 −5.31121755243036 −0.360946994958538
O 2.2314748981023 −0.573631349922579 0.549731642369252

H −3.22788849986449 −4.59691111702763 0.270530688619552
H 2.590070788755 −0.427299205600423 −0.337953668954715
H 2.49422154934655 0.266581164872757 1.06978363950113
H −2.48711734720085 −4.91689928068101 −1.000531292664
H −2.29955288546917 −0.508904400349395 −1.53227884909828
Re 0.0328308059052906 −0.144335944606923 0.306377015096941
O 0.201591054824783 1.38208031668293 1.38337115068727
O 0.626229635270383 0.999557453025885 −1.37025732556826
O 1.20939464986629 3.10567893232397 −0.0900808359507891
P 1.34628554594994 2.50102408150354 1.43415552603231
P 1.54967808040935 2.22853698658675 −1.41347859156969
O 3.0527275532547 1.72215524106364 −1.09613427278423
O 1.4899790092523 3.04733155642267 −2.62522753398886
O 1.10984939714128 3.57702180349676 2.40057153797912
O 2.67791197511085 1.74523177850174 1.47446844117129
H 3.14597151806239 1.7859320173691 −0.116769264817552
27
converged
O −0.31621916141715 −1.58555422992194 2.25084576901673
O −0.0747393263298919 −1.88900340318867 −0.506679443092541
O −1.96295311630353 −2.8740728816649 0.851208483824198
P −1.8300416286044 −1.59200360491268 1.92084307900978
P −1.41487872094752 −2.66219836458087 −0.640844627535014
O −2.44848736987485 −1.63436420487073 −1.29601886977545
O −1.36695214916883 −3.93008462542688 −1.37874239957949
O −2.83123757908581 −1.7759784831649 2.97211057575515
O −1.92340524216594 −0.362087063140863 0.959120505228664
O −3.21996102504691 −3.70929196932017 −3.42760852174164
O 2.37599063982773 −0.593291502470379 0.466431851330529
H −2.55306607055292 −3.98116305281558 −2.77227581228055
H 2.54393041832754 −0.391306638548803 −0.465840348177717
H 2.66620314313474 0.251633994263206 0.963520954999083
H −3.36069630362313 −2.78720471771404 −3.19909639561502
H −2.45897435795633 −0.866288879994509 −0.695623179514011
Re 0.143851998160381 −0.350537241680808 0.665109847603637
O 0.394554151114197 1.15397581581667 1.75762768725957
O 0.302515077490253 0.891380184909047 −1.03935143902196
O 0.958357167973787 3.00085174097069 0.199668223001713
P 1.43487135382802 2.36625792866701 1.64111778097225
P 1.1026571791254 2.19480261449925 −1.20264594083985
O 2.67302974286965 1.80776996810058 −1.20003286707004
O 0.746172525626226 3.0411920883404 −2.34210317149658
O 1.30438172276279 3.38898853646901 2.68250420842418
O 2.80523106918377 1.72832858191792 1.39340760183157
H 2.94926585406207 1.85374941962537 −0.254753537694039
27
converged
O −0.793382146240302 −1.66723994893668 2.26531678212848
O 0.479490922934925 −2.62175779790734 −0.00161463042567091
O −1.73097950118816 −3.51447077626818 0.836661589118505
P −2.07287563160396 −2.00325600103888 1.44846487412359
P −0.653542505068792 −3.63661715442359 −0.356720723792153
O −1.39435037290902 −3.02351414178892 −1.62839404241827
O −0.244101024699171 −5.02436617258674 −0.56882608968236
O −3.38011225787754 −2.05717577803324 2.10399565615568
O −1.84901760796984 −1.09260199175659 0.200559092827123
O −2.8060878951498 0.469280392304364 −1.94233556396235
O 2.29300870012071 −0.685745759744719 1.27261267344013
H −2.02935152386597 1.05205961409743 −1.96296735816734
H 2.73179270051052 −0.512029291664123 0.427590844869105
H 2.29436368076897 0.261916797731615 1.76846966241827

H -2.67578303253291 -0.0238122181721734 -1.1215553617492
H -1.68771579987906 -2.12601838224619 -1.40436863932932
Re 0.184368485851387 -0.830310580424131 0.661531570999969
O -0.0813274225335099 0.943729648018391 1.18344803162755
O 1.07024685253313 -0.0945603751895545 -1.15721250369005
O 1.59068951402048 2.17936821805536 -0.183328718879384
P 0.965482397226387 2.1427507155671 1.3621961940879
P 0.880808978323537 1.39702123354806 -1.39156720369732
O 1.95107065998279 1.73589822350568 -2.56639862786024
O -0.434291632231246 1.9419633524609 -1.77399540100389
O 0.373316925687799 3.44105532236074 1.69846768416553
O 2.10931505532306 1.60338411720422 2.20070852439184
H 1.54836347816675 2.43954872961432 -3.06883830616474
27
Coordinates from ORCA-job Tc_Felprot_m_job2 E -82.229518009190
O -0.92218677020409 -1.24019143883411 1.60016839612996
O 0.26504205514989 -1.90978297550177 -0.76968323045814
O -2.00752076762036 -2.74277469349590 -0.10057661031522
P -2.20726062816597 -1.31918184411947 0.74281450339017
P -0.93257245760204 -2.75063242072349 -1.30728246140034
O -1.61688308123943 -1.85385634102402 -2.43028597684464
O -0.64573150904129 -4.11262056772402 -1.75084469815531
O -3.53163796768283 -1.37382562971716 1.37741469647977
O -1.89942564158915 -0.24168728561585 -0.33600292732780
O -4.61745014298577 -3.89232163102747 0.83787481135413
O 2.26714312491980 -0.52833966179750 0.85014236642513
H -3.84851672063148 -4.12977513647025 0.31231098591240
H 2.79283075322609 -0.46269972930745 0.04062007532216
H 2.39812360564405 0.36854364475470 1.31085432382255
H -4.39585598268303 -2.99118548112556 1.13192336379716
H -1.80745257552400 -0.99902729116814 -2.00448172210091
Tc 0.15164944179875 -0.26395989329634 0.15890097599762
O 0.08378029138580 1.36614435536996 0.99770179359510
O 1.02549014103261 0.70098919539091 -1.47572492610762
O 1.29769566419102 2.98021396303941 -0.41900888054060
P 1.13797806260914 2.55947887372028 1.16263817752012
P 1.90727722053998 1.95601543944089 -1.52302929852909
O 3.33739335673367 1.57989296369361 -0.86416241915039
O 2.05952921944869 2.60490966688295 -2.82589418554232
O 0.64974704604768 3.71201486572570 1.92395901760131
O 2.46109001024802 1.90486460742311 1.56446506929890
H 3.23712424440360 1.77329445037765 0.09708878935730
27
Coordinates from ORCA-job Tc_Felprot_m_job3 E -82.229182671320
O -1.28096623902741 -1.32890362634349 1.26648021285636
O 0.32243481660264 -2.22466551852085 -0.75600144489024
O -1.97858462937244 -3.13496482857256 -0.33742863362475
P -2.39945859799476 -1.63547832621535 0.23248919639058
P -0.71843034920049 -3.22872686130212 -1.34324711618012
O -1.24927204858766 -2.55303392137678 -2.68447588293617
O -0.27646989039618 -4.60821711213958 -1.52970078552530
O -3.80134218687974 -1.69108787564848 0.66653114929088
O -1.97207354525149 -0.69303177468770 -0.92944765227551
O -3.14385124013655 -1.78288979602501 3.45089993195174
O 1.95008450991960 -0.47019320783662 0.91642587394063
H -2.26183418013979 -1.55659172144864 3.13400657821226
H 2.59530492529148 -0.47916347452651 0.19578163378704
H 1.95369768037635 0.49139890906675 1.24835236835330
H -3.64854659291714 -1.79692216548158 2.62522131873307
H -1.55771268043042 -1.66629477136550 -2.42579592348538
Tc -0.03509263871986 -0.48037640202294 -0.12326368323283

O	−0.33588591830687	1.23731928433957	0.44640710953303
O	1.03340517011462	0.31391807783948	−1.72901754386803
O	0.98979283182006	2.73200002806071	−1.00173572520543
P	0.60446404569329	2.52603712354713	0.58354956486048
P	1.83236507953635	1.61980312770300	−1.83297435554015
O	3.15805984243664	1.46209461190867	−0.91788822937139
O	2.15162731020118	2.08969043436086	−3.18170606246420
O	−0.06756903955731	3.72989705505788	1.07798033173510
O	1.88294960905648	2.04570324843879	1.27294341774038
H	2.89230394298702	1.77717947747799	−0.02248562867042

27

Coordinates from ORCA−job Tc_Felprot_m_job4 E −82.229013029900

O	−0.82392079071104	−1.25118247296803	1.78420670645906
O	−0.13491635685793	−1.65132124223678	−0.83045171651706
O	−2.29531342349576	−2.47364898918523	0.15682427459114
P	−2.25734010385082	−1.16415012547920	1.19765864574939
P	−1.46754412107952	−2.34808278583079	−1.22008810527716
O	−2.28814790739122	−1.29177381010869	−2.07809179527548
O	−1.36210038665361	−3.65330244390065	−1.88075364416683
O	−3.43683227376627	−1.24851266695336	2.05980747119203
O	−2.07628735344970	0.02869074973618	0.20971620206728
O	−3.05247240268795	−5.32969502172454	−0.36608151959421
O	2.21003441534070	−0.56850202229362	0.54632080929790
H	−3.19134114120180	−4.62216859078835	0.27001627619211
H	2.58017360726905	−0.42210228448998	−0.33536723259727
H	2.47793019133267	0.25715024686715	1.07424384245727
H	−2.44867320897740	−4.91801846858108	−1.00625478105784
H	−2.34757037294430	−0.49433875314241	−1.52170178699031
Tc	0.02198042575785	−0.13546355559500	0.30350572175852
O	0.20902253398135	1.37844199746987	1.32223124010862
O	0.64065211649963	0.99195520495068	−1.34521284699877
O	1.24038710168762	3.10861380047418	−0.10139517802689
P	1.34792713240788	2.49975607948270	1.42190787494863
P	1.57366410581476	2.20792409149006	−1.41241976345616
O	3.07467744276822	1.69800828039383	−1.08404090662752
O	1.52428935604314	3.00968273056621	−2.63583107486839
O	1.07848052422620	3.56540262812979	2.39059815548110
O	2.67903493040620	1.74901692464874	1.48785269525149
H	3.16360595723319	1.77212049864719	−0.10529954927752

27

Coordinates from ORCA−job Tc_Felprot_m_job5 E −82.228053931760

O	−0.30490120213372	−1.57586245725112	2.22366540650942
O	−0.10515301542219	−1.83615843346308	−0.48916282021945
O	−1.95543352763620	−2.88272294317281	0.85209871652802
P	−1.82747210727067	−1.60627310526999	1.92618870001243
P	−1.42368024705810	−2.64248749132780	−0.64187487895578
O	−2.48306127347369	−1.63428884792461	−1.28073841676148
O	−1.34160477723539	−3.90106646255507	−1.39131049589544
O	−2.80454774317513	−1.81322113484604	2.99541860675413
O	−1.94299858570703	−0.37298960702967	0.97626686903228
O	−3.21732957476886	−3.71236246494252	−3.43147517804452
O	2.36194961652460	−0.58250802920650	0.46529236319263
H	−2.53951960292832	−3.97414614326255	−2.78406561127578
H	2.54110489680903	−0.37755130808934	−0.46302726628226
H	2.65613936544377	0.24887854145802	0.97003842441015
H	−3.38094206194445	−2.79664709863119	−3.19269505195675
H	−2.49775284520182	−0.86925761861113	−0.67634012284477
Tc	0.13919471780535	−0.34483717149748	0.66289326197699
O	0.39118046238314	1.14985279860872	1.69672915652166
O	0.32771585660146	0.88380217670037	−1.01846896937839
O	0.97885877433580	3.00836564822544	0.18582682096162

P 1.42985188740496 2.36697271624053 1.63066632415011
P 1.12540344593168 2.18120912613837 −1.20551536275082
O 2.69869900057135 1.80002578227075 −1.19302057392172
O 0.77261010634490 3.01149032932635 −2.35779064962169
O 1.26484370754565 3.37400585600932 2.68200707275621
O 2.80682927258492 1.73924540857534 1.40721535182846
H 2.96941544607830 1.85303194369010 −0.24692166190211
27
Coordinates from ORCA−job Tc_Felprot_m_job6 E −82.222853774680
O −0.74825567651042 −1.80752830817496 2.35563371869012
O 0.23742062607761 −2.38185364933277 −0.09368085379095
O −1.97297167532491 −3.29748170973891 0.72223090633271
P −2.12816761966555 −1.93098785455154 1.63412122172964
P −0.86007308685433 −3.43520010569002 −0.44167342418597
O −1.54850357242090 −2.95091339660200 −1.77800484054530
O −0.39758869712015 −4.82131518823891 −0.54432862205058
O −3.35979363878392 −2.04061912441865 2.41925506299248
O −1.95263344535967 −0.79680261513864 0.58520980019231
O −2.22573359878192 −0.38858660526406 −2.05729895367405
O 2.33796413686093 −0.79297870407361 1.19909590710045
H −1.56318208468880 0.29847736334609 −2.25741773357567
H 2.64410616542651 −0.91375118837202 0.29032921403285
H 2.46615167181050 0.22703663374900 1.35062709825429
H −2.28345354487451 −0.36995133318195 −1.08485894894188
H −1.77851888554758 −1.98543614970823 −1.86098935430752
Tc 0.13030128018996 −0.76080681064064 0.85225781302952
O 0.04575491538220 0.91177405351780 1.57977430481286
O 0.93675843746807 0.13351722088599 −0.89720970016786
O 0.54520072220021 2.55734930948725 −0.16864129310794
P 0.98458621565433 2.19843303238503 1.39015868111370
P 0.65315938305318 1.53439101971572 −1.39172694763592
O 2.05462319848183 1.96730993884513 −2.09736383745482
O −0.45029801839867 1.76117746326496 −2.35112856811988
O 0.61808639933609 3.35467980325205 2.21851242941277
O 2.41359545664752 1.70717993296455 1.37587971240179
H 1.84086493344353 2.71738696600050 −2.64686278300581
27
Coordinates from ORCA−job Tc_Felprot_m E −82.237010080170
O −0.46419022838727 −1.75815992813479 2.20725284699369
O 0.03738833587549 −2.25643020077859 −0.40046210781654
O −1.86714190772945 −3.35547839580343 0.85434563596165
P −1.94622916327195 −1.99880418168981 1.78847337412339
P −0.98403905361654 −3.43513732180145 −0.49453074141433
O −1.96644930246579 −3.06858486823678 −1.67661715692790
O −0.40784224725774 −4.77214980423745 −0.64788179994690
O −2.98146196340532 −2.19830422001818 2.80467256402718
O −2.09083967643578 −0.86227257722992 0.72915774724173
O −2.37036575927874 −0.47131696008584 −1.92731108139413
O 2.24080605039252 −0.63621515274680 0.59371934004051
H −1.48117778276878 −0.08526235930153 −2.00721739330149
H 2.48640276122196 −0.55240517698369 −0.33707531636559
H 2.38263198428303 0.28906620536240 0.98075495640179
H −2.53930145265768 −0.42893155751719 −0.96995487616397
H −2.13809160684056 −2.09251645521615 −1.80542687085229
Tc −0.01489875979113 −0.65085455479188 0.57376790017813
O −0.04208878166802 1.00112714948982 1.37433353595067
O 0.20034770728586 0.37416575280814 −1.28266832424100
O 0.47666098421996 2.70303826830284 −0.33553080826755
P 0.85745329509845 2.31320889501976 1.21964954542936
P 0.86371932915785 1.73195900018600 −1.57355502353764
O 2.45359798665214 1.55207455167402 −1.37627228632253

O	0.52564337418605	2.34005526739280	−2.86191923774009
O	0.48295008117981	3.42285216517053	2.10069784552479
O	2.31255125980653	1.84261025485763	1.19190106221051
H	2.61336451804091	1.74716620388844	−0.42040331496828

10.2.10 Table 8, SI 2

27
converged

O	−0.22125242761917163214	−1.33229300281317653010	2.01051996956280287776
O	0.18200287959055880260	−1.74933293522803490205	−0.66049044566083658925
O	−1.28472443492059973380	−3.24697620404415898676	0.76924427499832936928
P	−1.62775601627357091594	−1.93851893956700194188	1.70575223010792864642
P	−0.56096684167573651614	−3.13157980128537660747	−0.67121909641874377694
O	−1.72757938671510080653	−2.98077978037193425109	−1.72756516544528282431
O	0.26099908559665147001	−4.31383023688549638308	−0.92068747802681805137
O	−2.49795268541390180772	−2.35269338332960664673	2.80679994759461415299
O	−2.13798714751879792573	−0.89864069013642433603	0.65768422893689060249
O	−2.71515366256200785244	−0.52751071683114525435	−1.93774504391301194417
O	2.59002157223540718789	−1.10191878604109683160	2.39724548506303580808
H	−1.94378170467061051063	0.04939915578899310017	−2.06900061849530558789
H	2.54020195846038632226	−0.35858160789333776153	1.78144620390076258154
H	1.67489815709628353524	−1.40708637754915666385	2.43798719525349083526
H	−2.81554815210446873763	−0.53966382329694484454	−0.97047759175372927398
H	−2.11044449653713206416	−2.06755454994877752029	−1.83370306404677685386
Re	−0.19665112633748504933	−0.14858396577284208040	0.33637748800720124187
O	−0.41288002090322717397	1.54015718018906411224	1.21820390848321258304
O	−0.40421100324189868092	0.89000329710777470460	−1.48418575272465025350
O	0.74309297350954384154	2.92668395338581488474	−0.52523565158197504932
P	1.01479743808179367903	2.14776315391394945209	0.88660761984991731666
P	0.55001173649443790836	2.03839271133083999032	−1.88433449709985612053
O	2.00092114474871696217	1.40789616382347859691	−2.10136025273594428953
O	0.12488895910782990550	2.87889860082773907379	−3.00210046103819694352
O	1.61116381249500029149	3.06601668398374993529	1.85690193884691523429
O	1.77423309988614530397	0.85602952887573835472	0.47719078215506133267
H	2.22905628160030788720	0.98880436605448507326	−1.25195614957943557322

27
converged

O	0.11552012163839533998	−1.62723568737697044106	1.74092898307207111230
O	0.10311798412530120972	−1.85196869842556721686	−1.00556920562377460904
O	−1.19024388571933625869	−3.40720663026965997489	0.52820713191128720876
P	−1.34446852459596155605	−2.16617154590080041032	1.59915108058702393201
P	−0.69250199762217246047	−3.20365769785346365950	−0.99424958593924117611
O	−1.99902644378234040090	−2.93274596089328554172	−1.84400268634665587264
O	0.02981960264992644660	−4.38929122300932927914	−1.45289859679422539429
O	−2.06724989704395145651	−2.64215632554480173155	2.77829350309917444406
O	−1.95602808261153260361	−1.03351077614901298851	0.71340351758926223713
O	−2.85375644408180084710	−0.41669778927621747844	−1.74229731817790578496
O	1.87559701929634492501	−0.08328884756340271256	3.44688876000208255235
H	−2.08381094270329114693	0.14546775234011721789	−1.92990957886965763812
H	1.97333545460870185551	0.72095033210659975609	2.92648612792715123732
H	1.28354523864798375499	−0.65160020486856007338	2.93387886038347733830
H	−2.82846764175518217144	−0.50589918979153924994	−0.77434851104737889571
H	−2.33642164598587420699	−1.99690551552871320418	−1.83332812294084290805
Re	−0.04293205309046638218	−0.33397239087052882356	0.16801510153994747565
O	−0.02222676696132182272	1.26429348900771221231	1.23569413355266588539
O	−0.44656622137693940955	0.91071432461769341593	−1.48119994523048448265
O	0.98030820027858500687	2.76391418282964451691	−0.50756060078850384620
P	1.38803150609520242753	1.80473813645085057189	0.75283497952978029044
P	0.52304081019267845143	2.03961650033441976859	−1.90392883760353215372

O 1.87554144114114507147 1.35109771204815509726 −2.39829620189887959825
O 0.00479930488298460634 3.02179821814248983358 −2.85428022572109085786
O 2.18455519172556833496 2.55272739086387323582 1.73007945465677392960
O 1.97775761745128964009 0.52990954224743080925 0.10323482926521278247
H 2.18813106288073111472 0.83158089661997780517 −1.63332704189414079643
27
converged
O 0.82116743646523593370 −1.74781863870667275584 1.46284338891752208056
O 0.26884922233808000724 −1.80959773404040835665 −1.22427724663462500487
O −0.69453312366660902022 −3.48346955841338523641 0.44059632690372008135
P −0.63038317916333042046 −2.30262701456077856221 1.57691484471058318562
P −0.49259606719275006448 −3.17516769960783307170 −1.13856032688564878796
O −1.94364021362758010447 −2.89381245306666912143 −1.70207401493197263420
O 0.14356688376293408327 −4.31896283373958311813 −1.79047649313847045960
O −1.09417107465177454451 −2.82674834389800011536 2.86539616183835876839
O −1.42211799376625802616 −1.13630129848287708683 0.90356771894671183976
O −2.78885618989827221981 −0.40547727779110431801 −1.29519792194942118257
O −0.49794321522867068808 0.12495963780103741536 3.76362225715643328172
H −2.07443822936070487728 0.18305189505933766592 −1.59006196425808443706
H −0.67595019196457373223 −0.78930313660632878126 3.51699691801878344677
H −0.11616344724538998912 0.53294673540381709564 2.97517220632312184847
H −2.58403409454576138415 −0.54451566800548234504 −0.35517170531169944825
H −2.28267138450736117861 −1.96722618734051990508 −1.57418588392734237758
Re 0.34694240175725299391 −0.35341937278363610986 0.05745062613844645338
O 0.55495289207263442943 1.17435533946193859656 1.22635794062379099856
O −0.38593382507888163158 0.95999951985764764295 −1.39442530884012771963
O 1.15234234767034693370 2.79529256186397256911 −0.59252247822225589946
P 1.83370717936317118024 1.78889603615400050174 0.51003797113387228102
P 0.46534953013876600991 2.14233527076563312619 −1.92103103234157845947
O 1.71337634222929913186 1.52076561913517460667 −2.69860960322261123068
O −0.24328442402651320742 3.15516657786072274305 −2.70118699546897511610
O 2.78975570662000560418 2.52995670759834689889 1.33278952107301362773
O 2.30104339557824166818 0.56657674362839727245 −0.31868053176961824713
H 2.17506331892136195449 0.97464456144859634712 −2.03338437593410326087
27
converged
O −0.60143409619958498880 −1.07840976927255893791 2.24975887829750531566
O 0.20911930471239098539 −1.71588743419805833668 −0.27874972269644437972
O −1.44093034721710178125 −3.12484206931123464912 1.04605239540477290561
P −1.93860901175362965176 −1.75086377541815507719 1.80503566220111966167
P −0.51018441214636545133 −3.10916084503771328329 −0.27376125602098189127
O −1.50514043763615124050 −3.09046303552849144225 −1.50189425557689726354
O 0.37032986736368200820 −4.27624760772923995233 −0.28735275957878098074
O −2.95201608919606206527 −2.09756453819165455954 2.80275655315889116181
O −2.31348775363018610562 −0.82180268416393242870 0.60555807603066558187
O −2.46666286280818747301 −0.68145077529620290413 −2.07342659725514577573
O 2.94488084135318040069 −1.45505217747418225116 0.81847174730354022021
H −1.69403546274030114560 −0.09509524495573717928 −2.13217465564068620054
H 2.56856984832324730661 −0.59495957905440666202 1.04341452702655357321
H 2.20297141265792850717 −1.94899186560195580853 0.45217584780164066238
H −2.71952919095386524972 −0.61698259211989636697 −1.13645801431667625714
H −1.87583735752703328892 −2.19977648573156070455 −1.74612756738971985904
Re −0.35782038796361625987 −0.03110332846391272721 0.52933584480509210390
O −0.73114107694177765673 1.72566306775910094373 1.23303290896010753208
O −0.27945151795009293139 0.85114334776404176885 −1.37140599507115523714
O 0.67992025325384652934 2.98716187818857870084 −0.40909466726407373027
P 0.72898083039355532353 2.31925124800411319725 1.08606018547711702382
P 0.70021946873651552679 2.00085165207385751174 −1.70969473334262622721
O 2.17768386299540361861 1.39788328327481758961 −1.67888609053308490715
O 0.42261040546594580425 2.74721733663455358609 −2.93539971624816864448
O 1.17012412239466656594 3.31557921969220315006 2.06307482147680820717

O 1.54333223234072991481 1.01073608983294205288 0.89141383753138925261
H 2.30693752591512835082 1.02766666802825112903 −0.78981525559293819239
27
converged
O −0.36178845366812473205 −1.06693085137080556635 2.32030712362480118216
O 1.07204604743519382737 −1.84427665235132431931 0.10318425125043628743
O −0.59764880585949264891 −3.35414068159406619785 1.28319886588048492904
P −1.44269563697901403998 −2.01698615761492927589 1.71728549987327006043
P 0.59713036895350779520 −3.33234173330299388027 0.19121089379579636613
O −0.09341858922697107714 −3.64847392611541598839 −1.19657412515907379813
O 1.61502200551904007142 −4.32164459345764750964 0.54349176845112179279
O −2.59562949647759921845 −2.41742654031739334641 2.52426921549168259418
O −1.68063116910372434276 −1.30301907491588764287 0.34165407006932951850
O −3.31489795751372806620 1.02152955856816229918 0.19696048360539447630
O −1.18509026517823179425 −1.50600613494652146862 −2.32215915446432896729
H −3.03260706896677501021 0.10195321800099210741 0.29419836454944425785
H −1.68850063685255102897 −1.37400317629010570997 −1.50292554608491735202
H −0.52048665790353876304 −0.79792809648177809301 −2.29197940064325411669
H −2.51134473354196785522 1.51562100680274580711 0.40199465634765391142
H −0.50273708076568934811 −2.86489762324832275553 −1.65325062646368636798
Re 0.13148290603237272611 −0.20344570239818646207 0.54978021944729671411
O −0.61353060890878785560 1.55874224430911967332 0.90184401484737763965
O 0.50114582788892148013 0.47969045739770332926 −1.39617283454859975045
O 0.98563884516966171478 2.81502491521629316651 −0.56671752424881405297
P 0.77129099138561818538 2.33138133084299781217 0.98524518487051060411
P 1.42197069070517545519 1.69434447712262326036 −1.67203771992876482066
O 2.89422927689676701135 1.29036292834816190833 −1.20595484023393506590
O 1.38589034948584366447 2.24586475170629640274 −3.02472395829738260886
O 0.85364062866344725400 3.48537691417415151918 1.88155478999177838695
O 1.74384381959759005021 1.13831730960202759384 1.15782789545604725134
H 2.80707540091418072947 1.06781183718476468947 −0.25961157382361921275
27
converged
O −0.08493016472877951140 −1.13298054193867070438 2.48327664479650334073
O 1.01406784421092677206 −1.93808220080452486833 0.06138011880015140087
O −0.50652368084621590683 −3.39856532241299369446 1.47779227944346569501
P −1.26458172706358196180 −2.04497189259871614553 2.01488597478088182768
P 0.36606957730243405713 −3.36560447982514077125 0.11282636742956822506
O −0.67463362486683620123 −3.43321843516447877676 −1.07032831893843782822
O 1.29822261733380384108 −4.49381785238161679530 0.10920884118102919258
O −2.31716377252233529660 −2.41089936748805655498 2.96531414718094232796
O −1.63552439143134464850 −1.31875362486813552110 0.68434490821424154294
O −2.86520392626911757361 0.66421807181541980825 −0.64687769995346944274
O −1.44114087807983803913 −1.18972121904755834265 −2.27210189493093439594
H −2.15025136697660057550 1.29001761475712140026 −0.49122557509325442959
H −2.11940496111283804836 −0.68166291760868702454 −1.79522452657027331568
H −0.64145419542935533386 −0.65097696942676797072 −2.15452982292412009713
H −2.66970164273988874726 −0.05647146217627824205 −0.02338084298572478542
H −1.01585814292544074355 −2.56723607614553994694 −1.42744142959803266280
Re 0.21906571020515583359 −0.27638133869175057766 0.68504669113715821460
O −0.48413896464911787554 1.49774228176448631622 1.09268315499180435069
O 0.45181486483787491881 0.47697321815650994825 −1.27922095544691050151
O 1.02151098961676156662 2.78834227744485918521 −0.43805374808343472415
P 0.90931342627306022131 2.25352439842543361692 1.10988777453190579081
P 1.36022813962704014479 1.68925132037923408923 −1.59948334565179139943
O 2.86139570509020346734 1.26759733399822582811 −1.26035033930748996411
O 1.21834449799453614638 2.27669101066728618576 −2.93016263798666454221
O 1.06358030311101470211 3.37828916025565462178 2.03469208231051190694
O 1.88570968260831306473 1.05173150800144266093 1.17526179878361469733
H 2.84058809500660602865 1.01946550449212947065 −0.31631965245518700591
27

converged			
O	-0.50084759228664688102	-0.91666441521144048643	1.98369073151901953445
O	0.67424765877343928455	-1.46207851738807326214	-0.43912104361131459873
O	-1.42691304531603835315	-2.63341783451541866512	0.38095815656173098729
P	-1.80145704534041639633	-1.25876902916958988143	1.20703772829259103894
P	-0.25417011194727245904	-2.68581407109425507329	-0.73552477662925119795
O	-0.96801219422353823596	-2.36734172389813490867	-2.10893451240703155847
O	0.37299022660842795496	-4.00660219951218632417	-0.72649837362506697325
O	-3.04913473449439642593	-1.50550458963869493445	1.94404167190160559464
O	-1.76628649609972865342	-0.16902208939146173838	0.09478595985143210834
O	-3.72082853703193050521	-4.17970777482289879146	1.53610973452023569230
O	-1.33672608401282433377	0.22437029403240094094	-2.53431870389760849349
H	-3.63700574489315053839	-3.24087899422218228551	1.78032993049587306977
H	-1.76323508353872160370	0.25994036028586076092	-1.66126336558011478139
H	-0.47677405127239858018	0.64833485222354625410	-2.37583246718102358841
H	-2.94234371463095989441	-4.31668071015386356493	0.98927456961015935999
H	-1.11150766101996656410	-1.40184255237866306487	-2.30691277554120821591
Re	0.28759895887652098256	0.20929919730199908479	0.46758178868471800271
O	0.10282991159939733339	1.92934350491774342373	1.30847093999530295072
O	0.89719846303573713708	1.23397213094394597732	-1.26496822708686806891
O	2.02545593862444928135	3.02087249127590107278	0.12271144255196560213
P	1.65065352692130584700	2.22249639150227462636	1.50682437425461301927
P	2.13707003759261882081	2.16145488595517143438	-1.26225833730118108811
O	3.41955794026401704855	1.24558884841038142177	-1.00755554408363945385
O	2.28920919238868370016	3.04441723915737672002	-2.41741363424702093710
O	2.05328242276137995503	3.02408977869451556231	2.66396595849516648613
O	2.23181567261442470596	0.79997248261613407294	1.30417900760441507479
H	3.25273214374871422194	0.81467204895027056644	-0.14746024478321340112
27			
converged			
O	0.14439030387540607858	-0.32368680943582694987	2.16454624454594535266
O	0.66633853797599251756	-1.14557248225477081860	-0.40622627886773027273
O	-0.45018079396737831210	-2.62652288092201757763	1.33418353900598596340
P	-1.08803182583958002461	-1.26786101935378470174	2.02329690150303420637
P	0.22587694918023049073	-2.61516019747023165110	-0.13353713705001099554
O	-0.93637895600918497063	-2.93495913206627312420	-1.15193515400446977459
O	1.28273142581752330749	-3.63318292529506470245	-0.17866448062223103110
O	-1.84757517894754919041	-1.66732483667327779919	3.20925714847749610215
O	-1.81177761635750922409	-0.59237551597300264206	0.81636722413863116454
O	-2.39682948583338761850	-0.82079956827207078973	-1.80000908803466685981
O	0.96411690269057537250	-5.14018897691316745124	2.17961307365300749339
H	-1.76267561771214942290	-0.12721786545158855763	-2.04813413970018887156
H	0.34038086106406062470	-4.49602879293244761527	2.52633741883512286108
H	1.20554123097818743915	-4.75405944964582083401	1.32068473253909601794
H	-2.51729670634977198418	-0.67691615505908531158	-0.84591430404903378282
H	-1.49704296212986820436	-2.15929292967437103101	-1.42789939197520854286
Re	-0.07072618079891024312	0.51169340087630188485	0.32073352024763718271
O	-0.66954038812138882975	2.25508348238975875333	0.88494251956267033776
O	-0.45353770438769802631	1.13024896949136377522	-1.65003212986149727648
O	0.22261946932696591306	3.50320549257673219401	-1.10035665057031795122
P	0.61876927842522944090	3.06370970468339098858	0.43108176115779567317
P	0.25605752438453072140	2.36068524106664945350	-2.26714711966856441450
O	1.80965636684237374254	2.01117915949816872256	-2.37632442716941882921
O	-0.30198521921753379571	2.87104942309063160266	-3.51829156323144820462
O	0.99086443194149165237	4.25033823048173253767	1.20391371490306986480
O	1.62844195400079438940	1.90142542557270055603	0.25194225409917220260
H	2.08719340216144644273	1.81703100195248112847	-1.46052818891605507012
27			
converged			
O	0.47899035880825191480	-2.22274629095462605832	1.85353322129716779898
O	0.27254469990823682046	-2.12068137912607568296	-0.89340194528017446984

O	−1.06331862906041285655	−3.73036045631612189766	0.55197279020271516892
P	−1.02941039716243332158	−2.61227257945905710912	1.76256280090950001593
P	−0.64423161278291707355	−3.39046874601404546468	−0.97043895042240824189
O	−1.97196343486320735394	−2.91776280482524574822	−1.68750573221279220881
O	−0.05988164155374331443	−4.57094250251385059158	−1.60700371698057642433
O	−1.71426135457272965645	−3.16228165443953335156	2.93378857759191902232
O	−1.58811966922128022794	−1.33452499112787936753	1.05743993404997316254
O	−2.56151762996002796413	−0.36856105894290841496	−1.26744798873108832638
O	−1.42759776971437002402	2.67715201921716516864	0.79375026146025751128
H	−2.51621755347121345281	−0.53439339579829603366	−0.31126028460363025507
H	−1.03335830866625100199	2.75055746969907932709	−0.08064219549154585942
H	−0.89154480896464272099	2.01380701541224782503	1.24872920149511301702
H	−1.75068129127388916189	0.13534840690362343052	−1.44233786799174446003
H	−2.21666207137939208494	−1.96165493781468813772	−1.54918177871707518634
Re	0.35185089104276745520	−0.74881304776517998434	0.46871177802642283172
O	0.56600427444552314782	0.75836890435183113013	1.67825337557385267928
O	−0.01079646592767258273	0.69664895470901155861	−1.02170895406226636126
O	1.63979239991631575712	2.30794054966230355319	0.03232131978261906791
P	2.01158719122185880579	1.19417821248014499602	1.18192775660963644846
P	1.03557817339923685651	1.77710612481951568675	−1.38634569701024124022
O	2.28436402918204439771	1.03150747197620762385	−2.04204013245960425138
O	0.53624681010752361932	2.89670906364466196337	−2.18449741194391222621
O	2.92301570362231322164	1.77998236552332067539	2.16505300418687696151
O	2.43211617951723102493	−0.05643914672679822830	0.37347223810697754987
H	2.58687193568754825179	0.40709642771230619696	−1.35580359914637638141

27
converged

O	1.32484809046770535268	−1.56575866493770576859	1.34983095881651604664
O	0.44971805397222619494	−1.64293257123839731015	−1.24601681051936652445
O	−0.22657901783675721274	−3.36621663575129748125	0.49799066955851395422
P	−0.06725389437977391527	−2.19545384064004434066	1.63116068820904613901
P	−0.18704555153702495662	−3.06435837650328934956	−1.09354388803573909783
O	−1.70043583081886939823	−2.90421881784917079727	−1.52342307981291824603
O	0.47994931657943940007	−4.15899330859949678540	−1.79671309673885959413
O	−0.35289058874992551562	−2.77730051138158717805	2.95020937462569809995
O	−1.00084092280012360021	−1.05927652389025195312	1.09732039019778482292
O	−2.64241052513847796135	−0.48704536818002153176	−0.97373435069365255057
O	−2.27429624154490594989	−0.91952136892841795923	3.81132082247790737739
H	−2.01367645241918635790	0.15027170026678093873	−1.35141001224722323570
H	−1.66520618097680594971	−1.66899794973174131485	3.71083958722317364121
H	−2.10322012650736134276	−0.41414271183186390779	3.01107160238680870989
H	−2.31266640660788747041	−0.61162212202883325940	−0.06777982702547405425
H	−2.09445640949729661173	−2.00692615258195905525	−1.34760821712639211611
Re	0.60399685904167699224	−0.18365921846189600664	0.02187136376197486298
O	0.83983686039016214320	1.37929878860244725658	1.11466812337433829327
O	−0.38051701655663228152	1.05481418503079016347	−1.36296445203262139856
O	1.13446554137197752610	2.99334058411483416862	−0.78360979907149685975
P	1.99137945493384038720	2.05222072588379766955	0.25169228478055849507
P	0.33264805304693101373	2.26784274093227145030	−2.00856312307256779093
O	1.51534179223618048482	1.69977582610591326784	−2.91808297018441509252
O	−0.52026708380822317412	3.22119370080912270637	−2.71611320008606949017
O	2.98639730962701399619	2.86570116189480250668	0.95256448034391172364
O	2.43967012811488492829	0.84102954611140257057	−0.60494965502685105196
H	2.08291080297365960305	1.19543517177915026295	−2.30412787042653155467

27
converged

O	−0.17518613550740846918	−1.75556442835876480579	2.64034867684695306878
O	0.50283867109917346205	−2.14989276028628717441	0.00329601384645303308
O	−1.23252441519201583198	−3.55041694518148265303	1.22188138401577828240
P	−1.57882803204422961230	−2.23452813616263679108	2.15291334529627187422
P	−0.35648090340447763369	−3.45473541040908704858	−0.13138049187539146967

O −1.39174711990886668112 −3.16741719584705894164 −1.29444414158802301174
O 0.38056015553861255452 −4.69994158517268001418 −0.34261195522415072157
O −2.60725056113795705670 −2.60098259488757532765 3.12818812557483028058
O −1.87456367960975778253 −1.13662890231801783081 1.07994844262366385124
O −2.03356407988773790763 −0.58116608854912032989 −1.56204067489006992453
O −1.37075304622873206384 2.07066123055643425133 −3.40439883328849690614
H −1.16012038726747856998 −0.15409740283952677586 −1.49904297405533992738
H −0.50643880180040612426 2.27256276236783438804 −3.00696794627941432765
H −1.69173321223741268682 1.30674485250972316486 −2.91601355331531486570
H −2.32001974623448825596 −0.61520068851358222783 −0.63459643885062877455
H −1.64759051875488915506 −2.21766855163264153816 −1.42024249018673853762
Re 0.15415586337186537591 −0.54988722041205084778 1.03540311411103624906
O −0.02834235117041990215 1.14685316309652174382 1.92509777274052318319
O 0.28622868971136289362 0.54224865226525120754 −0.76896603694370102566
O 1.45789833692246184604 2.45984494962875777091 0.39084913820877759116
P 1.48276809092099415288 1.61118586545306108349 1.80258139083164925509
P 1.37031207457374781811 1.61747435830046026872 −0.99533370363112816381
O 2.78202544999743750509 0.88249088464690239597 −1.06202469723016301728
O 1.13690462738405795307 2.50519622910725736631 −2.13898267194624969179
O 2.03326393228223256671 2.45110906236207926057 2.86757855203416456291
O 2.16521666970506609928 0.27112591424937676488 1.43407106557480434894
H 2.86237042128862428925 0.42512998560573650453 −0.20321040286872599223
27
converged
O −0.51066503259890838606 −0.05482889146845376649 2.55814578139448345340
O 0.73741550559152546640 −1.00707386390841513446 0.30214589368974720252
O −0.69061844961957652433 −2.46010636640112556606 1.82581370414373611588
P −1.58732986853499724589 −1.12249054534035952457 2.19308334842960750422
P 0.34535045443552458977 −2.48520773112314818931 0.59561549479271580498
O −0.47991332402951170089 −2.98585440469110086070 −0.66177329028696429969
O 1.45059862809091666414 −3.40382021692296055093 0.89779872152627171467
O −2.61169607926802216369 −1.49490568643924537362 3.16982496593734008883
O −2.00796864947259434686 −0.60898551164162806337 0.77868603399618574468
O −1.85706315592088611055 −1.07644967677395486660 −1.86289586552924646412
O 1.13072513991549694623 −5.56096867989023913026 −0.80498790407887010900
H −1.23250079735726281172 −0.34472171587594885311 −2.00291174313241127081
H 1.40845088702007315007 −4.91855638476442891260 −0.12760613994205652344
H 0.38492889961284570965 −5.11006925088028474136 −1.20865487324197684593
H −2.24156132730470902814 −0.87326686220280658191 −0.99299307639575107309
H −1.00114836957981689913 −2.28385178197898275343 −1.14169207977339381976
Re −0.28795901011263519420 0.62083272649272014387 0.65303559324963766919
O −1.14282032166826286179 2.33570901628751492751 0.87437719649279266321
O −0.17581482898180689212 1.05848109344431717815 −1.39854430027625697974
O 0.15462556814935521365 3.51851939018584136676 −0.91712748045366487837
P 0.15748119710139710237 3.22932668643195874836 0.69888076003080379373
P 0.58112766362342282989 2.30425068892827145106 −1.92292589020401050171
O 2.12843022916913104936 2.09694716845546391681 −1.59124477985245160738
O 0.34074138379262108867 2.66911147864686348896 −3.31789676977500480604
O 0.22064108591237943657 4.49794069656726058781 1.42729934033452465769
O 1.26064783456697671049 2.15921937138441721871 0.89939970614329944887
H 2.16529475104376745165 1.99531924776556657264 −0.62095234297949020164
27
converged
O 0.52974611933061310776 −1.72616219169023499269 2.06658943406564921119
O 0.50990238845106672372 −1.79084973476452313790 −0.68871855222510913741
O −0.88045468190056674906 −3.36085997780140210978 0.75848069727541811424
P −0.94858975525852839983 −2.16745580003191218310 1.89224524205213739947
P −0.15664075989301279312 −3.20800493423299259987 −0.68101220856737620934
O −1.35171265642216065217 −3.14675329329696973346 −1.71301350117380524196
O 0.74689901012685189219 −4.32535529226122505975 −0.95299468969999368007
O −1.72144012425182268977 −2.65662854322384411887 3.03569164920168699240

O −1.50127358716752579149 −0.95263830173620123176 1.06855140030795103l0
O −3.14717469018526241342 −1.34580612429756207860 −1.01738455878033251523
O −2.41393793478138984909 1.26552365629952712212 −0.71340073979029261508
H −2.96677541088370100297 −0.41969002710342745432 −1.27029251298772183354
H −1.50693263288612255835 1.35229362686348975053 −1.05266122631822889311
H −2.28009392500936014514 0.92543672288366640721 0.17831821656312324409
H −2.74390459259216834553 −1.38285057681516376782 −0.12855099901938729645
H −2.03512340752037523828 −2.44689886080758167708 −1.52774372794067847536
Re 0.48243114102179707192 −0.33392201256249248731 0.57486326783920249639
O 0.57135709144447344165 1.22602264329472565585 1.70307161478968693835
O 0.27579980443642404531 1.01097163970271020084 −1.04435138416302208952
O 1.79352309568247991756 2.69422293971909443400 0.07327045990859035185
P 2.05138436626122944162 1.65648669465043529847 1.31822551301482948638
P 1.36414853817719028406 2.06183866924314118663 −1.37285864493371190242
O 2.67774385455640606679 1.27612881686240964285 −1.82152405259796990045
O 0.98368996809040054607 3.11619695765263848841 −2.31134293477811691986
O 2.85772962427926557893 2.31239938290359337003 2.34969864521678450942
O 2.55638324194898736152 0.35935829625957421696 0.64032875032748504118
H 2.89271591793770754819 0.70149561328586740316 −1.06158890894536384053
27
converged
O 1.23838980504102025293 −1.17948293162587236615 2.07621493517797839345
O 0.81805777301934801127 −1.59920380867945399750 −0.62130604658112342786
O 0.05608384618054708393 −3.21896373372866761997 1.19560373026775512706
P −0.12012406965832439498 −1.92870799273710180621 2.20508021794816766459
P 0.24093416475954027023 −3.03727508496178666419 −0.39851008801549098193
O −1.23940898363139218041 −3.00129765513515778252 −0.96300139097478609784
O 1.03087092903074495354 −4.14750825850083693069 −0.93409602014680026549
O −0.57985146213423066630 −2.40875025049032931435 3.50985408482387883922
O −1.03513040266998435079 −0.97091762585350327441 1.37024655508275117199
O −2.20407632568613953694 −0.68528803930339110551 −1.69058632158304922299
O −3.53166791552185621583 −1.53248983396767424203 0.60949184375972853545
H −2.76209824034359385792 −0.64517491915258573520 −0.89408380296473244364
H −3.29480591503367925554 −2.40727024972606207243 0.28760730576438603423
H −2.71268347635252515815 −1.22911566734847421678 1.05351626612863991106
H −1.50221726674885158737 −0.02030992329350372883 −1.56837489686068165540
H −1.54535609819219921235 −2.09991567394788436474 −1.28201128381831486180
Re 0.64637822101212361048 −0.01962205694402496814 0.50945392847379666890
O 0.59582153338703780499 1.65036186775339399624 1.48765503293004042185
O −0.16862787947077334527 1.03563580393256149392 −1.11035498677919042265
O 1.10337719507016518428 3.12604239456576538103 −0.47827983125537393283
P 1.82919387076352291110 2.33009943443470479707 0.76461456485855128218
P 0.56124219829410837423 2.25012049995565366345 −1.74194134280940926551
O 1.90852637459503138295 1.70160172237066653622 −2.39956480515856140912
O −0.22396274054952361454 3.07608886453139973582 −2.65697881006180258723
O 2.64834145169675716502 3.26856803428341669004 1.53500853926212976752
O 2.48508240364300769798 1.09471351070057987265 0.09573286235595367122
H 2.39711101778478452573 1.29256156186351200965 −1.65909024616839095856
27
converged
O 1.00657177096108507719 −1.28617581175298933971 2.43983628797940355071
O 0.75636584544652574191 −1.79709539015828601016 −0.26042890148841274511
O −0.11246361119565084774 −3.36293316844940815002 1.55618560045136278269
P −0.35756787201268414034 −2.03717459519951260560 2.49830106197261958556
P 0.32863884166249351937 −3.28388199157386928562 0.00041801519684428468
O −1.00251001865154609760 −3.52171543635499162406 −0.81179527262958339762
O 1.34527785580516479058 −4.29037853174679817414 −0.30586283472543263073
O −0.90379532733273415523 −2.46957276873682562979 3.78619278042501639092
O −1.20916257061712517640 −1.10693815837375719724 1.56761142548959830911
O −2.33697999789366672729 0.31981335678336175743 −2.42798958267725861759
O −2.86535622355939079142 −1.69409027918485888264 −0.47930920173328583900

H	−2.38787844947832272879	1.20575655487446065095	−2.79469798588895201874
H	−2.38395415796568865829	−1.37464735355224898505	0.31028433727244236762
H	−2.73841042805285983519	−1.01739321036027119405	−1.16201183412662478744
H	−1.50354722597738477141	0.35896535984552935261	−1.91928165615692192247
H	−1.72775391920366128140	−2.84268296520931329496	−0.69715289400008684240
Re	0.52348529612501193409	−0.19012083638106994088	0.79358220287465930376
O	0.37563367931899760421	1.51892353206259289600	1.69332159653931602072
O	−0.15881857585199654626	0.77643501032287720598	−0.93747199658467539329
O	0.95721190401254219182	2.95036315981396857211	−0.28555512835822138840
P	1.63285023357904912622	2.20901024141995705463	1.02604868910658408865
P	0.54452353986389501195	2.01366953328558384584	−1.54688619915901282020
O	1.95556333572282814437	1.53291990093419450147	−2.11505443297934681013
O	−0.24766542582047695875	2.75547752296187731247	−2.53137148579220649935
O	2.37463025249717407661	3.20025889832165422177	1.80858977161915857046
O	2.36425394385287912513	0.97742600566424586717	0.43650384391964042186
H	2.41025731305021206907	1.15028141503100633791	−1.34010621818234532121
27			
converged			
O	0.62796768500321253903	−1.86824305086512243435	2.24948066947944713689
O	0.62429077149409195613	−2.28261090483519923922	−0.49697700094279267935
O	−0.83900247281277162692	−3.57610090410219427781	1.14424333244420650679
P	−0.88685989320865399321	−2.23159687776349491273	2.09343072603758706407
P	−0.37515747885591976818	−3.49465455594555685082	−0.40124799677935696351
O	−1.65932791494532705379	−3.07711193420510387142	−1.21181351045507179620
O	0.16211243629176746994	−4.79177441771100642853	−0.82003893210077682951
O	−1.68965016864111738926	−2.51056844207151019788	3.28614731370644985731
O	−1.31621568626760665488	−1.11925000130993645975	1.09233270793106762575
O	−1.86572683601885480620	−0.57741831558191403229	−1.94279582451685661759
O	−2.62918115358596438114	1.06876759497010120903	0.05727338582509475251
H	−0.94283634087412382385	−0.28962399083953976886	−1.85218719366509376734
H	−1.91353822212895297028	1.70321225542916931950	−0.12666298294459144014
H	−2.22744035288148811702	0.39715506411580403512	0.63192050494409157668
H	−2.33248441135362538645	−0.03841687135089483229	−1.26357668888624186110
H	−1.76718360425169684902	−2.09853602034242214813	−1.41140535971297431672
Re	0.69368646724556604077	−0.69442600037275159508	0.60514831295662219190
O	0.78564071411273261880	1.0077595910715667138	1.49440718404377537176
O	0.60419943293279310126	0.43700538672628141956	−1.23492278240444264270
O	2.04150807114374588380	2.29738047199997552639	−0.22431567282624359505
P	2.29040424301263945139	1.35398645611999768334	1.11470047550876971698
P	0.72145753350763630607	1.95906697217082359508	−1.08842922799454555616
O	1.22103061554116343324	2.45172576515410156617	−2.54295019389474097338
O	−0.46873069947036705152	2.71093750135796085488	−0.67100735482541962185
O	3.10192100773448897755	2.10392830148497189668	2.07718057042742509921
O	2.76962697980160221789	0.00280612785830891553	0.57026322111156435568
H	1.90888928575970506785	1.85108443038087755461	−2.82629768351913046587
27			
converged			
O	0.19636676319188423046	−1.63436548528830893900	1.82402438459742488597
O	−0.18753862518392275249	−1.79926365789979825216	−0.92897012775221066150
O	−1.17509166567779410606	−3.43543696801408948716	0.76671432453282306607
P	−1.27265490400166303431	−2.18345261685137348451	1.84965975283429640008
P	−1.00801006827237427110	−3.13343874763557384000	−0.79606947262531191356
O	−2.47874672416328101221	−2.78145373108538418450	−1.28907270286888553379
O	−0.43066406286376596180	−4.28986857782999031485	−1.48817230919268950196
O	−1.84172289745758321899	−2.68346177357643256300	3.10108432756158602928
O	−1.98236829270752279442	−1.06578633543298773034	1.03786981550721968404
O	−2.11979253584603455351	−0.57455759613640244865	−2.78177060922898888862
O	2.20627694235521953914	−0.21668842901377088905	3.35493828336433264070
H	−1.29032382500592968100	−0.95039509081106876565	−3.08909801633736247339
H	1.54838540150988301924	−0.74737693553737116758	2.88333808755186860040
H	2.25146997901665502084	0.61767494979893544649	2.87586366925720993137

H	−1.83496600860077263562	0.13942693375404918710	−2.17722562007879627544
H	−2.50724666213866154152	−1.90708056994143571039	−1.73756300760857596011
Re	−0.16430273501817704851	−0.31117707586690446231	0.33706754046607123598
O	0.03694382895240132247	1.24603799872222253953	1.48602863230310355824
O	−0.75770501638755793916	1.05273770716848602547	−1.15188689019413725845
O	0.85082748444404088239	2.81527563762440635742	−0.30294906673044103007
P	1.38141257524650495903	1.77582079101407308563	0.85454329898229830498
P	0.18836128668875906644	2.17889752453012430777	−1.64759659354306564616
O	1.42944706363015994199	1.45741060462916438567	−2.35269418926846540785
O	−0.41572015880821050482	3.21094528957463509755	−2.48742228447932012259
O	2.32418811346031617404	2.45902580553214011871	1.74550878408684750909
O	1.85120377844825401148	0.53140003916531275419	0.05982030499031910920
H	1.84137097347384348645	0.92365030369445566549	−1.64407031188755370366
27			
converged			
O	1.52747827862957619161	−1.19653441103130608880	1.82632246954528154603
O	0.51614057341034524917	−1.54500909819105469722	−0.71751914709306274798
O	0.16572598847452596482	−3.20595947632640232428	1.19460994809810783224
P	0.21212720187934691607	−1.92953121153316842218	2.25223322471964149116
P	−0.06855683909924123465	−2.95330025217724667641	−0.36835154615655540944
O	−1.65492237640429062928	−2.79714950004837525910	−0.49215420741576976216
O	0.46092529088002631710	−4.06471854227890005973	−1.16450253934777170350
O	0.07860789880165239274	−2.44032937407471095526	3.61674817563755768290
O	−0.84182852175348699131	−0.94618124382982327081	1.66489879128630957617
O	−1.81305514052513383483	−0.75831520984769817950	−2.21376943897013278573
O	−3.48982017387017684484	−1.93134724689195680369	1.60053190049513105464
H	−0.97961228993614557492	−1.03832325847257078699	−2.60312525634786462447
H	−2.70666398461872326209	−1.41737680012285838238	1.84777029792633551430
H	−3.14479869551066926192	−2.50361418848564909467	0.90656233806637942507
H	−1.56364952593611272746	0.02878377926738712178	−1.69044146413183127287
H	−1.86885176864171187461	−2.01498004722451362625	−1.05500991457860227030
Re	0.63327111945269587423	0.02079883962196138469	0.50081107750492892539
O	0.84446630013936085923	1.67551898911357111110	1.51993149575703823118
O	−0.47232909123324634537	1.14739524100331702350	−0.87147739915383248821
O	0.97394113596304299207	3.18576534170578451466	−0.48726100854239262627
P	1.91552137719499482316	2.33690946447639458228	0.57122909142838917962
P	0.15343491375490975193	2.35963386206041825233	−1.61744388947215700369
O	1.30996829609430864849	1.76679315778801293568	−2.55037061213685500860
O	−0.77556663024017447050	3.21919630680227975006	−2.34787421159491405120
O	2.91091708121505332585	3.23527215377081489578	1.15937775578527069165
O	2.37461355287461906016	1.10416184471536804601	−0.25401940661053928894
H	1.94191603199755036258	1.35694085862272495469	−1.92580654691735064787
27			
converged			
O	0.57687477569104628206	−1.89475123032530756717	2.28644834005484032247
O	0.62542936334142462673	−2.37274005099784490724	−0.44451503576554785013
O	−0.92738867690169290992	−3.57295336445727818031	1.18268697264141642300
P	−0.94630864943839843040	−2.20794421491948167358	2.10136608561114046623
P	−0.42330339172739089859	−3.54248211398306400710	−0.35330864068873624184
O	−1.66976057153279522360	−3.08858751194844627364	−1.20296207640444463571
O	0.06711811444011618610	−4.86975014787514126624	−0.73157158136415734173
O	−1.78713813446604974366	−2.42879421102505110852	3.27996897522733821262
O	−1.31134002137059146875	−1.10334666329423636277	1.06573899570982399965
O	−1.77402960761607331186	−0.59503858686848110082	−1.98764404910265968240
O	−2.54555066663276097216	1.08876196628076837847	−0.01633509486225415835
H	−0.83940992436670136545	−0.34766375717622005626	−1.88999058902224814638
H	−1.79301651449969257079	1.68935167770905003692	−0.16961188132910637316
H	−2.19260130203643566205	0.40261458101218744465	0.57365968654032017948
H	−2.22639865631629518816	−0.03366810436576678223	−1.31674109437527020461
H	−1.73269063964107616904	−2.11161040500406205211	−1.42600389488931456050
Re	0.72843235450791676833	−0.76092001221692839863	0.61486544790126829163

O	0.86714638148050005029	0.94873029518430995743	1.47619285259450161440
O	0.71345026490802987418	0.33134460874845000911	−1.25504421892881357081
O	2.18847953443304632515	2.16161394395586459183	−0.24453869041100700921
P	2.39184461794503810950	1.23377886873565367765	1.11708163948919625419
P	0.86697338895679088022	1.83795789283184740626	−1.09207798118616428162
O	1.33236099023570098154	2.38001508407340622853	−2.54566183366055698301
O	−0.31743573494814686375	2.61632264781066403714	−0.66917266914709905290
O	3.20488168529612549307	1.98678724323517652550	2.07607838731855620651
O	2.82888931510351859799	−0.14665488128369277843	0.62292353455305782273
H	0.73389169756420447044	3.09412645632605576296	−2.74993157697270929063
27			
converged			
O	−1.18683337842767722137	−2.04286433479743001485	2.27716182162423264046
O	−0.26328590267444829109	−2.41555830788089576089	−0.28963661304642596139
O	−2.32293129188569347221	−3.58408633936794585040	0.63426151523627161133
P	−2.57697968203703631218	−2.27236291360261422057	1.60260934895779394438
P	−1.26558173354896608132	−3.58276499684175497862	−0.58772433698678594460
O	−2.08777112288021626796	−3.12756100374544843135	−1.86282874246779228677
O	−0.68846466816740858885	−4.91635642537781070160	−0.75620152397628859209
O	−3.75597686916732831364	−2.52539282122602903868	2.43451715530942047749
O	−2.58130586710062459588	−1.09251497347163417651	0.58157383854731348194
O	−2.39286108424876031719	−0.48980447471818944205	−2.02901638925633420740
O	3.59264238771025024022	3.21416825296876895734	−0.86631337215490589188
H	−1.50096491273991494708	−0.12019264248278413443	−1.92733782922992591402
H	3.25685308877203549827	3.13722623922286869558	0.03251411965520889513
H	2.78546127371644169912	3.14153882793590533140	−1.40715346035045807405
H	−2.71793972532150984733	−0.52931599473150325874	−1.11214581173963544813
H	−2.21313360640267342561	−2.14597888036411443835	−1.95947308718352863899
Re	−0.47930519221596112889	−0.83352510416076830424	0.79873918589399539592
O	−0.54253662265262481146	0.83992703077237740317	1.75730451021453037974
O	0.01977262036952674717	0.33819893092910474630	−0.92669245997391114233
O	1.32881803829746436563	1.97975245650577980072	0.53853389569046961061
P	1.03082411833314679939	1.04532616784539178489	1.86983852522577764788
P	1.18066061801660460162	1.31873614099450509052	−0.93495095946333239834
O	2.52551218992785209139	0.46108669140994179569	−1.10277387454743536210
O	1.14029820071965959372	2.41910311342055939221	−1.92304434702517057509
O	1.55395639306628519094	1.76157715716730578492	3.03820793855550697060
O	1.53114664277142042970	−0.35471409888384297648	1.50104391320234720197
H	3.26932608547128555898	1.07085230205914072599	−1.07911295647133931297
27			
converged			
O	−0.98327796954313495537	−1.23522164279428681155	2.52448591965806334869
O	1.11910780846110857745	−2.13006292507307426476	0.99199728165786615897
O	−0.74473338997230653646	−3.64427351278448297123	1.82760310958884053534
P	−1.76632997934838842369	−2.34973189033359908251	1.75185349658249900351
P	0.71947556758129427301	−3.64009364304809679425	1.14612903717746594801
O	0.51253002374603340296	−4.18374426819329414684	−0.32467138015163266029
O	1.63705467353342681847	−4.47895754269567536454	1.91791480656363622082
O	−3.08822369228038784428	−2.76751946224177558520	2.22242199659155481584
O	−1.57049448589127704778	−1.84822672730704695709	0.29056203863859103587
O	−1.69453767207574479414	3.88454092429134156816	−1.02365363591446212332
O	−0.30568465065703531724	−2.33514182237792944363	−2.03259478779510782687
H	−1.44557418041092100580	3.06617983345003475293	−1.47991022326996257696
H	0.28588504996726671337	−1.57858977760446617111	−1.87509429514032199826
H	−1.04986763365279700544	−2.14754665930870514146	−1.43571027063344702768
H	−1.29105750042172617853	3.79523142181128925188	−0.15302302249851196247
H	0.20869957493206994781	−3.50630352337066319990	−0.99046300605744352907
Re	0.00398751644269554339	−0.55690413798287363711	0.88912417232660456712
O	−0.91340103015467133396	1.09995999153359891309	0.62137448047195320555
O	0.98267217480373969529	−0.11892828454662832116	−1.00390802704048498484
O	0.97569394119841557078	2.36962738785605120384	−0.41487574680121103166

P 0.28225009125509681329 2.06255174150415854228 1.05025999963512228774
P 0.69107941463959308 81 1.26760867952660039037 −1.55669471407455839440
O 1.92668084917640158871 1.56917990143587360308 −2.55812112249975420752
O −0.57391477204427021608 1.52751329247440503778 −2.27012801700149724482
O −0.07642332719784797335 3.34373141707827459257 1.67011778795091103689
O 1.21635422663176151481 1.06551645101728054144 1.73639598357549695962
H 1.58143084745013418058 2.16410476667903495240 −3.21949186788416241711
27
converged
O −0.07258678667811513385 −1.65662420584414804559 1.90926746758929888337
O 0.21063004184468286928 −2.20804107940959681855 −0.78343368724846973450
O −1.45924026382218374742 −3.39097515822494655069 0.72069046511072554217
P −1.57270033067614756206 −2.01239217793008418056 1.61806628758445403271
P −0.78175986487787918477 −3.42388005908996628790 −0.74453912422626444823
O −1.93441914603764453240 −3.06105246596661162428 −1.76607392278910291239
O −0.20899112321276430704 −4.74430985864158483167 −1.00529425101077163340
O −2.49109035546170254349 −2.24760677436348288438 2.73223572100198808243
O −1.89437721949856041626 −0.93245609817156593913 0.54494991910561985105
O −2.37797568034797102143 −0.46062520239401749667 −2.06061033575897001313
O 1.65558118616200999895 −0.11618335295170509336 3.68052742746131666962
H −1.49158048987591751455 −0.07162659592482145676 −2.15972305655231755495
H 1.94603459761929098448 0.59042844753631773536 3.09330633910270513809
H 1.06156539054058773885 −0.66482304232089517448 3.14974078934096146654
H −2.53114181336243104781 −0.40411864772655337052 −1.10316182110511329384
H −2.10954686860014772876 −2.08642441260298205918 −1.87121144348777224131
Re 0.16855233775478190017 −0.55812961086891910156 0.21231827282414381108
O 0.24677724146215390677 1.15753933498722805240 1.05349908361991029260
O 0.18319526991906032176 0.51614546197734556543 −1.65275712443571665133
O 1.67604170556735287967 2.34213949418305134031 −0.61627834709034390315
P 1.78445199887033534303 1.44655013738321946981 0.75251103361175020190
P 0.45895849871561167355 2.01532761630435297562 −1.63059218148836726492
O 1.19578362094036871888 2.31320602650037132619 −3.04671778215534727607
O −0.66851735517331600800 2.94845154668889231075 −1.47863409634778042445
O 2.53954866134755663509 2.19163446546408424709 1.76689116709083893220
O 2.25243524541088513402 0.05436593319172106087 0.31552687057381689772
H 0.85377150446300142939 3.15798028837772548627 −3.32860368724868349588
27
Coordinates from ORCA-job Tc_Felprot_s E −82.237384010820
O −0.22116603483224 −1.34186457819342 1.99369312066994
O 0.14940719809074 −1.72540821160477 −0.63735422040753
O −1.28076034474230 −3.26125669197188 0.75920796247113
P −1.62698467212269 −1.95894239026746 1.70080193233483
P −0.57406302277906 −3.11404577261817 −0.68772674092166
O −1.74901010215704 −2.95652222404689 −1.73167121772748
O 0.26115823232370 −4.28057374855730 −0.96364559168462
O −2.49010921689229 −2.38040984670488 2.80429989279245
O −2.13480411880754 −0.91700724317986 0.65616943475366
O −2.73994202539501 −0.50820517457874 −1.93771301870748
O 2.60723612906679 −1.06375045418489 2.45666263547916
H −1.95623498185547 0.05260183521963 −2.06456271819239
H 2.55950892280886 −0.33309484544001 1.82604357697117
H 1.69776492926082 −1.38512934841821 2.48018999515466
H −2.84119935764047 −0.52022465844892 −0.97126143578922
H −2.13733961494737 −2.04318293568032 −1.83325815879005
Tc −0.19299363275750 −0.15767031449628 0.34312128112377
O −0.39507724000737 1.49184532164603 1.18222563337379
O −0.38368059488811 0.86158140325579 −1.47282666682444
O 0.73980061293966 2.91441442758848 −0.53399224054841
P 1.02880799962903 2.13919732390886 0.87056137542659
P 0.55423001275792 2.01212275981198 −1.89030543700876
O 2.01419914121178 1.40532328182542 −2.10693358233116

O	0.11371020344929	2.84234913490174	−3.00980791448092
O	1.58542997063984	3.06742183847805	1.85471281359417
O	1.80461309526499	0.86289276354825	0.47065827621040
H	2.24689850479038	0.99203834249485	−1.25538898270199

27
Coordinates from ORCA–job Tc_Felprot_s_job10 E −82.234461908970

O	1.32117564759425	−1.57482949040829	1.35323941257353
O	0.45432553496319	−1.62217795525511	−1.18767853555115
O	−0.22747481118962	−3.37428209430750	0.49801811796480
P	−0.06869320491615	−2.20801087760453	1.63668363217441
P	−0.18822506543371	−3.04401633259420	−1.08753184642400
O	−1.69696203079675	−2.86990129142959	−1.52077296665878
O	0.48247986549842	−4.12218751846997	−1.81102972859582
O	−0.35865095698186	−2.79518585199291	2.95205575737149
O	−0.98980676573779	−1.06779271509972	1.09639759095954
O	−2.65558520671283	−0.47095059838699	−0.95885519672935
O	−2.28750011424421	−0.92380992706688	3.80423924739622
H	−2.01334296963199	0.15099812222806	−1.33987858100732
H	−1.68242403655465	−1.67674339006773	3.71113716386356
H	−2.10456825818373	−0.41875403467349	3.00626836297151
H	−2.32751927785013	−0.59360044947039	−0.05266155787155
H	−2.09848851281585	−1.97492614547478	−1.33361664932436
Tc	0.62295626906561	−0.19407882267214	0.03256767101844
O	0.84340872219804	1.33295482402582	1.06920789921133
O	−0.35005716203775	1.01911845862903	−1.36645651118399
O	1.11988274246559	2.98471548493536	−0.78754265146500
P	1.99684027278947	2.05354004570111	0.22913365033916
P	0.33074828115095	2.24092760490667	−2.01766484376370
O	1.52855096667940	1.70990898164443	−2.92887174518887
O	−0.54377440131131	3.17958070549430	−2.71848869452988
O	2.95593729230648	2.87930126077519	0.96458284145417
O	2.47196371408853	0.86131679558423	−0.62534213802060
H	2.10420347917485	1.21338520004535	−2.31523970732774

27
Coordinates from ORCA–job Tc_Felprot_s_job11 E −82.234669313970

O	−0.18657901195766	−1.76281028944915	2.63416002474608
O	0.48292422387413	−2.12033025123060	0.04918668875810
O	−1.24149909780295	−3.55217848750296	1.21017113010789
P	−1.59030877858454	−2.24257030051066	2.14677833567291
P	−0.35674502471654	−3.42807999676865	−0.13622432662736
O	−1.38060170129415	−3.12711471056464	−1.30362780815719
O	0.39367629092267	−4.66222055467653	−0.36118641543771
O	−2.62219077745766	−2.61371021856371	3.11650124469089
O	−1.86779896499353	−1.13937536807356	1.07834842310506
O	−2.03522299427019	−0.54849293475568	−1.56580843734696
O	−1.39970349685589	2.03900878702683	−3.39757131448785
H	−1.15082725001730	−0.14593593404615	−1.48689218431754
H	−0.53181152260360	2.23788343885376	−3.00759200963675
H	−1.72184251363826	1.27948545318189	−2.90231917428968
H	−2.33379864208827	−0.57977705573245	−0.64288643328912
H	−1.64518405026820	−2.17701965900701	−1.42449139028906
Tc	0.16186470181484	−0.55756526857285	1.05608245164386
O	−0.00903126005835	1.09909126786707	1.89248386362492
O	0.31541200053249	0.50496597339130	−0.74971029981213
O	1.45274476891028	2.45080482880950	0.38472715678958
P	1.49803677141641	1.60946618402939	1.79234733059166
P	1.37878614370722	1.58956878344607	−0.99673633157864
O	2.80687459486843	0.88667489948488	−1.05470653325924
O	1.12718300416595	2.46383631933584	−2.14678030270718
O	2.00028114133056	2.46846264566667	2.86598441934564
O	2.20305298277066	0.28496023179012	1.44310715510983

H 2.89170845470282 0.43747221615016 -0.19144525341866
27
Coordinates from ORCA-job Tc_Felprot_s_job12 E -82.234119445830
O -0.52247543717929 -0.06683001161992 2.56617220553503
O 0.68428116107805 -0.97541662714406 0.33883294743277
O -0.70424084325335 -2.46843027169894 1.82955459919094
P -1.60105702344028 -1.13158403203845 2.20473535397004
P 0.32851341248921 -2.46891112703938 0.59812817185384
O -0.48700667210869 -2.96208534819040 -0.66553149844350
O 1.45143917278119 -3.36884246283142 0.88799641661867
O -2.62757806512568 -1.51251520998508 3.17542644895959
O -2.00517048151568 -0.60814138609924 0.79048456974592
O -1.87185429048173 -1.07150426425898 -1.86190184623045
O 1.14657764360477 -5.53016781225336 -0.82315447755212
H -1.23466557420439 -0.34783866694269 -1.98968019358784
H 1.42313426750524 -4.88963648033090 -0.14435486259659
H 0.39652315697609 -5.08278198981667 -1.22289461919038
H -2.26182076358262 -0.86297166863630 -0.99642358589654
H -1.01625134316562 -2.26218299639001 -1.14754533408084
Tc -0.28507899341976 0.61615854219590 0.67478147764499
O -1.09787687202081 2.27722744805847 0.86190570147168
O -0.15100471341961 1.03182775040477 -1.37408478230273
O 0.15198683089356 3.49658607186769 -0.92654897451059
P 0.18102553343934 3.22063362218848 0.68381336291275
P 0.59065854952511 2.26916640542329 -1.92194343859476
O 2.14303018860445 2.09643900447012 -1.59725391737961
O 0.33611208347928 2.61755517413815 -3.31882110752271
O 0.18977535088864 4.49193415979545 1.41006063132960
O 1.29835061138671 2.18015634838373 0.89562583806443
H 2.18407312384232 2.00665582263685 -0.62547908260197
27
Coordinates from ORCA-job Tc_Felprot_s_job13 E -82.232296250020
O 0.52600574464144 -1.73601338634191 2.07423020348144
O 0.52417842631925 -1.77993123922624 -0.62464460334727
O -0.86186705489788 -3.37964623128905 0.75745424731625
P -0.94909715130654 -2.18788393213176 1.89279751952564
P -0.15281909304170 -3.18904189861923 -0.68388491685805
O -1.34813585669340 -3.07976934258872 -1.70874911753752
O 0.74037355333688 -4.30280404566810 -0.99792460312776
O -1.72914062034526 -2.68568341935080 3.02746447744208
O -1.48836992266309 -0.97461588525096 1.06492046387363
O -3.17627145404523 -1.33300892358381 -0.98831779825073
O -2.40270523773772 1.26374275315823 -0.76501075184439
H -2.97536180994997 -0.40982323950099 -1.24332508458754
H -1.49084965190318 1.32649237442573 -1.09684940401534
H -2.27744847629984 0.97449441363889 0.14531902310100
H -2.77542676947424 -1.37721352275054 -0.09897514477259
H -2.04678711247935 -2.39704901624242 -1.50183948287391
Tc 0.49948281804640 -0.35145634039887 0.59378948956260
O 0.57872973752666 1.17251470231721 1.66860784758691
O 0.29624313774857 0.96222336351051 -1.03128608990823
O 1.76670669619933 2.68734379746995 0.07057774305116
P 2.05675652831038 1.66000139164732 1.30908589369540
P 1.35413642994204 2.03190784070868 -1.37567300327370
O 2.69173011599461 1.28511217163878 -1.81775168810615
O 0.94483209135154 3.07321927983963 -2.31670541510676
O 2.81673890694920 2.34234898170938 2.35817461840465
O 2.59801516312084 0.37922271742692 0.64437631917985
H 2.91975086434314 0.71981662444750 -1.05396075424639
27
Coordinates from ORCA-job Tc_Felprot_s_job14 E -82.231295672220

O	1.23172759886657	−1.19782276674832	2.08172572856516
O	0.80893104241986	−1.56760008004423	−0.56256273447814
O	0.04689836973486	−3.22880712413667	1.18945405922537
P	−0.12543329471398	−1.94594486916852	2.21084200638202
P	0.24309596991564	−3.01799115400520	−0.39854725041765
O	−1.22743376187720	−2.98107192936137	−0.97974338543647
O	1.05410559050496	−4.10462234766619	−0.94904286680789
O	−0.59201895300730	−2.43800691971201	3.50848419991118
O	−1.02457275898034	−0.97810586464586	1.37368501018355
O	−2.21297618682419	−0.67948351066792	−1.68742193051322
O	−3.53486174451708	−1.50639773018925	0.62567466211614
H	−2.76760407606547	−0.62606941643467	−0.88945011921587
H	−3.31409853493756	−2.38650358840794	0.30746686229156
H	−2.70929949052727	−1.21340342821905	1.06470082892246
H	−1.49839346481052	−0.02635001179257	−1.56924481375775
H	−1.55042958654758	−2.07746117215300	−1.28862972323688
Tc	0.66000855309344	−0.03157049002282	0.52388097206359
O	0.60083149455884	1.58895588888374	1.44253967830614
O	−0.13996492270756	0.99891792127495	−1.11288117574495
O	1.08198200613899	3.11195294399311	−0.48171963761984
P	1.83041631542556	2.32718213257915	0.74412232756080
P	0.55992445809811	2.21930341190114	−1.75118379285354
O	1.92428365907412	1.71194153584626	−2.40590414301643
O	−0.24233397300735	3.03132540690208	−2.66412346230245
O	2.59663703952200	3.28006434561460	1.55017183534721
O	2.52095412038358	1.11846891918527	0.08245661807187
H	2.41902453907150	1.31359988619064	−1.66284975988992

27

Coordinates from ORCA–job Tc_Felprot_s_job15 E −82.231622075300

O	0.99537877135399	−1.28814290974309	2.44055241213059
O	0.76491993005245	−1.77142413891421	−0.20657434612881
O	−0.10570959102546	−3.36961811199760	1.55251977027197
P	−0.36338720901996	−2.04734287310893	2.49783398683300
P	0.32970819867270	−3.26331207590224	−0.00242429142141
O	−0.99955060894847	−3.47225961809672	−0.82184515863885
O	1.34288149906602	−4.26501354475667	−0.33169027001577
O	−0.91623892084115	−2.48741097998263	3.78003654613490
O	−1.20151542358396	−1.11793697049749	1.55881471781749
O	−2.33467925998300	0.31567673560024	−2.39829362258030
O	−2.88894676109591	−1.68554428305220	−0.46922599942450
H	−2.38652234475650	1.20576660286601	−2.75505014429978
H	−2.41092040544498	−1.35989915910923	0.31918672295144
H	−2.76153999437525	−1.01004117963232	−1.15436967686767
H	−1.49317177283592	0.34676731434422	−1.90177618811326
H	−1.73549422311611	−2.80334057100475	−0.69424465642758
Tc	0.53612345988170	−0.19859765817209	0.79982941976160
O	0.37560562963887	1.46271693753482	1.64229466558413
O	−0.11957617379280	0.73985802813982	−0.94377130997181
O	0.94024389515021	2.93601296647129	−0.29656497346664
P	1.63009958036347	2.20737230032374	1.00611074719708
P	0.55570119546273	1.98232682675152	−1.56112652827300
O	1.98327047769794	1.54255162131380	−2.11992812069178
O	−0.24913601046314	2.70741641016776	−2.54791458721860
O	2.31450075127064	3.21343308547120	1.82110236882363
O	2.39663867333251	1.00255323798268	0.42827194937621
H	2.44071664562407	1.17193200129016	−1.33985344497802

27

Coordinates from ORCA–job Tc_Felprot_s_job16 E −82.227319638420

O	0.62110325593082	−1.87633047486927	2.24966897937973
O	0.61719071118164	−2.23758038690566	−0.44123659371561
O	−0.84580298554594	−3.57394825196613	1.13474019356561

P	-0.89442627459048	-2.24283867493473	2.10232743117468
P	-0.36687633126343	-3.46364700100904	-0.40369550078254
O	-1.64204366917171	-3.04650470664043	-1.22438482223707
O	0.19625458383161	-4.74421573334945	-0.83686051412545
O	-1.69441836299174	-2.54148354452227	3.29161686392368
O	-1.31559645126253	-1.11944030516530	1.11351040021802
O	-1.87360223510577	-0.55936111315771	-1.94765035662841
O	-2.64562801338029	1.06745990075801	0.05911042672439
H	-0.94983210605976	-0.27770310446015	-1.84016274418183
H	-1.92926023527203	1.69895128950536	-0.13172526865174
H	-2.24061232915129	0.40106376484332	0.63680591064936
H	-2.34381769284163	-0.02506013519892	-1.26620222177816
H	-1.76040185930995	-2.06537128939877	-1.42642114262132
Tc	0.69905271285425	-0.69861498474693	0.63011696120955
O	0.80296946869864	0.95700214941128	1.47138905444963
O	0.60432740396091	0.41893426576560	-1.21380552263098
O	2.02430379330513	2.29672123093511	-0.21913889610858
P	2.31001042484388	1.34887049502730	1.09896059107138
P	0.71243792922930	1.93848661642698	-1.09576628431667
O	1.22595719600733	2.41310372584638	-2.55143181243711
O	-0.47710355379173	2.70044628761894	-0.69441942248631
O	3.09142566682931	2.11308172191095	2.07483082530647
O	2.80550339210092	0.01102852192126	0.55695133946387
H	1.90828556924920	1.80144973593315	-2.82522787548677

27

Coordinates from ORCA-job Tc_Felprot_s_job17 E -82.236923744980

O	0.08781300932145	-1.61479555607823	1.75526010926928
O	0.11060660953627	-1.85366585982261	-0.94177559254363
O	-1.17950850905155	-3.42367761057154	0.54873854589869
P	-1.36552733296744	-2.16806988499710	1.59885585383884
P	-0.69265871635791	-3.19911763486221	-0.97407468421785
O	-1.99831846320661	-2.90424069306776	-1.81492747118870
O	0.02341635924608	-4.37685218970179	-1.45788025208505
O	-2.10936089074721	-2.63201822735895	2.76929784949151
O	-1.95473568050506	-1.05420777344894	0.67936264893515
O	-2.86621080712154	-0.38059760289968	-1.75887139718352
O	1.84421863198857	-0.03982957928592	3.49759370637147
H	-2.08030354154481	0.15618140790881	-1.94932188007223
H	1.26358535654910	-0.62155780983868	2.98774144299010
H	1.94343866860284	0.75371610678947	2.96063853614316
H	-2.84186359407615	-0.45326016397294	-0.79315594298412
H	-2.34031850116623	-1.97248857938078	-1.77810641414290
Tc	-0.02978607014839	-0.35030840647366	0.17248508020935
O	-0.03212669528294	1.22829124835480	1.16642787547760
O	-0.39045841297670	0.84855352177973	-1.49752854785916
O	0.97763627511385	2.74595581674104	-0.54731417209509
P	1.38450027302926	1.80941860684178	0.72316269840385
P	0.55492002128250	1.98368596230119	-1.94079822980705
O	1.93135248565059	1.32398059342987	-2.40306536536359
O	0.02535743733677	2.93900548214285	-2.91195108180519
O	2.12226412684374	2.58437947428535	1.72530281295686
O	2.01135356588300	0.54316078044893	0.10935530664983
H	2.24011440305319	0.82285856502407	-1.62355143104804

27

Coordinates from ORCA-job Tc_Felprot_s_job18 E -82.224271103800

O	1.52160031876915	-1.21691683336121	1.86158457422754
O	0.51853172724021	-1.49087503223864	-0.63224146267797
O	0.15206033228662	-3.20812207968224	1.19954706998376
P	0.20639802928969	-1.94427593535275	2.27873040578130
P	-0.06036209593843	-2.91916447297828	-0.35652732611481
O	-1.64153522208699	-2.76886390286656	-0.50419139712884

O	0.49939910894764	−3.99409801646688	−1.18071275410064
O	0.05158308284978	−2.47857607462060	3.63159672618571
O	−0.82679742374112	−0.94484095580373	1.68297179671633
O	−1.85366036918159	−0.74103309462801	−2.21216903371339
O	−3.48018890180659	−1.93460006244341	1.61346920843543
H	−1.06045131618156	−0.99520907442636	−2.69141909005662
H	−2.69909880631904	−1.41978520554867	1.86551445349174
H	−3.13603305521334	−2.49481737069088	0.90960116914335
H	−1.55904906255839	0.02421130230109	−1.67509226522352
H	−1.85955010729674	−1.99038340658710	−1.07657004982768
Tc	0.65109017562135	0.01116935421517	0.52754789617606
O	0.84223524059035	1.61054842834461	1.46585292334328
O	−0.45716725108394	1.11601255106364	−0.87825955270593
O	0.96232487111402	3.16609265465298	−0.49429820487808
P	1.92161237503727	2.33138411296703	0.54075912147284
P	0.15132767858941	2.32070815267190	−1.63154517763759
O	1.32521920050471	1.75417422086908	−2.55894463061134
O	−0.78154751984492	3.17387828591039	−2.36529882689806
O	2.86639129103531	3.24576442142153	1.18525483220458
O	2.42365305050174	1.12412967935013	−0.27477768001930
H	1.96141465186830	1.35798833233957	−1.92848274778740

27

Coordinates from ORCA–job Tc_Felprot_s_job19 E −82.223584407170

O	0.57139995722872	−1.90310594281695	2.28531688239232
O	0.61730621856274	−2.32962607037179	−0.39184435034933
O	−0.93348620976924	−3.57202937601970	1.17395772223746
P	−0.95314977614426	−2.21865182354795	2.10927670728252
P	−0.41721875576710	−3.51293113982574	−0.35681561648017
O	−1.65610889936610	−3.05808615088124	−1.21270400421141
O	0.09663043707932	−4.82610416992521	−0.75058443716876
O	−1.79093481588522	−2.45743365247613	3.28611597269659
O	−1.30952428436856	−1.10448256407897	1.08507918502870
O	−1.78173390461267	−0.57659507254799	−1.99295989696342
O	−2.55960944447125	1.08695366882625	−0.01293423502642
H	−0.84658145264611	−0.33538868357963	−1.87894321577041
H	−1.80629314580972	1.68495964026138	−0.17152244826408
H	−2.20336786576775	0.40509313223535	0.57938908447193
H	−2.23790748109289	−0.02072680298415	−1.31914060081613
H	−1.72826616527289	−2.07805601629413	−1.43838743281973
Tc	0.73451313509292	−0.76789013481988	0.63975791386054
O	0.87964549105380	0.89900497079602	1.45070351606690
O	0.71321468852454	0.31307531499099	−1.23458091471697
O	2.17590712508352	2.15809301441980	−0.24071567949460
P	2.40973339258452	1.22852107665339	1.10457747589127
P	0.85915529509626	1.81783851287949	−1.09736556369553
O	1.33659477898859	2.34366831324336	−2.55304325519007
O	−0.32319675912020	2.60676355756748	−0.68756704807906
O	3.18921906175439	1.99845164994343	2.07779594279358
O	2.86596685837721	−0.14073422927635	0.61824712884043
H	0.73749251307680	3.05391898779129	−2.76920882298477

27

Coordinates from ORCA–job Tc_Felprot_s_job20 E −82.221513042600

O	−1.20310306531044	−2.04487792356853	2.27191970257279
O	−0.28628682557770	−2.38844291998133	−0.23999437151837
O	−2.33156138731186	−3.58734303830898	0.62803862880805
P	−2.59370266007731	−2.27892248306377	1.59702570977308
P	−1.26391936342720	−3.56007452546519	−0.58555634575600
O	−2.07295258145716	−3.09445390786043	−1.86307287139724
O	−0.67038518112076	−4.88456359532670	−0.76360553153249
O	−3.77591702209113	−2.53637348444511	2.42234070075863
O	−2.58180251814736	−1.09924854455101	0.58027325826118

O −2.39143024880932 −0.46569330568513 −2.03005660053731
O 3.56936584108725 3.23384592067195 −0.90453748138996
H −1.49178599187256 −0.11848263383225 −1.91717604815739
H 3.24897093626482 3.16909343082130 0.00059010313850
H 2.75594165424320 3.13408319919902 −1.43229610098042
H −2.72308439156652 −0.50272540058461 −1.11588767924561
H −2.20625165451033 −2.11132204533974 −1.95895641815074
Tc −0.47973740417985 −0.84638812500520 0.82210428442679
O −0.52725943180295 0.78491612923430 1.73162649614552
O 0.04726333583344 0.28924983689152 −0.90230928813528
O 1.32638813336939 1.96400896240821 0.54322312823916
P 1.04727014636474 1.02918457548496 1.87130419855676
P 1.18897895758496 1.28517700046072 −0.92748379609040
O 2.55366317607386 0.45757932888044 −1.08754083055835
O 1.12690595560927 2.37789411101448 −1.92335774375275
O 1.52898474012297 1.76522786623618 3.04484626272049
O 1.56203810413768 −0.36233192801255 1.51966757086432
H 3.28280874427200 1.08548349930634 −1.07922893282337
27
Coordinates from ORCA−job Tc_Felprot_s_job21 E −82.217810389580
O −0.97829259707238 −1.24389794872492 2.52849708101511
O 1.07193523552030 −2.08893612943276 0.99538896644472
O −0.72793834585374 −3.64921482329802 1.83187940360876
P −1.76431653894604 −2.36798019283127 1.76928239977857
P 0.72335930105184 −3.61136793723692 1.12154419543540
O 0.50342786816264 −4.13951786082635 −0.35092336074911
O 1.67048069326279 −4.43784388927423 1.86925213343666
O −3.07597427434468 −2.79995175161488 2.25439833658623
O −1.57832992682851 −1.85510562562016 0.31326921527322
O −1.71170519205141 3.85275896583546 −1.04728874840089
O −0.34509709294118 −2.30630608997972 −2.04638472114772
H −1.44221699606908 3.03952376096937 −1.50131984088357
H 0.25147103162600 −1.55735798361593 −1.86967789921594
H −1.09133153614886 −2.12128320076394 −1.45285749865710
H −1.31375378359712 3.77208428664007 −0.17316039044469
H 0.18087665807685 −3.46408083216489 −1.01443782809642
Tc −0.01047181008949 −0.55364649931924 0.91591689522138
O −0.87748995590352 1.06171581238020 0.63134999747796
O 0.98109986167508 −0.12794638828310 −0.97242134258461
O 0.97414983651345 2.36533654208085 −0.40971100601121
P 0.29942243614589 2.06622566108491 1.05683508431248
P 0.70995438400904 1.24788234589110 −1.54823269334443
O 1.96626922764901 1.53936656420925 −2.52683723351244
O −0.54021017493914 1.51551749910174 −2.28509503966685
O −0.10088181504617 3.34443110184660 1.65712125873910
O 1.23291607651443 1.09013036348967 1.75861260501410
H 1.63204743261690 2.12396423845246 −3.20309997597265
27
Coordinates from ORCA−job Tc_Felprot_s_job22 E −82.237002093200
O 0.19357007835420 −1.66389355675651 1.70127402865731
O 0.08734006624489 −1.81114660410620 −0.99736762512166
O −1.10756973999407 −3.45232386072966 0.49749876284512
P −1.25666792554835 −2.23735498781108 1.59926978967449
P −0.67655873633061 −3.17895990371788 −1.03409386264662
O −2.01618036311455 −2.90303037756266 −1.82562430493374
O 0.05752482060531 −4.32529044900010 −1.56631090505587
O −1.93194231056829 −2.75451381663458 2.78901141077809
O −1.90876420486163 −1.10177800260855 0.75133079161768
O −2.92490559589325 −0.41483453180341 −1.64928773234847
O 1.99953450046292 −0.11526107203852 3.40866370686370
H −2.16089838634812 0.15163635030408 −1.84802853535379

H	2.06018456229738	0.69713949463178	2.89532565819650
H	1.41096672191735	−0.69323900462605	2.90409826910807
H	−2.87256252783991	−0.51638227950110	−0.68451244375895
H	−2.37491079723669	−1.97400344598601	−1.77832589471614
Tc	−0.02451296262108	−0.34880253139633	0.17199767590271
O	−0.00538507839632	1.19444881768031	1.21793046535673
O	−0.48693276809149	0.89525755484889	−1.44416361711675
O	0.88964534721873	2.78708009372009	−0.48849758320378
P	1.37731791182354	1.81821153049746	0.72807081384976
P	0.41739026164890	2.06405738586167	−1.88625208716175
O	1.78372511337387	1.44810725207860	−2.43381778791202
O	−0.17325247783188	3.04080503520116	−2.79911845871031
O	2.14842625594210	2.57562116919116	1.71833585051072
O	1.99692730953167	0.58596007866005	0.04216140631015
H	2.13789092824830	0.92698967176583	−1.68766780855868

27

Coordinates from ORCA–job Tc_Felprot_s_job2 E −82.236998977720

O	0.10751089641849	−1.63325556537994	1.73794062510195
O	0.08820060361310	−1.83346755651919	−0.95887668223000
O	−1.19040170315251	−3.41975465923128	0.52620372211051
P	−1.35003461270029	−2.18006957049049	1.59826144419690
P	−0.69922975954004	−3.18773628432571	−0.99438079719402
O	−2.00375923011228	−2.90548827233394	−1.84015070566505
O	0.03206959546639	−4.35854391425233	−1.47445387037629
O	−2.07466160974804	−2.65927472900570	2.77477806725447
O	−1.95018445364609	−1.04887939684041	0.70714250089857
O	−2.87048303208348	−0.39980864097341	−1.74129541162536
O	1.89108580703083	−0.08028402211267	3.47171334932971
H	−2.08714443529231	0.14403236882047	−1.92738702358046
H	1.98739731179825	0.71972608382061	2.94438310908222
H	1.29877505453016	−0.65118727451590	2.96349351816302
H	−2.84860245147997	−0.48494640122780	−0.77356459819906
H	−2.34828772324982	−1.96958268942038	−1.82593812723014
Tc	−0.03272908477598	−0.34554779545096	0.17690786668170
O	−0.02005330657615	1.21954765685342	1.18997385172008
O	−0.41580382498503	0.87234966899858	−1.48060176343258
O	0.96290347725234	2.75908836976918	−0.51710959440070
P	1.38980393123722	1.80702828756552	0.73470141344339
P	0.52484106700530	2.01458602696143	−1.91558523835238
O	1.89770226021652	1.36331876365741	−2.40249037185662
O	−0.01599197262244	2.98227862341280	−2.86811237362944
O	2.14223281142539	2.57025277768291	1.73481725609923
O	2.00804408597094	0.54879879887761	0.09566517207490
H	2.21620030628419	0.85131933994727	−1.63413533414495

27

Coordinates from ORCA–job Tc_Felprot_s_job3 E −82.236254151410

O	0.82384258273070	−1.76353712360516	1.46234880611126
O	0.26816465910339	−1.79143618834010	−1.16830860998015
O	−0.70081289758166	−3.48874818297592	0.43998497961763
P	−0.62788222167783	−2.31220494982366	1.58311339485333
P	−0.49710121333736	−3.15701991638779	−1.13264756124263
O	−1.94020803150117	−2.85818086888673	−1.70005650560934
O	0.14456597116418	−4.28624281558716	−1.80295437705691
O	−1.09726492364551	−2.84077563761781	2.86766066266510
O	−1.40281279269299	−1.14045902926361	0.90511725905703
O	−2.79526216314772	−0.38727792268840	−1.27803835175384
O	−0.52680931395768	0.11799751768293	3.77988723723896
H	−2.06826595287241	0.18440317028766	−1.57643058581384
H	−0.70447891029143	−0.79866061968693	3.54152917108767
H	−0.13640718724385	0.51295868642865	2.99040594508442
H	−2.59258825829436	−0.52021534642041	−0.33724364719570

H	−2.28608498364847	−1.93171871891000	−1.56223975830638
Tc	0.37048995064843	−0.36975471773032	0.06081541453945
O	0.56179216518768	1.12018842267364	1.17174839348205
O	−0.35362939777289	0.92108079004833	−1.40527947753927
O	1.13499374861493	2.78753917515922	−0.60262954788664
P	1.84053294259631	1.78954903097856	0.48198519722494
P	0.46355255229133	2.11710106425862	−1.93813099933287
O	1.73208560016387	1.53817005762767	−2.71454605777058
O	−0.27024982849770	3.11614697320708	−2.71291392022043
O	2.75270668823017	2.54574291142578	1.34022842535719
O	2.34317416695531	0.58929639987949	−0.34328356161409
H	2.20335705146960	1.00055782726169	−2.04822192604855

27

Coordinates from ORCA–job Tc_Felprot_s_job4 E −82.236264844980

O	−0.61841115553418	−1.08771433577306	2.24960716882081
O	0.16601297689109	−1.66768963567073	−0.24481106685042
O	−1.44644686740135	−3.12963938631179	1.03510933791444
P	−1.95200713604362	−1.76501913294938	1.80672928901552
P	−0.51411141703465	−3.07788069762674	−0.28235108100136
O	−1.50106609802913	−3.05658241446030	−1.51405173607977
O	0.39441703743844	−4.22259742426248	−0.30810148986355
O	−2.96649351695572	−2.12765932800138	2.79736253833312
O	−2.31583092590118	−0.82764024192562	0.61222071789692
O	−2.49059540034495	−0.66644992944404	−2.07450603556586
O	2.98953456453579	−1.46229075771884	0.85313054024820
H	−1.70999005174550	−0.08925635403241	−2.12333228153888
H	2.60629235538381	−0.59807713698902	1.04913630970602
H	2.25092205709500	−1.97579097991357	0.51013429338443
H	−2.74930369240118	−0.59911895039625	−1.14002905097753
H	−1.88645018406844	−2.16731203915293	−1.75431405496283
Tc	−0.36314260903510	−0.03319163931127	0.54797676995874
O	−0.70702690000760	1.67493744299177	1.20325392017293
O	−0.27395316468485	0.82900736474078	−1.35564027762681
O	0.67231709311369	2.97639520687296	−0.42051851247917
P	0.74804942219744	2.31225575342981	1.06654886216179
P	0.69291503197035	1.97389167114872	−1.71773713958650
O	2.17994710082428	1.39533390049094	−1.68877964796345
O	0.39955548196413	2.71137014629257	−2.94522632714331
O	1.14362371078333	3.31963051758049	2.05163047707824
O	1.57704524015003	1.02125936110479	0.88443768276953
H	2.31359701808232	1.03432900299053	−0.79597920687344

27

Coordinates from ORCA–job Tc_Felprot_s_job5 E −82.235643682690

O	−0.36585175751052	−1.08484115707390	2.32184176284931
O	1.03247595435136	−1.81721911879057	0.14235851896092
O	−0.60958617092653	−3.36313288081559	1.27211843181683
P	−1.45183303164618	−2.02456696632198	1.71708954830929
P	0.58840672730809	−3.31727644034554	0.18742340914278
O	−0.08397515564826	−3.61196812173342	−1.21069147918635
O	1.61877258093807	−4.29541872525477	0.53133032845940
O	−2.60886827615594	−2.43182669916727	2.51437247537205
O	−1.66907372023418	−1.29899852945159	0.34665382988194
O	−3.34065544983985	1.02585477643942	0.24033242451346
O	−1.19358428894402	−1.49106049744167	−2.32995888664278
H	−3.05867994349275	0.10601544859274	0.33143739353178
H	−1.70229564355951	−1.35068990239429	−1.51601498761475
H	−0.51373523087256	−0.79755809107334	−2.28584647718775
H	−2.52859400199334	1.51630876092447	0.41516171973334
H	−0.50784269963379	−2.82885822716375	−1.66387724058958
Tc	0.14541863813136	−0.21473638857714	0.56912938203881
O	−0.57573561928026	1.48499908231413	0.87360554873807

O 0.54410164751625 0.44140028811246 -1.37805578796032
O 0.97326916209126 2.79462568228783 -0.58681793661410
P 0.78299402584247 2.32474436735245 0.96426179134458
P 1.43994493164198 1.66093684146488 -1.67732219751114
O 2.92268863122142 1.31130874735968 -1.20150080369278
O 1.39049971614262 2.19444665120273 -3.03693970732409
O 0.79189305141050 3.49001926693948 1.84956239813267
O 1.78308722664255 1.17014964864701 1.16380585279779
H 2.83615869420087 1.10184218883821 -0.25155931764333
27
Coordinates from ORCA-job Tc_Felprot_s_job6 E -82.235908648340
O -0.06095830496006 -1.15660466389846 2.52227825980265
O 0.93207852571591 -1.88311766786831 0.10164899181509
O -0.55580196658644 -3.38586108071229 1.48253086019605
P -1.27249920555846 -2.02693805831708 2.06642865821677
P 0.32938920999500 -3.33267907366525 0.13123034067575
O -0.69460700514399 -3.41641408690986 -1.06210194546985
O 1.29415474892002 -4.43253183576524 0.13450824821027
O -2.31741234604578 -2.39729432272665 3.02271281617381
O -1.63928963973155 -1.26113435805584 0.75615358372345
O -2.87650296686068 0.57488674737311 -0.79589982965997
O -1.39915194249840 -1.22114557357854 -2.32469676086668
H -2.12818201766805 1.17972310753498 -0.79030840773968
H -2.11701382185149 -0.73251428616917 -1.88077280148738
H -0.62434518685930 -0.65808052731615 -2.15895900353381
H -2.65630840943049 -0.06446158502781 -0.09662383623770
H -1.00735209926864 -2.55357362839688 -1.46466145183885
Tc 0.22727932515356 -0.26552747554982 0.74187573125131
O -0.37312037278431 1.44678211618728 1.20741659957645
O 0.38337692193713 0.51367259061918 -1.21284632215665
O 1.01884177624748 2.79772592388174 -0.36260341474196
P 1.01744455280686 2.22799998812579 1.17181478196044
P 1.28114994311488 1.71793240238386 -1.56737475418775
O 2.79919695776785 1.29018613650572 -1.32991904821787
O 1.05679108377462 2.33514814518813 -2.87355110299759
O 1.19611414165993 3.33408141995814 2.11432427469266
O 1.99304184651996 1.03523623363009 1.15793597368394
H 2.83308626521091 1.02900341214822 -0.38864044718685
27
Coordinates from ORCA-job Tc_Felprot_s_job7 E -82.235312750830
O -0.50984748731068 -0.91888246555488 1.98394324367764
O 0.64160812595635 -1.42626064203292 -0.39523711013815
O -1.43101137611299 -2.63732573459863 0.38249179502250
P -1.81110211354690 -1.26509147886635 1.21071226557795
P -0.25414745623152 -2.66231339650978 -0.73018888779491
O -0.95923380891544 -2.33578144767987 -2.10381400944715
O 0.39538879834458 -3.97164476290689 -0.73059871218807
O -3.06053553929187 -1.51653866389726 1.94266850253852
O -1.76167690399170 -0.17581513414947 0.10155035024457
O -3.72853230427196 -4.19979974776733 1.52584522994332
O -1.34730066114501 0.24391001863796 -2.53383541212513
H -3.65039444979188 -3.26195845682271 1.77389551023863
H -1.77632046400575 0.28436311480829 -1.66288567952359
H -0.48036767768238 0.65049196616396 -2.36665526765354
H -2.94647205893091 -4.33225057211293 0.98305818284644
H -1.11287443756469 -1.36890634349186 -2.30247775390832
Tc 0.28924037530723 0.20251117139278 0.48868723763328
O 0.12378825960921 1.87881102093087 1.27707905787650
O 0.91200482891019 1.19491692660741 -1.24517834698220
O 2.01777362217474 3.01509007353000 0.10613016164274
P 1.66932903249673 2.22180183273598 1.49187570955382

P 2.13700255681154 2.13258813208497 -1.27080170255416
O 3.43924749398754 1.24901353756149 -1.00554446703657
O 2.26829851975104 3.00382944044012 -2.43740047986899
O 2.02559072878212 3.04971307957210 2.64561171530192
O 2.27126470406562 0.81369794284106 1.31255223684671
H 3.27867969029794 0.82633059395457 -0.13958338135947
27
Coordinates from ORCA-job Tc_Felprot_s_job8 E -82.234934689070
O 0.12706360947245 -0.32826464938556 2.17191010928146
O 0.62394472904554 -1.11124901374667 -0.35724346174212
O -0.46507761817821 -2.62924478645161 1.34080814404453
P -1.10410987347081 -1.27070465440531 2.03382490790551
P 0.21042100701388 -2.59513886102924 -0.12488930447865
O -0.94217088421419 -2.91124798055378 -1.15215534181949
O 1.28431704093799 -3.59349811770354 -0.18237197848764
O -1.86836963746399 -1.67691593222003 3.21426607978781
O -1.81206106865953 -0.59227573777318 0.82166408411927
O -2.40719702366991 -0.81433982806838 -1.80408070770882
O 0.98607565542570 -5.14068813235712 2.15953786225422
H -1.76010834316655 -0.12925713637353 -2.04353816007472
H 0.35619035153135 -4.50796240161723 2.51598702804747
H 1.22287493749100 -4.74106643838510 1.30589757501338
H -2.53521324895737 -0.66673263965284 -0.85215962780063
H -1.50962741318159 -2.13747466731070 -1.43237155571401
Tc -0.06731321211788 0.50791129208533 0.33682220140464
O -0.63578818604037 2.19867434280846 0.85834642646301
O -0.42896286188622 1.10886577079567 -1.63575130658085
O 0.21830584098445 3.48760426224738 -1.10394008948392
P 0.63865703932701 3.05572711324640 0.41538664828970
P 0.26333517386878 2.33417958087447 -2.26815898535856
O 1.82297290249332 2.01507789478671 -2.38094668923065
O -0.30791051728114 2.83364767676903 -3.51779194670924
O 0.95888805236820 4.24751898879290 1.20314432158576
O 1.66573472644933 1.92031102626412 0.24323052603905
H 2.10452882487165 1.83104302265045 -1.46352676009868
27
Coordinates from ORCA-job Tc_Felprot_s_job9 E -82.234333237410
O 0.47691596742900 -2.24990746491193 1.85364023425076
O 0.26863375706557 -2.10653825075700 -0.83333614289317
O -1.06766802375461 -3.74587161006170 0.54478724580102
P -1.03127960717300 -2.64043758784956 1.76698192521550
P -0.64439183530868 -3.37258485192623 -0.96851891007254
O -1.96358767976762 -2.88240834840441 -1.68404693085971
O -0.04987423517813 -4.53472367346680 -1.62746407169965
O -1.71825090652283 -3.20138780778144 2.93145608155076
O -1.57498902119250 -1.35582762966415 1.06766335254925
O -2.57533384975987 -0.35325002183238 -1.24072662048389
O -1.42368789795502 2.72092026436149 0.74047617912895
H -2.52921276572064 -0.51717412362867 -0.28490038915111
H -1.00445100488238 2.80194897771090 -0.12198647195326
H -0.91973697936969 2.03482224764511 1.19522490774540
H -1.75371384645886 0.13296558412304 -1.41752027782374
H -2.21884272562356 -1.92763483200510 -1.53046147463149
Tc 0.36807831080909 -0.77376024586692 0.48844324115407
O 0.56942627532156 0.69652676840517 1.63569651396316
O 0.01083622087343 0.65016045081401 -1.01344615474047
O 1.61919813474174 2.29771104155687 0.03898548965570
P 2.01909882602361 1.18523023124485 1.17009405937674
P 1.02309762257854 1.75161140572553 -1.38441205517703
O 2.29559591965746 1.04977800189923 -2.04240491291084
O 0.49329137857851 2.86490938063617 -2.17246356506367

O	2.88669276554227	1.78690678701137	2.18289864155648
O	2.47288425142695	−0.04630447256590	0.36526991464686
H	2.61067095690433	0.42881977387559	−1.35802980489448

27

Coordinates from ORCA–job Tc_Felprot_s E −82.237384010820

O	−0.22116603483224	−1.34186457819342	1.99369312066994
O	0.14940719809074	−1.72540821160477	−0.63735422040753
O	−1.28076034474230	−3.26125669197188	0.75920796247113
P	−1.62698467212269	−1.95894239026746	1.70080193233483
P	−0.57406302277906	−3.11404577261817	−0.68772674092166
O	−1.74901010215704	−2.95652222404689	−1.73167121772748
O	0.26115823232370	−4.28057374855730	−0.96364559168462
O	−2.49010921689229	−2.38040984670488	2.80429989279245
O	−2.13480411880754	−0.91700724317986	0.65616943475366
O	−2.73994202539501	−0.50820517457874	−1.93771301870748
O	2.60723612906679	−1.06375045418489	2.45666263547916
H	−1.95623498185547	0.05260183521963	−2.06456271819239
H	2.55950892280886	−0.33309484544001	1.82604357697117
H	1.69776492926082	−1.38512934841821	2.48018999515466
H	−2.84119935764047	−0.52022465844892	−0.97126143578922
H	−2.13733961494737	−2.04318293568032	−1.83325815879005
Tc	−0.19299363275750	−0.15767031449628	0.34312128112377
O	−0.39507724000737	1.49184532164603	1.18222563337379
O	−0.38368059488811	0.86158140325579	−1.47282666682444
O	0.73980061293966	2.91441442758848	−0.53399224054841
P	1.02880799962903	2.13919732390886	0.87056137542659
P	0.55423001275792	2.01212275981198	−1.89030543700876
O	2.01419914121178	1.40532328182542	−2.10693358233116
O	0.11371020344929	2.84234913490174	−3.00980791448092
O	1.58542997063984	3.06742183847805	1.85471281359417
O	1.80461309526499	0.86289276354825	0.47065827621040
H	2.24689850479038	0.99203834249485	−1.25538898270199
